# Supplementary material for: Pincer-cobalt boosts divergent alkene carbonylation under tandem electro-thermo-catalysis
Source: Nat Commun. 2025 Oct 2;16:8803. doi: 10.1038/s41467-025-63875-4 (PMC12491415; doi:10.1038/s41467-025-63875-4)
Supplement: Supplementary file 1 — Supplementary Information [file 41467_2025_63875_MOESM1_ESM.pdf]

Supplementary information

Pincer-Cobalt Boosts Divergent Alkene Carbonylation under Tandem Electro-Thermo-Catalysis

Shulei Ge,[1,2‡] *Zhili Cui,*[1,2‡] *Lei Peng,*[3,4‡] *Xintong Wang,*[5] *Kaixin Chen,*[1,2] *Changrui Nie,*[1,2] *Shoucheng Dong,*[1,2] *Yang Huang,*[3,4] *Gen Luo,*[5]* *Lin He,*[3,4]* *and Jie Li*[1,2,6,7]*

[1]State Key Laboratory of Bioinspired Interfacial Materials Science, Soochow University, Suzhou, China

[2]College of Chemistry, Chemical Engineering and Materials Science, Soochow University, Suzhou, China

[3]State Key Laboratory of Low Carbon Catalysis and Carbon Dioxide Utilization, Lanzhou Institute of Chemical Physics (LICP), Chinese Academy of Sciences, Lanzhou, China

[4]State Key Laboratory for Oxo Synthesis and Selective Oxidation, Lanzhou Institute of Chemical Physics (LICP), Chinese Academy of Sciences, Lanzhou, China

[5]Institutes of Physical Science and Information Technology, Anhui University, 230601 Hefei, China

[6]Suzhou Key Laboratory of Pathogen Bioscience and Anti-infective Medicine, Soochow University, Suzhou, China

[7]MOE Key Laboratory of Geriatric Diseases and Immunology, Soochow University, Suzhou, China

E-mail: luogen@ahu.edu.cn; helin@licp.cas.cn; jjackli@suda.edu.cn

‡ These authors contributed equal

Contents

1. General Remarks······S-2
2. Optimization Studies······S-3
3. Mechanistic Experiments······S-6
4. Preparation of Starting Materials······S-12
    4.1 Preparation of Organozinc Pivalates······S-14
    4.2 Preparation of Alkenes······S-15
5. Substrate limitations······S-16
6. General Procedure for Divergent Radical Alkene Carbonylation······S-17
7. Scale-Up Experiments······S-20
8. Transformations of Products······S-21
9. Characterization Data······S-24
    9.1 Characterization Data of Alkenes······S-24
    9.2 Characterization Data of Poducts······S-26
10. Single Crystal X-Ray Diffraction Studies······S-76
11. DFT Calculations······S-78
12. NMR Spectra······S-80
13. References······S-207

1. General Remarks

All reactions were carried out under argon atmosphere and anhydrous conditions unless otherwise indicated. Syringes used to transfer reagents and solvents were purged with argon prior to use. Cobalt catalysts were obtained from commercial sources. Superdry solvents, THF, MeCN, 1,4-dioxane, MTBE were purchased form commercial sources. Dry toluene and Et_2O was continuously refluxed and freshly distilled from sodium benzophenone ketyl under nitrogen. Yields refer to isolated compounds, estimated to be > 95% pure as determined by ^{1}H-NMR. Reactions were monitored by thin layer chromatography (TLC). TLC were performed using aluminum plates covered with SiO_2 (Merck 60, F-254) and visualized by UV detection. Purification *via* column chromatography was performed using Merck silica gel 60 (40–63 mm 230–400 mesh ASTM from Merck). NMR spectra were recorded in $CDCl_3$ (δ = 7.26) and chemical shifts (δ) are reported in parts per million (ppm). High-resolution mass spectra (HR-MS) were recorded on an Agilent 1290 mass spectrometer using ESI-TOF (electrospray ionization time-of-flight).

2. Optimization for Cobalt-Catalyzed Alkene Sulfonylcarbonylation.

Table S1. Ligand Screening for Cobalt-Catalyzed Alkene Sulfonylcarbonylation.[a]

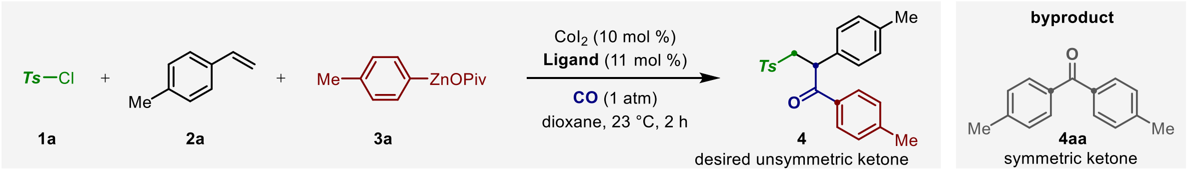

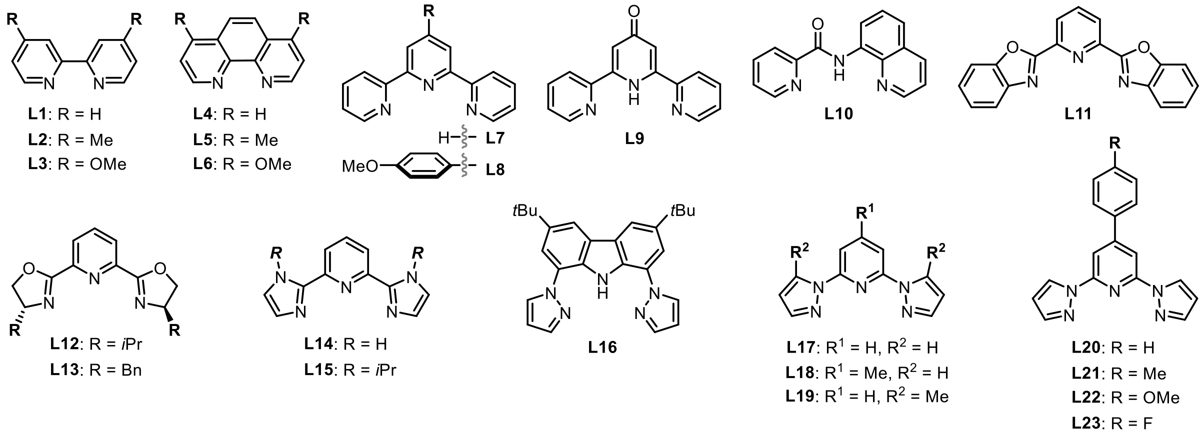

Entry	Ligand	Yield of **4a** (**4aa**) (%)[b]
1	**L1**	0 (93)
2	**L2**	2
3	**L3**	0
4	**L4**	3 (91)
5	**L5**	0
6	**L6**	2
7	**L7**	3
8	**L8**	4
9	**L9**	3 (92)
10	**L10**	0 (92)
11	**L11**	0
12	**L12**	7 (85)
13	**L13**	6
14	**L14**	0
15	**L15**	0
16	**L16**	0
17	**L17**	**75**
18	**L18**	64
19	**L19**	0
20	**L20**	63
21	**L21**	55
22	**L22**	67
23	**L23**	42

[a] Reaction conditions: **1a** (0.2 mmol, 2.0 equiv), **2a** (0.1 mmol, 1.0 equiv), **3a** (0.2 mmol, 2.0 equiv), [CoI_2] (10 mol %), ligand (**L**, 12 mol %), CO (1 atm), dioxane (1.0 mL), at 23 ℃, 2 h. [b] Isolated yields.

Table S2. Arylzinc Reagent Screening for Cobalt-Catalyzed Alkene Sulfonylcarbonylation.

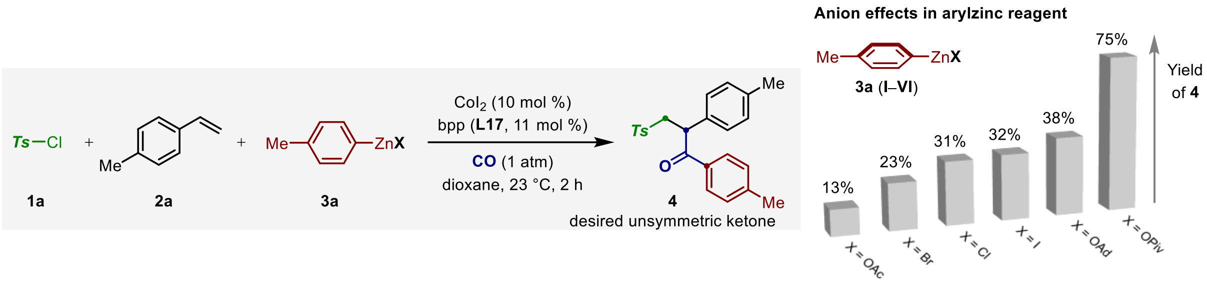

Entry	X	Yield of **4** (%)[b]
1	OAc	13
2	Br	23
3	Cl	31
4	I	32
5	OAd	38
6	OPiv	**75**

[a] Reaction conditions: **1a** (0.2 mmol, 2.0 equiv), **2a** (0.1 mmol, 1.0 equiv), **3a** (0.2 mmol, 2.0 equiv), CoI_2 (10 mol %), ligand (**L17**, 12 mol %), CO (1 atm), solvent (1.0 mL), at 23 ℃, 2 h. [b] Isolated yields.

Table S3. Solvent Screening for Cobalt-Catalyzed Alkene Sulfonylcarbonylation.[a]

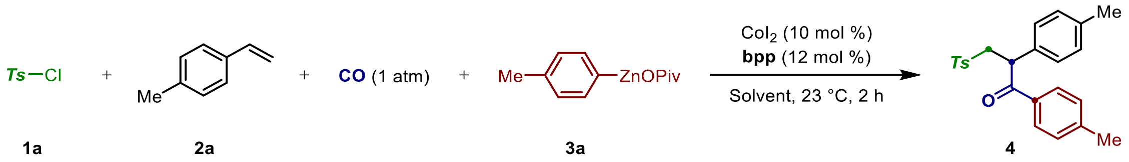

Entry	Solvent	Yield (%)[b]
1	THF	40
2	MeCN	35
3	Anisole	21
4	Dioxane	**75**
5	DME	33
6	DEDM	27
7	Et_2O	trace
8	DMA	0
9	$^nBu_2O$	trace
10	MeO^tBu	trace
11	Toluene	trace
12	DCE	17
13	DCM	24
14	DMSO	0

[a] Reaction conditions: **1a** (0.2 mmol, 2.0 equiv), **2a** (0.1 mmol, 1.0 equiv), **3a** (0.2 mmol, 2.0 equiv), CoI_2 (10 mol %), ligand (**L17**, 12 mol %), CO (1 atm), solvent (1.0 mL), at 23 ℃, 2 h. [b] Isolated yields.

Table S4. Catalyst Screening for Cobalt-Catalyzed Alkene Sulfonylcarbonylation.[a]

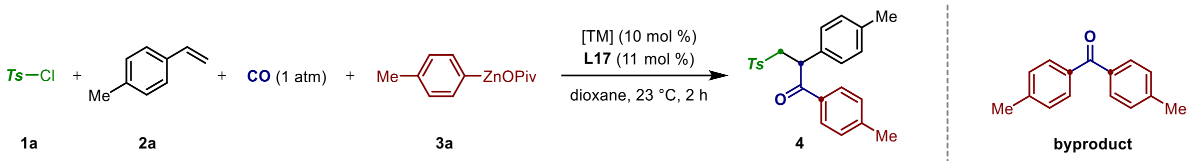

Entry	Catalyst	Yield (%)[b]
1	CoI_2 as the catalyst	**75**
2	$CoCl_2$ as the catalyst	21
3	$CoBr_2$ as the catalyst	trace
4	$Co(OAc)_2$ as the catalyst	50
5	$Co(PPh_3)_2Cl_2$ as the catalyst	26
6	$Co(acac)_2$ as the catalyst	52
7	$Co(acac)_3$ as the catalyst	trace
8	$Co(PPh_3)_3Cl$ as the catalyst	trace
9	5 mol % CoI_2 was used	62
10	2.5 mol % CoI_2 was used	40
11	$NiCl_2$ as the catalyst	0
12	$NiCl_2(PPh_3)_2$ as the catalyst	0
13	$NiCl_2(PCy_3)_2$ as the catalyst	0
14	$NiBr_2(PCy_3)_2$ as the catalyst	0
15	$NiCl_2(DME)$ as the catalyst	0
16	$Ni(OTf)_2$ as the catalyst	0
17	$Ni(acac)_2$ as the catalyst	0
18	$Ni(OAc)_2$ as the catalyst	0
19	NiI_2 as the catalyst	0
20	$Fe(OTf)_2$ as the catalyst	0
21	$Fe(OTf)_3$ as the catalyst	0
21	$FeCl_3$ as the catalyst	0
23	$FeBr_3$ as the catalyst	0
24	$Fe(acac)_3$ as the catalyst	0
25	$FeCl_2$ as the catalyst	0
26	$Cu(OTf)_2$ as the catalyst	0
27	CuI as the catalyst	0

[a] Reaction conditions: **1a** (0.2 mmol, 2.0 equiv), **2a** (0.1 mmol, 1.0 equiv), **3a** (0.2 mmol, 2.0 equiv), catalyst (10 mol %), ligand (**L17**, 12 mol %), CO (1 atm), dioxane (1.0 mL), at 23 ℃, 2 h. [b] Isolated yields.

3. Mechanistic Experiments

a) Radical Inhibition Experiments

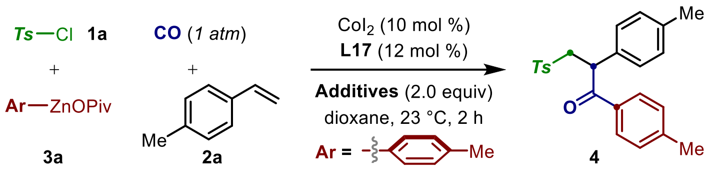

Additives:	w/o additive	TEMPO	1,1-Diphenylethylene
Yield of **4**:	75%	0%	19%

Figure S1. Radical inhibition experiments.

Procedure for Figure S1: An oven-dried tube was charged with CoI_2 (10 mol %), **L17** (12 mol %), tosyl chloride **1a** (0.4 mmol, 2.0 equiv). Then the tube was evacuated and backfilled with CO (three times, 1 atm, balloon). 1,4-dioxane (1.0 mL) was added and stirred for 10 minutes vigorously. Then alkene **2a** (0.2 mmol, 1.0 equiv), and additive (TEMPO (2.0 equiv), or 1,1- diphenylethene (2.0 equiv) were added. Then **3a** (0.4 mmol, 2.0 equiv) resolved in 1,4-dioxane (0.5 mL) were added dropwise over 5 minutes. The reaction mixture was stirred at 23 °C for 2 h. When the reaction was completed, product **4** not detected with TEMPO, only a portion of product **4** was detected with 1,1- diphenylethene by TLC. The resulting residue was purified by column chromatography on silica gel (petroleum ether/EtOAc 6:1) to yield product **4**.

b) EPR Experiments

An oven-dried tube was charged with CoI_2 (10 mol %), **L17** (12 mol %), tosyl chloride **1a** (0.3 mmol, 2.0 equiv), 1,4-dioxane (1.0 mL) and alkene **2a** (0.15 mmol, 1.0 equiv) and **DMPO** (2.0 equiv) were added in glovebox and stirred for 5 minutes. Then **3a** (0.45 mmol, 3.0 equiv) resolved in 1,4-dioxane (0.5 mL) were added and stired for 10 minutes. A series of EPR spintrapping experiments show the existence of S-centered radicals trapped by **DMPO** (A_N = 13.81 G, A_H = 18.61 G), which was considered to be $\bullet SO_2Ar$.

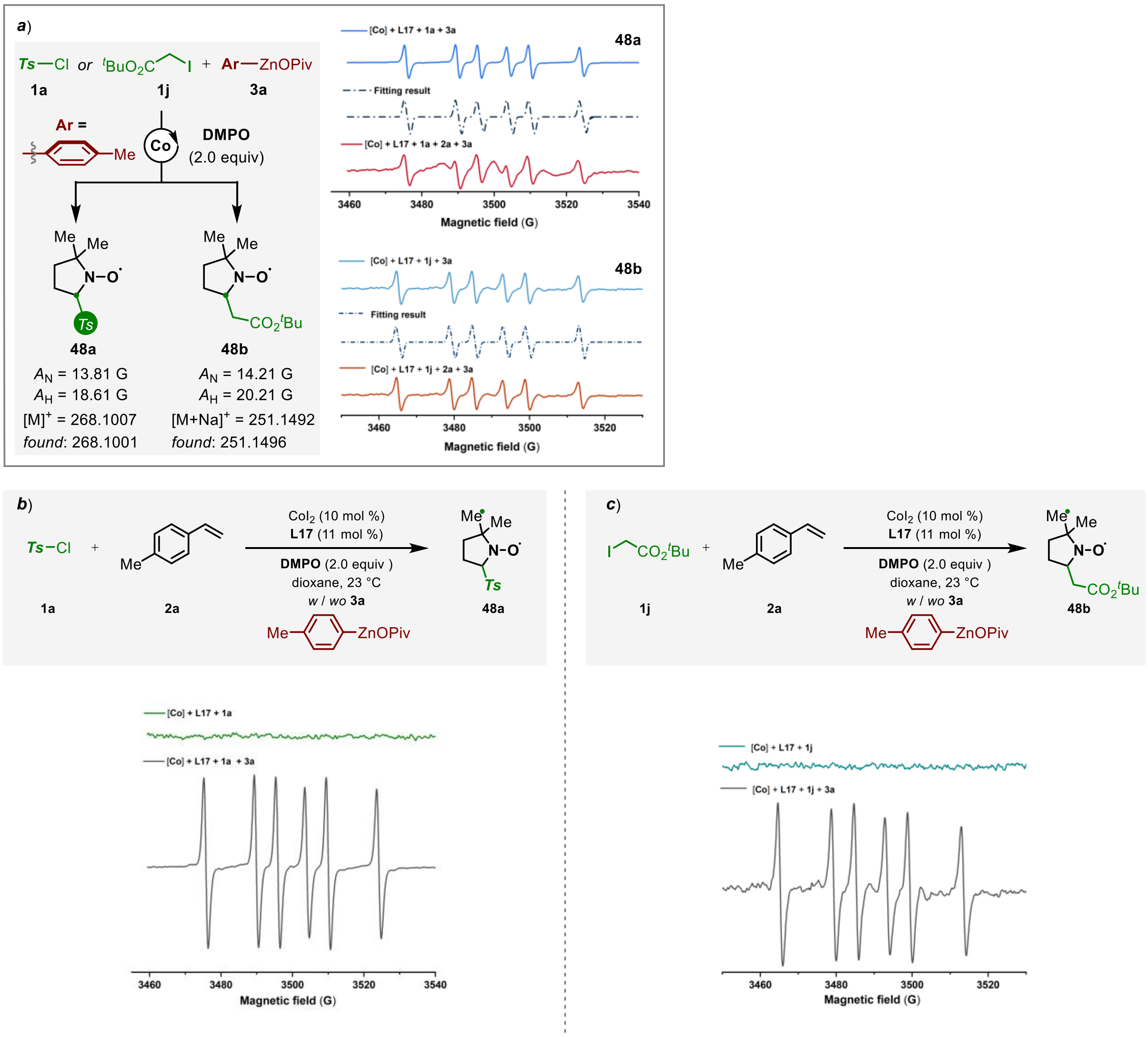

Figure S2. EPR experiments.

c) Catalytic Reactivity of the Pincer-Cobalt Complex 49

i) Preparation of Cobalt(II) Complex 49[1]

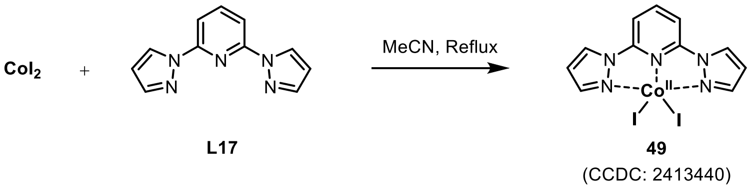

Figure S3. Preparation of cobalt(II) complex **49**.

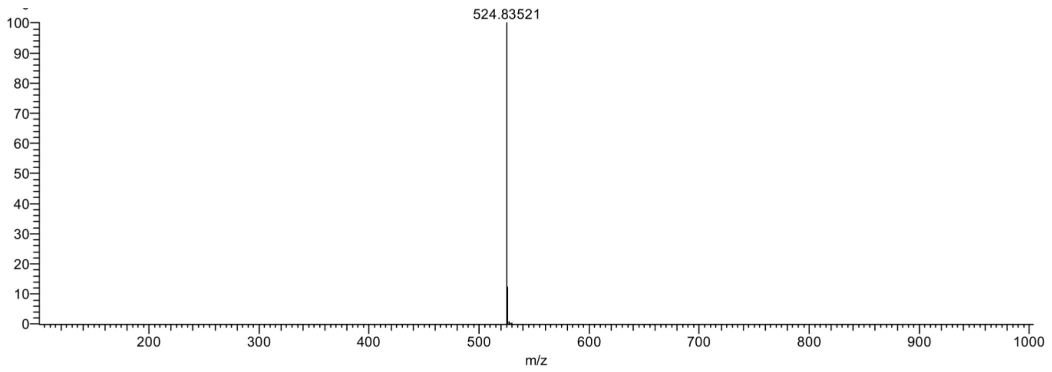

Figure S4. HR-MS spectra of cobalt(II) complex **49**.

An oven-dried tube was charged with cobalt(II) iodide (468.2 mg, 1.5 mmol) and acetonitrile (50 mL), then a solution of **L17** (316.5 mg,1.5 mmol) in acetonitrile (50 mL) was added. The reaction mixture was refluxed for 30 min. Cooling the mixture to room temperature, the green precipitate collected by filtration (Yield: 558.9 mg, 71%). Single crystals were obtained by vapour diffusion of diethyl ether into a THF solution of the complex. HR-MS (ESI) m/z calcd for $C_{11}H_9CoI_2N_5$ [M+H$^+$] 524.83521, found 524.83521.

ii) Catalytic Reactivity of the Pincer-Cobalt Complex 49

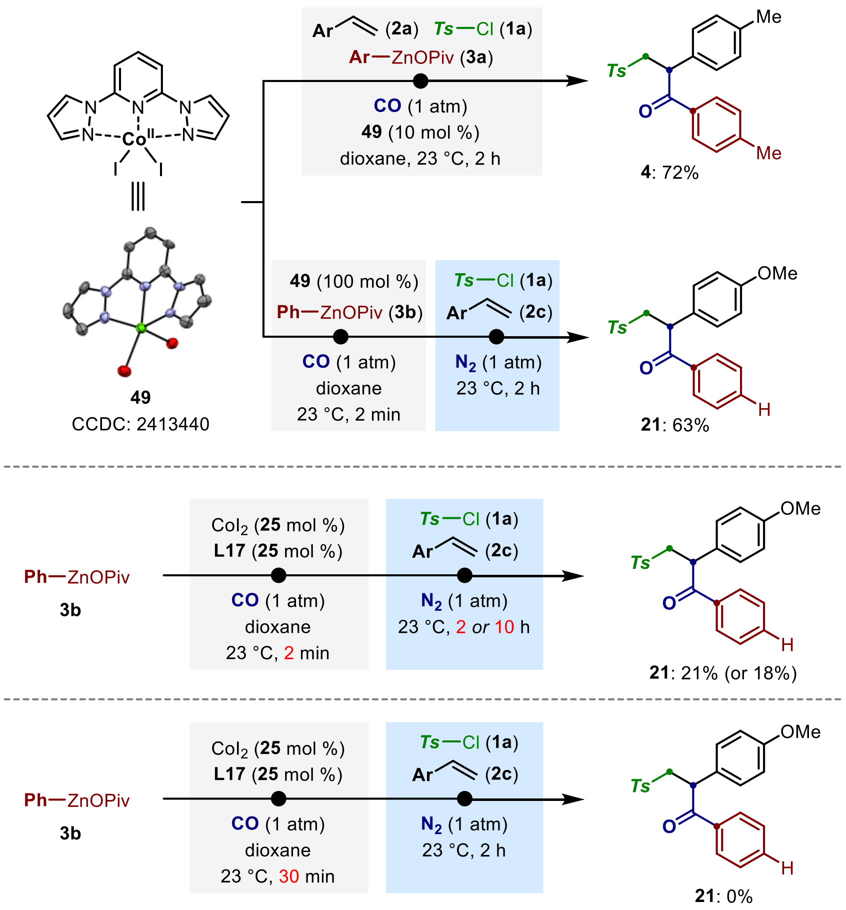

Figure S5. Catalytic reactivity of the pincer-cobalt complex 49.

Procedure for synthesis of 4: an oven-dried tube was charged with **49** (10 mol %), tosyl chloride **1a** (0.4 mmol, 2.0 equiv). Then the tube was evacuated and backfilled with CO (three times, 1 atm, balloon). 1,4-dioxane (1.0 mL) was added and stirred for 2 minutes vigorously. Alkene **2a** (0.2 mmol, 1.0 equiv) was added. Then **3a** (0.4 mmol, 2.0 equiv) resolved in 1,4-dioxane (1.0 mL) were added dropwise over 5 minutes. The reaction mixture was stirred at 23 °C for 2 h. When the reaction was completed, the resulting residue was purified by column chromatography on silica gel (petroleum ether/EtOAc 6:1) to yield product **4**.

Procedure for synthesis of 21: an oven-dried tube was charged with **49** (100 mol %), Then the tube was evacuated and backfilled with CO (three times, 1 atm, balloon), 1,4-dioxane (0.5 mL) was added and stirred for 2 minutes vigorously. Then **3b** (0.25 mmol, 5.0 equiv) resolved in 1,4-dioxane (0.5 mL) was added in one portion. The red mixture was stirred at 23 °C for 2 minutes. Then the mixture was added to another schlenk tube charged with tosyl chloride **1a** (0.1 mmol, 2.0 equiv) and alkene **2c** (0.05 mmol) in one portion under an atmosphere of argon. The reaction was stirred for 2 h at room temperature. The resulting residue was purified by column chromatography on silica gel (petroleum ether/EtOAc 6:1) to yield **21** as a colourless oil.

d) Reactivity of the *in situ* Formed Carbonyl Metal Species

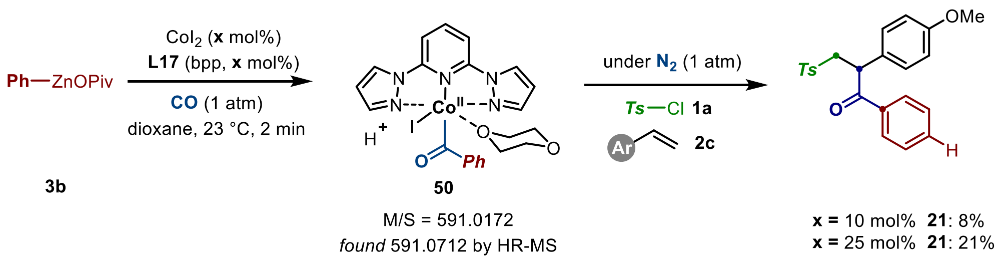

Figure S6. Reactivity of the in situ formed carbonyl metal species.

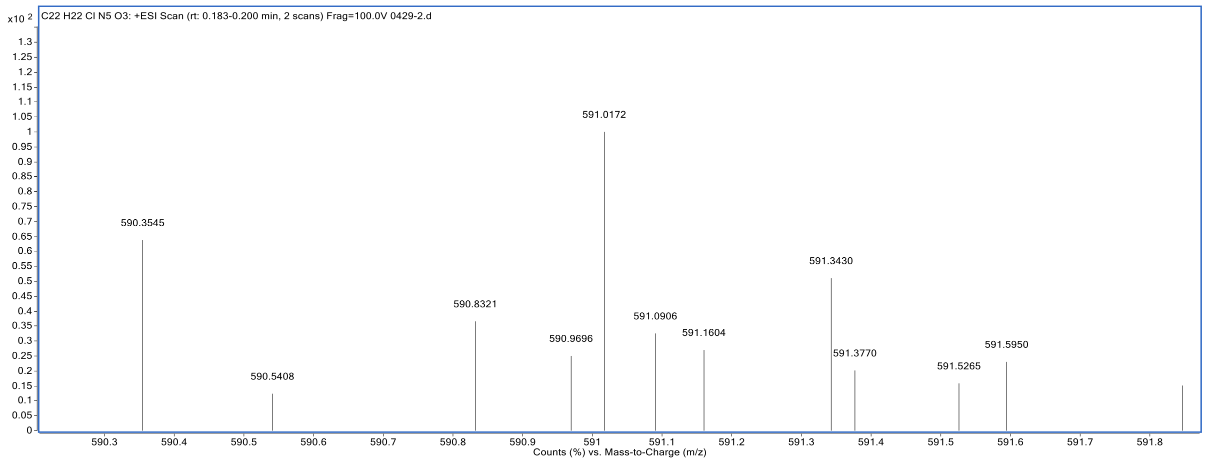

Figure S7. HR-MS spectra of **50**.

Procedure for Figure S6: An oven-dried tube was charged with CoI_2 (10 mol % or 25 mol %), **L17** (11 mol % or 27 mol %), Then the tube was evacuated and backfilled with CO (three times, 1 atm, balloon), 1,4-dioxane (0.5 mL) was added and stirred for 10 minutes vigorously. Then **3b** (0.4 mmol, 2.0 equiv) resolved in 1,4-dioxane (0.5 mL) was added in one portion. The red mixture was stirred at 23 °C for 2 minutes. After that, the yelow solution was analyzed by ESI-HRMS. Then the mixture was added to another schlenk tube charged with tosyl chloride **1a** (0.4 mmol, 2.0 equiv) and alkene **2c** (0.2 mmol) in one portion under an atmosphere of argon. The reaction was stirred for 2 h at room temperature. The resulting residue was purified by column chromatography on silica gel (petroleum ether/EtOAc 6:1) to yield **21** as a colourless oil.

e) Competition Experiment between Organozinc Pivalates

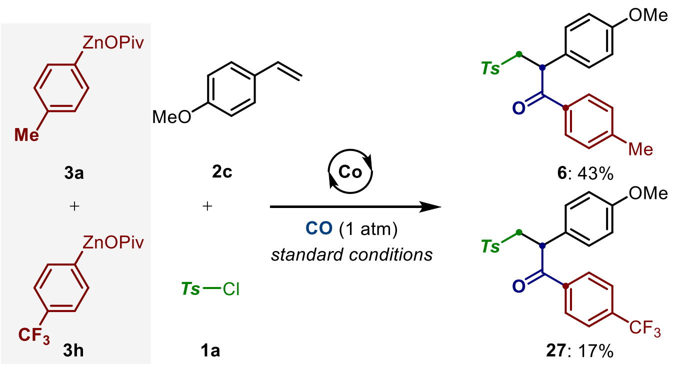

Figure S8. Competition experiment between organozinc pivalates **3a** and **3h**.

Procedure for Figure S8: An oven-dried tube was charged with CoI_2 (10 mol %), **L17** (12 mol %), tosyl chloride **1a** (0.2 mmol, 1.0 equiv). Then the tube was evacuated and backfilled with CO (three times, 1 atm, balloon). Alkene **2c** (0.2 mmol, 1.0 equiv) was addded. 1,4-dioxane (0.4 mL) was added and stirred for 10 minutes vigorously. Then **3a** (0.2 mmol, 1.0 equiv) and **3h** (0.2 mmol, 1.0 equiv) resolved in 1,4-dioxane (1.0 mL) were added simultaneously dropwise over 5 minutes. The reaction mixture was stirred at 23 °C for 2 h. When the reaction was completed, the resulting residue was purified by column chromatography on silica gel (petroleum ether/EtOAc 6:1) to yield products.

f) Competition Experiment between Alkenes 2c and 2f

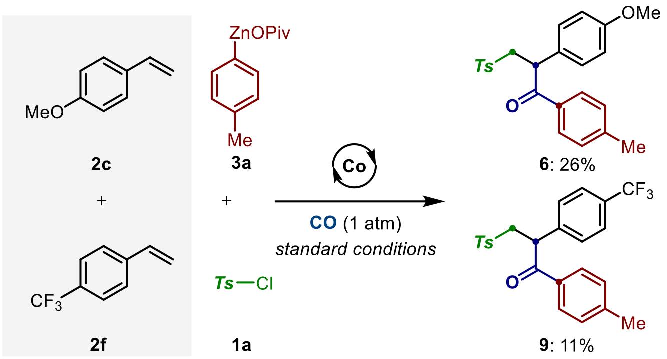

Figure S9. Competition experiment between arylalkene **2c** and **2f**.

Procedure for Figure S9: An oven-dried tube was charged with CoI_2 (10 mol %), **L17** (12 mol %), tosyl chloride **1a** (0.2 mmol, 1.0 equiv). Then the tube was evacuated and backfilled with CO (three times, 1 atm, balloon). Alkene **2c** (0.2 mmol, 1.0 equiv) and alkene **2f** (0.2 mmol, 1.0 equiv) were added. 1,4-dioxane (0.4 mL) was added and stirred for 10 minutes vigorously. Then **3a** (0.2 mmol, 1.0 equiv) resolved in 1,4-dioxane (1.0 mL) were added simultaneously dropwise over 5 minutes. The reaction mixture was stirred at 23 °C for 2 h. When the reaction was completed, the resulting residue was purified by column chromatography on silica gel (petroleum ether/EtOAc) to yield products.

g) Radical Clock Experiment with 107

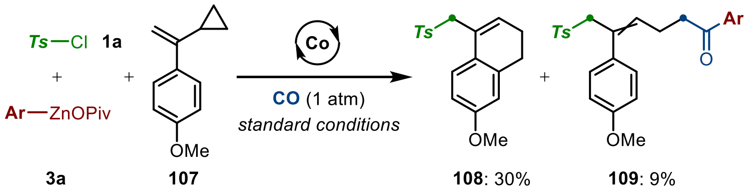

Figure S10. Radical clock experiment with **107**.

Procedure for Figure S10: An oven-dried tube was charged with CoI_2 (10 mol %), **L17** (12 mol %), tosyl chloride **1a** (0.4 mmol, 2.0 equiv). Then the tube was evacuated and backfilled with CO (three times, 1 atm, balloon). 1-(1-cyclopropylvinyl)-4-methoxybenzene **107** (0.2 mmol, 1.0 equiv) was added. 1,4-dioxane (1.0 mL) was added and stirred for 10 minutes vigorously. Then **3a** (0.4 mmol, 2.0 equiv) resolved in 1,4-dioxane (0.5 mL) were added dropwise over 5 minutes. The reaction mixture was stirred at 23 °C for 2 h. When the reaction was completed, the resulting residue was purified by column chromatography on silica gel (petroleum ether/EtOAc 6:1) to yield products.

4. Preparation of Starting Materials

4.1 Preparation of Solid Arylzinc Pivalates

The arylzinc pivalates were prepared according to **TP1 (Method A)**, while arylzinc pivalates **3i** and **3j** were prepared according to **TP1 (Method B)**.[2]

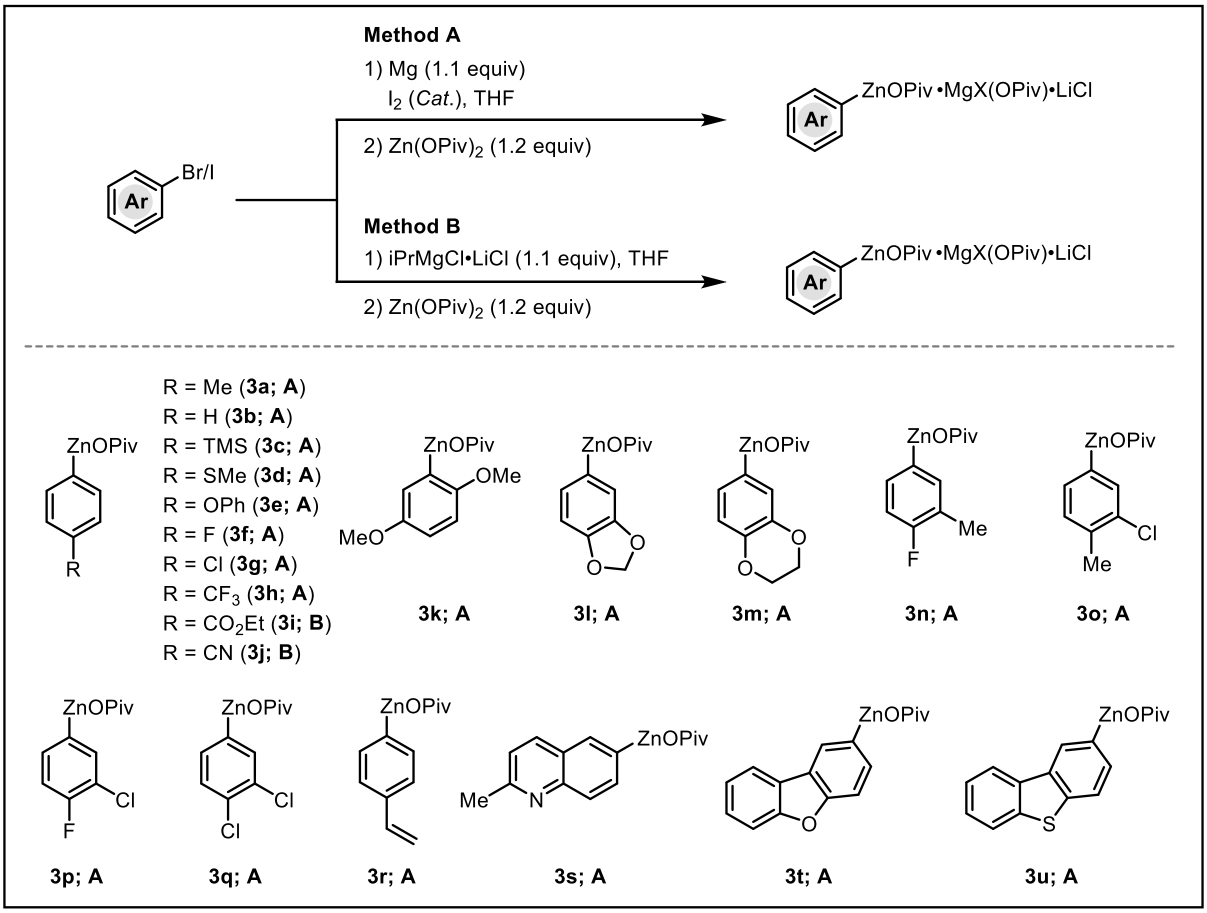

Figure S11. List of solid zinc pivalates.

Preparation of Zn(OPiv)$_2$: Pivalic acid (10.2 g, 100 mmol) was placed in a dry and nitrogen-flushed 250 mL three-necked roundbottom flask, equipped with a magnetic stirring bar, a septum and a pressure equalizer, and was dissolved in dry THF (30 mL). The mixture was cooled to 0 °C, and a solution of Et_2Zn (50 mL, 50 mmol, 1.0 M in hexane) was added over a period of 30 minutes under vigorous stirring. Then, the ice-bath was removed and stirring was continued at 25 °C for one additional h at which point bubbling has ceased (a thick slurry was formed). The solvent was removed in vacuo and the solid residue was dried for at least 4 h longer. $Zn(OPiv)_2$ was obtained in quantitative yield as a puffy amorphous white solid.

Preparation of Zn(OAd)$_2$: 1-adamantaneacetic acid (1.94 g, 10 mmol) was placed in a dry and

nitrogen flushed 100 mL three-necked roundbottom flask, equipped with a magnetic stirring bar, a septum and a pressure equalizer, and was dissolved in dry THF (5.0 mL). The mixture was cooled to 0 °C, and a solution of Et_2Zn (5.2 mL, 5.2 mmol, 1.0 M in hexane) was added over a period of 30 minutes under vigorous stirring. Then, the ice-bath was removed and stirring was continued at 25 °C for one additional h at which point bubbling has ceased (a thick slurry was formed). The solvent was removed in vacuo and the solid residue was dried for at least 4 h longer. $Zn(OAd)_2$ was yielded (1.8 g, 80%) as a puffy amorphous white solid.

Typical procedure 1 (TP1) for the preparation of organozinc pivalates:

Method A: A dry argon flushed two neck Schlenk-flask equipped with a magnetic stir bar and a septum was charged with magnesium turnings (57.6 mg, 2.4 mmol, 1.2 equiv) and heated with a heat gun under high vacuum for 5 minutes. After cooling to room temperature anhydrous THF (2.0 mL) was added and magnesium turnings were activated using iodine (a small crystal). The corresponding aryl bromide was added slowly over a period of 2 minutes to keep the reaction refluxing. Then the reaction mixture was refluxed for another 40 minutes. The solids were allowed to settle and the yield of the insertion reaction was determined by iodometric titration of the supernatant solution. Next, this solution was added via syringe into a 50-mL two-necked round-bottomed flask equipped with a magnetic stirring and $Zn(OPiv)_2$ (1.2 equiv. with respect to titrated Grignard reagent) at 0 °C, and the reaction mixture was stirred at room temperature for 10 minutes. The residual THF was removed under reduced pressure resulting in the corresponding solid organozinc pivalates reagents.

Method B: A dry argon flushed two neck Schlenk-flask equipped with a magnetic stir bar and a septum was charged with aryl iodides (2.0 mmol, 1.0 equiv, 1.0 M) in 2 mL THF, then *i*-PrMgCl·LiCl (1.1 equiv, 1.2 M) was added dropwise to the reaction mixture under -15 °C. The reaction was stirred until iodolysis and protolysis of a reaction aliquot indicated full consumption of the starting material. Thereafter, $Zn(OPiv)_2$ (1.2 equiv) was added to the reaction mixture at 0 °C, and the reaction mixture was stirred at room temperature for 10 minutes. The residual THF was removed under reduced pressure resulting in the corresponding solid organozinc pivalates reagents.

4.2 Preparation of Alkenes

Alkenes **2a-2i, 2k, 2n-2q, 2ac-2ad** were all purchased from commercial sources. The alkenes **2l, 2u-2w, 2y, 2ab** derived from drug-like molecules were prepared according to the **TP2**.

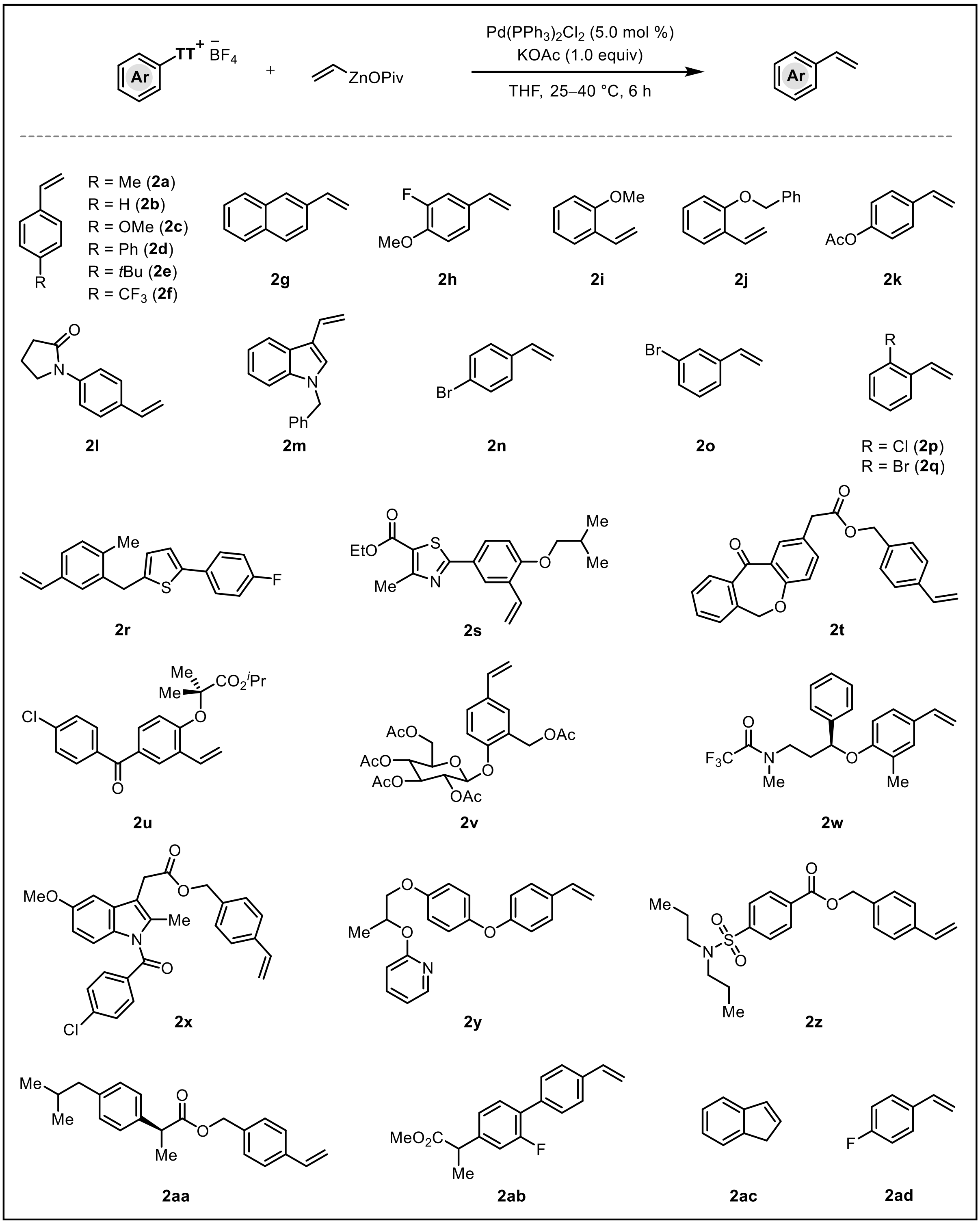

Figure S12. List of alkenes.

Typical procedure 2 (TP2) for the preparation of alkenes:[3]

A 10 ml Schlenk-tube, equipped with a magnetic stir bar, and fitted with a septum, was charged with $Pd(PPh_3)_2Cl_2$ (5 mol %), thianthrenium salt (1.0 equiv), and KOAc (4.0 equiv). Then the

tube was evacuated and backfilled with argon. THF solution of vinyl zinc pivalates (3.0 equiv) was slowly added to the mixture while stirring at 25 °C. Subsequently, the mixture was heated to 40 °C, and stirred for 6 h at 40 °C. When the reaction was completed, the residue was purified by chromatography on silica gel (petroleum ether/EtOAc) to afford alkenes.

Note: All sulfonyl chlorides and alkyl halides **1a**–**1aa** were purchased from commercial sources.

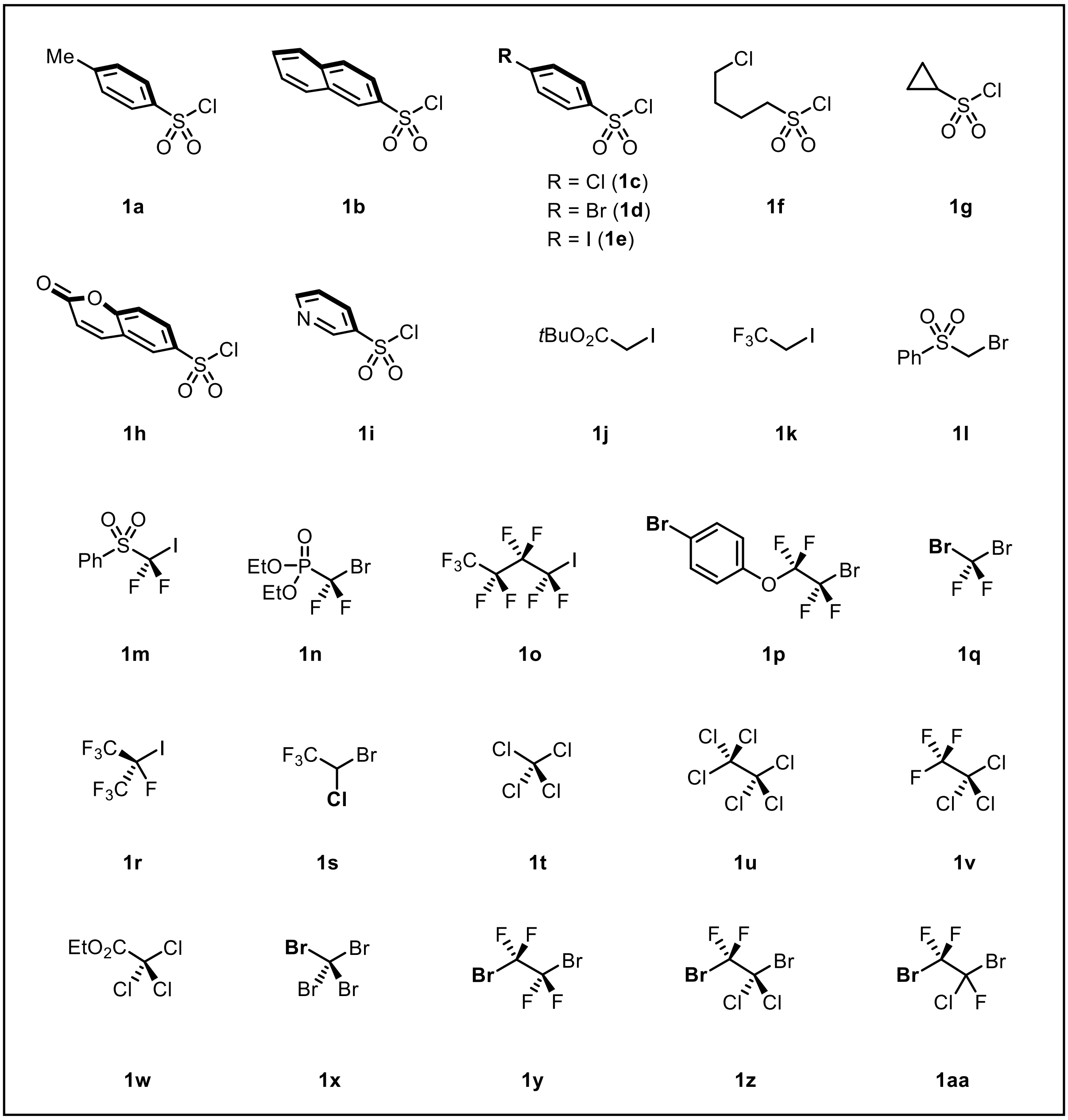

Figure S13. The list of commercially available radical sources.

5. Substrate Limitations

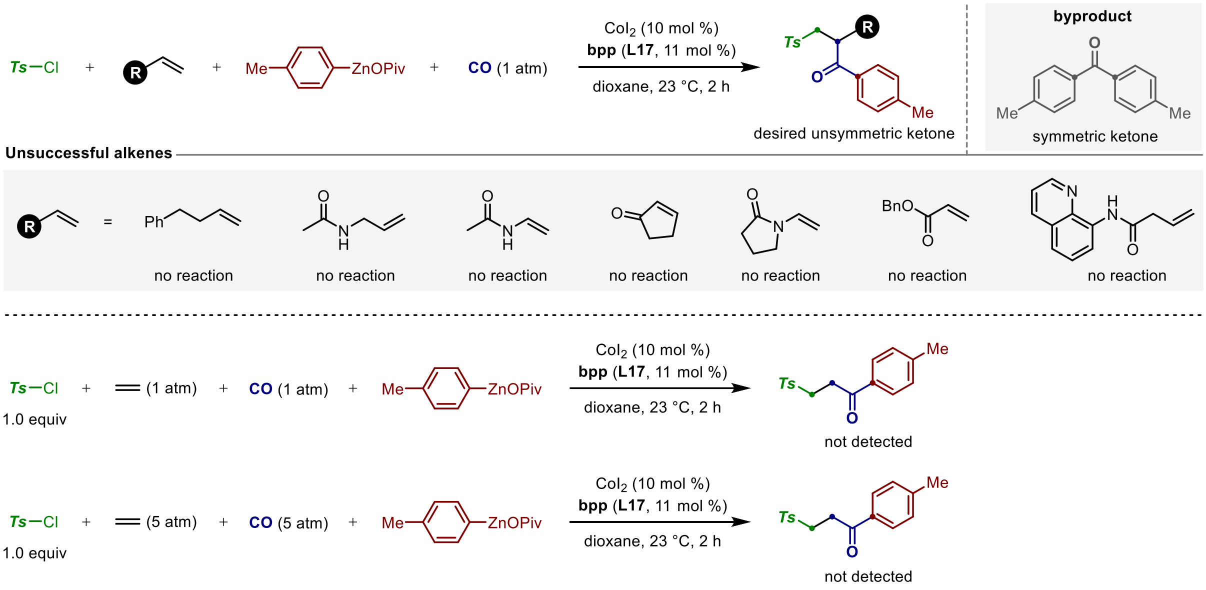

Figure S14. Substrate Limitations.

As shown in **Figure S14**, aliphatic alkene, as well as various alkenes bearing a heteroatom-containing directing group have been utilized as the unsaturated substrates under the standard reaction conditions. However, no positive results for the envisioned cobalt-catalyzed four-component carbonylative functionalization reaction were detected. The corresponding alkenes remained untouched in the reaction mixture, which can be also detected by GC-analysis, only forming the symmetric diarylmethyl ketone as the byproduct. Moreover, ethylene was also used as the unsaturated substrate for the envisioned four-component carbonylative transformation. Unfortunately, no desired product was observed under 1 atm or 5 atm of ethylene and CO gaseous mixture.

6. General Procedure for Cobalt-Catalyzed Alkene Sulfonylcarbonylation.

Typical procedure 3 (TP3) for cobalt-catalyzed alkene sulfonylcarbonylation under 1 atm of CO:

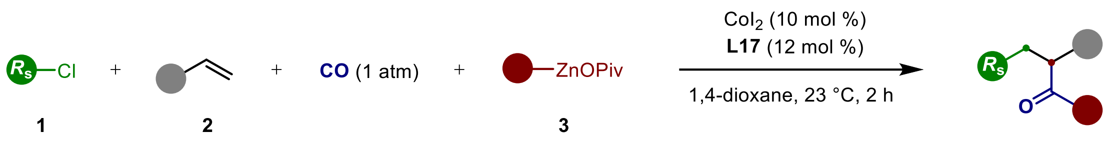

Figure S15. Cobalt-catalyzed alkene sulfonylcarbonylation.

An oven-dried tube was charged with CoI_2 (10 mol %), **L17** (12 mol %), sulfuryl chloride **1** (0.4 mmol, 2.0 equiv). Then the tube was evacuated and backfilled with CO (three times, 1 atm, balloon). Anhydrous 1,4-dioxane (1.0 mL) was added and stirred for 10 minutes vigorously. Alkene **2** (0.2 mmol, 1.0 equiv) was added. Then arylzinc pivalates **3** (0.4 mmol, 2.0 equiv) resolved in 1,4-dioxane (0.5 mL) was added dropwise over 5 minutes. The reaction mixture was stirred at 23 °C for 2 h. When the reaction was completed, the resulting residue was purified by column chromatography on silica gel (petroleum ether/EtOAc) to yield products.

Typical procedure 4 (TP4) for cobalt-catalyzed alkene sulfonylcarbonylation under 1 atm of CO:

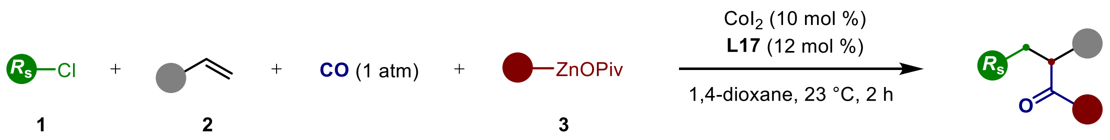

Figure S16. Cobalt-catalyzed alkene sulfonylcarbonylation.

An oven-dried tube was charged with CoI_2 (10 mol %), **L17** (12 mol %), sulfuryl chloride **1** (0.4 mmol, 2.0 equiv). Then the tube was evacuated and backfilled with CO (three times, 1 atm, balloon). Anhydrous 1,4-dioxane (1.0 mL) was added and stirred for 10 minutes vigorously. Alkene **2** (0.2 mmol, 1.0 equiv) was added. Then arylzinc pivalates **3** (0.6 mmol, 3.0 equiv) resolved in 1,4-dioxane (0.5 mL) was added dropwise over 5 minutes. The reaction mixture was stirred at 23 °C for 2 h. When the reaction was completed, the resulting residue was purified by column chromatography on silica gel (petroleum ether/EtOAc) to yield products.

Typical procedure 5 (TP5) for cobalt-catalyzed alkene sulfonylcarbonylation Using CO_2 as the C1 Source:

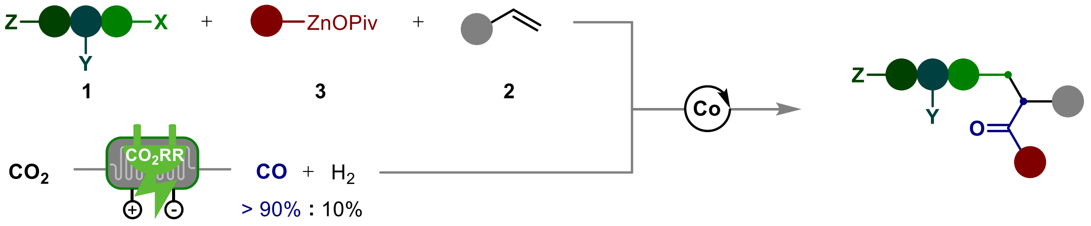

Figure S17. Cobalt-catalyzed alkene sulfonylcarbonylation with CO_2.

An oven-dried tube was charged with CoI_2 (10 mol %), **L17** (12 mol %). Then the tube was evacuated and backfilled with the gas mixture of CO:H_2 (> 90:10 ratio, three times, 1 atm, balloon)[4] (**Figure S17**). Anhydrous 1,4-dioxane (1.0 mL) was added and stirred for 10 minutes vigorously. Radical source **1** (0.4 mmol, 2.0 equiv) and alkene **2** (0.2 mmol, 1.0 equiv) were addded. Then arylzinc pivalates **3** (0.6 mmol, 3.0 equiv) resolved in 1,4-dioxane (0.5 mL) was added dropwise over 5 minutes. The reaction mixture was stirred at 23 °C for 2 h. When the reaction was completed, the resulting residue was purified by column chromatography on silica gel (petroleum ether/EtOAc) to yield products.

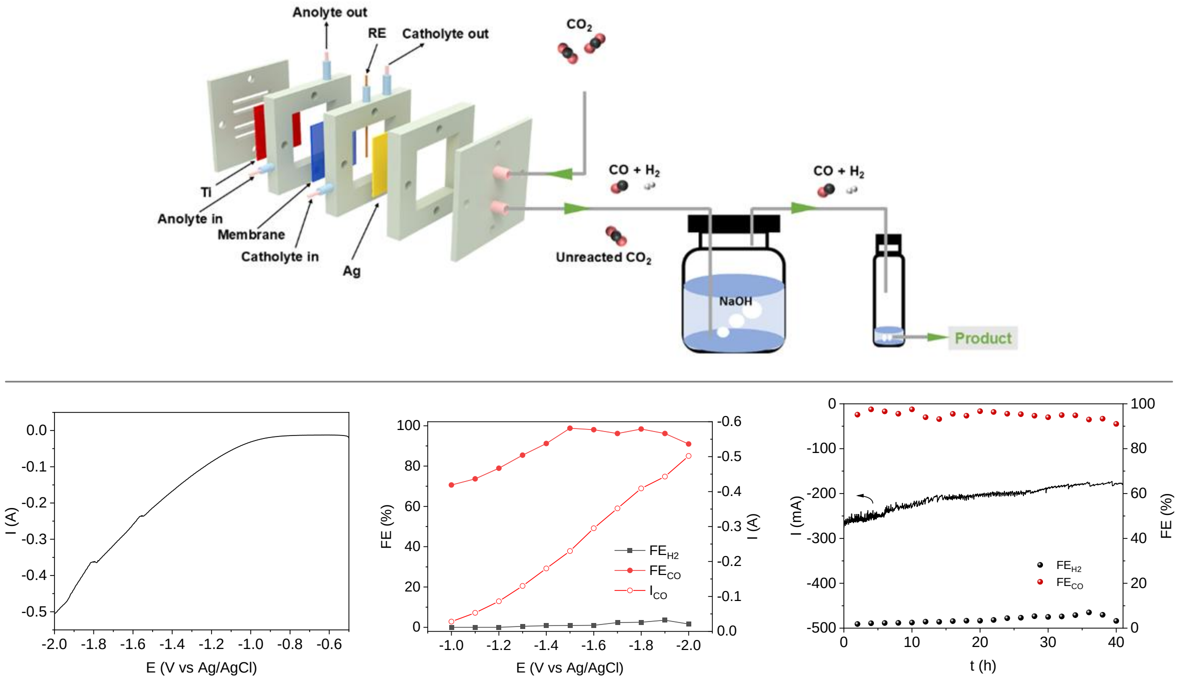

Figure S18. Schematic diagram of divergent radical alkene carbonylation under tandem electro-thermo-Catalysis using CO_2 as the C1 source.

We first loaded commercial Ag powder 60-150 nm (CAS number: 7440-22-4; purchased from Macklin) on the carbon black support. 4 mg of electrocatalyst and 2 mg carbon black (Vulcan

XC-72) were dispersed in 600 μL ethanol and 35 μL of 5% Nafion solution, and sonicated to form a uniform catalyst ink. The ink was dropcast onto a Teflon-treated carbon paper (1×1 cm^2, Toray YLS-30T) to reach the catalyst loading of 1 mg cm^{-2}. CO_2RR measurements were carried out in a customized flow cell separated by a AMI7001 anion exchange membrane. The 1 M KOH electrolyte and CO_2 were flowed at a rate of 10 mL min^{-1} and 20 mL min^{-1}, respectively. Catalyst-loaded carbon paper as the working electrode and a Ag/AgCl reference electrode were housed in the cathodic compartment; a Ti foil counter electrode was housed in the anodic compartment. Polarization curves were collected at the scan rate of 10 mV s^{-1}. Potentiostatic studies were carried out at a few selected working potentials. Reduction products during the potentiostatic studies were analyzed by an online gas chromatograph (Aligent 7890B) for H_2 and CO. As shown in Figure S17 bottom, a commercial available silver powder was tested in a cus-tomized flow cell and exhibited satisfactory CO2-to-CO conversion at an average rate of 3.73 mmol h^{-1} for over 40 h, providing a product gas mixture of CO/H_2 with >90/10 ratio.

7. Scale-Up Experiments

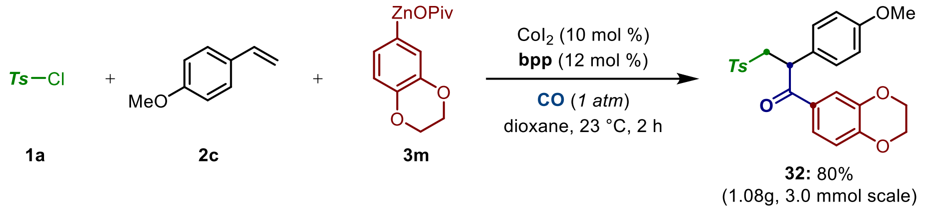

Figure S19. Scale-up experiment.

A 100-mL two-necked Schlenk tube was charged with CoI_2 (93.6 mg, 0.3 mmol, 10 mol %), **L17** (76.0 mg, 0.36 mmol, 12 mol %), tosyl chloride **1a** (1.14 g, 6.0 mmol, 2.0 equiv). Then the tube was evacuated and backfilled with CO (three times, 1 atm, balloon). Anhydrous 1,4-dioxane (15.0 mL) was added and stirred for 10 minutes vigorously. Alkene **2c** (402.5 mg, 3.0 mmol, 1.0 equiv) was added. Then arylzinc pivalates **3m** (6.0 mmol, 2.0 equiv) resolved in 1,4-dioxane (7.5 mL) were added dropwise over 20 minutes. The reaction mixture was stirred at 23 °C for 12 h. When the reaction was completed, the resulting residue was purified by column chromatography on silica gel (petroleum ether/EtOAc 4:1) to yield **32** (1.08 g, 80%) as a pale yellow solid. When 5 mol % of CoI_2 was used, the product **32** was yielded 61% (827 mg).

8. Transformations of Products

Typical procedure 6 (TP6) for accessing to halogenated cyclopropanes synthesis via monodehalogenation: [5]

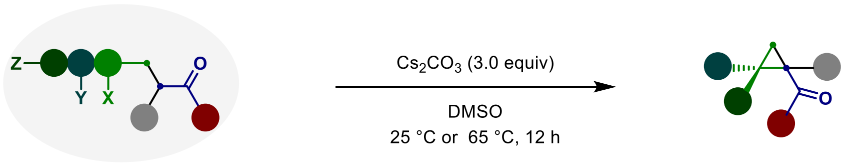

Figure S20. Synthesis of Halogenated cyclopropanes.

To a solution of carbonylation products (0.1 mmol) in DMSO (1.0 mL) was added **Cs_2CO_3** (0.3 mmol, 3.0 equiv). Then the reaction was stirred for 12 h at 25℃ or 65℃. The rection was quenched with H_2O and extracted with Et_2O. Then the combined organic phase was dried over anhydrous Na_2SO_4, filtered and concentrated under vacuum. The remaining residue was purified by column chromatography on silica gel (petroleum ether/EtOAc) to yield products **94**—**98**.

Typical procedure 7 (TP7) for accessing to [5,3]-bicyclic cores via selective monodehalogenation: [5]

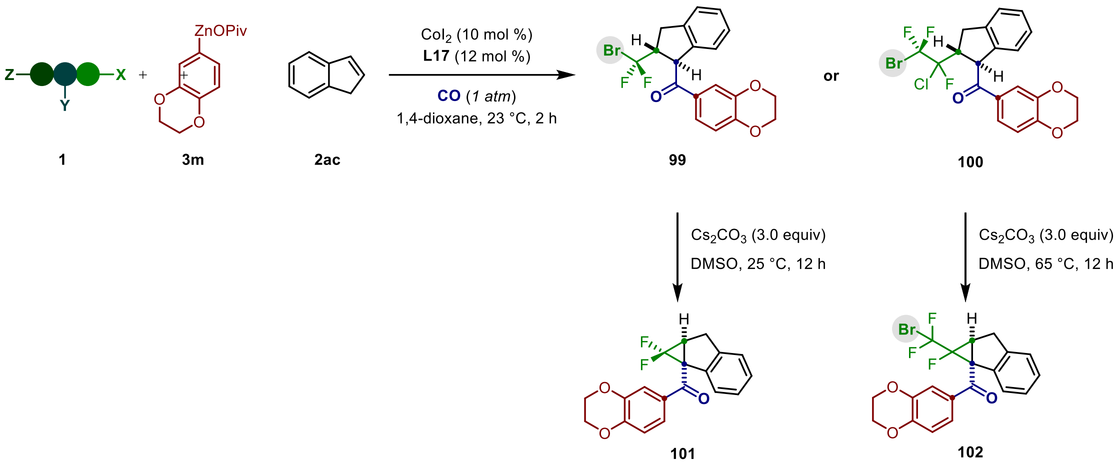

Figure S21. Synthesis of [5,3]-bicyclic cores.

An oven-dried tube was charged with CoI_2 (10 mol %), **L17** (12 mol %), sulfuryl chloride **1** (0.4 mmol, 2.0 equiv). Then the tube was evacuated and backfilled with CO (three times, 1 atm, balloon). Anhydrous 1,4-dioxane (1.0 mL) was added and stirred for 10 minutes vigorously. Alkene **2** (0.2 mmol, 1.0 equiv) was added. Then arylzinc pivalates **3** (0.4 mmol, 2.0 equiv) resolved in 1,4-dioxane (0.5 mL) was added dropwise over 5 minutes. The reaction mixture was stirred at 23 °C for 2 h. When the reaction was completed, the resulting residue was purified by column chromatography on silica gel (petroleum ether/EtOAc) to yield products **99** or **100**.

Then to a solution of carbonylation products (0.1 mmol) in DMSO (1.0 mL) was added **Cs_2CO_3** (0.3 mmol, 3.0 equiv). Then the reaction was stirred for 12 h at 25℃ or 65℃. The rection was quenched with H_2O and extracted with Et_2O. Then the combined organic phase was dried over anhydrous Na_2SO_4, filtered and concentrated under vacuum. The remaining residue was purified by column chromatography on silica gel (petroleum ether/EtOAc) to yield products **101** or **102**.

Typical procedure 8 (TP8) for reduction of carbonylation product:

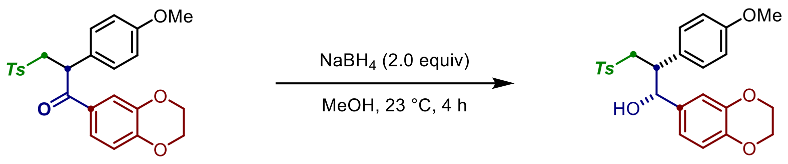

32 103

Figure S22. Reduction of carbonylation products.

To a solution of carbonylation products (0.5 mmol) in MeOH (3.0 mL) was added **$NaBH_4$** (1.0 mmol, 2.0 equiv). Then the reaction was stirred for 4 h at 25℃. The rection was quenched with H_2O and extracted with Et_2O. Then the combined organic phase was dried over anhydrous Na_2SO_4, filtered and concentrated under vacuum. The remaining residue was purified by column chromatography on silica gel (petroleum ether/EtOAc) to yield products **103**.

Typical procedure 9 (TP9) for desulfonylation of carbonylation products:[6]

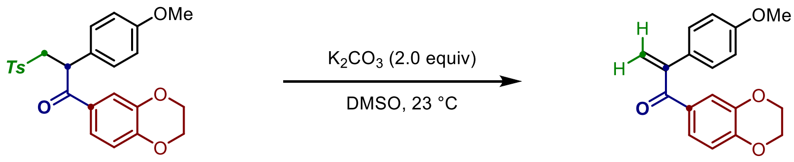

32 104

Figure S23. Desulfonylation of carbonylation products.

To a solution of carbonylation products (1.0 mmol) in DMSO (5.0 mL) was added **K_2CO_3** (2.0 mmol, 2.0 equiv). Then the reaction was stirred for 2.5 h at 25℃. The rection was quenched with H_2O and extracted with Et_2O. Then the combined organic phase was dried over anhydrous Na_2SO_4, filtered and concentrated under vacuum. The remaining residue was purified by column chromatography on silica gel (petroleum ether/EtOAc) to yield products **104**.

Typical procedure 10 (TP10) for silylation of desulfonylation product:[7]

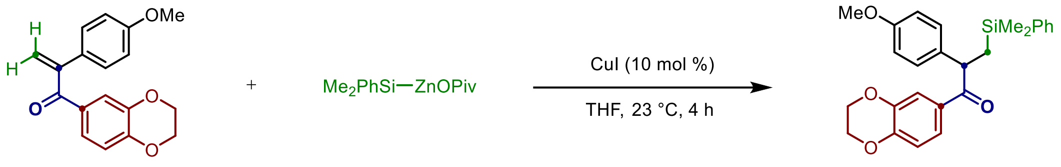

Figure S24. Silylation of desulfonylation product.

An oven-dried tube was charged with CuI (10 mol %), desulfonylation product **104** (0.1 mmol, 1.0 equiv). Then the tube was evacuated and backfilled with argon three times. Anhydrous THF (1.0 mL) was added and stirred for 2 minutes vigorously. Then **Me_2PhSi-ZnOPiv** (0.12 mmol, 1.2 equiv) resolved in THF (0.5 mL) was added dropwise in 1 minute. The reaction mixture was stirred at 23 °C for 4 h. When the reaction was completed, the resulting residue was purified by column chromatography on silica gel (petroleum ether/EtOAc) to yield products **105**.

Typical procedure 11 (TP11) for Friedel-Crafts reaction of desulfonylation product:[8]

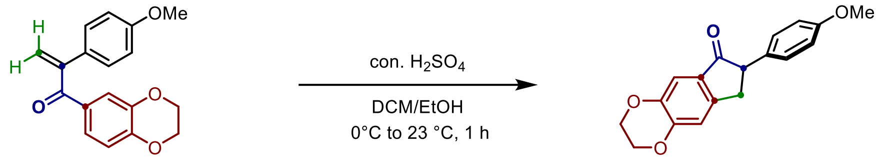

Figure S25. Friedel-Crafts reaction of desulfonylation product.

To a solution of desulfonylation product **104** (0.1 mmol) in EtOH/DCM 1:1 (1.0 mL) was added con.H_2SO_4 (1.0 mL) dropwise at 0 °C. Then the reaction was removed to room temperature and stirred for 1 h. The rection was quenched with H_2O (8.0 mL) and extracted with DCM. Then the combined organic phase was dried over anhydrous Na_2SO_4, filtered and concentrated under vacuum. The remaining residue was purified by column chromatography on silica gel (petroleum ether/EtOAc) to yield products **106**.

9. Characterization Data

9.1 Characterization Data of Alkenes

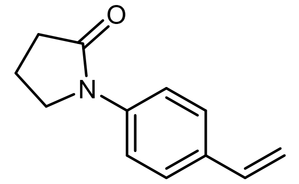

1-(4-Vinylphenyl)pyrrolidin-2-one (2l)

The general procedure **TP2** was followed using **thianthrenium salt** (1.0 equiv), **KOAc** (4.0 equiv) and **vinyl zinc pivalates** (3.0 equiv) for 6 h. Purification by column chromatography (petroleum ether/EtOAc 10:1) yielded **2l** (60%) as a solid. ^{1}H NMR (400 MHz, $CDCl_3$) δ = 7.63 – 7.52 (m, 2H), 7.44 – 7.36 (m, 2H), 6.69 (dd, *J* = 17.6, 10.9 Hz, 1H), 5.70 (dd, *J* = 17.6, 0.8 Hz, 1H), 5.21 (dd, *J* = 10.9, 0.7 Hz, 1H), 3.87 (t, *J* = 7.0 Hz, 2H), 2.62 (t, *J* = 8.1 Hz, 2H), 2.42 – 1.94 (m, 2H). ^{13}C NMR (100 MHz, $CDCl_3$) δ = 174.2, 136.1, 128.8, 126.6, 124.5, 119.9, 113.2, 48.8, 32.7, 18.0. HR-MS (ESI) m/z calcd for $C_{12}H_{13}NO$ [M+H$^+$] 188.1070, found 188.1074.

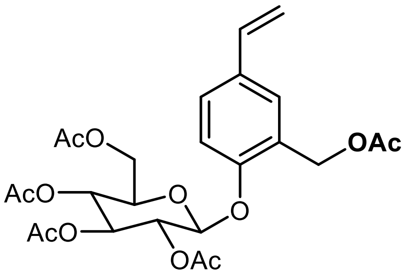

(2R,3R,4S,5R,6S)-2-(Acetoxymethyl)-6-[2-(acetoxymethyl)-4-vinylphenoxy]tetrahydro-2H-pyran-3,4,5-triyl triacetate (2v)

The general procedure **TP2** was followed using **thianthrenium salt** (1.0 equiv), **KOAc** (4.0 equiv) and **vinyl zinc pivalates** (3.0 equiv) for 6 h. Purification by column chromatography (petroleum ether/EtOAc 3:1) yielded **2v** (61%) as a solid. ^{1}H NMR (400 MHz, $CDCl_3$) δ = 7.38 (d, *J* = 1.7 Hz, 1H), 7.32 (dd, *J* = 8.4, 2.2 Hz, 1H), 7.04 (d, *J* = 8.5 Hz, 1H), 6.66 (dd, *J* = 17.6, 10.9 Hz, 1H), 5.66 (d, *J* = 17.6 Hz, 1H), 5.36 – 5.28 (m, 2H), 5.19 (dd, *J* = 17.7, 10.1 Hz, 2H), 5.14 – 5.01 (m, 3H), 4.28 (dd, *J* = 12.3, 5.2 Hz, 1H), 4.19 (dd, *J* = 12.3, 2.4 Hz, 1H), 3.90 – 3.81 (m, 1H), 2.12 – 2.02 (m, 15H). ^{13}C NMR (100 MHz, $CDCl_3$) δ = 170.7, 170.5, 170.2, 169.3, 169.2, 154.1, 135.6, 133.2, 127.4, 127.1, 126.3, 116.0, 113.5, 99.4, 72.6, 72.0, 71.0, 68.2, 61.8, 61.0, 20.9, 20.6, 20.6. HR-MS (ESI) m/z calcd for $C_{25}H_{30}O_{12}$ [M+H$^+$] 523.1810, found 523.1811.

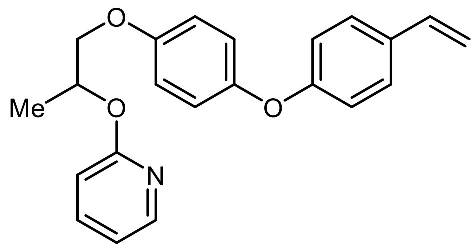

2-{{1-[4-(4-Vinylphenoxy)phenoxy]propan-2-yl}oxy}pyridine (2y)

The general procedure **TP2** was followed using **thianthrenium salt** (1.0 equiv), **KOAc** (4.0 equiv) and **vinyl zinc pivalates** (3.0 equiv) for 6 h. Purification by column chromatography (petroleum ether/EtOAc 20:1) yielded **2y** (66%) as a colourless oil. ^{1}H NMR (400 MHz, $CDCl_3$) δ = 8.15 (dd, J = 4.8, 1.5 Hz, 1H), 7.64 – 7.47 (m, 1H), 7.34 (d, J = 8.7 Hz, 2H), 7.00 – 6.82 (m, 7H), 6.74 (d, J = 8.4 Hz, 1H), 6.67 (dd, J = 17.6, 10.9 Hz, 1H), 5.70 – 5.52 (m, 2H), 5.16 (d, J = 10.9 Hz, 1H), 4.19 (dd, J = 9.9, 5.3 Hz, 1H), 4.07 (dd, J = 9.9, 4.8 Hz, 1H), 1.48 (d, J = 6.4 Hz, 3H). ^{13}C NMR (100 MHz, $CDCl_3$) δ = 158.2, 155.3, 150.1, 146.7, 138.7, 136.0, 132.1, 127.4, 120.7, 117.6, 116.7, 115.8, 112.4, 111.7, 71.0, 69.3, 17.0. HR-MS (ESI) m/z calcd for $C_{22}H_{21}NO_3$ [M+H$^+$] 348.1594, found 348.1596.

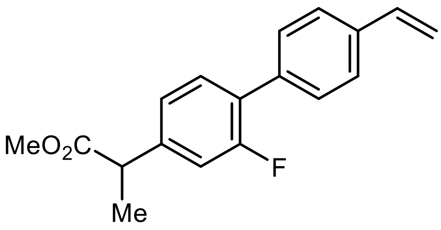

Methyl 2-(2-fluoro-4'-vinyl-[1,1'-biphenyl]-4-yl)propanoate (2ab)

The general procedure **TP2** was followed using **thianthrenium salt** (1.0 equiv), **KOAc** (4.0 equiv) and **vinyl zinc pivalates** (3.0 equiv) for 6 h. Purification by column chromatography (petroleum ether/EtOAc 20:1) yielded **2ab** (63%) as a colourless oil. ^{1}H NMR (400 MHz, $CDCl_3$) δ = 7.49 (q, J = 8.6 Hz, 4H), 7.40 (t, J = 7.9 Hz, 1H), 7.13 (t, J = 9.7 Hz, 2H), 6.75 (dd, J = 17.6, 10.9 Hz, 1H), 5.80 (d, J = 17.6 Hz, 1H), 5.29 (d, J = 10.8 Hz, 1H), 3.76 (q, J = 7.1 Hz, 1H), 3.70 (s, 3H), 1.53 (d, J = 7.2 Hz, 3H). ^{13}C NMR (100 MHz, $CDCl_3$) δ = 174.4, 159.7 (d, J_{C-F} = 248.6 Hz), 141.8 (d, J_{C-F} = 7.4 Hz), 136.9, 136.3, 130.6 (d, J_{C-F} = 3.8 Hz), 129.0 (d, J_{C-F} = 3.2 Hz), 126.3, 123.5 (d, J_{C-F} = 3.5 Hz), 115.3 (d, J_{C-F} = 23.8 Hz), 114.2, 52.2, 44.9, 18.4. ^{19}F NMR (376 MHz, $CDCl_3$) δ = -117.30. HR-MS (ESI) m/z calcd for $C_{18}H_{17}FO_2$ [M+H$^+$] 285.1285, found 285.1286.

9.2 Characterization Data of Products 4–47, 51–106, 108-109

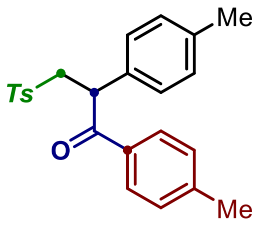

1,2-Di-p-tolyl-3-tosylpropan-1-one (4)

The general procedure **TP3** was followed using **1a** (0.4 mmol), **2a** (0.2 mmol) and **3a** (0.4 mmol) for 2 h. Purification by column chromatography (petroleum ether/EtOAc 4:1) yielded **4** (58.8 mg, 75%) as a yellow oil. ^{1}H NMR (400 MHz, $CDCl_3$) δ = 7.71 (d, *J* = 8.2 Hz, 2H), 7.60 (d, *J* = 8.3 Hz, 2H), 7.13 (d, *J* = 8.1 Hz, 2H), 7.08 (d, *J* = 8.1 Hz, 2H), 7.02 (d, *J* = 8.1 Hz, 2H), 6.94 (d, *J* = 8.0 Hz, 2H), 5.13 (dd, *J* = 8.6, 3.9 Hz, 1H), 4.29 (dd, *J* = 14.2, 8.6 Hz, 1H), 3.32 (dd, *J* = 14.2, 3.9 Hz, 1H), 2.29 (s, 3H), 2.25 (s, 3H), 2.14 (s, 3H). ^{13}C NMR (100 MHz, $CDCl_3$) δ = 195.5, 144.6, 144.2, 137.6, 136.5, 133.7, 132.9, 130.0, 129.7, 129.2, 129.0, 128.0, 127.9, 59.3, 47.0, 21.6, 21.6, 21.0. HR-MS (ESI) m/z calcd for $C_{24}H_{24}O_3S$ [M+Na$^+$] 415.1338, found 415.1340.

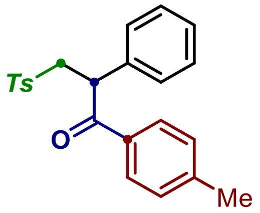

2-Phenyl-1-(p-tolyl)-3-tosylpropan-1-one (5)

The general procedure **TP3** was followed using **1a** (0.4 mmol), **2b** (0.2 mmol) and **3a** (0.4 mmol) for 2 h. Purification by column chromatography (petroleum ether/EtOAc 4:1) yielded **5** (51.5 mg, 68%) as a yellow oil. ^{1}H NMR (400 MHz, $CDCl_3$) δ = 7.83 – 7.77 (m, 2H), 7.69 (d, *J* = 8.3 Hz, 2H), 7.25 – 7.20 (m, 6H), 7.20 – 7.15 (m, 3H), 5.26 (dd, *J* = 8.7, 3.8 Hz, 1H), 4.40 (dd, *J* = 14.1, 8.7 Hz, 1H), 3.42 (dd, *J* = 14.2, 3.8 Hz, 1H), 2.38 (s, 3H), 2.34 (s, 3H). ^{13}C NMR (100 MHz, $CDCl_3$) δ = 195.4, 144.6, 144.3, 136.7, 136.4, 132.9, 129.8, 129.3, 129.3, 129.0, 128.1, 128.1, 127.8, 59.2, 47.3, 21.6, 21.6. HR-MS (ESI) m/z calcd for $C_{23}H_{22}O_3S$ [M+Na$^+$] 401.1182, found 401.1185.

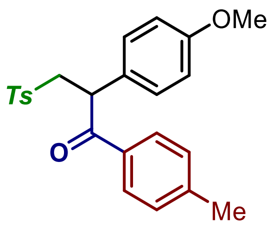

2-(4-Methoxyphenyl)-1-(p-tolyl)-3-tosylpropan-1-one (6)

The general procedure **TP3** was followed using **1a** (0.4 mmol), **2c** (0.2 mmol) and **3a** (0.4 mmol) for 2 h. Purification by column chromatography (petroleum ether/EtOAc 5:1) yielded **6** (59.6 mg, 73%) as a yellow oil. ^{1}H NMR (400 MHz, $CDCl_3$) δ = 7.79 (d, *J* = 8.2 Hz, 2H), 7.68 (d, *J* = 8.2 Hz, 2H), 7.22 (d, *J* = 8.1 Hz, 2H), 7.17 (d, *J* = 8.1 Hz, 2H), 7.13 (d, *J* = 8.7 Hz, 2H), 6.75 (d, *J* = 8.7 Hz, 2H), 5.21 (dd, *J* = 8.5, 4.1 Hz, 1H), 4.35 (dd, *J* = 14.2, 8.5 Hz, 1H), 3.70 (s, 3H), 3.41 (dd, *J* = 14.2, 4.1 Hz, 1H), 2.38 (s, 3H), 2.34 (s, 3H). ^{13}C NMR (100 MHz, $CDCl_3$) δ = 195.5, 159.1, 144.5, 144.2, 136.5, 132.9, 129.7, 129.3, 129.2, 129.0, 128.5, 128.0, 114.6, 59.3, 55.2, 46.5, 21.6, 21.6. HR-MS (ESI) m/z calcd for $C_{24}H_{24}O_4S$ [M+Na$^+$] 431.1288, found 431.1286.

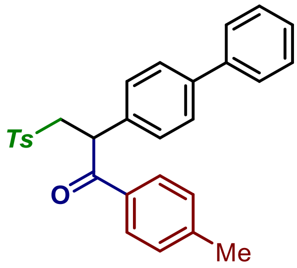

2-[(1,1'-Biphenyl)-4-yl]-1-(p-tolyl)-3-tosylpropan-1-one (7)

The general procedure **TP3** was followed using **1a** (0.4 mmol), **2d** (0.2 mmol) and **3a** (0.4 mmol) for 2 h. Purification by column chromatography (petroleum ether/EtOAc 5:1) yielded **7** (74.6 mg, 82%) as a colorless oil. ^{1}H NMR (400 MHz, $CDCl_3$) δ = 7.84 (d, *J* = 8.2 Hz, 2H), 7.68 (d, *J* = 8.3 Hz, 2H), 7.49 – 7.36 (m, 6H), 7.35 – 7.26 (m, 3H), 7.23 – 7.15 (m, 4H), 5.30 (dd, *J* = 8.3, 4.2 Hz, 1H), 4.39 (dd, *J* = 14.2, 8.4 Hz, 1H), 3.50 (dd, *J* = 14.2, 4.2 Hz, 1H), 2.36 (s, 3H), 2.36 (s, 3H). ^{13}C NMR (100 MHz, $CDCl_3$) δ = 195.4, 144.6, 144.4, 140.8, 140.2, 136.5, 135.5, 132.9, 129.7, 129.3, 129.0, 128.8, 128.5, 128.0, 127.9, 127.5, 126.9, 59., 47.1, 21.6, 21.6. HR-MS (ESI) m/z calcd for $C_{29}H_{26}O_3S$ [M+Na$^+$] 477.1495, found 477.1498.

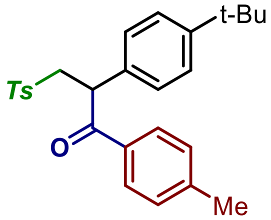

2-[4-(*tert*-Butyl)phenyl]-1-(p-tolyl)-3-tosylpropan-1-one (8)

The general procedure **TP3** was followed using **1a** (0.4 mmol), **2e** (0.2 mmol) and **3a** (0.4 mmol) for 2 h. Purification by column chromatography (petroleum ether/EtOAc 4:1) yielded **8** (67.8 mg, 78%) as a yellow oil. ^{1}H NMR (400 MHz, $CDCl_3$) δ = 7.82 (d, *J* = 8.2 Hz, 2H), 7.66 (d, *J* = 8.3 Hz, 2H), 7.24 – 7.16 (m, 6H), 7.13 (d, *J* = 8.4 Hz, 2H), 5.23 (dd, *J* = 8.7, 3.8 Hz, 1H), 4.39 (dd, *J* = 14.2, 8.7 Hz, 1H), 3.44 (dd, *J* = 14.2, 3.8 Hz, 1H), 2.37 (s, 3H), 2.35 (s, 3H), 1.22 (s, 9H). ^{13}C NMR (100 MHz, $CDCl_3$) δ = 195.5, 150.7, 144.4, 144.3, 136.4, 133.4, 133.0, 129.7, 129.2, 129.0, 128.0, 127.7, 126.2, 59.3, 46.9, 34.4, 31.2, 21.6, 21.6. HR-MS (ESI) m/z calcd for $C_{27}H_{30}O_3S$ [M+Na$^+$] 457.1808, found 457.1810.

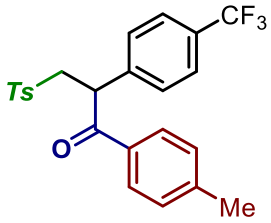

1-(*p*-Tolyl)-3-tosyl-2-[4-(trifluoromethyl)phenyl]propan-1-one (9)

The general procedure **TP3** was followed using **1a** (0.4 mmol), **2f** (0.2 mmol) and **3a** (0.4 mmol) for 2 h. Purification by column chromatography (petroleum ether/EtOAc 5:1) yielded **9** (59.8 mg, 67%) as a yellow oil. ^{1}H NMR (400 MHz, $CDCl_3$) δ = 7.80 (d, *J* = 8.2 Hz, 2H), 7.64 (d, *J* = 8.3 Hz, 2H), 7.47 (d, *J* = 8.2 Hz, 2H), 7.35 (d, *J* = 8.1 Hz, 2H), 7.21 (d, *J* = 8.0 Hz, 4H), 5.34 (dd, *J* = 7.4, 5.2 Hz, 1H), 4.28 (dd, *J* = 14.3, 7.6 Hz, 1H), 3.50 (dd, *J* = 14.3, 5.2 Hz, 1H), 2.39 (s, 3H), 2.37 (s, 3H). ^{13}C NMR (100 MHz, $CDCl_3$) δ = 194.9, 144.9, 144.8, 140.4, 136.2, 132.5, 130.0 (q, $J_{C\text{-}F}$ = 32.0 Hz), 129.8, 129.5, 129.0, 128.7, 127.9, 126.1 (q, $J_{C\text{-}F}$ = 4.0 Hz), 124.9 (q, $J_{C\text{-}F}$ = 272.0 Hz), 58.9, 47.1, 21.6, 21.5. ^{19}F NMR (376 MHz, $CDCl_3$) δ = -62.79. HR-MS (ESI) m/z calcd for $C_{24}H_{21}F_3O_3S$ [M+Na$^+$] 469.1056, found 469.1055.

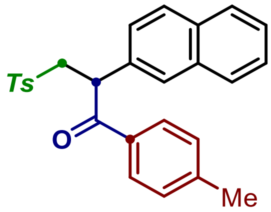

2-(Naphthalen-2-yl)-1-(p-tolyl)-3-tosylpropan-1-one (10)

The general procedure **TP3** was followed using **1a** (0.4 mmol), **2g** (0.2 mmol) and **3a** (0.4 mmol) for 2 h. Purification by column chromatography (petroleum ether/EtOAc 5:1) yielded **10** (42.9 mg, 50%) as a colorless oil. ^{1}H NMR (400 MHz, $CDCl_3$) δ = 7.84 (d, *J* = 8.2 Hz, 2H), 7.77 – 7.60 (m, 6H), 7.47 – 7.39 (m, 2H), 7.34 (dd, *J* = 8.5, 1.6 Hz, 1H), 7.15 (t, *J* = 8.6 Hz, 4H), 5.41 (dd, *J* = 8.1, 4.5 Hz, 1H), 4.43 (dd, *J* = 14.3, 8.1 Hz, 1H), 3.55 (dd, *J* = 14.3, 4.4 Hz, 1H), 2.33 (s, 3H), 2.31 (s, H). ^{13}C NMR (100 MHz, $CDCl_3$) δ = 195.4, 144.5, 144.4 136.4, 133.9, 133.4, 132.9, 132.6, 129.6, 129.3, 129.2, 129.0, 128.0, 127.8, 127.5, 127.5, 126.4, 126.3, 125.5, 59.1, 47.6, 29.7, 21.6. HR-MS (ESI) m/z calcd for $C_{27}H_{24}O_3S$ [M+Na$^+$] 451.1338, found 451.1336.

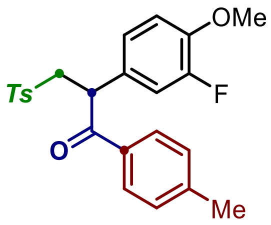

2-(3-Fluoro-4-methoxyphenyl)-1-(p-tolyl)-3-tosylpropan-1-one (11)

The general procedure **TP4** was followed using **1a** (0.4 mmol), **2h** (0.2 mmol) and **3a** (0.6 mmol) for 2 h. Purification by column chromatography (petroleum ether/EtOAc 5:1) yielded **11** (55.4 mg, 65%) as a colorless oil. ^{1}H NMR (400 MHz, $CDCl_3$) δ = 7.79 (d, *J* = 8.2 Hz, 2H), 7.67 (d, *J* = 8.2 Hz, 2H), 7.21 (dd, *J* = 15.6, 8.1 Hz, 4H), 6.99 – 6.89 (m, 2H), 6.80 (t, *J* = 8.5 Hz, 1H), 5.18 (dd, *J* = 8.0, 4.5 Hz, 1H), 4.29 (dd, *J* = 14.2, 8.1 Hz, 1H), 3.79 (s, 3H), 3.43 (dd, *J* = 14.2, 4.5 Hz, 1H), 2.39 (s, 3H), 2.36 (s, 3H). ^{13}C NMR (100 MHz, $CDCl_3$) δ = 195.2, 152.3 (d, J_{C-F} = 247.8 Hz), 147.3 (d, J_{C-F} = 10.7 Hz), 144.6 (d, J_{C-F} = 16.1 Hz), 136.4, 132.7, 129.7, 129.3, 128.9, 128.0, 124.8, 124.1 (d, J_{C-F} = 3.5 Hz), 115.8 (d, J_{C-F} = 19.2 Hz), 113.9 (d, J_{C-F} = 2.0 Hz), 59.1, 56.1, 46.3, 21.6, 21.5. ^{19}F NMR (376 MHz, $CDCl_3$) δ = -133.22. HR-MS (ESI) m/z calcd for $C_{24}H_{23}FO_4S$ [M+Na$^+$] 449.1193, found 449.1196.

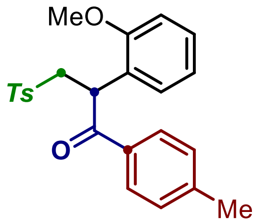

2-(2-Methoxyphenyl)-1-(p-tolyl)-3-tosylpropan-1-one (12)

The general procedure **TP3** was followed using **1a** (0.4 mmol), **2i** (0.2 mmol) and **3a** (0.4 mmol) for 2 h. Purification by column chromatography (petroleum ether/EtOAc 5:1) yielded **12** (50.6

mg, 62%) as a colorless oil. ^{1}H NMR (400 MHz, $CDCl_3$) δ = 7.80 (d, *J* = 8.3 Hz, 2H), 7.71 (d, *J* = 8.3 Hz, 2H), 7.24 (t, *J* = 6.0 Hz, 2H), 7.18 – 7.10 (m, 3H), 6.99 (dd, *J* = 7.7, 1.6 Hz, 1H), 6.83 – 6.72 (m, 2H), 5.62 (dd, *J* = 8.6, 3.7 Hz, 1H), 4.36 (dd, *J* = 14.2, 8.6 Hz, 1H), 3.82 (s, 3H), 3.34 (dd, *J* = 14.2, 3.7 Hz, 1H), 2.40 (s, 3H), 2.33 (s, 3H). ^{13}C NMR (100 MHz, $CDCl_3$) δ = 196.0, 155.7, 144.3, 144.0, 136.7, 133.1, 129.6, 129.1, 128.8, 128.4, 128.1, 125.1, 121.1, 111.1, 58.1, 55.5, 40.3, 21.6, 21.6. HR-MS (ESI) m/z calcd for $C_{24}H_{24}O_4S$ [M+Na$^+$] 431.1288, found 431.1285.

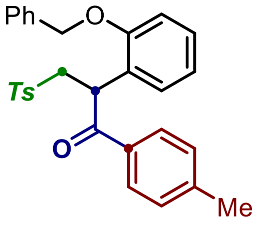

2-[2-(Benzyloxy)phenyl]-1-(p-tolyl)-3-tosylpropan-1-one (13)

The general procedure **TP3** was followed using **1a** (0.4 mmol), **2j** (0.2 mmol) and **3a** (0.4 mmol) for 2 h. Purification by column chromatography (petroleum ether/EtOAc 5:1) yielded **13** (82.4 mg, 85%) as a white powder. ^{1}H NMR (400 MHz, $CDCl_3$) δ = 7.80 (d, *J* = 8.3 Hz, 2H), 7.67 (d, *J* = 8.3 Hz, 2H), 7.53 – 7.47 (m, 2H), 7.47 – 7.42 (m, 2H), 7.40 – 7.35 (m, 1H), 7.14 (d, *J* = 8.1 Hz, 2H), 7.12 – 7.03 (m, 4H), 6.85 (d, *J* = 7.7 Hz, 1H), 6.81 – 6.75 (m, 1H), 5.73 (dd, *J* = 8.3, 4.2 Hz, 1H), 5.19 – 5.05 (m, 2H), 4.36 (dd, *J* = 14.3, 8.3 Hz, 1H), 3.42 (dd, *J* = 14.3, 4.2 Hz, 1H), 2.33 (s, 3H), 2.30 (s, 3H). ^{13}C NMR (100 MHz, $CDCl_3$) δ = 196.1, 154.8, 144.3, 144.0, 136.6, 133.0, 129.6, 129.1, 128.9, 128.7, 128.1, 128.1, 127.4, 125.3, 121.4, 112.5, 70.3, 58.4, 40.1, 21.6, 21.6. HR-MS (ESI) m/z calcd for $C_{30}H_{28}O_4S$ [M+Na$^+$] 507.1601, found 507.1603.

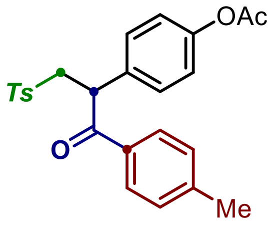

4-[1-Oxo-1-(p-tolyl)-3-tosylpropan-2-yl]phenyl acetate (14)

The general procedure **TP3** was followed using **1a** (0.4 mmol), **2k** (0.2 mmol) and **3a** (0.4 mmol) for 2 h. Purification by column chromatography (petroleum ether/EtOAc 3:1) yielded **14** (54.1 mg, 62%) as a white powder. ^{1}H NMR (400 MHz, $CDCl_3$) δ = 7.80 (d, *J* = 8.2 Hz, 2H), 7.68 (d, *J* = 8.3 Hz, 2H), 7.26 – 7.21 (m, 4H), 7.18 (d, *J* = 8.1 Hz, 2H), 7.00 – 6.94 (m, 2H), 5.27 (dd, *J* = 8.8, 3.7 Hz, 1H), 4.38 (dd, *J* = 14.2, 8.7 Hz, 1H), 3.41 (dd, *J* = 14.2, 3.8 Hz, 1H),

2.38 (s, 3H), 2.35 (s, 3H), 2.23 (s, 3H). ^{13}C NMR (100 MHz, $CDCl_3$) δ = 195.3, 169.1, 150.2, 144.7, 144.5, 136., 134.1, 132.8, 129.8, 129.3, 129.1, 129.0, 128.0, 122.4, 59.2, 46.6, 21.6, 21.6, 21.0. HR-MS (ESI) m/z calcd for $C_{25}H_{24}O_5S$ [M+Na$^+$] 459.1237, found 457.1239.

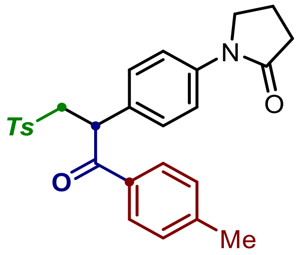

1-{4-[1-Oxo-1-(p-tolyl)-3-tosylpropan-2-yl]phenyl}pyrrolidin-2-one (15)

The general procedure **TP4** was followed using **1a** (0.4 mmol), **2l** (0.2 mmol) and **3a** (0.6 mmol) for 2 h. Purification by column chromatography (petroleum ether/EtOAc 2:1) yielded **15** (66.4 mg, 72%) as a white powder. ^{1}H NMR (400 MHz, $CDCl_3$) δ = 7.78 (d, J = 8.2 Hz, 2H), 7.69 (d, J = 8.2 Hz, 2H), 7.50 (d, J = 8.7 Hz, 2H), 7.23 (t, J = 8.0 Hz, 4H), 7.17 (d, J = 8.0 Hz, 2H), 5.23 (dd, J = 8.4, 4.0 Hz, 1H), 4.36 (dd, J = 14.1, 8.5 Hz, 1H), 3.77 (t, J = 7.1 Hz, 2H), 3.39 (dd, J = 14.1, 4.0 Hz, 1H), 2.57 (t, J = 8.1 Hz, 2H), 2.40 (s, 3H), 2.36 (s, 3H), 2.17 – 2.07 (m, 2H). ^{13}C NMR (100 MHz, $CDCl_3$) δ = 195.2, 174.2, 144.6, 144.3, 139.0, 136.4, 132.9, 132.5, 129.8, 129.3, 129.0, 128.5, 128.0, 120.4, 59.1, 48.5, 46.8, 32.6, 21.6, 21.6, 17.9. HR-MS (ESI) m/z calcd for $C_{27}H_{27}NO_4S$ [M+Na$^+$] 484.1553, found 484.1556.

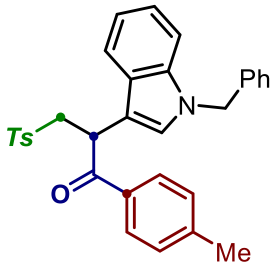

2-(1-Benzyl-1H-indol-3-yl)-1-(p-tolyl)-3-tosylpropan-1-one (16)

The general procedure **TP3** was followed using **1a** (0.4 mmol), **2m** (0.2 mmol) and **3a** (0.4 mmol) for 2 h. Co(acac)$_2$ (10 mol %) as the catalyst instead of CoI_2 and THF as solvent instead of dioxane. Purification by column chromatography (petroleum ether/EtOAc 6:1) yielded **16** (66.0 mg, 65%) as a yellow oil. ^{1}H-NMR (400 MHz, $CDCl_3$): δ = 7.85 – 7.77 (m, 2H), 7.73 – 7.61 (m, 3H), 7.25 – 7.11 (m, 10H), 7.00 – 6.91 (m, 2H), 6.84 (s, 1H), 5.52 (dd, J = 8.9, 3.2 Hz, 1H), 5.22 – 5.03 (m, 2H), 4.45 (dd, J = 14.2, 8.9 Hz, 1H), 3.53 (dd, J = 14.3, 3.3 Hz, 1H), 2.38 (s, 3H), 2.35 (s, 3H). ^{13}C-NMR (100 MHz, $CDCl_3$): δ = 195.6, 144.6, 144.1, 137.0, 137.0, 136.6, 133.3, 129.8, 129.3, 129.0, 128.9, 128.2, 127.8, 127.4, 126.8, 126.3, 122.6, 120.2, 118.9, 110.7,

110.2, 58.6, 50.2, 39.1, 21.8, 21.7. HR-MS (ESI) m/z calcd for $C_{32}H_{29}NO_3S$ [M+H$^+$] 508.1941, found 508.1943.

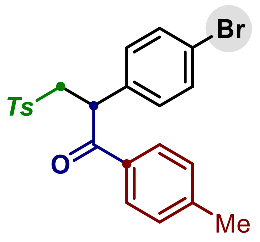

2-(4-Bromophenyl)-1-(p-tolyl)-3-tosylpropan-1-one (17)

The general procedure **TP3** was followed using **1a** (0.4 mmol), **2n** (0.2 mmol) and **3a** (0.4 mmol) for 2 h. Purification by column chromatography (petroleum ether/EtOAc 5:1) yielded **17** (53.0 mg, 58%) as a yellow oil. ^{1}H NMR (400 MHz, $CDCl_3$) δ = 7.78 (d, *J* = 8.2 Hz, 2H), 7.65 (d, *J* = 8.3 Hz, 2H), 7.36 – 7.31 (m, 2H), 7.21 (dd, *J* = 14.7, 8.0 Hz, 4H), 7.14 – 7.07 (m, 2H), 5.23 (dd, *J* = 7.8, 4.8 Hz, 1H), 4.28 (dd, *J* = 14.2, 7.9 Hz, 1H), 3.45 (dd, *J* = 14.2, 4.8 Hz, 1H), 2.40 (s, 3H), 2.36 (s, 3H). ^{13}C NMR (100 MHz, $CDCl_3$) δ = 195.1, 144.8, 144.7, 136.3, 135.6, 132.6, 132.3, 129.9, 129.8, 129.4, 129.0, 128.0, 122.1, 58.9, 46.8, 21.6, 21.6. HR-MS (ESI) m/z calcd for $C_{23}H_{21}BrO_3S$ [M+Na$^+$] 479.0287, found 479.0284.

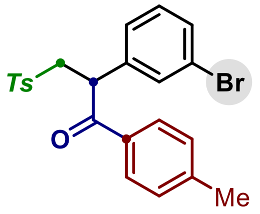

2-(3-Bromophenyl)-1-(p-tolyl)-3-tosylpropan-1-one (18)

The general procedure **TP3** was followed using **1a** (0.4 mmol), **2o** (0.2 mmol) and **3a** (0.4 mmol) for 2 h. Purification by column chromatography (petroleum ether/EtOAc 5:1) yielded **18** (55.8 mg, 61%) as a yellow oil. ^{1}H NMR (400 MHz, $CDCl_3$) δ = 7.80 (d, *J* = 8.2 Hz, 2H), 7.66 (d, *J* = 8.3 Hz, 2H), 7.33 (t, *J* = 1.7 Hz, 1H), 7.31 – 7.27 (m, 1H), 7.25 – 7.17 (m, 5H), 7.09 (t, *J* = 7.8 Hz, 1H), 5.22 (dd, *J* = 8.1, 4.5 Hz, 1H), 4.31 (dd, *J* = 14.2, 8.1 Hz, 1H), 3.45 (dd, *J* = 14.2, 4.5 Hz, 1H), 2.39 (s, 3H), 2.36 (s, 3H). ^{13}C NMR (100 MHz, $CDCl_3$) δ = 194.9, 144.8, 144.7, 138.7, 136.2, 132.6, 131.1, 131.0, 130.7, 129.8, 129.4, 129.0, 128.0, 126.9, 123.2, 59.0, 46.9, 21.7, 21.6. HR-MS (ESI) m/z calcd for $C_{23}H_{21}BrO_3S$ [M+Na$^+$] 479.0287, found 479.0285.

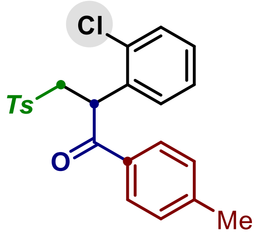

2-(2-Chlorophenyl)-1-(p-tolyl)-3-tosylpropan-1-one (19)

The general procedure **TP4** was followed using **1a** (0.4 mmol), **2p** (0.2 mmol) and **3a** (0.6 mmol) for 2 h. Purification by column chromatography (petroleum ether/EtOAc 4:1) yielded **19** (34.7 mg, 42%) as a yellow oil. ^{1}H NMR (400 MHz, $CDCl_3$) δ = 7.80 (d, *J* = 8.2 Hz, 2H), 7.75 (d, *J* = 8.2 Hz, 2H), 7.36 (d, *J* = 7.8 Hz, 1H), 7.27 (d, *J* = 8.2 Hz, 2H), 7.19 (d, *J* = 8.1 Hz, 2H), 7.15 – 7.05 (m, 3H), 5.67 (dd, *J* = 9.3, 3.1 Hz, 1H), 4.37 (dd, *J* = 14.2, 9.3 Hz, 1H), 3.31 (dd, *J* = 14.2, 3.1 Hz, 1H), 2.41 (s, 3H), 2.36 (s, 3H). ^{13}C NMR (100 MHz, $CDCl_3$) δ = 195.1, 144.7, 144.6, 136.3, 134.2, 133.2, 132.8, 130.4, 129.8, 129.4, 129.2, 128.9, 128.8, 128.2, 127.6, 58.0, 43.7, 21.6, 21.6. HR-MS (ESI) m/z calcd for $C_{23}H_{21}ClO_3S$ [M+Na$^+$] 435.0792, found 435.0795.

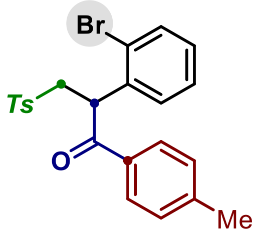

2-(2-Bromophenyl)-1-(p-tolyl)-3-tosylpropan-1-one (20)

The general procedure **TP4** was followed using **1a** (0.4 mmol), **2q** (0.2 mmol) and **3a** (0.6 mmol) for 2 h. Purification by column chromatography (petroleum ether/EtOAc 5:1) yielded **20** (43.9 mg, 48%) as a yellow oil. ^{1}H NMR (400 MHz, $CDCl_3$) δ = 7.81 (d, *J* = 8.2 Hz, 2H), 7.76 (d, *J* = 8.2 Hz, 2H), 7.58 – 7.53 (m, 1H), 7.28 (d, *J* = 8.1 Hz, 2H), 7.20 (d, *J* = 8.1 Hz, 2H), 7.14 – 7.05 (m, 3H), 5.64 (dd, *J* = 9.8, 2.6 Hz, 1H), 4.36 (dd, *J* = 14.1, 9.9 Hz, 1H), 3.28 (dd, *J* = 14.1, 2.6 Hz, 1H), 2.41 (s, 3H), 2.36 (s, 3H). ^{13}C NMR (100 MHz, $CDCl_3$) δ = 195.0, 144.7, 144.6, 136.4, 135.8, 133.8, 132.9, 129.8, 129.5, 129.4, 129.0, 128.9, 128.3, 128.2, 124.0, 58.1, 46.5, 21.6, 21.6. HR-MS (ESI) m/z calcd for $C_{23}H_{21}BrO_3S$ [M+Na$^+$] 479.0287, found 479.0285.

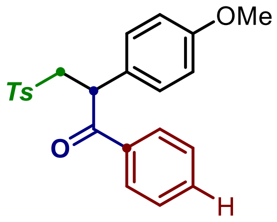

2-(4-Methoxyphenyl)-1-phenyl-3-tosylpropan-1-one (21)

The general procedure **TP3** was followed using **1a** (0.4 mmol), **2c** (0.2 mmol) and **3b** (0.4 mmol) for 2 h. Purification by column chromatography (petroleum ether/EtOAc 6:1) yielded **21** (52.1 mg, 66%) as a colourless oil. ^{1}H-NMR (400 MHz, $CDCl_3$): δ = 7.92 – 7.85 (m, 2H), 7.73 – 7.65 (m, 2H), 7.55 – 7.45 (m, 1H), 7.43 – 7.34 (m, 2H), 7.25 – 7.19 (m, 2H), 7.17 – 7.10 (m, 2H), 6.79 – 6.72 (m, 2H), 5.23 (dd, J = 8.7, 3.9 Hz, 1H), 4.36 (dd, J = 14.1, 8.6 Hz, 1H), 3.72 (s, 3H), 3.41 (dd, J = 14.1, 3.9 Hz, 1H), 2.39 (s, 3H). ^{13}C-NMR (100 MHz, $CDCl_3$): δ = 196.1, 159.3, 144.7, 136.6, 135.6, 133.5, 129.9, 129.4, 129.0, 128.7, 128.4, 128.2, 114.9, 59.5, 55.3, 46.8, 21.7. HR-MS (ESI) m/z calcd for $C_{23}H_{22}O_4S$ [M+H$^+$] 395.1312, found 395.1310.

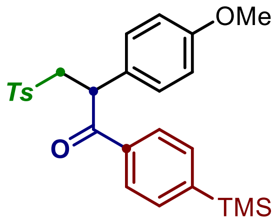

2-(4-Methoxyphenyl)-3-tosyl-1-[4-(trimethylsilyl)phenyl]propan-1-one (22)

The general procedure **TP3** was followed using **1a** (0.4 mmol), **2c** (0.2 mmol) and **3c** (0.4 mmol) for 2 h. Purification by column chromatography (petroleum ether/EtOAc 6:1) yielded **22** (79.2 mg, 84%) as a colourless oil. ^{1}H-NMR (400 MHz, $CDCl_3$): δ = 7.86 (d, J = 8.1 Hz, 2H), 7.69 (d, J = 8.0 Hz, 2H), 7.54 (d, J = 7.9 Hz, 2H), 7.22 (d, J = 8.0 Hz, 2H), 7.17 – 7.11 (m, 2H), 6.80 – 6.72 (m, 2H), 5.23 (dd, J = 8.6, 4.0 Hz, 1H), 4.36 (dd, J = 14.1, 8.6 Hz, 1H), 3.70 (s, 3H), 3.42 (dd, J = 14.2, 4.0 Hz, 1H), 2.38 (s, 3H), 0.25 (s, 9H). ^{13}C-NMR (100 MHz, $CDCl_3$): δ = 196.2, 159.3, 147.7, 144.7, 136.6, 135.6, 133.5, 129.8, 129.4, 128.4, 128.1, 127.8, 114.8, 59.4, 55.3, 46.8, 21.7, -1.3. HR-MS (ESI) m/z calcd for $C_{26}H_{30}O_4SSi$ [M+H$^+$] 467.1707, found 467.1707.

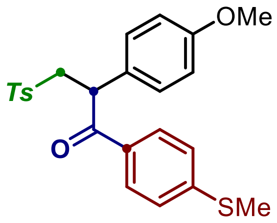

2-(4-Methoxyphenyl)-1-[4-(methylthio)phenyl]-3-tosylpropan-1-one (23)

The general procedure **TP3** was followed using **1a** (0.4 mmol), **2c** (0.2 mmol) and **3d** (0.4 mmol) for 2 h. Purification by column chromatography (petroleum ether/EtOAc 5:1) yielded **23** (61.3 mg, 69%) as a colourless oil. ^{1}H-NMR (400 MHz, $CDCl_3$): δ = 7.82 – 7.75 (m, 2H), 7.71 – 7.64 (m, 2H), 7.25 – 7.19 (m, 2H), 7.19 – 7.09 (m, 4H), 6.78 – 6.70 (m, 2H), 5.17 (dd, J = 8.5, 4.0 Hz, 1H), 4.34 (dd, J = 14.1, 8.5 Hz, 1H), 3.70 (s, 3H), 3.40 (dd, J = 14.1, 4.1 Hz, 1H), 2.46 (s, 3H), 2.38 (s, 3H). ^{13}C-NMR (100 MHz, $CDCl_3$): δ = 195.0, 159.3, 146.6, 144.7, 136.6, 131.7, 129.8, 129.3, 129.3, 128.6, 128.1, 124.9, 114.8, 59.3, 55.3, 46.6, 21.7, 14.7. HR-MS (ESI) m/z calcd for $C_{24}H_{24}O_4S_2$ [M+H$^+$] 441.1189, found 441.1188.

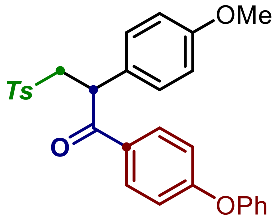

2-(4-Methoxyphenyl)-1-(4-phenoxyphenyl)-3-tosylpropan-1-one (24)

The general procedure **TP3** was followed using **1a** (0.4 mmol), **2c** (0.2 mmol) and **3e** (0.4 mmol) for 2 h. Purification by column chromatography (petroleum ether/EtOAc 5:1) yielded **24** (63.3 mg, 64%) as a colourless oil. ^{1}H-NMR (400 MHz, $CDCl_3$): δ = 7.93 – 7.84 (m, 2H), 7.74 – 7.64 (m, 2H), 7.40 – 7.35 (m, 2H), 7.26 – 7.22 (m, 2H), 7.21 – 7.16 (m, 1H), 7.16 – 7.10 (m, 2H), 7.05 – 7.01 (m, 2H), 6.94 – 6.88 (m, 2H), 6.79 – 6.72 (m, 2H), 5.18 (dd, J = 8.6, 3.9 Hz, 1H), 4.36 (dd, J = 14.1, 8.6 Hz, 1H), 3.71 (s, 2H), 3.41 (dd, J = 14.1, 4.0 Hz, 1H), 2.39 (s, 3H). ^{13}C-NMR (100 MHz, $CDCl_3$): δ = 194.6, 162.3, 159.3, 155.3, 144.7, 136.7, 131.3, 130.1, 130.0, 129.8, 129.3, 128.6, 128.1, 124.9, 120.4, 117.2, 114.8, 59.4, 55.3, 46.6, 21.7. HR-MS (ESI) m/z calcd for $C_{29}H_{26}O_5S$ [M+H$^+$] 487.1574, found 487.1577.

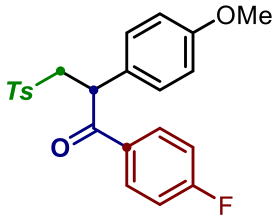

1-(4-Fluorophenyl)-2-(4-methoxyphenyl)-3-tosylpropan-1-one (25)

The general procedure **TP3** was followed using **1a** (0.4 mmol), **2c** (0.2 mmol) and **3f** (0.4 mmol) for 2 h. Purification by column chromatography (petroleum ether/EtOAc 6:1) yielded **25** (55.1 mg, 67%) as a colourless oil. ^{1}H-NMR (400 MHz, $CDCl_3$): δ = 7.98 – 7.89 (m, 2H), 7.76 – 7.63

(m, 2H), 7.26 – 7.22 (m, 2H), 7.16 – 7.10 (m, 2H), 7.09 – 7.00 (m, 2H), 6.82 – 6.73 (m, 2H), 5.18 (dd, J = 8.7, 3.8 Hz, 1H), 4.35 (dd, J = 14.1, 8.7 Hz, 1H), 3.72 (s, 3H), 3.39 (dd, J = 14.1, 3.8 Hz, 1H), 2.40 (s, 3H). ^{13}C-NMR (100 MHz, $CDCl_3$): δ = 194.6, 165.9 (d, $J_{C–F}$ = 255.7 Hz), 159.4, 144.8, 136.6, 132.0 (d, $J_{C–F}$ = 2.9 Hz), 131.6(d, $J_{C–F}$ = 9.3 Hz), 129.9, 129.3, 128.2, 128.1, 115.9 (d, $J_{C–F}$ = 22.0 Hz), 114.9, 59.4, 55.4, 46.8, 21.7. ^{19}F-NMR (376 MHz, $CDCl_3$): δ = -104.42. HR-MS (ESI) m/z calcd for $C_{23}H_{21}FO_4S$ [M+H$^+$] 413.1217, found 413.1216.

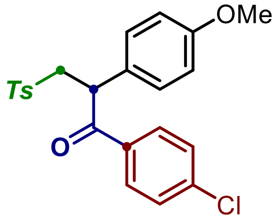

1-(4-Chlorophenyl)-2-(4-methoxyphenyl)-3-tosylpropan-1-one (26)

The general procedure **TP3** was followed using **1a** (0.4 mmol), **2c** (0.2 mmol) and **3g** (0.4 mmol) for 2 h. Purification by column chromatography (petroleum ether/EtOAc 6:1) yielded **26** (58.3 mg, 68%) as a colourless oil. ^{1}H-NMR (400 MHz, $CDCl_3$): δ = 7.86 – 7.80 (m, 2H), 7.73 – 7.63 (m, 2H), 7.37 – 7.31 (m, 2H), 7.26 – 7.20 (m, 2H), 7.15 – 7.07 (m, 2H), 6.80 – 6.71 (m, 2H), 5.17 (dd, J = 8.7, 3.8 Hz, 1H), 4.35 (dd, J = 14.1, 8.7 Hz, 1H), 3.71 (s, 3H), 3.40 (dd, J = 14.1, 3.8 Hz, 1H), 2.39 (s, 3H). ^{13}C-NMR (100 MHz, $CDCl_3$): δ = 195.0, 159.4, 144.8, 139.9, 136.6, 133.9, 130.3, 129.9, 129.3, 129.0, 128.1, 128.0, 114.9, 59.3, 55.3, 46.8, 21.7. HR-MS (ESI) m/z calcd for $C_{23}H_{21}ClO_4S$ [M+H$^+$] 429.0922, found 429.0922.

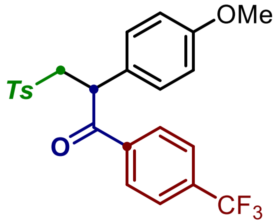

2-(4-Methoxyphenyl)-3-tosyl-1-[4-(trifluoromethyl)phenyl]propan-1-one (27)

The general procedure **TP3** was followed using **1a** (0.4 mmol), **2c** (0.2 mmol) and **3h** (0.4 mmol) for 2 h. Purification by column chromatography (petroleum ether/EtOAc 6:1) yielded **27** (59.2 mg, 64%) as a colourless oil. ^{1}H-NMR (400 MHz, $CDCl_3$): δ = 8.00 (d, J = 8.1 Hz, 2H), 7.71 (d, J = 8.1 Hz, 2H), 7.64 (d, J = 8.2 Hz, 2H), 7.25 (d, J = 7.7 Hz, 2H), 7.18 – 7.05 (m, 2H), 6.84 – 6.72 (m, 2H), 5.23 (dd, J = 8.9, 3.7 Hz, 1H), 4.38 (dd, J = 14.1, 8.9 Hz, 1H), 3.71 (s, 3H), 3.42 (dd, J = 14.1, 3.7 Hz, 1H), 2.39 (s, 3H). ^{13}C-NMR (100 MHz, $CDCl_3$): δ = 195.4, 159.6, 144.9, 138.5, 136.6, 134.5 (q, J_{C-F} = 32.8 Hz), 129.9, 129.4, 129.2, 128.1, 127.5, 125.7 (q, J_{C-F}

= 3.8 Hz), 123.6 (d, $J_{C\text{-}F}$ = 273.1 Hz), 115.0, 59.3, 55.3, 47.2, 21.6. ^{19}F-NMR (376 MHz, $CDCl_3$): δ = -63.21. HR-MS (ESI) m/z calcd for $C_{24}H_{21}F_3O_4S$ [M+H$^+$] 463.1185, found 463.1188.

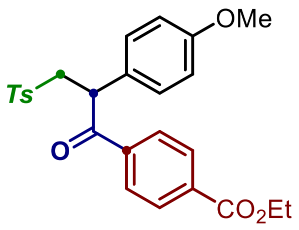

Ethyl 4-[2-(4-methoxyphenyl)-3-tosylpropanoyl]benzoate (28)

The general procedure **TP3** was followed using **1a** (0.4 mmol), **2c** (0.2 mmol) and **3i** (0.4 mmol) for 2 h. Purification by column chromatography (petroleum ether/EtOAc 5:1) yielded **28** (70.1 mg, 75%) as a colourless oil. ^{1}H-NMR (400 MHz, $CDCl_3$): δ = 8.07 – 8.00 (m, 2H), 7.95 – 7.89 (m, 2H), 7.72 – 7.65 (m, 2H), 7.26 – 7.20 (m, 2H), 7.14 – 7.07 (m, 2H), 6.79 – 6.69 (m, 2H), 5.21 (dd, J = 8.7, 3.8 Hz, 1H), 4.41 – 4.31 (m, 3H), 3.70 (s, 3H), 3.41 (dd, J = 14.1, 3.8 Hz, 1H), 2.38 (s, 3H), 1.37 (t, J = 7.1 Hz, 3H).^{13}C-NMR (100 MHz, $CDCl_3$): δ = 195.8, 165.6, 159.4, 144.8, 138.9, 136.5, 134.4, 129.9, 129.8, 129.4, 128.8, 128.1, 127.7, 114.9, 61.5, 59.2, 55.3, 47.2, 21.7, 14.3. HR-MS (ESI) m/z calcd for $C_{26}H_{26}O_6S$ [M+H$^+$] 467.1523, found 467.1521.

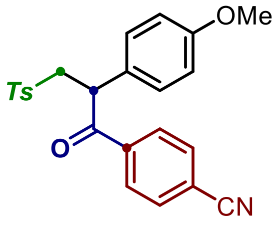

4-[2-(4-Methoxyphenyl)-3-tosylpropanoyl]benzonitrile (29)

The general procedure **TP3** was followed using **1a** (0.4 mmol), **2c** (0.2 mmol) and **3j** (0.4 mmol) for 2 h. Purification by column chromatography (petroleum ether/EtOAc 6:1) yielded **29** (52.1 mg, 62%) as a colourless oil. ^{1}H-NMR (400 MHz, $CDCl_3$): δ = 8.06 – 7.95 (m, 2H), 7.78 – 7.64 (m, 4H), 7.31 – 7.26 (m, 2H), 7.17 – 7.06 (m, 2H), 6.84 – 6.72 (m, 2H), 5.20 (dd, J = 9.1, 3.6 Hz, 1H), 4.36 (dd, J = 14.1, 9.1 Hz, 1H), 3.72 (s, 3H), 3.41 (dd, J = 14.0, 3.6 Hz, 1H), 2.42 (s, 3H). ^{13}C-NMR (100 MHz, $CDCl_3$): δ = 195.1, 159.7, 145.0, 138.9, 136.5, 132.5, 130.0, 129.4, 129.3, 128.1, 127.2, 117.9, 116.5, 115.1, 59.3, 55.4, 47.2, 21.7. HR-MS (ESI) m/z calcd for $C_{24}H_{21}NO_4S$ [M+H$^+$] 420.1264, found 420.1264.

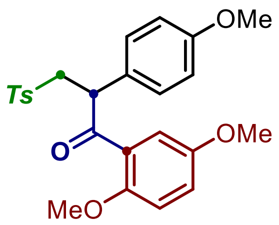

1-(2,5-Dimethoxyphenyl)-2-(4-methoxyphenyl)-3-tosylpropan-1-one (30)

The general procedure **TP4** was followed using **1a** (0.4 mmol), **2c** (0.2 mmol) and **3k** (0.6 mmol) for 2 h. Purification by column chromatography (petroleum ether/EtOAc 3:1) yielded **30** (32.3 mg, 36%) as a colourless oil. ^{1}H-NMR (400 MHz, $CDCl_3$): δ = 7.71 – 7.65 (m, 2H), 7.22 (d, J = 8.0 Hz, 2H), 7.08 (d, J = 3.2 Hz, 1H), 7.06 – 7.02 (m, 2H), 6.97 – 6.92 (m, 1H), 6.81 (d, J = 9.1 Hz, 1H), 6.73 – 6.67 (m, 2H), 5.46 (dd, J = 7.8, 4.9 Hz, 1H), 4.32 (dd, J = 14.3, 7.9 Hz, 1H), 3.84 (s, 3H), 3.71 (s, 6H), 3.39 (dd, J = 14.3, 5.0 Hz, 1H), 2.38 (s, 3H). ^{13}C-NMR (100 MHz, $CDCl_3$): δ = 197.7, 159.1, 153.5, 152.9, 144.5, 136.9, 129.7, 129.7, 128.6, 128.2, 126.8, 120.7, 115.1, 114.3, 113.3, 59.3, 56.2, 55.9, 55.3, 50.4, 21.7. HR-MS (ESI) m/z calcd for $C_{25}H_{26}O_6S$ [M+H$^+$] 455.1523, found 455.1526.

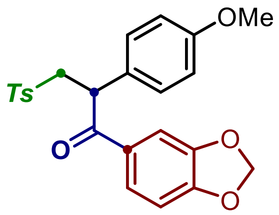

1-(Benzo[d][1,3]dioxol-5-yl)-2-(4-methoxyphenyl)-3-tosylpropan-1-one (31)

The general procedure **TP3** was followed using **1a** (0.4 mmol), **2c** (0.2 mmol) and **3l** (0.4 mmol) for 2 h. Purification by column chromatography (petroleum ether/EtOAc 5:1) yielded **31** (54.7 mg, 62%) as a colourless oil. ^{1}H-NMR (400 MHz, $CDCl_3$): δ = 7.68 (d, J = 8.1 Hz, 2H), 7.59 – 7.48 (m, 1H), 7.32 (d, J = 1.7 Hz, 1H), 7.23 (d, J = 8.0 Hz, 2H), 7.16 – 7.06 (m, 2H), 6.81 – 6.70 (m, 3H), 6.03 – 5.93 (m, 2H), 5.12 (dd, J = 8.6, 3.9 Hz, 1H), 4.33 (dd, J = 14.1, 8.6 Hz, 1H), 3.71 (s, 3H), 3.38 (dd, J = 14.2, 3.9 Hz, 1H), 2.39 (s, 3H). ^{13}C-NMR (100 MHz, $CDCl_3$): δ = 194.2, 159.3, 152.1, 148.3, 144.7, 136.7, 130.3, 129.8, 129.2, 128.8, 128.2, 125.4, 114.8, 108.7, 108.0, 102.0, 59.5, 55.3, 46.6, 21.7. HR-MS (ESI) m/z calcd for $C_{24}H_{22}O_6S$ [M+H$^+$] 439.1210, found 439.1211.

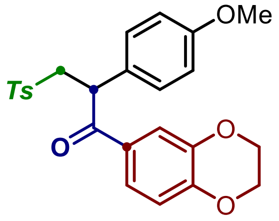

1-(2,3-Dihydrobenzo[b][1,4]dioxin-6-yl)-2-(4-methoxyphenyl)-3-tosylpropan-1-one (32)

The general procedure **TP3** was followed using **1a** (0.4 mmol), **2c** (0.2 mmol) and **3m** (0.4 mmol) for 2 h. Purification by column chromatography (petroleum ether/EtOAc 4:1) yielded **32** (75.3 mg, 83%) as a yellow solid. ^{1}H-NMR (400 MHz, $CDCl_3$): δ = 7.74 – 7.62 (m, 2H), 7.48 – 7.39 (m, 2H), 7.25 – 7.18 (m, 2H), 7.16 – 7.07 (m, 2H), 6.83 – 6.78 (m, 1H), 6.78 – 6.70 (m, 2H), 5.12 (dd, J = 8.5, 4.0 Hz, 1H), 4.32 (dd, J = 14.1, 8.6 Hz, 1H), 4.27 – 4.16 (m, 4H), 3.70 (s, 3H), 3.38 (dd, J = 14.2, 4.0 Hz, 1H), 2.38 (s, 3H). ^{13}C-NMR (100 MHz, $CDCl_3$): δ = 194.4, 159.2, 148.4, 144.6, 143.4, 136.6, 129.8, 129.3, 128.8, 128.1, 123.1, 118.5, 117.3, 114.8, 64.8, 64.1, 59.5, 55.3, 46.5, 21.7. HR-MS (ESI) m/z calcd for $C_{25}H_{24}O_6S$ [M+H$^+$] 453.1366, found 453.1369.

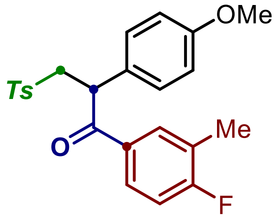

1-(4-Fluoro-3-methylphenyl)-2-(4-methoxyphenyl)-3-tosylpropan-1-one (33)

The general procedure **TP3** was followed using **1a** (0.4 mmol), **2c** (0.2 mmol) and **3n** (0.4 mmol) for 2 h. Purification by column chromatography (petroleum ether/EtOAc 4:1) yielded **32** (65.3 mg, 76%) as a colourless oil. ^{1}H-NMR (400 MHz, $CDCl_3$): δ = 7.81 – 7.64 (m, 4H), 7.24 (d, J = 8.0 Hz, 2H), 7.16 – 7.08 (m, 2H), 6.98 (t, J = 8.8 Hz, 1H), 6.81 – 6.72 (m, 2H), 5.19 (dd, J = 8.8, 3.9 Hz, 1H), 4.34 (dd, J = 14.1, 8.7 Hz, 1H), 3.71 (s, 3H), 3.40 (dd, J = 14.1, 3.9 Hz, 1H), 2.39 (s, 3H), 2.26 (d, J = 2.0 Hz, 3H). ^{13}C-NMR (100 MHz, $CDCl_3$): δ = 194.9, 164.5 (d, $J_{C\text{-}F}$ = 254.3 Hz), 159.4, 144.7, 136.7, 132.8 (d, $J_{C\text{-}F}$ = 6.6 Hz), 131.7 (d, $J_{C\text{-}F}$ = 3.4 Hz), 129.9, 129.3, 128.9 (d, $J_{C\text{-}F}$ = 9.5 Hz), 128.3, 128.1, 125.6 (d, $J_{C\text{-}F}$ = 17.6 Hz), 115.3 (d, $J_{C\text{-}F}$ = 22.9 Hz), 114.9, 59.4, 55.3, 46.7, 21.7, 14.7 (d, $J_{C\text{-}F}$ = 3.6 Hz). ^{19}F-NMR (376 MHz, $CDCl_3$): δ = -108.54. HR-MS (ESI) m/z calcd for $C_{24}H_{23}FO_4S$ [M+H$^+$] 427.1374, found 427.1373.

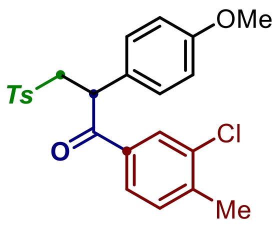

1-(3-Chloro-4-methylphenyl)-2-(4-methoxyphenyl)-3-tosylpropan-1-one (34)

The general procedure **TP3** was followed using **1a** (0.4 mmol), **2c** (0.2 mmol) and **3o** (0.4 mmol) for 2 h. Purification by column chromatography (petroleum ether/EtOAc 6:1) yielded **34** (65.1 mg, 73%) as a colourless oil. ^{1}H-NMR (400 MHz, $CDCl_3$): δ = 7.83 (d, J = 1.8 Hz, 1H), 7.72 – 7.64 (m, 3H), 7.26 – 7.18 (m, 3H), 7.15 – 7.08 (m, 2H), 6.80 – 6.71 (m, 2H), 5.15 (dd, J = 8.8, 3.8 Hz, 1H), 4.34 (dd, J = 14.1, 8.8 Hz, 1H), 3.70 (s, 3H), 3.40 (dd, J = 14.1, 3.8 Hz, 1H), 2.39 (s, 3H), 2.36 (s, 3H). ^{13}C-NMR (100 MHz, $CDCl_3$): δ = 194.7, 159.4, 144.8, 142.1, 136.5, 135.0, 134.8, 131.1, 129.9, 129.5, 129.3, 128.1, 128.0, 127.1, 114.9, 59.3, 55.3, 46.8, 21.7, 20.4. HR-MS (ESI) m/z calcd for $C_{24}H_{23}ClO_4S$ [M+H$^+$] 443.1078, found 443.1078.

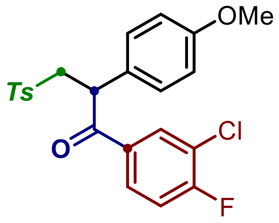

1-(3-Chloro-4-fluorophenyl)-2-(4-methoxyphenyl)-3-tosylpropan-1-one (35)

The general procedure **TP3** was followed using **1a** (0.4 mmol), **2c** (0.2 mmol) and **3p** (0.4 mmol) for 2 h. Purification by column chromatography (petroleum ether/EtOAc 6:1) yielded **35** (52.1 mg, 58%) as a colourless oil. ^{1}H-NMR (400 MHz, $CDCl_3$): δ = 7.98 – 7.90 (m, 1H), 7.84 – 7.78 (m, 1H), 7.74 – 7.66 (m, 2H), 7.29 – 7.23 (m, 2H), 7.18 – 7.08 (m, 3H), 6.81 – 6.73 (m, 2H), 5.13 (dd, J = 9.0, 3.6 Hz, 1H), 4.34 (dd, J = 14.1, 9.0 Hz, 1H), 3.73 (s, 3H), 3.38 (dd, J = 14.1, 3.6 Hz, 1H), 2.41 (s, 3H). ^{13}C-NMR (100 MHz, $CDCl_3$): δ = 193.8, 161.2 (d, $J_{C–F}$ = 257.8 Hz), 159.6, 145.0, 136.5, 132.8 (d, $J_{C–F}$ = 3.7 Hz), 131.9, 130.0, 129.4, 129.3, 128.1, 127.6, 122.1 (d, $J_{C–F}$ = 18.3 Hz), 116.9 (d, $J_{C–F}$ = 21.8 Hz), 115.1, 59.4, 55.4, 46.8, 21.7. ^{19}F-NMR (376 MHz, $CDCl_3$): δ = -106.81. HR-MS (ESI) m/z calcd for $C_{23}H_{20}ClFO_4S$ [M+H$^+$] 447.0828, found 447.0829.

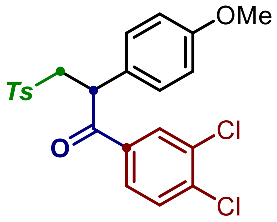

1-(3,4-Dichlorophenyl)-2-(4-methoxyphenyl)-3-tosylpropan-1-one (36)

The general procedure **TP3** was followed using **1a** (0.4 mmol), **2c** (0.2 mmol) and **3q** (0.4 mmol) for 2 h. Purification by column chromatography (petroleum ether/EtOAc 6:1) yielded **36** (66.2 mg, 71%) as a colourless oil. ^{1}H-NMR (400 MHz, $CDCl_3$): δ = 7.94 (d, J = 2.0 Hz, 1H), 7.71 (dd, J = 10.5, 8.2 Hz, 3H), 7.46 (d, J = 8.4 Hz, 1H), 7.26 (d, J = 8.1 Hz, 2H), 7.11 (d, J = 8.7 Hz, 2H), 6.78 (d, J = 8.6 Hz, 2H), 5.13 (dd, J = 8.9, 3.7 Hz, 1H), 4.34 (dd, J = 14.1, 9.0 Hz, 1H), 3.72 (s, 3H), 3.39 (dd, J = 14.1, 3.6 Hz, 1H), 2.41 (s, 3H). ^{13}C-NMR (100 MHz, $CDCl_3$): δ = 194.1, 159.6, 145.0, 138.0, 136.5, 135.2, 133.4, 130.8, 130.8, 130.0, 129.3, 128.1, 127.9, 127.4, 115.1, 59.3, 55.3, 46.9, 21.7. HR-MS (ESI) m/z calcd for $C_{23}H_{20}Cl_2O_4S$ [M+H$^+$] 463.0532, found 463.0536.

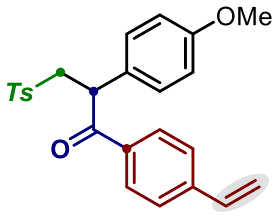

2-(4-Methoxyphenyl)-3-tosyl-1-(4-vinylphenyl)propan-1-one (37)

The general procedure **TP3** was followed using **1a** (0.4 mmol), **2c** (0.2 mmol) and **3r** (0.4 mmol) for 2 h. Purification by column chromatography (petroleum ether/EtOAc 6:1) yielded **37** (34.5 mg, 41%) as a colourless oil. ^{1}H-NMR (400 MHz, $CDCl_3$): δ = 7.89 – 7.83 (m, 2H), 7.70 – 7.67 (m, 2H), 7.42 – 7.37 (m, 2H), 7.24 – 7.20 (m, 2H), 7.16 – 7.09 (m, 2H), 6.78 – 6.64 (m, 3H), 5.83 (d, J = 17.6 Hz, 1H), 5.37 (d, J = 11.0 Hz, 1H), 5.21 (dd, J = 8.6, 4.0 Hz, 1H), 4.35 (dd, J = 14.2, 8.6 Hz, 1H), 3.71 (s, 3H), 3.41 (dd, J = 14.2, 4.0 Hz, 1H), 2.39 (s, 3H). ^{13}C-NMR (100 MHz, $CDCl_3$): δ = 195.5, 159.3, 144.7, 142.4, 136.6, 135.9, 134.7, 129.9, 129.4, 128.5, 128.2, 126.4, 117.1, 114.8, 59.4, 55.3, 46.8, 21.7. HR-MS (ESI) m/z calcd for $C_{25}H_{24}O_4S$ [M+H$^+$] 421.1468, found 421.1463.

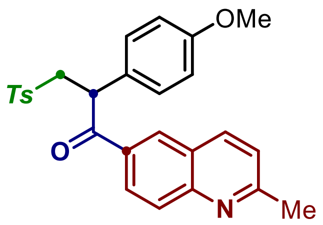

2-(4-Methoxyphenyl)-1-(2-methylquinolin-6-yl)-3-tosylpropan-1-one (38)

The general procedure **TP3** was followed using **1a** (0.4 mmol), **2c** (0.2 mmol) and **3s** (0.4 mmol) for 2 h. Purification by column chromatography (petroleum ether/EtOAc 1:1) yielded **38** (47.1 mg, 51%) as a colourless oil. ^{1}H-NMR (400 MHz, $CDCl_3$): δ = 8.38 (d, J = 2.0 Hz, 1H), 8.16 – 8.05 (m, 2H), 7.97 (d, J = 8.9 Hz, 1H), 7.77 – 7.62 (m, 2H), 7.36 – 7.31 (m, 1H), 7.26 – 7.11 (m, 4H), 6.86 – 6.69 (m, 2H), 5.37 (dd, J = 8.8, 3.8 Hz, 1H), 4.42 (dd, J = 14.1, 8.8 Hz, 1H), 3.70 (s, 3H), 3.45 (dd, J = 14.1, 3.7 Hz, 1H), 2.75 (s, 3H), 2.37 (s, 3H). ^{13}C-NMR (100 MHz, $CDCl_3$): δ = 195.6, 162.2, 159.4, 149.9, 144.8, 137.8, 136.6, 132.8, 130.3, 129.9, 129.4, 129.3, 128.3, 128.2, 125.7, 123.1, 115.0, 59.5, 55.4, 47.0, 25.7, 21.7. HR-MS (ESI) m/z calcd for $C_{27}H_{25}NO_4S$ [M+H$^+$] 460.1577, found 460.1579.

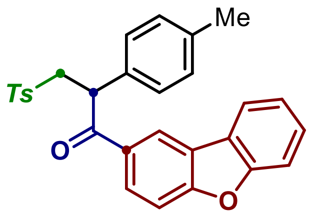

1-(Dibenzo[b,d]furan-2-yl)-2-(p-tolyl)-3-tosylpropan-1-one (39)

The general procedure **TP3** was followed using **1a** (0.4 mmol), **2a** (0.2 mmol) and **3t** (0.4 mmol) for 2 h. Purification by column chromatography (petroleum ether/EtOAc 6:1) yielded **39** (57.1 mg, 61%) as a colourless oil. ^{1}H-NMR (400 MHz, $CDCl_3$): δ = 8.54 (d, J = 1.8 Hz, 1H), 8.09 – 8.01 (m, 1H), 7.99 – 7.94 (m, 1H), 7.72 (d, J = 8.1 Hz, 2H), 7.58 – 7.46 (m, 3H), 7.41 – 7.36 (m, 1H), 7.25 – 7.14 (m, 4H), 7.07 (d, J = 8.0 Hz, 2H), 5.39 (dd, J = 8.8, 3.7 Hz, 1H), 4.47 (dd, J = 14.1, 8.7 Hz, 1H), 3.48 (dd, J = 14.1, 3.7 Hz, 1H), 2.37 (s, 3H), 2.24 (s, 3H). ^{13}C-NMR (100 MHz, $CDCl_3$): δ = 195.3, 159.1, 156.9, 144.8, 137.9, 136.6, 133.8, 130.9, 130.2, 129.9, 128.5, 128.2, 128.2, 128.1, 124.7, 123.7, 123.5, 122.4, 121.1, 112.0, 111.8, 59.6, 47.4, 21.7, 21.1. HR-MS (ESI) m/z calcd for $C_{29}H_{24}O_4S$ [M+H$^+$] 469.1468, found 469.1471.

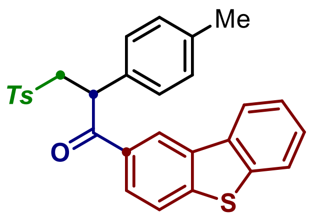

1-(Dibenzo[b,d]thiophen-2-yl)-2-(p-tolyl)-3-tosylpropan-1-one (40)

The general procedure **TP3** was followed using **1a** (0.4 mmol), **2a** (0.2 mmol) and **3u** (0.4 mmol) for 2 h. Purification by column chromatography (petroleum ether/EtOAc 8:1) yielded **40** (54.2 mg, 56%) as a colourless oil. ^{1}H-NMR (400 MHz, $CDCl_3$): δ = 8.68 (d, J = 1.6 Hz, 1H), 8.22 – 8.12 (m, 1H), 8.03 – 7.95 (m, 1H), 7.88 – 7.78 (m, 2H), 7.76 – 7.69 (m, 2H), 7.55 – 7.45 (m, 2H), 7.25 – 7.16 (m, 4H), 7.07 (d, J = 8.0 Hz, 2H), 5.39 (dd, J = 8.7, 3.8 Hz, 1H), 4.47 (dd, J = 14.1, 8.7 Hz, 1H), 3.48 (dd, J = 14.1, 3.8 Hz, 1H), 2.36 (s, 3H), 2.24 (s, 3H). ^{13}C-NMR (100 MHz, $CDCl_3$): δ = 195.7, 145.0, 144.8, 139.7, 138.0, 136.6, 135.7, 135.2, 133.7, 132.1, 130.2, 129.9, 128.2, 128.1, 127.6, 126.7, 125.1, 123.0, 122.8, 122.5, 122.0, 59.5, 47.5, 21.7, 21.1. HR-MS (ESI) m/z calcd for $C_{29}H_{24}O_3S_2$ [M+H$^+$] 485.1240, found 485.1244.

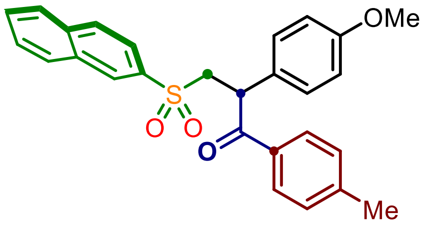

2-(4-Methoxyphenyl)-3-(naphthalen-2-ylsulfonyl)-1-(p-tolyl)propan-1-one (41)

The general procedure **TP3** was followed using **1b** (0.4 mmol), **2c** (0.2 mmol) and **3a** (0.4 mmol) for 2 h. Purification by column chromatography (petroleum ether/EtOAc 3:1) yielded **41** (72.9 mg, 82%) as a white powder. ^{1}H NMR (400 MHz, $CDCl_3$) δ = 8.27 (d, J = 0.9 Hz, 1H), 7.88 (dd, J = 13.5, 8.5 Hz, 2H), 7.81 – 7.75 (m, 2H), 7.71 (d, J = 8.2 Hz, 2H), 7.66 – 7.60 (m, 1H), 7.58 – 7.51 (m, 1H), 7.11 (t, J = 8.5 Hz, 4H), 6.66 (d, J = 8.7 Hz, 2H), 5.26 (dd, J = 8.2, 4.6 Hz, 1H), 4.39 (dd, J = 14.3, 8.2 Hz, 1H), 3.60 (s, 3H), 3.57 (dd, J = 14.4, 4.7 Hz, 1H), 2.31 (s, 3H). ^{13}C NMR (100 MHz, $CDCl_3$) δ = 195.5, 159.1, 144.2, 136.2, 135.1, 132.8, 131.9, 130.0, 129.5, 129.3, 129.3, 129.2, 129.1, 128.9, 128.2, 127.8, 127.4, 122.6, 114.6, 59.2, 55.1, 46.7, 21.6. HR-MS (ESI) m/z calcd for $C_{27}H_{24}O_4S$ [M+Na$^+$] 467.1288, found 467.1290.

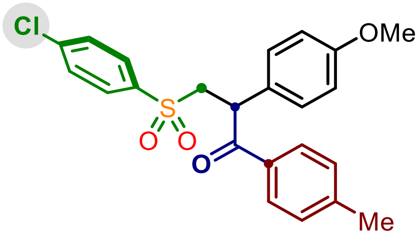

3-[(4-Chlorophenyl)sulfonyl]-2-(4-methoxyphenyl)-1-(p-tolyl)propan-1-one (42)

The general procedure **TP3** was followed using **1c** (0.4 mmol), **2c** (0.2 mmol) and **3a** (0.4 mmol) for 2 h. Purification by column chromatography (petroleum ether/EtOAc 3:1) yielded **42** (48.0 mg, 56%) as a yellow oil. ^{1}H NMR (400 MHz, $CDCl_3$) δ = 7.78 (d, *J* = 8.2 Hz, 2H), 7.74 – 7.67 (m, 2H), 7.40 – 7.36 (m, 2H), 7.18 (d, *J* = 8.1 Hz, 2H), 7.12 (d, *J* = 8.7 Hz, 2H), 6.74 (d, *J* = 8.7 Hz, 2H), 5.19 (dd, *J* = 8.2, 4.5 Hz, 1H), 4.32 (dd, *J* = 14.3, 8.3 Hz, 1H), 3.72 (s, 3H), 3.48 (dd, *J* = 14.3, 4.6 Hz, 1H), 2.36 (s, 3H). ^{13}C NMR (100 MHz, $CDCl_3$) δ = 195.4, 159.3, 144.4, 140.2, 138.0, 132.7, 129.5, 129.3, 129.3, 129.3, 128.9, 128.1, 114.7, 59.3, 55.2, 46.7, 21.6. HR-MS (ESI) m/z calcd for Chemical Formula: $C_{23}H_{21}ClO_4S$ [M+Na$^+$] 451.0741, found 451.0744.

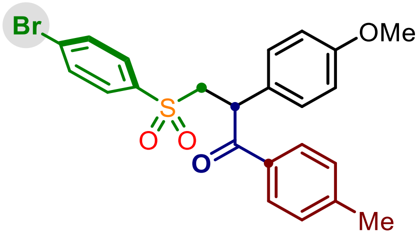

3-[(4-Bromophenyl)sulfonyl]-2-(4-methoxyphenyl)-1-(p-tolyl)propan-1-one (43)

The general procedure **TP3** was followed using **1d** (0.4 mmol), **2c** (0.2 mmol) and **3a** (0.4 mmol) for 2 h. Purification by column chromatography (petroleum ether/EtOAc 3:1) yielded **43** (61.5 mg, 65%) as a yellow oil. ^{1}H NMR (400 MHz, $CDCl_3$) δ = 7.78 (d, *J* = 8.2 Hz, 2H), 7.58 (dd, *J* = 30.9, 8.5 Hz, 4H), 7.18 (d, *J* = 8.1 Hz, 2H), 7.11 (d, *J* = 8.7 Hz, 2H), 6.74 (d, *J* = 8.7 Hz, 2H), 5.19 (dd, *J* = 8.2, 4.6 Hz, 1H), 4.31 (dd, *J* = 14.3, 8.2 Hz, 1H), 3.72 (s, 3H), 3.48 (dd, *J* = 14.3, 4.6 Hz, 1H), 2.36 (s, 3H). ^{13}C NMR (100 MHz, $CDCl_3$) δ = 195.4, 159.3, 144.4, 138.5, 132.7, 132.3, 129.5, 129.3, 129.3, 128.9, 128.8, 128.0, 114.7, 59.3, 55.2, 46.7, 21.6. HR-MS (ESI) m/z calcd for Chemical Formula: $C_{23}H_{21}BrO_4S$ [M+Na$^+$] 495.0236, found 495.0233.

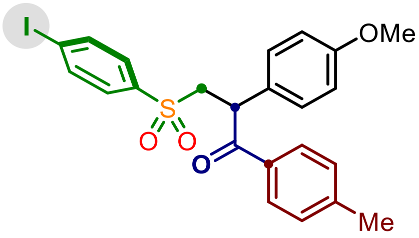

3-[(4-Iodophenyl)sulfonyl]-2-(4-methoxyphenyl)-1-(p-tolyl)propan-1-one (44)

The general procedure **TP3** was followed using **1e** (0.4 mmol), **2c** (0.2 mmol) and **3a** (0.4 mmol) for 2 h. Purification by column chromatography (petroleum ether/EtOAc 3:1) yielded **44** (57.2 mg, 55%) as a yellow oil. ^{1}H NMR (400 MHz, $CDCl_3$) δ = 7.77 (t, *J* = 7.8 Hz, 4H), 7.46 (d, *J* = 8.4 Hz, 2H), 7.18 (d, *J* = 8.1 Hz, 2H), 7.11 (d, *J* = 8.6 Hz, 2H), 6.74 (d, *J* = 8.6 Hz, 2H), 5.18 (dd, *J* = 8.1, 4.6 Hz, 1H), 4.31 (dd, *J* = 14.3, 8.2 Hz, 1H), 3.72 (s, 3H), 3.48 (dd, *J* = 14.3, 4.6 Hz, 1H), 2.36 (s, 3H). ^{13}C NMR (100 MHz, $CDCl_3$) δ = 195.4, 159.3, 144.4, 139.1, 138.3, 132.7, 129.3, 129.3, 129.3, 128.9, 128.0, 114.7, 101.5, 59.3, 55.2, 46.7, 21.6. HR-MS (ESI) m/z calcd for Chemical Formula: $C_{23}H_{21}IO_4S$ [M+Na$^+$] 543.0097, found 543.0099.

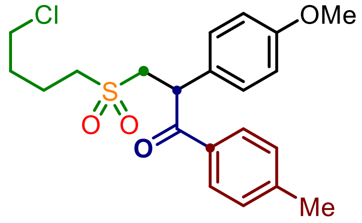

3-[(4-Chlorobutyl)sulfonyl]-2-(4-methoxyphenyl)-1-(p-tolyl)propan-1-one (45)

The general procedure **TP4** was followed using **1f** (0.4 mmol), **2c** (0.2 mmol) and **3a** (0.6 mmol) for 2 h. Purification by column chromatography (petroleum ether/EtOAc 5:1) yielded **45** (54.8 mg, 67%) as a colorless oil. ^{1}H NMR (400 MHz, $CDCl_3$) δ = 7.87 (d, *J* = 8.2 Hz, 2H), 7.24 (d, *J* = 8.7 Hz, 2H), 7.20 (d, *J* = 8.1 Hz, 2H), 6.84 (d, *J* = 8.7 Hz, 2H), 5.22 (dd, *J* = 7.8, 5.5 Hz, 1H), 4.09 (dd, *J* = 14.6, 7.8 Hz, 1H), 3.75 (s, 3H), 3.50 (t, *J* = 6.2 Hz, 2H), 3.35 (dd, *J* = 14.6, 5.4 Hz, 1H), 2.89 – 2.62 (m, 2H), 2.35 (s, 3H), 2.05 – 1.76 (m, 4H). ^{13}C NMR (100 MHz, $CDCl_3$) δ = 196.2, 159.4, 144.5, 132.7, 129.4, 129.4, 129.1, 128.3, 114.9, 56.1, 55.2, 53.4, 47.1, 43.8, 30.9, 21.6, 19.6. HR-MS (ESI) m/z calcd for $C_{21}H_{25}ClO_4S$ [M+Na$^+$] 431.1054, found 431.1056.

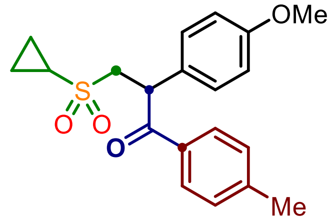

3-(Cyclopropylsulfonyl)-2-(4-methoxyphenyl)-1-(p-tolyl)propan-1-one (46)

The general procedure **TP3** was followed using **1g** (0.4 mmol), **2c** (0.2 mmol) and **3a** (0.4 mmol) for 2 h. Purification by column chromatography (petroleum ether/EtOAc 8:1) yielded **46** (38.0 mg, 53%) as a colorless oil. ^{1}H NMR (400 MHz, $CDCl_3$) δ = 7.89 (d, *J* = 8.2 Hz, 2H), 7.26 (d, *J* = 8.6 Hz, 2H), 7.20 (d, *J* = 8.1 Hz, 2H), 6.83 (d, *J* = 8.7 Hz, 2H), 5.23 (dd, *J* = 8.1, 4.9 Hz,

1H), 4.24 (dd, J = 14.4, 8.2 Hz, 1H), 3.74 (s, 3H), 3.38 (dd, J = 14.4, 4.9 Hz, 1H), 2.35 (s, 3H), 2.17 – 2.06 (m, 1H), 1.24 – 1.05 (m, 2H), 0.97 – 0.77 (m, 2H). ^{13}C NMR (100 MHz, $CDCl_3$) δ = 196.1, 159.3, 144.4, 132.8, 129.4, 129.4, 129.0, 128.7, 114.8, 57.1, 55.2, 47.0, 30.7, 21.6, 5.5, 4.7. HR-MS (ESI) m/z calcd for $C_{20}H_{22}O_4S$ [M+Na$^+$] 381.1131, found 381.1129.

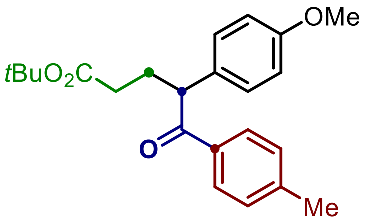

4-[1-(4-Chlorophenyl)-1-oxo-3-(pyridin-3-ylsulfonyl)propan-2-yl]phenyl acetate (47)

The general procedure **TP4 (TP5)** was followed using **1j** (0.4 mmol), **2c** (0.2 mmol) and **3a** (0.6 mmol) for 2 h. Purification by column chromatography (petroleum ether/EtOAc 20:1) yielded **47** [44.6 mg, 61% (57%)] as a colourless oil. ^{1}H-NMR (400 MHz, $CDCl_3$): δ = 7.85 (d, J = 8.2 Hz, 2H), 7.22 – 7.13 (m, 4H), 6.84 – 6.79 (m, 2H), 4.59 (t, J = 7.3 Hz, 1H), 3.74 (s, 3H), 2.40 – 2.31 (m, 4H), 2.19 (t, J = 7.1 Hz, 2H), 2.13 – 2.03 (m, 1H), 1.42 (s, 9H). ^{13}C-NMR (100 MHz, $CDCl_3$): δ = 199.4, 172.8, 158.8, 143.7, 134.3, 131.2, 129.4, 129.3, 128.9, 114.5, 80.3, 55.3, 51.4, 33.2, 29.1, 28.2, 21.7. HR-MS (ESI) m/z calcd for $C_{23}H_{28}O_4$ [M+Na$^+$] 391.1880, found 391.1878.

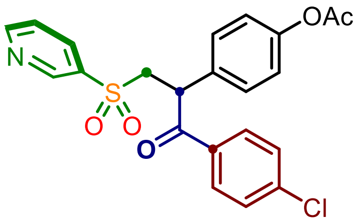

***tert*-Butyl 4-(4-methoxyphenyl)-5-oxo-5-(p-tolyl)pentanoate (51)**

The general procedure **TP5** was followed using **1i** (0.4 mmol), **2k** (0.2 mmol) and **3g** (0.6 mmol) for 2 h. Purification by column chromatography (petroleum ether/EtOAc 1.5:1) yielded **51** (46.2 mg, 52%) as a colourless oil. ^{1}H-NMR (400 MHz, $CDCl_3$): δ = 9.00 (d, J = 2.4 Hz, 1H), 8.85 – 8.73 (m, 1H), 8.06 – 7.97 (m, 1H), 7.90 – 7.78 (m, 2H), 7.44 – 7.33 (m, 3H), 7.25 – 7.18 (m, 2H), 7.02 – 6.93 (m, 2H), 5.27 (dd, J = 8.5, 4.5 Hz, 1H), 4.36 (dd, J = 14.3, 8.5 Hz, 1H), 3.54 (dd, J = 14.4, 4.5 Hz, 1H), 2.24 (s, 3H). ^{13}C-NMR (100 MHz, $CDCl_3$): δ = 194.6, 169.2, 154.4, 150.7, 149.0, 140.5, 136.1, 135.8, 133.4, 132.9, 130.4, 129.3, 123.9, 122.9, 59.5, 47.0, 21.2. HR-MS (ESI) m/z calcd for $C_{22}H_{18}ClNO_5S$ [M+H$^+$] 444.0667, found 444.0669.

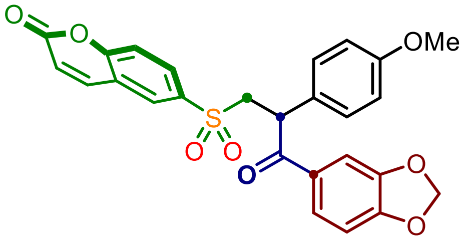

6-{[3-(Benzo[d][1,3]dioxol-5-yl)-2-(4-methoxyphenyl)-3-oxopropyl]sulfonyl}-2H-chromen-2-one (52)

The general procedure **TP5** was followed using **1h** (0.4 mmol), **2c** (0.2 mmol) and **3l** (0.6 mmol) for 2 h. Purification by column chromatography (petroleum ether/EtOAc 1.5:1) yielded **52** (55.7 mg, 57%) as a white solid. ^{1}H-NMR (400 MHz, $CDCl_3$): δ = 7.95 – 7.83 (m, 2H), 7.61 (d, J = 9.7 Hz, 1H), 7.56 – 7.48 (m, 1H), 7.35 (d, J = 8.5 Hz, 1H), 7.26 (d, J = 9.6 Hz, 2H), 7.16 – 7.07 (m, 2H), 6.81 – 6.68 (m, 3H), 6.48 (d, J = 9.7 Hz, 1H), 5.99 (dd, J = 5.3, 1.3 Hz, 2H), 5.15 (dd, J = 8.4, 4.4 Hz, 1H), 4.36 (dd, J = 14.3, 8.5 Hz, 1H), 3.69 (s, 3H), 3.53 (dd, J = 14.4, 4.4 Hz, 1H). ^{13}C-NMR (100 MHz, $CDCl_3$): δ = 193.9, 159.4, 159.2, 157.0, 152.3, 148.3, 142.3, 135.7, 131.1, 130.0, 129.3, 129.0, 128.0, 125.4, 118.9, 118.3, 118.0, 114.8, 108.5, 108.1, 102.2, 59.7, 55.3, 46.8. HR-MS (ESI) m/z calcd for $C_{26}H_{20}O_8S$ [M+Na$^+$] 515.0771, found 515.0768.

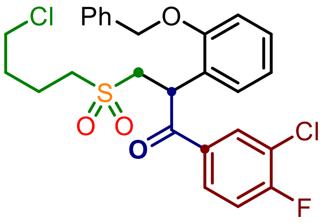

2-[2-(Benzyloxy)phenyl]-1-(3-chloro-4-fluorophenyl)-3-[(4-chlorobutyl)sulfonyl]propan-1-one (53)

The general procedure **TP5** was followed using **1f** (0.4 mmol), **2j** (0.2 mmol) and **3p** (0.6 mmol) for 2 h. Purification by column chromatography (petroleum ether/EtOAc 5:1) yielded **53** (48.3 mg, 46%) as a colourless oil. ^{1}H-NMR (400 MHz, $CDCl_3$): δ = 8.11 – 8.03 (m, 1H), 7.87 – 7.79 (m, 1H), 7.53 – 7.38 (m, 5H), 7.27 – 7.23 (m, 1H), 7.12 (dd, J = 7.7, 1.6 Hz, 1H), 7.08 – 6.98 (m, 2H), 6.92 (t, J = 7.5 Hz, 1H), 5.63 (dd, J = 8.3, 4.5 Hz, 1H), 5.20 (s, 2H), 4.16 (dd, J = 14.5, 8.3 Hz, 1H), 3.48 (t, J = 6.2 Hz, 2H), 3.28 (dd, J = 14.5, 4.5 Hz, 1H), 2.90 – 2.75 (m, 2H), 1.97 – 1.76 (m, 4H). ^{13}C-NMR (100 MHz, $CDCl_3$): δ = 194.7, 161.2 (d, $J_{C\text{-}F}$ = 257.9 Hz), 155.0, 136.2, 132.6 (d, $J_{C\text{-}F}$ = 3.7 Hz), 131.9, 130.0, 129.4 (d, $J_{C\text{-}F}$ = 8.5 Hz), 129.0, 128.9, 128.6, 127.7, 124.4, 122.0, 121.9 (d, $J_{C\text{-}F}$ = 18.3 Hz), 116.8 (d, $J_{C\text{-}F}$ = 21.7 Hz), 113.0, 70.8, 55.0, 53.3, 44.0,

41.3, 31.0, 19.7. ^{19}F-NMR (376 MHz, $CDCl_3$): δ = -106.82. HR-MS (ESI) m/z calcd for $C_{26}H_{25}Cl_2FO_4S$ [M+Na$^+$] 545.0727, found 545.0726.

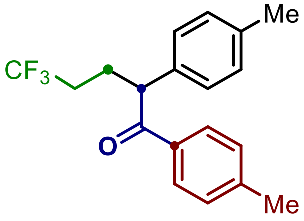

5,5,5-Trifluoro-1,2-di-p-tolylpentan-1-one (54)

The general procedure **TP4 (TP5)** was followed using **1k** (0.4 mmol), **2a** (0.2 mmol) and **3a** (0.6 mmol) for 2 h. Purification by column chromatography (petroleum ether/EtOAc 20:1) yielded **54** [28.8 mg, 45% (55%)] as a colourless oil. ^{1}H-NMR (400 MHz, $CDCl_3$): δ = 7.84 (d, J = 8.3 Hz, 2H), 7.23 – 7.01 (m, 6H), 4.53 (t, J = 7.0 Hz, 1H), 2.40 – 2.31 (m, 4H), 2.28 (s, 3H), 2.15 – 1.92 (m, 3H). ^{13}C-NMR (100 MHz, $CDCl_3$): δ = 198.4, 144.1, 137.3, 135.6, 133.9, 130.1, 129.4, 129.0, 128.1, 51.9, 33.1 – 30.5 (m), 27.3 – 24.9 (m), 21.7, 21.2. ^{19}F-NMR (376 MHz, $CDCl_3$): δ = -65.89. HR-MS (ESI) m/z calcd for $C_{19}H_{19}F_3O$ [M+Na$^+$] 343.1280, found 343.1272.

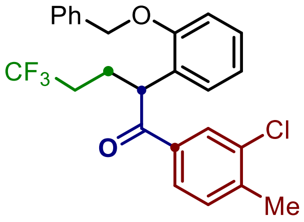

2-(2-(benzyloxy)phenyl)-1-(3-chloro-4-methylphenyl)-5,5,5-trifluoropentan-1-one (55)

The general procedure **TP5** was followed using **1k** (0.4 mmol), **2j** (0.2 mmol) and **3o** (0.6 mmol) for 2 h. Purification by column chromatography (petroleum ether/EtOAc 40:1) yielded **55** (42 mg, 47%) as a colourless oil. ^{1}H NMR (400 MHz, $CDCl_3$) δ = 7.95 (s, 1H), 7.70 (d, J = 7.9 Hz, 1H), 7.49 (d, J = 7.1 Hz, 2H), 7.44 (t, J = 7.2 Hz, 2H), 7.41 – 7.35 (m, 1H), 7.22 – 7.16 (m, 1H), 7.14 (d, J = 7.9 Hz, 1H), 7.08 (d, J = 7.6 Hz, 1H), 6.99 (d, J = 8.2 Hz, 1H), 6.88 (t, J = 7.5 Hz, 1H), 5.19 (s, 2H), 5.06 (t, J = 6.9 Hz, 1H), 2.43 – 2.25 (m, 4H), 2.24 – 1.86 (m, 4H). ^{13}C NMR (100 MHz, $CDCl_3$) δ = 197.8, 155.1, 141.5, 136.5, 135.5, 134.7, 130.9, 129.2, 128.8, 128.7, 128.2, 128.2, 127.5, 126.8, 126.7, 121.6, 112.2, 70.5, 44.1, 31.5 (q, J_{C-F} = 28.8 Hz), 25.0, 20.2. ^{19}F NMR (376 MHz, $CDCl_3$) δ = -66.19 (s). HR-MS (ESI) m/z calcd for $C_{25}H_{22}ClF_3O_2$ [M+Na$^+$] 469.1153, found 469.1151.

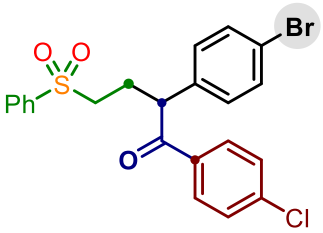

2-(4-Bromophenyl)-1-(4-chlorophenyl)-4-(phenylsulfonyl)butan-1-one (56)

The general procedure **TP5** was followed using **1l** (0.4 mmol), **2n** (0.2 mmol) and **3g** (0.6 mmol) for 2 h. Purification by column chromatography (petroleum ether/EtOAc 6:1) yielded **56** (61.2 mg, 60%) as a colourless oil. ^{1}H-NMR (400 MHz, $CDCl_3$): δ = 7.94 – 7.73 (m, 4H), 7.65 (t, J = 7.4 Hz, 1H), 7.56 (t, J = 7.6 Hz, 2H), 7.47 – 7.29 (m, 4H), 7.16 – 7.04 (m, 2H), 4.83 (t, J = 7.4 Hz, 1H), 3.16 – 2.97 (m, 2H), 2.56 – 2.43 (m, 1H), 2.32 – 2.19 (m, 1H). ^{13}C-NMR (100 MHz, $CDCl_3$): δ = 197.1, 140.1, 139.1, 136.8, 134.2, 134.0, 132.7, 130.3, 130.0, 129.5, 129.2, 128.1, 122.1, 53.6, 50.9, 26.7. HR-MS (ESI) m/z calcd for $C_{22}H_{18}BrClO_3S$ [M+Na$^+$] 498.9741, found 498.9745.

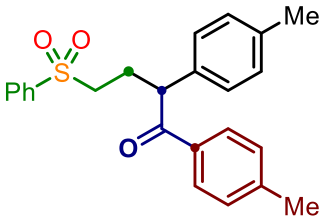

4-(Phenylsulfonyl)-1,2-di-p-tolylbutan-1-one (57)

The general procedure **TP5** was followed using **1l** (0.4 mmol), **2a** (0.2 mmol) and **3a** (0.6 mmol) for 2 h. Purification by column chromatography (petroleum ether/EtOAc 6:1) yielded **57** (57.3mg, 73%) as a yellow oil. ^{1}H NMR (400 MHz, $CDCl_3$) δ = 7.88 (d, J = 7.5 Hz, 2H), 7.80 (d, J = 8.1 Hz, 2H), 7.63 (d, J = 7.4 Hz, 1H), 7.56 (d, J = 7.8 Hz, 2H), 7.15 (d, J = 8.0 Hz, 2H), 7.08 (d, J = 4.0 Hz, 4H), 4.72 (t, J = 7.4 Hz, 1H), 3.13 (ddd, J = 14.9, 8.6, 6.6 Hz, 1H), 2.41 (dd, J = 14.8, 7.2 Hz, 1H), 2.33 (s, 3H), 2.26 (s, 4H). ^{13}C NMR (100 MHz, CDCl3) δ = 198.1, 144.0, 139.0, 137.3, 135.0, 133.6, 133.5, 129.9, 129.3, 129.2, 128.9, 128.0, 53.9, 51.0, 26.8, 21.6, 21.0. HR-MS (ESI) m/z calcd for $C_{24}H_{24}O_3S$ [M+Na$^+$] 415.1338, found 415.1335.

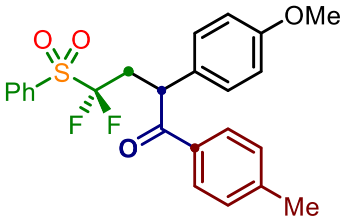

4,4-Difluoro-2-(4-methoxyphenyl)-4-(phenylsulfonyl)-1-(p-tolyl)butan-1-one (58)

The general procedure **TP4 (TP5)** was followed using **1m** (0.4 mmol), **2c** (0.2 mmol) and **3a** (0.6 mmol) for 2 h. Purification by column chromatography (petroleum ether/EtOAc 8:1) yielded **58** [68.3 mg, 77% (80%)] as a colourless oil. ^{1}H-NMR (400 MHz, $CDCl_3$): δ = 7.99 – 7.93 (m, 2H), 7.90 – 7.80 (m, 2H), 7.75 – 7.69 (m, 1H), 7.58 (t, J = 7.9 Hz, 2H), 7.26 – 7.22 (m, 2H), 7.18 (d, J = 8.0 Hz, 2H), 6.85 – 6.76 (m, 2H), 5.09 (dd, J = 8.1, 4.9 Hz, 1H), 3.73 (s, 3H), 3.65 – 3.46 (m, 1H), 2.86 – 2.70 (m, 1H), 2.34 (s, 3H). ^{13}C-NMR (100 MHz, $CDCl_3$): δ = 196.5, 159.2, 144.2, 135.5, 133.3, 132.4, 130.9, 129.8, 129.4, 129.4, 129.3, 129.1, 124.0 (t, J_{C-F} = 286.0 Hz), 114.8, 55.3, 45.4, 33.2 (t, J_{C-F} = 18.9 Hz), 21.7. ^{19}F-NMR (376 MHz, $CDCl_3$): δ = -100.64 (d, J = 229.2 Hz), -101.73 (d, J = 229.1 Hz). HR-MS (ESI) m/z calcd for $C_{24}H_{22}F_2O_4S$ [M+Na$^+$] 467.1099, found 467.1105.

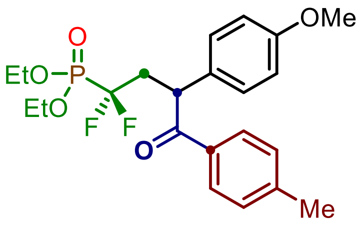

Diethyl [1,1-difluoro-3-(4-methoxyphenyl)-4-oxo-4-(p-tolyl)butyl]phosphonate (59)

The general procedure **TP4 (TP5)** was followed using **1n** (0.4 mmol), **2c** (0.2 mmol) and **3a** (0.6 mmol) for 2 h. Purification by column chromatography (petroleum ether/EtOAc 3:1) yielded **59** [30.1 mg, 34% (30%)] as a colourless oil. ^{1}H-NMR (400 MHz, $CDCl_3$): δ = 7.88 (d, J = 6.4 Hz, 2H), 7.25 – 7.15 (m, 4H), 6.82 – 6.77 (m, 2H), 5.07 (dd, J = 8.5, 3.8 Hz, 1H), 4.32 – 4.20 (m, 4H), 3.73 (s, 3H), 3.48 – 3.26 (m, 1H), 2.44 – 2.30 (m, 4H), 1.38 – 1.33 (m, 6H). ^{13}C-NMR (100 MHz, $CDCl_3$): δ = 197.5, 158.9, 144.0, 133.6, 130.8, 129.4, 129.3, 129.1, 120.3 (d, J_{C-F} = 215.1 Hz), 114.6, 64.7 (dd, J_{C-F} = 8.9, 6.9 Hz), 55.3, 47.7 – 43.1 (m), 40.5 – 35.7 (m), 21.7, 16.5 (d, J_{C-F} = 5.2 Hz). ^{19}F-NMR (376 MHz, $CDCl_3$): δ = -110.37 , -110.65. HR-MS (ESI) m/z calcd for $C_{22}H_{27}F_2O_5P$ [M+H$^+$] 441.1637, found 441.1635.

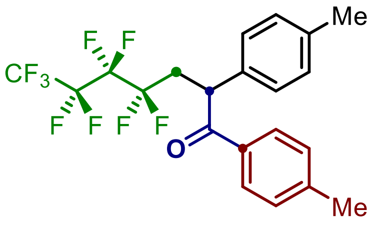

4,4,5,5,6,6,7,7,7-Nonafluoro-1,2-di-p-tolylheptan-1-one (60)

The general procedure **TP4 (TP5)** was followed using **1o** (0.4 mmol), **2a** (0.2 mmol) and **3a** (0.6 mmol) for 2 h. Purification by column chromatography (petroleum ether/EtOAc 40:1) yielded **60** [48.4 mg, 53% (60%)] as a colourless oil. ^{1}H-NMR (400 MHz, $CDCl_3$): δ = 7.89 (d, J = 8.3 Hz, 2H), 7.21 (d, J = 7.8 Hz, 4H), 7.12 (d, J = 7.9 Hz, 2H), 4.99 (dd, J = 8.3, 4.2 Hz, 1H), 3.54 – 3.34 (m, 1H), 2.52 – 2.39 (m, 1H), 2.36 (s, 3H), 2.28 (s, 3H). ^{13}C-NMR (100 MHz, $CDCl_3$): δ = 196.5, 144.4, 137.7, 135.0, 133.2, 130.2, 129.5, 129.1, 128.0, 45.2, 34.4 (t, J_{C-F} = 20.7 Hz), 21.7, 21.1. ^{19}F-NMR (376 MHz, $CDCl_3$): δ = -81.05 (t, J = 10.1 Hz), -110.51 – -116.20 (m), -121.54 – -128.17 (m). HR-MS (ESI) m/z calcd for $C_{21}H_{17}F_9O$ [M+Na^{+}] 479.1028, found 479.1026.

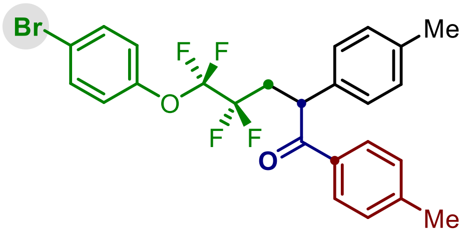

5-(4-Bromophenoxy)-4,4,5,5-tetrafluoro-1,2-di-p-tolylpentan-1-one (61)

The general procedure **TP4 (TP5)** was followed using **1p** (0.4 mmol), **2a** (0.2 mmol) and **3a** (0.6 mmol) for 2 h. Purification by column chromatography (petroleum ether/EtOAc 40:1) yielded **61** [41.8 mg, 41% (42%)] as a colourless oil. ^{1}H-NMR (400 MHz, $CDCl_3$): δ = 7.90 (d, J = 8.3 Hz, 2H), 7.57 – 7.40 (m, 2H), 7.26 – 7.15 (m, 4H), 7.15 – 6.96 (m, 4H), 5.03 (dd, J = 8.3, 4.3 Hz, 1H), 3.60 – 3.36 (m, 1H), 2.58 – 2.41 (m, 1H), 2.36 (s, 3H), 2.28 (s, 3H). ^{13}C-NMR (100 MHz, $CDCl_3$): δ = 197.0, 148.2, 144.2, 137.5, 135.6, 133.4, 132.8, 130.1, 129.5, 129.1, 128.1, 123.7, 119.8, 45.5, 34.8 (t, J_{C-F} = 21.1 Hz), 21.8, 21.2. ^{19}F-NMR (376 MHz, $CDCl_3$): δ = -88.30 , -115.14 (d, J = 259.2 Hz), -116.78 (d, J = 261.1 Hz). HR-MS (ESI) m/z calcd for $C_{25}H_{21}BrF_4O_2$ [M+H^{+}] 509.0734, found 509.0734.

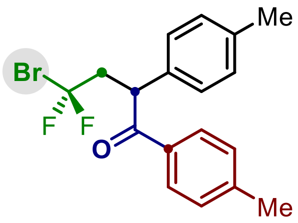

4-Bromo-4,4-difluoro-1,2-di-p-tolylbutan-1-one (62)

The general procedure **TP4 (TP5)** was followed using **1q** (0.4 mmol), **2a** (0.2 mmol) and **3a** (0.6 mmol) for 2 h. Purification by column chromatography (petroleum ether/EtOAc 40:1) yielded **62** [49.2 mg, 67% (75%)] as a colourless oil. ^{1}H-NMR (400 MHz, $CDCl_3$): δ = 7.89 (d,

J = 8.3 Hz, 2H), 7.26 – 7.15 (m, 4H), 7.11 (d, J = 7.9 Hz, 2H), 4.95 (dd, J = 7.7, 4.6 Hz, 1H), 3.77 – 3.58 (m, 1H), 2.88 – 2.71 (m, 1H), 2.36 (s, 3H), 2.28 (s, 3H). ^{13}C-NMR (100 MHz, $CDCl_3$): δ = 196.5, 144.3, 137.6, 134.8, 133.4, 130.1, 129.5, 129.1, 128.1, 121.9 (t, $J_{C\text{-}F}$ = 306.7 Hz), 48.3 (t, $J_{C\text{-}F}$ = 2.1 Hz), 47.6 (t, $J_{C\text{-}F}$ = 21.0 Hz), 21.8, 21.2. ^{19}F-NMR (376 MHz, $CDCl_3$): δ = -42.50 (d, J = 155.4 Hz), -43.32 (d, J = 155.4 Hz). HR-MS (ESI) m/z calcd for $C_{18}H_{17}BrF_2O$ [M+Na$^+$] 389.0323, found 389.0314.

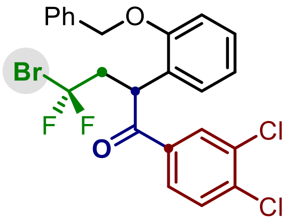

2-(2-(benzyloxy)phenyl)-4-bromo-1-(3,4-dichlorophenyl)-4,4-difluorobutan-1-one (63)

The general procedure **TP5** was followed using **1q** (0.4 mmol), **2j** (0.2 mmol) and **3q** (0.6 mmol) for 2 h. Purification by column chromatography (petroleum ether/EtOAc 40:1) yielded **63** (59.6 mg, 58%) as a colourless oil. ^{1}H NMR (400 MHz, $CDCl_3$) δ = 8.08 (d, J = 1.7 Hz, 1H), 7.77 (dd, J = 8.4, 1.8 Hz, 1H), 7.50 (d, J = 7.3 Hz, 2H), 7.44 (t, J = 7.3 Hz, 2H), 7.39 (d, J = 7.0 Hz, 1H), 7.34 (d, J = 8.4 Hz, 1H), 7.22 (t, J = 7.8 Hz, 1H), 7.10 (d, J = 7.4 Hz, 1H), 6.98 (d, J = 8.3 Hz, 1H), 6.90 (t, J = 7.5 Hz, 1H), 5.52 – 5.38 (m, 1H), 5.19 (s, 2H), 3.60 (ddd, J = 27.9, 15.1, 7.1 Hz, 1H), 2.77 (ddd, J = 28.7, 15.1, 5.2 Hz, 1H). ^{13}C NMR (100 MHz, $CDCl_3$) δ = 195.1, 154.8, 137.7, 136.3, 135.2, 133.1, 130.7, 130.5, 129.4, 128.7, 128.4, 128.3, 127.7, 127.6, 125.3, 121.6, 121.5 (t, J = 306.5 Hz), 112.5, 70.6, 46.6 (t, J = 21.3 Hz), 40.9. ^{19}F NMR (376 MHz, $CDCl_3$) δ = -43.28 (dd). HR-MS (ESI) m/z calcd for $C_{23}H_{17}BrCl_2F_2O_2$ [M+Na$^+$] 534.9649, found 534.9652.

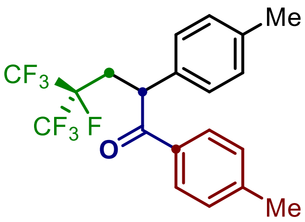

4,5,5,5-Tetrafluoro-1,2-di-p-tolyl-4-(trifluoromethyl)pentan-1-one (64)

The general procedure **TP5** was followed using **1r** (0.4 mmol), **2a** (0.2 mmol) and **3a** (0.6 mmol) for 2 h. Purification by column chromatography (petroleum ether/EtOAc 40:1) yielded **64** (45.5 mg, 56%) as a colourless oil. ^{1}H-NMR (400 MHz, $CDCl_3$): δ = 7.86 (d, J = 8.1 Hz, 2H), 7.24 – 7.14 (m, 4H), 7.11 (d, J = 7.9 Hz, 2H), 4.98 (dd, J = 9.3, 2.7 Hz, 1H), 3.69 – 3.53 (m, 1H), 2.35

(s, 3H), 2.28 (s, 4H). ^{13}C-NMR (100 MHz, $CDCl_3$): δ = 196.2, 144.3, 137.8, 135.4, 133.2, 130.3, 129.5, 129.0, 127.8, 46.8 – 44.5 (m), 31.7 (d, J_{C-F} = 18.8 Hz), 21.7, 21.1. ^{19}F-NMR (376 MHz, $CDCl_3$): δ = -69.86 – -82.43 (m), -174.08 – -198.07 (m). HR-MS (ESI) m/z calcd for $C_{20}H_{17}F_7O$ [M+H$^+$] 407.1240, found 407.1244.

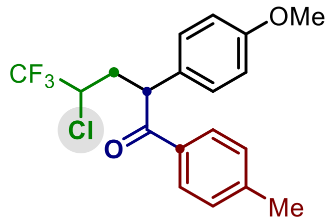

4-Chloro-5,5,5-trifluoro-2-(4-methoxyphenyl)-1-(p-tolyl)pentan-1-one (65)

The general procedure **TP4 (TP5)** was followed using **1s** (0.4 mmol), **2c** (0.2 mmol) and **3a** (0.6 mmol) for 2 h. Purification by column chromatography (petroleum ether/EtOAc 20:1) yielded **65** [49.7 mg, 67% (70%), dr = 1:1] as a colourless oil. ^{1}H-NMR (400 MHz, $CDCl_3$): δ = 7.88 – 7.82 (m, 2H), 7.26 – 7.21 (m, 2H), 7.18 (d, J = 7.9 Hz, 2H), 6.89 – 6.82 (m, 2H), 4.80 (dd, J = 11.1, 4.0 Hz, 1H), 3.76 (s, 4H), 2.73 – 2.63 (m, 1H), 2.38 – 2.30 (m, 4H). ^{13}C-NMR (100 MHz, $CDCl_3$): δ = 197.7, 159.4, 144.2, 133.4, 129.5, 129.4, 129.1, 128.5, 115.1, 55.9 (d, J_{C-F} = 33.5 Hz), 55.4, 48.8, 34.5, 21.8. ^{19}F-NMR (376 MHz, $CDCl_3$): δ = -74.51. HR-MS (ESI) m/z calcd for $C_{19}H_{18}ClF_3O_2$ [M+H$^+$] 371.1020, found 371.1017.

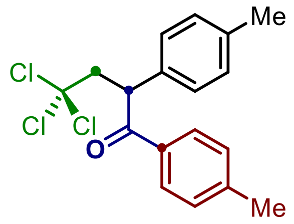

4,4,4-Trichloro-1,2-di-p-tolylbutan-1-one (66)

The general procedure **TP5** was followed using **1t** (0.4 mmol), **2a** (0.2 mmol) and **3a** (0.6 mmol) for 2 h. Purification by column chromatography (petroleum ether/EtOAc 80:1) yielded **66** (49.1 mg, 72%) as a yellow oil. ^{1}H NMR (400 MHz, $CDCl_3$) δ = 7.91 (d, J = 8.2 Hz, 2H), 7.21 (d, J = 7.9 Hz, 4H), 7.10 (d, J = 8.0 Hz, 2H), 5.08 (dd, J = 7.8, 2.4 Hz, 1H), 4.24 (dd, J = 14.9, 7.8 Hz, 1H), 2.99 (dd, J = 14.9, 2.4 Hz, 1H), 2.36 (s, 3H), 2.27 (s, 3H). ^{13}C NMR (100 MHz, $CDCl_3$) δ = 196.9, 144.1, 137.4, 135.2, 133.6, 130.0, 129.4, 129.0, 128.0, 98.8, 77.4, 77.0, 76.7, 57.5, 50.6, 21.6, 21.0. HR-MS (ESI) m/z calcd for $C_{18}H_{17}Cl_3O$ [M+Na$^+$] 377.0237, found 377.0239.

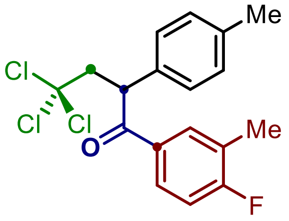

4,4,4-Trichloro-1-(4-fluoro-3-methylphenyl)-2-(p-tolyl)butan-1-one (67)

The general procedure **TP5** was followed using **1t** (4.0 mmol), **2a** (2.0 mmol) and **3n** (6.0 mmol) for 2 h. Purification by column chromatography (petroleum ether/EtOAc 20:1) yielded **67** (568.1 mg, 76%) as a colourless oil. ^{1}H-NMR (400 MHz, $CDCl_3$): δ = 7.94 – 7.79 (m, 2H), 7.24 – 7.17 (m, 2H), 7.12 (d, J = 7.8 Hz, 2H), 7.02 (t, J = 8.8 Hz, 1H), 5.05 (dd, J = 7.9, 2.5 Hz, 1H), 4.24 (dd, J = 14.9, 7.9 Hz, 1H), 2.99 (dd, J = 14.9, 2.5 Hz, 1H), 2.29 (d, J = 2.7 Hz, 6H). ^{13}C-NMR (100 MHz, $CDCl_3$): δ = 196.2, 164.5 (d, $J_{C\text{-}F}$ = 254.1 Hz), 137.7, 135.0, 132.8 (d, $J_{C\text{-}F}$ = 6.6 Hz), 132.3 (d, $J_{C\text{-}F}$ = 3.6 Hz), 130.2, 128.9 (d, $J_{C\text{-}F}$ = 9.4 Hz), 128.1, 125.7 (d, $J_{C\text{-}F}$ = 17.9 Hz), 115.4 (d, $J_{C\text{-}F}$ = 23.1 Hz), 98.8, 57.6, 50.8, 21.2, 14.8 (d, $J_{C\text{-}F}$ = 3.6 Hz). ^{19}F-NMR (376 MHz, $CDCl_3$): δ = -108.82. HR-MS (ESI) m/z calcd for $C_{18}H_{16}Cl_3FO$ [M+H$^+$] 373.0324, found 373.0323.

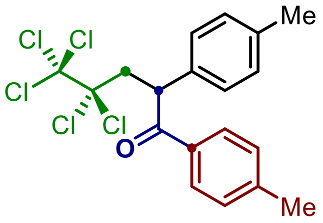

4,4,5,5,5-Pentachloro-1,2-di-p-tolylpentan-1-one (68)

The general procedure **TP4 (TP5)** was followed using **1u** (0.4 mmol), **2a** (0.2 mmol) and **3a** (0.6 mmol) for 2 h. Purification by column chromatography (petroleum ether/EtOAc 20:1) yielded **68** [43.9 mg, 50% (40%)] as a colourless oil. ^{1}H-NMR (400 MHz, $CDCl_3$): δ = 7.95 (d, J = 8.0 Hz, 2H), 7.23 (t, J = 7.8 Hz, 4H), 7.11 (d, J = 7.9 Hz, 2H), 5.24 (dd, J = 8.2, 2.0 Hz, 1H), 4.26 (dd, J = 14.5, 8.1 Hz, 1H), 2.81 (dd, J = 14.5, 2.0 Hz, 1H), 2.36 (s, 3H), 2.27 (s, 3H). ^{13}C-NMR (100 MHz, $CDCl_3$): δ = 197.3, 144.2, 137.5, 135.8, 133.7, 130.2, 129.5, 129.2, 128.1, 104.8, 99.7, 50.4, 45.3, 21.8, 21.2. HR-MS (ESI) m/z calcd for $C_{19}H_{17}Cl_5O$ [M+H$^+$] 436.9795, found 436.9797.

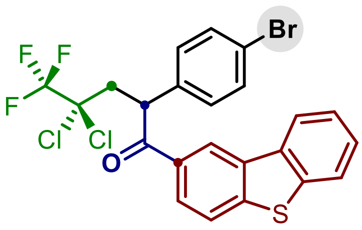

2-(4-Bromophenyl)-4,4-dichloro-1-(dibenzo[b,d]thiophen-2-yl)-5,5,5-trifluoropentan-1-one (69)

The general procedure **TP5** was followed using **1v** (0.4 mmol), **2n** (0.2 mmol) and **3u** (0.6 mmol) for 2 h. Purification by column chromatography (petroleum ether/EtOAc 20:1) yielded **69** (54.6 mg, 50%) as a colourless oil. ^{1}H-NMR (400 MHz, $CDCl_3$): δ = 8.79 (d, J = 1.7 Hz, 1H), 8.28 – 8.16 (m, 1H), 8.07 (dd, J = 8.4, 1.8 Hz, 1H), 7.94 – 7.80 (m, 2H), 7.59 – 7.41 (m, 4H), 7.34 – 7.26 (m, 2H), 5.29 (dd, J = 7.8, 2.7 Hz, 1H), 3.92 (dd, J = 14.8, 7.7 Hz, 1H), 2.61 (dd, J = 14.9, 2.7 Hz, 1H). ^{13}C-NMR (100 MHz, $CDCl_3$): δ = 196.3, 145.2, 139.7, 137.3, 135.9, 135.0, 132.7, 132.0, 129.8, 127.7, 126.5, 125.0, 123.0, 122.4, 122.1, 121.9, 84.8, 48.7, 43.5. ^{19}F-NMR (376 MHz, $CDCl_3$): δ = -79.24. HR-MS (ESI) m/z calcd for $C_{23}H_{14}BrCl_2F_3OS$ [M+H$^+$] 544.9351, found 544.9350.

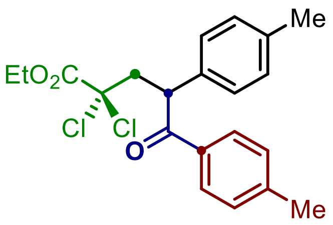

Ethyl 2,2-dichloro-5-oxo-4,5-di-p-tolylpentanoate (70)

The general procedure **TP5** was followed using **1w** (0.4 mmol), **2a** (0.2 mmol) and **3a** (0.6 mmol) for 2 h. Purification by column chromatography (petroleum ether/EtOAc 80:1) yielded **70** (43.3 mg, 55%) as a yellow oil. ^{1}H NMR (400 MHz, $CDCl_3$) δ = 7.88 (d, J = 8.2 Hz, 2H), 7.19 (d, J = 6.7 Hz, 4H), 7.08 (d, J = 7.9 Hz, 2H), 5.02 (dd, J = 7.1, 4.1 Hz, 1H), 4.15 (dd, J = 10.7, 7.1 Hz, 1H), 4.01 (dd, J = 10.7, 7.1 Hz, 1H), 3.74 (dd, J = 14.9, 7.1 Hz, 1H), 2.88 (dd, J = 14.9, 4.1 Hz, 1H), 2.35 (s, 3H), 2.26 (s, 3H), 1.23 (t, J = 7.1 Hz, 3H).^{13}C NMR (100 MHz, $CDCl_3$) δ = 197.3, 165.5, 143.9, 137.3, 135.2, 133.6, 129.8, 129.3, 129.0, 128.3, 84.4, 77.4, 77.1, 76.7, 63.9, 49.7, 48.0, 21.6, 21.0, 13.7. HR-MS (ESI) m/z calcd for $C_{21}H_{22}Cl_2O_3$ [M+Na$^+$] 415.0838, found 415.0835.

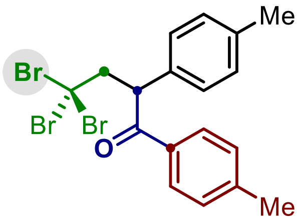

4,4,4-Tribromo-1,2-di-p-tolylbutan-1-one (71)

The general procedure **TP4 (TP5)** was followed using **1x** (0.4 mmol), **2a** (0.2 mmol) and **3a** (0.6 mmol) for 2 h. Purification by column chromatography (petroleum ether/EtOAc 20:1) yielded **71** [42.1 mg, 43% (46%)] as a colourless oil. ^{1}H-NMR (400 MHz, $CDCl_3$): δ = 7.95 (d, J = 7.9 Hz, 2H), 7.26 – 7.20 (m, 4H), 7.11 (d, J = 7.8 Hz, 2H), 4.99 (dd, J = 7.5, 2.0 Hz, 1H), 4.65 (dd, J = 15.2, 7.5 Hz, 1H), 3.36 (dd, J = 15.1, 2.1 Hz, 1H), 2.36 (s, 3H), 2.28 (s, 3H). ^{13}C-NMR (100 MHz, $CDCl_3$): δ = 197.0, 144.2, 137.5, 135.2, 133.9, 130.1, 129.5, 129.2, 128.2, 61.9, 53.4, 39.9, 21.8, 21.2. HR-MS (ESI) m/z calcd for $C_{18}H_{17}Br_3O$ [M+H$^+$] 486.8902, found 486.8900.

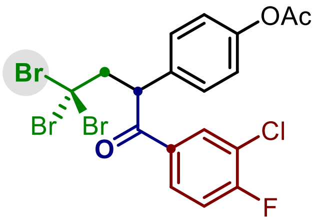

4-[4,4,4-Tribromo-1-(3-chloro-4-fluorophenyl)-1-oxobutan-2-yl]phenyl acetate (72)

The general procedure **TP5** was followed using **1x** (0.4 mmol), **2k** (0.2 mmol) and **3p** (0.6 mmol) for 2 h. Purification by column chromatography (petroleum ether/EtOAc 10:1) yielded **72** (43.5 mg, 38%) as a colourless oil. ^{1}H-NMR (400 MHz, $CDCl_3$): δ = 8.08 (dd, J = 7.0, 2.2 Hz, 1H), 7.97 – 7.88 (m, 1H), 7.40 – 7.32 (m, 2H), 7.20 (t, J = 8.5 Hz, 1H), 7.10 – 7.04 (m, 2H), 4.94 (dd, J = 7.7, 1.9 Hz, 1H), 4.61 (dd, J = 15.2, 7.7 Hz, 1H), 3.38 (dd, J = 15.2, 1.9 Hz, 1H), 2.28 (s, 3H). ^{13}C-NMR (100 MHz, $CDCl_3$): δ = 194.8, 169.4, 161.3 (d, $J_{C\text{-}F}$ = 258.2 Hz), 150.5, 134.5, 133.4 (d, $J_{C\text{-}F}$ = 3.6 Hz), 132.0, 129.4, 129.3, 122.9, 122.4 (d, $J_{C\text{-}F}$ = 18.3 Hz), 117.1 (d, $J_{C\text{-}F}$ = 21.8 Hz), 61.7, 53.2, 39.0, 21.3. ^{19}F-NMR (376 MHz, $CDCl_3$): δ = -106.65. HR-MS (ESI) m/z calcd for $C_{18}H_{13}Br_3ClFO_3$ [M+H$^+$] 568.8160, found 568.8164.

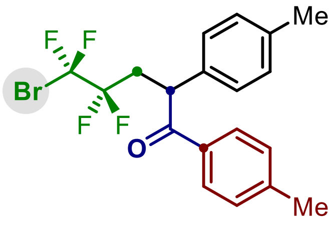

5-Bromo-4,4,5,5-tetrafluoro-1,2-di-p-tolylpentan-1-one (73)

The general procedure **TP4 (TP5)** was followed using **1y** (0.4 mmol), **2a** (0.2 mmol) and **3a** (0.6 mmol) for 2 h. Purification by column chromatography (petroleum ether/EtOAc 40:1) yielded **73** [54.2 mg, 65% (61%)] as a colourless oil. ^{1}H-NMR (400 MHz, $CDCl_3$): δ = 7.92 – 7.84 (m, 2H), 7.24 – 7.16 (m, 4H), 7.11 (d, J = 7.9 Hz, 2H), 4.98 (dd, J = 8.3, 4.4 Hz, 1H), 3.51 – 3.32 (m, 1H), 2.52 – 2.39 (m, 1H), 2.36 (s, 3H), 2.28 (s, 3H). ^{13}C-NMR (100 MHz, $CDCl_3$): δ = 196.6, 144.3, 137.6, 135.1, 133.3, 130.1, 129.5, 129.1, 128.0, 45.6, 34.1 (t, $J_{C\text{-}F}$ = 21.1 Hz), 21.8, 21.2. ^{19}F-NMR (376 MHz, $CDCl_3$): δ = -63.97 – -68.29 (m), -108.78 – -110.01 (m), -110.30 – -111.89 (m). HR-MS (ESI) m/z calcd for $C_{19}H_{17}BrF_4O$ [M+Na$^+$] 439.0291, found 439.0293.

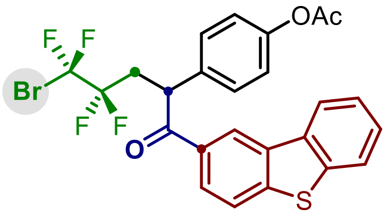

4-[5-Bromo-1-(dibenzo[b,d]thiophen-2-yl)-4,4,5,5-tetrafluoro-1-oxopentan-2-yl]phenyl acetate (74)

The general procedure **TP5** was followed using **1y** (0.4 mmol), **2k** (0.2 mmol) and **3u** (0.6 mmol) for 2 h. Purification by column chromatography (petroleum ether/EtOAc 20:1) yielded **74** (78.6 mg, 71%) as a colourless oil. ^{1}H-NMR (400 MHz, $CDCl_3$): δ = 8.77 (d, J = 1.7 Hz, 1H), 8.22 – 8.16 (m, 1H), 8.09 – 8.00 (m, 1H), 7.91 – 7.79 (m, 2H), 7.53 – 7.40 (m, 4H), 7.15 – 7.01 (m, 2H), 5.19 (dd, J = 8.6, 4.1 Hz, 1H), 3.64 – 3.42 (m, 1H), 2.65 – 2.44 (m, 1H), 2.25 (s, 3H). ^{13}C-NMR (100 MHz, $CDCl_3$): δ = 196.4, 169.3, 150.4, 145.2, 139.8, 135.9, 135.4, 135.1, 132.1, 129.2, 127.7, 126.6, 125.1, 123.0, 123.0, 122.7, 122.4, 122.0, 45.6, 34.3 (t, $J_{C\text{-}F}$ = 21.3 Hz), 21.2. ^{19}F-NMR (376 MHz, $CDCl_3$): δ = -56.66 – -72.13 (m), -107.26 – -109.88 (m), -110.12 – -112.73 (m). HR-MS (ESI) m/z calcd for $C_{25}H_{17}BrF_4O_3S$ [M+H$^+$] 553.0091, found 553.0095.

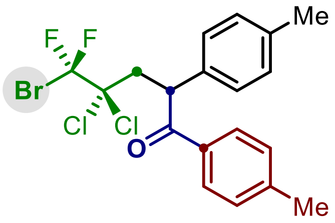

5-Bromo-4,4-dichloro-5,5-difluoro-1,2-di-p-tolylpentan-1-one (75)

The general procedure **TP4 (TP5)** was followed using **1z** (0.4 mmol), **2a** (0.2 mmol) and **3a** (0.6 mmol) for 2 h. Purification by column chromatography (petroleum ether/EtOAc 40:1) yielded **75** [27.9 mg, 31% (39%)] as a colourless oil. ^{1}H-NMR (400 MHz, $CDCl_3$): δ = 7.93 (d, J = 8.2 Hz, 2H), 7.22 (d, J = 8.0 Hz, 4H), 7.11 (d, J = 7.9 Hz, 2H), 5.18 (dd, J = 8.0, 2.3 Hz, 1H), 3.97 (dd, J = 14.7, 8.0 Hz, 1H), 2.60 (dd, J = 14.7, 2.3 Hz, 1H), 2.37 (s, 3H), 2.28 (s, 3H). ^{13}C-NMR (100 MHz, $CDCl_3$): δ = 197.0, 144.3, 137.6, 135.7, 133.6, 130.2, 129.5, 129.2, 128.1, 122.1 (t, J_{C-F} = 313.0 Hz), 91.6 (t, J_{C-F} = 27.2 Hz), 49.1, 44.6, 21.8, 21.2. ^{19}F-NMR (376 MHz, $CDCl_3$): δ = -56.31. HR-MS (ESI) m/z calcd for $C_{19}H_{17}BrCl_2F_2O$ [M+H$^+$] 448.9881, found 448.9884.

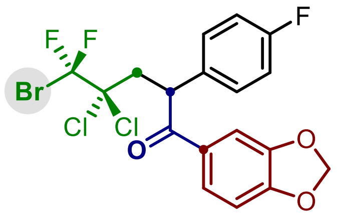

1-(Benzo[d][1,3]dioxol-5-yl)-5-bromo-4,4-dichloro-5,5-difluoro-2-(4-fluorophenyl) pentan-1-one (76)

The general procedure **TP5** was followed using **1z** (0.4 mmol), **2ad** (0.2 mmol) and **3l** (0.6 mmol) for 2 h. Purification by column chromatography (petroleum ether/EtOAc 20:1) yielded **76** (46.7 mg, 48%) as a colourless oil. ^{1}H-NMR (400 MHz, $CDCl_3$): δ = 7.68 – 7.62 (m, 1H), 7.46 (d, J = 1.8 Hz, 1H), 7.36 – 7.27 (m, 2H), 7.04 – 6.98 (m, 2H), 6.83 (d, J = 8.2 Hz, 1H), 6.02 (s, 2H), 5.12 (dd, J = 7.9, 2.5 Hz, 1H), 3.91 (dd, J = 14.7, 7.9 Hz, 1H), 2.59 (dd, J = 14.7, 2.5 Hz, 1H). ^{13}C-NMR (100 MHz, $CDCl_3$): δ = 195.4, 162.3 (d, J_{C-F} = 247.3 Hz), 152.3, 148.5, 134.4 (d, J_{C-F} = 3.3 Hz), 130.6, 129.8 (d, J_{C-F} = 8.1 Hz), 125.3, 121.9 (t, J_{C-F} = 313.4 Hz), 116.5 (d, J_{C-F} = 21.6 Hz), 108.8, 108.2, 102.1, 91.4 (t, J_{C-F} = 27.3 Hz), 48.5, 44.7. ^{19}F-NMR (376 MHz, $CDCl_3$): δ = -56.44, -114.22. HR-MS (ESI) m/z calcd for $C_{18}H_{12}BrCl_2F_3O_3$ [M+H$^+$] 482.9372, found 482.9370.

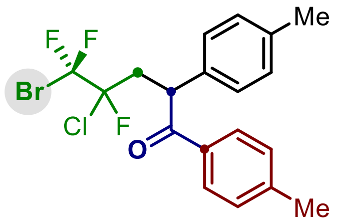

5-Bromo-4-chloro-4,5,5-trifluoro-1,2-di-p-tolylpentan-1-one (77)

The general procedure **TP4 (TP5)** was followed using **1aa** (0.4 mmol), **2a** (0.2 mmol) and **3a** (0.6 mmol) for 2 h. Purification by column chromatography (petroleum ether/EtOAc 40:1) yielded **77** [65.1mg, 75% (80%), dr = 1:1] as a colourless oil. ^{1}H-NMR (400 MHz, $CDCl_3$): δ = 7.92 (d, J = 8.0 Hz, 2H), 7.25 – 7.19 (m, 4H), 7.15 – 7.08 (m, 2H), 5.20 – 5.05 (m, 1H), 3.94 – 3.82 (m, 0.5H), 3.62 – 3.42 (m, 0.5H), 2.81 – 2.69 (m, 0.5H), 2.47 – 2.33 (m, 3.5H), 2.28 (d, J = 3.5 Hz, 3H). ^{13}C-NMR (100 MHz, $CDCl_3$): δ = 196.9, 196.6, 144.3, 137.6, 137.5, 135.3, 133.5, 133.4, 130.2, 130.1, 129.5, 129.2, 129.1, 128.2, 128.0, 47.7, 47.4, 40.2 (d, J_{C-F} = 19.4 Hz), 39.6 (d, J_{C-F} = 20.0 Hz), 21.8, 21.2. ^{19}F-NMR (376 MHz, $CDCl_3$): δ = -54.67 – -64.75 (m), -107.98 – -121.40 (m). HR-MS (ESI) m/z calcd for $C_{19}H_{17}BrClF_3O$ [M+Na^{+}] 454.9996, found 454.9997.

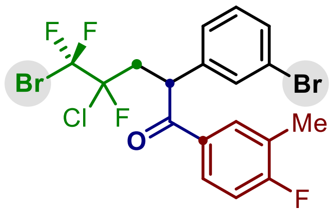

5-Bromo-2-(3-bromophenyl)-4-chloro-4,5,5-trifluoro-1-(4-fluoro-3-methylphenyl)pentan-1-one (78)

The general procedure **TP5** was followed using **1aa** (0.4 mmol), **2o** (0.2 mmol) and **3n** (0.6 mmol) for 2 h. Purification by column chromatography (petroleum ether/EtOAc 40:1) yielded **78** (63.0 mg, 61%, dr = 1:1) as a colourless oil. ^{1}H-NMR (400 MHz, $CDCl_3$): δ = 7.92 – 7.73 (m, 2H), 7.53 – 7.44 (m, 1H), 7.43 – 7.34 (m, 1H), 7.29 – 7.24 (m, 1H), 7.23 – 7.15 (m, 1H), 7.09 – 6.98 (m, 1H), 5.18 – 4.99 (m, 1H), 3.91 – 3.76 (m, 0.5H), 3.58 – 3.39 (m, 0.5H), 2.82 – 2.66 (m, 0.5H), 2.50 – 2.24 (m, 3.5H). ^{13}C-NMR (100 MHz, $CDCl_3$): δ = 195.4, 195.2, 164.8 (dd, J_{C-F} = 255.0, 4.1 Hz), 140.2, 140.1, 133.0, 132.9, 132.8, 131.8 (d, J_{C-F} = 3.5 Hz), 131.7 (d, J_{C-F} = 3.4 Hz), 131.3, 131.2, 131.2, 131.1, 131.0, 129.0, 128.9, 128.8, 126.9, 126.8, 126.1, 126.0, 123.5, 123.5, 119.8 (d, J_{C-F} = 6.7 Hz), 119.5, 115.8, 115.5, 112.4 (d, J_{C-F} = 11.8 Hz), 109.9 (d, J_{C-F} = 9.9 Hz), 47.6, 47.3, 40.3 (d, J_{C-F} = 19.5 Hz), 39.7 (d, J_{C-F} = 20.1 Hz), 14.8 , 14.8. ^{19}F-NMR (376 MHz, $CDCl_3$): δ = -56.10 – -64.26 (m), -107.88 (d, J = 19.1 Hz), -112.65 – -123.88 (m). HR-MS (ESI) m/z calcd for $C_{18}H_{13}Br_2ClF_4O$ [M+H^{+}] 514.9031, found 514.9032.

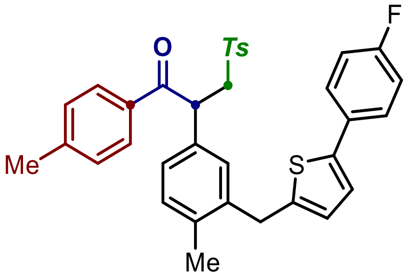

2-{3-{[5-(4-Fluorophenyl)thiophen-2-yl]methyl}-4-methylphenyl}-1-(p-tolyl)-3-tosylpropan-1-one (79)

The general procedure **TP5** was followed using **1a** (0.4 mmol), **2r** (0.2 mmol) and **3a** (0.6 mmol) for 2 h. Purification by column chromatography (petroleum ether/EtOAc 4:1) yielded **79** (90.9 mg, 78%) as a white powder. ^{1}H NMR (400 MHz, $CDCl_3$) δ = 7.78 (d, J = 8.2 Hz, 2H), 7.68 (d, J = 8.2 Hz, 2H), 7.48 – 7.40 (m, 2H), 7.20 (d, J = 8.0 Hz, 2H), 7.17 – 7.09 (m, 3H), 7.04 – 6.98 (m, 5H), 6.56 (d, J = 3.6 Hz, 1H), 5.20 (dd, J = 8.4, 4.0 Hz, 1H), 4.38 (dd, J = 14.2, 8.5 Hz, 1H), 4.01 (s, 2H), 3.43 (dd, J = 14.2, 4.0 Hz, 1H), 2.37 (s, 3H), 2.31 (s, 3H), 2.20 (s, 3H). ^{13}C NMR (100 MHz, $CDCl_3$) δ = 195.3, 162.1 (d, $J_{C\text{-}F}$ = 246.6 Hz), 144.5, 144.2, 142.9, 142.4, 141.5, 139.2, 136.5, 136.1, 134.6, 132.9, 131.4, 130.8 (d, $J_{C\text{-}F}$ = 3.4 Hz), 129.7, 129.5, 129.3 (d, $J_{C\text{-}F}$ = 7.9 Hz), 129.2, 129.0, 128.1, 127.1, 127.0, 126.4, 125.9, 122.6, 115.7 (d, $J_{C\text{-}F}$ = 21.6 Hz), 109.3, 108.0, 59.1, 47.1, 33.9, 21.6, 21.6, 19.0. ^{19}F NMR (376 MHz, $CDCl_3$) δ = -115.04. HR-MS (ESI) m/z calcd for $C_{35}H_{31}FO_3S_2$ [M+Na$^+$] 605.1591, found 605.1593.

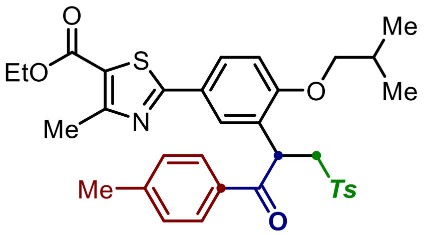

Ethyl 2-{4-isobutoxy-3-[1-oxo-1-(p-tolyl)-3-tosylpropan-2-yl]phenyl}-4-methylthiazole-5-carboxylate (80)

The general procedure **TP5** was followed using **1a** (0.4 mmol), **2s** (0.2 mmol) and **3a** (0.6 mmol) for 2 h. Purification by column chromatography (petroleum ether/EtOAc 5:1) yielded **80** (55.8 mg, 45%) as a white powder. ^{1}H NMR (400 MHz, $CDCl_3$) δ = 7.84 (d, J = 8.2 Hz, 2H), 7.75 (dd, J = 8.6, 2.2 Hz, 1H), 7.68 (d, J = 8.2 Hz, 2H), 7.62 (d, J = 2.2 Hz, 1H), 7.21 (d, J = 8.0 Hz, 2H), 7.15 (d, J = 8.1 Hz, 2H), 6.84 (d, J = 8.7 Hz, 2H), 5.71 (dd, J = 8.0, 4.6 Hz, 1H), 4.45 – 4.22 (m, 3H), 3.94 – 3.76 (m, 2H), 3.44 (dd, J = 14.2, 4.5 Hz, 1H), 2.72 (s, 3H), 2.37 (s, 3H), 2.34 (s, 3H), 2.29 – 2.15 (m, 1H), 1.37 (t, J = 7.1 Hz, 3H), 1.18 (d, J = 2.4 Hz, 3H), 1.16 (d, J

= 2.4 Hz, 3H). ^{13}C NMR (100 MHz, $CDCl_3$) δ = 195.6, 168.9, 162.3, 161.0, 157.5, 144.4, 144.3, 136.7, 132.9, 129.6, 129.1, 128.8, 128.0, 127.8, 127.5, 126.1, 125.8, 121.0, 112.0, 75.2, 61.1, 58.1, 40.2, 28.4, 21.6, 21.5, 19.5, 19.3, 17.4, 14.3. HR-MS (ESI) m/z calcd for $C_{34}H_{37}NO_6S_2$ [M+Na$^+$] 642.1955, found 642.1958.

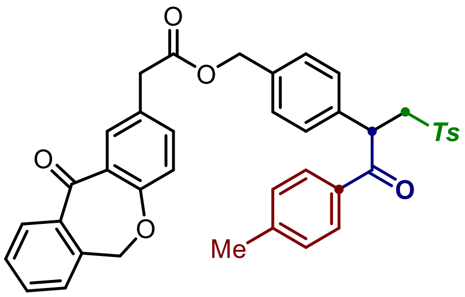

4-[1-Oxo-1-(p-tolyl)-3-tosylpropan-2-yl]benzyl 2-(11-oxo-6,11-dihydrodibenzo[b,e]oxepi-2-yl)acetate (81)

The general procedure **TP5** was followed using **1a** (0.4 mmol), **2t** (0.2 mmol) and **3a** (0.6 mmol) for 2 h. Purification by column chromatography (petroleum ether/EtOAc 5:1) yielded **81** (65.8 mg, 50%) as a white powder. ^{1}H NMR (400 MHz, $CDCl_3$) δ = 8.10 (d, *J* = 2.3 Hz, 1H), 7.88 (dd, *J* = 7.7, 1.0 Hz, 1H), 7.79 (d, *J* = 8.2 Hz, 2H), 7.68 (d, *J* = 8.2 Hz, 2H), 7.55 (td, *J* = 7.4, 1.3 Hz, 1H), 7.47 (td, *J* = 7.5, 1.1 Hz, 1H), 7.40 – 7.33 (m, 2H), 7.24 – 7.14 (m, 8H), 6.99 (d, *J* = 8.4 Hz, 1H), 5.26 (dd, *J* = 8.6, 3.9 Hz, 1H), 5.17 (s, 2H), 5.03 (s, 2H), 4.37 (dd, *J* = 14.1, 8.6 Hz, 1H), 3.65 (s, 2H), 3.40 (dd, *J* = 14.1, 3.9 Hz, 1H), 2.38 (s, 3H), 2.35 (s, 3H). ^{13}C NMR (100 MHz, $CDCl_3$) δ = 195.2, 190.7, 171.1, 160.5, 144.6, 144.5, 140.4, 136.8, 136.4, 136.3, 135.5, 135.5, 132.8, 132.8, 132.4, 129.8, 129.4, 129.3, 129.3, 129.0, 128.3, 128.0, 127.8, 127.5, 125.1, 121.1, 73.6, 66.0, 59.2, 47.0, 40.1, 21.6, 21.6. HR-MS (ESI) m/z calcd for $C_{40}H_{34}O_7S$ [M+Na$^+$] 681.1917, found 681.1919.

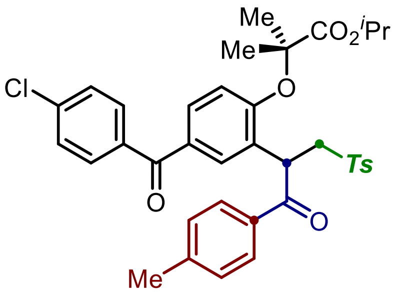

Isopropyl 2-{4-(4-chlorobenzoyl)-2-[1-oxo-1-(p-tolyl)-3-tosylpropan-2-yl]phenoxy}-2-methylpropanoate (82)

The general procedure **TP5** was followed using **1a** (0.4 mmol), **2u** (0.2 mmol) and **3a** (0.6 mmol) for 2 h. Purification by column chromatography (petroleum ether/EtOAc 5:1) yielded

82 (60.8 mg, 46%) as a white powder. ^{1}H NMR (400 MHz, $CDCl_3$) δ = 7.83 (d, *J* = 8.2 Hz, 2H), 7.72 (d, *J* = 8.2 Hz, 2H), 7.59 (dd, *J* = 8.7, 2.2 Hz, 1H), 7.57 – 7.52 (m, 3H), 7.38 (dd, *J* = 8.7, 2.0 Hz, 2H), 7.22 (dd, *J* = 27.7, 8.1 Hz, 4H), 6.65 (d, *J* = 8.7 Hz, 1H), 5.64 (dd, *J* = 6.9, 5.3 Hz, 1H), 5.07 (dt, *J* = 12.5, 6.3 Hz, 1H), 4.27 (dd, *J* = 14.2, 7.1 Hz, 1H), 3.43 (dd, *J* = 14.2, 5.2 Hz, 1H), 2.40 (s, 3H), 2.36 (s, 3H), 1.79 (s, 3H), 1.70 (s, 3H), 1.16 (d, *J* = 6.2 Hz, 3H), 1.12 (d, *J* = 6.3 Hz, 3H). ^{13}C NMR (100 MHz, $CDCl_3$) δ = 195.5, 193.5, 172.5, 156.0, 144.6, 144.3, 138.6, 136.8, 135.8, 132.8, 131.9, 131.2, 131.0, 130.0, 129.8, 129.2, 128.9, 128.5, 128.0, 126.7, 114.3, 80.2, 69.4, 57.6, 41.3, 26.3, 24.1, 21.6, 21.6, 21.5, 21.4. HR-MS (ESI) m/z calcd for $C_{37}H_{37}ClO_7S$ [M+Na$^+$] 683.1841, found 683.1839.

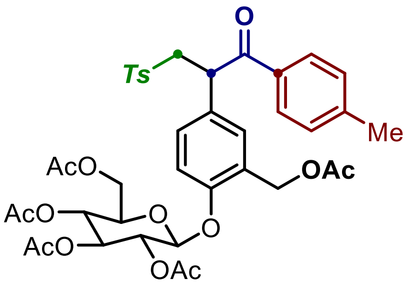

(2R,3R,4S,5R,6S)-2-(Acetoxymethyl)-6-{2-(acetoxymethyl)-4-[1-oxo-1-(p-tolyl)-3-tosylpropan-2-yl]phenoxy}tetrahydro-2H-pyran-3,4,5-triyl triacetate (83)

The general procedure **TP4** was followed using **1a** (0.4 mmol), **2v** (0.2 mmol) and **3a** (0.6 mmol) for 2 h. Purification by column chromatography (petroleum ether/EtOAc 1:1) yielded **83** (98.8 mg, 62%, dr = 1:1) as a white powder. ^{1}H NMR (400 MHz, $CDCl_3$) δ = 7.79 (d, *J* = 8.2 Hz, 2H), 7.67 (dd, *J* = 8.0, 6.1 Hz, 2H), 7.21 (dd, *J* = 12.8, 8.7 Hz, 5H), 7.17 – 7.09 (m, 1H), 6.93 (dd, *J* = 10.5, 8.7 Hz, 1H), 5.25 (dd, *J* = 9.6, 5.2 Hz, 3H), 5.18 – 5.10 (m, 1H), 5.02 – 4.87 (m, 3H), 4.37 – 4.27 (m, 1H), 4.27 – 4.20 (m, 1H), 4.19 – 4.13 (m, 1H), 3.86 – 3.79 (m, 1H), 3.45 – 3.35 (m, 1H), 2.40 (s, 3H), 2.37 (s, 3H), 2.10 – 2.02 (m, 15H). ^{13}C NMR (100 MHz, $CDCl_3$) δ = 195.2, 170.5, 170.4, 170.1, 169.3, 169.2, 153.8, 144.5, 136.4, 132.7, 131.5, 129.7, 129.3, 129.1, 129.0, 129.0, 128.0, 127.1, 116.0, 98.8, 77.3, 72.4, 72.0, 70.8, 68.1, 61.8, 60.6, 60.3, 59.1, 46.5, 46.4, 21.6, 21.5, 20.9, 20.6, 20.5, 14.2. HR-MS (ESI) m/z calcd for $C_{40}H_{44}O_{15}S$ [M+Na$^+$] 819.2293, found 819.2296.

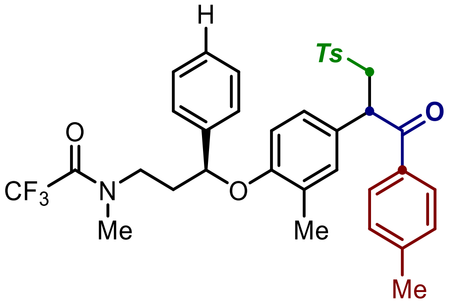

2,2,2-Trifluoro-N-methyl-N-{(3S)-3-{2-methyl-4-[1-oxo-1-(p-tolyl)-3-tosylpropan-2-yl]phenoxy}-3-phenylpropyl}acetamide (84)

The general procedure **TP5** was followed using **1a** (0.4 mmol), **2w** (0.2 mmol) and **3a** (0.6 mmol) for 2 h. Purification by column chromatography (petroleum ether/EtOAc 2:1) yielded **84** (62.6 mg, 48%, dr = 1:1) as a white powder. ^{1}H NMR (400 MHz, $CDCl_3$) δ = 7.76 (dd, *J* = 8.2, 3.9 Hz, 2H), 7.63 (dd, *J* = 13.4, 4.9 Hz, 2H), 7.37 – 7.26 (m, 4H), 7.24 – 7.23 (m, 1H), 7.21 – 7.07 (m, 4H), 6.99 – 6.89 (m, 1H), 6.81 (t, *J* = 10.1 Hz, 1H), 6.49 – 6.35 (m, 1H), 5.21 – 4.97 (m, 2H), 4.31 (dd, *J* = 14.1, 8.9 Hz, 1H), 3.70 – 3.46 (m, 2H), 3.46 – 3.24 (m, 1H), 3.05 (d, *J* = 1.2 Hz, 2H), 2.97 (s, 1H), 2.37 (s, 3H), 2.34 (d, *J* = 1.9 Hz, 3H), 2.26 – 2.08 (m, 5H). ^{13}C NMR (100 MHz, $CDCl_3$) δ = 195.4, 155.2, 144.4, 144.2, 140.7, 140.2, 136.5, 133.0, 130.2, 130.1, 129.7, 129.6, 129.2, 129.0, 128.9, 128.8, 128.4, 128.0, 127.9, 126.6, 126.4, 125.5, 125.4, 113.0, 112.8, 77.5, 77.3, 59.4, 59.3, 46.9, 46.4, 37.4, 35.5, 34.6, 21.6, 21.5, 16.4, 16.3. ^{19}F NMR (376 MHz, $CDCl_3$) δ = -68.99 (d, *J* = 2.0 Hz), -69.86 (d, *J* = 2.6 Hz). HR-MS (ESI) m/z calcd for $C_{36}H_{36}F_3NO_5S$ [M+Na$^+$] 674.2158, found 674.2160.

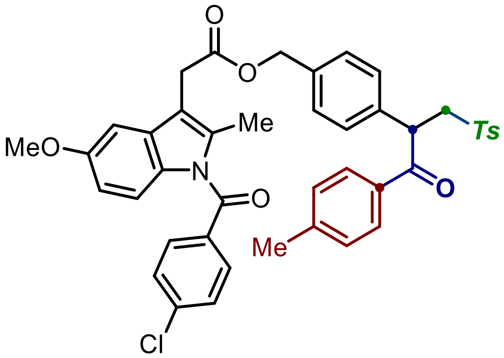

4-[1-Oxo-1-(p-tolyl)-3-tosylpropan-2-yl]benzyl 2-[1-(4-chlorobenzoyl)-5-methoxy-2-methyl-1H-indol-3-yl]acetate (85)

The general procedure **TP5** was followed using **1a** (0.4 mmol), **2x** (0.2 mmol) and **3a** (0.6 mmol) for 2 h. Purification by column chromatography (petroleum ether/EtOAc 6:1) yielded **85** (67.3 mg, 45%) as a white powder. ^{1}H NMR (400 MHz, $CDCl_3$) δ = 7.79 (d, *J* = 8.2 Hz, 2H), 7.66 (dd, *J* = 18.5, 8.4 Hz, 4H), 7.46 (d, *J* = 8.5 Hz, 2H), 7.25 – 7.12 (m, 8H), 6.87 (dd, *J* = 15.7, 5.7

Hz, 2H), 6.65 (dd, J = 9.0, 2.5 Hz, 1H), 5.29 (s, 2H), 5.26 (dd, J = 8.6, 3.9 Hz, 1H), 5.03 (s, 2H), 4.36 (dd, J = 14.1, 8.6 Hz, 1H), 3.72 (s, 3H), 3.39 (dd, J = 14.1, 3.9 Hz, 1H), 2.39 (s, 3H), 2.36 (s, 3H), 2.35 (s, 3H). ^{13}C NMR (100 MHz, $CDCl_3$) δ = 195.2, 170.5, 168.2, 156.0, 144.7, 144.5, 139.3, 136.8, 136.4, 136.0, 135.5, 133.8, 132.8, 131.1, 130.7, 130.5, 129.8, 129.3, 129.1, 129.0, 128.8, 128.3 128.0, 114.9, 112.3, 111.7, 101.2, 66.0, 59.2, 55.6, 46.9, 30.3, 21.6, 21.6, 13.3. HR-MS (ESI) m/z calcd for $C_{43}H_{38}ClNO_7S$ [M+Na$^+$] 770.1950, found 770.1953.

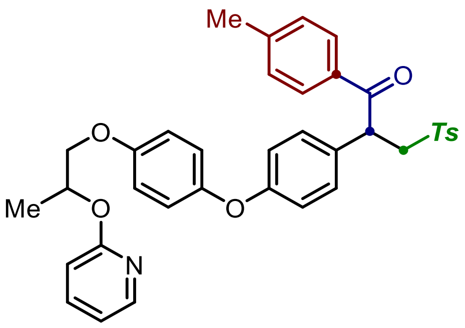

2-{4-{4-[2-(Pyridin-2-yloxy)propoxy]phenoxy}phenyl}-1-(p-tolyl)-3-tosylpropan-1-one (86)

The general procedure **TP5** was followed using **1a** (0.4 mmol), **2y** (0.2 mmol) and **3a** (0.6 mmol) for 2 h. Purification by column chromatography (petroleum ether/EtOAc 4:1) yielded **86** (88.3 mg, 71%, dr > 20:1) as a pale yellow powder. ^{1}H NMR (400 MHz, $CDCl_3$) δ = 8.13 (dd, J = 4.9, 1.5 Hz, 1H), 7.80 (d, J = 8.2 Hz, 2H), 7.68 (d, J = 8.2 Hz, 2H), 7.58 – 7.51 (m, 1H), 7.20 (dd, J = 15.7, 8.1 Hz, 4H), 7.13 (d, J = 8.7 Hz, 2H), 6.92 – 6.82 (m, 5H), 6.74 (dd, J = 15.0, 8.5 Hz, 3H), 5.57 (dd, J = 11.5, 5.3 Hz, 1H), 5.22 (dd, J = 8.5, 4.0 Hz, 1H), 4.35 (dd, J = 14.2, 8.5 Hz, 1H), 4.17 (dd, J = 9.8, 5.3 Hz, 1H), 4.05 (dd, J = 9.9, 4.8 Hz, 1H), 3.42 (dd, J = 14.2, 4.0 Hz, 1H), 2.38 (s, 3H), 2.36 (s, 3H), 1.47 (d, J = 6.4 Hz, 3H). ^{13}C NMR (100 MHz, $CDCl_3$) δ = 195.5, 163.1, 158.3, 155.5, 149.4, 146.7, 144.6, 144.3, 138.7, 136.5, 132.9, 130.2, 129.7, 129.4, 129.3, 129.0, 128.0, 121.0, 117.8, 116.8, 115.8, 111.6, 71.0, 69.2, 59.3, 46.5, 21.6, 21.6, 17.0. HR-MS (ESI) m/z calcd for $C_{37}H_{35}NO_6S$ [M+Na$^+$] 644.2077, found 644.2075.

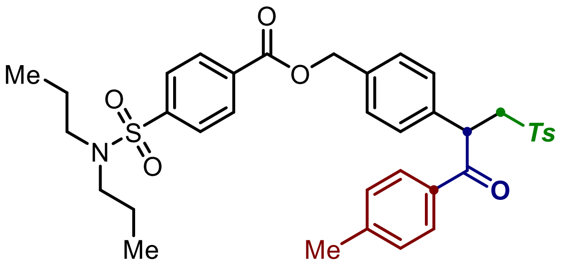

4-[1-Oxo-1-(p-tolyl)-3-tosylpropan-2-yl]benzyl 4-(N,N-dipropylsulfamoyl)benzoate (87)

The general procedure **TP5** was followed using **1a** (0.4 mmol), **2z** (0.2 mmol) and **3a** (0.6 mmol) for 2 h. Purification by column chromatography (petroleum ether/EtOAc 6:1) yielded **87** (79.7 mg, 59%) as a colorless oil. ^{1}H NMR (400 MHz, $CDCl_3$) δ = 8.14 (d, *J* = 8.5 Hz, 2H), 7.83 (dd, *J* = 17.1, 8.4 Hz, 4H), 7.69 (d, *J* = 8.3 Hz, 2H), 7.31 (dd, *J* = 19.7, 8.3 Hz, 4H), 7.21 (dd, *J* = 16.9, 8.0 Hz, 4H), 5.33 – 5.26 (m, 3H), 4.37 (dd, *J* = 14.1, 8.5 Hz, 1H), 3.41 (dd, *J* = 14.1, 4.0 Hz, 1H), 3.12 – 3.00 (m, 4H), 2.39 (s, 3H), 2.36 (s, 3H), 1.53 (dq, *J* = 14.9, 7.4 Hz, 4H), 0.86 (t, *J* = 7.4 Hz, 6H). ^{13}C NMR (100 MHz, $CDCl_3$) δ = 195.2, 164.9, 144.7, 144.5, 144.4, 137.1, 136.4, 135.2, 133.2, 132.8, 130.3, 129.8, 129.3, 129.2, 129.0, 128.5, 128.0, 127.0, 66.6, 59.2, 49.9, 46.9, 21.9, 21.6, 21.6, 11.1. HR-MS (ESI) m/z calcd for $C_{37}H_{41}NO_7S_2$ [M+Na$^+$] 698.2217, found 698.2219.

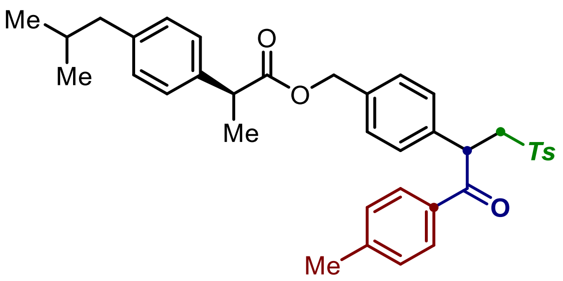

4-[1-Oxo-1-(p-tolyl)-3-tosylpropan-2-yl]benzyl (2S)-2-(4-isobutylphenyl)propanoate (88)

The general procedure **TP5** was followed using **1a** (0.4 mmol), **2aa** (0.2 mmol) and **3a** (0.6 mmol) for 2 h. Purification by column chromatography (petroleum ether/EtOAc 7:1) yielded **88** (77.6 mg, 65% , dr = 1:1) as a colorless oil. ^{1}H NMR (400 MHz, $CDCl_3$) δ = 7.78 (d, *J* = 8.1 Hz, 2H), 7.67 (d, *J* = 8.1 Hz, 2H), 7.24 – 7.12 (m, 8H), 7.06 (dd, *J* = 13.4, 7.2 Hz, 4H), 5.24 (dd, *J* = 8.5, 3.8 Hz, 1H), 5.05 – 4.94 (m, 2H), 4.35 (dd, *J* = 14.1, 8.6 Hz, 1H), 3.70 (q, *J* = 7.1 Hz, 1H), 3.38 (dd, *J* = 14.1, 3.9 Hz, 1H), 2.43 (dd, *J* = 7.1, 2.7 Hz, 2H), 2.38 (s, 3H), 2.36 (s, 3H), 1.91 – 1.75 (m, 1H), 1.47 (d, *J* = 7.1 Hz, 3H), 0.90 (s, 3H), 0.88 (s, 3H). ^{13}C NMR (100 MHz, $CDCl_3$) δ = 195.2, 174.4, 144.6, 144.4, 140.6, 137.4, 136.4, 136.4, 135.8, 132.8, 129.7, 129.3, 129.0, 128.5, 128.2, 128.0, 127.1, 65.5, 59.2, 47., 45.0, 45.0, 30.2, 22.3, 21.6, 21.6, 18.4. HR-MS (ESI) m/z calcd for $C_{37}H_{40}O_5S$ [M+Na$^+$] 619.2489, found 619.2488.

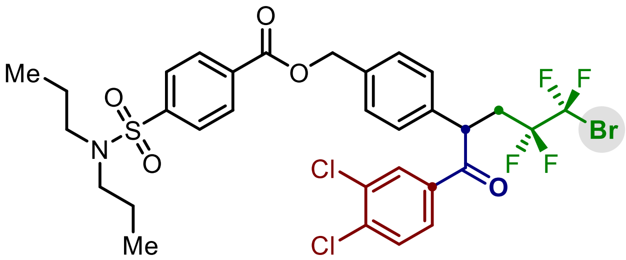

4-[5-Bromo-1-(3,4-dichlorophenyl)-4,4,5,5-tetrafluoro-1-oxopentan-2-yl]benzyl 4-(N,N-

dipropylsulfamoyl)benzoate (89)

The general procedure **TP5** was followed using **1y** (0.4 mmol), **2z** (0.2 mmol) and **3q** (0.6 mmol) for 2 h. Purification by column chromatography (petroleum ether/EtOAc 20:1) yielded **89** (60.4 mg, 40%) as a yellow oil. ^{1}H NMR (400 MHz, $CDCl_3$) δ = 8.15 (d, *J* = 8.5 Hz, 2H), 8.03 (d, *J* = 2.1 Hz, 1H), 7.86 (d, *J* = 8.5 Hz, 2H), 7.77 (dd, *J* = 8.4, 2.1 Hz, 1H), 7.49 (d, *J* = 8.5 Hz, 1H), 7.42 (d, *J* = 7.9 Hz, 2H), 7.32 (d, *J* = 8.2 Hz, 2H), 5.33 (s, 2H), 4.94 (dd, *J* = 8.3, 4.3 Hz, 1H), 3.52 – 3.29 (m, 1H), 3.14 – 3.02 (m, 4H), 2.54 – 2.36 (m, 1H), 1.58 – 1.49 (m, 4H), 0.86 (t, *J* = 7.4 Hz, 6H). ^{13}C NMR (100 MHz, $CDCl_3$) δ = 194.6, 165.1, 144.6, 138.4, 137.3, 135.8, 135.2, 133.7, 133.3, 131.0, 130.9, 130.5, 129.6, 128.5, 127.9, 127.2, 66.7, 50.1, 46.1, 34.1 (t, $J_{C\text{-}F}$ = 21.4 Hz), 22.1, 11.3. ^{19}F NMR (376 MHz, $CDCl_3$) δ = -66.08 (dd, *J* = 23.5, 4.8 Hz), -99.68 – -119.49 (m). HR-MS (ESI) m/z calcd for $C_{31}H_{30}BrCl_2F_4NO_5S$ [M+H$^+$] 754.0414, found 754.0418.

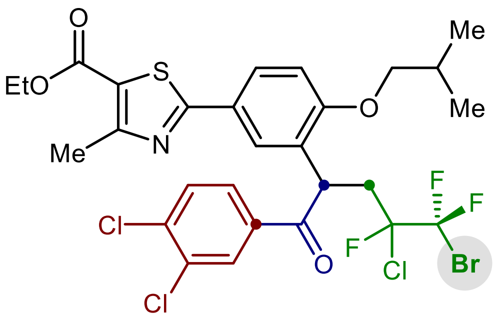

Ethyl 2-{3-[5-bromo-4-chloro-1-(3,4-dichlorophenyl)-4,5,5-trifluoro-1-oxopentan-2-yl]-4-isobutoxyphenyl}-4-methylthiazole-5-carboxylate (90)

The general procedure **TP5** was followed using **1aa** (0.4 mmol), **2s** (0.2 mmol) and **3q** (0.6 mmol) for 2 h. Purification by column chromatography (petroleum ether/EtOAc 20:1) yielded **90** (95.5 mg, 67%) as a yellow oil. ^{1}H NMR (400 MHz, $CDCl_3$) δ = 8.14 (t, *J* = 2.3 Hz, 1H), 7.89 – 7.80 (m, 2H), 7.72 (t, *J* = 2.1 Hz, 1H), 7.46 (dd, *J* = 8.4, 4.7 Hz, 1H), 6.97 (dd, *J* = 8.5, 7.2 Hz, 1H), 5.64 (ddd, *J* = 44.6, 7.8, 3.8 Hz, 1H), 4.33 (q, *J* = 7.1 Hz, 2H), 3.98 – 3.91 (m, 2H), 3.90 – 3.74 (m, 1H), 2.84 - 2.70 (m, 3.5H), 2.46 – 2.30 (m, 1.5H), 1.37 (t, *J* = 7.1 Hz, 3H), 1.22 – 1.14 (m, 6H).^{13}C NMR (100 MHz, $CDCl_3$) δ = 194.8, 168.7, 162.2, 161.1, 157.2, 135.1, 133.2, 130.7 (d, $J_{C\text{-}F}$ = 12.5 Hz), 128.2 (d, $J_{C\text{-}F}$ = 5.2 Hz), 127.7, 127.0 (d, $J_{C\text{-}F}$ = 16.2 Hz), 126.5 (d, $J_{C\text{-}F}$ = 5.7 Hz), 126.2, 112.3 (d, $J_{C\text{-}F}$ = 5.6 Hz), 77.3, 77.0, 76.7, 75.3, 61.2, 40.69 – 37.95 (m), 28.5 (d, $J_{C\text{-}F}$ = 2.7 Hz), 19.4 (dd, $J_{C\text{-}F}$ = 10.0, 4.3 Hz), 17.5, 14.3.^{19}F NMR (376 MHz, $CDCl_3$) δ = -60.87 – -61.48 (m), -116.84 (dt, *J* = 170.9, 11.2 Hz). HR-MS (ESI) m/z calcd for $C_{28}H_{26}BrCl_3F_3NO_4S$ [M+Na$^+$] 735.9676, found 735.9673.

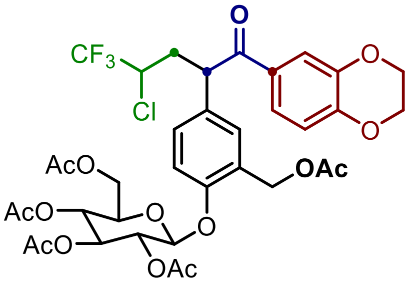

(2R,3R,4S,5R,6S)-2-(Acetoxymethyl)-6-{2-(acetoxymethyl)-4-[4-chloro-1-(2,3-dihydrobenzo[b][1,4]dioxin-6-yl)-5,5,5-trifluoro-1-oxopentan-2-yl]phenoxy}tetrahydro-2H-pyran-3,4,5-triyl triacetate (91)

The general procedure **TP5** was followed using **1s** (0.4 mmol), **2v** (0.2 mmol) and **3m** (0.6 mmol) for 2 h. Purification by column chromatography (petroleum ether/EtOAc 1:1) yielded **91** (67.4 mg, 42%, dr = 1:1) as a yellow oil. ^{1}H NMR (400 MHz, $CDCl_3$) δ = 7.52 – 7.45 (m, 2H), 7.35 – 7.14 (m, 2H), 7.07 – 6.98 (m, 1H), 6.84 (dd, J = 4.9, 4.3 Hz, 1H), 5.29 (d, J = 2.9 Hz, 1H), 5.27 (dd, J = 5.4, 2.9 Hz, 1H), 5.19 – 5.13 (m, 1H), 5.11 – 5.03 (m, 2H), 4.98 (dd, J = 13.2, 4.4 Hz, 1H), 4.89 – 4.75 (m, 1H), 4.30 – 4.14 (m, 2H), 3.94 – 3.66 (m, 2H), 3.01 – 2.28 (m, 1H), 2.73 – 2.00 (m, 16H). ^{13}C NMR (100 MHz, $CDCl_3$) δ = 195.8, 170.4, 170.1, 169.3, 169.2, 169.2, 153.9, 148.3, 143.3, 131.5, 129.3, 129.1, 128.9, 127.3, 127.2, 122.9, 118.3, 117.4, 117.3, 116.3, 98.8, 72.5, 72.0, 70.8, 68.2, 64.6, 64.0, 61.8, 60.7, 60.6, 48.4, 20.9, 20.8, 20.6, 20.5. ^{19}F NMR (376 MHz, $CDCl_3$) δ = -74.41 – -75.00 (m). HR-MS (ESI) m/z calcd for $C_{36}H_{38}ClF_3O_{15}$ [M+Na$^+$] 825.1744, found 824.1740.

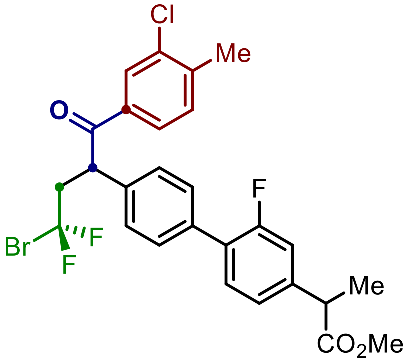

Methyl 2-{4'-[4-bromo-1-(3-chloro-4-methylphenyl)-4,4-difluoro-1-oxobutan-2-yl]-2-fluoro-[1,1'-biphenyl]-4-yl}propanoate (92)

The general procedure **TP5** was followed using **1q** (0.4 mmol), **2ab** (0.2 mmol) and **3o** (0.6 mmol) for 2 h. Purification by column chromatography (petroleum ether/EtOAc 15:1) yielded **92** (48.8 mg, 43%) as a yellow oil. ^{1}H NMR (400 MHz, $CDCl_3$) δ = 7.90 (d, J = 1.2 Hz, 1H), 7.71 (dd, J = 7.9, 1.4 Hz, 1H), 7.40 (d, J = 7.2 Hz, 2H), 7.27 – 7.15 (m, 4H), 7.03 (t, J = 9.3 Hz,

2H), 4.89 (dd, J = 7.9, 4.2 Hz, 1H), 3.66 (dd, J = 14.0, 7.0 Hz, 2H), 3.61 (s, 3H), 2.91 – 2.66 (m, 1H), 2.31 (s, 3H), 1.44 (d, J = 7.2 Hz, 3H). ^{13}C NMR (100 MHz, $CDCl_3$) δ = 195.1, 174.3, 159.6 (d, $J_{C\text{-}F}$ = 248.8 Hz), 142.3, 136.4, 135.0, 131.2, 130.6 (d, $J_{C\text{-}F}$ = 4.0 Hz), 129.9 (d, $J_{C\text{-}F}$ = 3.0 Hz), 129.5, 128.1, 127.0, 123.6 (d, $J_{C\text{-}F}$ = 3.1 Hz), 115.3 (d, $J_{C\text{-}F}$ = 23.5 Hz), 52.2, 48.2, 47.4 (t, $J_{C\text{-}F}$ = 21.2 Hz), 44.9, 20.3, 18.4. ^{19}F NMR (376 MHz, $CDCl_3$) δ = -43.26 (q, J = 156.7 Hz), -117.45. HR-MS (ESI) m/z calcd for $C_{27}H_{23}BrClF_3O_3$ [M+Na$^+$] 589.0363, found 589.0366.

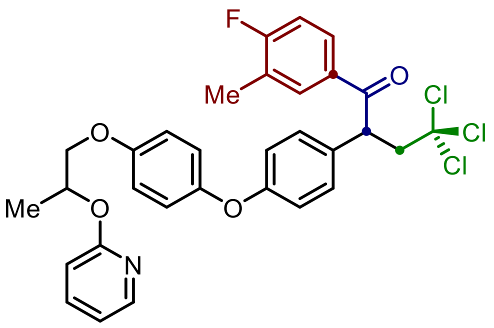

4,4,4-Trichloro-1-(4-fluoro-3-methylphenyl)-2-{4-{4-[2-(pyridin-2-yloxy)propoxy]phenoxy}phenyl}butan-1-one (93)

The general procedure **TP5** was followed using **1t** (0.4 mmol), **2y** (0.2 mmol) and **3n** (0.6 mmol) for 2 h. Purification by column chromatography (petroleum ether/EtOAc 15:1) yielded **93** (57.8 mg, 48%) as a yellow oil. ^{1}H NMR (400 MHz, $CDCl_3$) δ = 8.14 (dd, J = 4.9, 1.4 Hz, 1H), 7.94 – 7.79 (m, 2H), 7.62 – 7.51 (m, 1H), 7.22 (d, J = 8.7 Hz, 2H), 7.03 (t, J = 8.8 Hz, 1H), 6.91 (s, 4H), 6.87 – 6.79 (m, 3H), 6.73 (d, J = 8.3 Hz, 1H), 5.58 (dd, J = 11.5, 5.3 Hz, 1H), 5.04 (dd, J = 7.9, 2.4 Hz, 1H), 4.28 – 4.10 (m, 2H), 4.06 (dd, J = 9.9, 4.8 Hz, 1H), 3.00 (dd, J = 14.9, 2.4 Hz, 1H), 2.29 (s, 3H), 1.47 (d, J = 6.4 Hz, 3H). ^{13}C NMR (100 MHz, $CDCl_3$) δ = 196.0, 164.4 (d, $J_{C\text{-}F}$ = 254.5 Hz), 163.1, 158.3, 155.5, 149.6, 146.7, 138.7, 132.7 (d, $J_{C\text{-}F}$ = 6.6 Hz), 131.5, 129.3, 128.7 (d, $J_{C\text{-}F}$ = 9.4 Hz), 125.6 (d, $J_{C\text{-}F}$ = 17.8 Hz), 121.0, 118.0, 116.7, 115.8, 115.3 (d, $J_{C\text{-}F}$ = 23.0 Hz), 111.6, 98.5, 71.0, 69.2, 57.5, 50.1, 17.0, 14.6 (d, $J_{C\text{-}F}$ = 3.3 Hz). ^{19}F NMR (377 MHz, $CDCl_3$) δ = -108.62. HR-MS (ESI) m/z calcd for $C_{31}H_{27}Cl_3FNO_4$ [M+Na$^+$] 624.0882, found 624.0881.

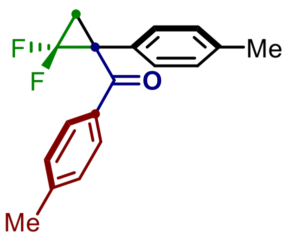

[2,2-Difluoro-1-(p-tolyl)cyclopropyl](p-tolyl)methanone (94)

The general procedure **TP6** was followed using **62** (0.15 mmol), **Cs_2CO_3** (0.45 mmol) at 25℃ for 12 h. Purification by column chromatography (petroleum ether/EtOAc 40:1) yielded **94** (38.4 mg, 90%) as a colourless oil. ^{1}H-NMR (400 MHz, $CDCl_3$): δ = 7.88 (d, J = 8.1 Hz, 2H), 7.37 (d, J = 7.9 Hz, 2H), 7.21 (d, J = 8.0 Hz, 2H), 7.10 (d, J = 8.0 Hz, 2H), 2.56 – 2.48 (m, 1H), 2.36 (s, 3H), 2.27 (s, 3H), 1.86 – 1.75 (m, 1H). ^{13}C-NMR (100 MHz, $CDCl_3$): δ = 191.9, 144.4, 138.0, 132.9, 131.6 (d, J_{C-F} = 1.9 Hz), 129.9, 129.7, 129.4, 129.1 (t, J_{C-F} = 1.9 Hz), 111.5 (dd, J_{C-F} = 296.0, 285.9 Hz), 43.5 (dd, J_{C-F} = 12.9, 8.6 Hz), 22.5 (dd, J_{C-F} = 10.4, 8.9 Hz), 21.8, 21.2. ^{19}F-NMR (376 MHz, $CDCl_3$): δ = -126.38 (d, J = 153.9 Hz), -130.12 (d, J = 153.9 Hz). HR-MS (ESI) m/z calcd for $C_{18}H_{16}F_2O$ [M+Na$^+$] 309.1061, found 309.1062.

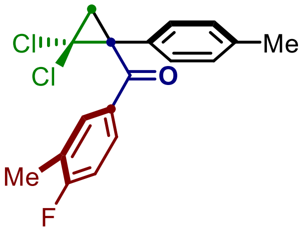

[2,2-Dichloro-1-(p-tolyl)cyclopropyl](4-fluoro-3-methylphenyl)methanone (95)

The general procedure **TP6** was followed using **67** (0.1 mmol), **Cs_2CO_3** (0.3 mmol) at 65℃ for 12 h. Purification by column chromatography (petroleum ether/EtOAc 40:1) yielded **95** (30.7 mg, 91%) as a colourless oil. ^{1}H-NMR (400 MHz, $CDCl_3$): δ = 7.95 – 7.82 (m, 2H), 7.50 – 7.42 (m, 2H), 7.13 (d, J = 8.0 Hz, 2H), 7.07 (t, J = 8.8 Hz, 1H), 2.55 (d, J = 7.4 Hz, 1H), 2.30 (d, J = 2.0 Hz, 3H), 2.29 (s, 3H), 2.19 (d, J = 7.4 Hz, 1H). ^{13}C-NMR (100 MHz, $CDCl_3$): δ = 191.9, 163.2, 138.4, 133.7 (d, J_{C-F} = 6.5 Hz), 131.8, 130.9 (d, J_{C-F} = 3.5 Hz), 130.3, 130.1 (d, J_{C-F} = 9.4 Hz), 129.5, 125.7 (d, J_{C-F} = 18.1 Hz), 115.2 (d, J_{C-F} = 22.8 Hz), 61.5, 48.3, 31.8, 21.2, 14.7 (d, J_{C-F} = 3.5 Hz). ^{19}F-NMR (376 MHz, $CDCl_3$): δ = -108.36. HR-MS (ESI) m/z calcd for $C_{18}H_{15}Cl_2FO$ [M+Na$^+$] 359.0376, found 359.0371.

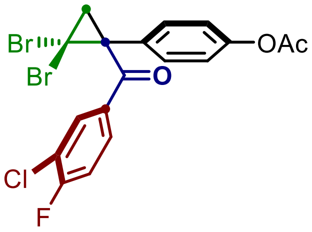

4-[2,2-Dibromo-1-(3-chloro-4-fluorobenzoyl)cyclopropyl]phenyl acetate (96)

The general procedure **TP6** was followed using **72** (0.1 mmol), **Cs_2CO_3** (0.3 mmol) at 25℃ for 4 h. Purification by column chromatography (petroleum ether/EtOAc 10:1) yielded **96** (28.9 mg, 59%) as a colourless oil. ^{1}H-NMR (400 MHz, $CDCl_3$): δ = 8.13 – 8.05 (m, 1H), 8.04 – 7.95

(m, 1H), 7.67 – 7.57 (m, 2H), 7.31 – 7.27 (m, 1H), 7.15 – 7.08 (m, 2H), 2.79 (d, J = 7.8 Hz, 1H), 2.45 (d, J = 7.8 Hz, 1H), 2.31 (s, 3H). ^{13}C-NMR (100 MHz, $CDCl_3$): δ = 190.7, 169.2, 161.2 (d, $J_{C\text{-}F}$ = 257.8 Hz), 150.8, 132.9, 132.7, 131.7, 130.5, 130.5, 122.1, 117.0, 116.8, 47.4, 33.8, 27.3, 21.3. ^{19}F-NMR (376 MHz, $CDCl_3$): δ = -106.32. HR-MS (ESI) m/z calcd for $C_{18}H_{12}Br_2ClFO_3$ [M+H$^+$] 488.8899, found 488.8897.

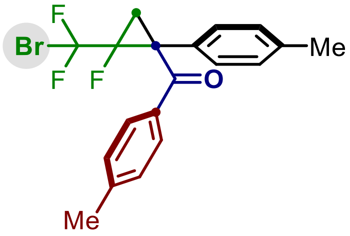

[2-(Bromodifluoromethyl)-2-fluoro-1-(p-tolyl)cyclopropyl](p-tolyl)methanone (97)

The general procedure **TP6** was followed using **77** (0.1 mmol), **Cs_2CO_3** (0.3 mmol) at 65℃ for 12 h. Purification by column chromatography (petroleum ether/EtOAc 40:1) yielded **97** (29.9 mg, 76%, dr = 5.7:1) as a colourless oil. ^{1}H-NMR (400 MHz, $CDCl_3$): δ = 7.93 (d, J = 8.3 Hz, 2H), 7.49 (d, J = 8.0 Hz, 2H), 7.21 (d, J = 8.0 Hz, 2H), 7.16 (d, J = 8.0 Hz, 2H), 2.36 (s, 3H), 2.30 (s, 3H), 2.26 – 2.19 (m, 1H), 2.16 – 2.08 (m, 1H). ^{13}C-NMR (100 MHz, $CDCl_3$): δ = 191.4, 191.3, 144.6, 144.5, 138.5, 138.4, 132.4, 131.9, 130.1, 130.1, 129.9, 129.8, 129.8, 129.7, 129.6, 129.4, 129.3, 121.7 – 113.6 (m), 84.7 – 83.6 (m), 82.1 – 81.3 (m), 46.0 (d, $J_{C\text{-}F}$ = 12.1 Hz), 45.4 (d, $J_{C\text{-}F}$ = 10.3 Hz), 21.8, 21.2, 20.7, 20.6. ^{19}F-NMR (376 MHz, $CDCl_3$): δ = -52.66 (dd, J = 175.0, 13.9 Hz), -54.41 (dd, J = 176.0, 13.8 Hz), -54.80 (dd, J = 177.7, 14.5 Hz), -56.04 (dd, J = 177.6, 14.7 Hz), -180.59 – -184.83 (m). HR-MS (ESI) m/z calcd for $C_{19}H_{16}BrF_3O$ [M+Na$^+$] 419.0229, found 419.0203.

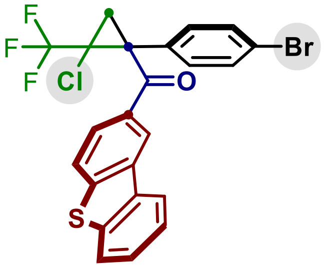

[1-(4-Bromophenyl)-2-chloro-2-(trifluoromethyl)cyclopropyl](dibenzo[b,d]thiophen-2-yl)methanone (98)

The general procedure **TP6** was followed using **69** (0.1 mmol), **Cs_2CO_3** (0.3 mmol) at 65℃ for 12 h. Purification by column chromatography (petroleum ether/EtOAc 20:1) yielded **98** (36.2 mg, 71%, dr > 20:1) as a colourless oil. ^{1}H-NMR (400 MHz, $CDCl_3$): δ = 8.77 (d, J = 1.7 Hz,

1H), 8.25 – 8.18 (m, 1H), 8.11 – 8.03 (m, 1H), 7.93 – 7.83 (m, 2H), 7.62 – 7.49 (m, 6H), 2.51 (d, J = 7.6 Hz, 1H), 2.26 (d, J = 7.8 Hz, 1H). ^{13}C-NMR (100 MHz, $CDCl_3$): δ = 191.8, 145.7, 139.8, 136.0, 135.0, 132.8, 132.3, 131.8, 130.6, 127.9, 126.9, 125.3, 123.4, 123.2, 123.0, 122.9, 122.0, 46.9 (d, $J_{C\text{-}F}$ = 40.0 Hz), 44.9, 23.1. ^{19}F-NMR (376 MHz, $CDCl_3$): δ = -108.36. HR-MS (ESI) m/z calcd for $C_{23}H_{13}BrClF_3OS$ [M+H$^+$] 508.9584, found 508.9582.

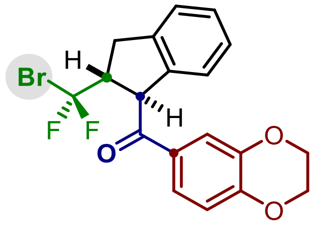

[2-(Bromodifluoromethyl)-2,3-dihydro-1H-inden-1-yl](2,3-dihydrobenzo[b][1,4]dioxin-6-yl)methanone (99)

The general procedure **TP7** was followed using **1q** (0.4 mmol), **2ac** (0.2 mmol) and **3m** (0.6 mmol) for 2 h. Purification by column chromatography (petroleum ether/EtOAc 6:1) yielded **99** (53.2 mg, 65%, dr > 20:1) as a colourless oil. ^{1}H-NMR (400 MHz, $CDCl_3$): δ = 7.88 – 7.80 (m, 2H), 7.42 – 7.34 (m, 2H), 7.24 (t, J = 7.6 Hz, 1H), 7.20 – 7.14 (m, 1H), 7.07 (d, J = 7.9 Hz, 1H), 5.36 (d, J = 7.1 Hz, 1H), 4.49 – 4.39 (m, 4H), 4.33 – 4.21 (m, 1H), 3.56 – 3.46 (m, 1H), 3.39 – 3.30 (m, 1H). ^{13}C-NMR (100 MHz, $CDCl_3$): δ = 196.0, 148.9, 143.9, 141.0, 139.7, 130.7, 128.2, 127.3, 125.1, 124.3, 123.6, 118.8, 117.8, 64.9, 64.3, 53.9, 53.7, 53.6 – 53.4 (m), 34.4 – 34.2 (m). ^{19}F-NMR (376 MHz, $CDCl_3$): δ = -47.85 (d, J = 158.2 Hz), -48.44 (d, J = 158.3 Hz). HR-MS (ESI) m/z calcd for $C_{19}H_{15}BrF_2O_3$ [M+H$^+$] 409.0245, found 409.0241.

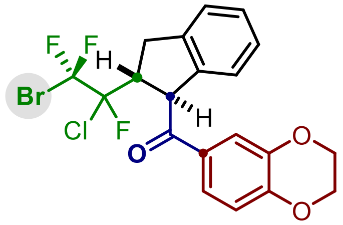

[2-(2-Bromo-1-chloro-1,2,2-trifluoroethyl)-2,3-dihydro-1H-inden-1-yl](2,3-dihydrobenzo[b][1,4]dioxin-6-yl)methanone (100)

The general procedure **TP7** was followed using **1aa** (0.4 mmol), **2ac** (0.2 mmol) and **3m** (0.6 mmol) for 2 h. Purification by column chromatography (petroleum ether/EtOAc 5:1) yielded **100** (60.2 mg, 63%, dr = 1:1) as a colourless oil. ^{1}H-NMR (400 MHz, $CDCl_3$): δ = 7.72 – 7.62 (m, 2H), 7.25 – 7.16 (m, 2H), 7.12 – 6.99 (m, 2H), 6.94 – 6.80 (m, 1H), 5.47 – 5.29 (m, 1H), 4.54 – 4.44 (m, 1H), 4.41 – 4.30 (m, 4H), 3.46 – 3.18 (m, 2H). ^{13}C-NMR (100 MHz, $CDCl_3$):

δ = 196.2, 196.1, 148.9, 148.9, 143.9, 143.9, 141.1, 141.1, 139.9, 139.9, 130.9, 130.8, 128.1, 128.1, 127.3, 127.3, 124.9, 124.8, 124.0, 123.5, 118.8, 117.9, 117.9, 64.9, 64.3, 54.2, 53.4, 47.6 (d, $J_{C\text{-}F}$ = 19.4 Hz), 47.1 (d, $J_{C\text{-}F}$ = 21.0 Hz), 35.1 – 34.6 (m), 34.4 – 34.0 (m). ^{19}F-NMR (376 MHz, $CDCl_3$): δ = -49.06 – -67.75 (m), -104.98 – -126.11 (m). HR-MS (ESI) m/z calcd for $C_{20}H_{15}BrClF_3O_3$ [M+H$^+$] 474.9918, found 474.9919.

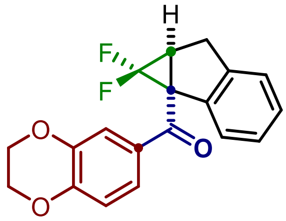

[1,1-Difluoro-6,6a-dihydrocyclopropa[a]inden-1a(1H)-yl](2,3-dihydrobenzo[b][1,4]dioxin-6-yl)methanone (101)

The general procedure **TP7** was followed using **99** (0.1 mmol), **Cs_2CO_3** (0.3 mmol) at 25℃ for 12 h. Purification by column chromatography (petroleum ether/EtOAc 4:1) yielded **101** (25.1 mg, 76%) as a colourless oil. ^{1}H-NMR (400 MHz, $CDCl_3$): δ = 7.65 – 7.54 (m, 2H), 7.26 – 7.19 (m, 2H), 7.14 – 7.05 (m, 2H), 7.03 – 6.97 (m, 1H), 4.39 – 4.29 (m, 4H), 3.55 (dd, J = 17.4, 7.7 Hz, 1H), 3.33 (d, J = 17.3 Hz, 1H), 2.94 (dd, J = 14.3, 7.6 Hz, 1H). ^{13}C-NMR (100 MHz, $CDCl_3$): δ = 189.8, 149.2, 143.9, 143.3, 136.8, 130.7, 128.0, 127.0, 125.1, 124.8, 124.4, 119.1, 117.8, 64.9, 64.2, 51.9, 31.5, 31.5 – 31.1 (m). ^{19}F-NMR (376 MHz, $CDCl_3$): δ = -128.56 (d, J = 148.4 Hz), -146.45 (d, J = 148.5 Hz). HR-MS (ESI) m/z calcd for $C_{19}H_{14}F_2O_3$ [M+Na$^+$] 351.0803, found 351.0801.

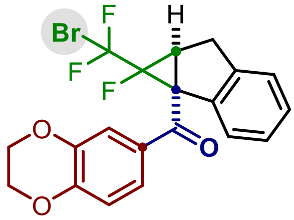

[1-(Bromodifluoromethyl)-1-fluoro-6,6a-dihydrocyclopropa[a]inden-1a(1H)-yl](2,3-dihydrobenzo[b][1,4]dioxin-6-yl)methanone (102)

The general procedure **TP7** was followed using **100** (0.17 mmol), **Cs_2CO_3** (0.51 mmol) at 65℃ for 12 h. Purification by column chromatography (petroleum ether/EtOAc 4:1) yielded **102** (54.3 mg, 73%, dr = 1:1) as a colourless oil. ^{1}H-NMR (400 MHz, $CDCl_3$): δ = 7.74 – 7.56 (m, 1H), 7.55 – 7.46 (m, 1H), 7.29 – 7.12 (m, 4H), 7.02 – 6.85 (m, 1H), 4.44 – 4.19 (m, 4H), 3.80 – 3.55 (m, 1H), 3.48 – 3.19 (m, 1H), 3.08 – 2.76 (m, 1H). ^{13}C-NMR (100 MHz, $CDCl_3$): δ =

189.3, 189.1, 149.1, 149.0, 144.6 – 144.4 (m), 143.8, 143.6, 143.6, 143.5, 136.7, 136.6, 135.9, 130.1, 129.3, 128.5, 128.4, 127.2, 125.3, 125.1, 124.8, 124.6, 124.6, 124.3, 124.2, 124.2, 119.2, 119.1, 117.8, 117.5, 85.3 – 84.0 (m), 83.0 – 81.7 (m), 64.9, 64.9, 64.2, 64.2, 53.9, 53.8, 53.7, 53.6, 33.6, 33.6, 33.5, 33.5, 32.0, 31.3, 31.3, 31.2, 31.2. ^{19}F-NMR (376 MHz, $CDCl_3$): δ = -49.58 (dd, J = 175.5, 15.8 Hz), -52.60 (dd, J = 176.2, 13.7 Hz), -53.59 (dd, J = 176.3, 16.3 Hz), -56.34 (dd, J = 176.6, 14.1 Hz), -159.74 – -210.85 (m). HR-MS (ESI) m/z calcd for $C_{20}H_{14}BrF_3O_3$ [M+H$^+$] 439.0151, found 439.0150.

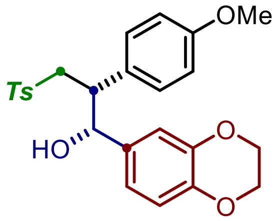

1-(2,3-Dihydrobenzo[b][1,4]dioxin-6-yl)-2-(4-methoxyphenyl)-3-tosylpropan-1-ol (103)

The general procedure **TP8** was followed using **32** (0.5 mmol), **NaBH$_4$** (1.0 mmol) at 25℃ for 2 h. Purification by column chromatography (petroleum ether/EtOAc 2:1) yielded **103** (165.1 mg, 73%, dr > 20:1) as a white solid. ^{1}H-NMR (400 MHz, $CDCl_3$): δ = 7.50 – 7.44 (m, 2H), 7.17 – 7.11 (m, 2H), 6.88 – 6.81 (m, 2H), 6.72 (d, J = 8.3 Hz, 1H), 6.66 – 6.60 (m, 3H), 6.52 (dd, J = 8.3, 2.1 Hz, 1H), 4.75 (d, J = 5.9 Hz, 1H), 4.22 (s, 4H), 3.73 (s, 3H), 3.56 – 3.49 (m, 1H), 3.47 – 3.35 (m, 2H), 2.37 (s, 3H). ^{13}C-NMR (100 MHz, $CDCl_3$): δ = 158.9, 144.2, 143.4, 143.3, 136.7, 134.3, 130.1, 129.6, 129.2, 128.0, 119.7, 117.1, 115.7, 113.8, 76.4, 64.4, 64.4, 58.4, 55.3, 47.7, 21.7. HR-MS (ESI) m/z calcd for $C_{25}H_{26}O_6S$ [M+H$^+$] 455.1523, found 455.1523.

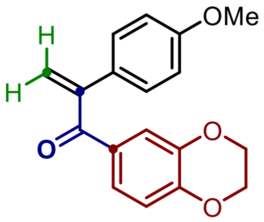

1-(2,3-Dihydrobenzo[b][1,4]dioxin-6-yl)-2-(4-methoxyphenyl)prop-2-en-1-one (104)

The general procedure **TP9** was followed using **32** (1.0 mmol), **K_2CO_3** (2.0 mmol) at 25℃ for 2.5 h. Purification by column chromatography (petroleum ether/EtOAc 5:1) yielded **104** (270.1 mg, 91%) as a colourless oil. ^{1}H-NMR (400 MHz, $CDCl_3$): δ = 7.56 – 7.43 (m, 2H), 7.39 – 7.30 (m, 2H), 6.91 – 6.80 (m, 3H), 5.89 (s, 1H), 5.43 (s, 1H), 4.33 – 4.22 (m, 4H), 3.80 (s, 3H). ^{13}C-NMR (100 MHz, $CDCl_3$): δ = 196.7, 159.9, 148.3, 147.7, 143.3, 130.8, 129.7, 128.2, 124.5,

119.6, 117.3, 117.2, 114.1, 64.8, 64.2, 55.4. HR-MS (ESI) m/z calcd for $C_{18}H_{16}O_4$ [$M+H^+$] 297.1121, found 297.1125.

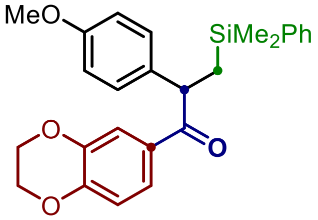

1-(2,3-Dihydrobenzo[b][1,4]dioxin-6-yl)-3-[dimethyl(phenyl)silyl]-2-(4-methoxyphenyl)propan-1-one (105)

The general procedure **TP10** was followed using **104** (0.1 mmol), **PhMe2Si-ZnOPiv** (0.12 mmol) at 25℃ for 4 h. Purification by column chromatography (petroleum ether/EtOAc 6:1) yielded **105** (37.8 mg, 87%) as a colourless oil. ^{1}H-NMR (400 MHz, $CDCl_3$): δ = 7.49 – 7.30 (m, 7H), 7.20 – 7.08 (m, 2H), 6.87 – 6.72 (m, 3H), 4.48 (t, J = 7.3 Hz, 1H), 4.31 – 4.15 (m, 4H), 3.74 (s, 3H), 1.79 – 1.67 (m, 1H), 1.44 – 1.34 (m, 1H), 0.13 (d, J = 2.9 Hz, 6H). ^{13}C-NMR (100 MHz, $CDCl_3$): δ = 199.0, 158.6, 147.8, 143.3, 138.9, 133.8, 133.7, 130.5, 129.3, 129.0, 127.9, 122.9, 118.3, 117.2, 114.3, 64.7, 64.2, 55.3, 47.9, 21.3, -2.3, -2.5. HR-MS (ESI) m/z calcd for $C_{26}H_{28}O_4Si$ [$M+Na^+$] 455.1649, found 455.1642.

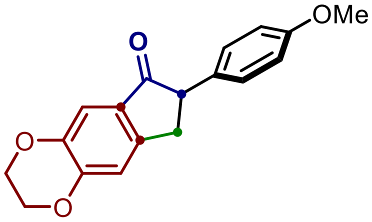

7-(4-Methoxyphenyl)-2,3,7,8-tetrahydro-6H-indeno[5,6-b][1,4]dioxin-6-one (106)

The general procedure **TP11** was followed using **104** (0.1 mmol) at 0 - 25℃ for 1 h. Purification by column chromatography (petroleum ether/EtOAc 3:1) yielded **106** (24.1 mg, 81%) as a colourless oil. ^{1}H-NMR (400 MHz, $CDCl_3$): δ = 7.29 (s, 1H), 7.13 – 7.04 (m, 2H), 6.95 (d, J = 1.0 Hz, 1H), 6.88 – 6.81 (m, 2H), 4.37 – 4.24 (m, 4H), 3.82 – 3.73 (m, 4H), 3.58 – 3.49 (m, 1H), 3.12 – 3.03 (m, 1H). ^{13}C-NMR (100 MHz, $CDCl_3$): δ = 205.0, 158.6, 150.4, 147.8, 144.1, 132.3, 129.9, 128.9, 114.4, 114.0, 112.4, 64.8, 64.0, 55.4, 53.1, 35.4. HR-MS (ESI) m/z calcd for $C_{18}H_{16}O_4$ [$M+H^+$] 297.1121, found 297.1120.

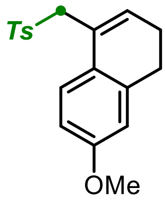

7-Methoxy-4-(tosylmethyl)-1,2-dihydronaphthalene (108)

The procedure **g** was followed using **1a** (0.4 mmol), **3a** (0.4 mmol) and **107** (0.2 mmol) for 2 h. Purification by column chromatography (petroleum ether/EtOAc 6:1) yielded **108** (20.1 mg, 30%) as a colorless oil. ^{1}H-NMR (400 MHz, $CDCl_3$): δ = 7.69 – 7.64 (m, 2H), 7.23 (d, J = 8.0 Hz, 2H), 7.10 (d, J = 8.4 Hz, 1H), 6.65 – 6.60 (m, 2H), 5.71 (t, J = 4.7 Hz, 1H), 4.17 (s, 2H), 3.78 (s, 3H), 2.65 (t, J = 8.0 Hz, 2H), 2.39 (s, 3H), 2.22 – 2.15 (m, 2H). ^{13}C-NMR (100 MHz, $CDCl_3$): δ = 158.9, 144.6, 138.0, 135.5, 132.3, 129.5, 128.8, 125.9, 125.6, 124.8, 114.0, 110.9, 60.4, 55.4, 28.4, 23.4, 21.7. HR-MS (ESI) m/z calcd for $C_{19}H_{20}O_3S$ [M+H$^+$] 329.1206, found 329.1208.

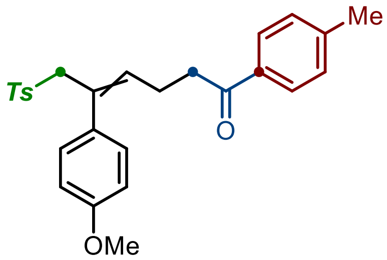

5-(4-Methoxyphenyl)-1-(p-tolyl)-6-tosylhex-4-en-1-one (109)

The procedure **g** was followed using **1a** (0.4 mmol), **3a** (0.4 mmol) and **107** (0.2 mmol) for 2 h. Purification by column chromatography (petroleum ether/EtOAc 6:1) yielded **109** (8.0 mg, 9%) as a colorless oil. ^{1}H-NMR (400 MHz, $CDCl_3$): δ = 7.84 (d, J = 7.9 Hz, 2H), 7.67 – 7.63 (m, 2H), 7.23 – 7.12 (m, 6H), 6.74 (d, J = 8.8 Hz, 2H), 5.95 (t, J = 7.7 Hz, 1H), 4.39 (s, 2H), 3.77 (s, 3H), 3.00 (t, J = 7.1 Hz, 2H), 2.51 – 2.44 (m, 2H), 2.41 (s, 3H), 2.33 (s, 3H). HR-MS (ESI) m/z calcd for $C_{27}H_{28}O_4S$ [M+H$^+$] 449.1781, found 449.1781.

10. Single Crystal X-Ray Diffraction Studies

Single crystals of compound **49** (CCDC: 2413440) suitable for X-ray diffraction, were obtained by slow evaporation of THF solution. Details for data collection and structure refinement are summarized in Table **S5**.

Table S5. Details for X-ray data collection and structure refinement for compound **49**.

	**49**
Empirical formula	$C_{11}H_9CoI_2N_5$
Formula weight	523.96
Temperature/K	212.99(10)
Crystal description	clear light green
Crystal system	triclinic
Space group	P-1
a/Å	8.3151(8)
b/Å	8.4258(6)
c/Å	11.2998(7)
α/°	110.481(6)
β/°	94.504(7)
γ/°	91.535(7)
Volume/Å^3	738.09(10)
Z	2
ρ_{calc}g/cm^3	2.358
μ/mm^{-1}	5.338
F(000)	486.0
Crystal size/mm^3	0.15 × 0.08 × 0.02
Radiation	Mo Kα (λ = 0.71073)
2Θ range for data collection/°	3.864 to 52.738

Index ranges	$-10 \le h \le 10$<br>$-10 \le k \le 10$<br>$-14 \le l \le 14$
Reflections collected	12601
Independent reflections	3013 [R_{int} = 0.0751, R_{sigma} = 0.0739]
Data/restraints/parameters	3013/1/173
Goodness-of-fit on F^2	1.049
Final R indexes [I>=2σ (I)]	R_1 = 0.0628, wR_2 = 0.1352
Final R indexes [all data]	R_1 = 0.0867, wR_2 = 0.1463
Largest diff. peak/hole / e $Å^{-3}$	2.43/-1.03

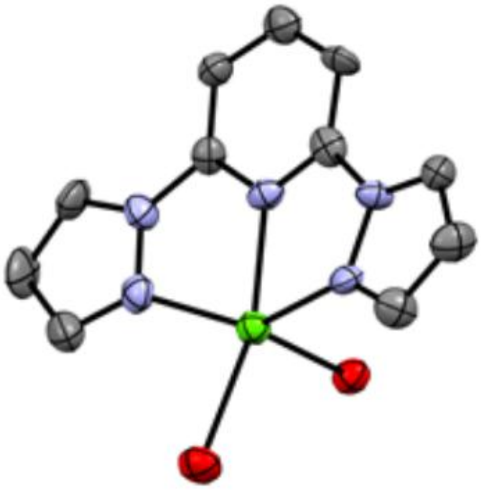

Figure S26. Molecular structure of compound **49** in the crystal.

11. DFT Calculations

Computational Details: All DFT calculations were performed with the Gaussian 16 programs.[9] The geometry optimizations were calculated using the B3LYP functional,[10] with the 6-31G(d) basis set for C, H, N, O, and S atoms and the SDD basis set[11] for Co and I atoms. During the calculations, we tested various spin states of cobalt species. The left superscript on the species name shown in energy profiles denotes its spin multiplicity. Frequency calculations at the same level of theory on the optimized geometries were performed to confirm all located stationary points as either local minima (with zero imaginary frequency) or transition states (with only one imaginary frequency), and to obtain the thermodynamic corrections to Gibbs free energy. Single-point calculations were carried out by using M06 functional,[12] with the 6-311+G(d,p) basis set C, H, N, O, and S atoms and the SDD basis set for Co and I atoms. The solvation energies (in dioxane) were calculated using the self-consistent reaction field with the SMD implicit solvent model.[13] In this work, relative Gibbs free energies in mesitylene solution (including gas-phase corrections) were used to discuss reaction pathways.

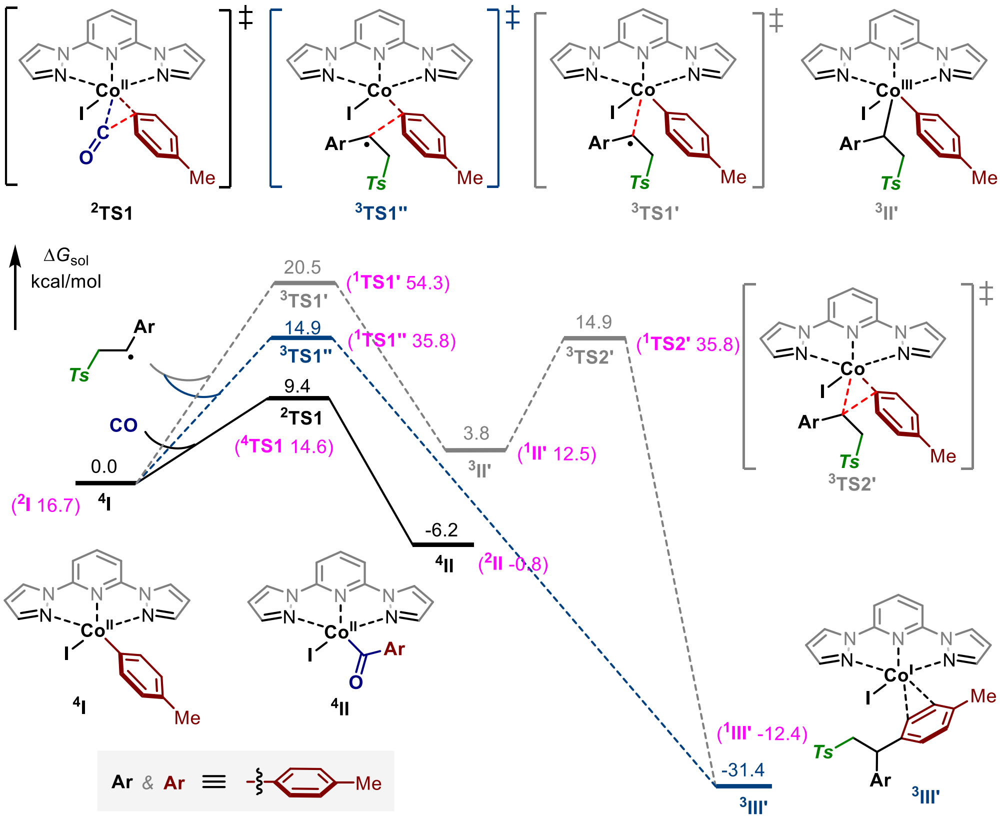

Figure S27. Computed free energy barrier for the reaction of Co^{II}-phenyl complex **^{4}I** with CO or the benzylic radical.

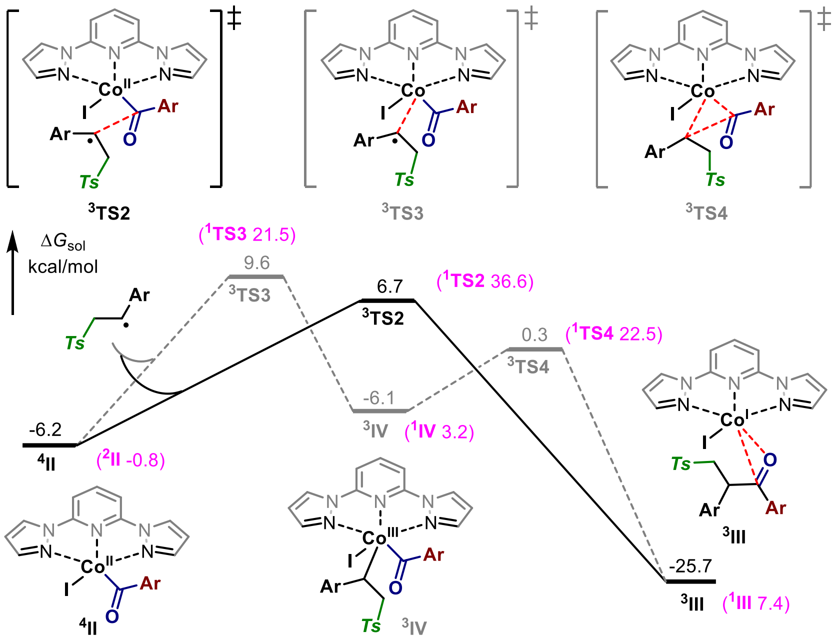

Figure S28. Computed free energy barrier for the reaction between the acyl-Co^{II} complex **^{4}II** and the benzylic radical via a radical-type oxidation and a radical-type substitution pathway.

12. NMR Spectra

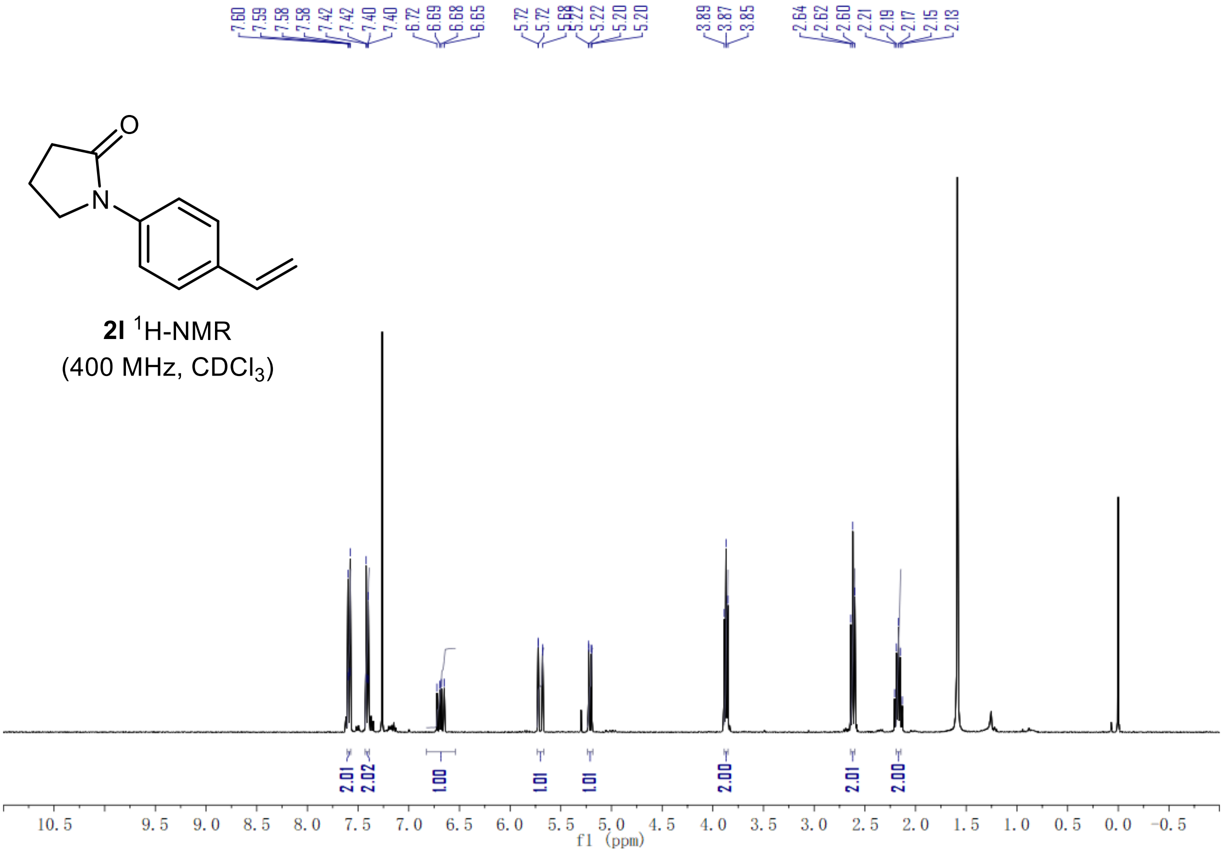

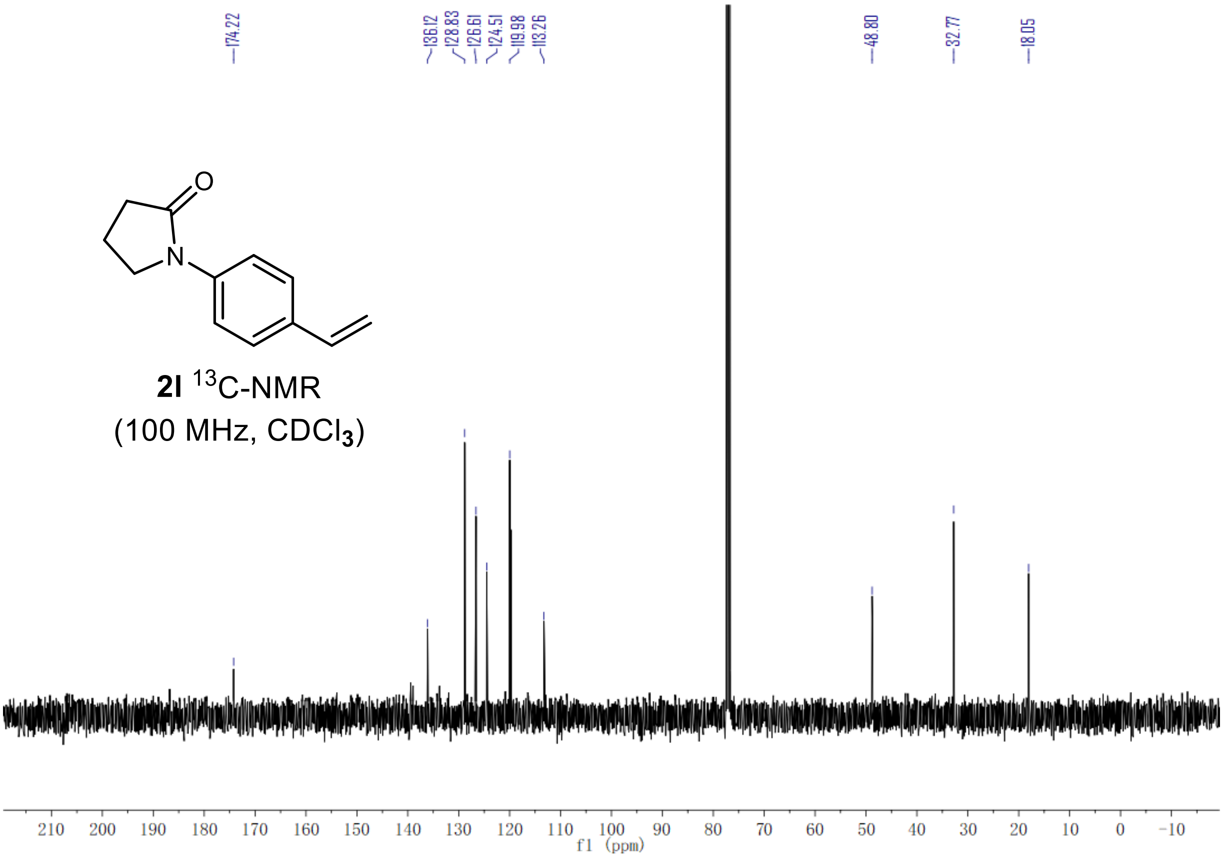

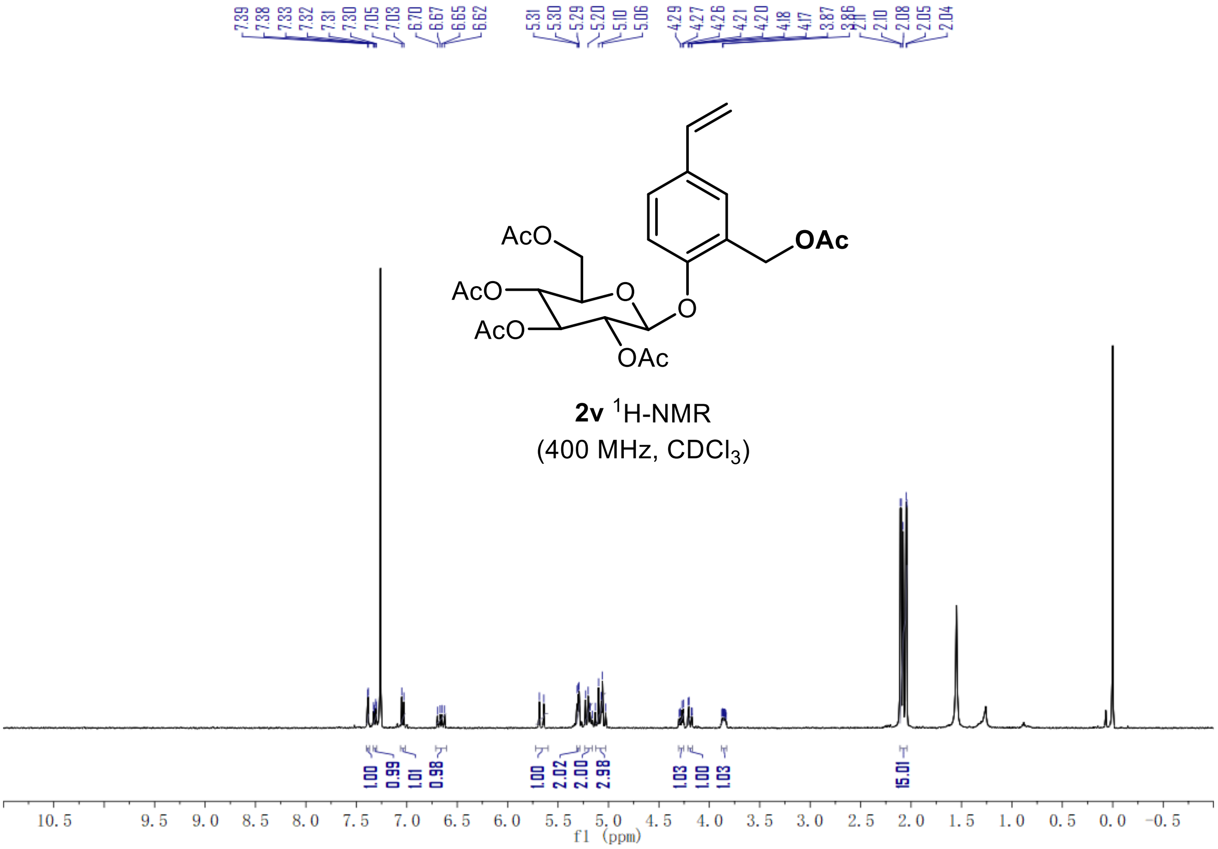
7.39
7.38
7.33
7.32
7.31
7.30
7.05
7.03
6.70
6.67
6.65
6.62
5.31
5.30
5.29
5.20
5.10
5.06
4.29
4.27
4.26
4.21
4.20
4.18
4.17
3.87
3.86
2.11
2.10
2.08
2.05
2.04
AcO
AcO
AcO
O
O
OAc
OAc
2v ¹H-NMR
(400 MHz, CDCl3)
1.00
0.99
1.01
0.98
1.00
2.02
2.00
2.98
1.03
1.00
1.03
15.01
f1 (ppm)

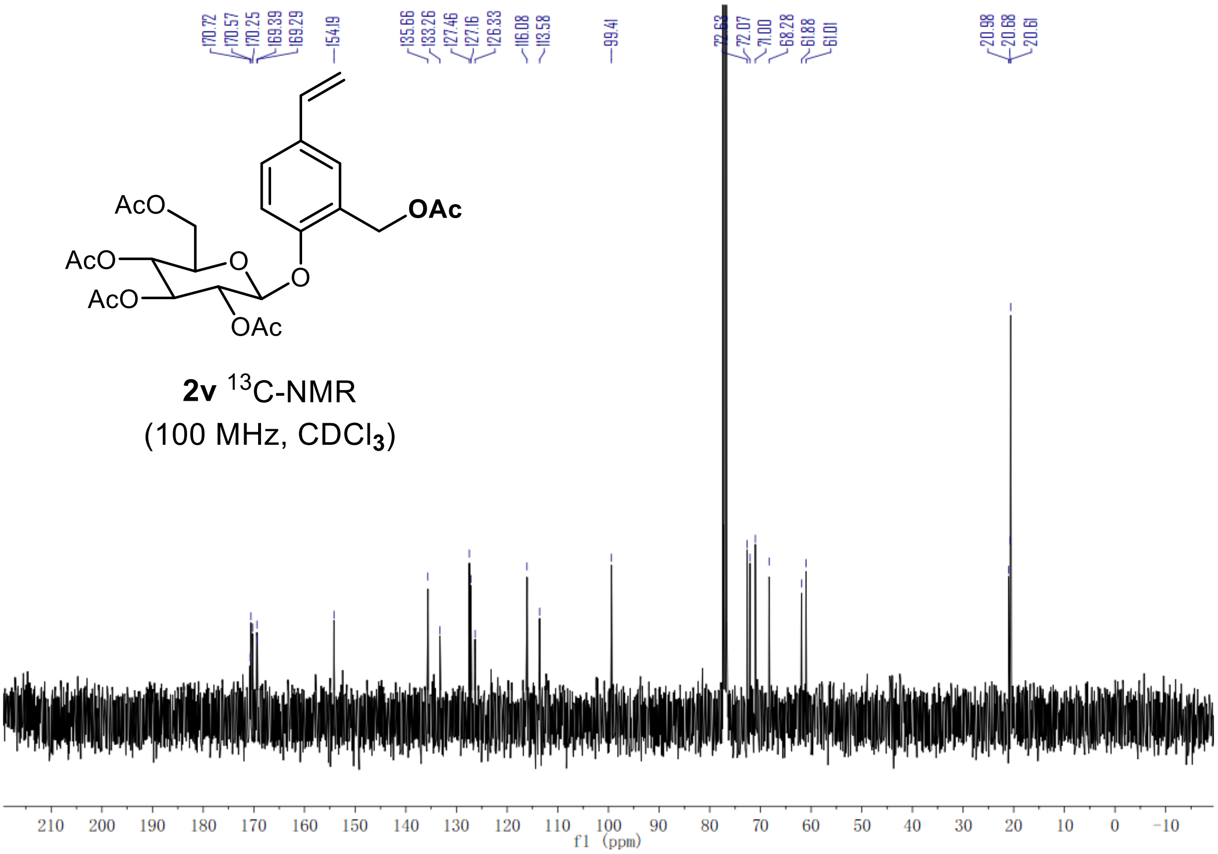
170.72
170.57
170.25
169.39
169.29
154.19
135.66
133.26
127.46
127.16
126.33
116.08
113.58
99.41
72.63
72.07
71.00
68.28
61.88
61.01
20.98
20.68
20.61
AcO
AcO
AcO
O
O
OAc
OAc
2v ¹³C-NMR
(100 MHz, CDCl3)
f1 (ppm)

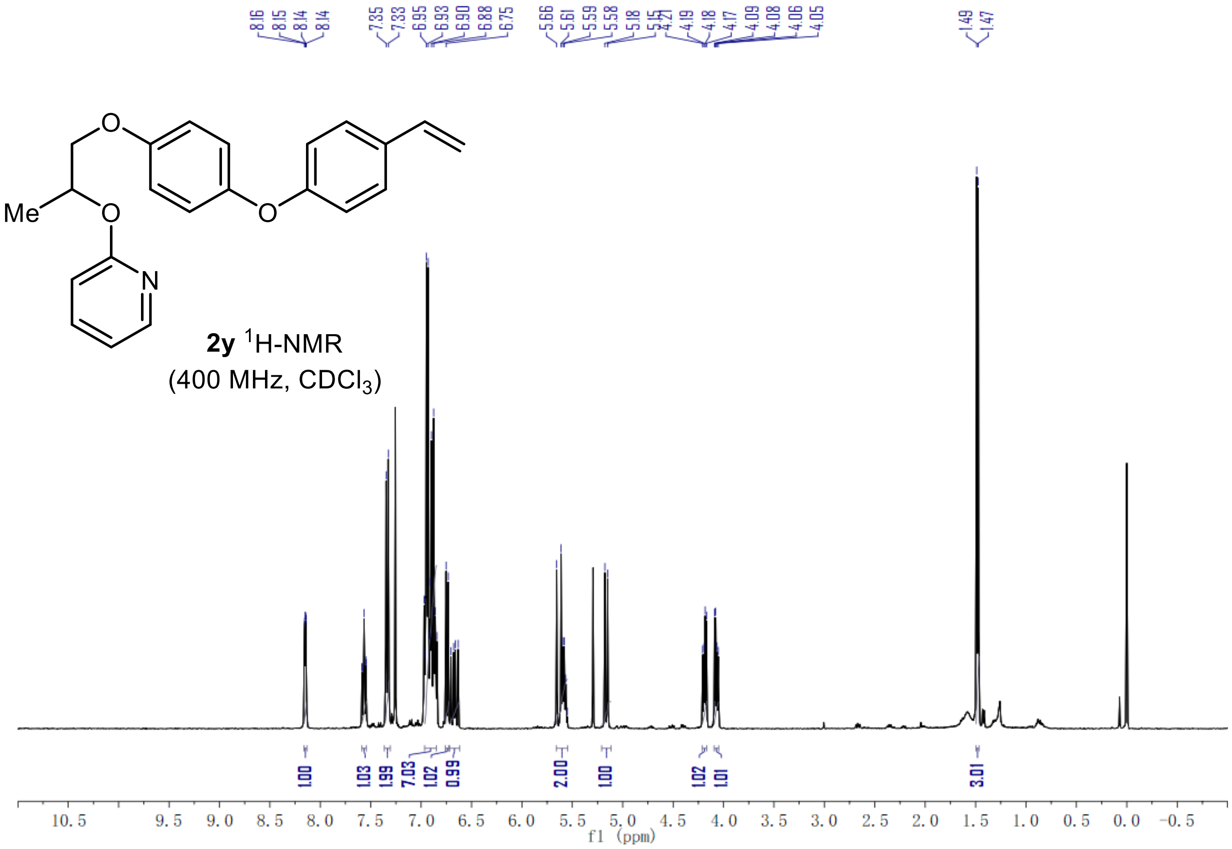

2y ¹H-NMR
(400 MHz, CDCl3)

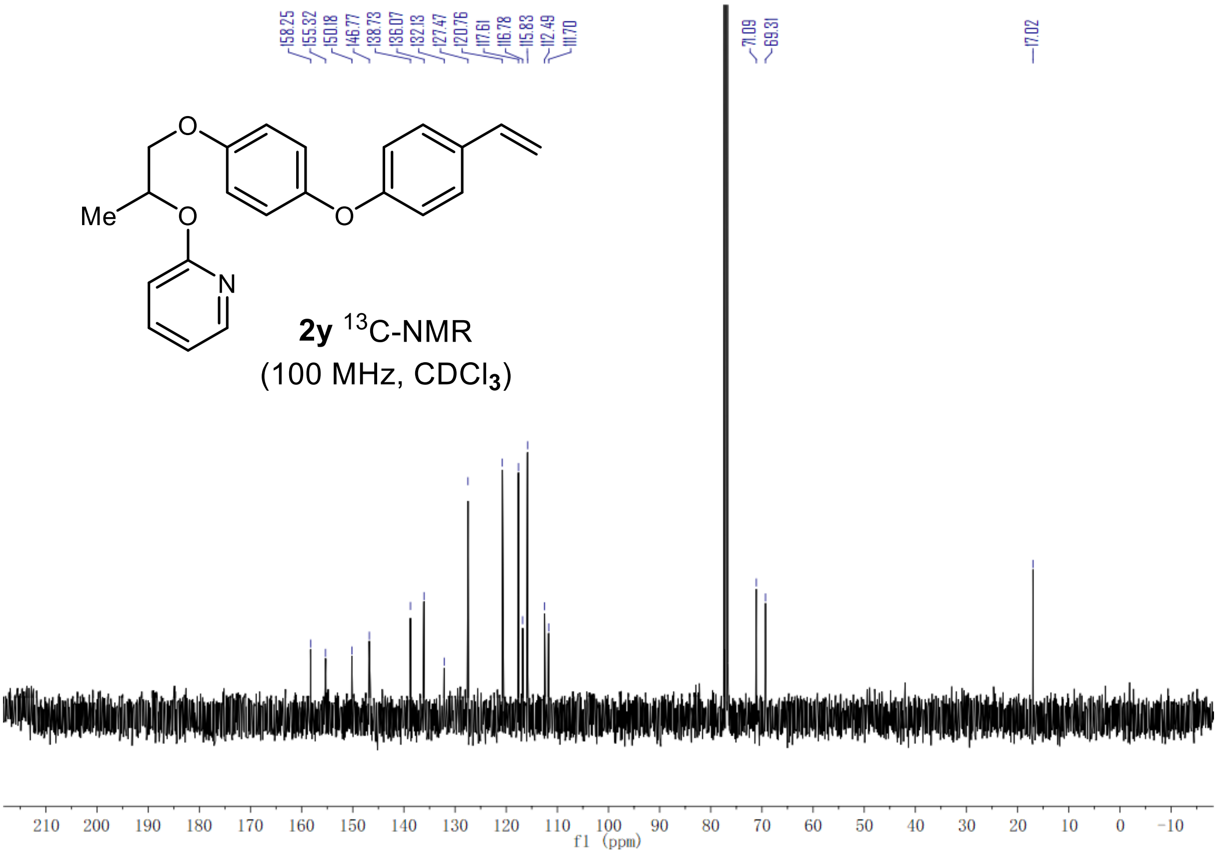

2y ¹³C-NMR
(100 MHz, CDCl3)

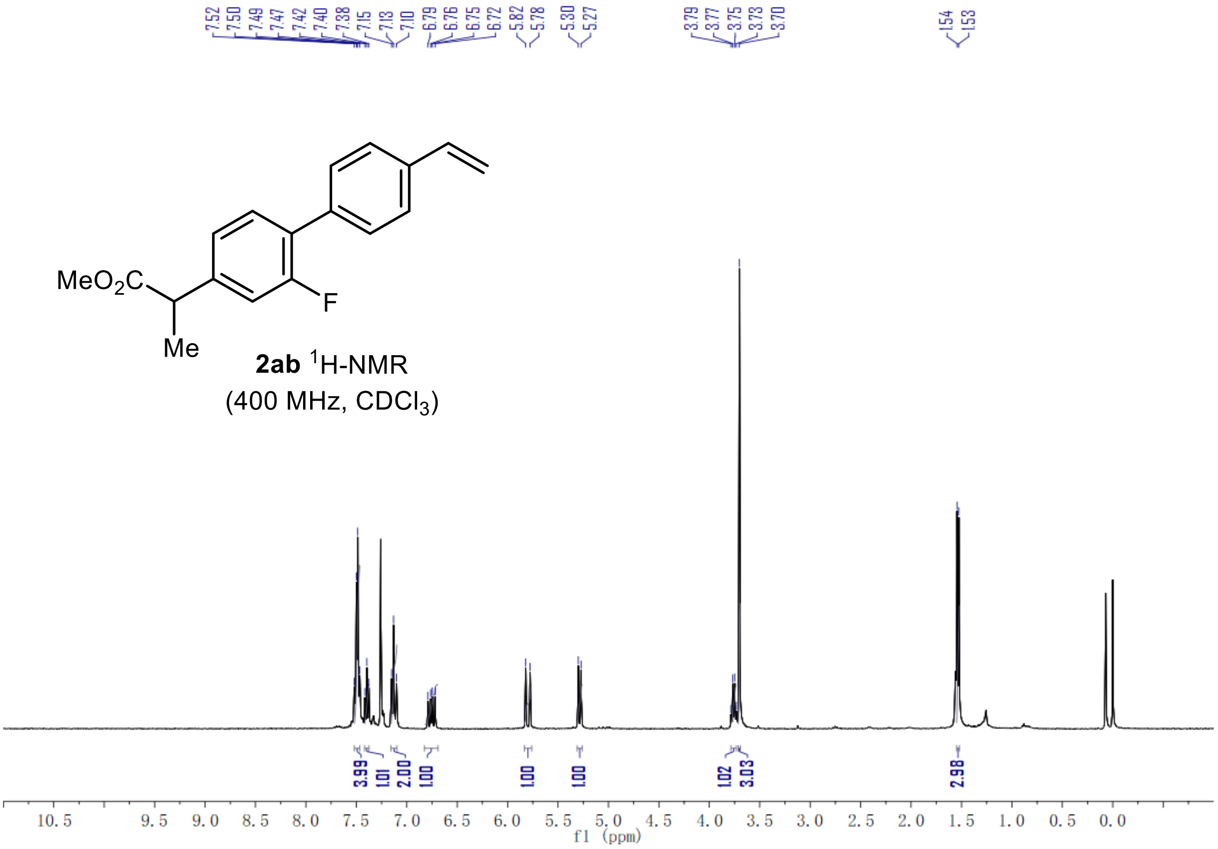
MeO2C
F
Me
2ab 1H-NMR
(400 MHz, CDCl3)
7.52
7.50
7.49
7.47
7.42
7.40
7.38
7.15
7.13
7.10
6.79
6.76
6.75
6.72
5.82
5.78
5.30
5.27
3.79
3.77
3.75
3.73
3.70
1.54
1.53
3.99
1.01
2.00
1.00
1.00
1.00
1.02
3.03
2.98
f1 (ppm)

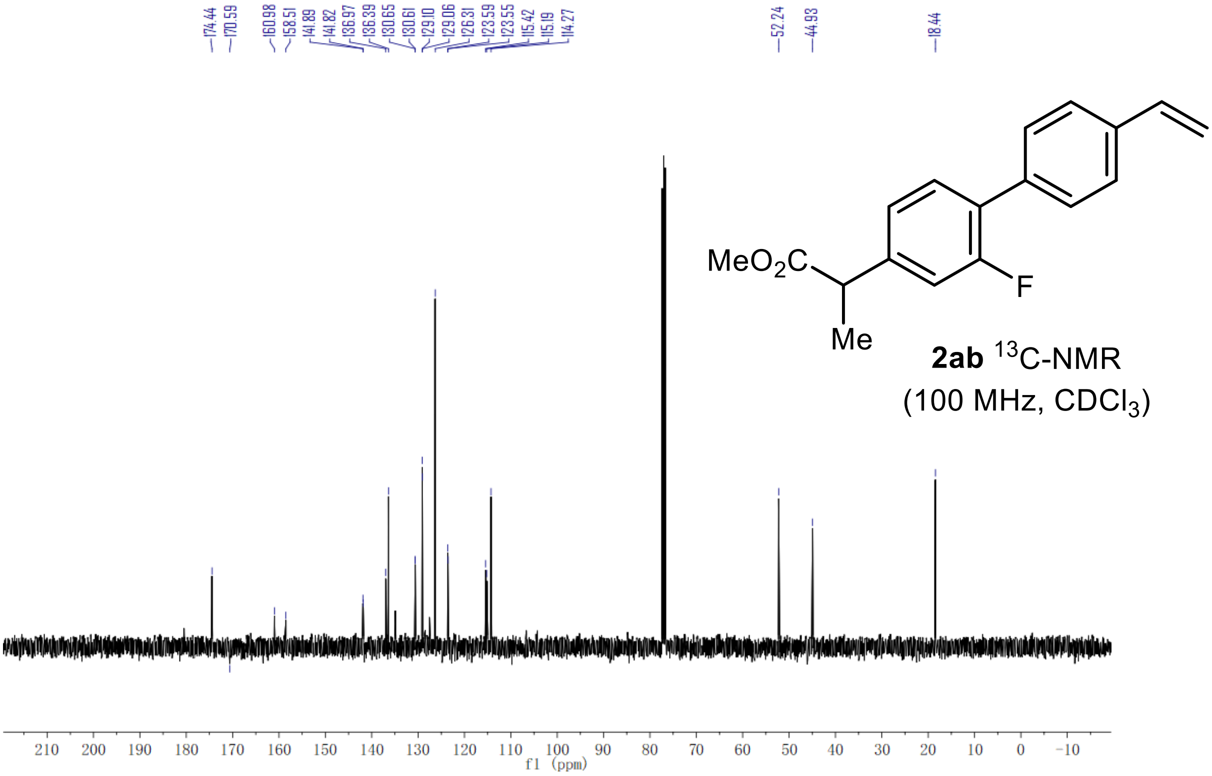
174.44
170.59
160.98
158.51
141.89
141.82
136.97
136.39
130.65
130.61
129.10
129.06
126.31
123.59
123.55
115.42
115.19
114.27
52.24
44.93
18.44
MeO2C
F
Me
2ab 13C-NMR
(100 MHz, CDCl3)
f1 (ppm)

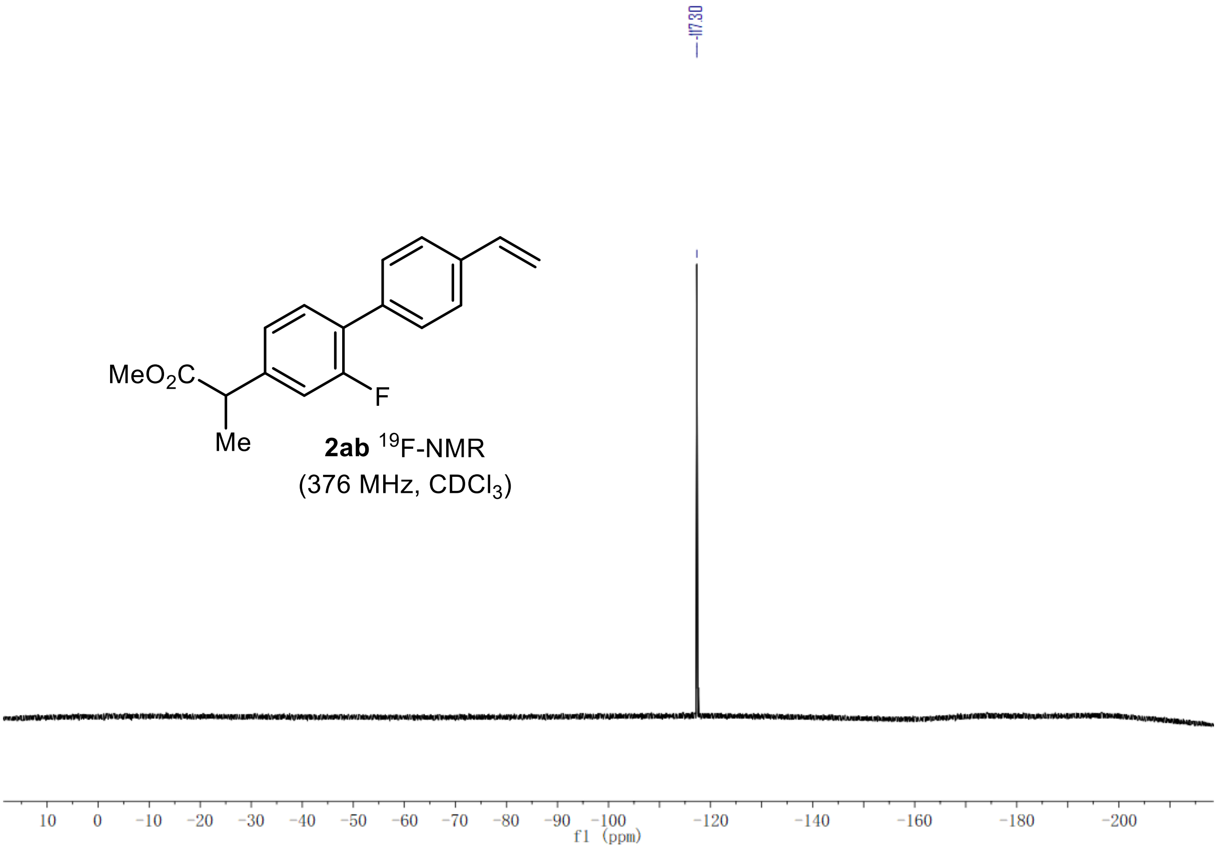

-117.30
MeO2C
F
Me
2ab 19F-NMR
(376 MHz, CDCl3)
f1 (ppm)

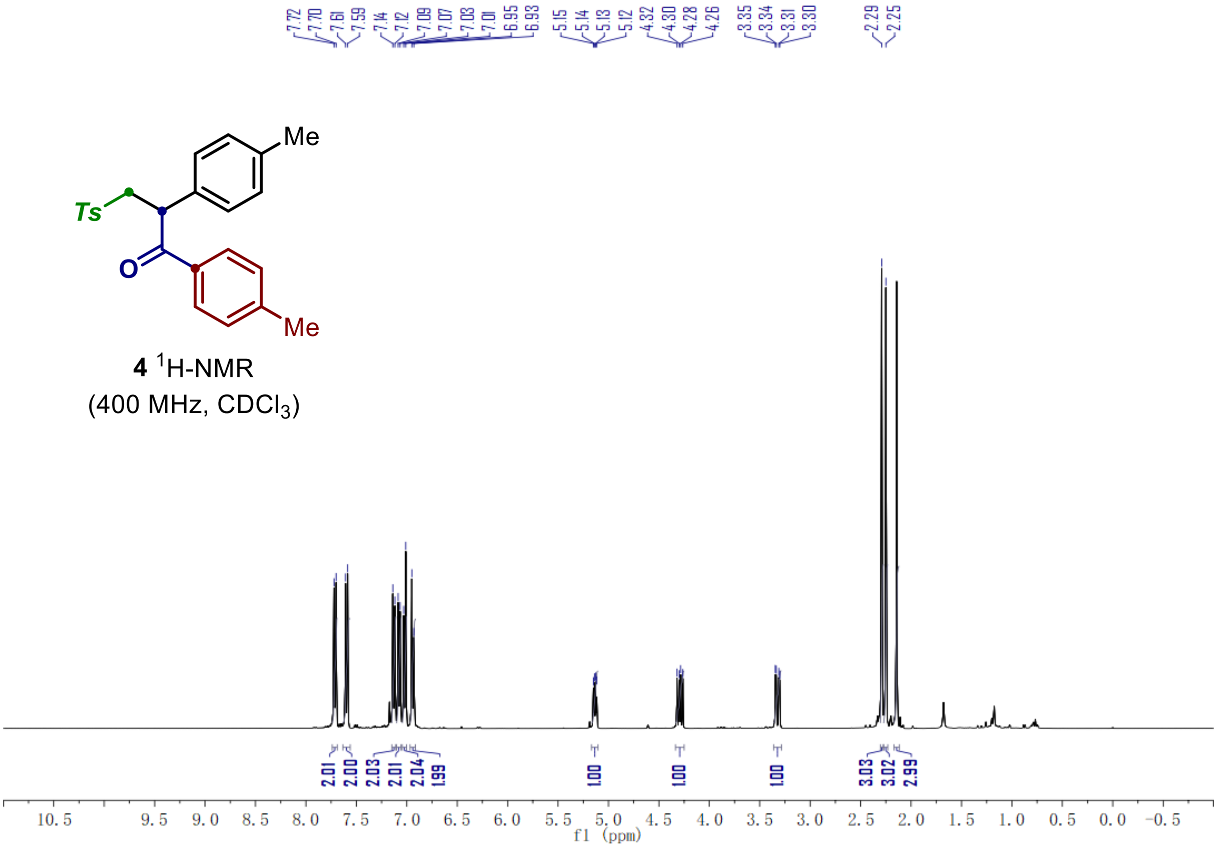

7.72
7.70
7.61
7.59
7.14
7.12
7.09
7.07
7.03
7.01
6.95
6.93
5.15
5.14
5.13
5.12
4.32
4.30
4.28
4.26
3.35
3.34
3.31
3.30
2.29
2.25
Me
Ts
O
Me
4 1H-NMR
(400 MHz, CDCl3)
2.01
2.00
2.03
2.01
2.04
1.99
1.00
1.00
1.00
3.03
3.02
2.99
f1 (ppm)

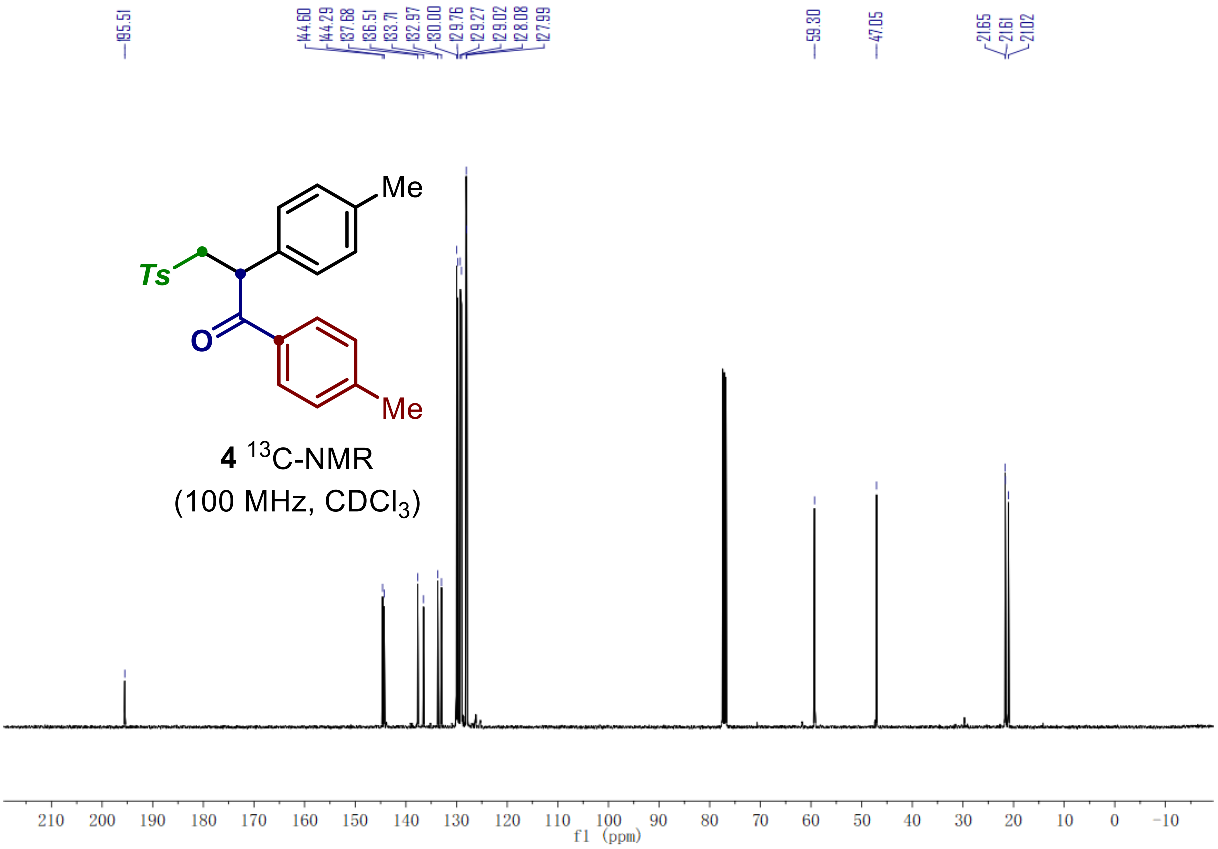
4 13C-NMR
(100 MHz, CDCl3)
f1 (ppm)

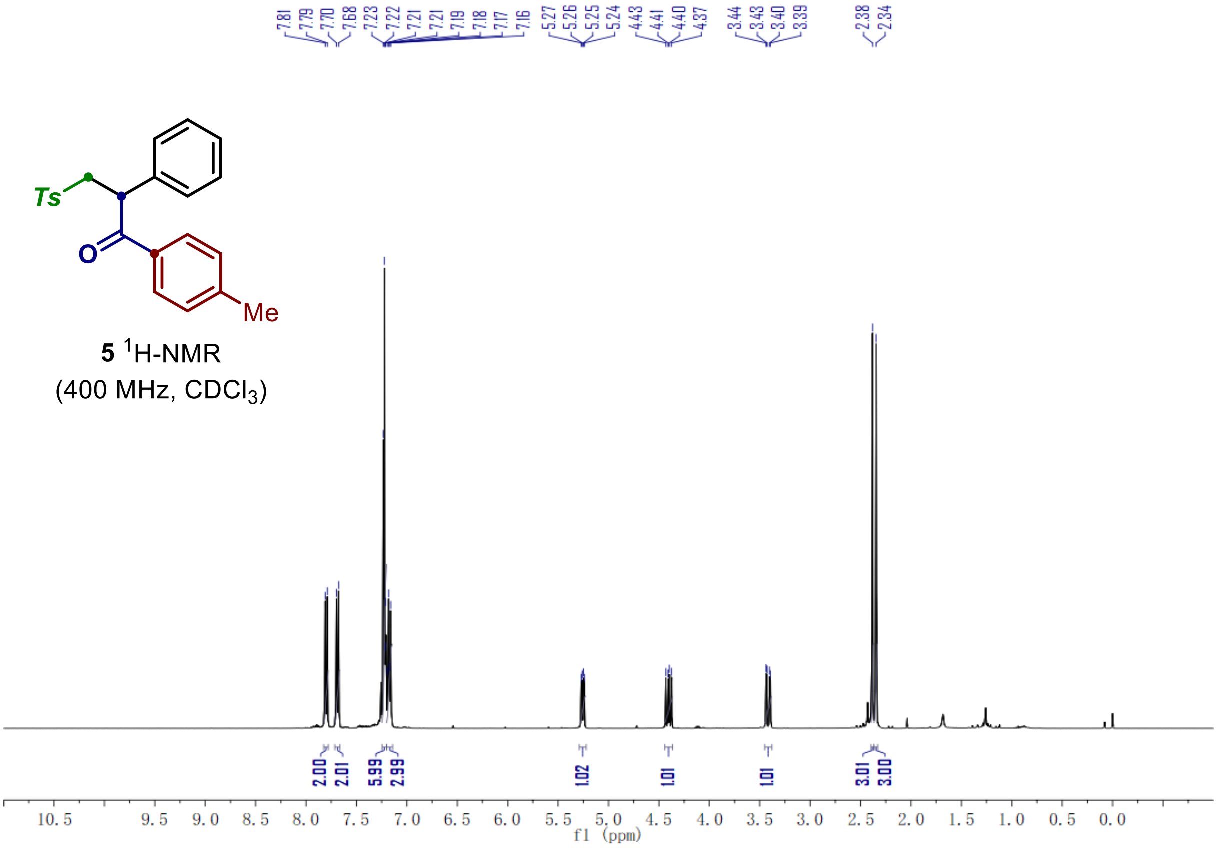
5 1H-NMR
(400 MHz, CDCl3)
f1 (ppm)

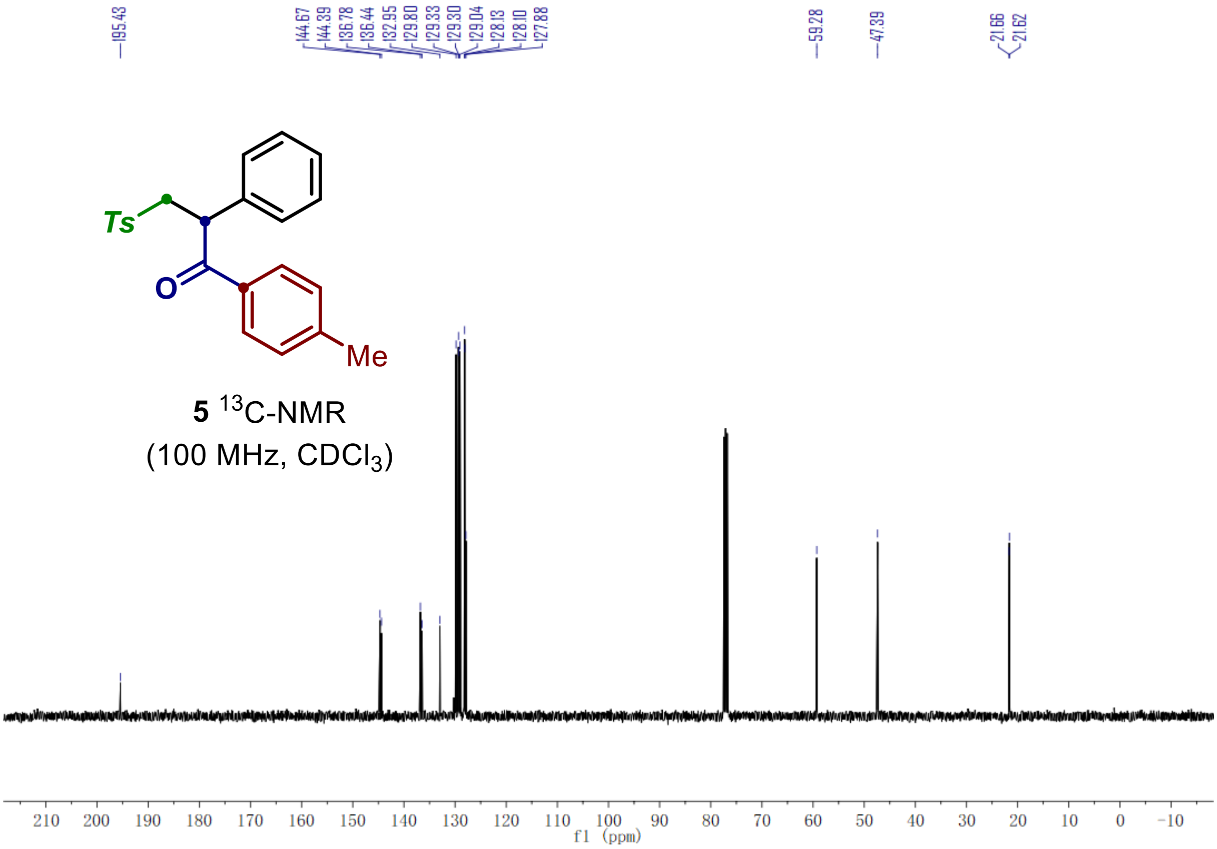
195.43
144.67
144.39
136.78
136.44
132.95
129.80
129.33
129.30
129.04
128.13
128.10
127.88
59.28
47.39
21.66
21.62
Ts
O
Me
5 13C-NMR
(100 MHz, CDCl3)
f1 (ppm)

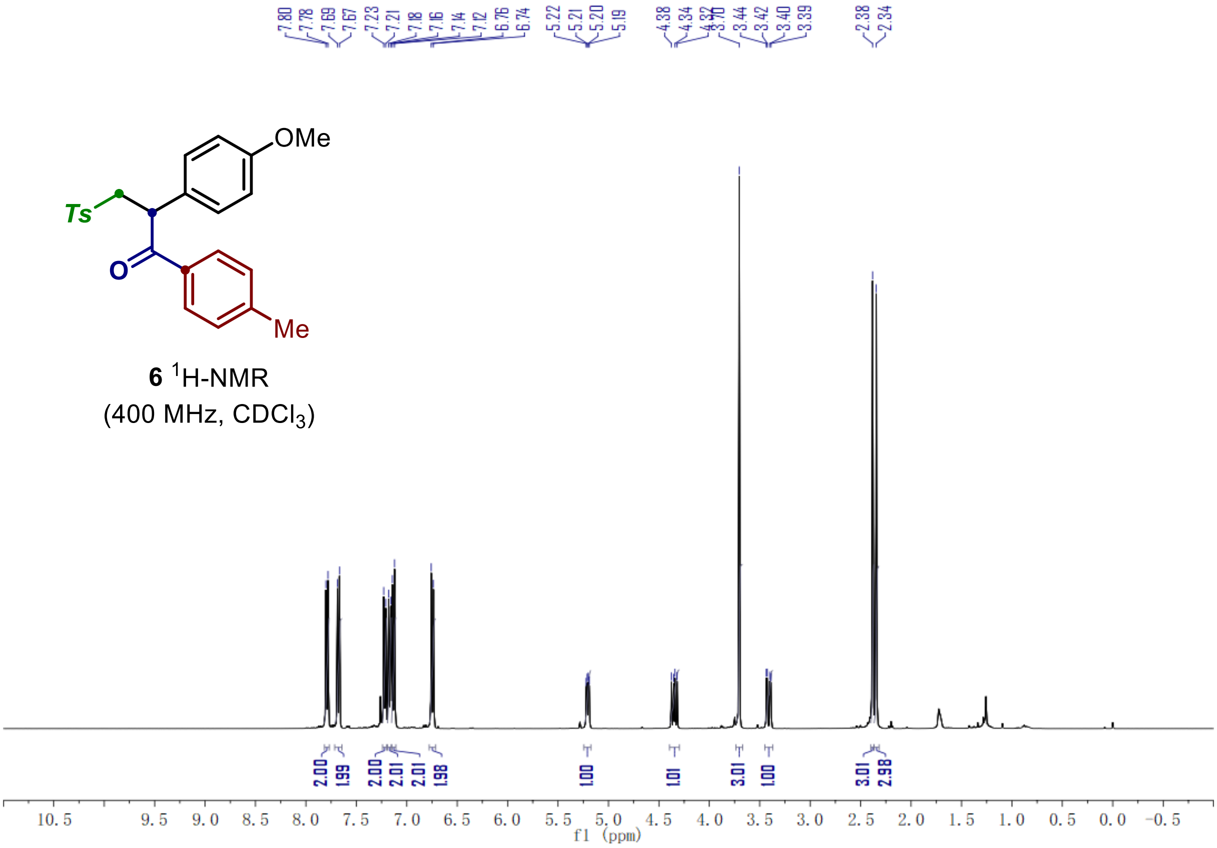
7.80
7.78
7.69
7.67
7.23
7.21
7.18
7.16
7.14
7.12
6.76
6.74
5.22
5.21
5.20
5.19
4.38
4.34
3.70
3.44
3.42
3.40
3.39
2.38
2.34
OMe
Ts
O
Me
6 1H-NMR
(400 MHz, CDCl3)
2.00
1.99
2.00
2.01
2.01
1.98
1.00
1.01
3.01
1.00
3.01
2.98
f1 (ppm)

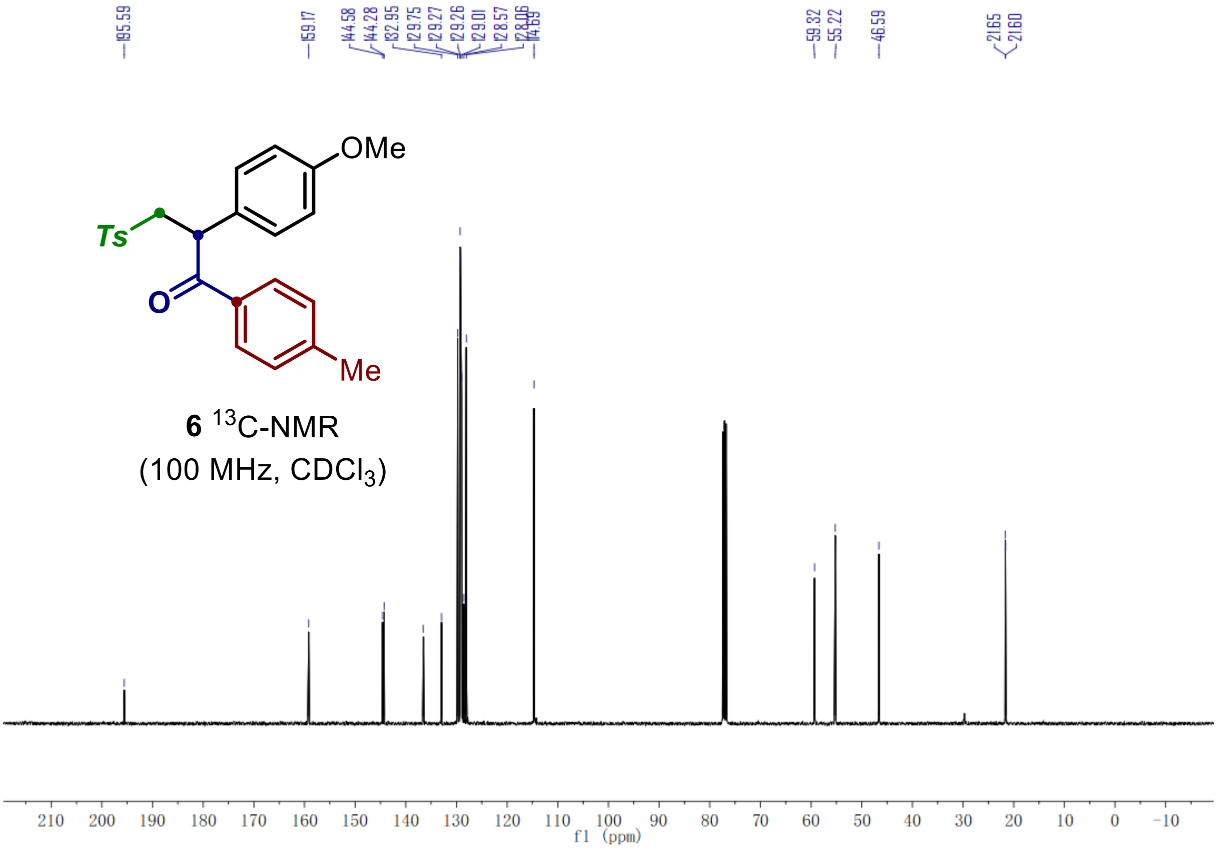
OMe
Ts
O
Me
6 13C-NMR
(100 MHz, CDCl3)
f1 (ppm)

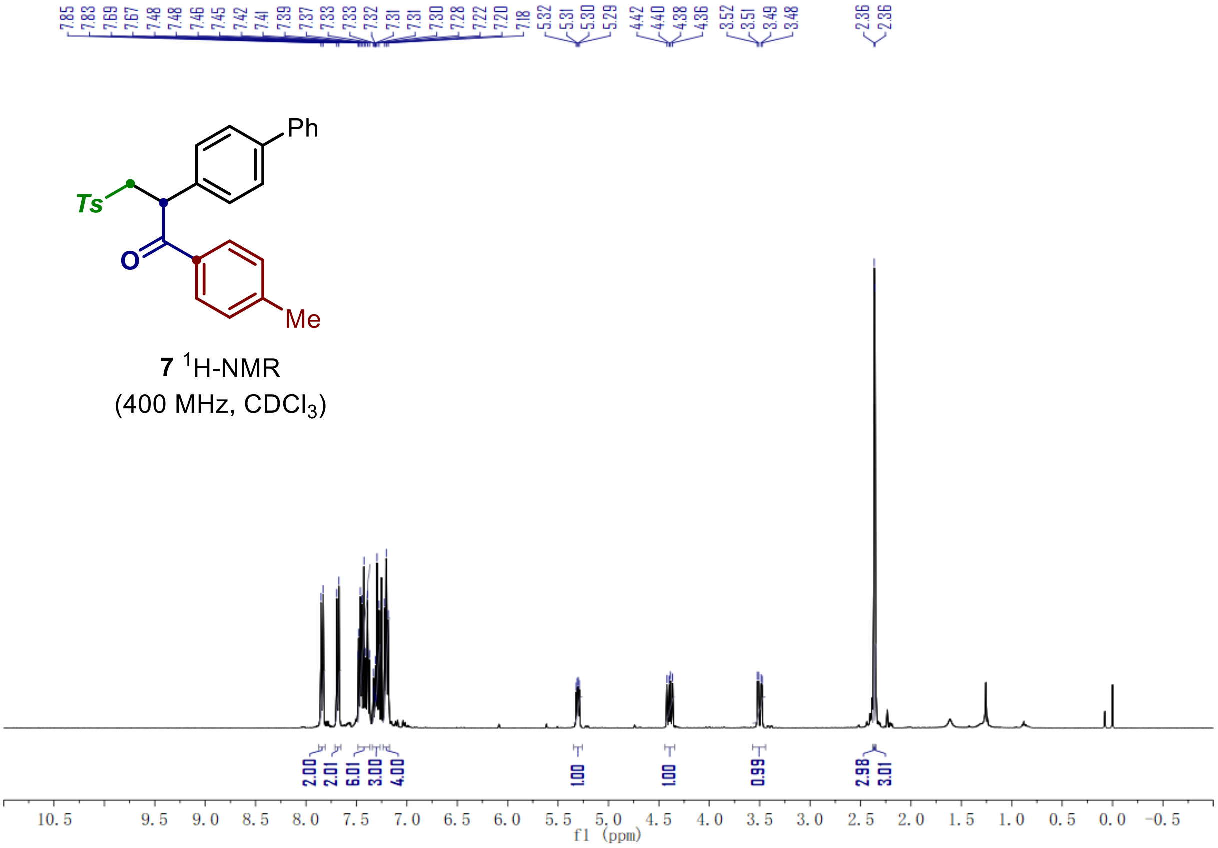
Ph
Ts
O
Me
7 1H-NMR
(400 MHz, CDCl3)
f1 (ppm)

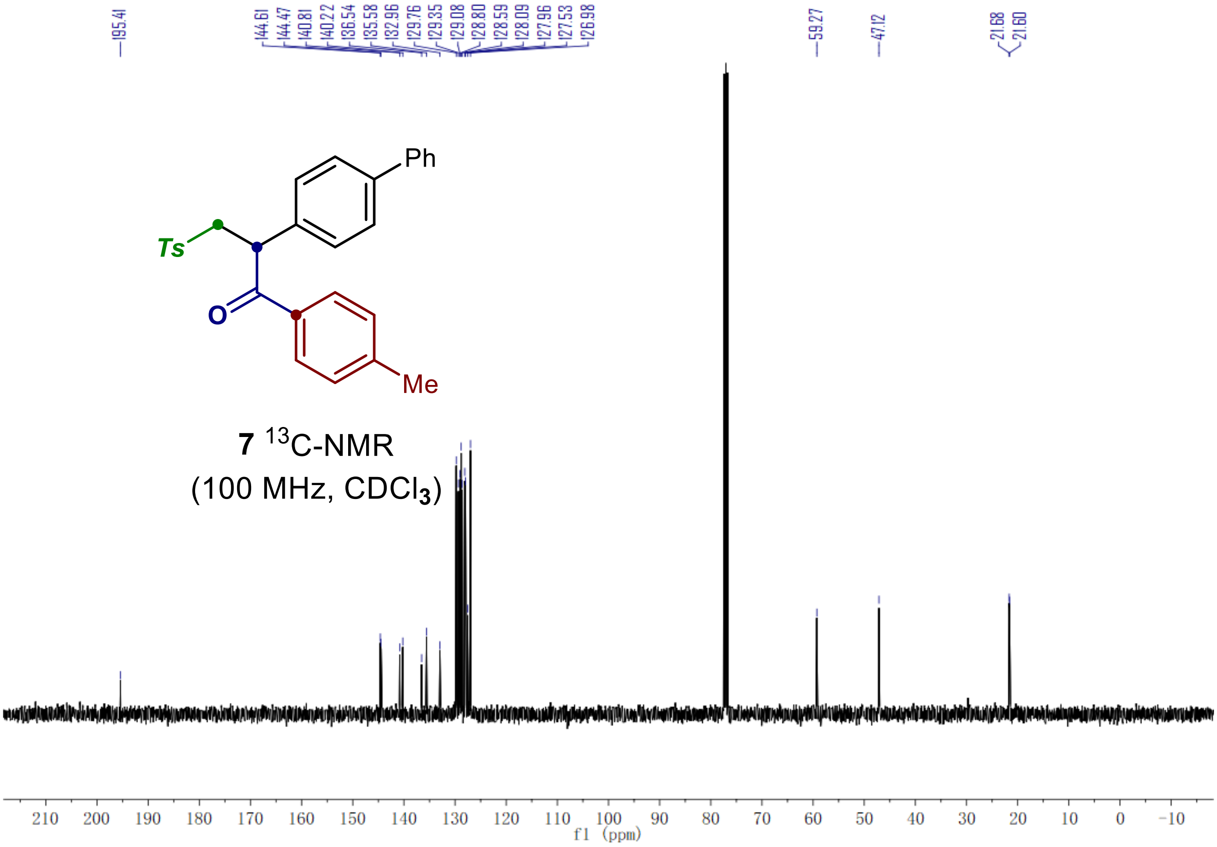

195.41
144.61
144.47
140.81
140.22
136.54
135.58
132.96
129.76
129.35
129.08
128.80
128.59
128.09
127.96
127.53
126.98
59.27
47.12
21.68
21.60
Ph
Ts
O
Me
7 13C-NMR
(100 MHz, CDCl3)
f1 (ppm)

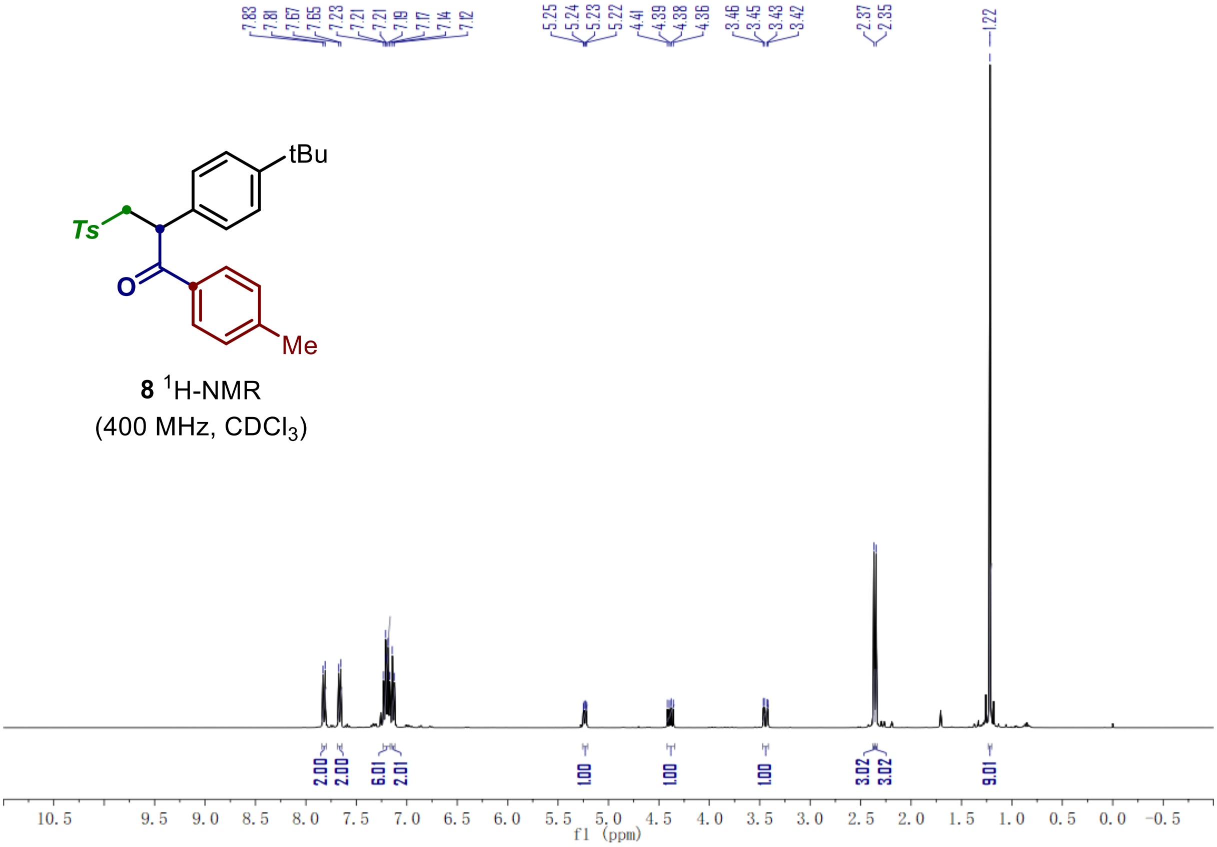

7.83
7.81
7.67
7.65
7.23
7.21
7.21
7.19
7.17
7.14
7.12
5.25
5.24
5.23
5.22
4.41
4.39
4.38
4.36
3.46
3.45
3.43
3.42
2.37
2.35
1.22
tBu
Ts
O
Me
8 1H-NMR
(400 MHz, CDCl3)
2.00
2.00
6.01
2.01
1.00
1.00
1.00
3.02
3.02
9.01
f1 (ppm)

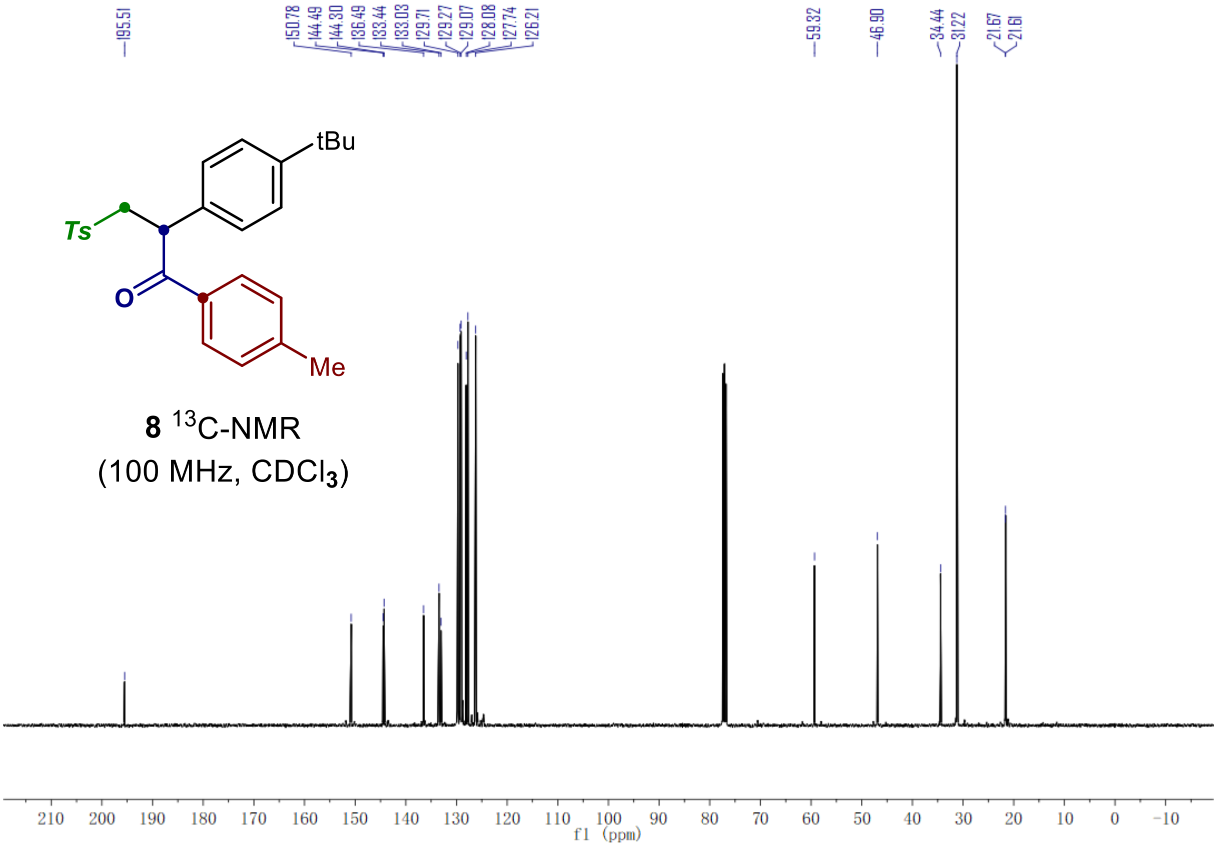
195.51
150.78
144.49
144.30
136.49
133.44
133.03
129.71
129.27
129.07
128.08
127.74
126.21
59.32
46.90
34.44
31.22
21.67
21.61
tBu
Ts
O
Me
8 ¹³C-NMR
(100 MHz, CDCl₃)
210 200 190 180 170 160 150 140 130 120 110 100 90 80 70 60 50 40 30 20 10 0 -10
f1 (ppm)

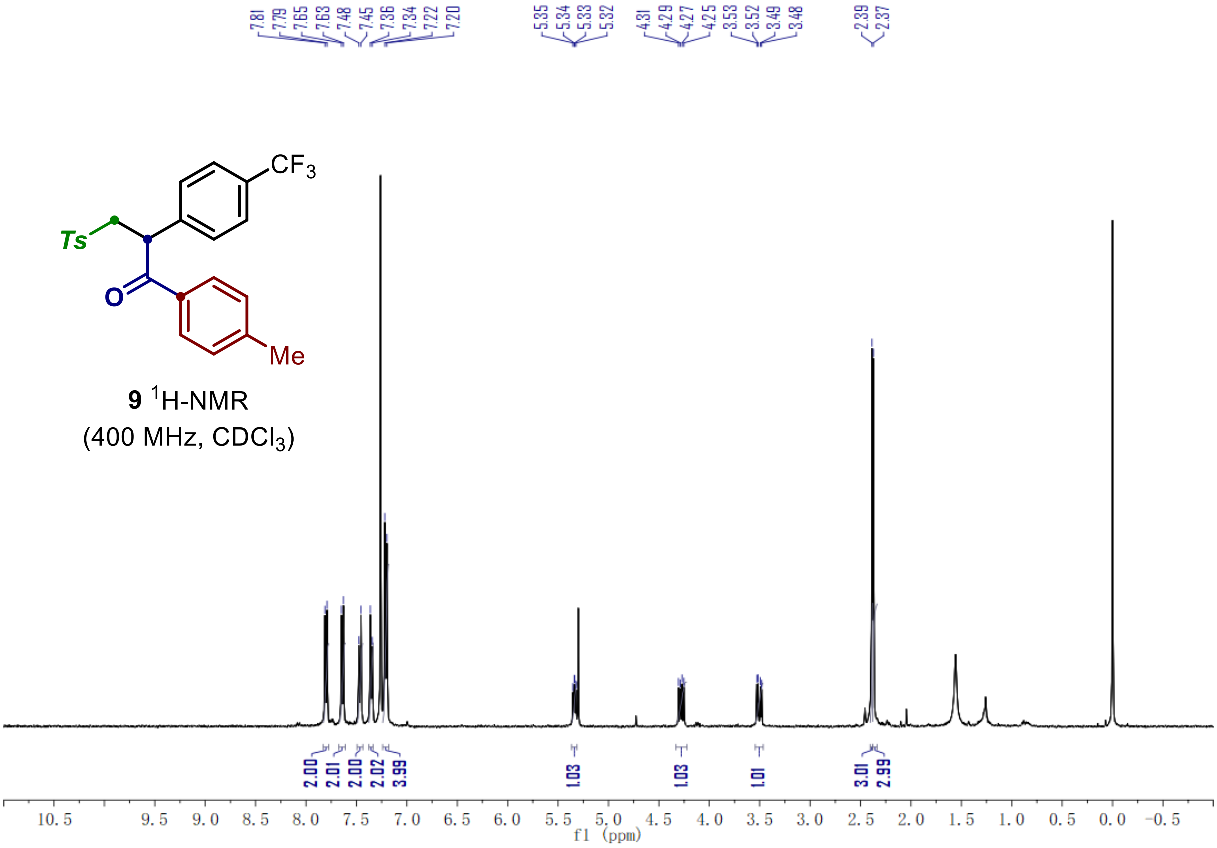
7.81
7.79
7.65
7.63
7.48
7.45
7.36
7.34
7.22
7.20
5.35
5.34
5.33
5.32
4.31
4.29
4.27
4.25
3.53
3.52
3.49
3.48
2.39
2.37
CF₃
Ts
O
Me
9 ¹H-NMR
(400 MHz, CDCl₃)
2.00
2.01
2.00
2.02
3.99
1.03
1.03
1.01
3.01
2.99
10.5 9.5 9.0 8.5 8.0 7.5 7.0 6.5 6.0 5.5 5.0 4.5 4.0 3.5 3.0 2.5 2.0 1.5 1.0 0.5 0.0 -0.5
f1 (ppm)

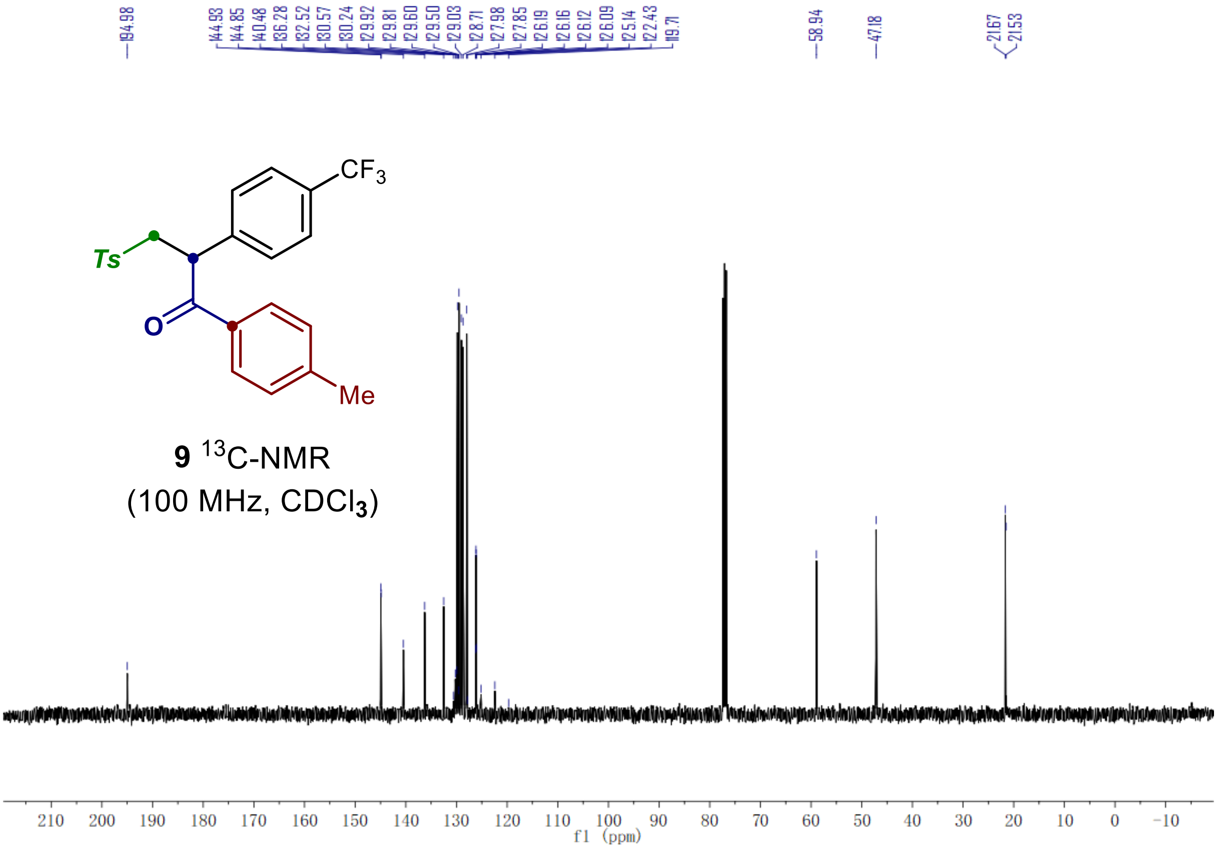
194.98
144.93
144.85
140.48
136.28
132.52
130.57
130.24
129.92
129.81
129.60
129.50
129.03
128.71
127.98
127.85
126.19
126.16
126.12
126.09
125.14
122.43
119.71
58.94
47.18
21.67
21.53
CF3
Ts
O
Me
9 13C-NMR
(100 MHz, CDCl3)
f1 (ppm)

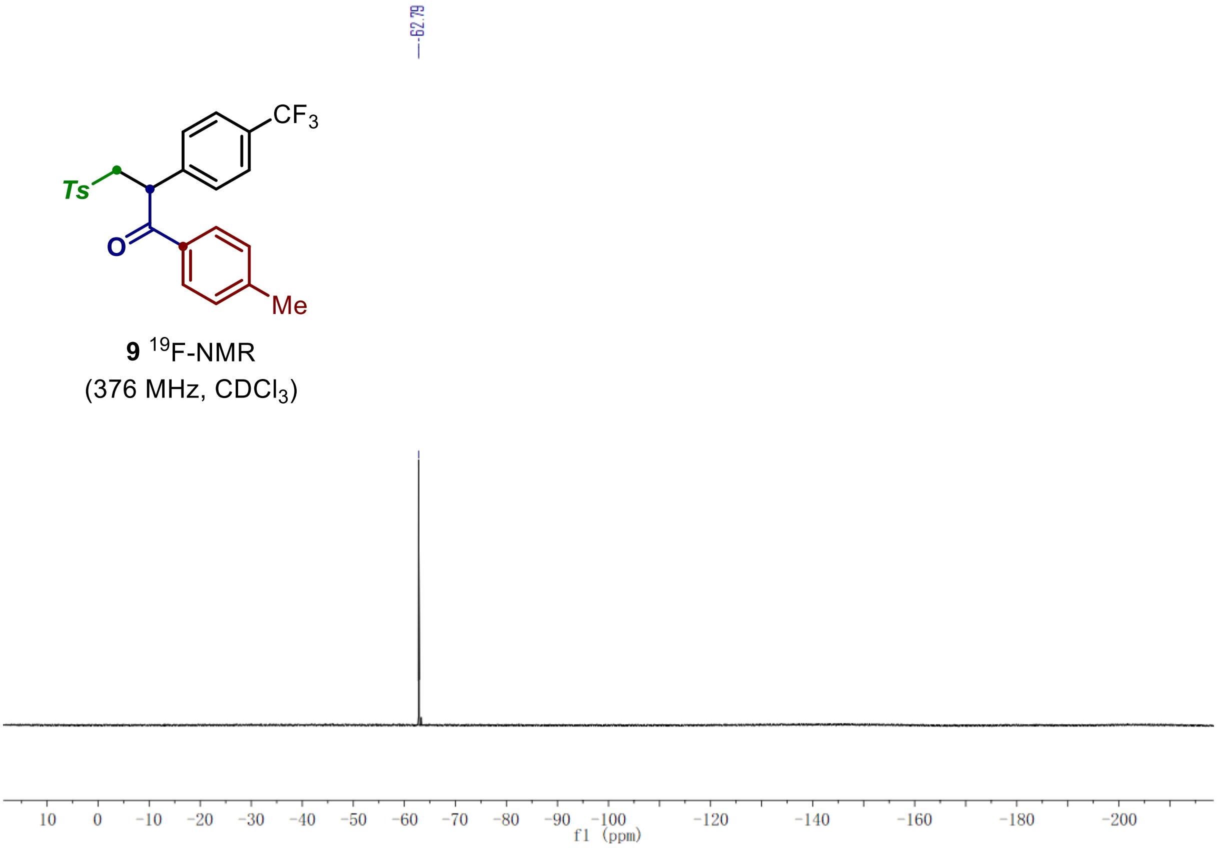
-62.79
CF3
Ts
O
Me
9 19F-NMR
(376 MHz, CDCl3)
f1 (ppm)

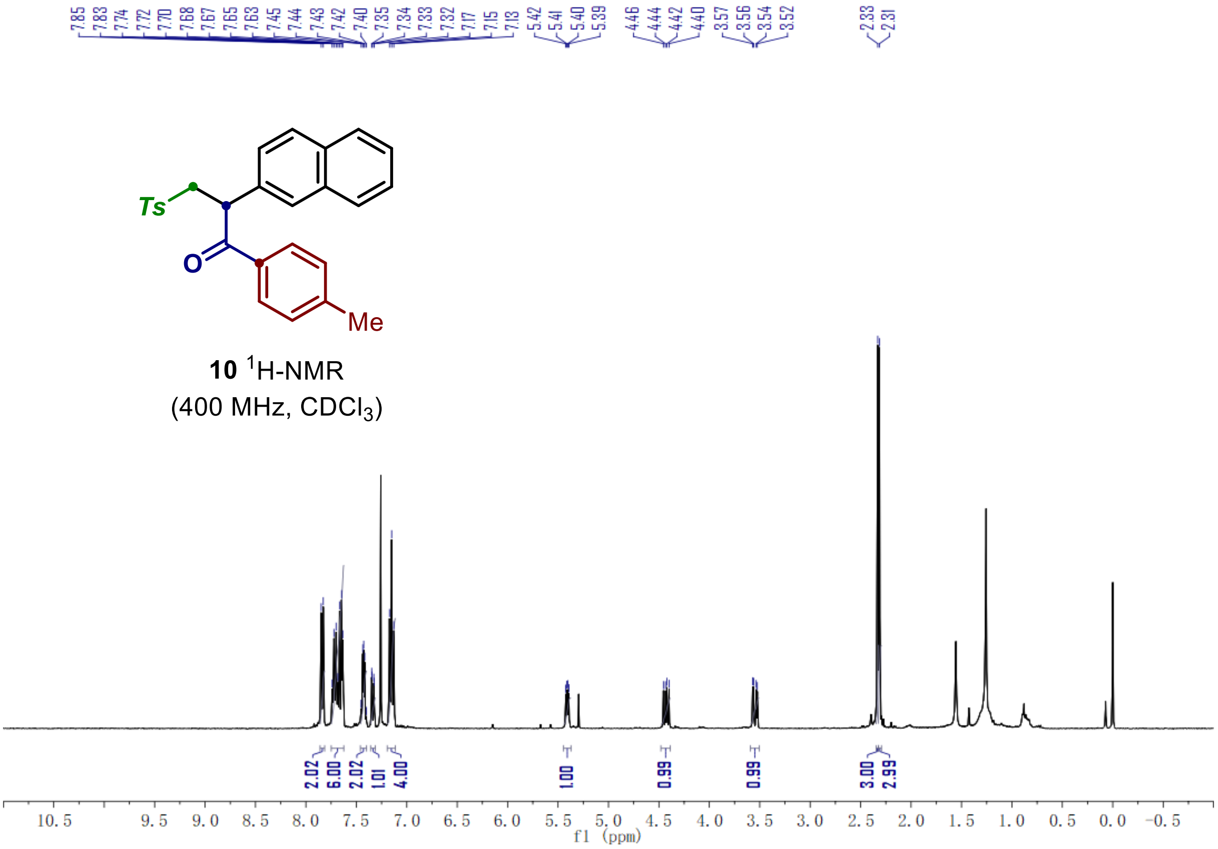

10 ^{1}H-NMR
(400 MHz, $CDCl_3$)

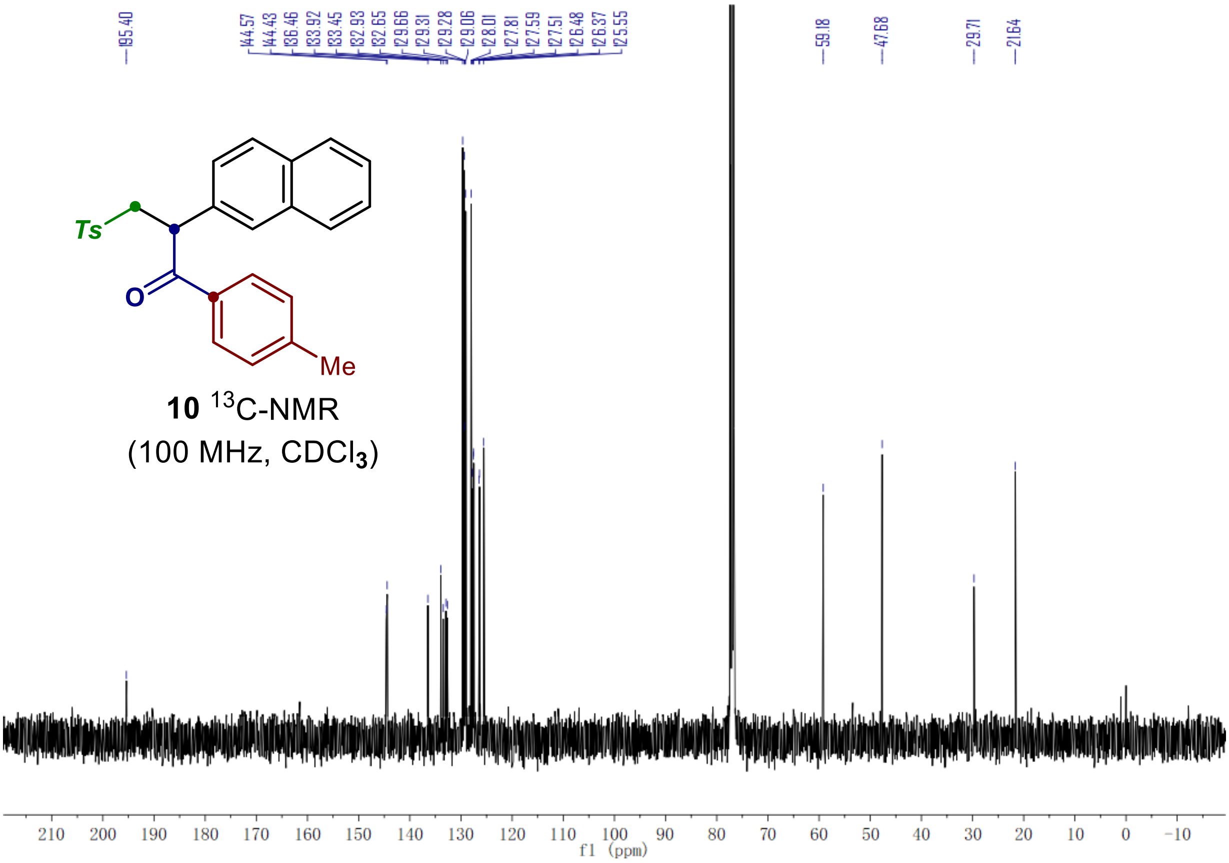

10 ^{13}C-NMR
(100 MHz, $CDCl_3$)

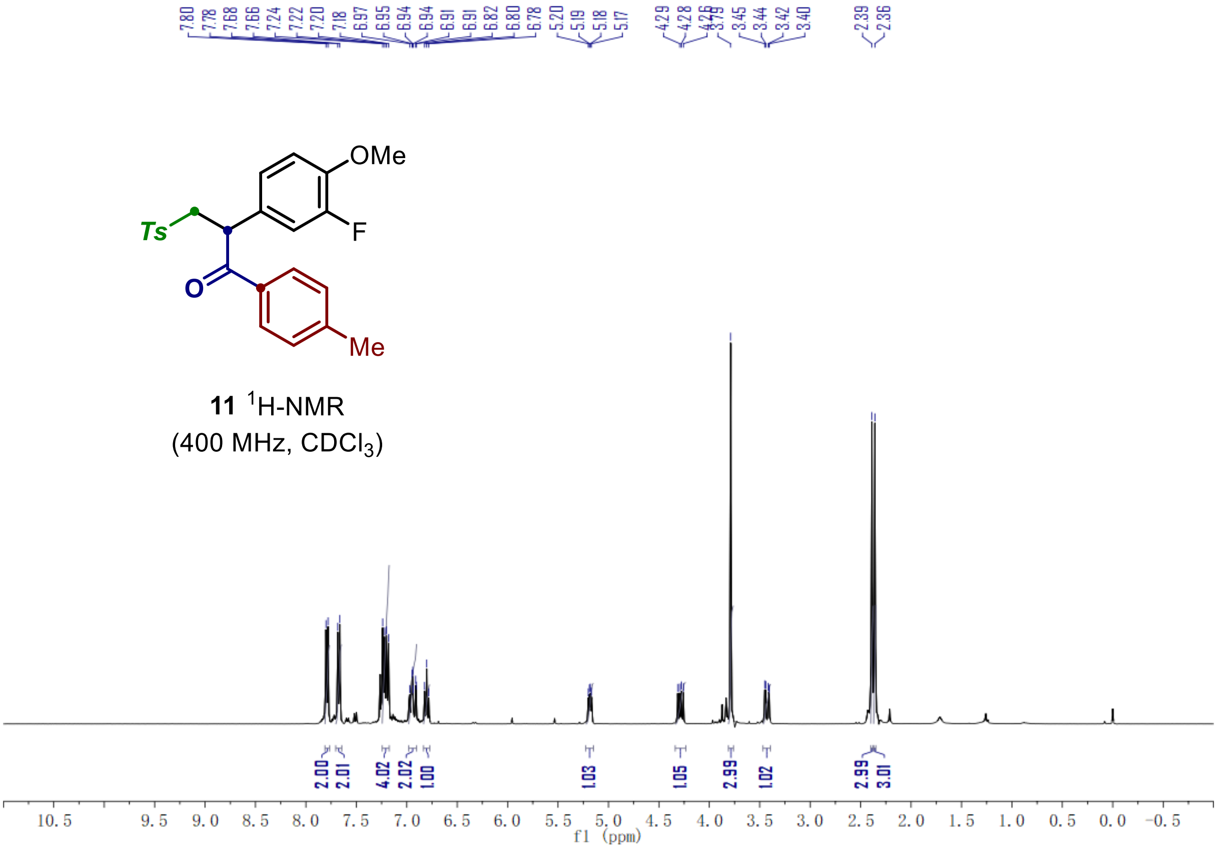

7.80
7.78
7.68
7.66
7.24
7.22
7.20
7.18
6.97
6.95
6.94
6.94
6.91
6.91
6.82
6.80
6.78
5.20
5.19
5.18
5.17
4.29
4.28
4.26
3.79
3.45
3.44
3.42
3.40
2.39
2.36
OMe
F
Ts
O
Me
11 ^{1}H-NMR
(400 MHz, $CDCl_3$)
2.00
2.01
4.02
2.02
1.00
1.03
1.05
2.99
1.02
2.99
3.01
f1 (ppm)

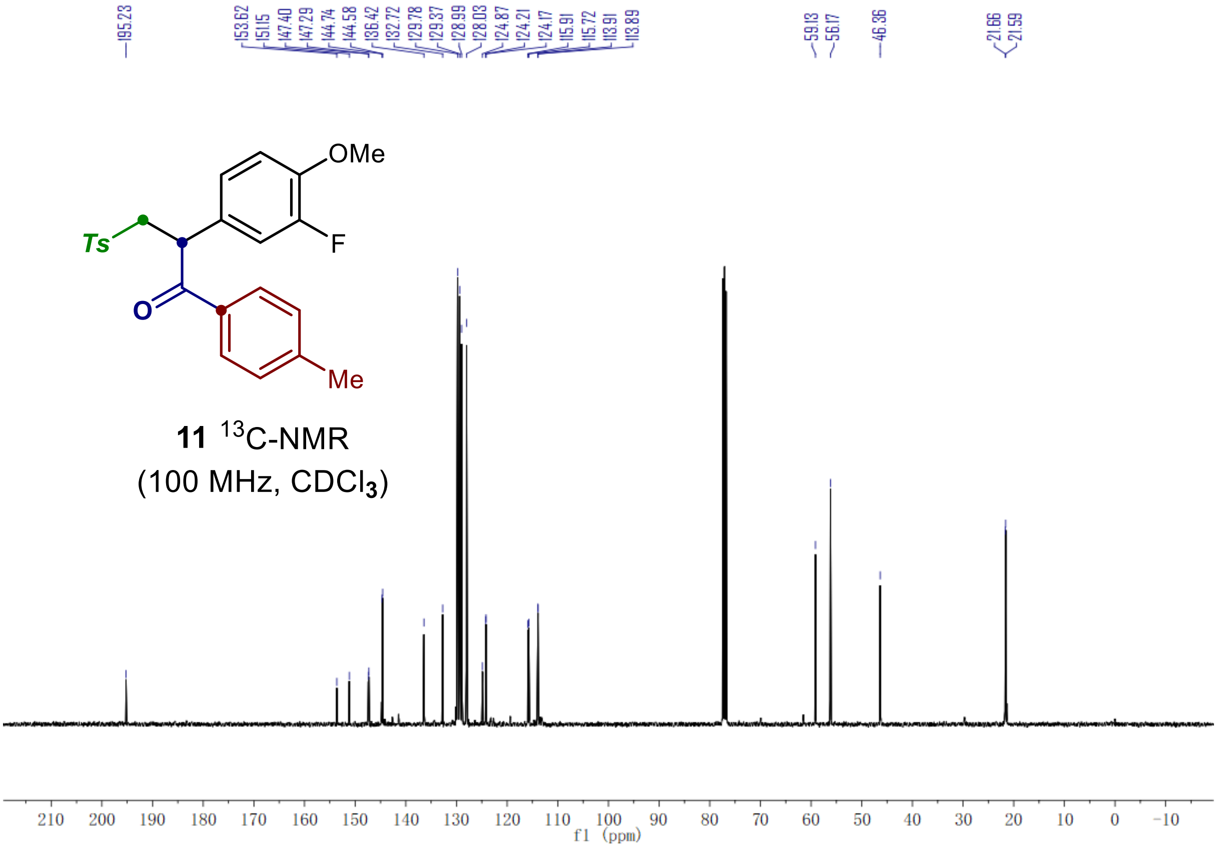

195.23
153.62
151.15
147.40
147.29
144.74
144.58
136.42
132.72
129.78
129.37
128.99
128.03
124.87
124.21
124.17
115.91
115.72
113.91
113.89
59.13
56.17
46.36
21.66
21.59
OMe
F
Ts
O
Me
11 ^{13}C-NMR
(100 MHz, $CDCl_3$)
f1 (ppm)

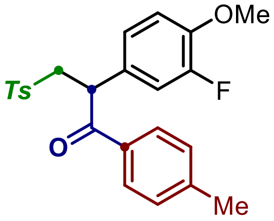

11 ^{19}F-NMR
(376 MHz, $CDCl_3$)

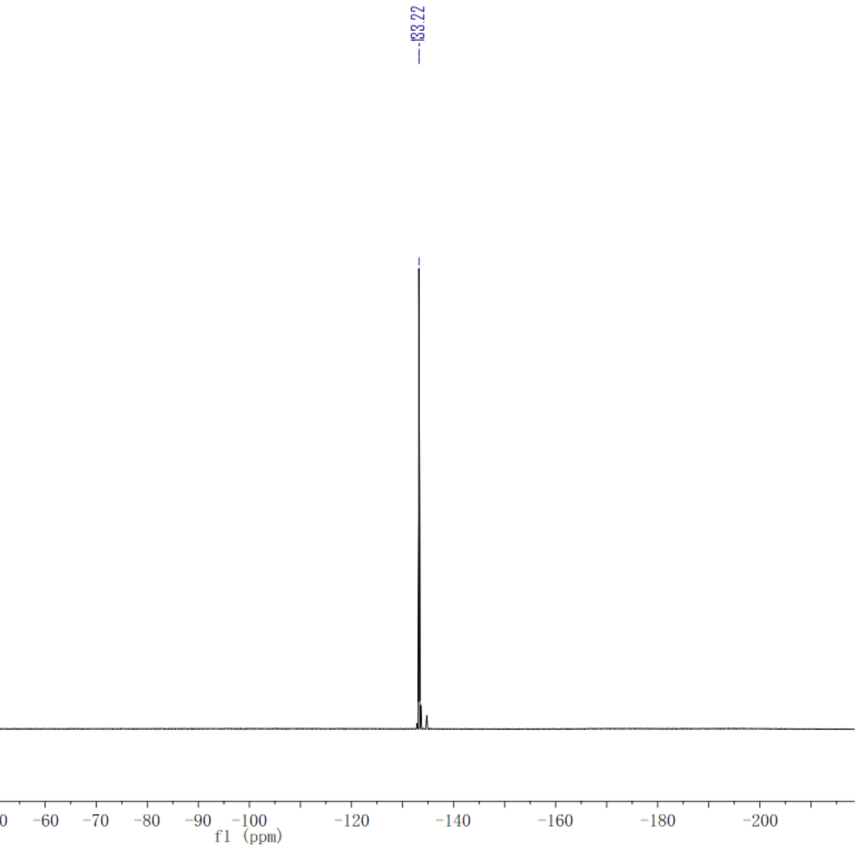

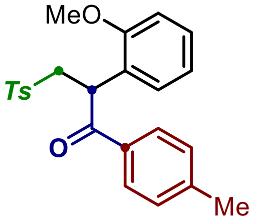

12 ^{1}H-NMR
(400 MHz, $CDCl_3$)

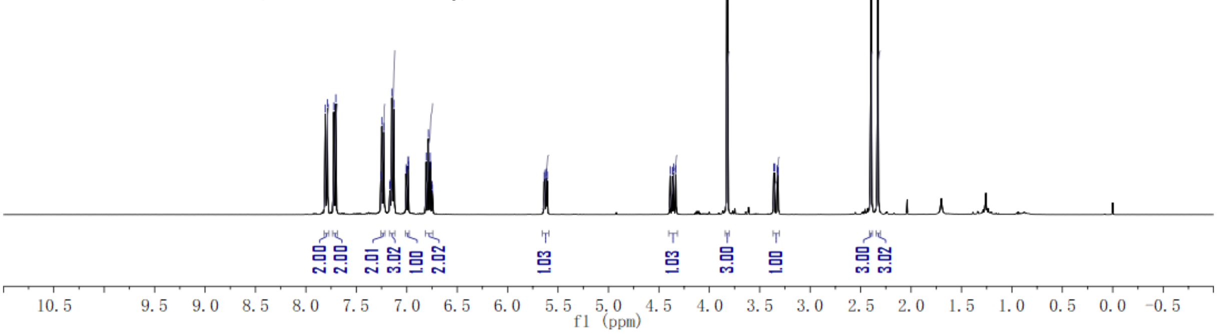

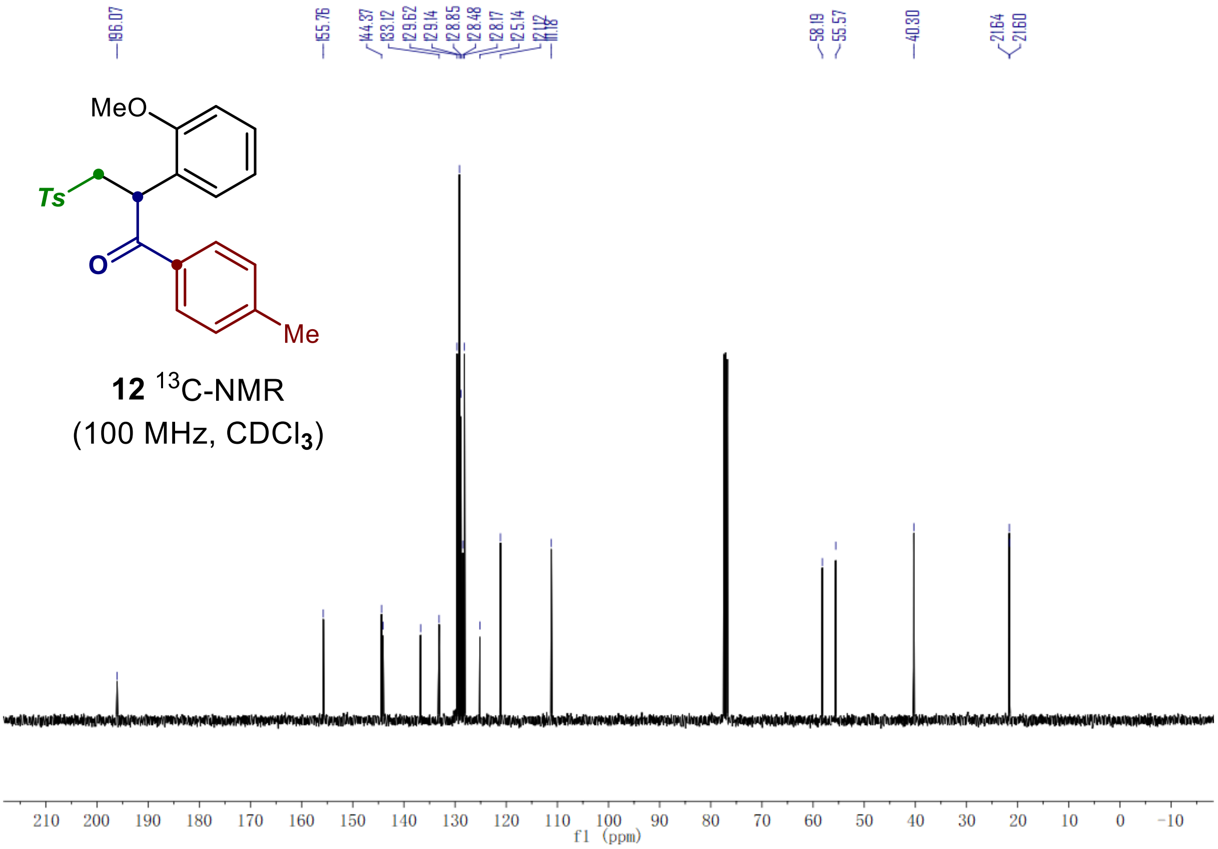
196.07
155.76
144.37
133.12
129.62
129.14
128.85
128.48
128.17
125.14
121.12
111.18
58.19
55.57
40.30
21.64
21.60
MeO
Ts
O
Me
12 ^{13}C-NMR
(100 MHz, $CDCl_3$)
210 200 190 180 170 160 150 140 130 120 110 100 90 80 70 60 50 40 30 20 10 0 -10
f1 (ppm)

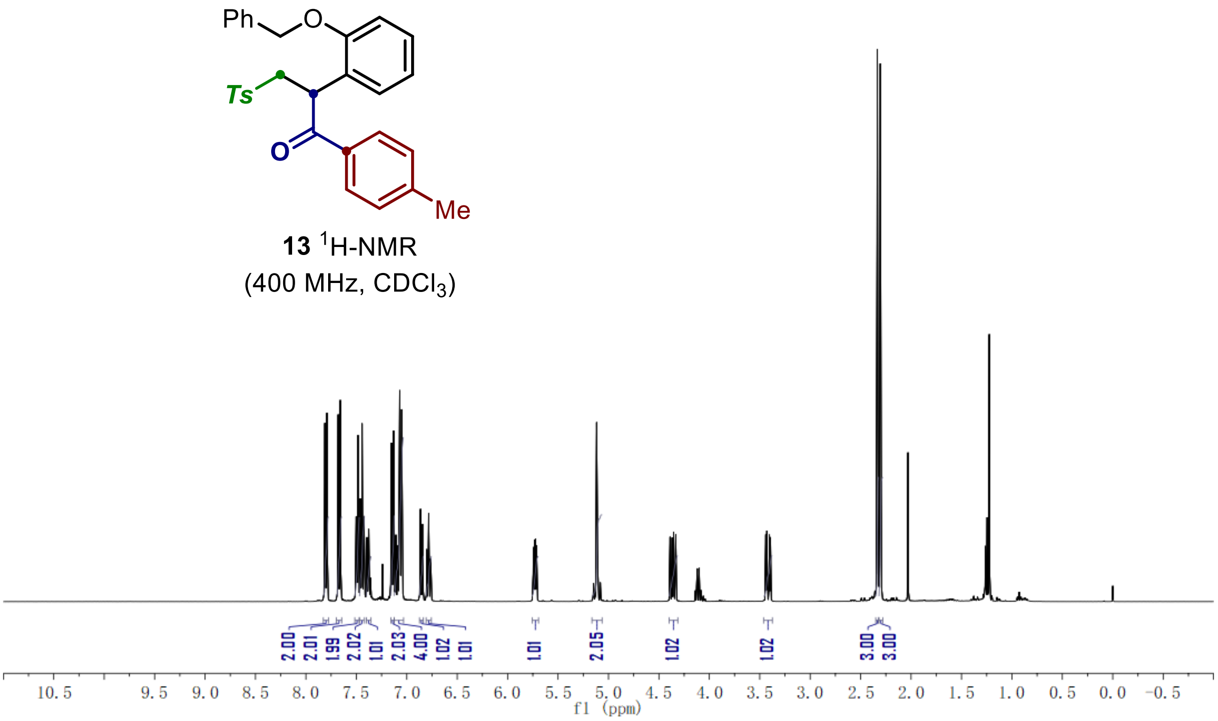
Ph
O
Ts
O
Me
13 ^{1}H-NMR
(400 MHz, $CDCl_3$)
2.00
2.01
1.99
2.02
1.01
2.03
4.00
1.02
1.01
1.01
2.05
1.02
1.02
3.00
3.00
10.5 9.5 9.0 8.5 8.0 7.5 7.0 6.5 6.0 5.5 5.0 4.5 4.0 3.5 3.0 2.5 2.0 1.5 1.0 0.5 0.0 -0.5
f1 (ppm)

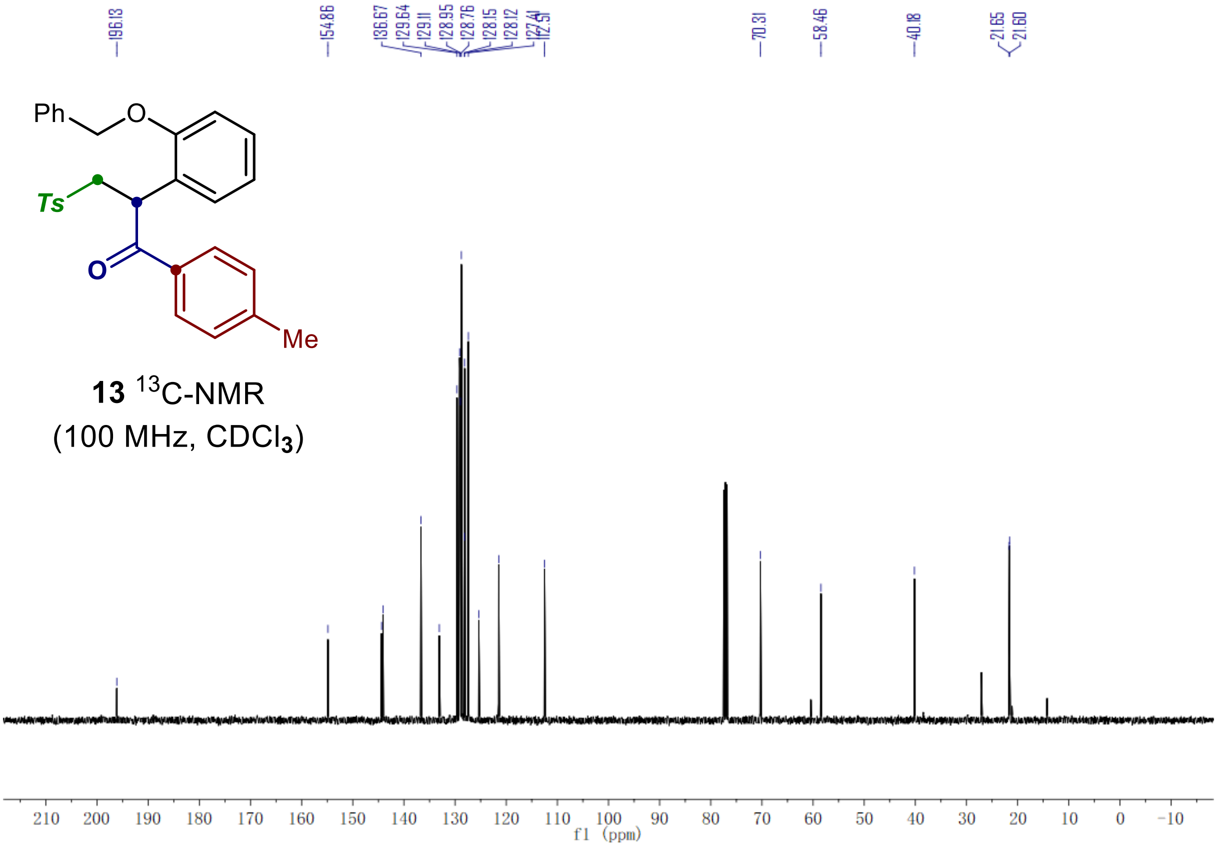
196.13
154.86
136.67
129.64
129.11
128.95
128.76
128.15
128.12
127.41
112.51
70.31
58.46
40.18
21.65
21.60
Ph
O
Ts
O
Me
13 13C-NMR
(100 MHz, CDCl3)
f1 (ppm)

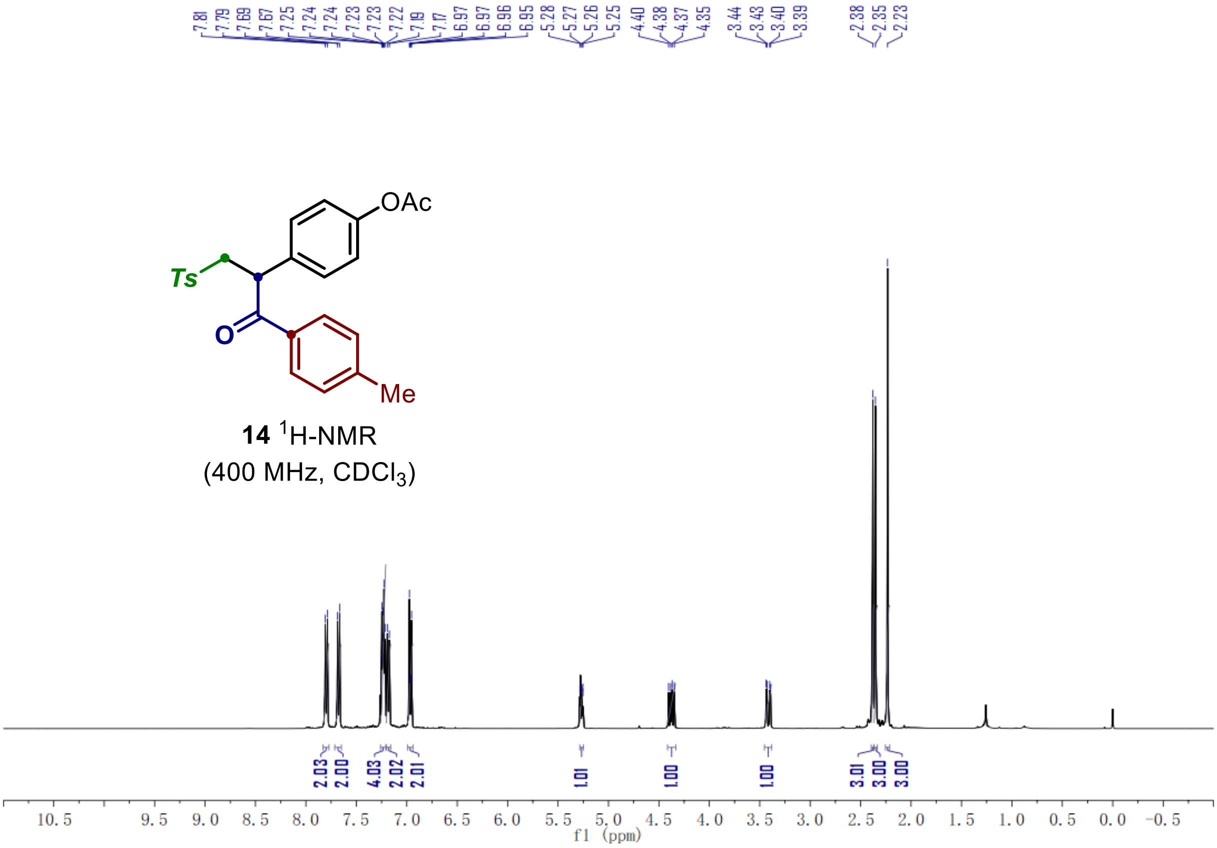
7.81
7.79
7.69
7.67
7.25
7.24
7.24
7.23
7.23
7.22
7.19
7.17
6.97
6.97
6.96
6.95
5.28
5.27
5.26
5.25
4.40
4.38
4.37
4.35
3.44
3.43
3.40
3.39
2.38
2.35
2.23
OAc
Ts
O
Me
14 1H-NMR
(400 MHz, CDCl3)
2.03
2.00
4.03
2.02
2.01
1.01
1.00
1.00
3.01
3.00
3.00
f1 (ppm)

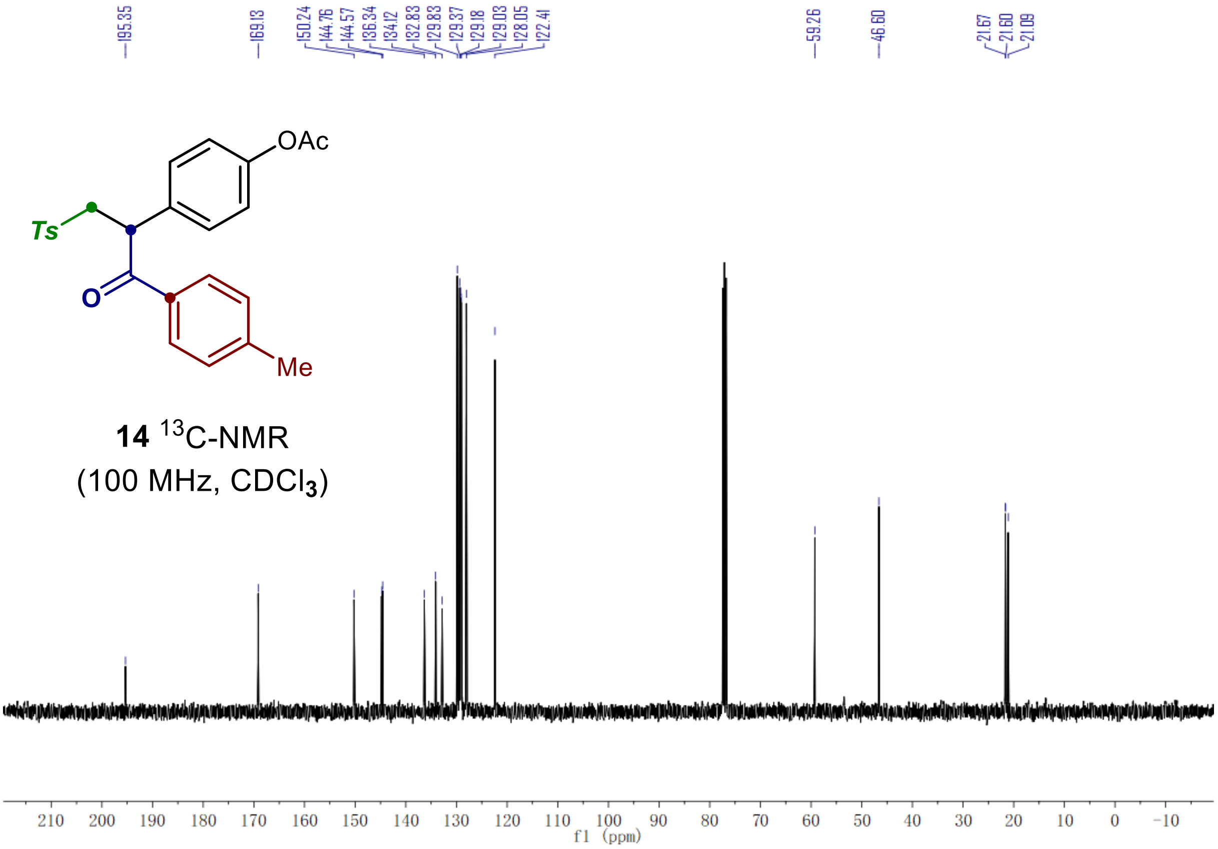
195.35
169.13
150.24
144.76
144.57
136.34
134.12
132.83
129.83
129.37
129.18
129.03
128.05
122.41
59.26
46.60
21.67
21.60
21.09
OAc
Ts
O
Me
14 ^{13}C-NMR
(100 MHz, $CDCl_3$)
210 200 190 180 170 160 150 140 130 120 110 100 90 80 70 60 50 40 30 20 10 0 -10
f1 (ppm)

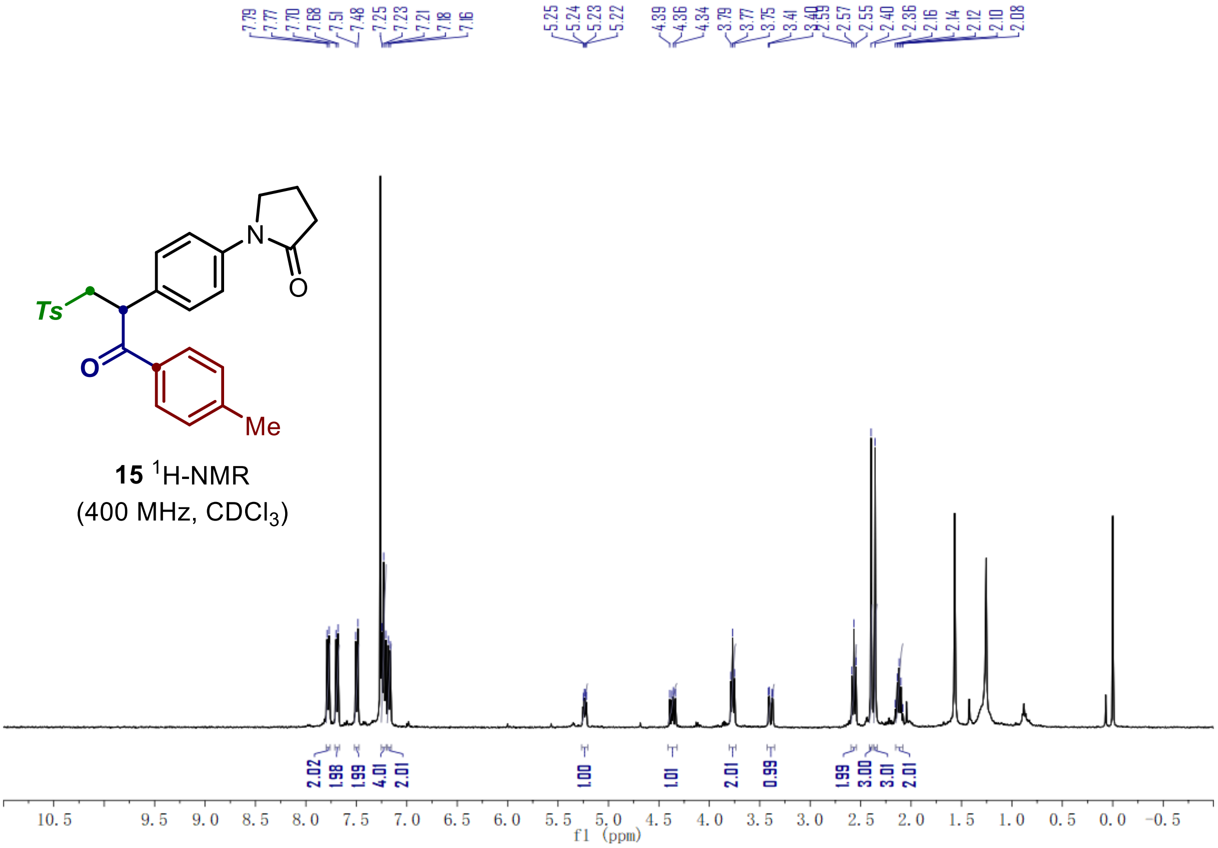
7.79
7.77
7.70
7.68
7.51
7.48
7.25
7.23
7.21
7.18
7.16
5.25
5.24
5.23
5.22
4.39
4.36
4.34
3.79
3.77
3.75
3.41
3.40
2.59
2.57
2.55
2.40
2.36
2.16
2.14
2.12
2.10
2.08
N
O
Ts
O
Me
15 ^{1}H-NMR
(400 MHz, $CDCl_3$)
2.02
1.98
1.99
4.01
2.01
1.00
1.01
2.01
0.99
1.99
3.00
3.01
2.01
10.5 9.5 9.0 8.5 8.0 7.5 7.0 6.5 6.0 5.5 5.0 4.5 4.0 3.5 3.0 2.5 2.0 1.5 1.0 0.5 0.0 -0.5
f1 (ppm)

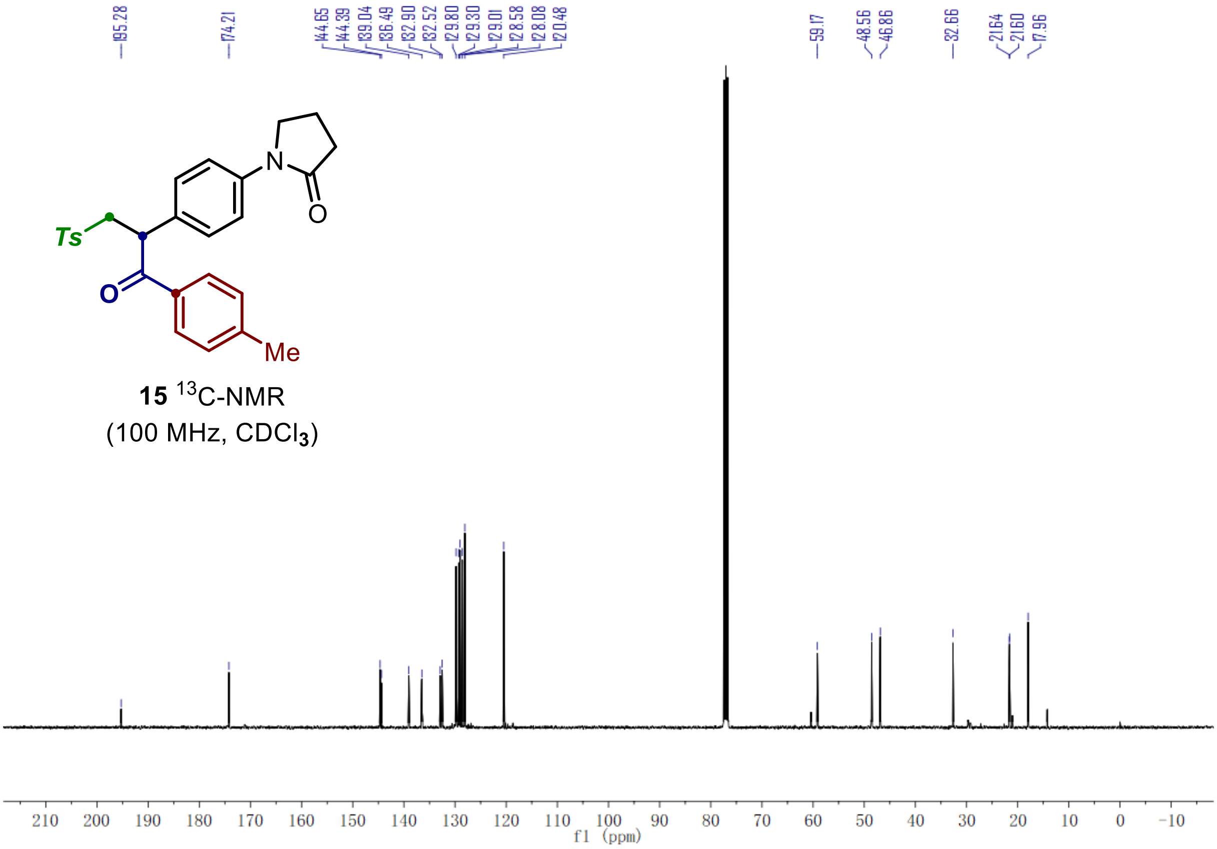
195.28
174.21
144.65
144.39
139.04
136.49
132.90
132.52
129.80
129.30
129.01
128.58
128.08
120.48
59.17
48.56
46.86
32.66
21.64
21.60
17.96
Ts
N
O
O
Me
15 13C-NMR
(100 MHz, CDCl3)
210 200 190 180 170 160 150 140 130 120 110 100 90 80 70 60 50 40 30 20 10 0 -10
f1 (ppm)

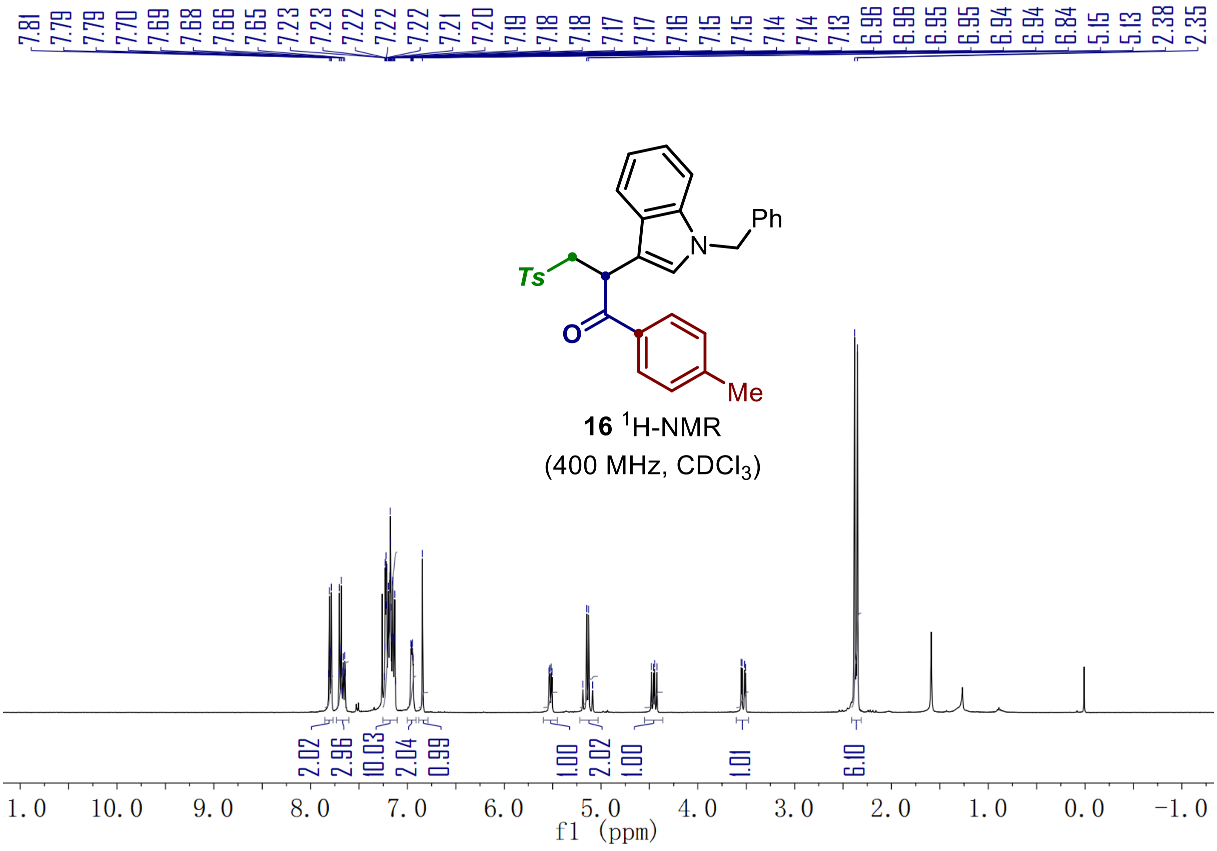
7.81
7.79
7.79
7.70
7.69
7.68
7.66
7.65
7.23
7.23
7.22
7.22
7.22
7.21
7.20
7.19
7.18
7.18
7.17
7.17
7.16
7.15
7.15
7.14
7.14
7.13
6.96
6.96
6.95
6.95
6.94
6.94
6.84
5.15
5.13
2.38
2.35
Ph
N
Ts
O
Me
16 1H-NMR
(400 MHz, CDCl3)
2.02
2.96
10.03
2.04
0.99
1.00
2.02
1.00
1.01
6.10
1.0 10.0 9.0 8.0 7.0 6.0 5.0 4.0 3.0 2.0 1.0 0.0 -1.0
f1 (ppm)

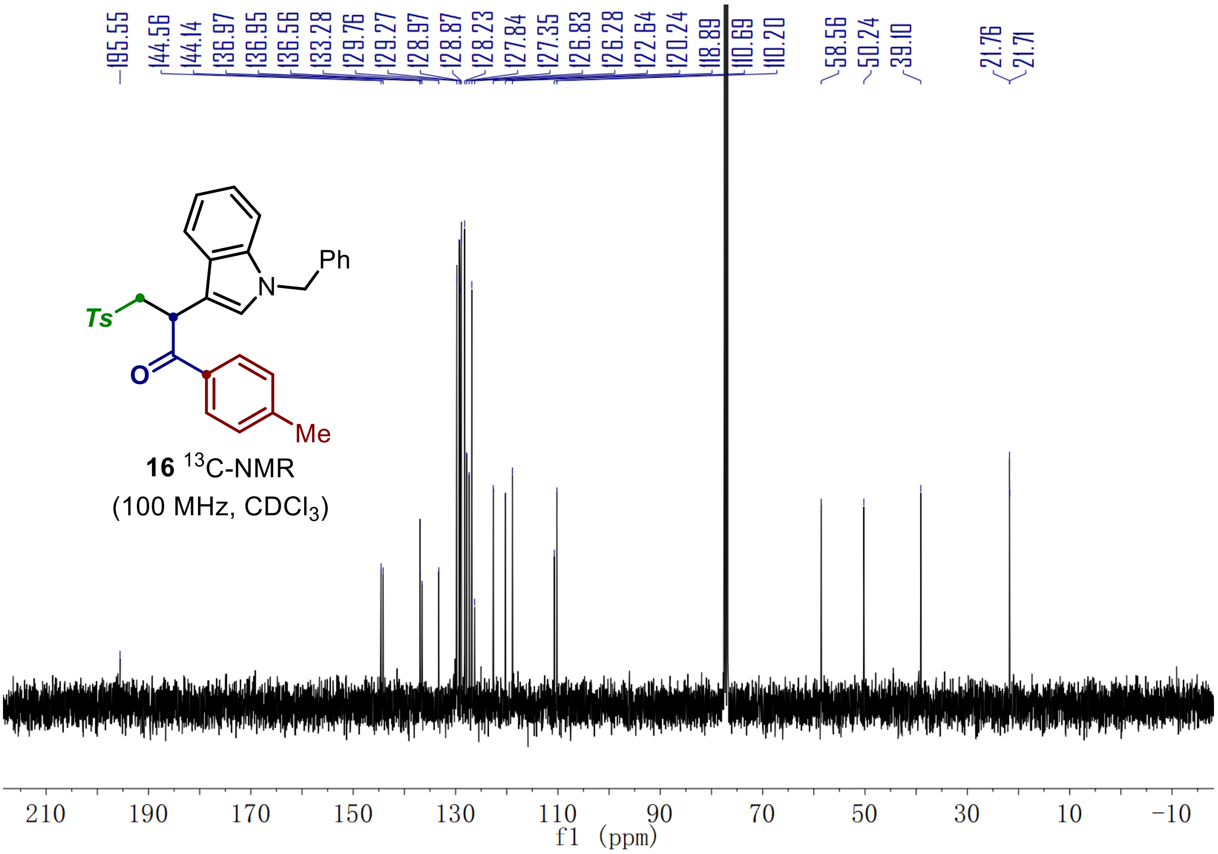
195.55
144.56
144.14
136.97
136.95
136.56
133.28
129.76
129.27
128.97
128.87
128.23
127.84
127.35
126.83
126.28
122.64
120.24
118.89
110.69
110.20
58.56
50.24
39.10
21.76
21.71
Ts
O
N
Ph
Me
16 13C-NMR
(100 MHz, CDCl3)
210 190 170 150 130 110 90 70 50 30 10 -10
f1 (ppm)

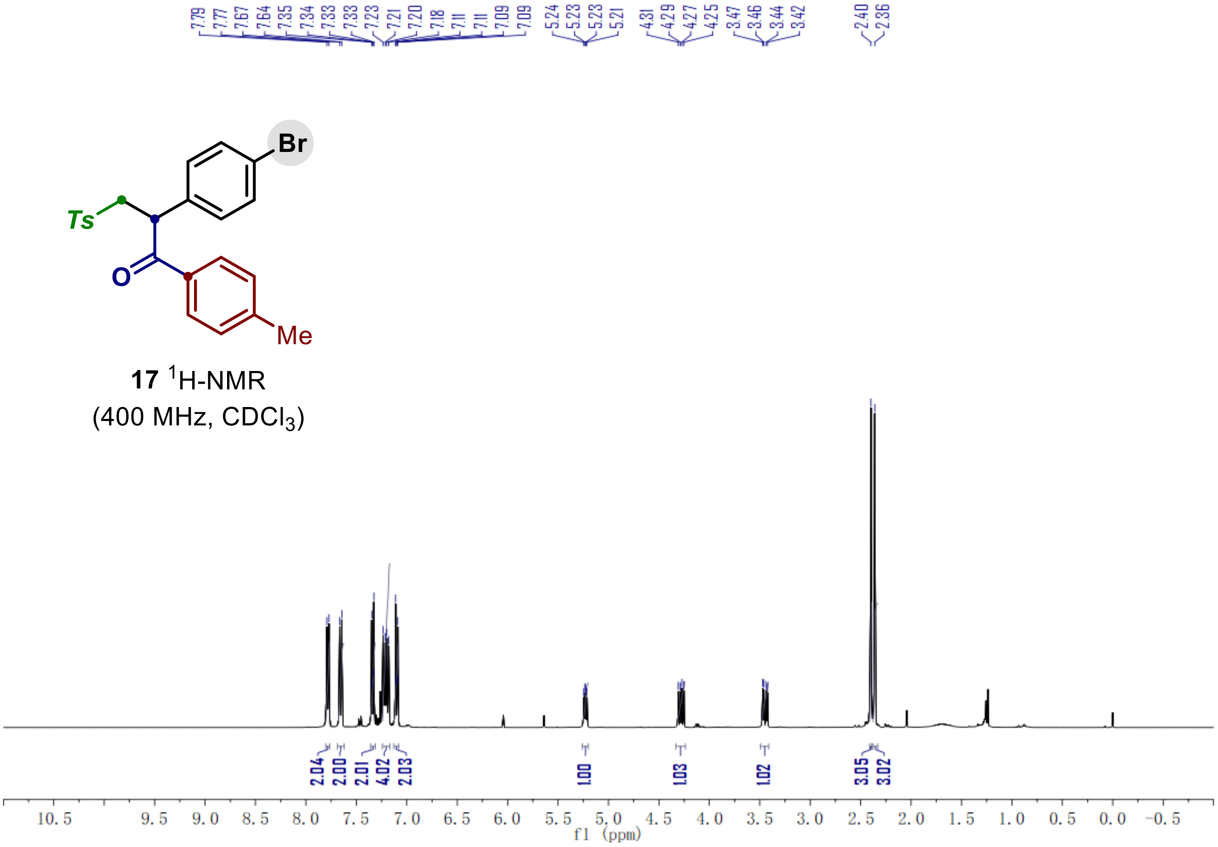
7.79
7.77
7.67
7.64
7.35
7.34
7.33
7.33
7.23
7.21
7.20
7.18
7.11
7.11
7.09
7.09
5.24
5.23
5.23
5.21
4.31
4.29
4.27
4.25
3.47
3.46
3.44
3.42
2.40
2.36
Br
Ts
O
Me
17 1H-NMR
(400 MHz, CDCl3)
2.04
2.00
2.01
4.02
2.03
1.00
1.03
1.02
3.05
3.02
10.5 9.5 9.0 8.5 8.0 7.5 7.0 6.5 6.0 5.5 5.0 4.5 4.0 3.5 3.0 2.5 2.0 1.5 1.0 0.5 0.0 -0.5
f1 (ppm)

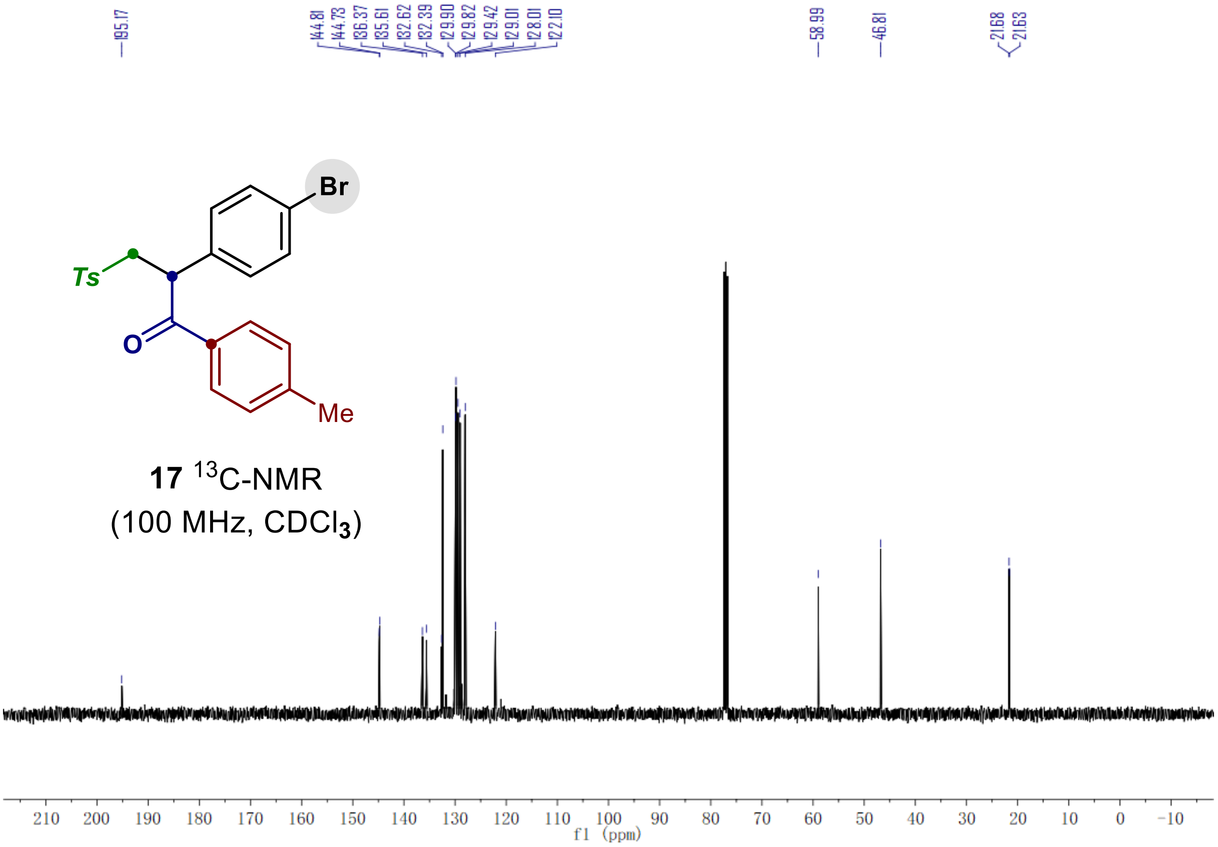

17 ^{13}C-NMR
(100 MHz, $CDCl_3$)

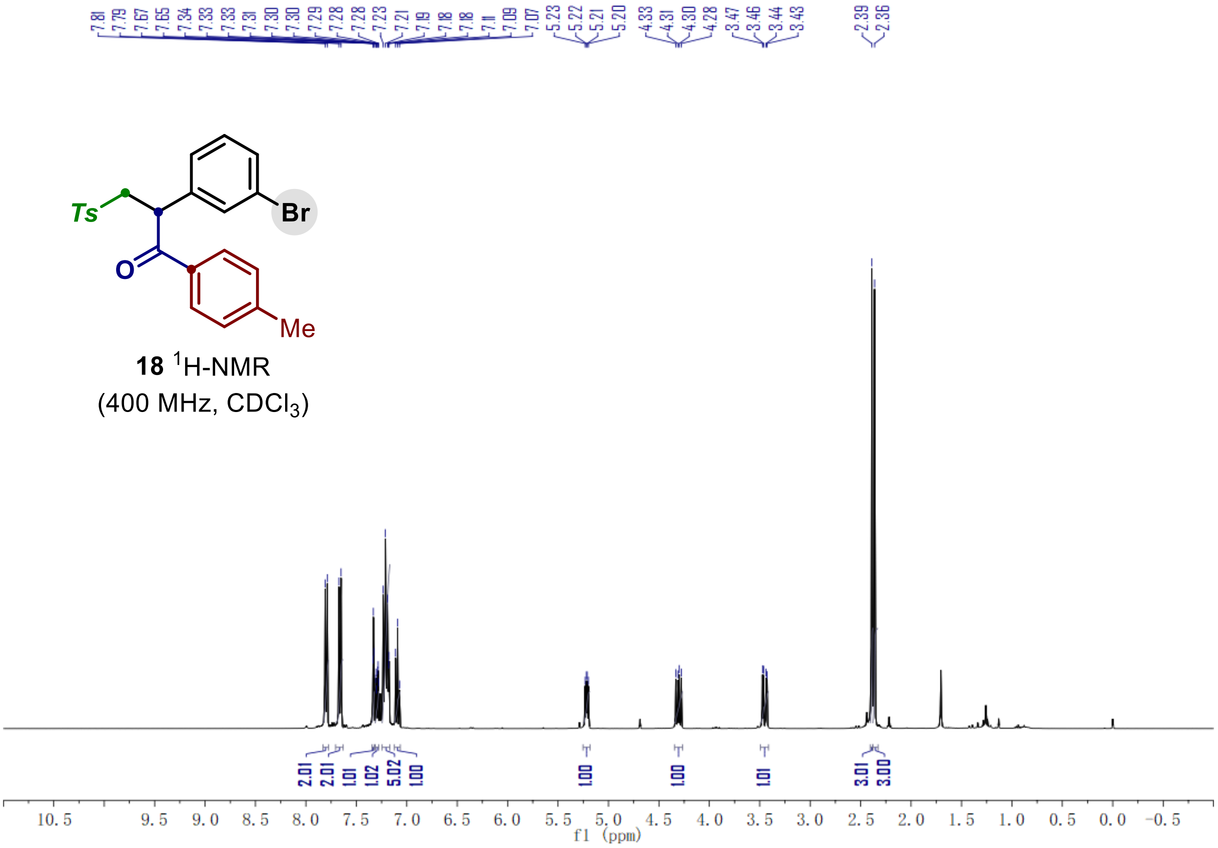

18 ^{1}H-NMR
(400 MHz, $CDCl_3$)

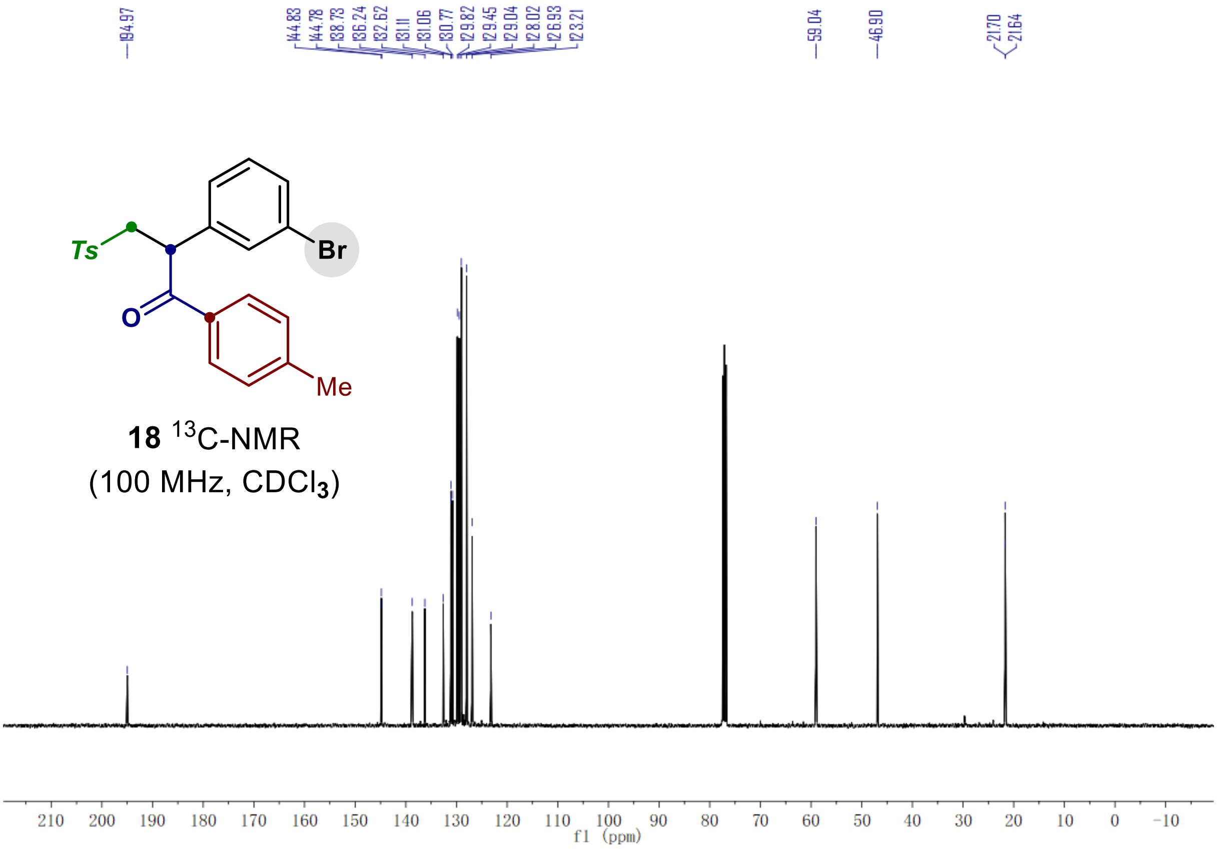
194.97
144.83
144.78
138.73
136.24
132.62
131.11
131.06
130.77
129.82
129.45
129.04
128.02
126.93
123.21
59.04
46.90
21.70
21.64
Ts
Br
O
Me
18 13C-NMR
(100 MHz, CDCl3)
210 200 190 180 170 160 150 140 130 120 110 100 90 80 70 60 50 40 30 20 10 0 -10
f1 (ppm)

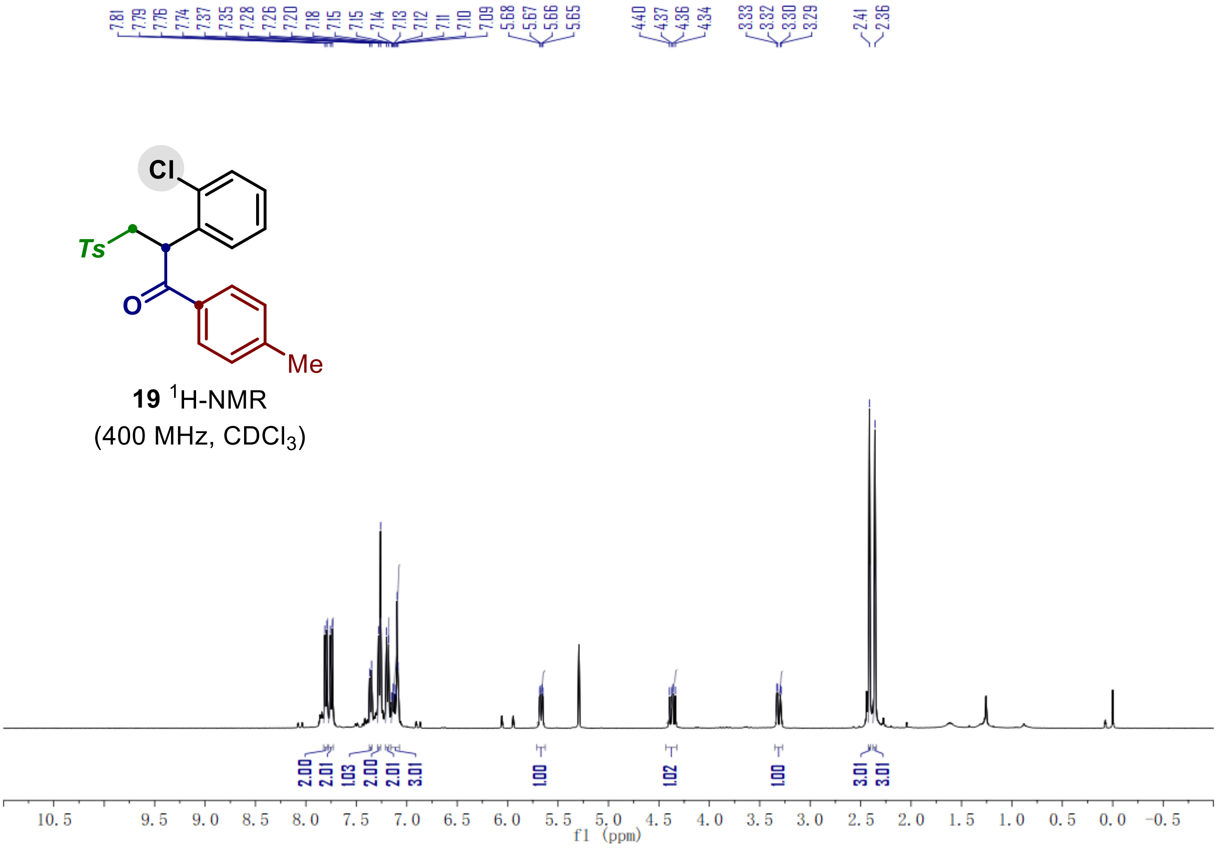
7.81
7.79
7.76
7.74
7.37
7.35
7.28
7.26
7.20
7.18
7.15
7.15
7.14
7.13
7.12
7.11
7.10
7.09
5.68
5.67
5.66
5.65
4.40
4.37
4.36
4.34
3.33
3.32
3.30
3.29
2.41
2.36
Cl
Ts
O
Me
19 1H-NMR
(400 MHz, CDCl3)
2.00
2.01
1.03
2.00
2.01
3.01
1.00
1.02
1.00
3.01
3.01
10.5 9.5 9.0 8.5 8.0 7.5 7.0 6.5 6.0 5.5 5.0 4.5 4.0 3.5 3.0 2.5 2.0 1.5 1.0 0.5 0.0 -0.5
f1 (ppm)

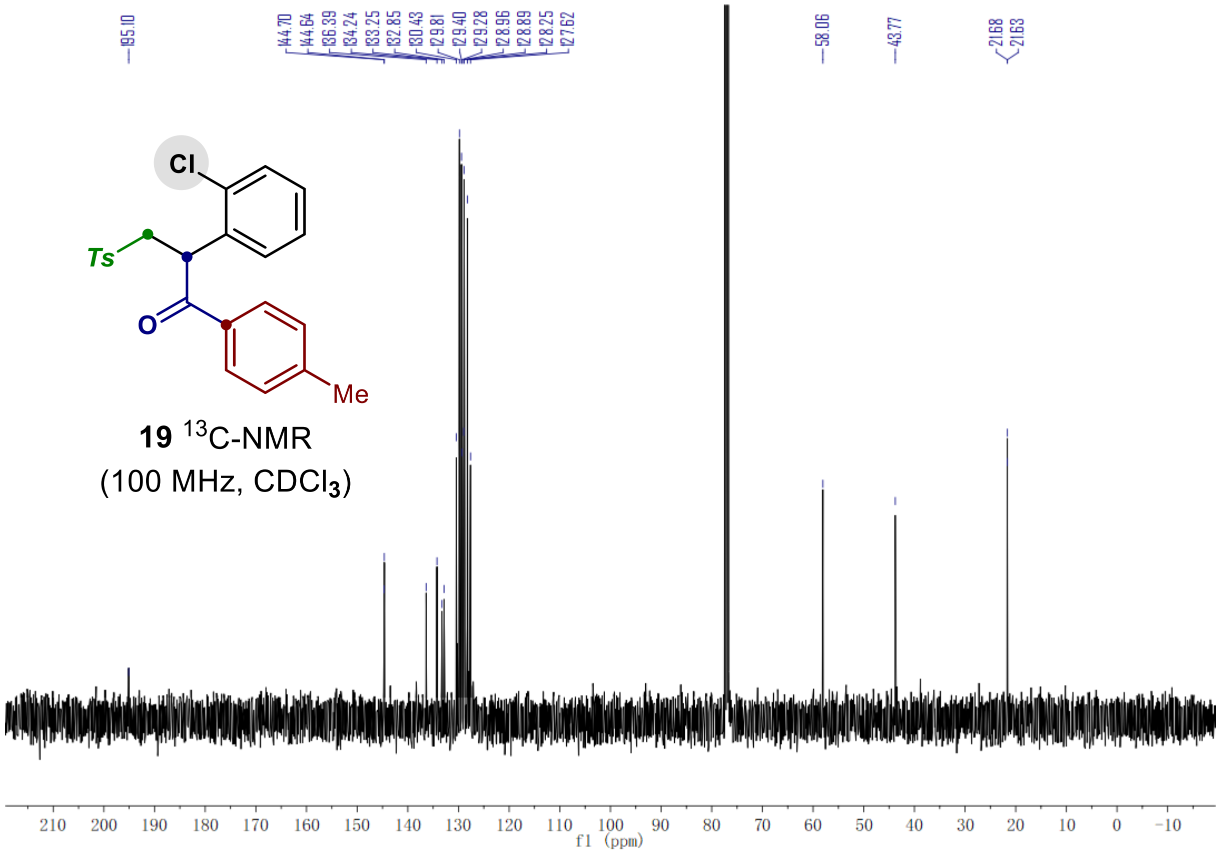
Cl
Ts
O
Me
19 13C-NMR
(100 MHz, CDCl3)
195.10
144.70
144.64
136.39
134.24
133.25
132.85
130.43
129.81
129.40
129.28
128.96
128.89
128.25
127.62
58.06
43.77
21.68
21.63
f1 (ppm)

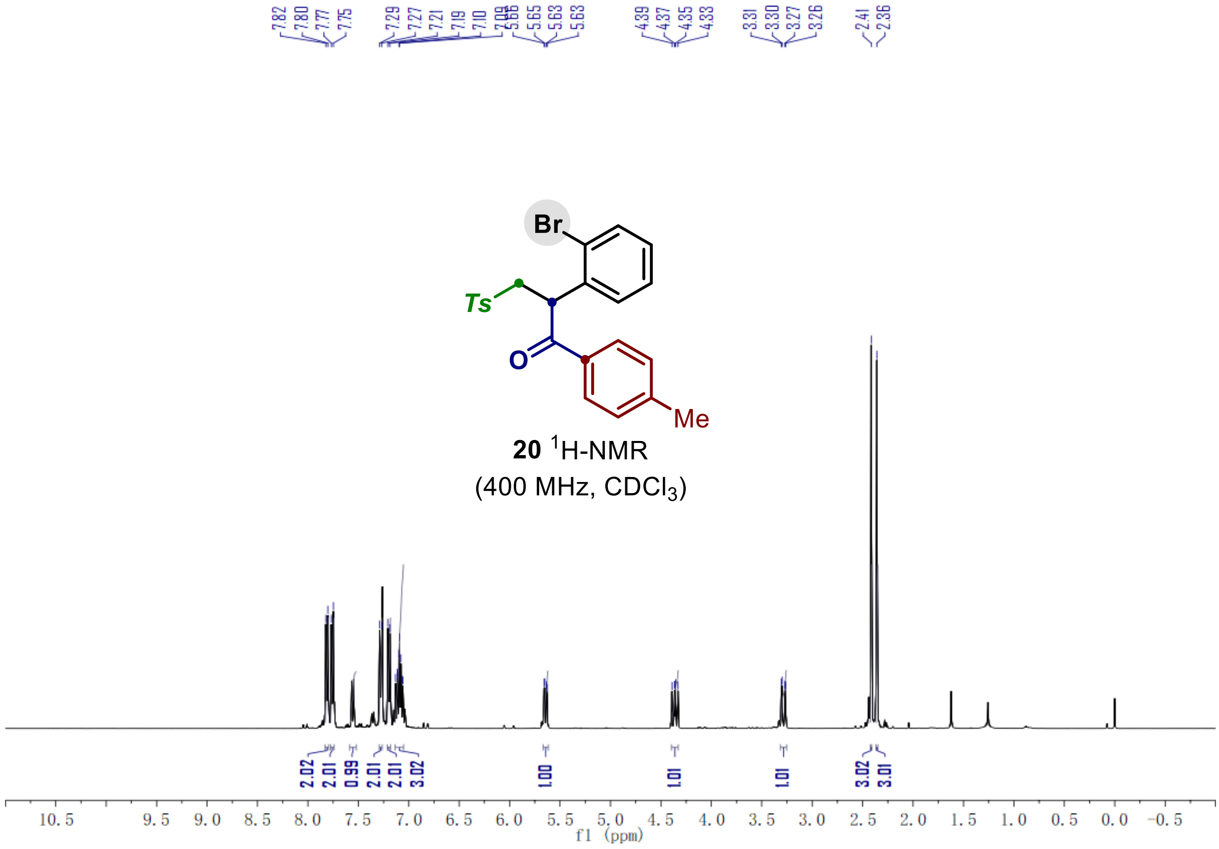
Br
Ts
O
Me
20 1H-NMR
(400 MHz, CDCl3)
f1 (ppm)

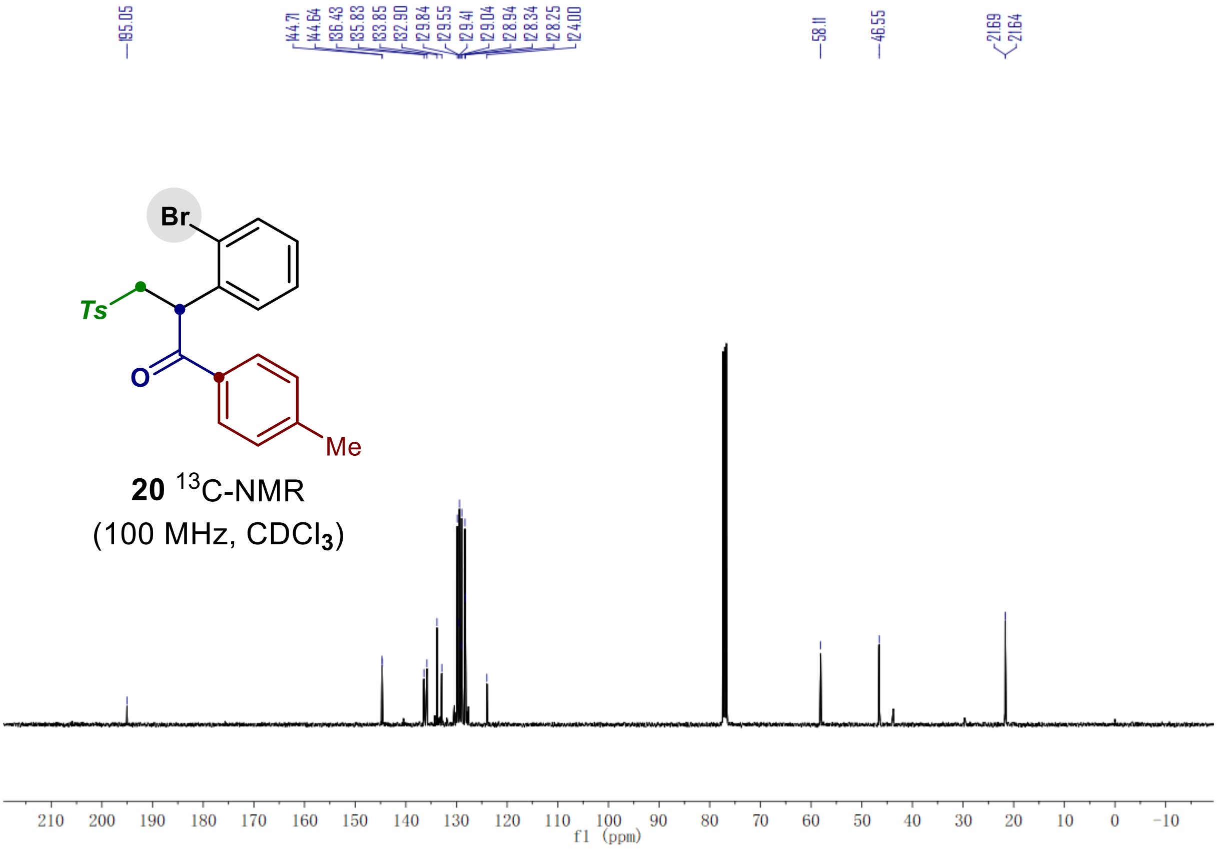
195.05
144.71
144.64
136.43
135.83
133.85
132.90
129.84
129.55
129.41
129.04
128.94
128.34
128.25
124.00
58.11
46.55
21.69
21.64
Br
Ts
O
Me
20 13C-NMR
(100 MHz, CDCl3)
f1 (ppm)

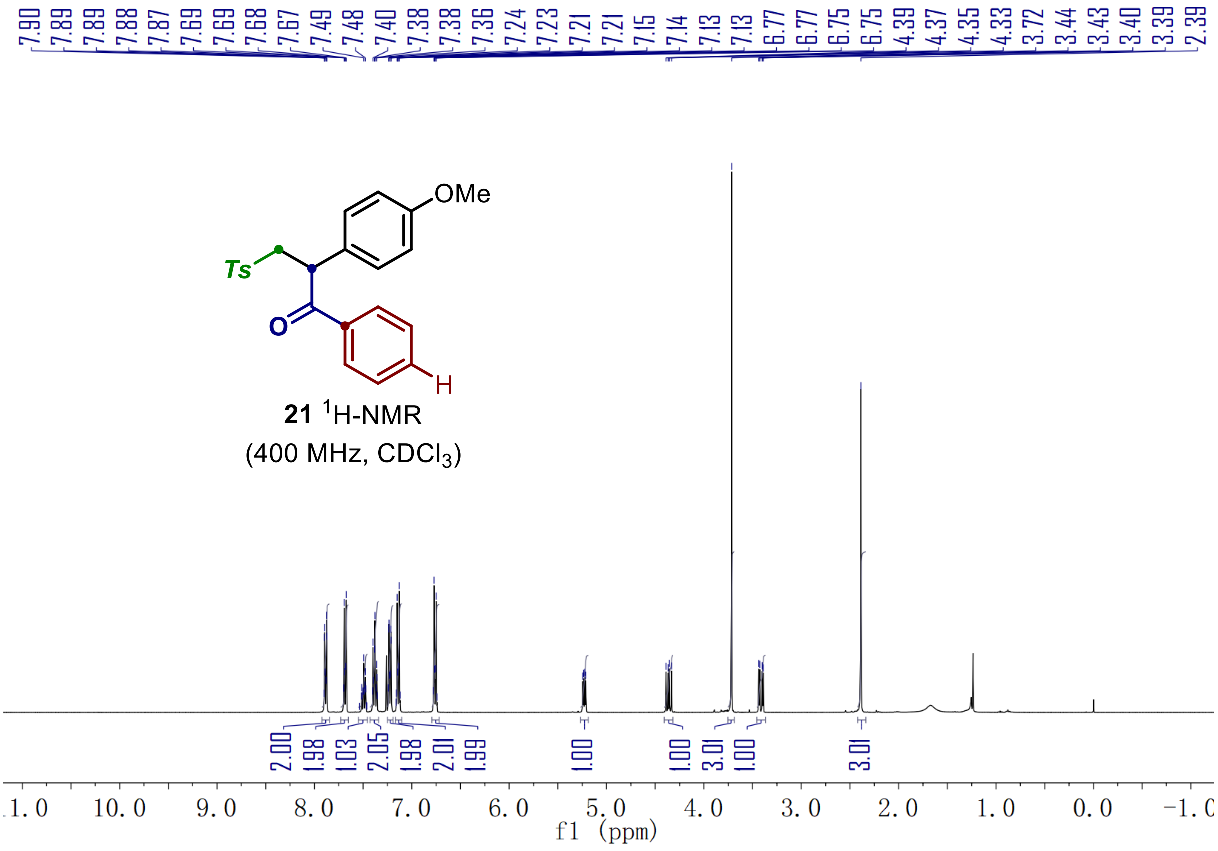
7.90
7.89
7.89
7.88
7.87
7.69
7.69
7.69
7.68
7.67
7.49
7.48
7.40
7.38
7.38
7.36
7.24
7.23
7.21
7.21
7.15
7.14
7.13
7.13
6.77
6.77
6.75
6.75
4.39
4.37
4.35
4.33
3.72
3.44
3.43
3.40
3.39
2.39
OMe
Ts
O
H
21 1H-NMR
(400 MHz, CDCl3)
2.00
1.98
1.03
2.05
1.98
2.01
1.99
1.00
1.00
3.01
1.00
3.01
f1 (ppm)

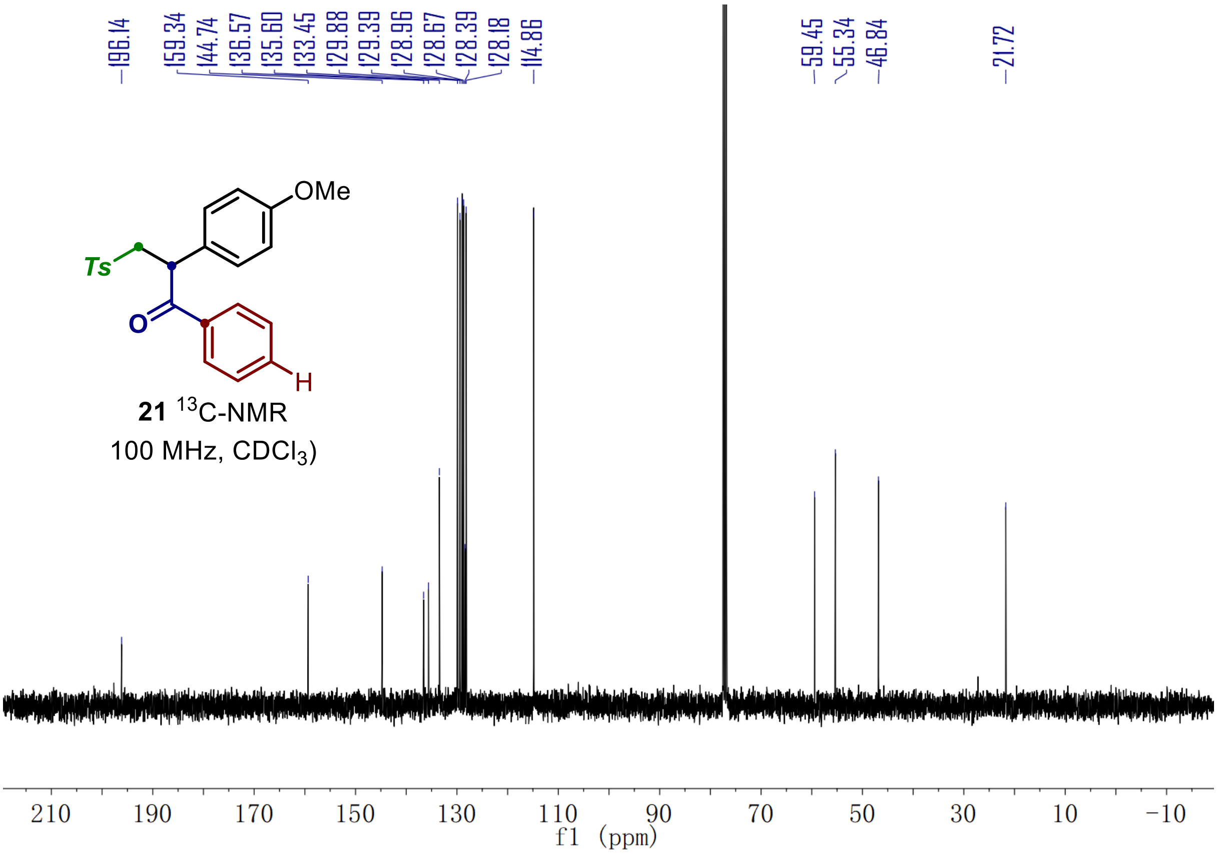
196.14
159.34
144.74
136.57
135.60
133.45
129.88
129.39
128.96
128.67
128.39
128.18
114.86
59.45
55.34
46.84
21.72
OMe
Ts
O
H
21 ^{13}C-NMR
100 MHz, $CDCl_3$)
210
190
170
150
130
110
90
70
50
30
10
−10
f1 (ppm)

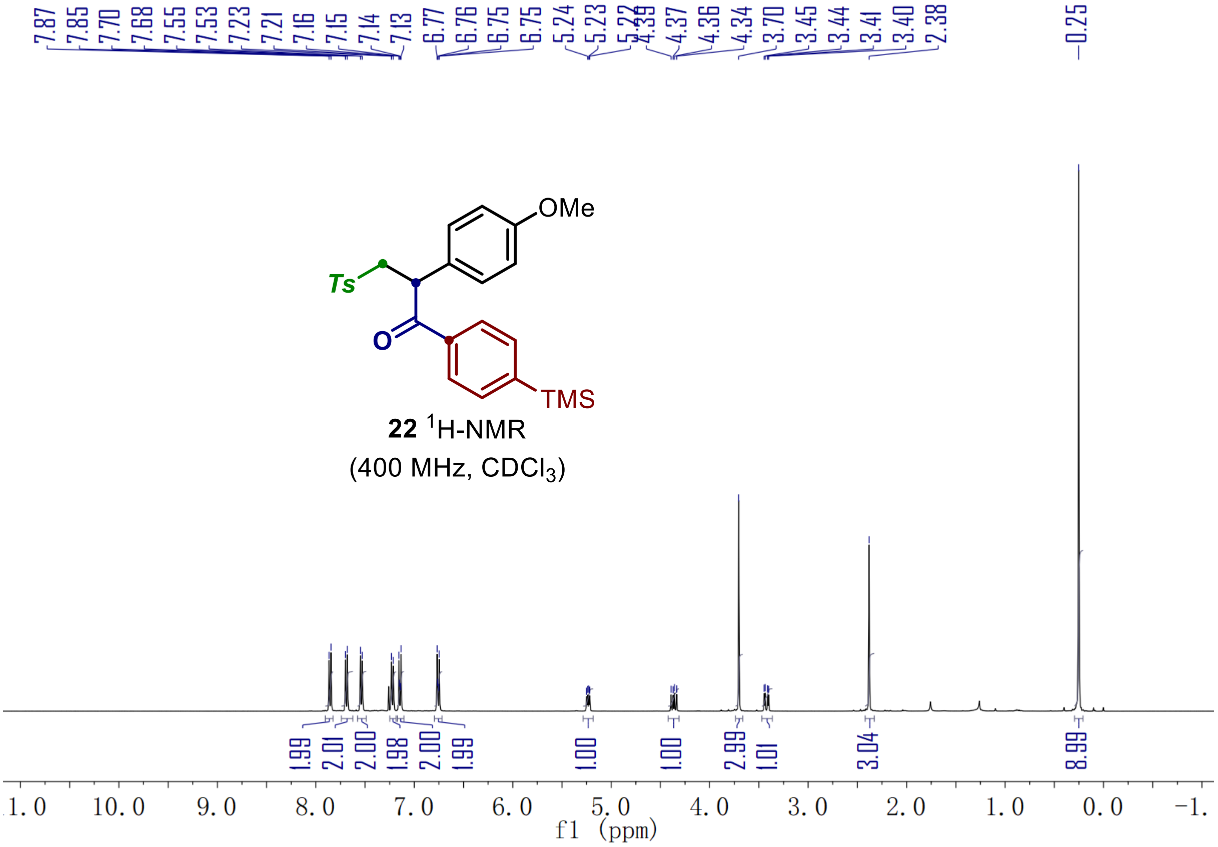
7.87
7.85
7.70
7.68
7.55
7.53
7.23
7.21
7.16
7.15
7.14
7.13
6.77
6.76
6.75
6.75
5.24
5.23
5.22
4.39
4.37
4.36
4.34
3.70
3.45
3.44
3.41
3.40
2.38
0.25
OMe
Ts
O
TMS
22 ^{1}H-NMR
(400 MHz, $CDCl_3$)
1.99
2.01
2.00
1.98
2.00
1.99
1.00
1.00
2.99
1.01
3.04
8.99
10.0
9.0
8.0
7.0
6.0
5.0
4.0
3.0
2.0
1.0
0.0
f1 (ppm)

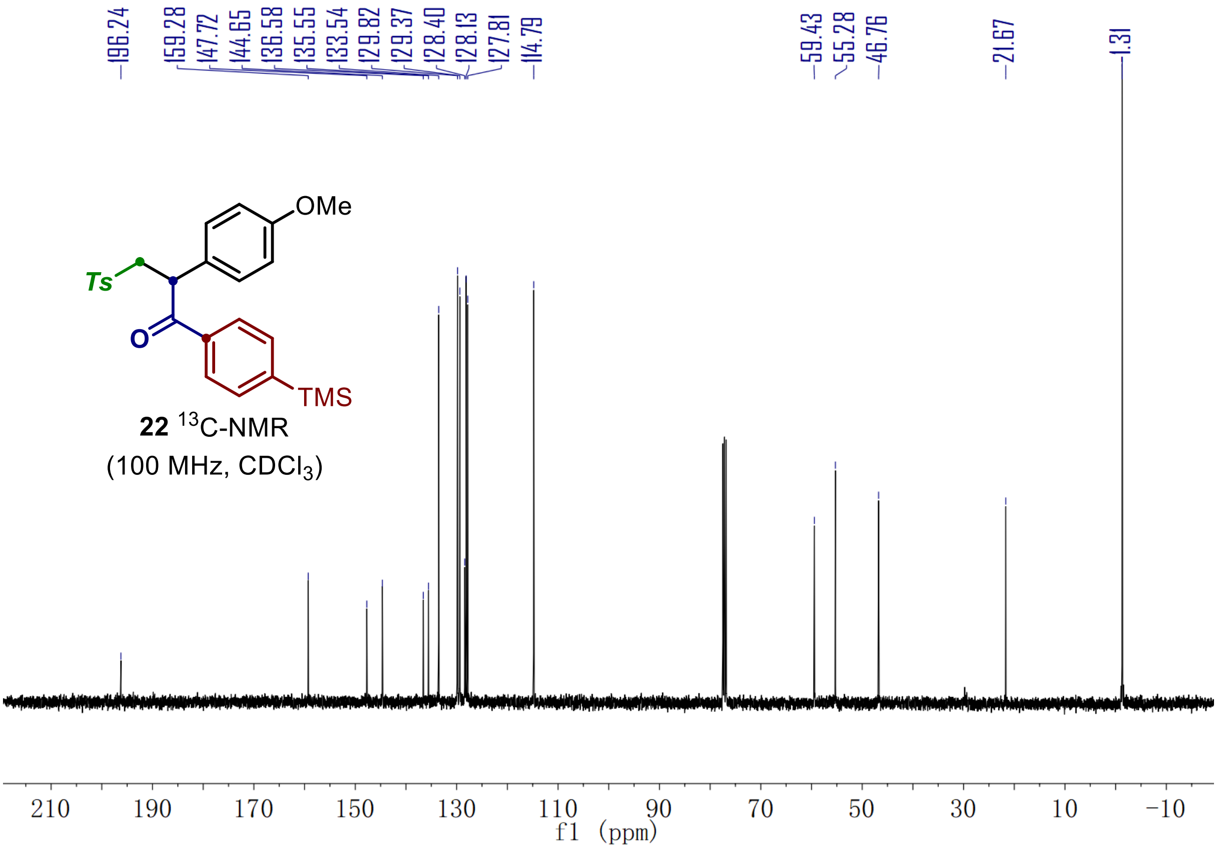
196.24
159.28
147.72
144.65
136.58
135.55
133.54
129.82
129.37
128.40
128.13
127.81
114.79
59.43
55.28
46.76
21.67
-1.31
OMe
Ts
O
TMS
22 13C-NMR
(100 MHz, CDCl3)
210 190 170 150 130 110 90 70 50 30 10 -10
f1 (ppm)

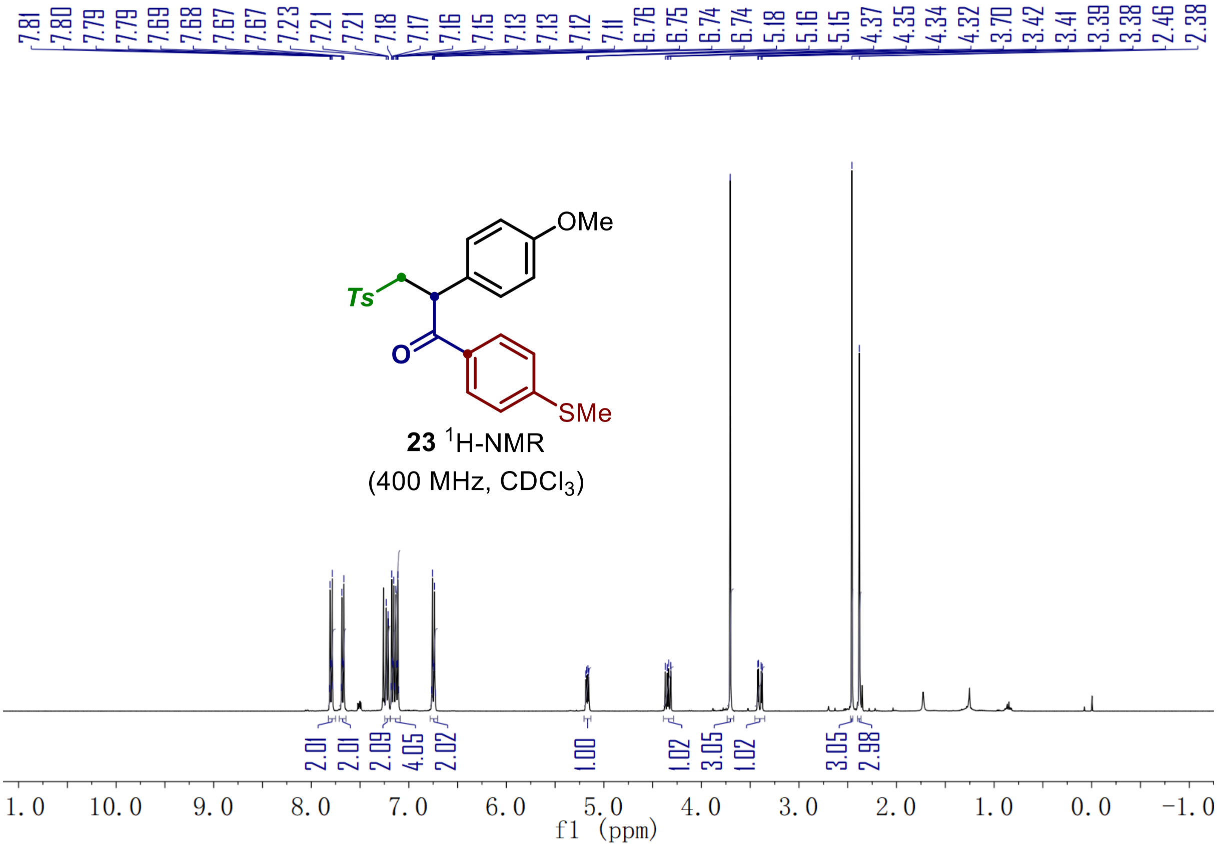
7.81
7.80
7.79
7.79
7.69
7.68
7.67
7.67
7.23
7.21
7.21
7.18
7.17
7.16
7.15
7.13
7.13
7.12
7.11
6.76
6.75
6.74
6.74
5.18
5.16
5.15
4.37
4.35
4.34
4.32
3.70
3.42
3.41
3.39
3.38
2.46
2.38
OMe
Ts
O
SMe
23 1H-NMR
(400 MHz, CDCl3)
2.01
2.01
2.09
4.05
2.02
1.00
1.02
3.05
1.02
3.05
2.98
1.0 10.0 9.0 8.0 7.0 6.0 5.0 4.0 3.0 2.0 1.0 0.0 -1.0
f1 (ppm)

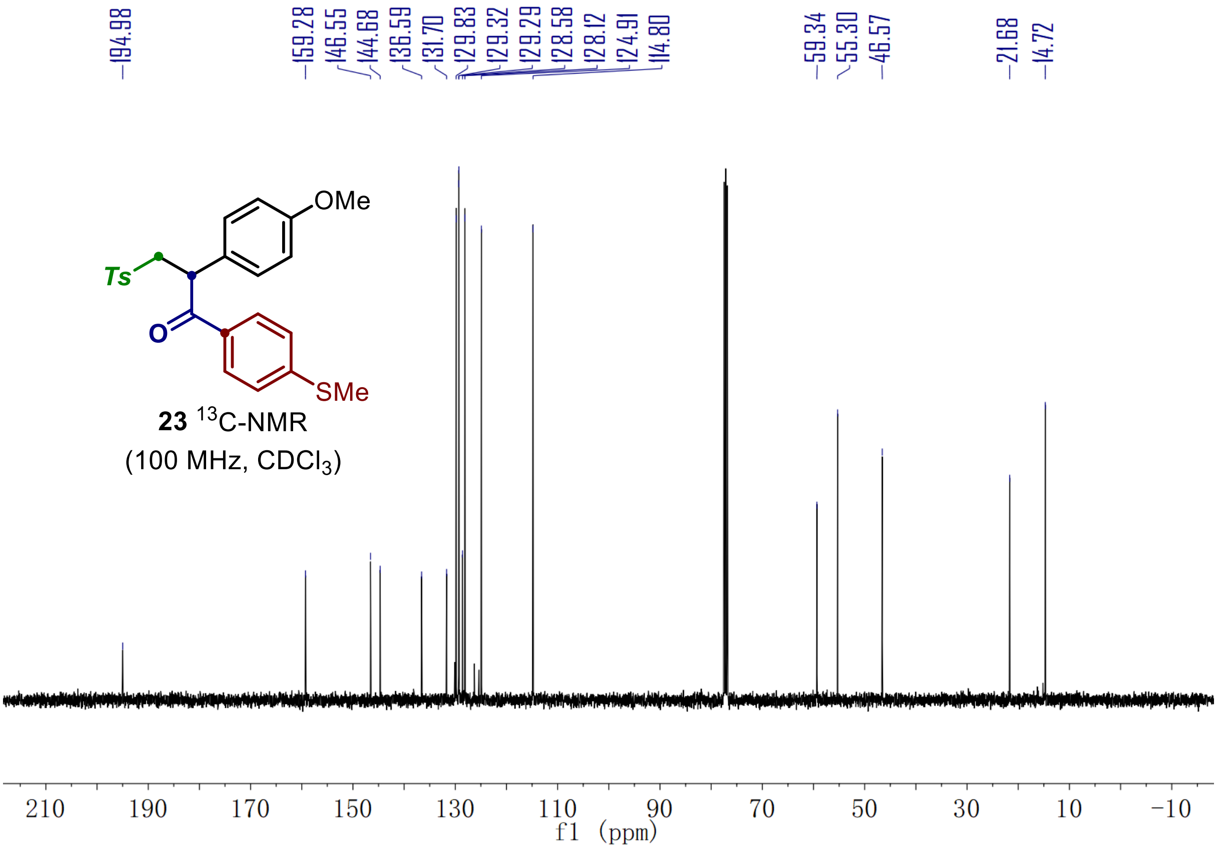
194.98
159.28
146.55
144.68
136.59
131.70
129.83
129.32
129.29
128.58
128.12
124.91
114.80
59.34
55.30
46.57
21.68
14.72
OMe
Ts
O
SMe
23 13C-NMR
(100 MHz, CDCl3)
210
190
170
150
130
110
90
70
50
30
10
−10
f1 (ppm)

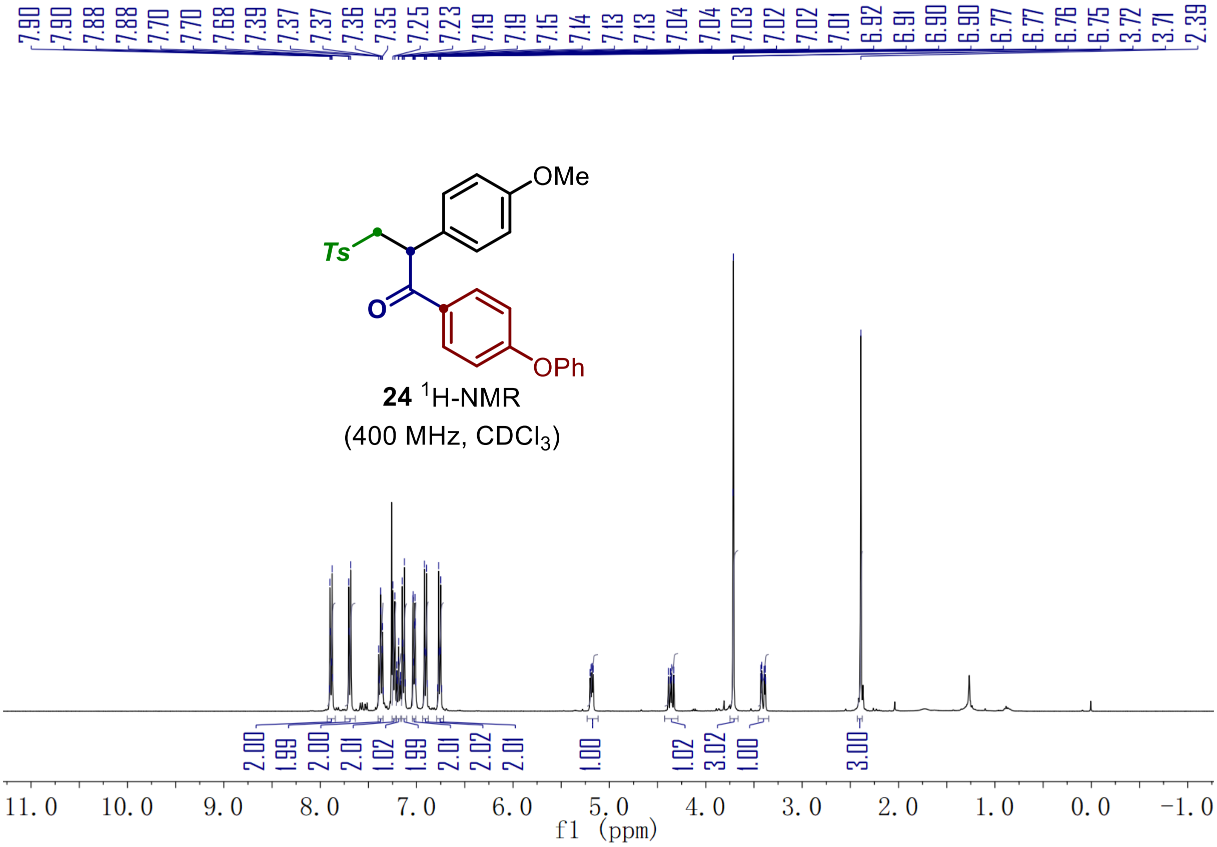
7.90
7.90
7.88
7.88
7.70
7.70
7.68
7.39
7.37
7.37
7.36
7.35
7.25
7.23
7.19
7.19
7.15
7.14
7.13
7.13
7.04
7.04
7.03
7.02
7.02
7.01
6.92
6.91
6.90
6.90
6.77
6.77
6.76
6.75
3.72
3.71
2.39
OMe
Ts
O
OPh
24 1H-NMR
(400 MHz, CDCl3)
2.00
1.99
2.00
2.01
1.02
1.99
2.01
2.02
2.01
1.00
1.02
3.02
1.00
3.00
11.0
10.0
9.0
8.0
7.0
6.0
5.0
4.0
3.0
2.0
1.0
0.0
−1.0
f1 (ppm)

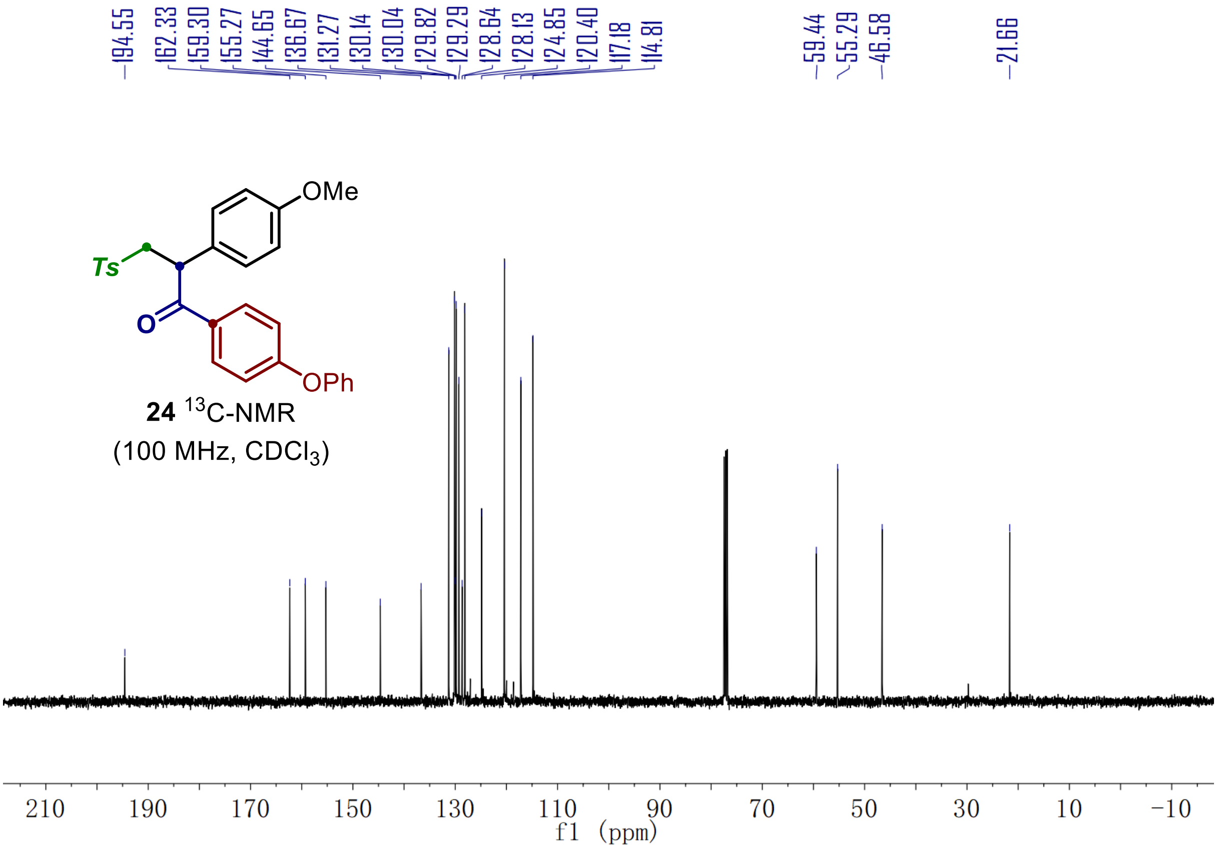
194.55
162.33
159.30
155.27
144.65
136.67
131.27
130.14
130.04
129.82
129.29
128.64
128.13
124.85
120.40
117.18
114.81
59.44
55.29
46.58
21.66
OMe
Ts
O
OPh
24 ^{13}C-NMR
(100 MHz, $CDCl_3$)
210 190 170 150 130 110 90 70 50 30 10 −10
f1 (ppm)

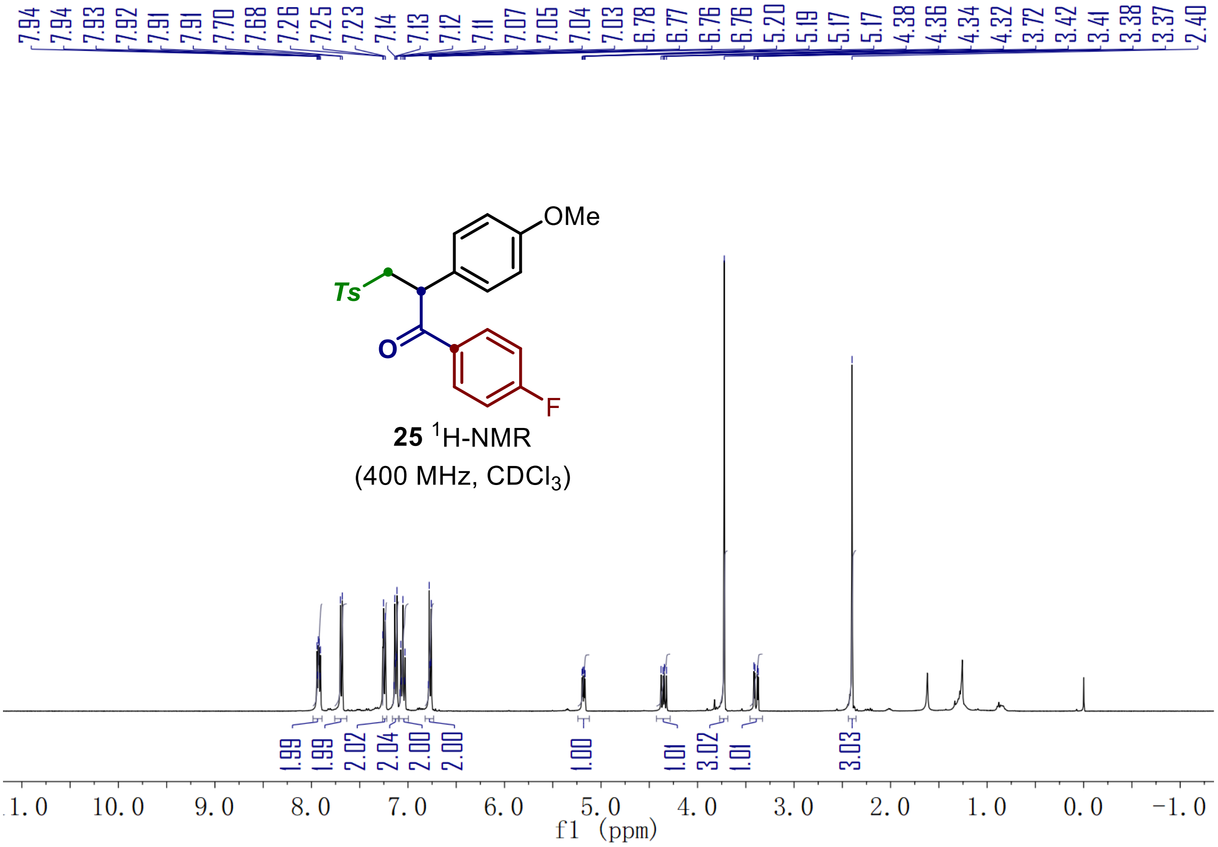
7.94
7.94
7.93
7.92
7.91
7.91
7.70
7.68
7.26
7.25
7.23
7.14
7.13
7.12
7.11
7.07
7.05
7.04
7.03
6.78
6.77
6.76
6.76
5.20
5.19
5.17
5.17
4.38
4.36
4.34
4.32
3.72
3.42
3.41
3.38
3.37
2.40
OMe
Ts
O
F
25 ^{1}H-NMR
(400 MHz, $CDCl_3$)
1.99
1.99
2.02
2.04
2.00
2.00
1.00
1.01
3.02
1.01
3.03
1.0 10.0 9.0 8.0 7.0 6.0 5.0 4.0 3.0 2.0 1.0 0.0 −1.0
f1 (ppm)

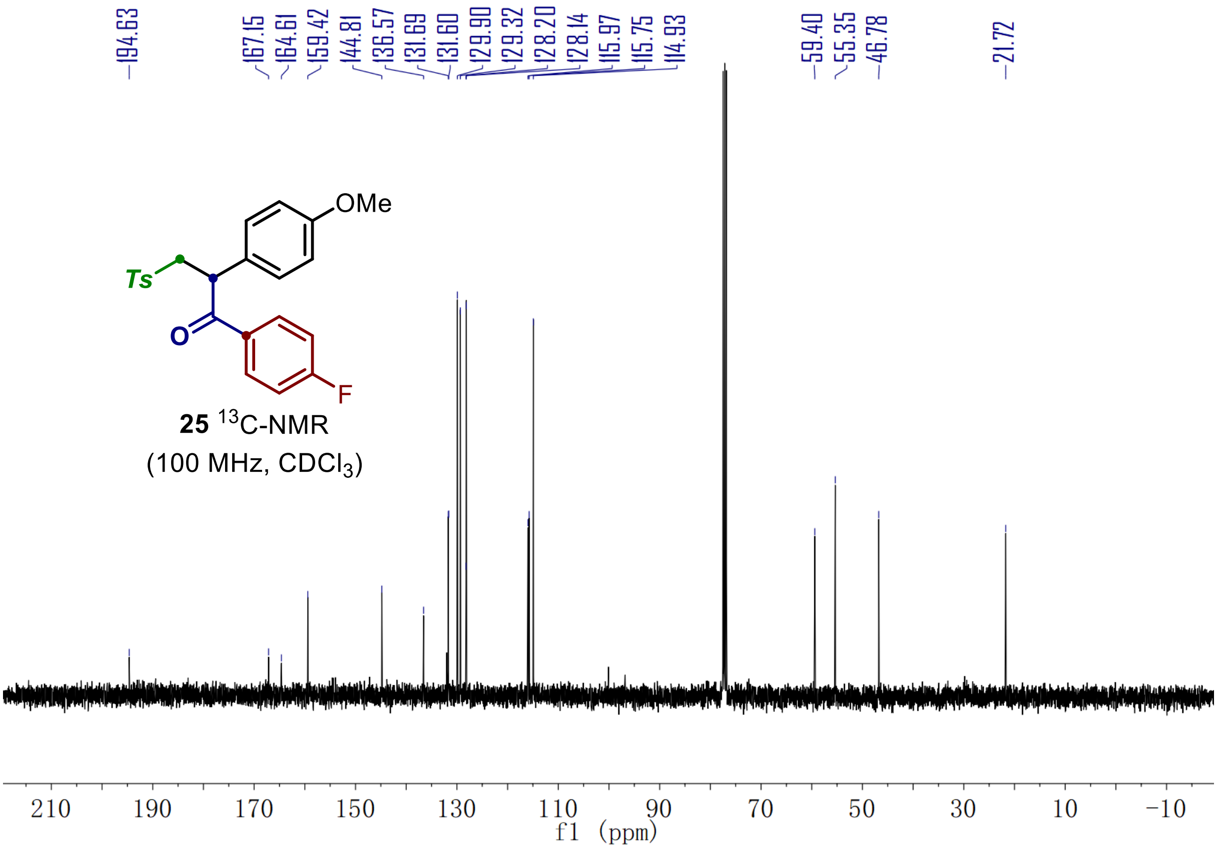
194.63
167.15
164.61
159.42
144.81
136.57
131.69
131.60
129.90
129.32
128.20
128.14
115.97
115.75
114.93
59.40
55.35
46.78
21.72
OMe
Ts
O
F
25 13C-NMR
(100 MHz, CDCl3)
210
190
170
150
130
110
90
70
50
30
10
−10
f1 (ppm)

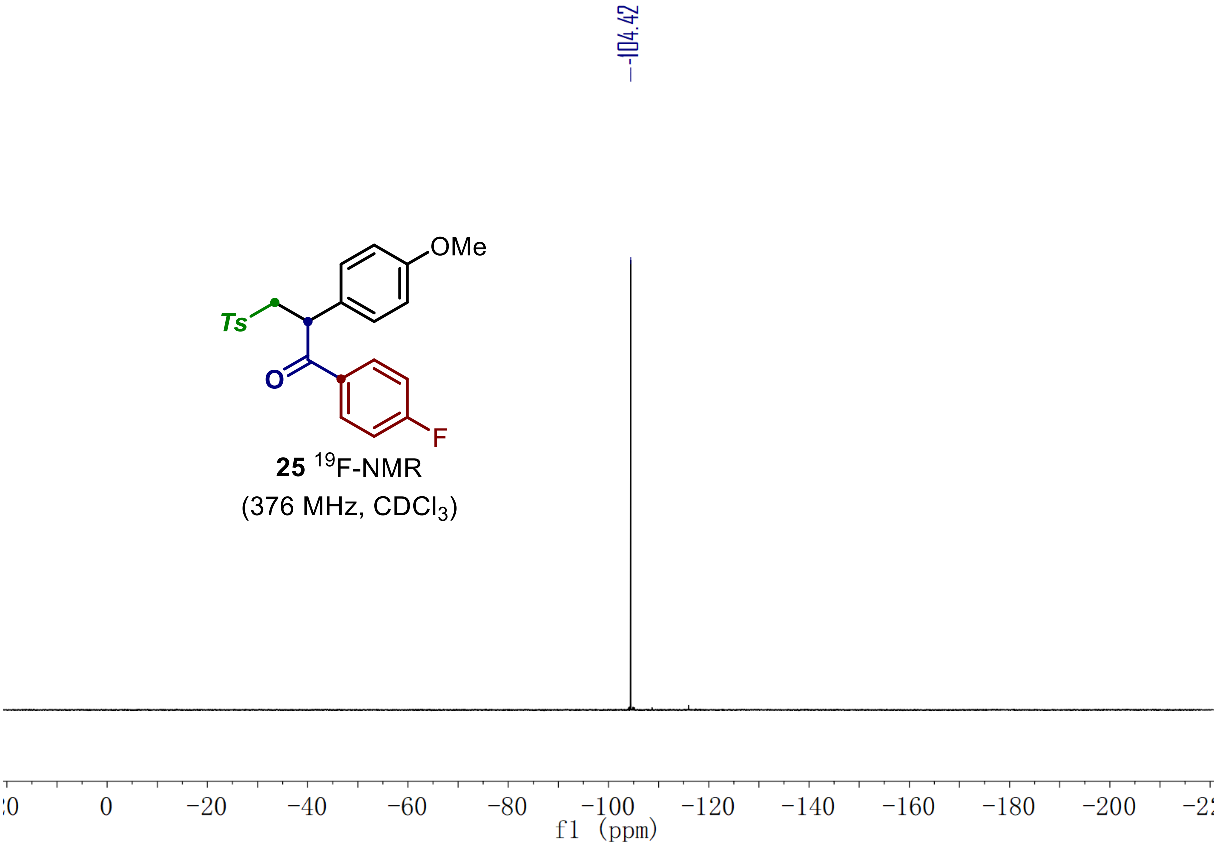
−104.42
OMe
Ts
O
F
25 19F-NMR
(376 MHz, CDCl3)
0
−20
−40
−60
−80
−100
−120
−140
−160
−180
−200
f1 (ppm)

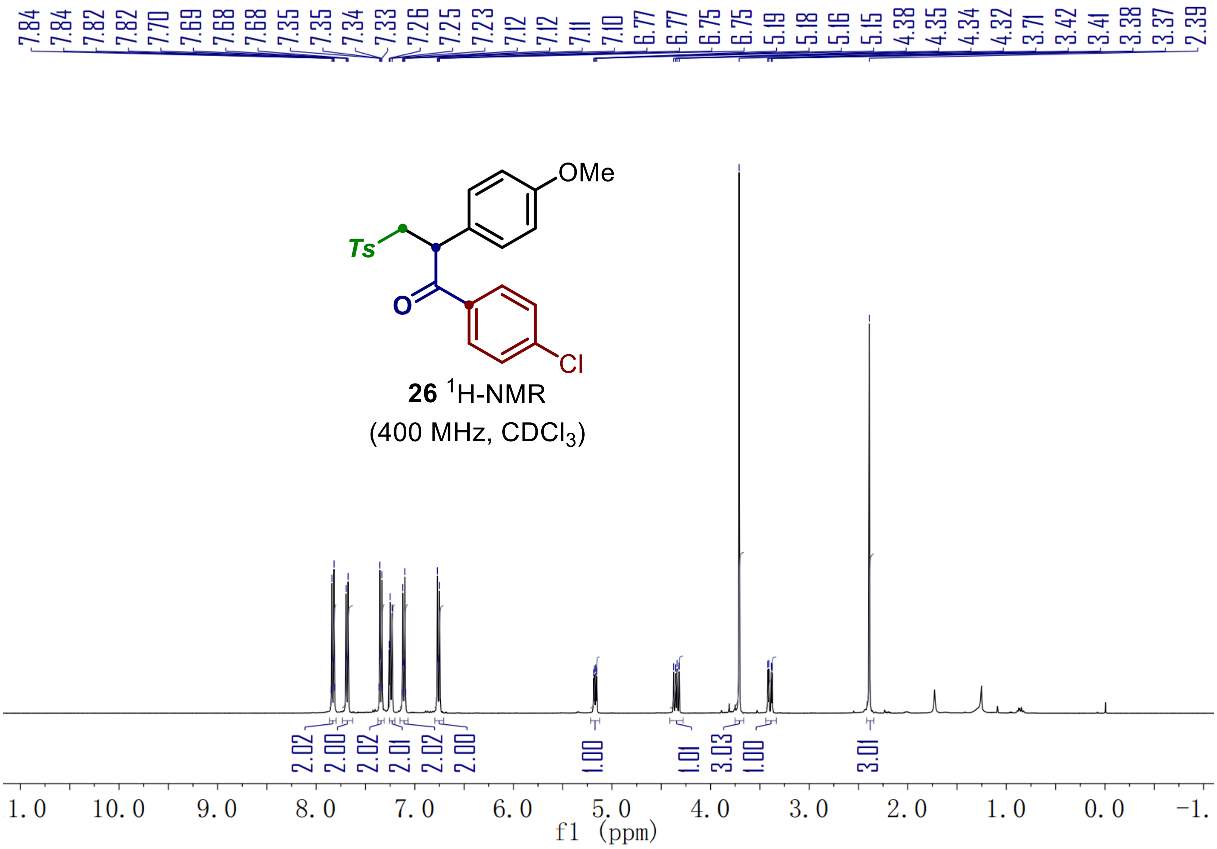

OMe
Ts
O
Cl
26 1H-NMR
(400 MHz, CDCl3)
f1 (ppm)

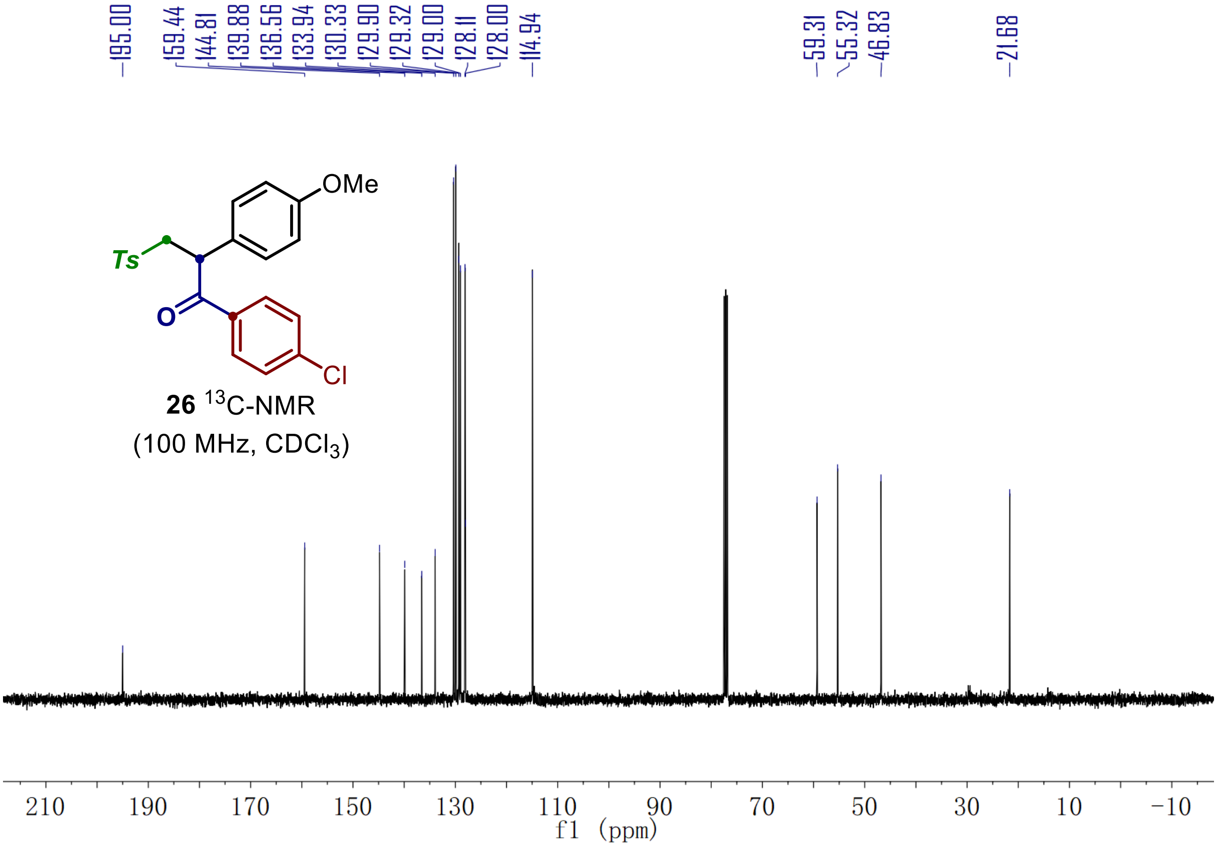

OMe
Ts
O
Cl
26 13C-NMR
(100 MHz, CDCl3)
f1 (ppm)

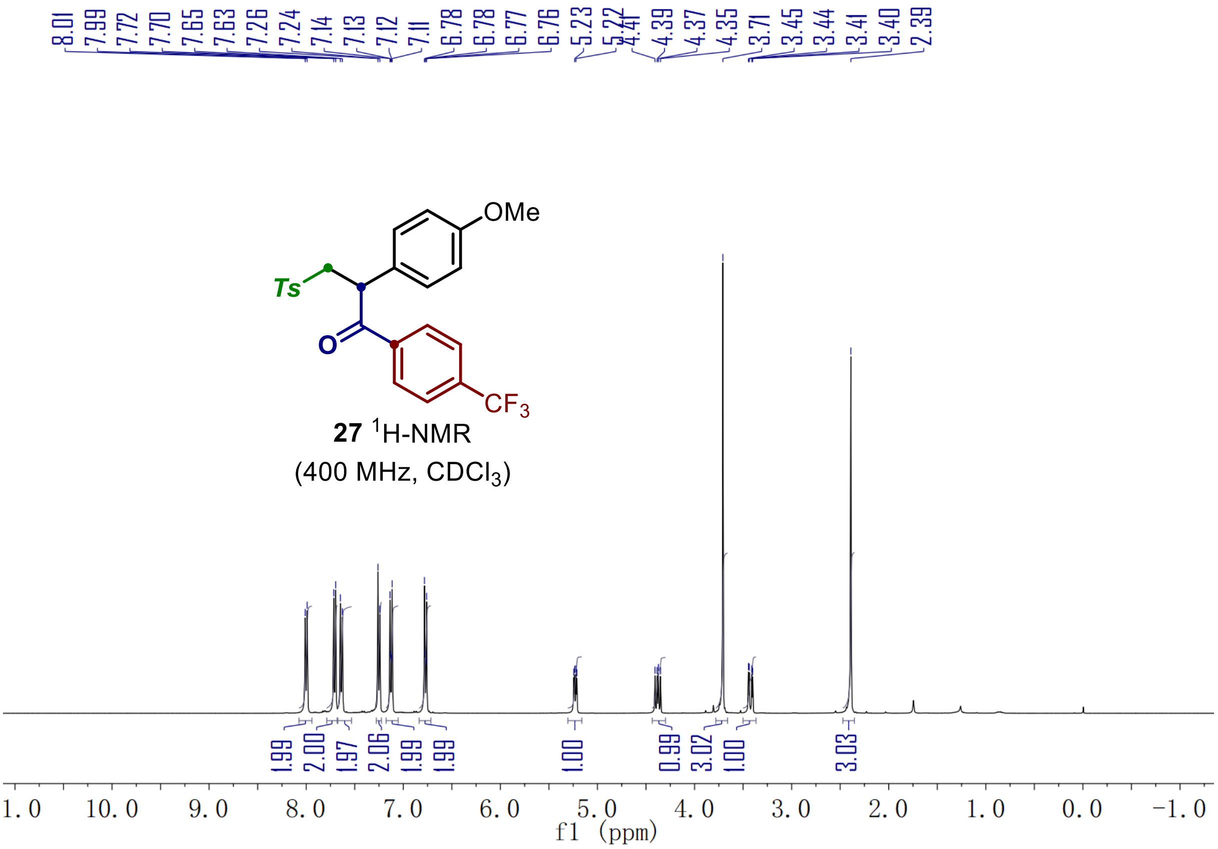

OMe
Ts
O
CF3
27 1H-NMR
(400 MHz, CDCl3)
f1 (ppm)

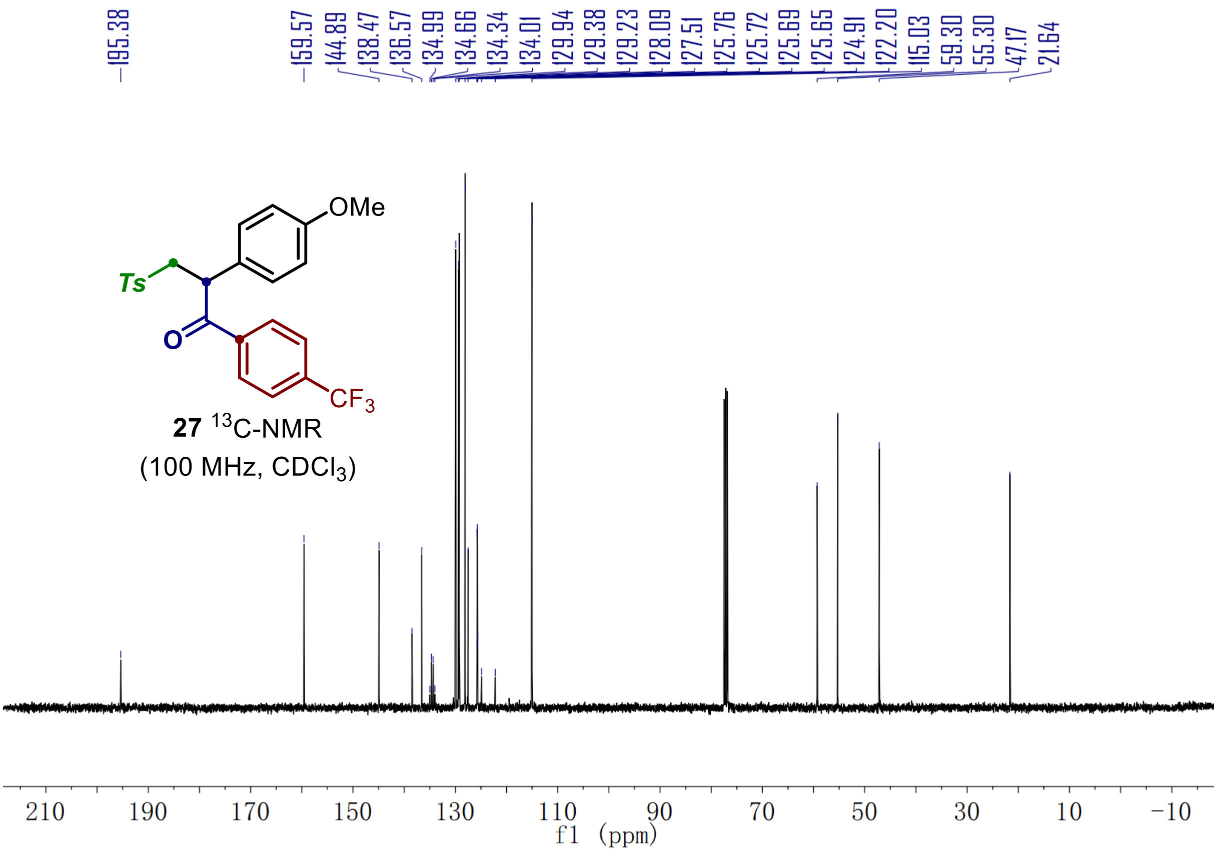

OMe
Ts
O
CF3
27 13C-NMR
(100 MHz, CDCl3)
f1 (ppm)

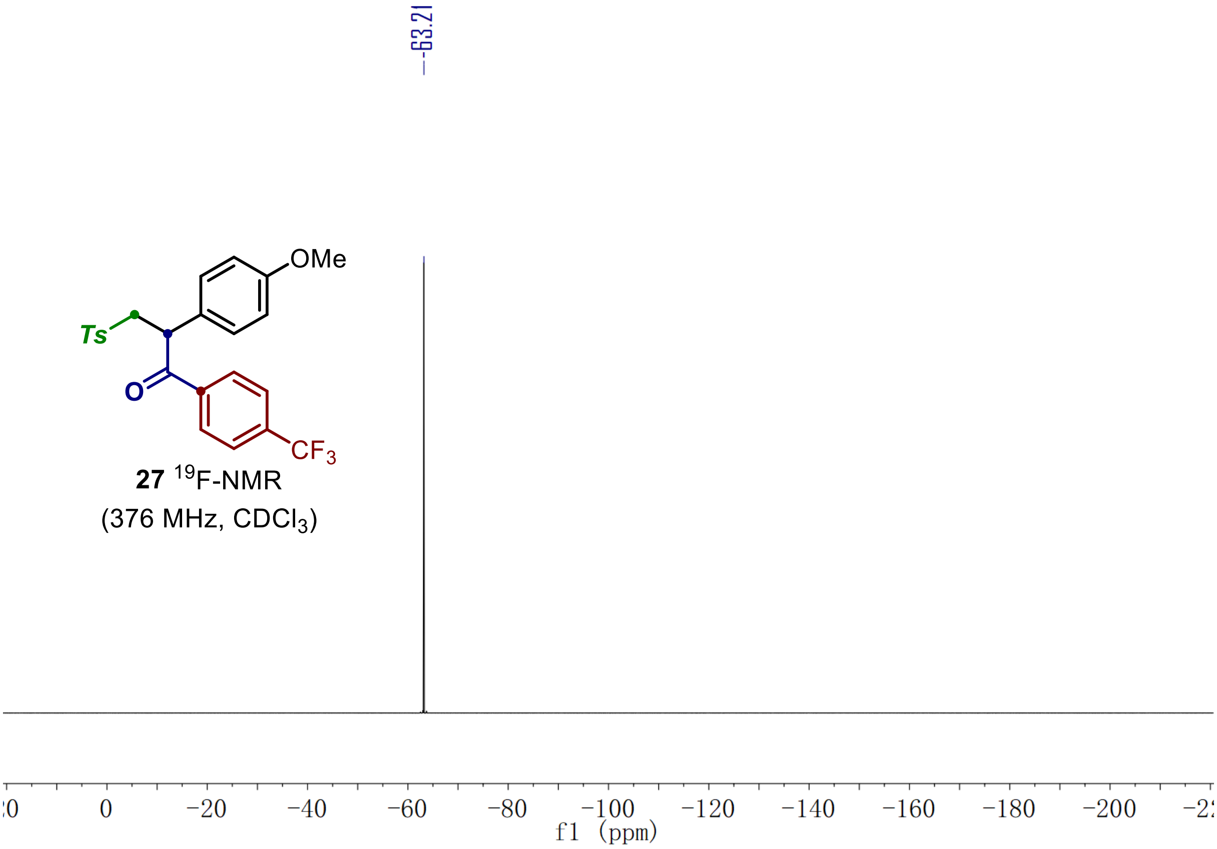
-63.21
OMe
Ts
O
CF3
27 19F-NMR
(376 MHz, CDCl3)
f1 (ppm)

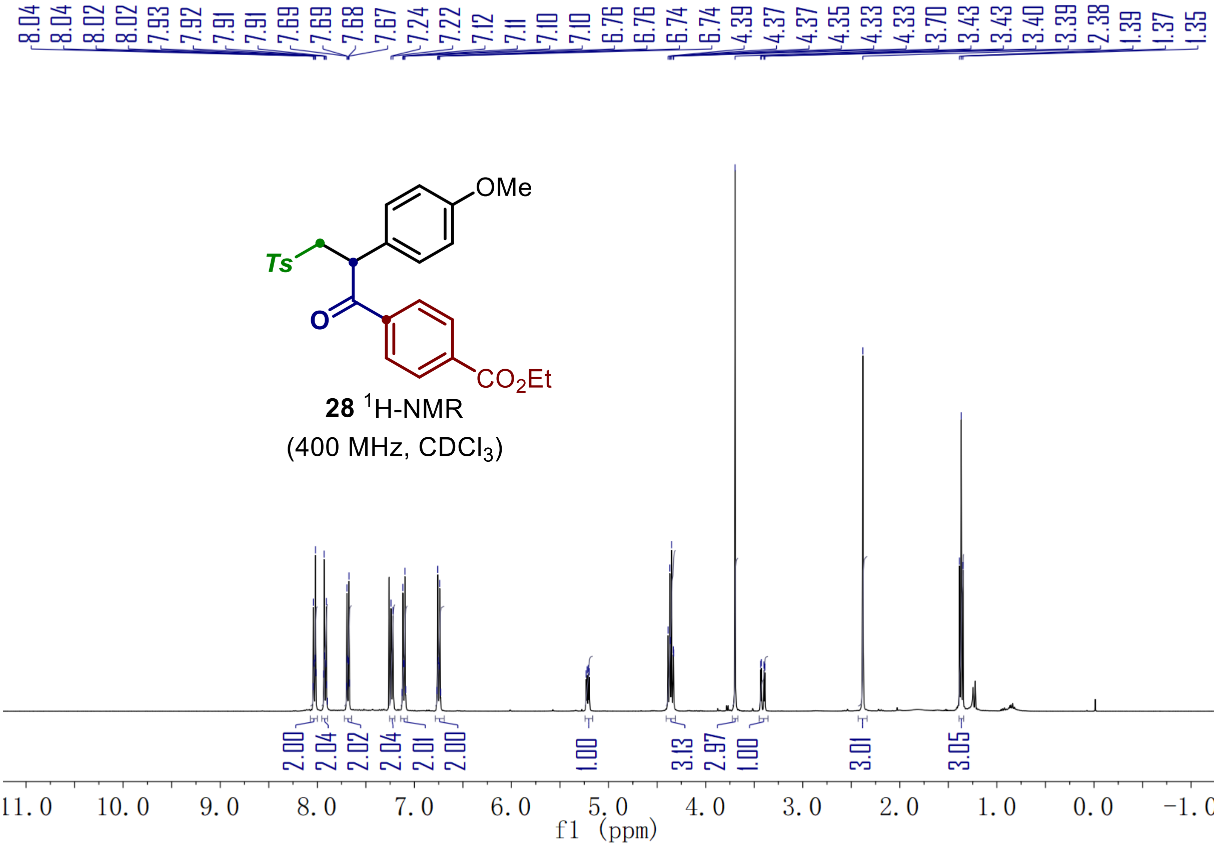
8.04 8.04 8.02 8.02 7.93 7.92 7.91 7.91 7.69 7.69 7.68 7.67 7.24 7.22 7.12 7.11 7.10 7.10 6.76 6.76 6.74 6.74 4.39 4.37 4.37 4.35 4.33 4.33 3.70 3.43 3.43 3.40 3.39 2.38 1.39 1.37 1.35
OMe
Ts
O
CO2Et
28 1H-NMR
(400 MHz, CDCl3)
2.00 2.04 2.02 2.04 2.01 2.00 1.00 3.13 2.97 1.00 3.01 3.05
f1 (ppm)

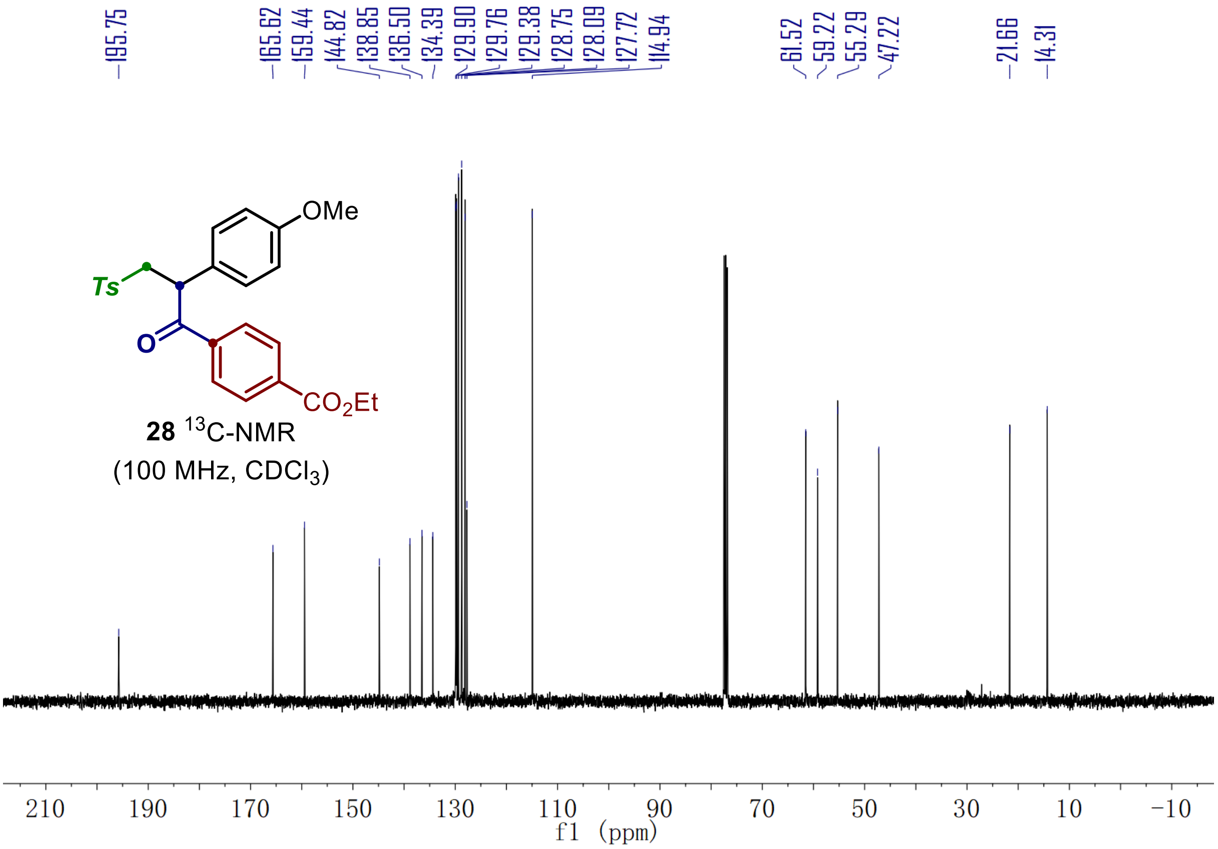
195.75
165.62
159.44
144.82
138.85
136.50
134.39
129.90
129.76
129.38
128.75
128.09
127.72
114.94
61.52
59.22
55.29
47.22
21.66
14.31
OMe
Ts
O
CO2Et
28 13C-NMR
(100 MHz, CDCl3)
f1 (ppm)

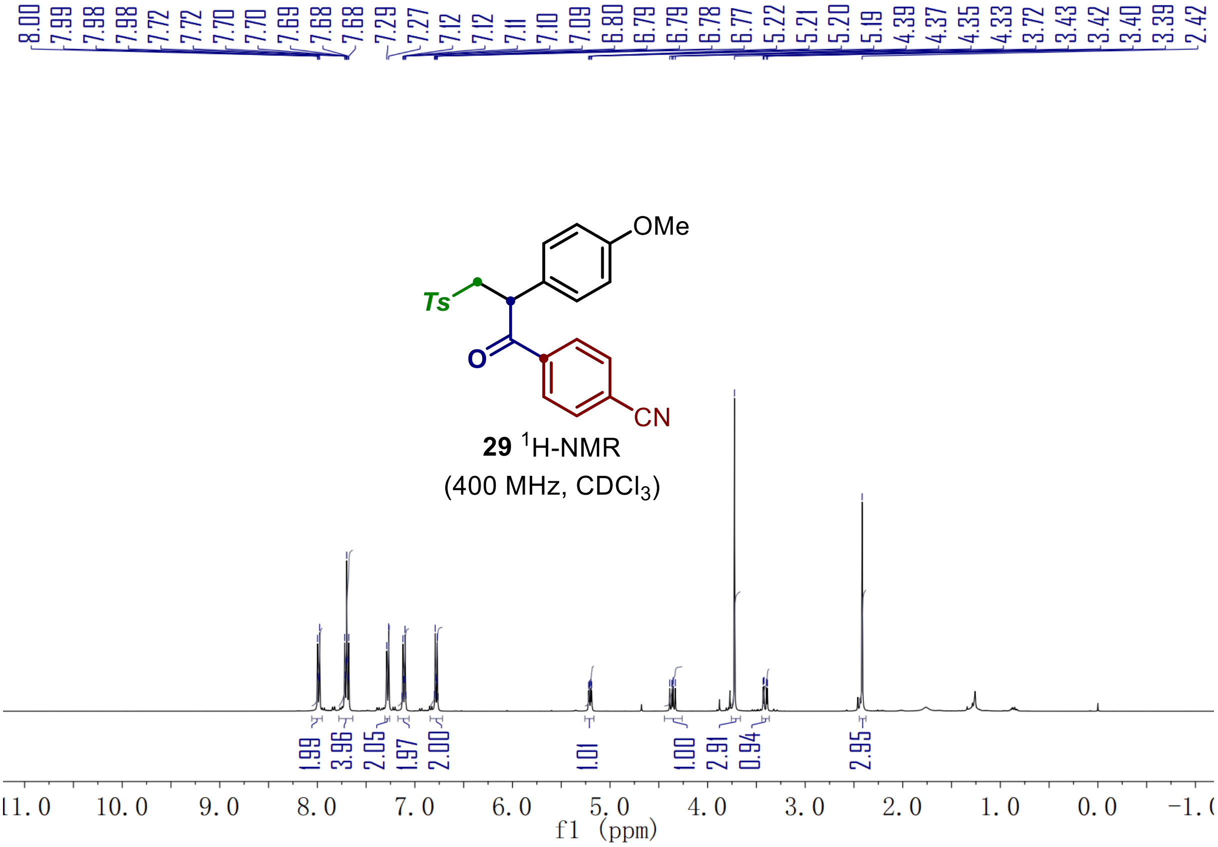
OMe
Ts
O
CN
29 1H-NMR
(400 MHz, CDCl3)
1.99
3.96
2.05
1.97
2.00
1.01
1.00
2.91
0.94
2.95
f1 (ppm)

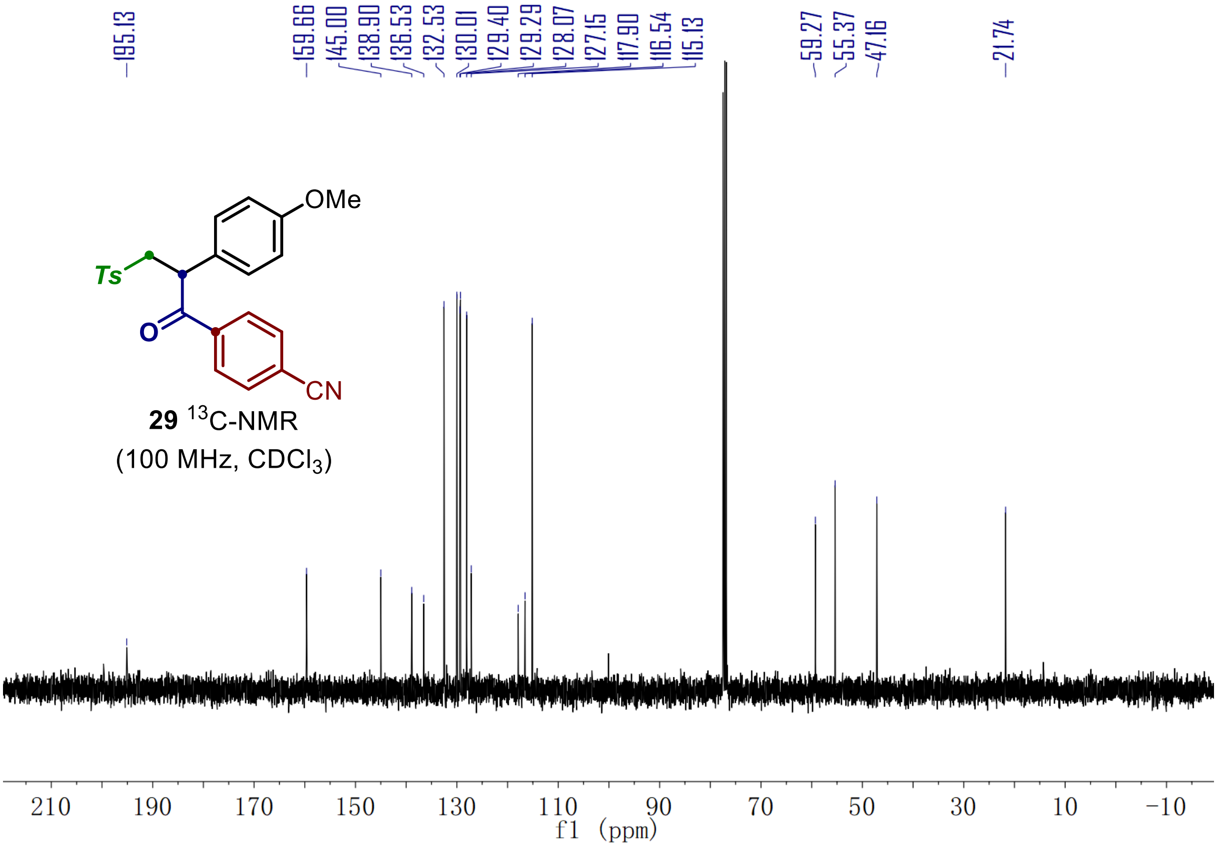
195.13
159.66
145.00
138.90
136.53
132.53
130.01
129.40
129.29
128.07
127.15
117.90
116.54
115.13
59.27
55.37
47.16
21.74
OMe
Ts
O
CN
29 ^{13}C-NMR
(100 MHz, $CDCl_3$)
210
190
170
150
130
110
90
70
50
30
10
−10
f1 (ppm)

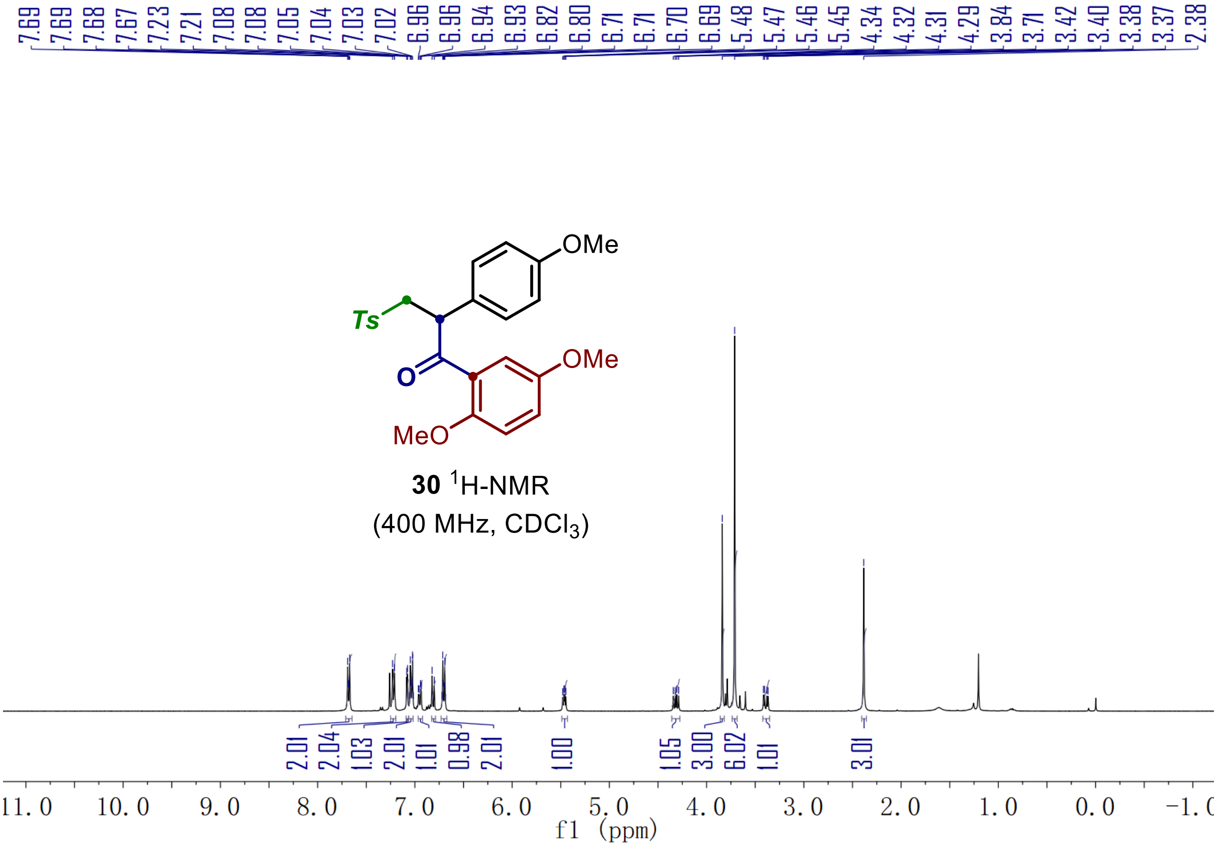
7.69
7.69
7.68
7.67
7.23
7.21
7.08
7.08
7.05
7.04
7.03
7.02
6.96
6.96
6.94
6.93
6.82
6.80
6.71
6.71
6.70
6.69
5.48
5.47
5.46
5.45
4.34
4.32
4.31
4.29
3.84
3.71
3.42
3.40
3.38
3.37
2.38
OMe
Ts
O
OMe
MeO
30 ^{1}H-NMR
(400 MHz, $CDCl_3$)
2.01
2.04
1.03
2.01
1.01
0.98
2.01
1.00
1.05
3.00
6.02
1.01
3.01
11.0
10.0
9.0
8.0
7.0
6.0
5.0
4.0
3.0
2.0
1.0
0.0
−1.0
f1 (ppm)

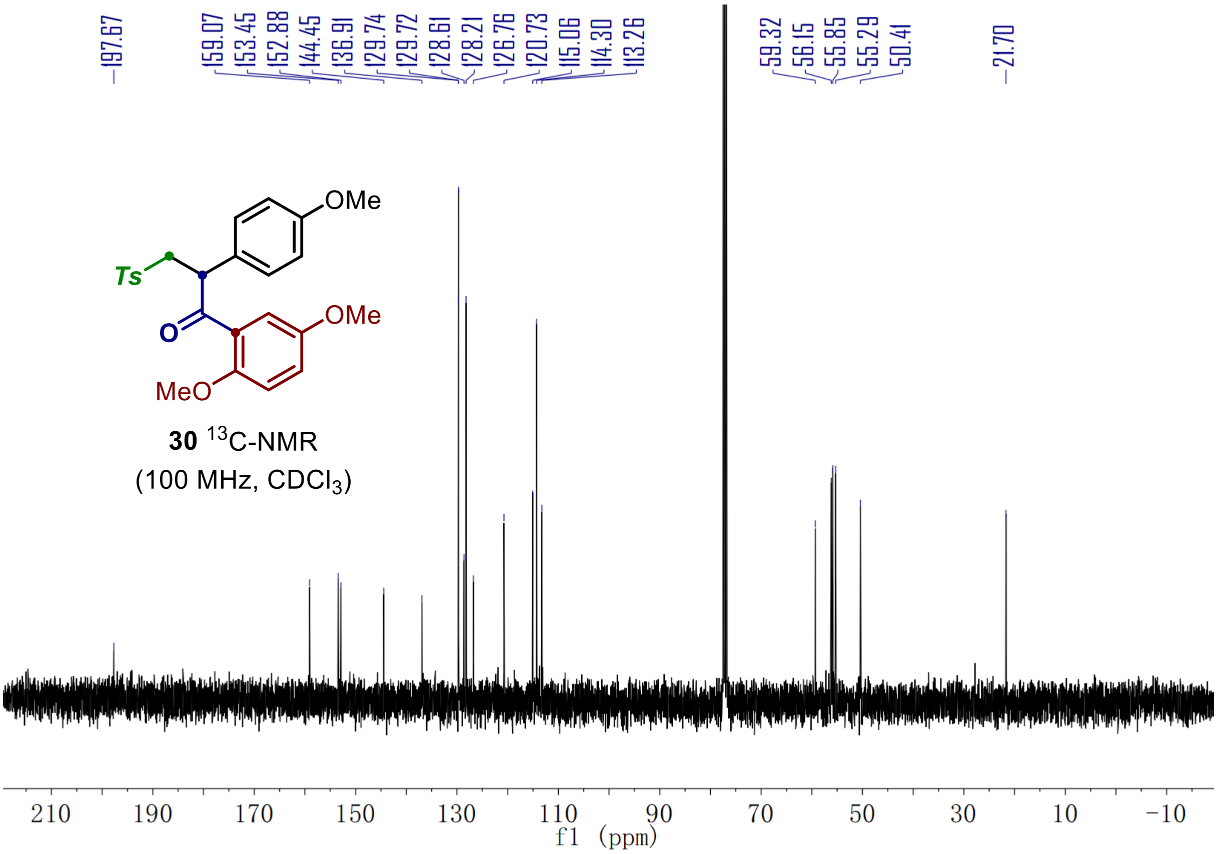
197.67
159.07
153.45
152.88
144.45
136.91
129.74
129.72
128.61
128.21
126.76
120.73
115.06
114.30
113.26
59.32
56.15
55.85
55.29
50.41
21.70
OMe
Ts
O
OMe
MeO
30 ^{13}C-NMR
(100 MHz, $CDCl_3$)
210 190 170 150 130 110 90 70 50 30 10 −10
f1 (ppm)

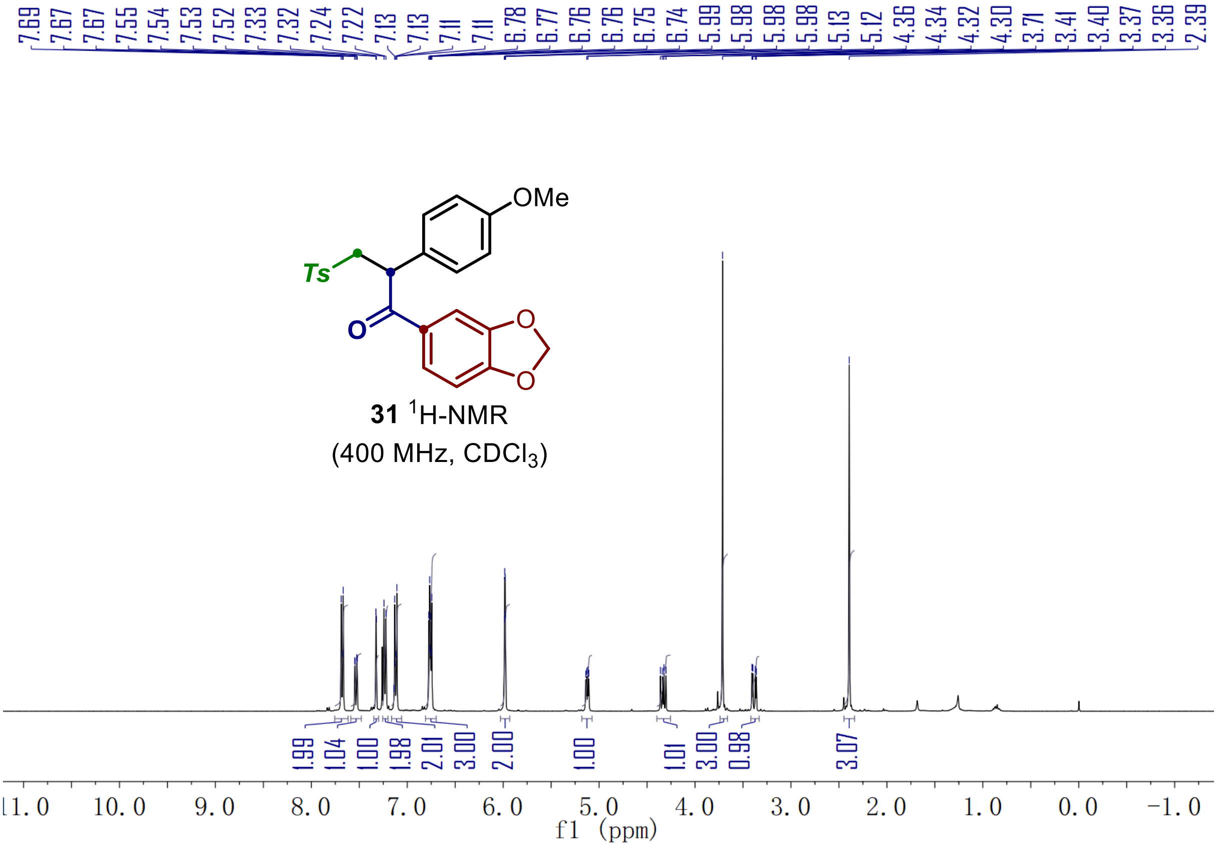
7.69 7.67 7.67 7.55 7.54 7.53 7.52 7.33 7.32 7.24 7.22 7.13 7.13 7.11 7.11 6.78 6.77 6.76 6.76 6.75 6.74 5.99 5.98 5.98 5.98 5.13 5.12 4.36 4.34 4.32 4.30 3.71 3.41 3.40 3.37 3.36 2.39
OMe
Ts
O
O
O
31 ^{1}H-NMR
(400 MHz, $CDCl_3$)
1.99 1.04 1.00 1.98 2.01 3.00 2.00 1.00 1.01 3.00 0.98 3.07
11.0 10.0 9.0 8.0 7.0 6.0 5.0 4.0 3.0 2.0 1.0 0.0 −1.0
f1 (ppm)

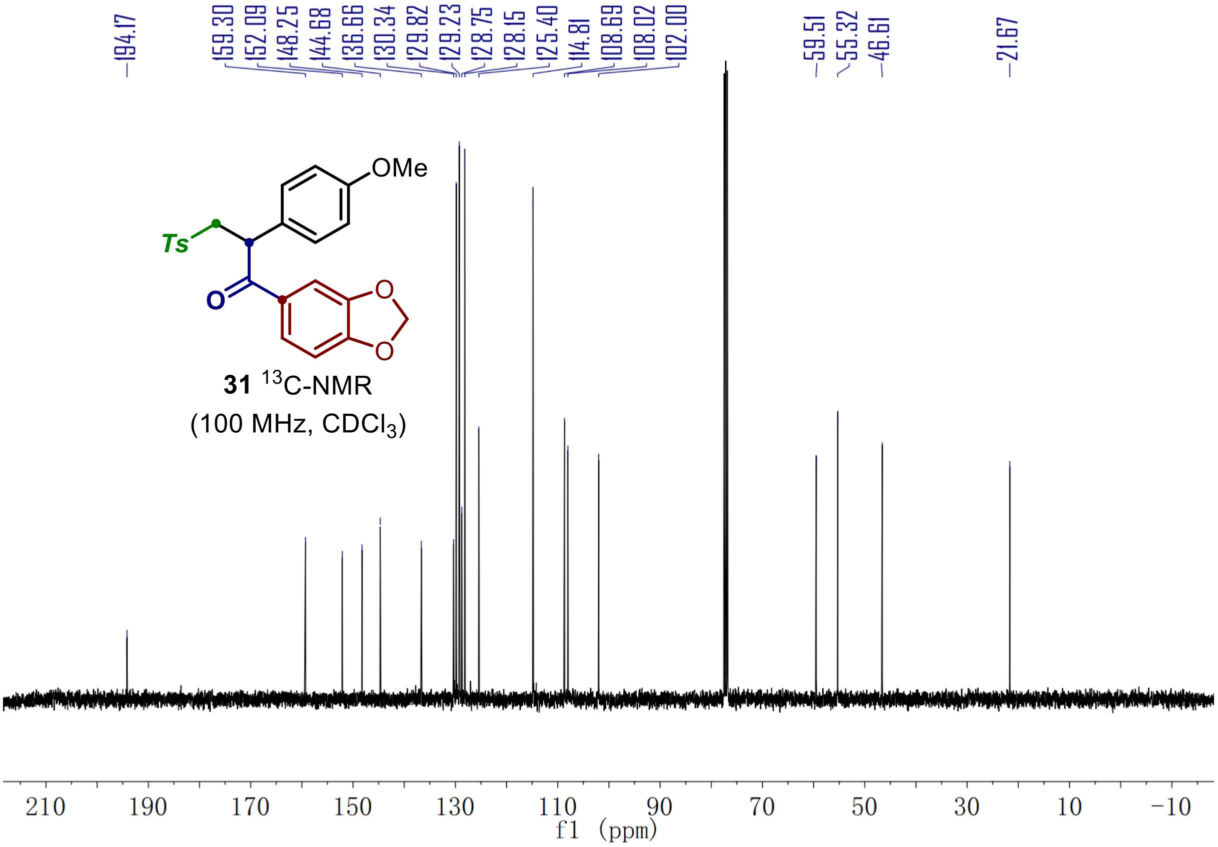
194.17
159.30
152.09
148.25
144.68
136.66
130.34
129.82
129.23
128.75
128.15
125.40
114.81
108.69
108.02
102.00
59.51
55.32
46.61
21.67
OMe
Ts
O
31 13C-NMR
(100 MHz, CDCl3)
210 190 170 150 130 110 90 70 50 30 10 -10
f1 (ppm)

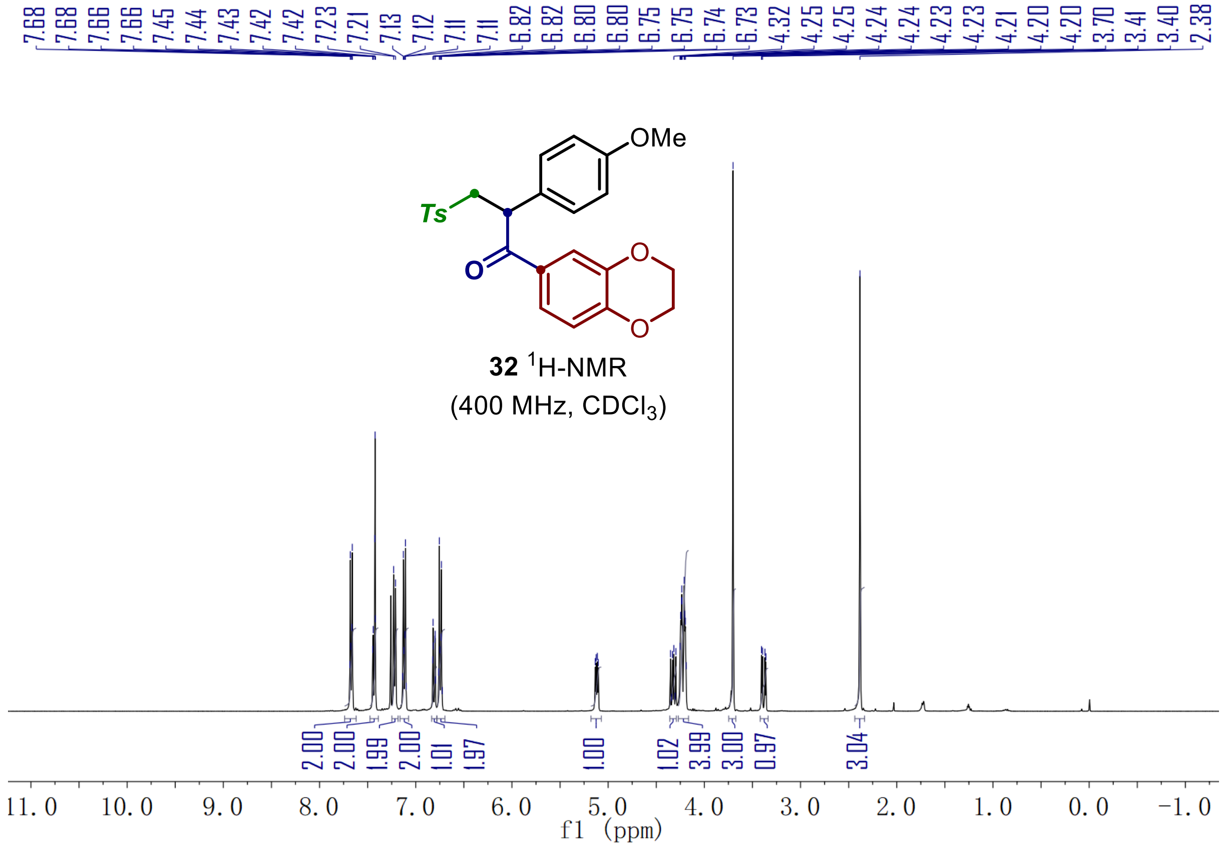
7.68 7.68 7.66 7.66 7.45 7.44 7.43 7.42 7.42 7.23 7.21 7.13 7.12 7.11 7.11 6.82 6.82 6.80 6.80 6.75 6.75 6.74 6.73 4.32 4.25 4.25 4.24 4.24 4.24 4.23 4.23 4.21 4.20 4.20 3.70 3.41 3.40 2.38
OMe
Ts
O
32 1H-NMR
(400 MHz, CDCl3)
2.00 2.00 1.99 2.00 1.01 1.97 1.00 1.02 3.99 3.00 0.97 3.04
11.0 10.0 9.0 8.0 7.0 6.0 5.0 4.0 3.0 2.0 1.0 0.0 -1.0
f1 (ppm)

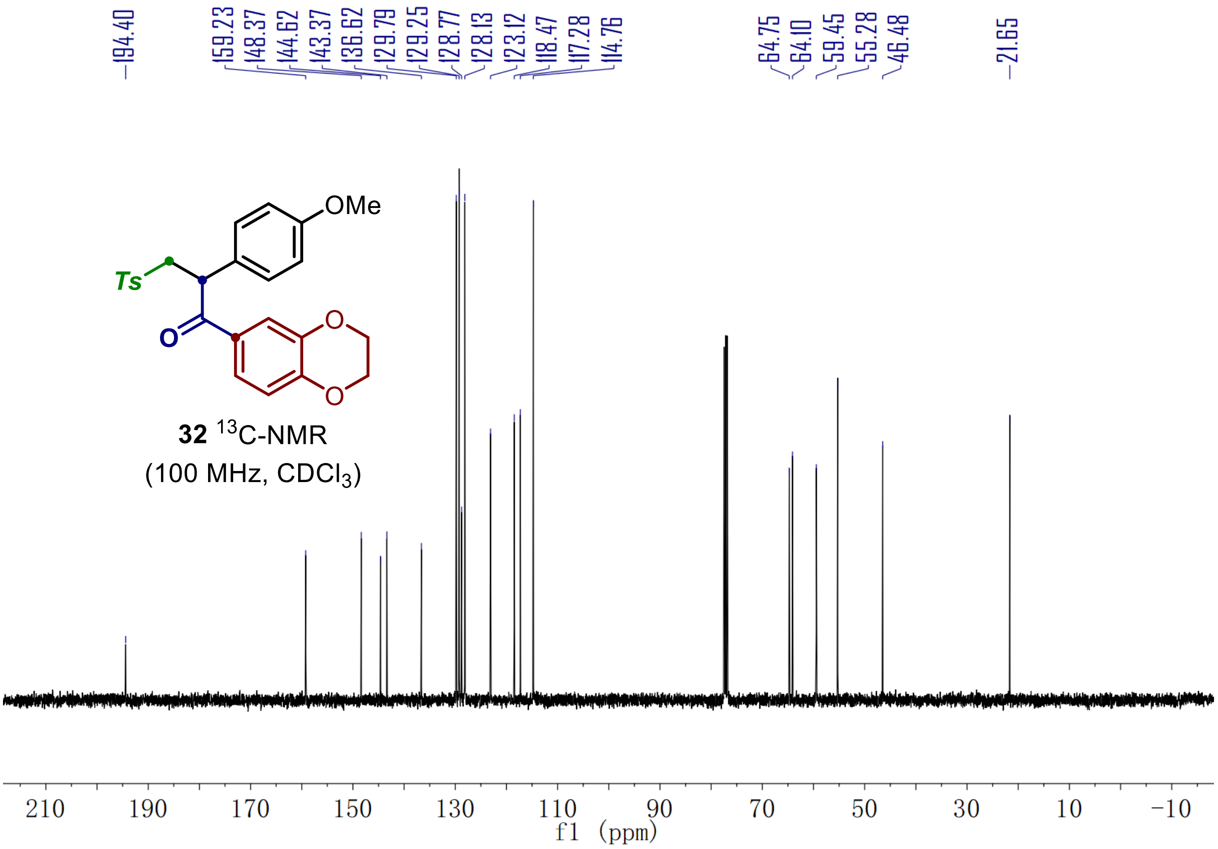
194.40
159.23
148.37
144.62
143.37
136.62
129.79
129.25
128.77
128.13
123.12
118.47
117.28
114.76
64.75
64.10
59.45
55.28
46.48
21.65
OMe
Ts
O
O
O
32 13C-NMR
(100 MHz, CDCl3)
210 190 170 150 130 110 90 70 50 30 10 −10
f1 (ppm)

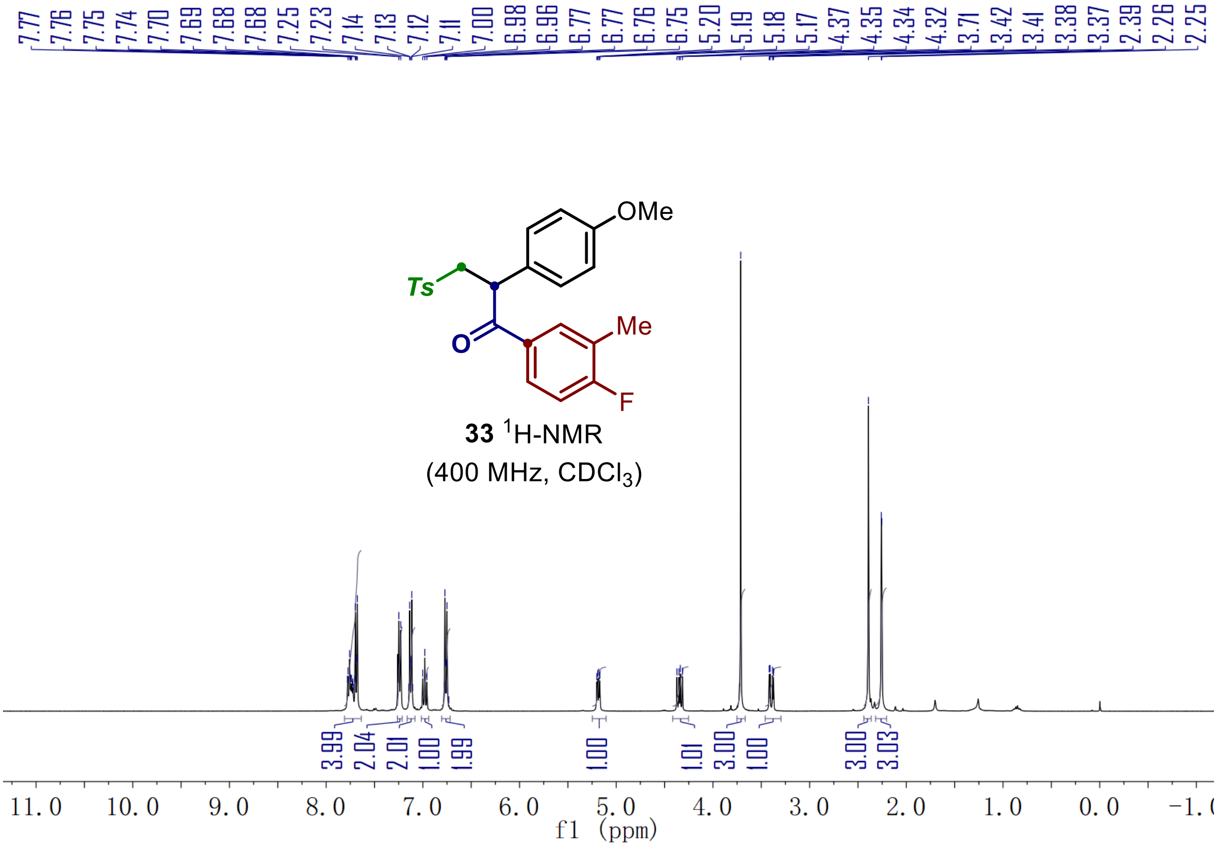
7.77
7.76
7.75
7.74
7.70
7.69
7.68
7.68
7.68
7.25
7.23
7.14
7.13
7.12
7.11
7.00
6.98
6.96
6.77
6.77
6.76
6.75
5.20
5.19
5.18
5.17
4.37
4.35
4.34
4.32
3.71
3.42
3.41
3.38
3.37
2.39
2.26
2.25
OMe
Ts
O
Me
F
33 1H-NMR
(400 MHz, CDCl3)
3.99
2.04
2.01
1.00
1.99
1.00
1.01
3.00
1.00
3.00
3.03
11.0 10.0 9.0 8.0 7.0 6.0 5.0 4.0 3.0 2.0 1.0 0.0
f1 (ppm)

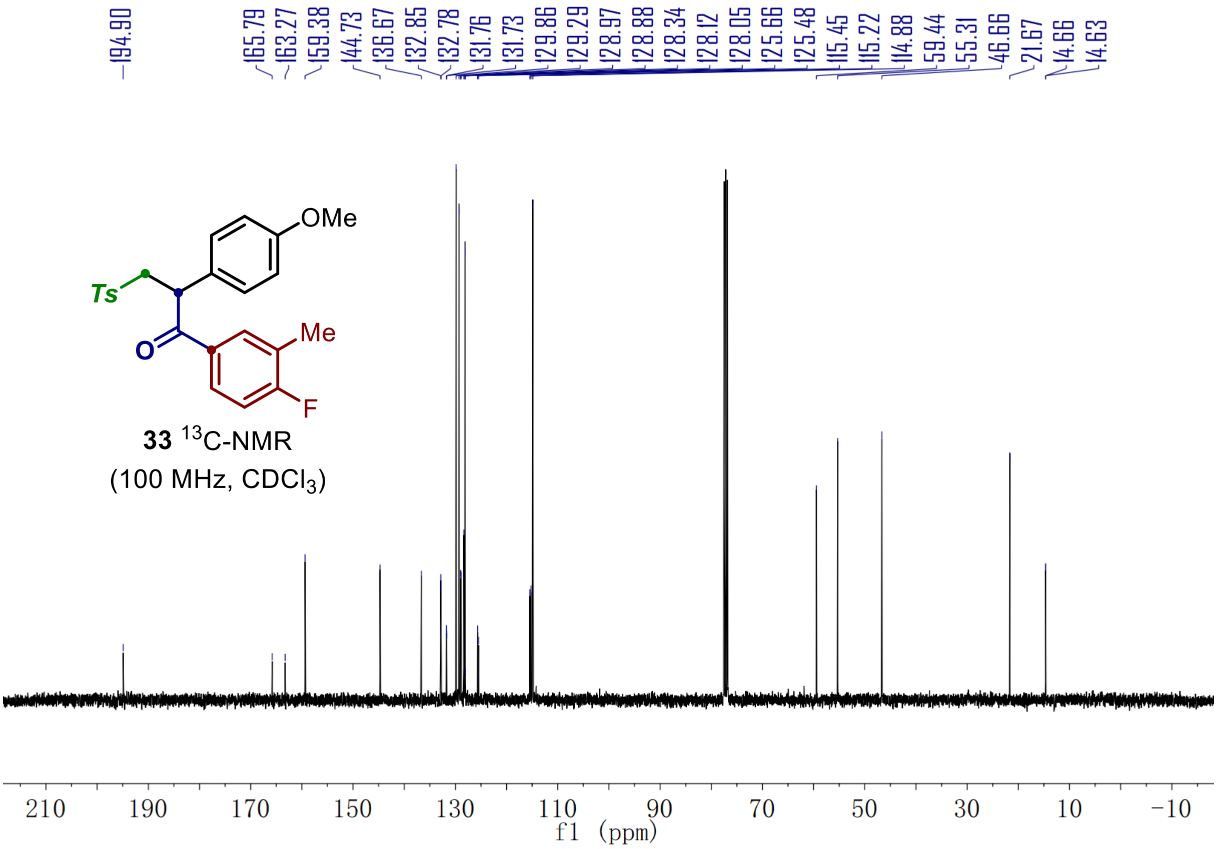
194.90
165.79
163.27
159.38
144.73
136.67
132.85
132.78
131.76
131.73
129.86
129.29
128.97
128.88
128.34
128.12
128.05
125.66
125.48
115.45
115.22
114.88
59.44
55.31
46.66
21.67
14.66
14.63
OMe
Ts
O
Me
F
33 13C-NMR
(100 MHz, CDCl3)
210 190 170 150 130 110 90 70 50 30 10 −10
f1 (ppm)

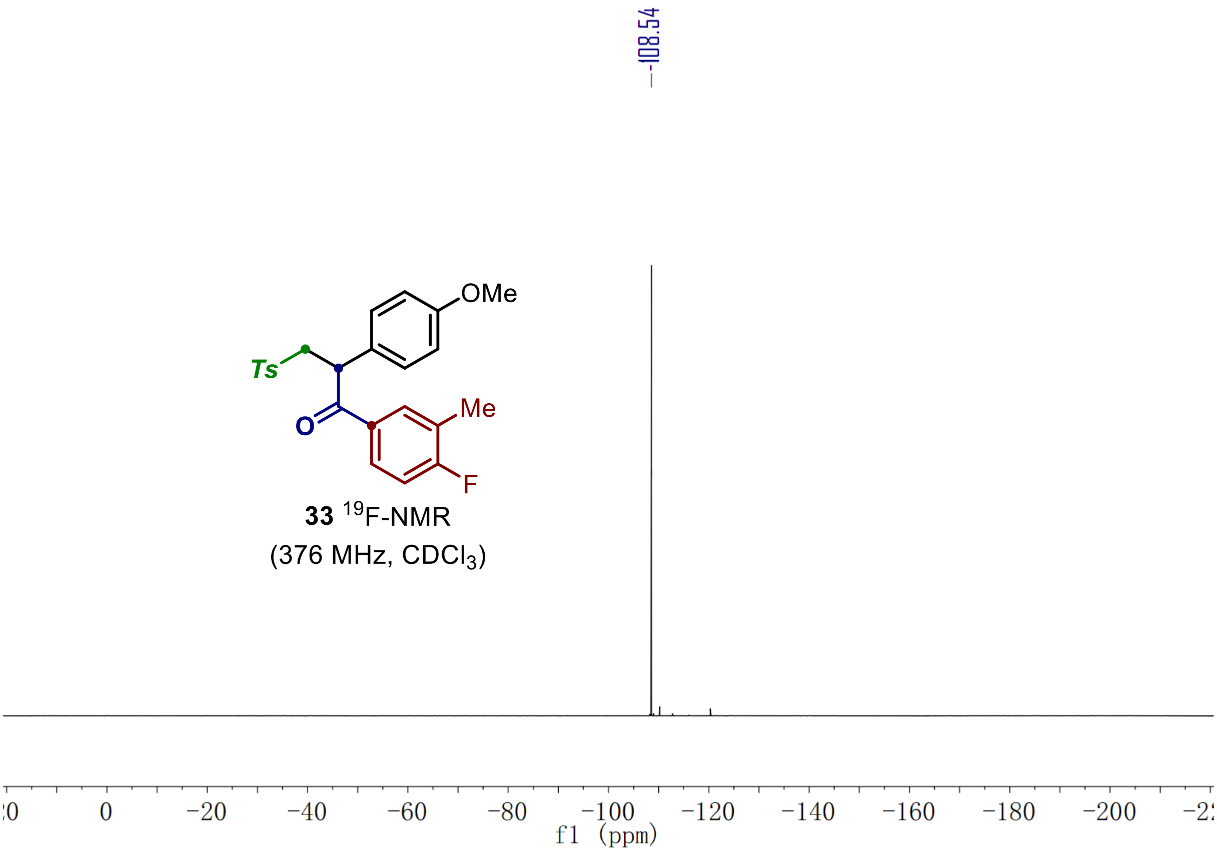
-108.54
OMe
Ts
O
Me
F
33 19F-NMR
(376 MHz, CDCl3)
0 −20 −40 −60 −80 −100 −120 −140 −160 −180 −200
f1 (ppm)

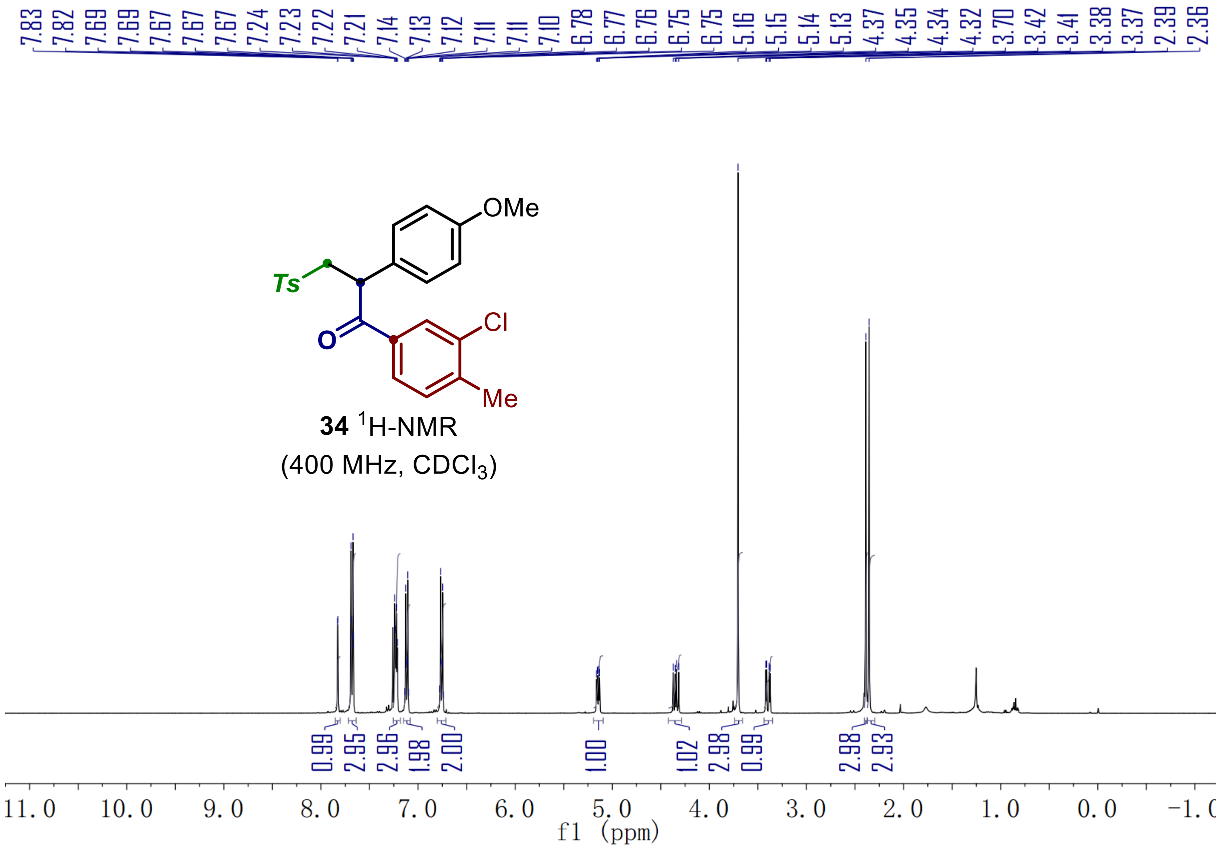
7.83 7.82 7.69 7.69 7.67 7.67 7.67 7.24 7.23 7.22 7.21 7.14 7.13 7.12 7.11 7.11 7.10 6.78 6.77 6.76 6.75 6.75 5.16 5.15 5.14 5.13 4.37 4.35 4.34 4.32 3.70 3.42 3.41 3.38 3.37 2.39 2.36
OMe
Ts
O
Cl
Me
34 1H-NMR
(400 MHz, CDCl3)
0.99 2.95 2.96 1.98 2.00 1.00 1.02 2.98 0.99 2.98 2.93
11.0 10.0 9.0 8.0 7.0 6.0 5.0 4.0 3.0 2.0 1.0 0.0 -1.0
f1 (ppm)

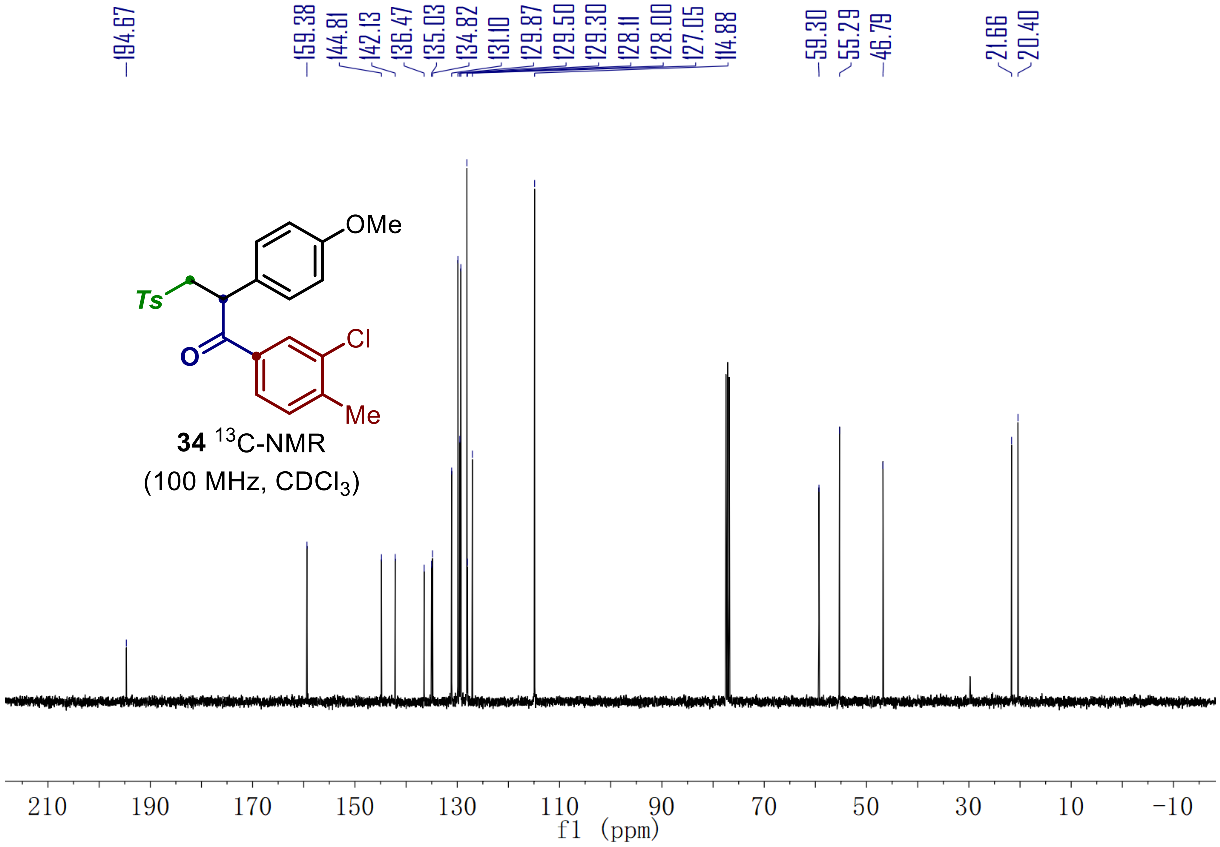
194.67
159.38 144.81 142.13 136.47 135.03 134.82 131.10 129.87 129.50 129.30 128.11 128.00 127.05 114.88
59.30 55.29 46.79
21.66 20.40
OMe
Ts
O
Cl
Me
34 13C-NMR
(100 MHz, CDCl3)
210 190 170 150 130 110 90 70 50 30 10 -10
f1 (ppm)

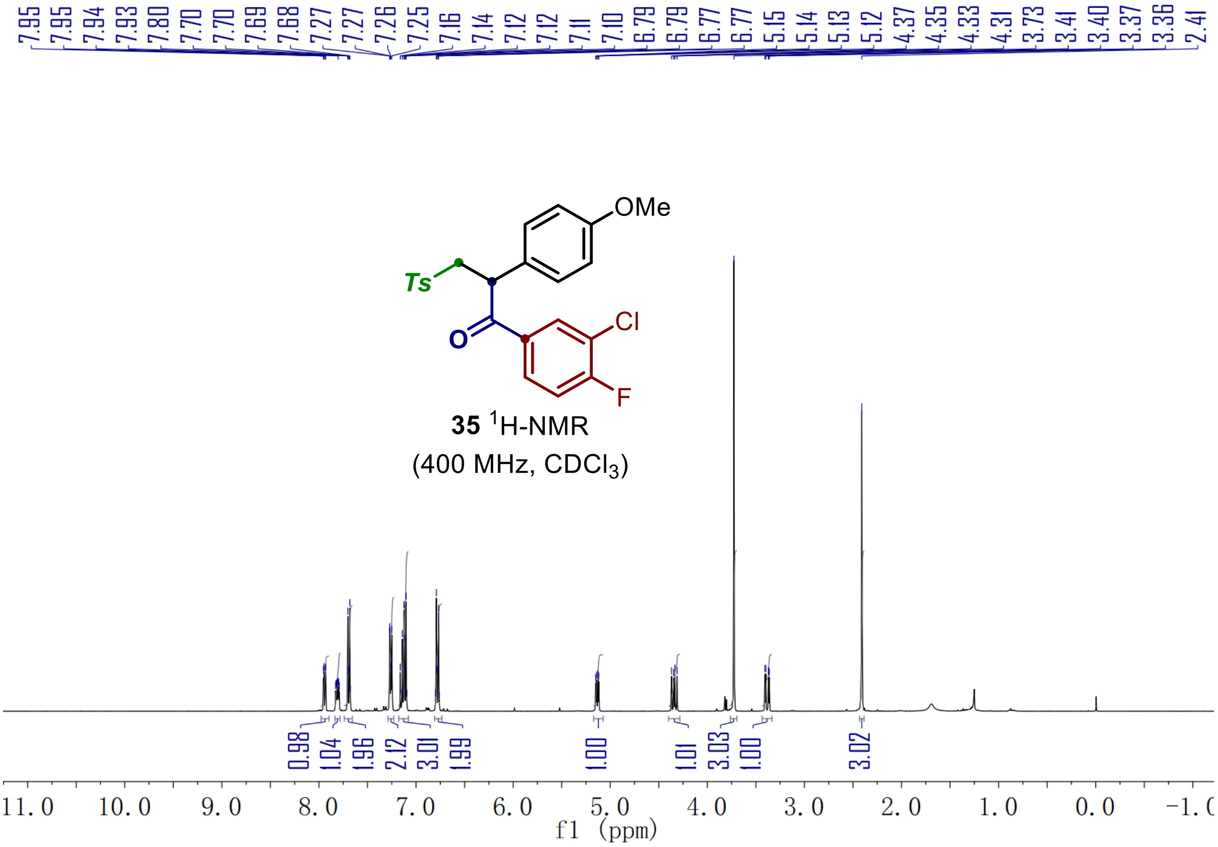
7.95 7.95 7.94 7.93 7.80 7.70 7.70 7.69 7.68 7.27 7.27 7.26 7.25 7.16 7.14 7.12 7.12 7.11 7.10 6.79 6.79 6.77 6.77 5.15 5.14 5.13 5.12 4.37 4.35 4.33 4.31 3.73 3.41 3.40 3.37 3.36 2.41
OMe
Ts
O
Cl
F
35 ¹H-NMR
(400 MHz, CDCl3)
0.98 1.04 1.96 2.12 3.01 1.99 1.00 1.01 3.03 1.00 3.02
11.0 10.0 9.0 8.0 7.0 6.0 5.0 4.0 3.0 2.0 1.0 0.0 -1.0
f1 (ppm)

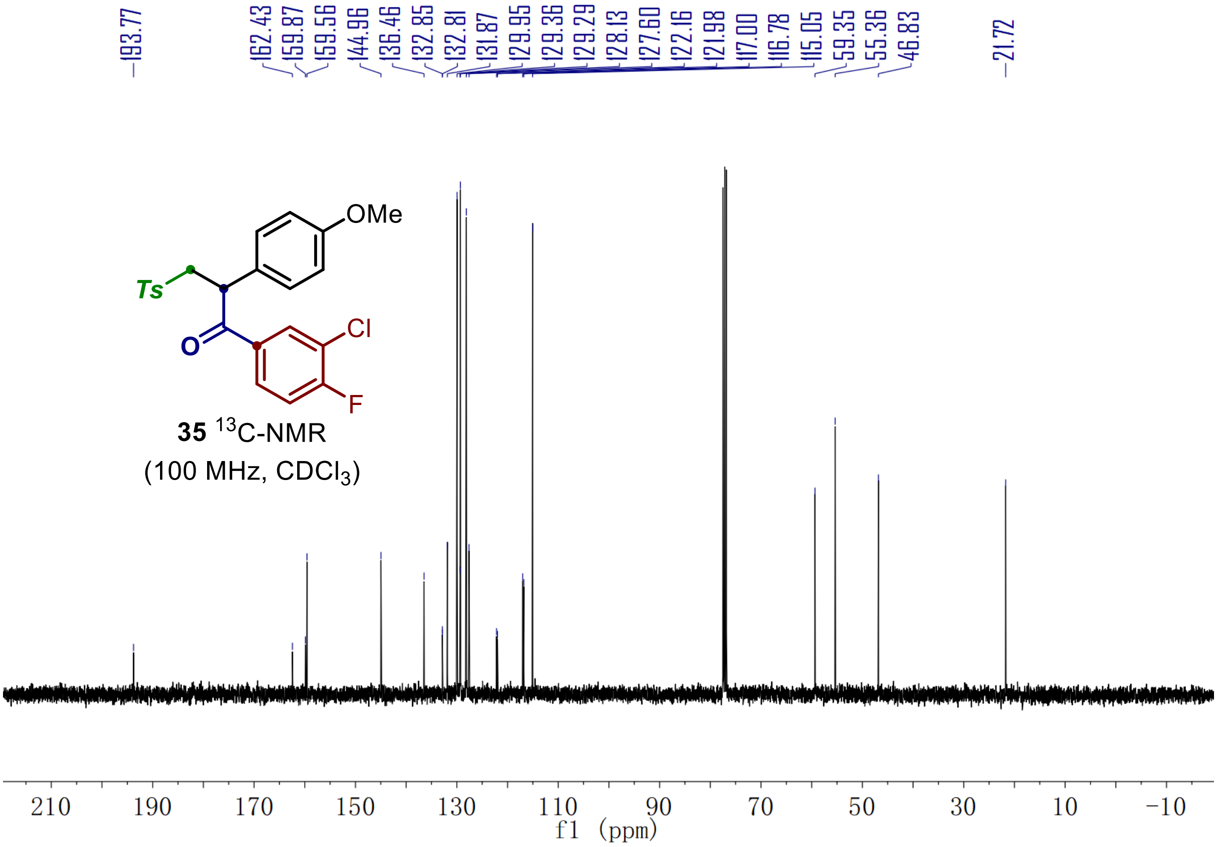
193.77
162.43 159.87 159.56 144.96 136.46 132.85 132.81 131.87 129.95 129.36 129.29 128.13 127.60 122.16 121.98 117.00 116.78 115.05 59.35 55.36 46.83
21.72
OMe
Ts
O
Cl
F
35 ¹³C-NMR
(100 MHz, CDCl3)
210 190 170 150 130 110 90 70 50 30 10 -10
f1 (ppm)

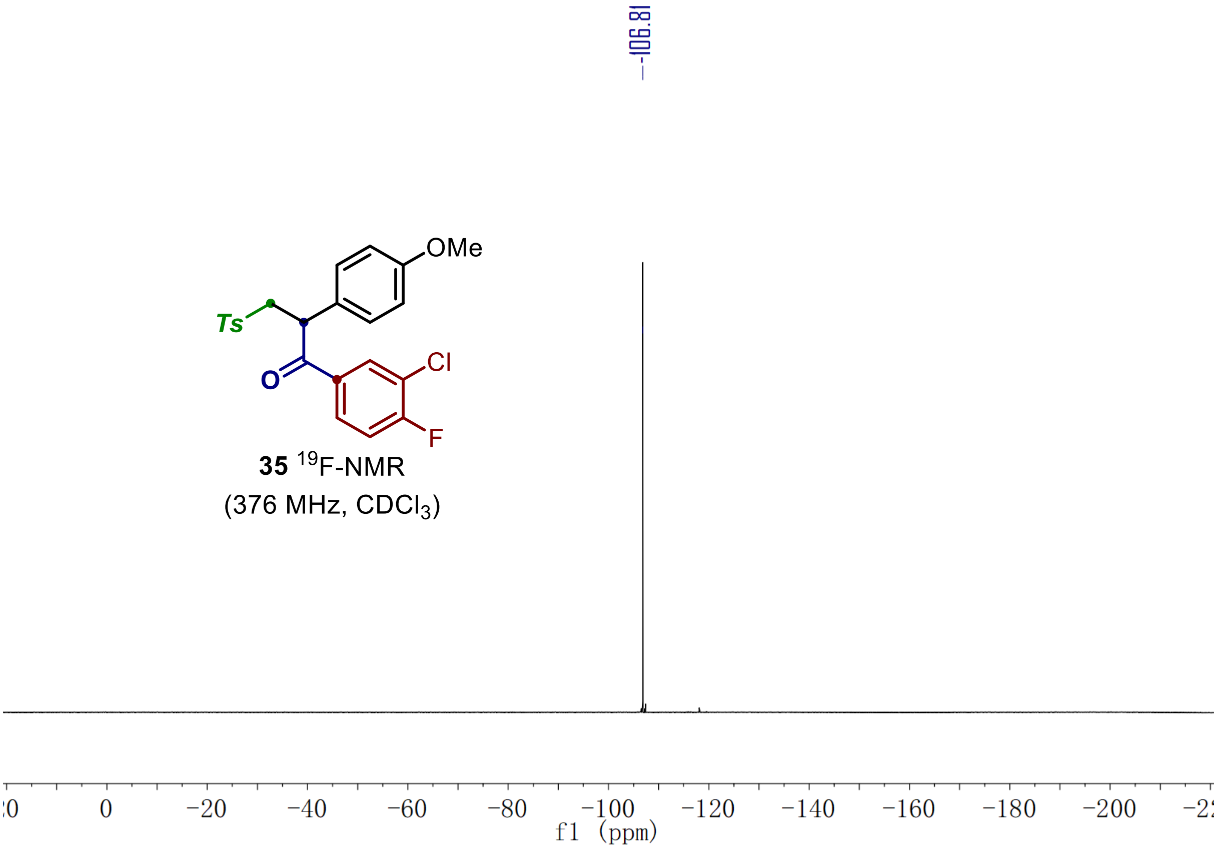

35 ^{19}F-NMR
(376 MHz, $CDCl_3$)

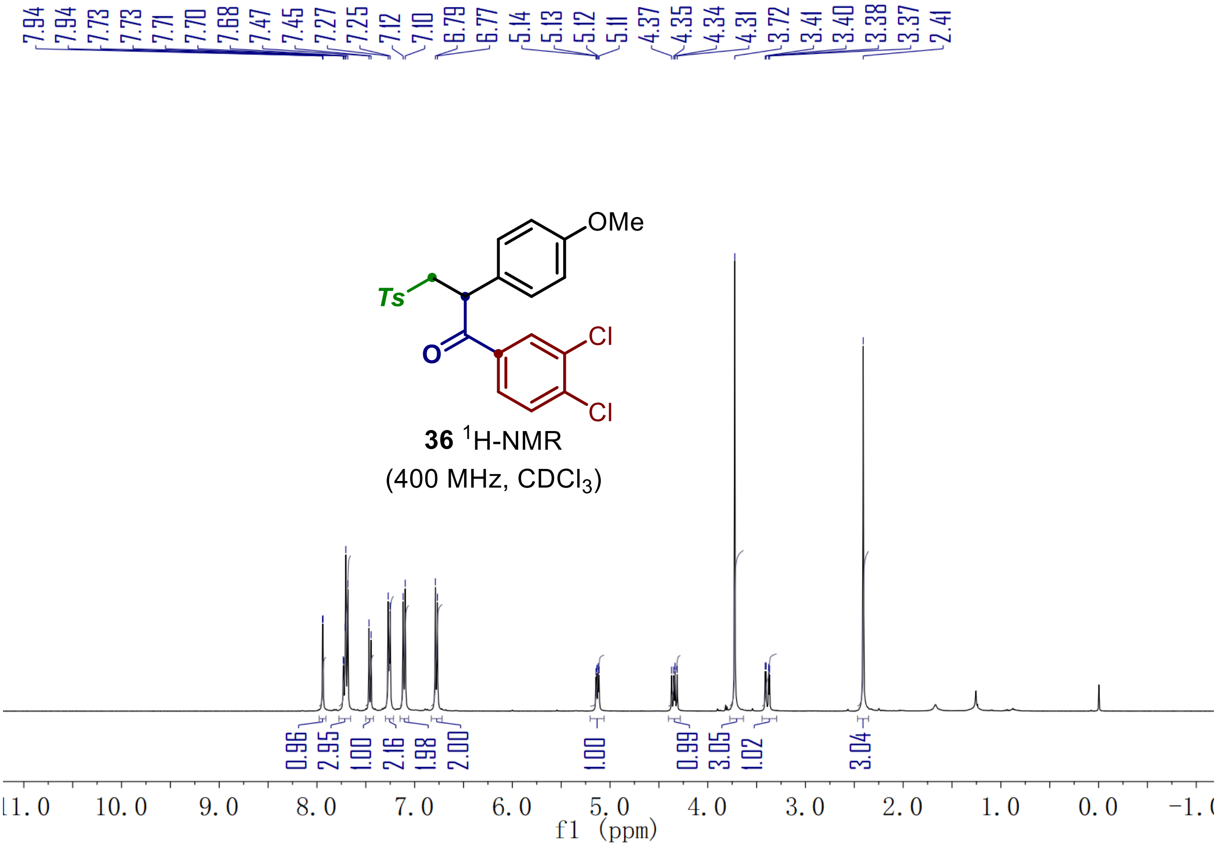

36 ^{1}H-NMR
(400 MHz, $CDCl_3$)

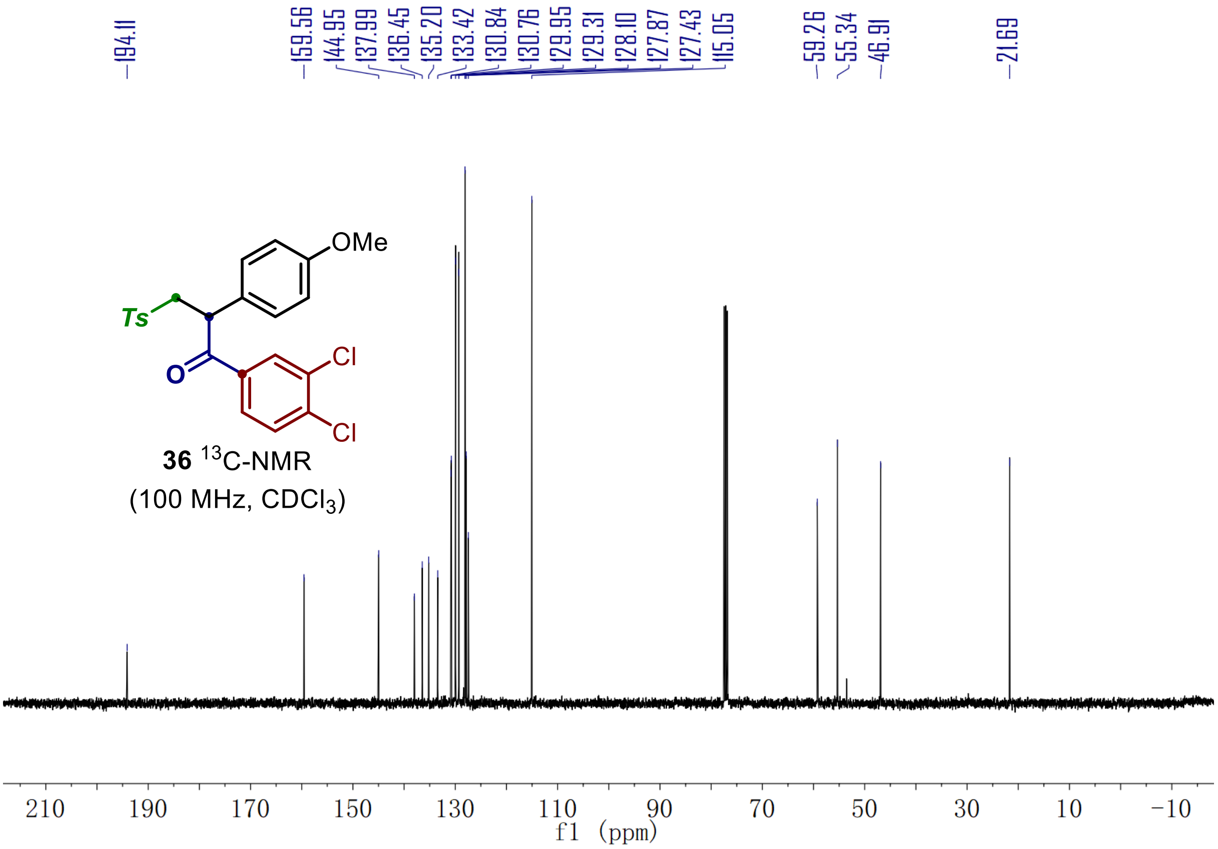
194.11
159.56
144.95
137.99
136.45
135.20
133.42
130.84
130.76
129.95
129.31
128.10
127.87
127.43
115.05
59.26
55.34
46.91
21.69
OMe
Ts
O
Cl
Cl
36 13C-NMR
(100 MHz, CDCl3)
210 190 170 150 130 110 90 70 50 30 10 −10
f1 (ppm)

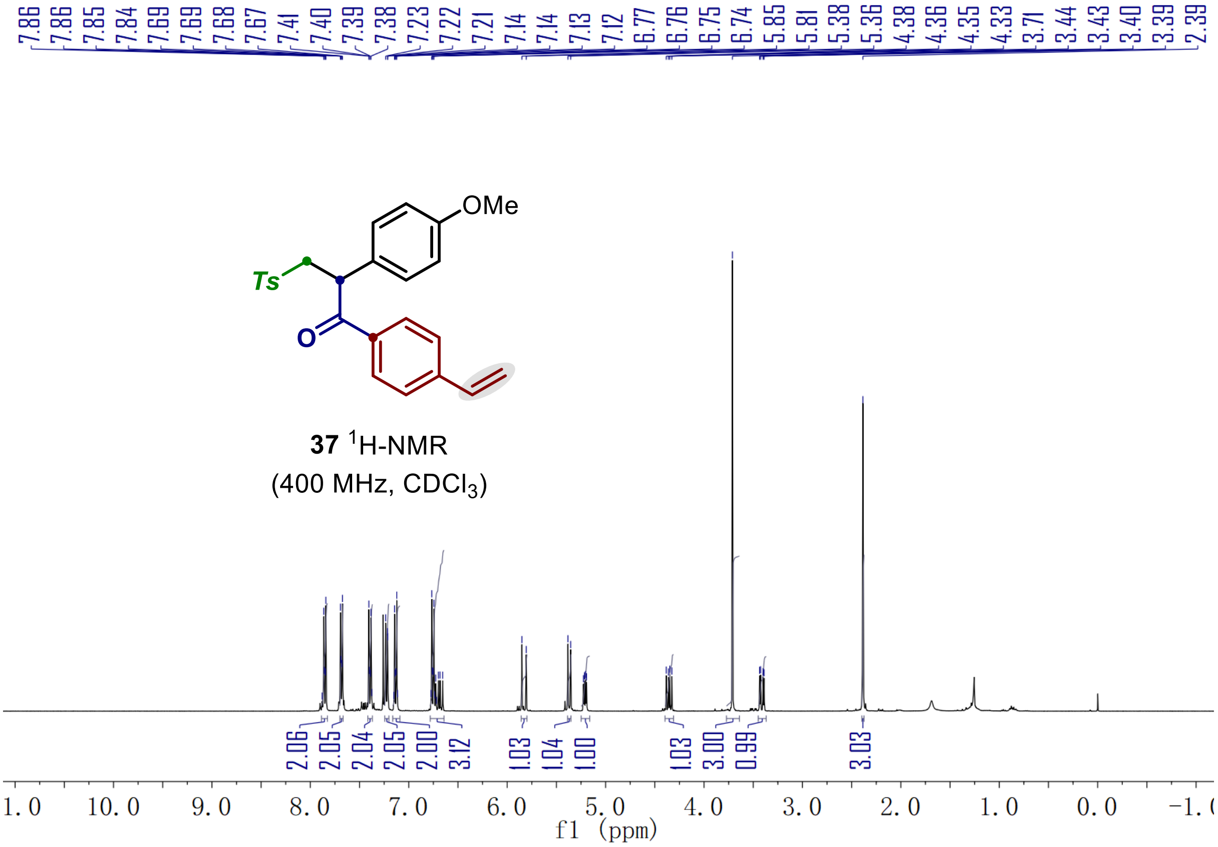
7.86 7.86 7.85 7.84 7.69 7.69 7.68 7.67 7.41 7.40 7.39 7.38 7.23 7.22 7.21 7.14 7.14 7.13 7.12 6.77 6.76 6.75 6.74 5.85 5.81 5.38 5.36 4.38 4.36 4.35 4.33 3.71 3.44 3.43 3.40 3.39 2.39
OMe
Ts
O
37 1H-NMR
(400 MHz, CDCl3)
2.06 2.05 2.04 2.05 2.00 3.12 1.03 1.04 1.00 1.03 3.00 0.99 3.03
10.0 9.0 8.0 7.0 6.0 5.0 4.0 3.0 2.0 1.0 0.0
f1 (ppm)

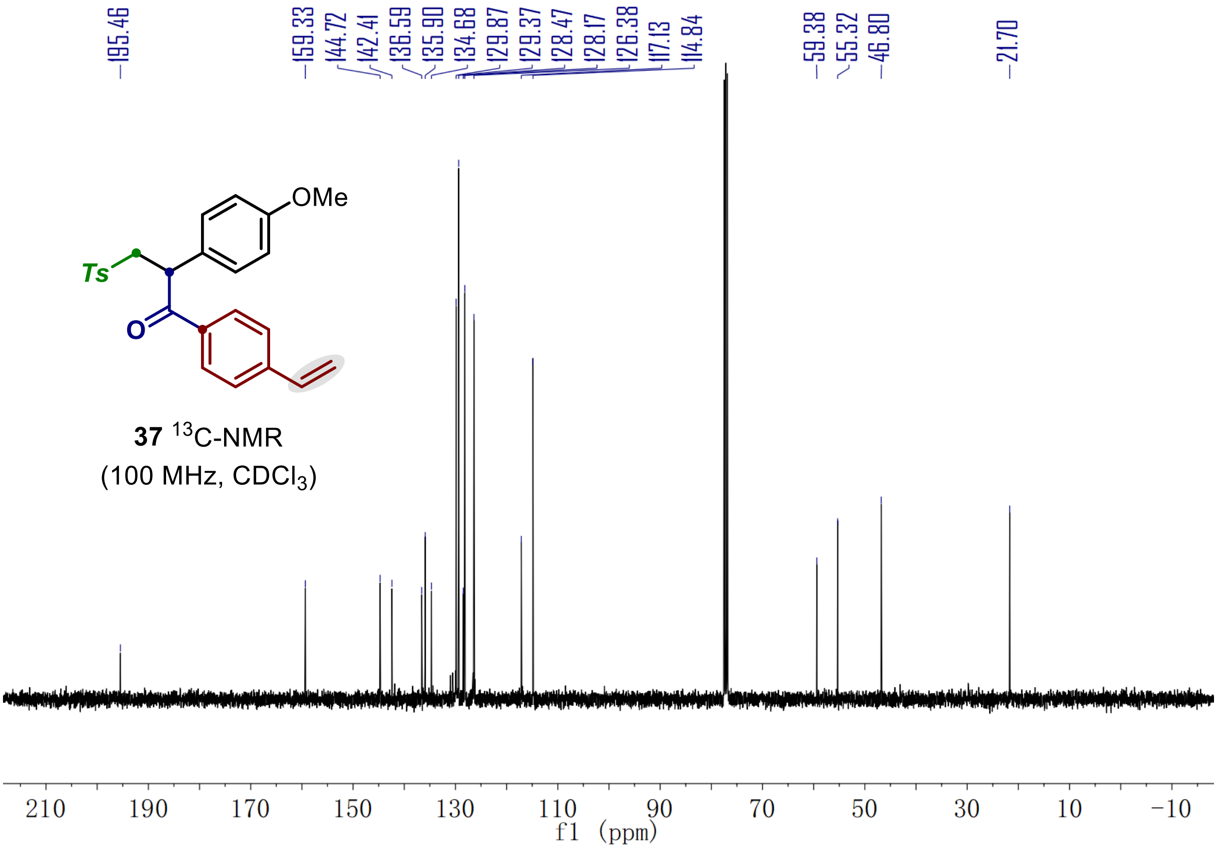
195.46
159.33
144.72
142.41
136.59
135.90
134.68
129.87
129.37
128.47
128.17
126.38
117.13
114.84
59.38
55.32
46.80
21.70
OMe
Ts
O
37 ^{13}C-NMR
(100 MHz, $CDCl_3$)
210 190 170 150 130 110 90 70 50 30 10 −10
f1 (ppm)

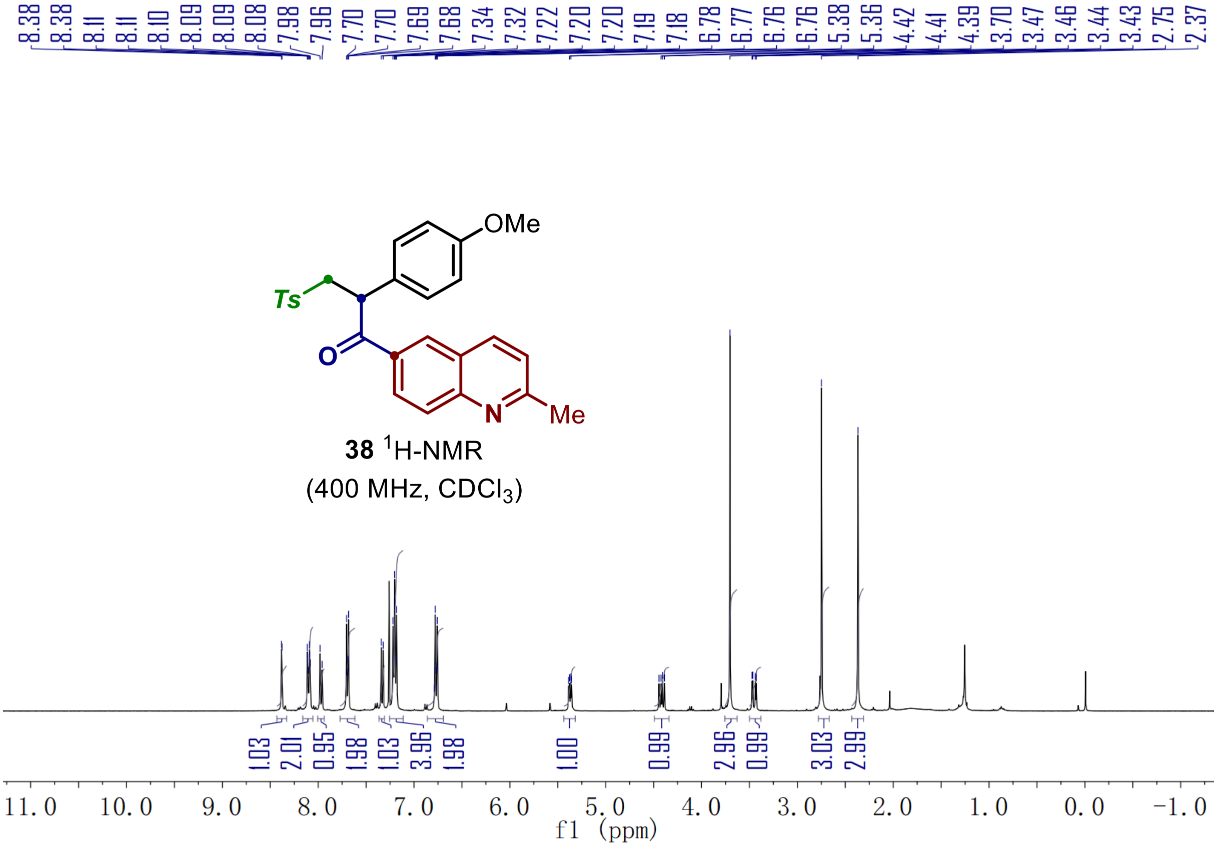
8.38 8.38 8.11 8.11 8.10 8.09 8.09 8.08 7.98 7.96 7.70 7.70 7.69 7.68 7.34 7.32 7.22 7.20 7.20 7.19 7.18 6.78 6.77 6.76 6.76 5.38 5.36 4.42 4.41 4.39 3.70 3.47 3.46 3.44 3.43 2.75 2.37
OMe
Ts
O
N
Me
38 ^{1}H-NMR
(400 MHz, $CDCl_3$)
1.03 2.01 0.95 1.98 1.03 3.96 1.98 1.00 0.99 2.96 0.99 3.03 2.99
11.0 10.0 9.0 8.0 7.0 6.0 5.0 4.0 3.0 2.0 1.0 0.0 −1.0
f1 (ppm)

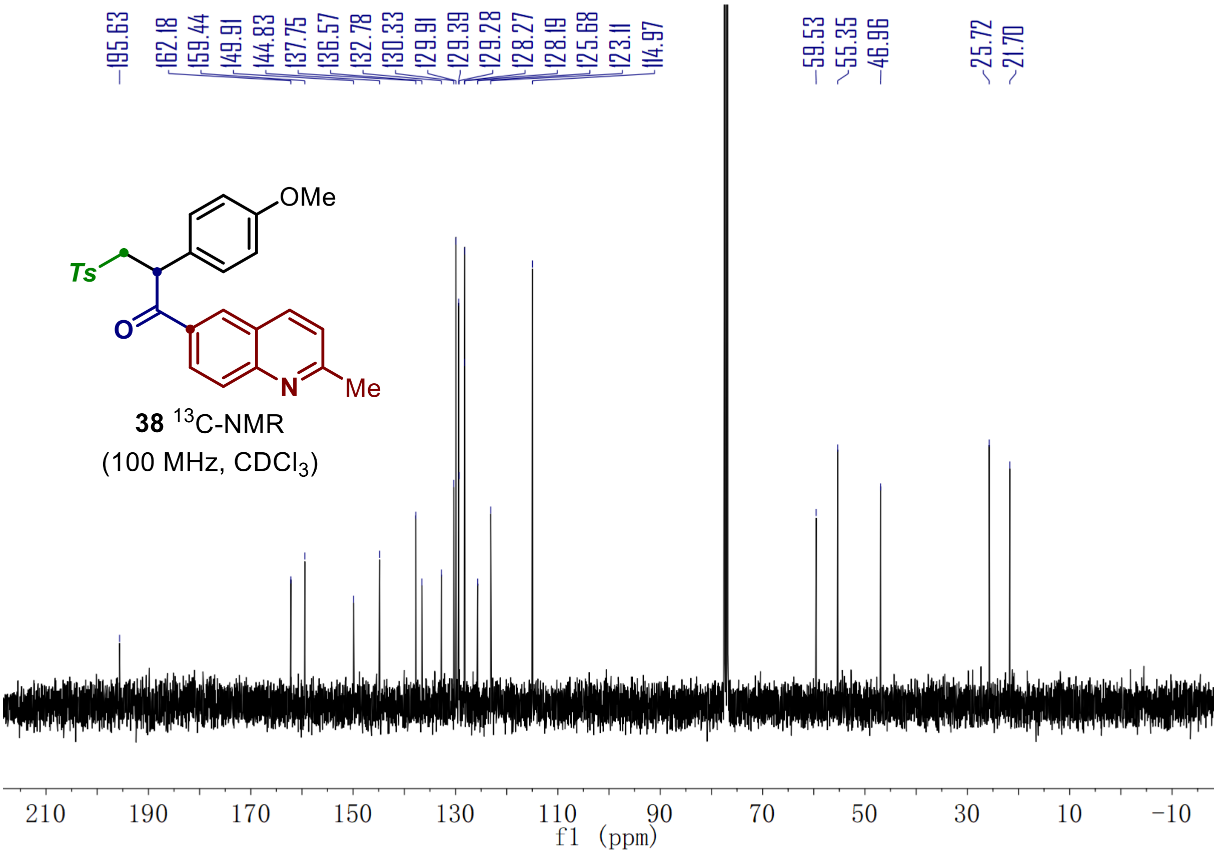
195.63
162.18 159.44 149.91 144.83 137.75 136.57 132.78 130.33 129.91 129.39 129.28 128.27 128.19 125.68 123.11 114.97
59.53 55.35 46.96
25.72 21.70
OMe
Ts
O
N
Me
38 13C-NMR
(100 MHz, CDCl3)
210 190 170 150 130 110 90 70 50 30 10 -10
f1 (ppm)

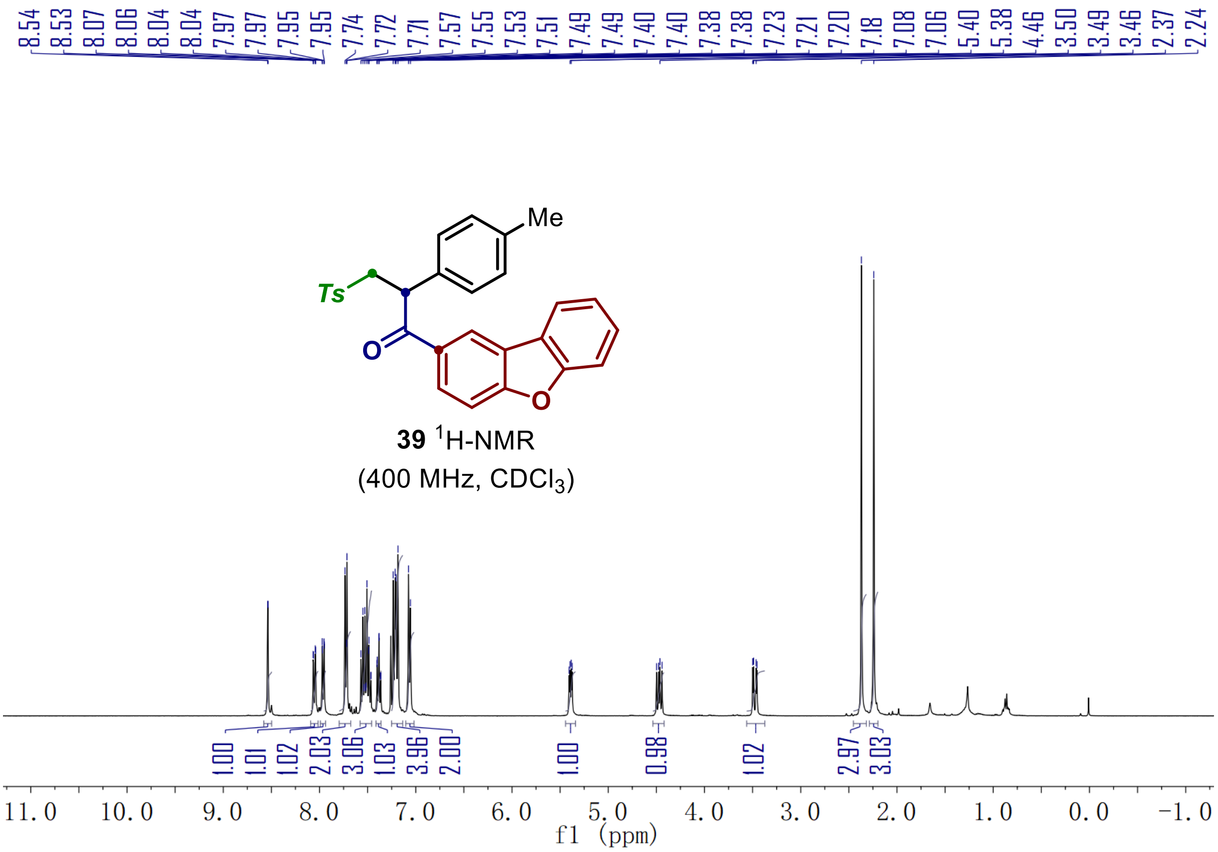
8.54 8.53 8.07 8.06 8.04 8.04 7.97 7.97 7.95 7.95 7.74 7.72 7.71 7.57 7.55 7.53 7.51 7.49 7.49 7.40 7.40 7.38 7.38 7.23 7.21 7.20 7.18 7.08 7.06 5.40 5.38 4.46 3.50 3.49 3.46 2.37 2.24
Me
Ts
O
O
39 1H-NMR
(400 MHz, CDCl3)
1.00 1.01 1.02 2.03 3.06 1.03 3.96 2.00 1.00 0.98 1.02 2.97 3.03
11.0 10.0 9.0 8.0 7.0 6.0 5.0 4.0 3.0 2.0 1.0 0.0 -1.0
f1 (ppm)

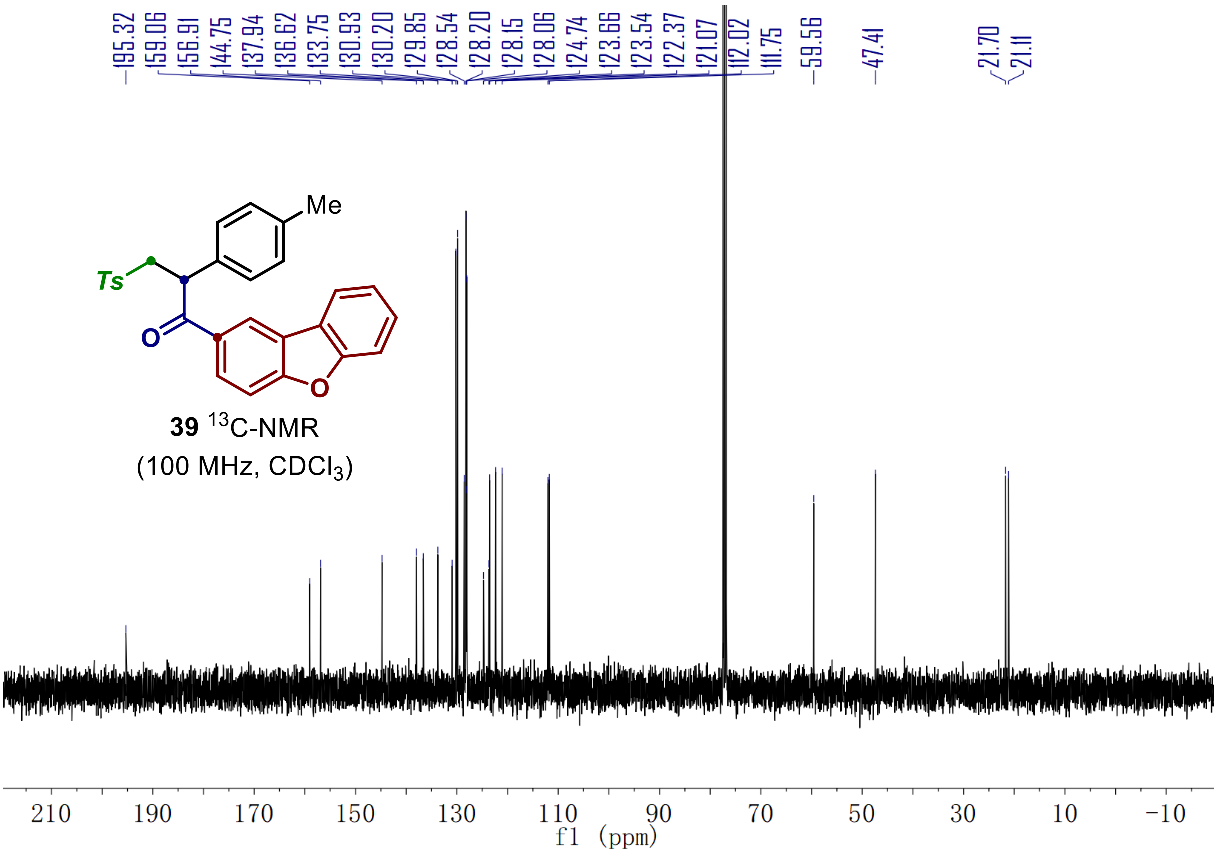
195.32
159.06
156.91
144.75
137.94
136.62
133.75
130.93
130.20
129.85
128.54
128.20
128.15
128.06
124.74
123.66
123.54
122.37
121.07
112.02
111.75
59.56
47.41
21.70
21.11
Me
Ts
O
O
39 ^{13}C-NMR
(100 MHz, CDCl$_3$)
210 190 170 150 130 110 90 70 50 30 10 −10
f1 (ppm)

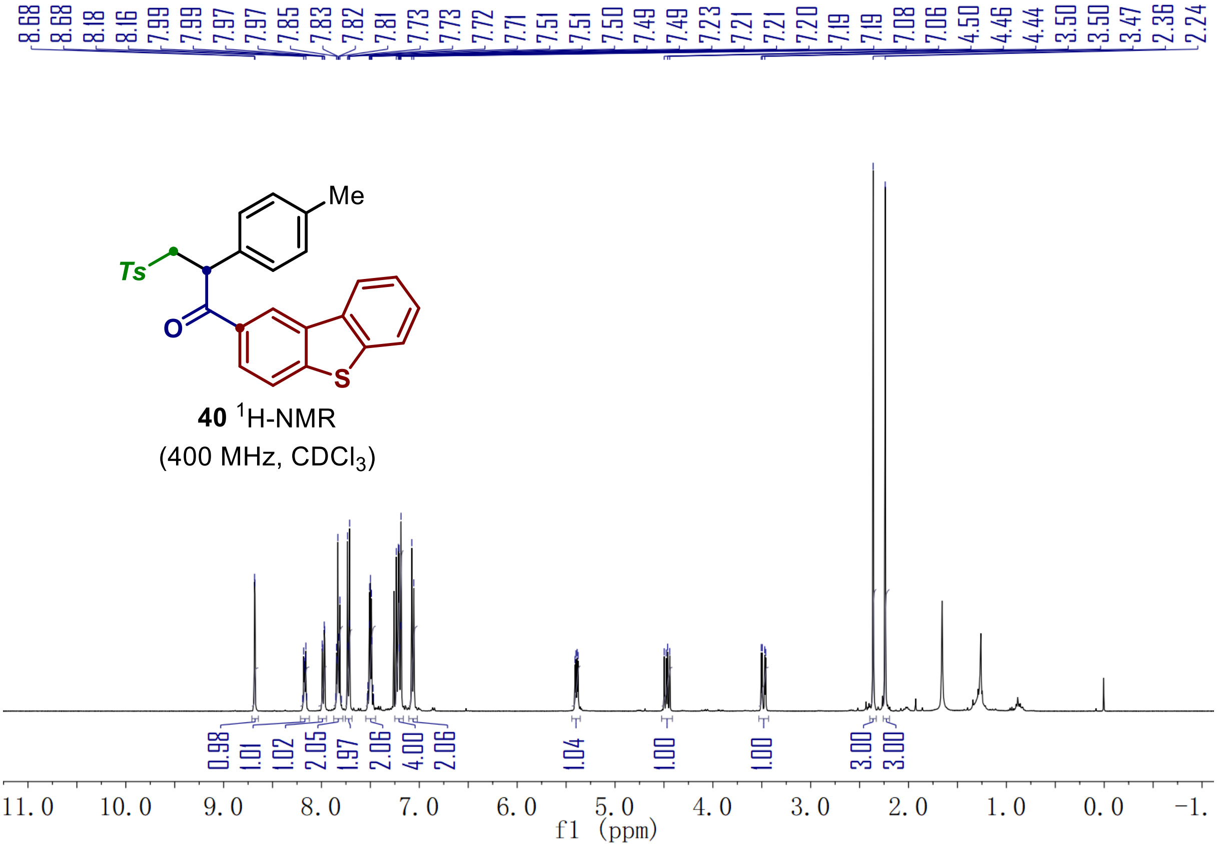
8.68
8.68
8.18
8.16
7.99
7.99
7.97
7.97
7.85
7.83
7.82
7.81
7.73
7.73
7.72
7.71
7.51
7.51
7.50
7.49
7.49
7.23
7.21
7.21
7.20
7.19
7.19
7.08
7.06
4.50
4.46
4.44
3.50
3.50
3.47
2.36
2.24
Me
Ts
O
S
40 ^{1}H-NMR
(400 MHz, CDCl$_3$)
0.98
1.01
1.02
2.05
1.97
2.06
4.00
2.06
1.04
1.00
1.00
3.00
3.00
11.0 10.0 9.0 8.0 7.0 6.0 5.0 4.0 3.0 2.0 1.0 0.0 −1.
f1 (ppm)

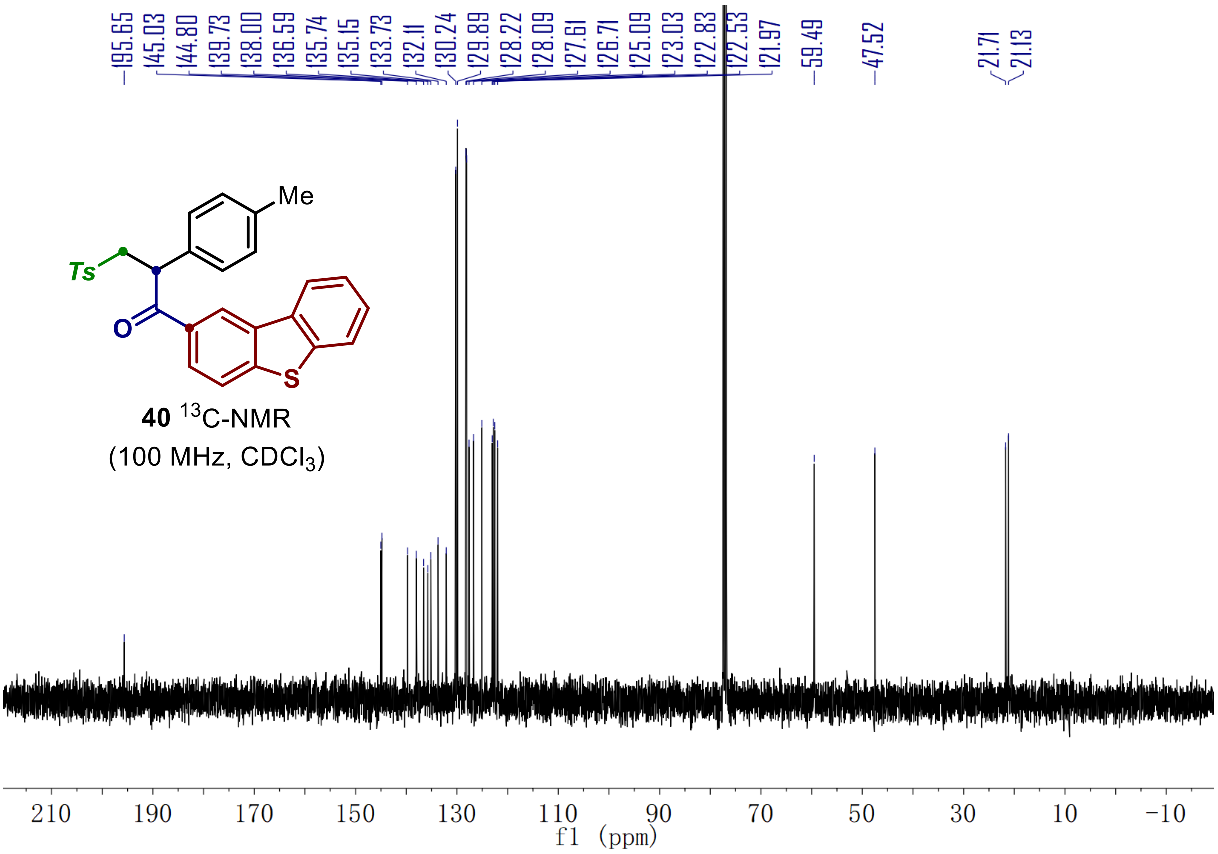
195.65
145.03
144.80
139.73
138.00
136.59
135.74
135.15
133.73
132.11
130.24
129.89
128.22
128.09
127.61
126.71
125.09
123.03
122.83
122.53
121.97
59.49
47.52
21.71
21.13
Me
Ts
O
S
40 13C-NMR
(100 MHz, CDCl3)
210 190 170 150 130 110 90 70 50 30 10 −10
f1 (ppm)

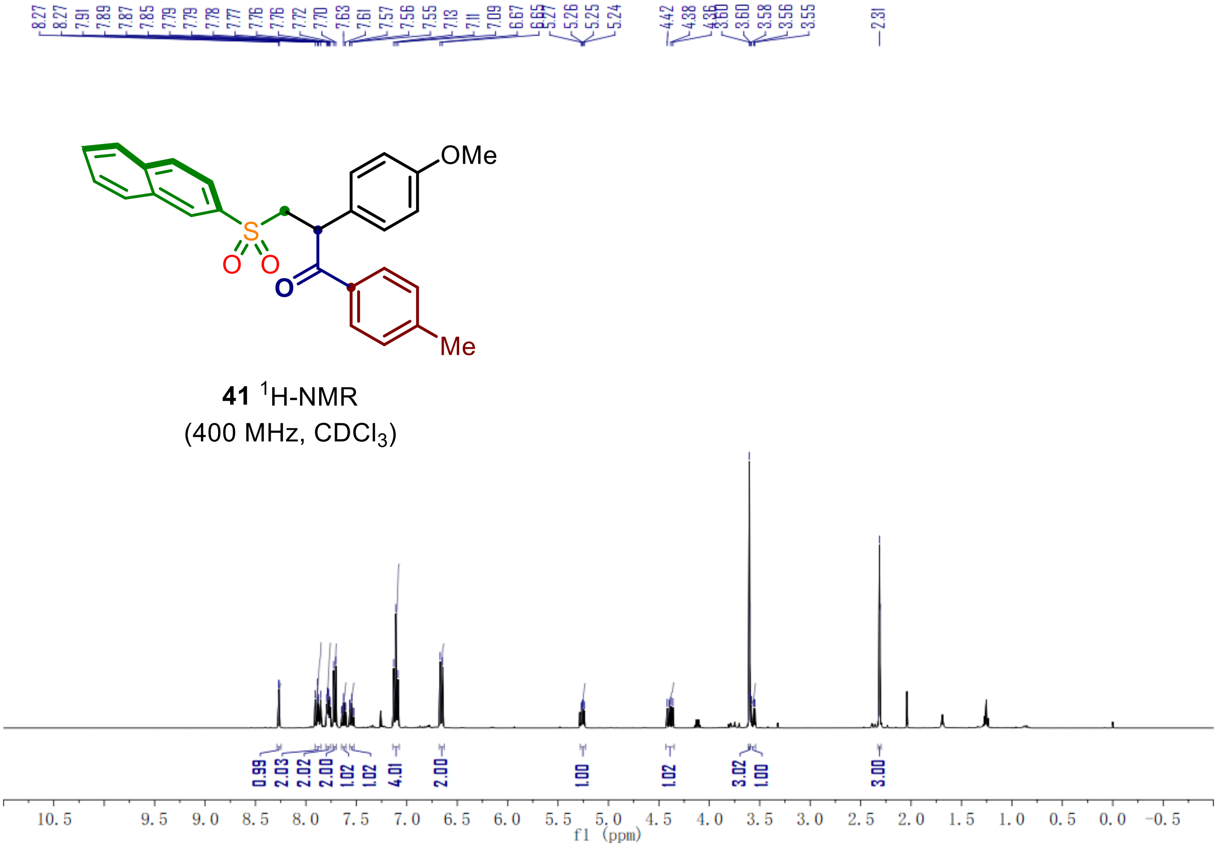
8.27
8.27
7.91
7.89
7.87
7.85
7.79
7.79
7.78
7.77
7.76
7.76
7.72
7.70
7.63
7.61
7.57
7.56
7.55
7.13
7.11
7.09
6.67
6.65
5.27
5.26
5.25
5.24
4.42
4.38
4.36
3.60
3.60
3.58
3.56
3.55
2.31
OMe
S
O
O
O
Me
41 1H-NMR
(400 MHz, CDCl3)
0.99
2.03
2.02
2.00
1.02
1.02
4.01
2.00
1.00
1.02
3.02
1.00
3.00
10.5 9.5 9.0 8.5 8.0 7.5 7.0 6.5 6.0 5.5 5.0 4.5 4.0 3.5 3.0 2.5 2.0 1.5 1.0 0.5 0.0 −0.5
f1 (ppm)

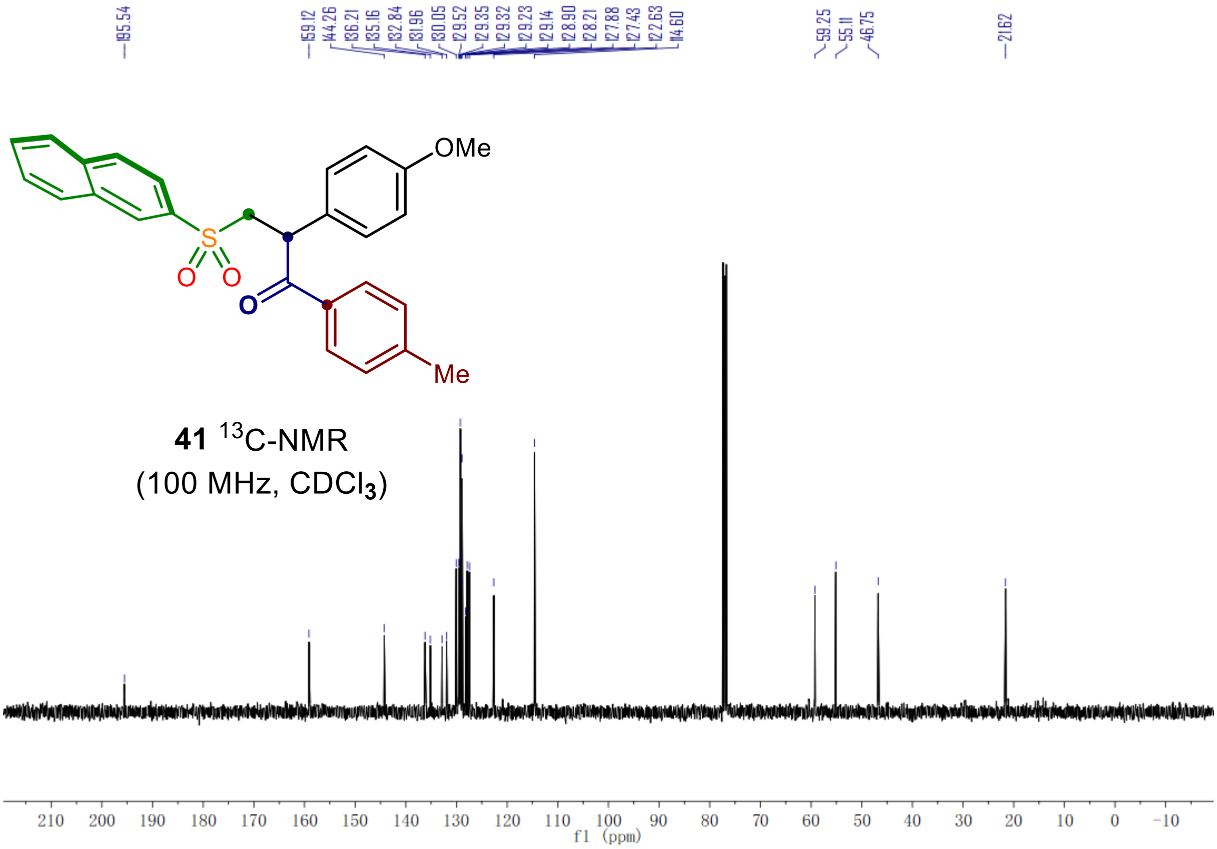
195.54
159.12
144.26
136.21
135.16
132.84
131.96
130.05
129.52
129.35
129.32
129.23
129.14
128.90
128.21
127.88
127.43
122.63
114.60
59.25
55.11
46.75
21.62
OMe
S
O
O
O
Me
41 ^{13}C-NMR
(100 MHz, $CDCl_3$)
f1 (ppm)

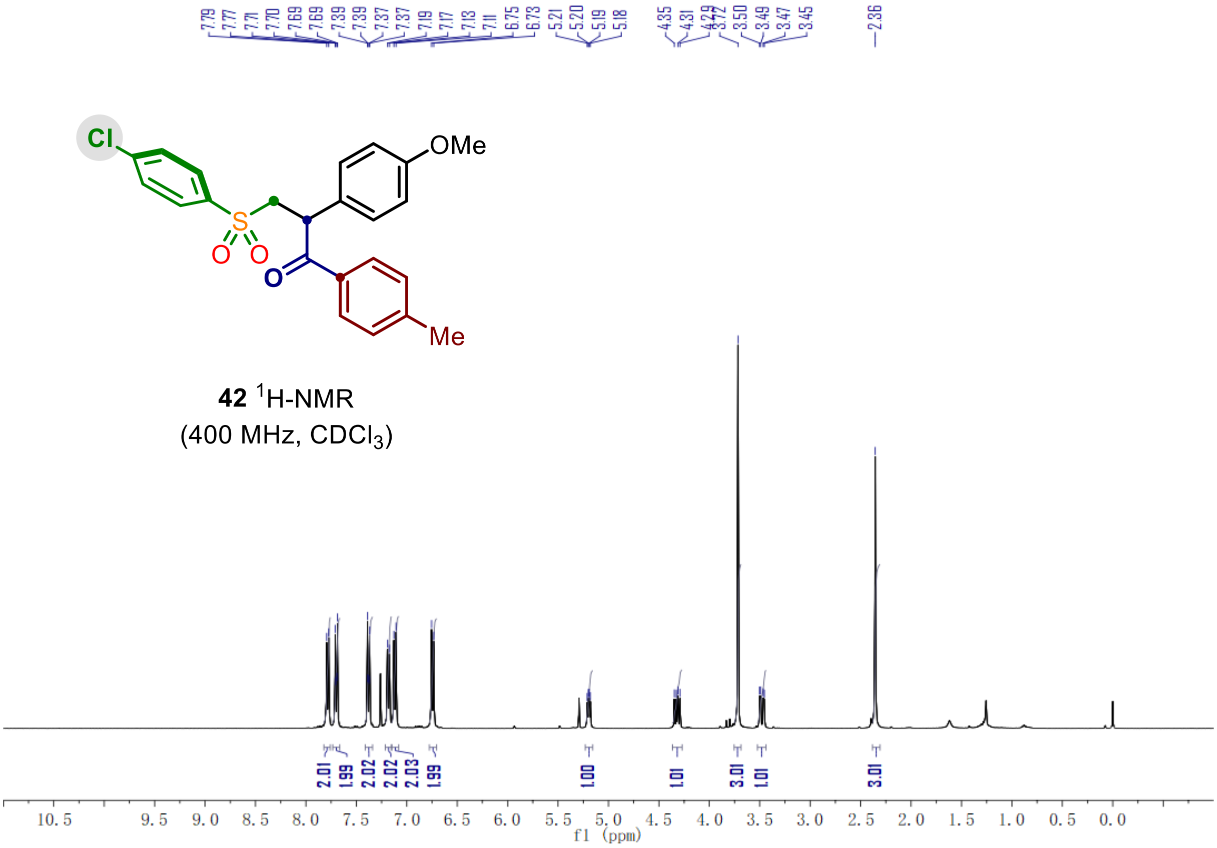
7.79
7.77
7.71
7.70
7.69
7.69
7.39
7.39
7.37
7.37
7.19
7.17
7.13
7.11
6.75
6.73
5.21
5.20
5.19
5.18
4.35
4.31
3.72
3.50
3.49
3.47
3.45
2.36
Cl
OMe
S
O
O
O
Me
42 ^{1}H-NMR
(400 MHz, $CDCl_3$)
2.01
1.99
2.02
2.02
2.03
1.99
1.00
1.01
3.01
1.01
3.01
f1 (ppm)

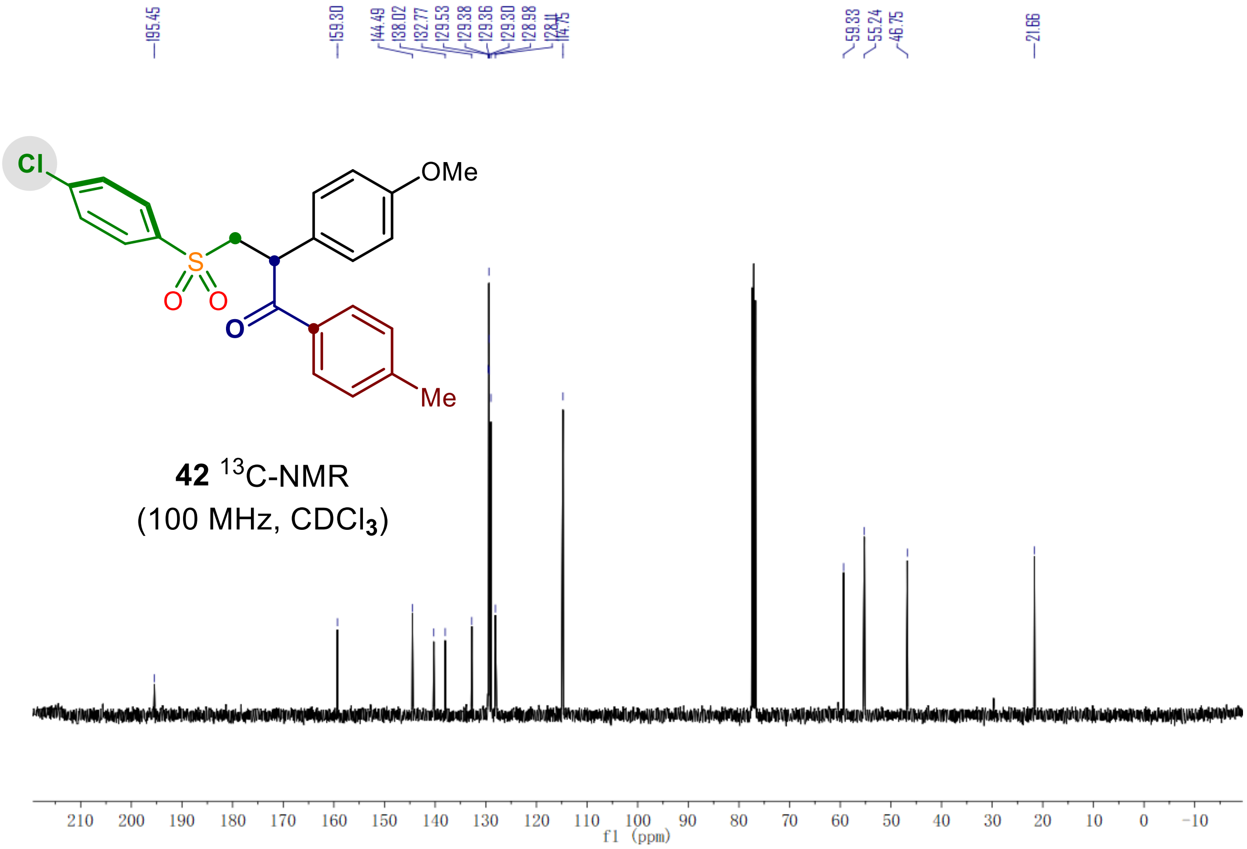

42 ^{13}C-NMR
(100 MHz, $CDCl_3$)

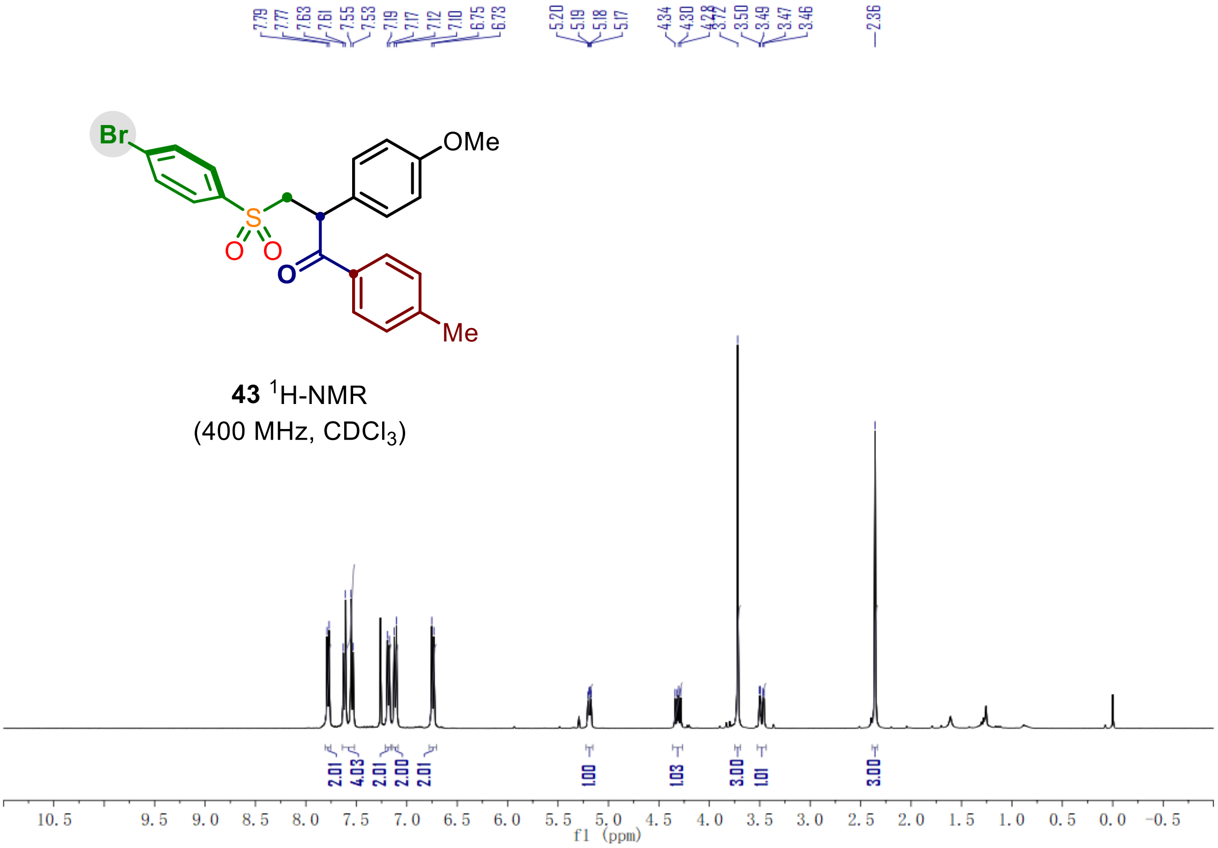

43 ^{1}H-NMR
(400 MHz, $CDCl_3$)

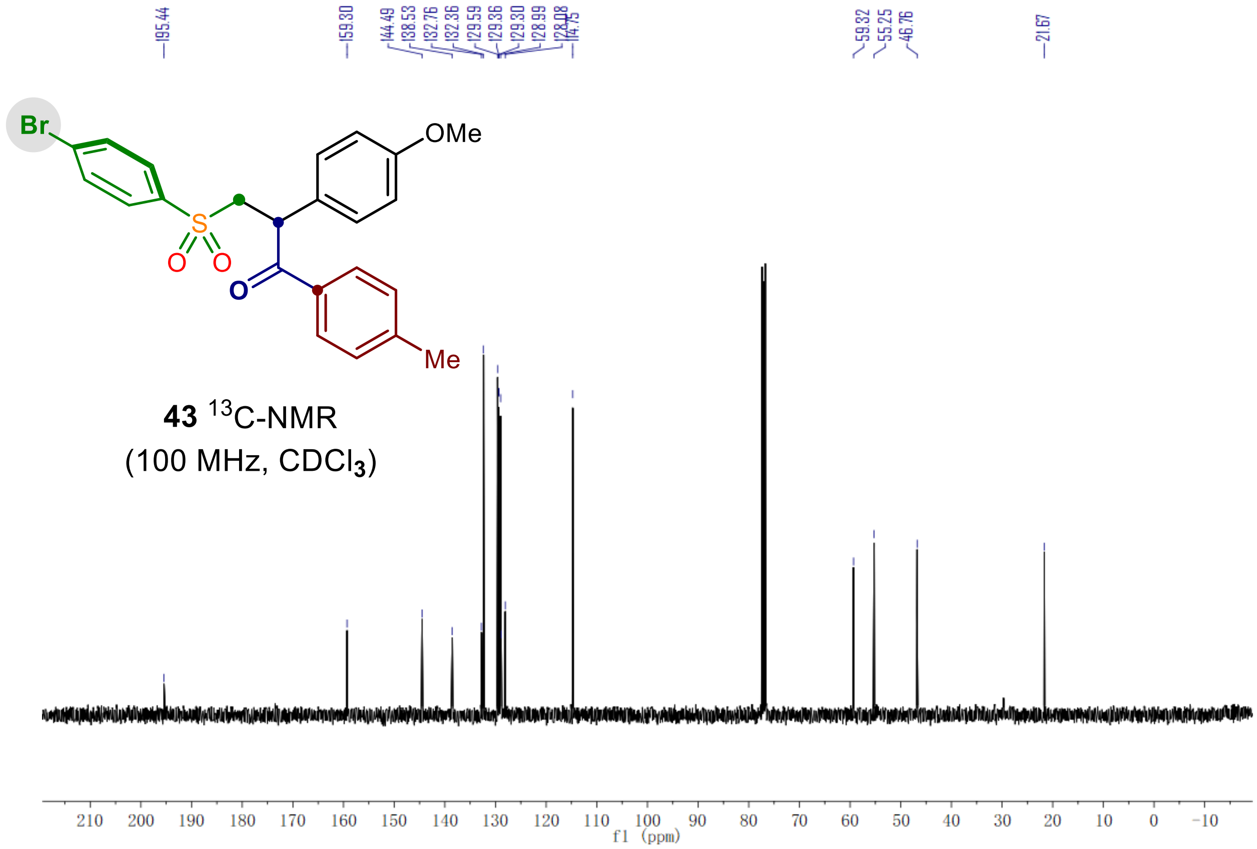
195.44
159.30
144.49
138.53
132.76
132.36
129.59
129.36
129.30
128.99
128.08
114.75
59.32
55.25
46.76
21.67
Br
OMe
S
O
O
O
Me
43 ^{13}C-NMR
(100 MHz, $CDCl_3$)
f1 (ppm)

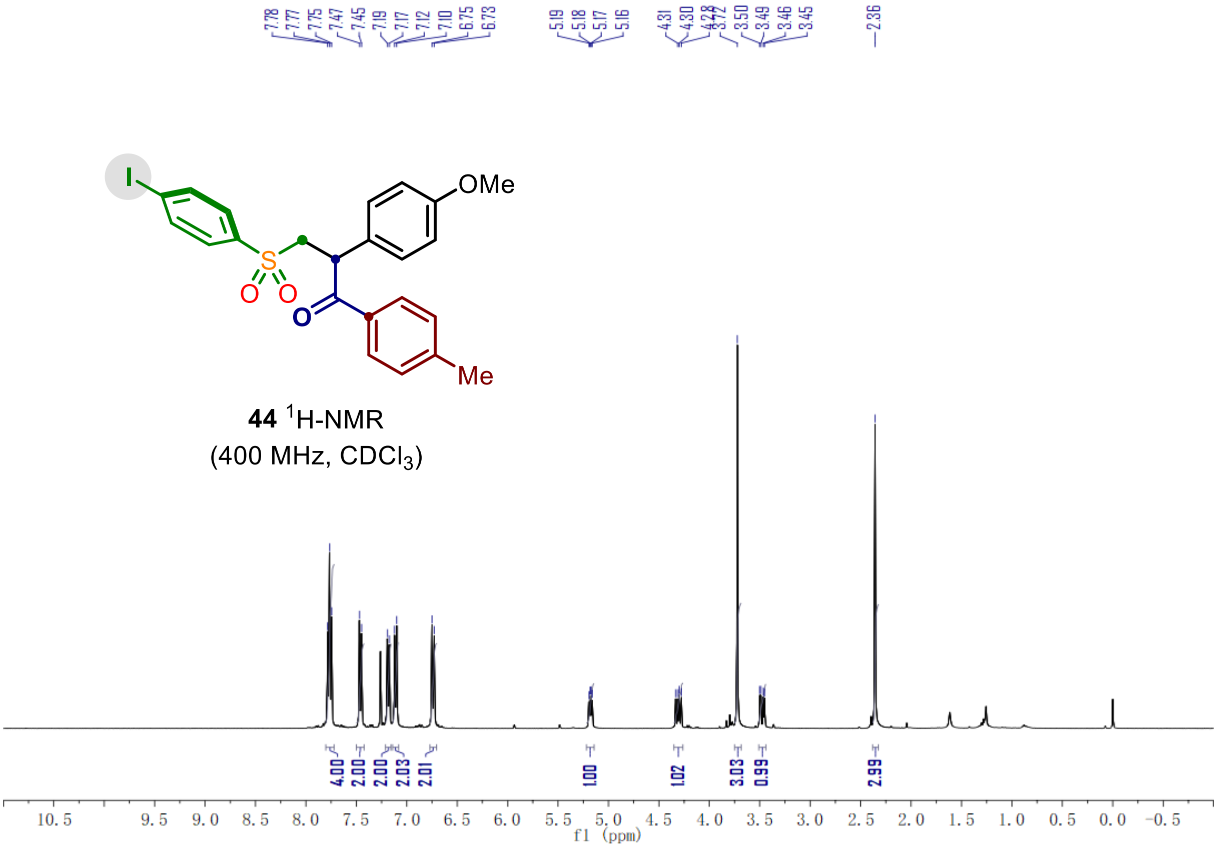
7.78
7.77
7.75
7.47
7.45
7.19
7.17
7.12
7.10
6.75
6.73
5.19
5.18
5.17
5.16
4.31
4.30
4.28
3.72
3.50
3.49
3.46
3.45
2.36
I
OMe
S
O
O
O
Me
44 ^{1}H-NMR
(400 MHz, $CDCl_3$)
4.00
2.00
2.00
2.03
2.01
1.00
1.02
3.03
0.99
2.99
f1 (ppm)

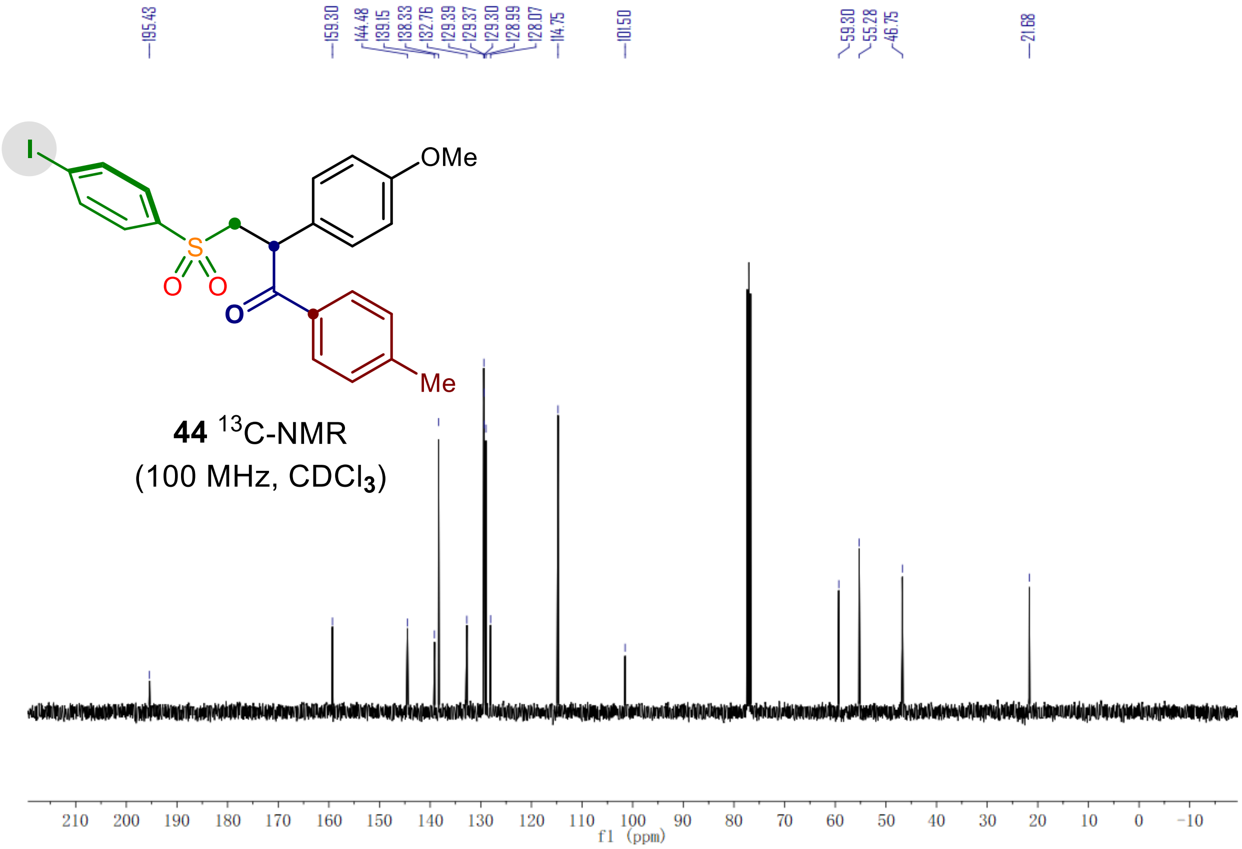
195.43
159.30
144.48
139.15
138.33
132.76
129.39
129.37
129.30
128.99
128.07
114.75
101.50
59.30
55.28
46.75
21.68
I
OMe
S
O O
O
Me
44 13C-NMR
(100 MHz, CDCl3)
210 200 190 180 170 160 150 140 130 120 110 100 90 80 70 60 50 40 30 20 10 0 -10
f1 (ppm)

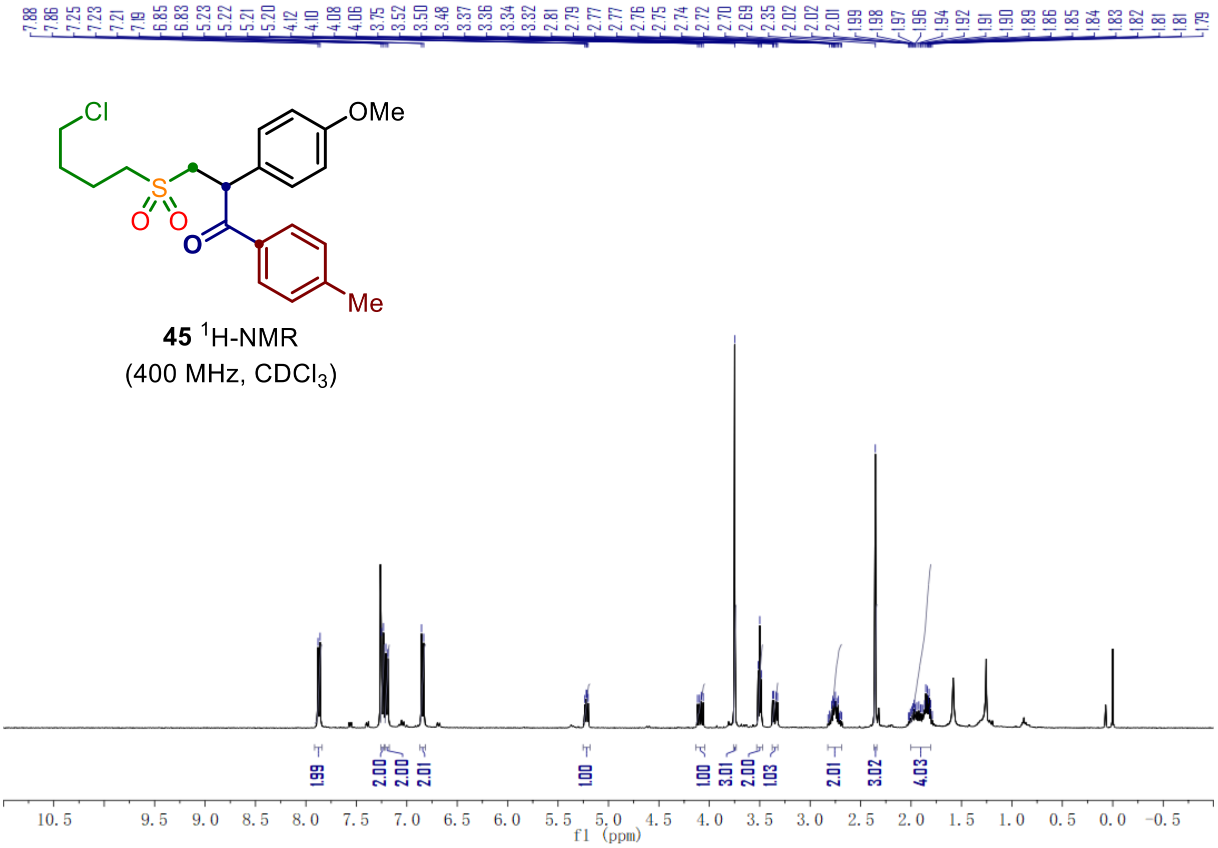
7.88 7.86 7.25 7.23 7.21 7.19 6.85 6.83 5.23 5.22 5.21 5.20 4.12 4.10 4.08 4.06 3.75 3.52 3.50 3.48 3.37 3.36 3.34 3.32 2.81 2.79 2.77 2.77 2.76 2.75 2.74 2.72 2.70 2.69 2.35 2.02 2.02 2.01 1.99 1.98 1.97 1.96 1.94 1.92 1.91 1.90 1.89 1.86 1.85 1.84 1.83 1.82 1.81 1.81 1.79
Cl
OMe
S
O O
O
Me
45 1H-NMR
(400 MHz, CDCl3)
1.99
2.00
2.00
2.01
1.00
1.00
3.01
2.00
1.03
2.01
3.02
4.03
10.5 9.5 9.0 8.5 8.0 7.5 7.0 6.5 6.0 5.5 5.0 4.5 4.0 3.5 3.0 2.5 2.0 1.5 1.0 0.5 0.0 -0.5
f1 (ppm)

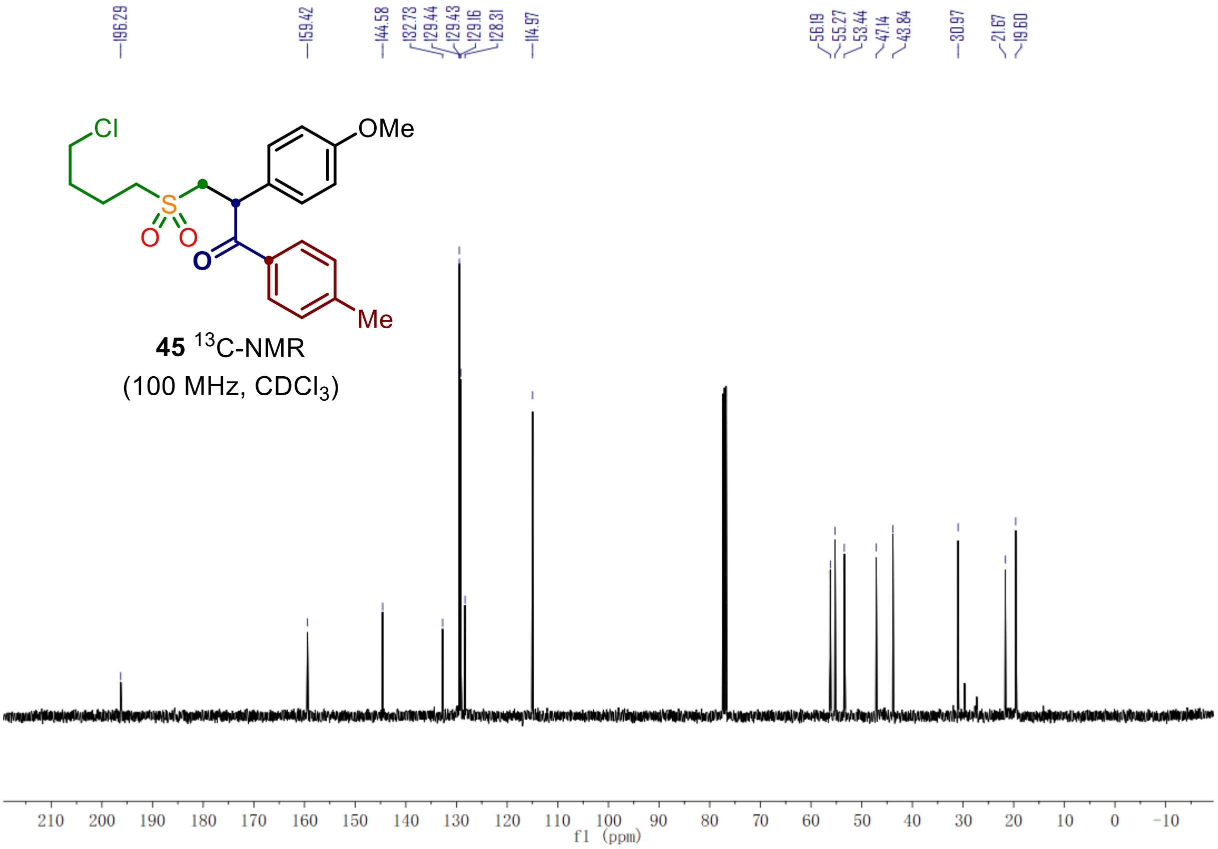

45 ^{13}C-NMR
(100 MHz, $CDCl_3$)

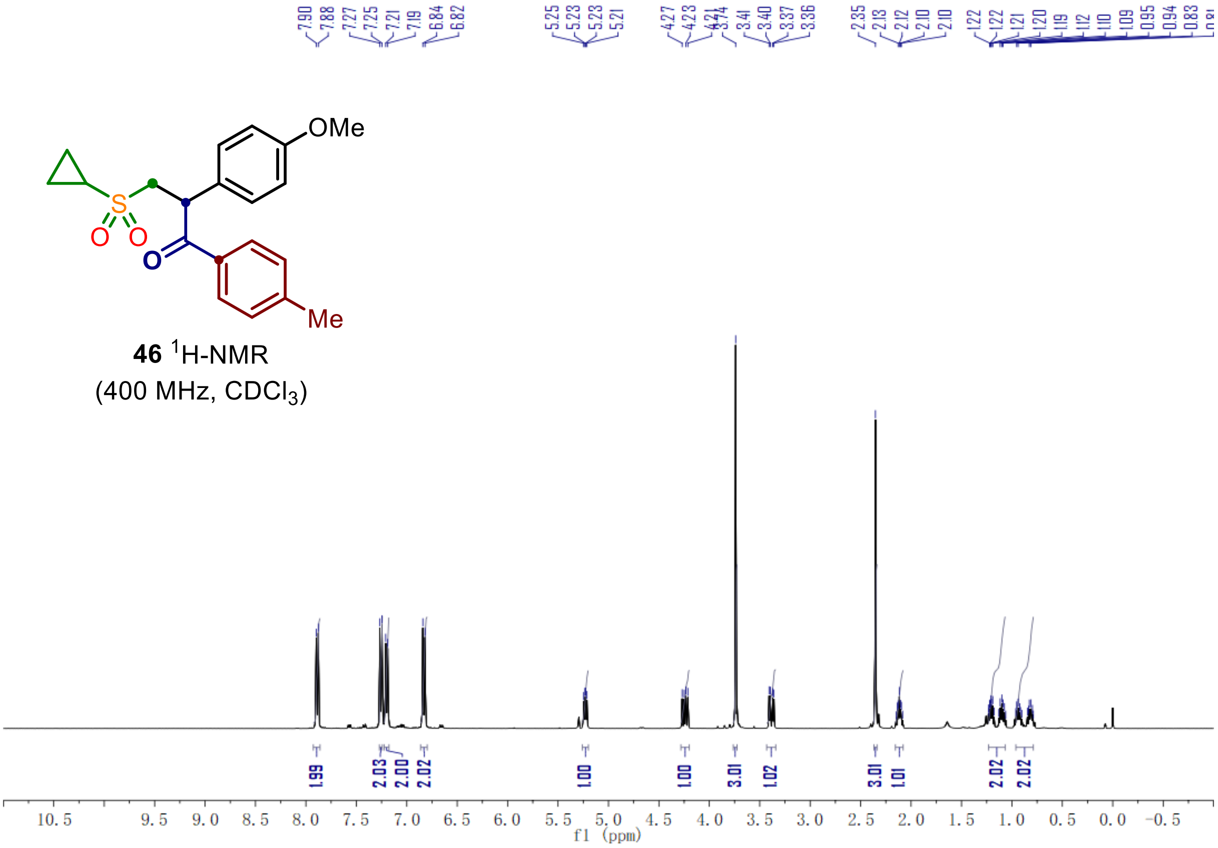

46 ^{1}H-NMR
(400 MHz, $CDCl_3$)

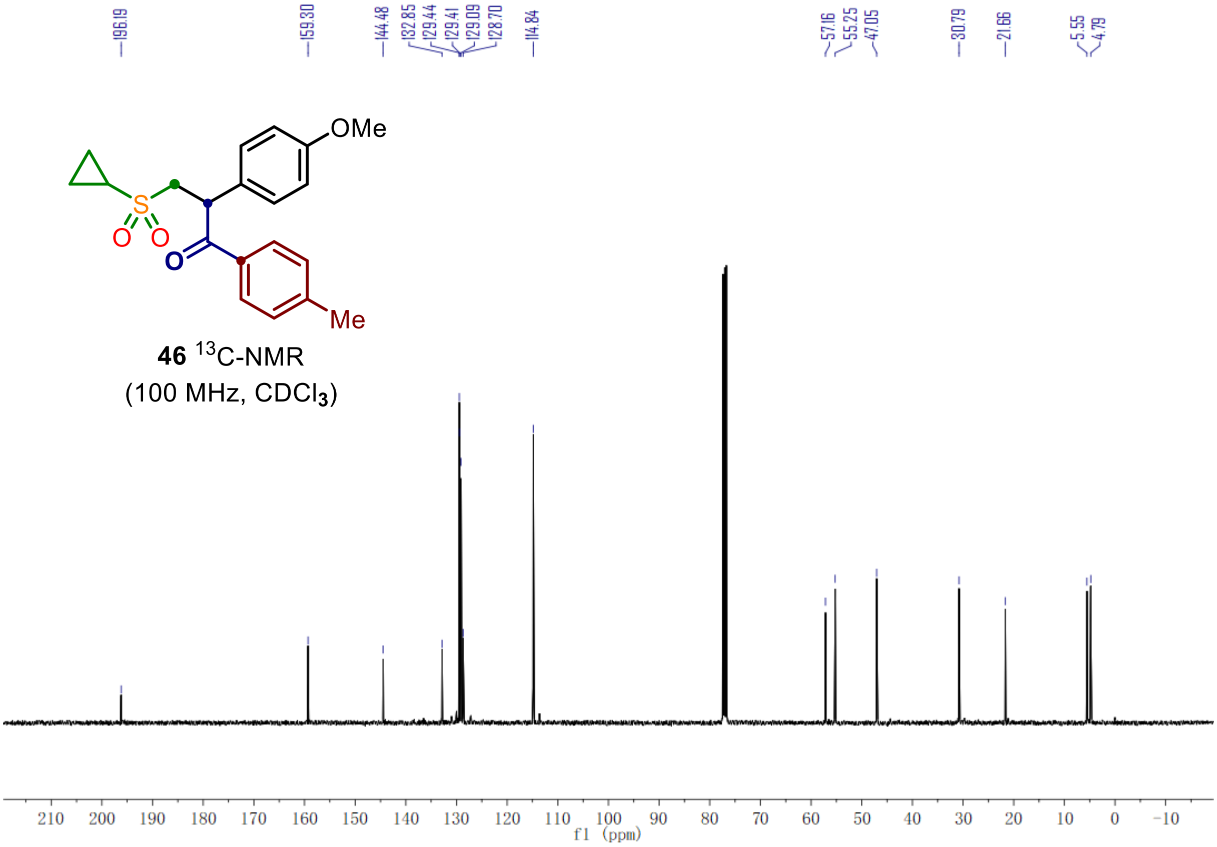

46 ^{13}C-NMR
(100 MHz, $CDCl_3$)

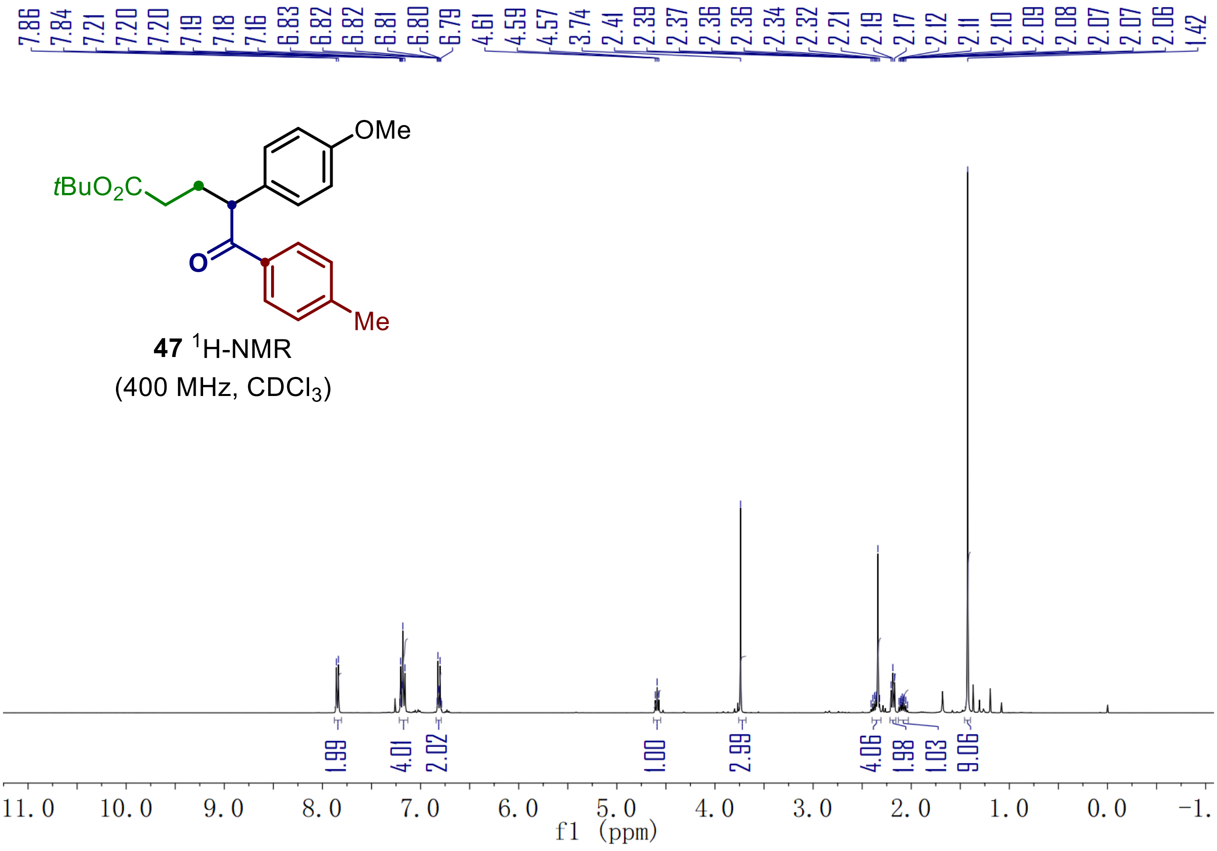

47 ^{1}H-NMR
(400 MHz, $CDCl_3$)

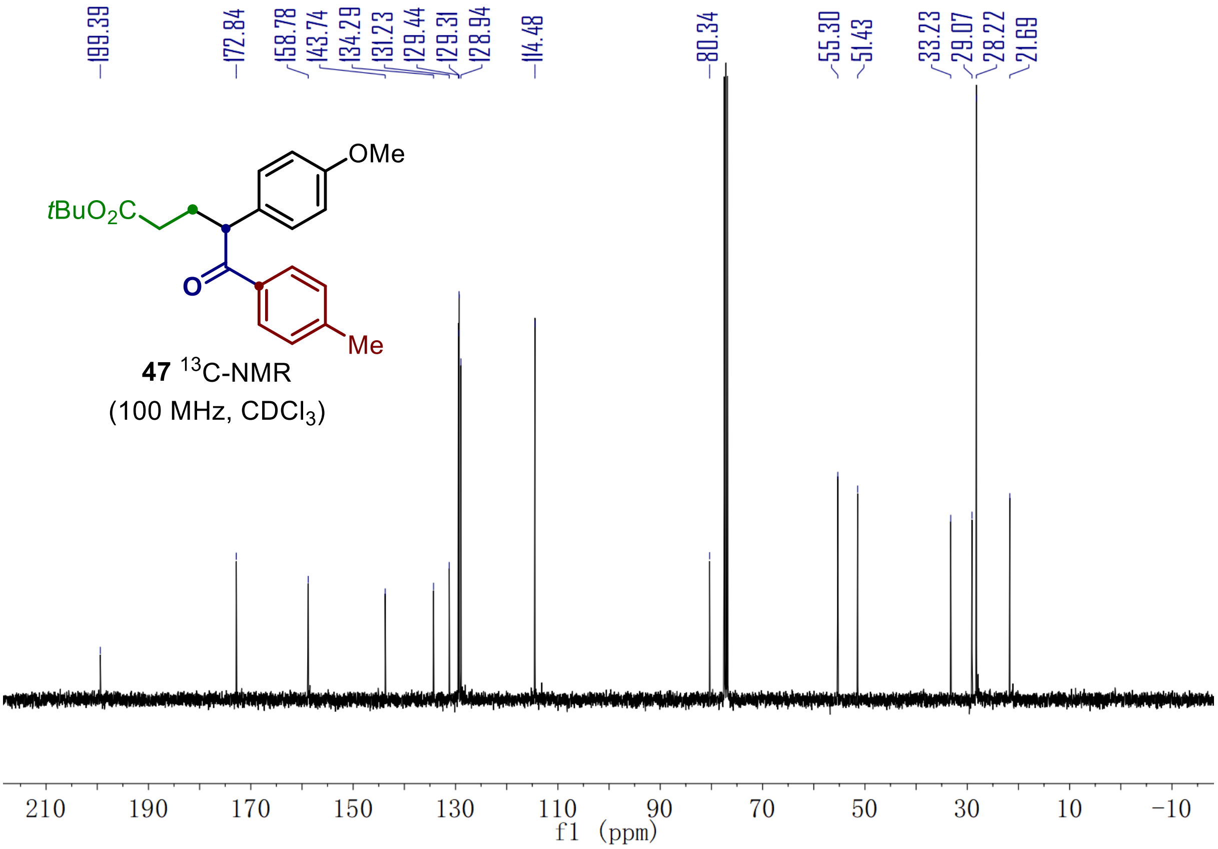
199.39
172.84
158.78
143.74
134.29
131.23
129.44
129.31
128.94
114.48
80.34
55.30
51.43
33.23
29.07
28.22
21.69
OMe
tBuO2C
O
Me
47 13C-NMR
(100 MHz, CDCl3)
210 190 170 150 130 110 90 70 50 30 10 -10
f1 (ppm)

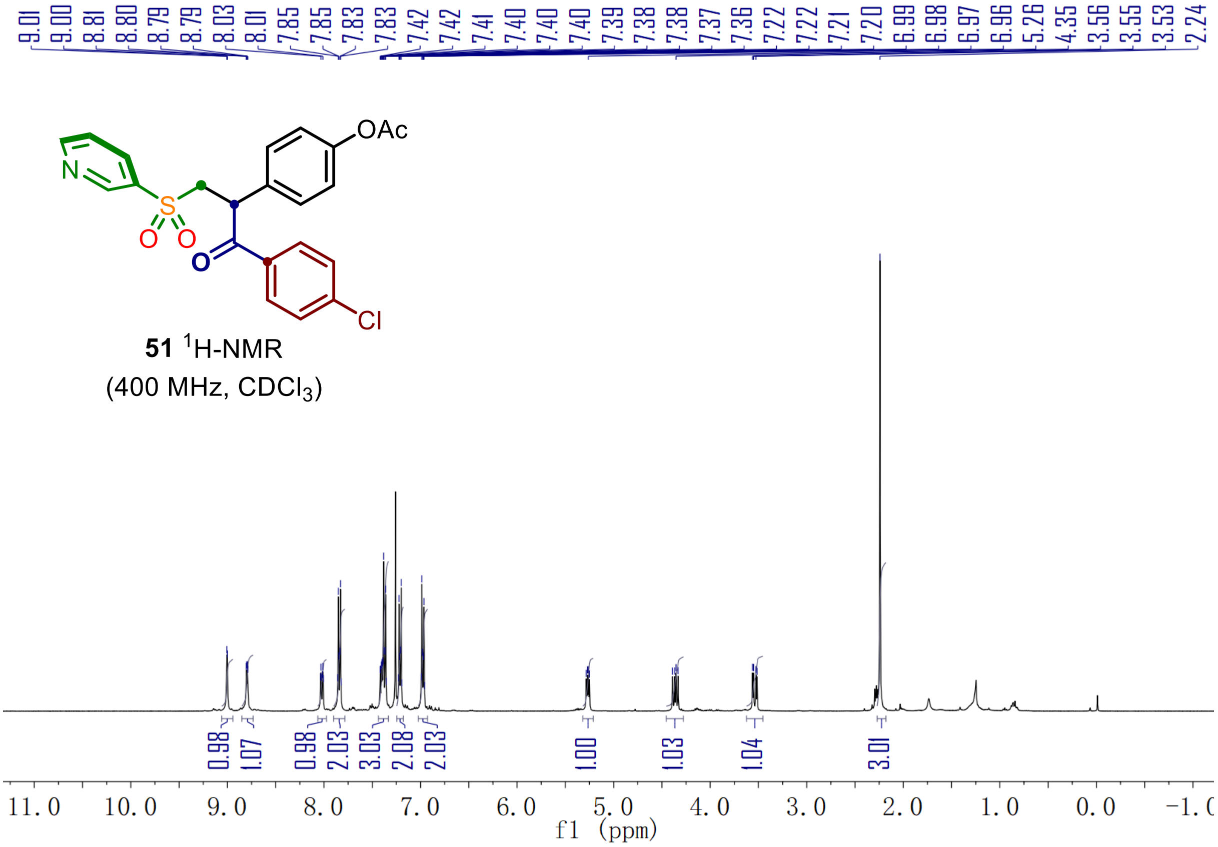
9.01 9.00 8.81 8.80 8.79 8.79 8.03 8.01 7.85 7.85 7.83 7.83 7.42 7.42 7.41 7.40 7.40 7.40 7.39 7.38 7.38 7.37 7.36 7.22 7.22 7.21 7.20 6.99 6.98 6.97 6.96 5.26 4.35 3.56 3.55 3.53 2.24
OAc
N
S
O O
O
Cl
51 1H-NMR
(400 MHz, CDCl3)
0.98 1.07 0.98 2.03 3.03 2.08 2.03 1.00 1.03 1.04 3.01
11.0 10.0 9.0 8.0 7.0 6.0 5.0 4.0 3.0 2.0 1.0 0.0 -1.0
f1 (ppm)

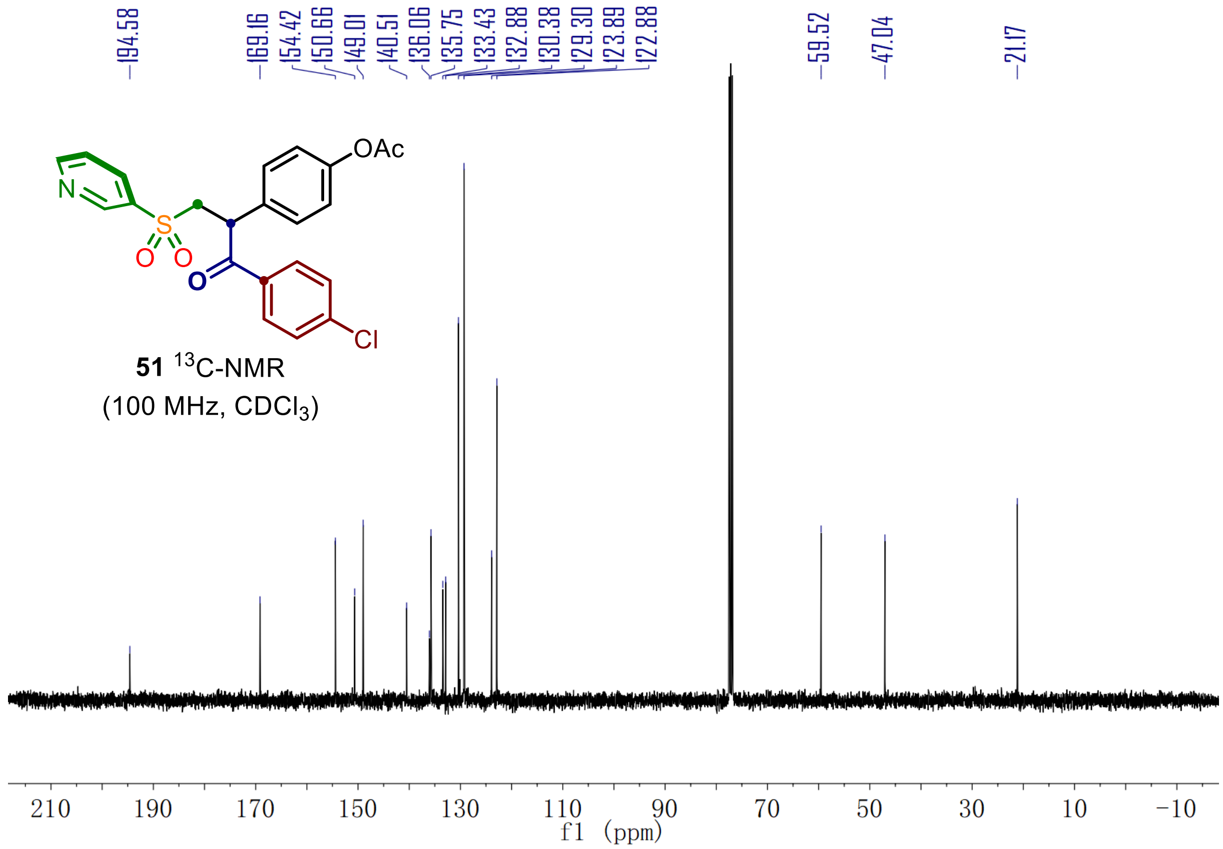
194.58
169.16
154.42
150.66
149.01
140.51
136.06
135.75
133.43
132.88
130.38
129.30
123.89
122.88
59.52
47.04
21.17
OAc
N
S
O
O
O
Cl
51 13C-NMR
(100 MHz, CDCl3)
210 190 170 150 130 110 90 70 50 30 10 −10
f1 (ppm)

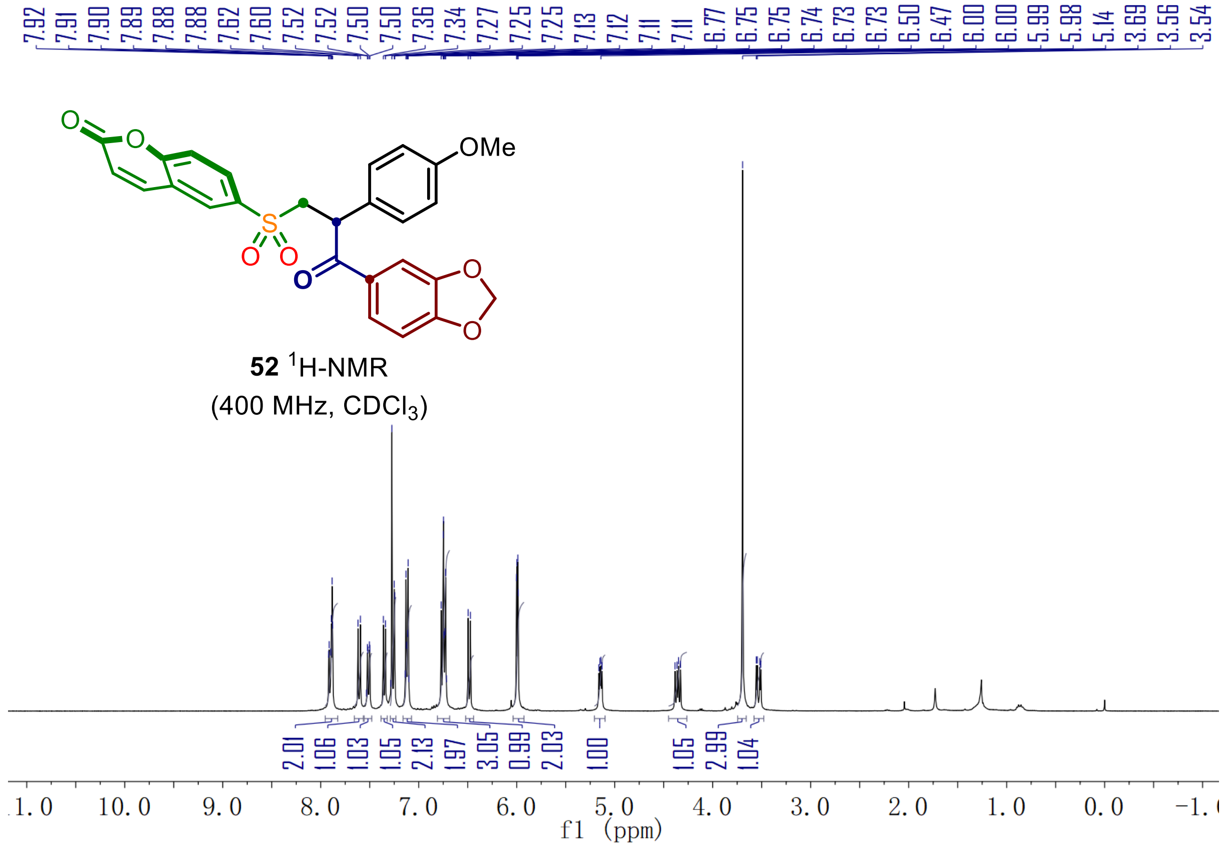
7.92
7.91
7.90
7.89
7.88
7.88
7.62
7.60
7.52
7.52
7.50
7.50
7.36
7.34
7.27
7.25
7.25
7.13
7.12
7.11
7.11
6.77
6.75
6.75
6.74
6.73
6.73
6.50
6.47
6.00
6.00
5.99
5.98
5.14
3.69
3.56
3.54
O
O
OMe
S
O
O
O
O
O
52 1H-NMR
(400 MHz, CDCl3)
2.01
1.06
1.03
1.05
2.13
1.97
3.05
0.99
2.03
1.00
1.05
2.99
1.04
10.0 9.0 8.0 7.0 6.0 5.0 4.0 3.0 2.0 1.0 0.0
f1 (ppm)

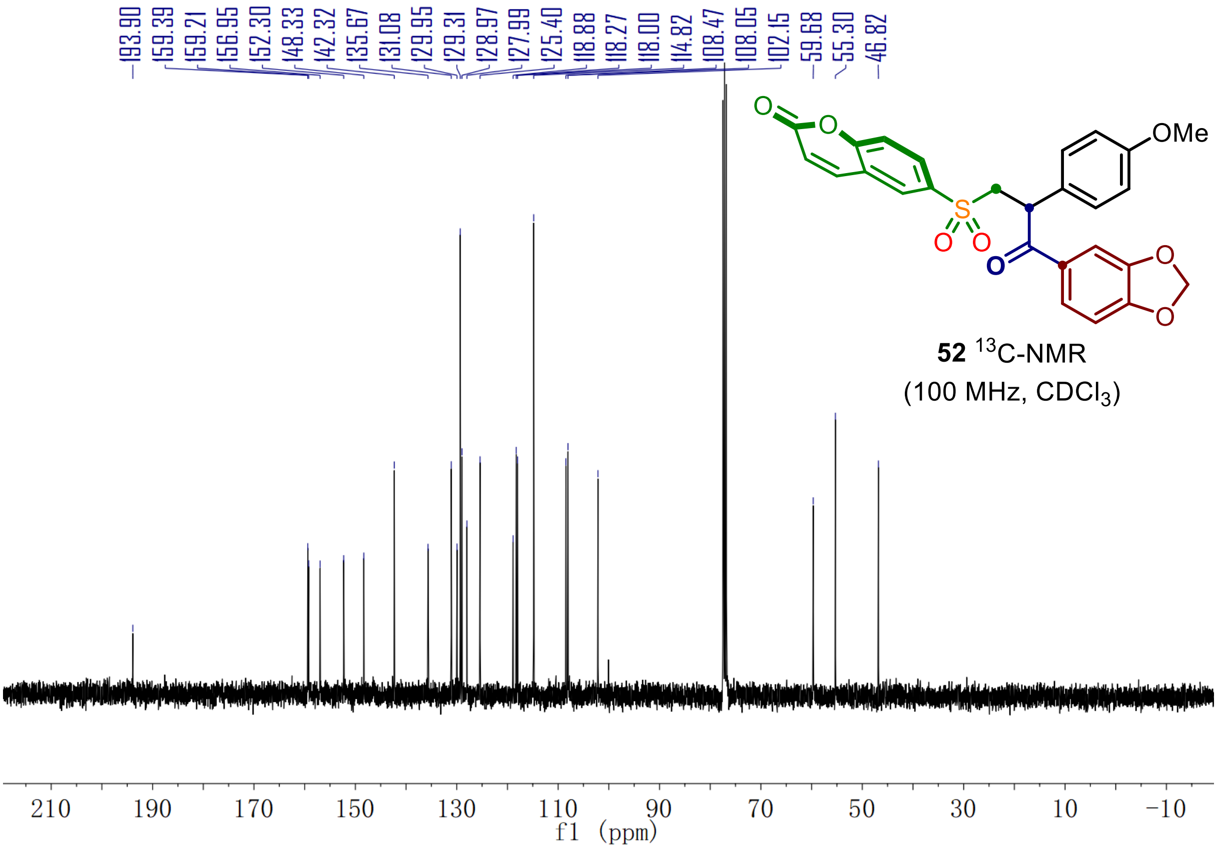
193.90 159.39 159.21 156.95 152.30 148.33 142.32 135.67 131.08 129.95 129.31 128.97 127.99 125.40 118.88 118.27 118.00 114.82 108.47 108.05 102.15 59.68 55.30 46.82
OMe
52 13C-NMR
(100 MHz, CDCl3)
f1 (ppm)

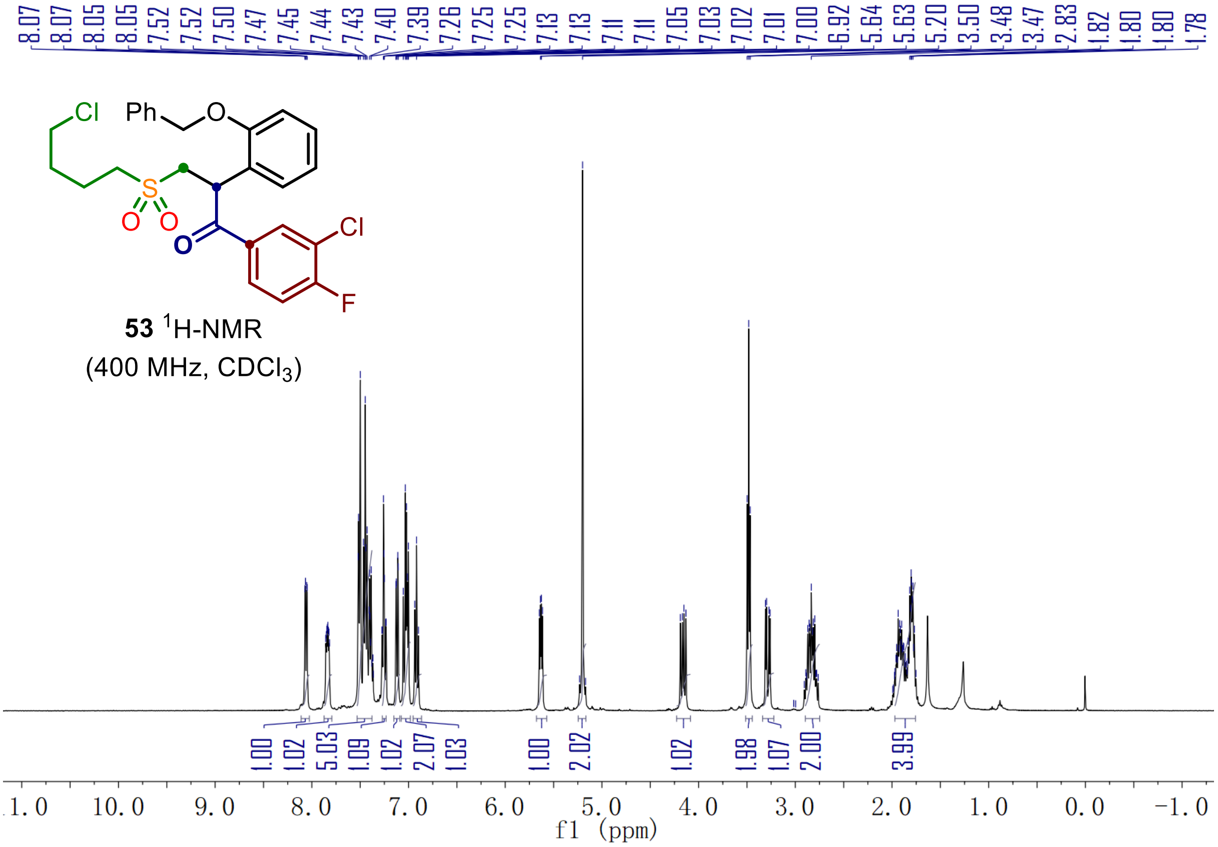
8.07 8.07 8.05 8.05 7.52 7.52 7.50 7.47 7.45 7.44 7.43 7.40 7.39 7.26 7.25 7.25 7.13 7.13 7.11 7.11 7.05 7.03 7.02 7.01 7.00 6.92 5.64 5.63 5.20 3.50 3.48 3.47 2.83 1.82 1.80 1.80 1.78
Cl
Ph
Cl
F
53 1H-NMR
(400 MHz, CDCl3)
1.00 1.02 5.03 1.09 1.02 2.07 1.03 1.00 2.02 1.02 1.98 1.07 2.00 3.99
f1 (ppm)

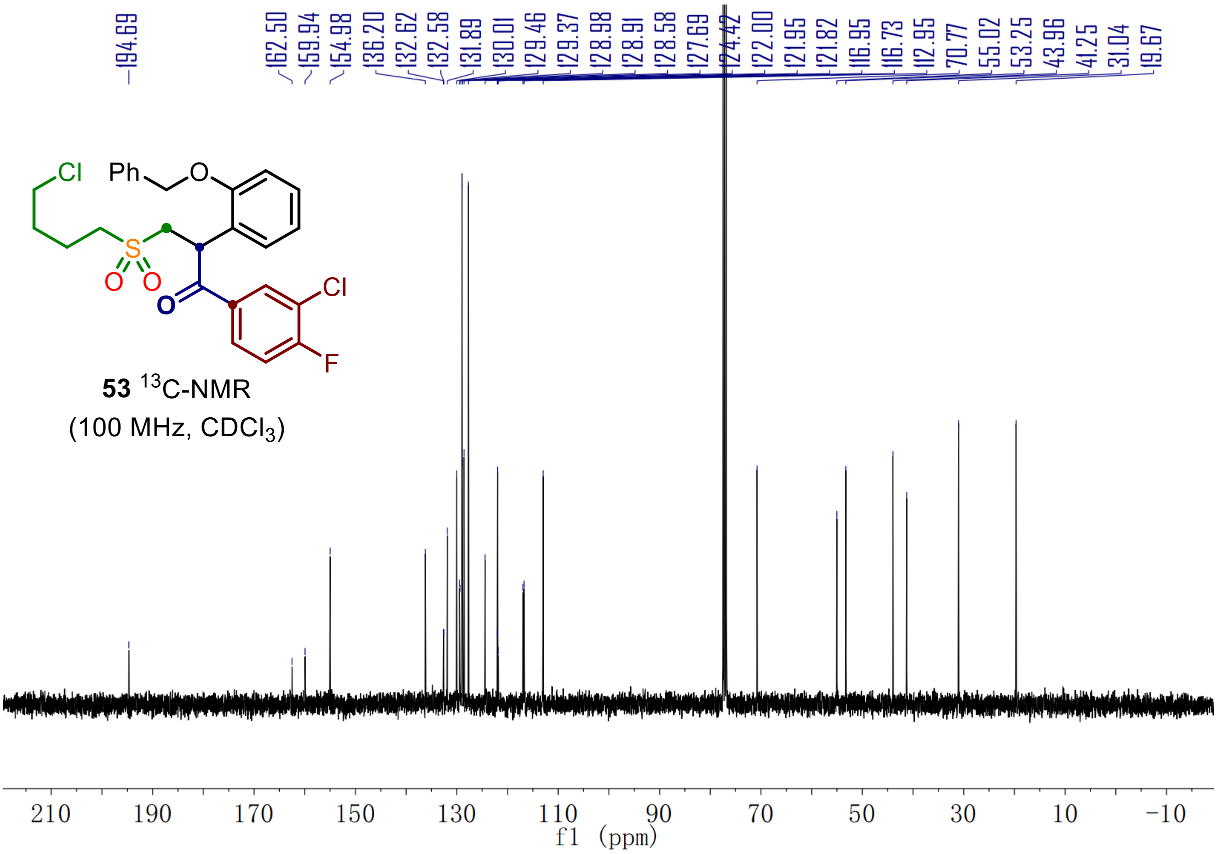
194.69
162.50
159.94
154.98
136.20
132.62
132.58
131.89
130.01
129.46
129.37
128.98
128.91
128.58
127.69
124.42
122.00
121.95
121.82
116.95
116.73
112.95
70.77
55.02
53.25
43.96
41.25
31.04
19.67
Cl
Ph
O
S
O
O
O
Cl
F
53 ^{13}C-NMR
(100 MHz, $CDCl_3$)
210 190 170 150 130 110 90 70 50 30 10 −10
f1 (ppm)

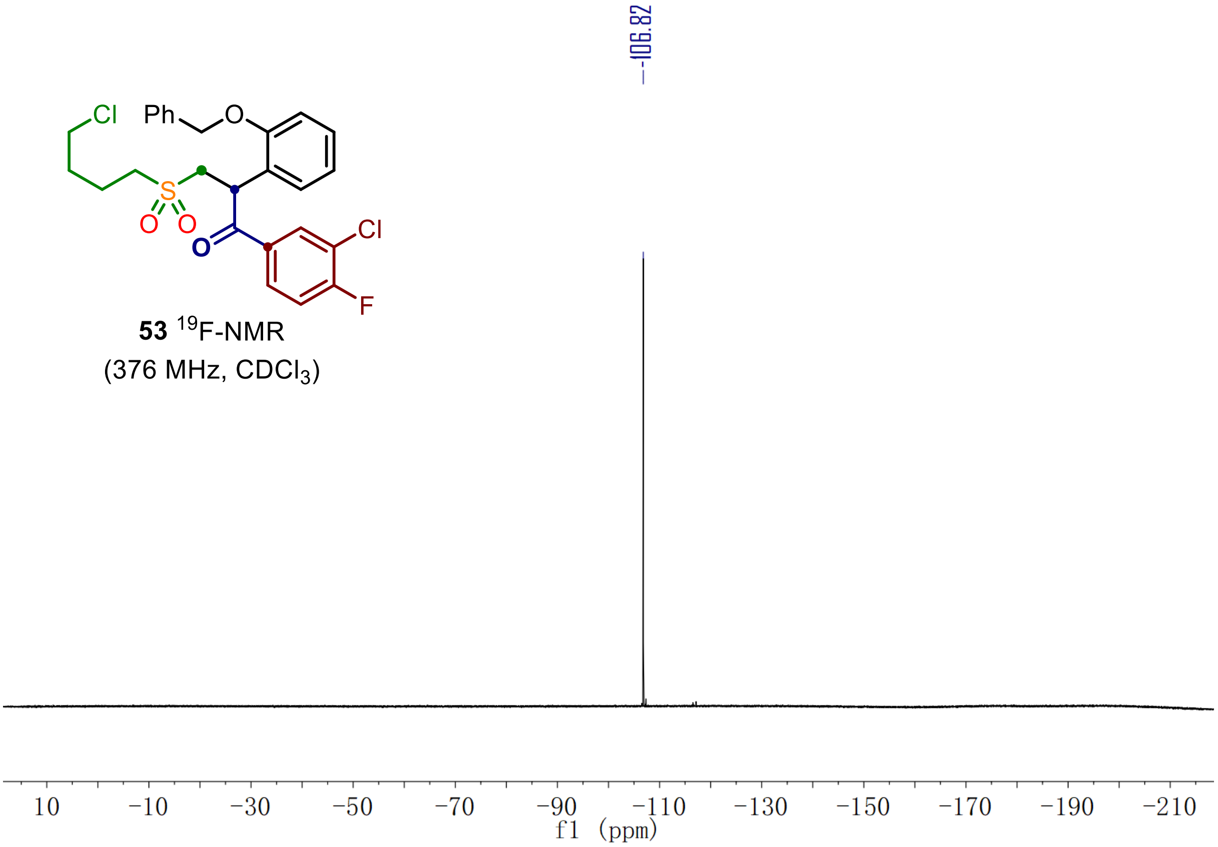
−106.82
Cl
Ph
O
S
O
O
O
Cl
F
53 ^{19}F-NMR
(376 MHz, $CDCl_3$)
10 −10 −30 −50 −70 −90 −110 −130 −150 −170 −190 −210
f1 (ppm)

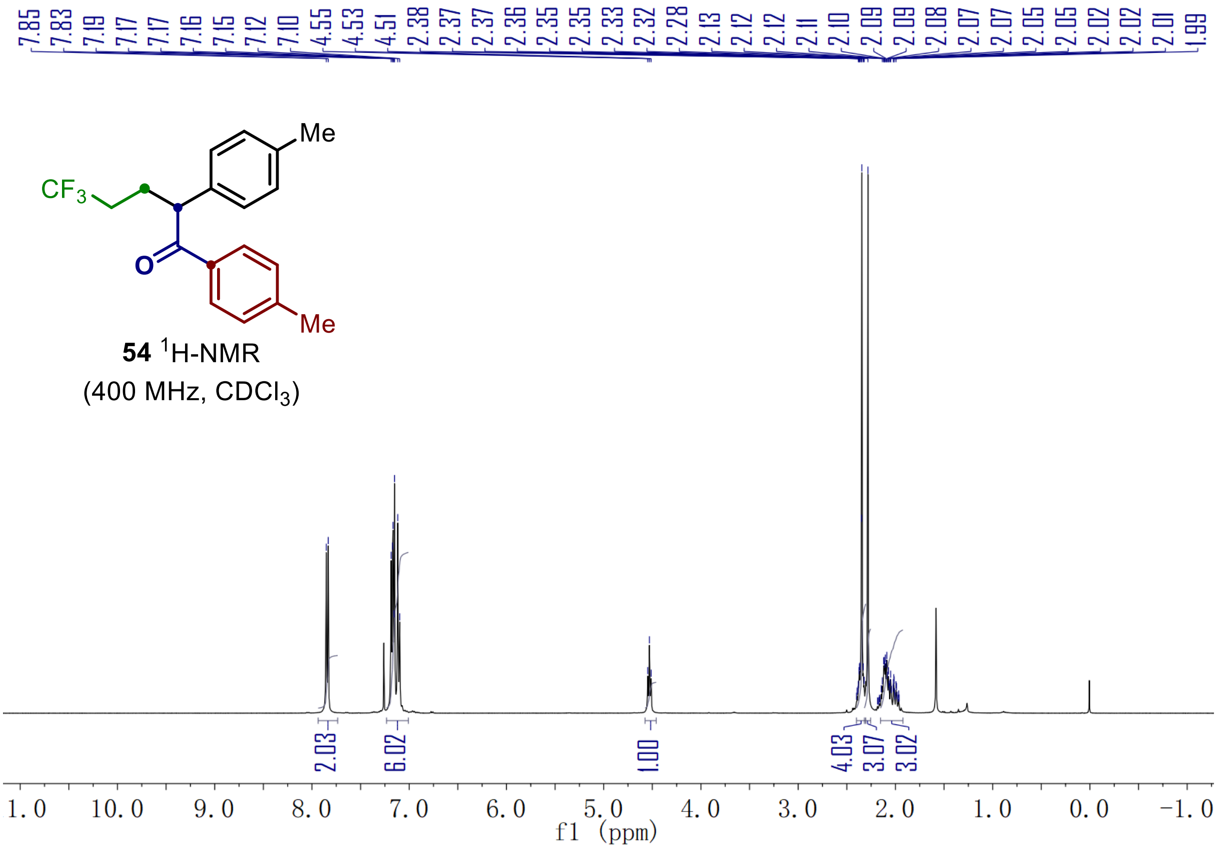

54 ^{1}H-NMR
(400 MHz, $CDCl_3$)

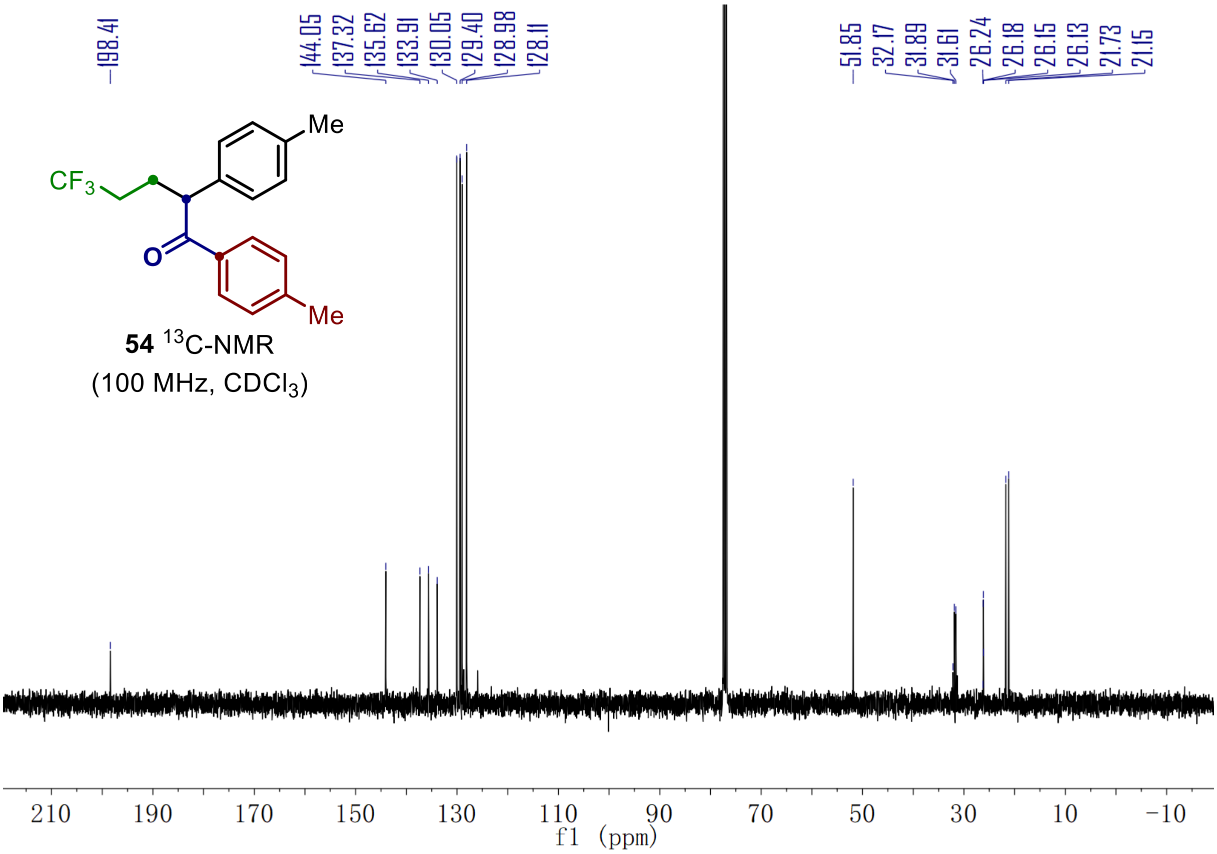

54 ^{13}C-NMR
(100 MHz, $CDCl_3$)

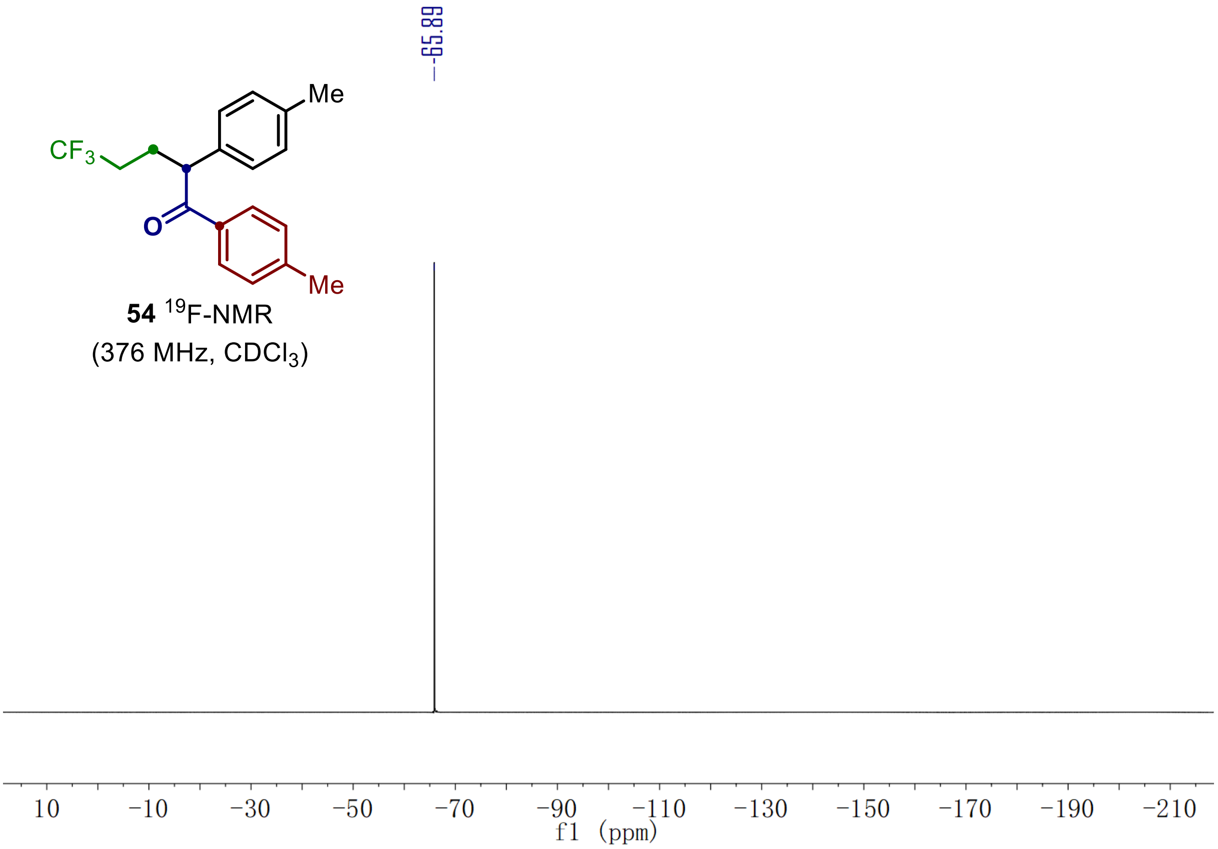
54 ^{19}F-NMR
(376 MHz, $CDCl_3$)
-65.89
f1 (ppm)

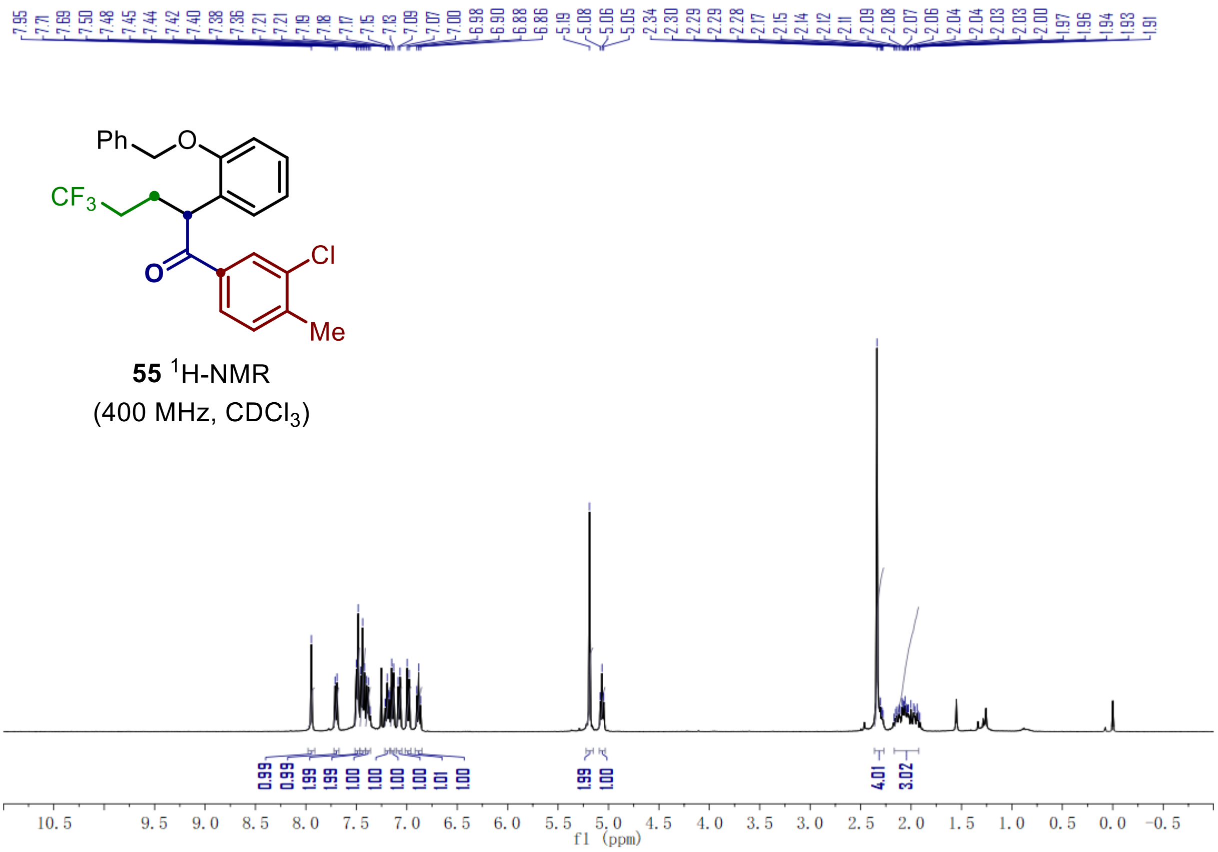
55 ^{1}H-NMR
(400 MHz, $CDCl_3$)
f1 (ppm)

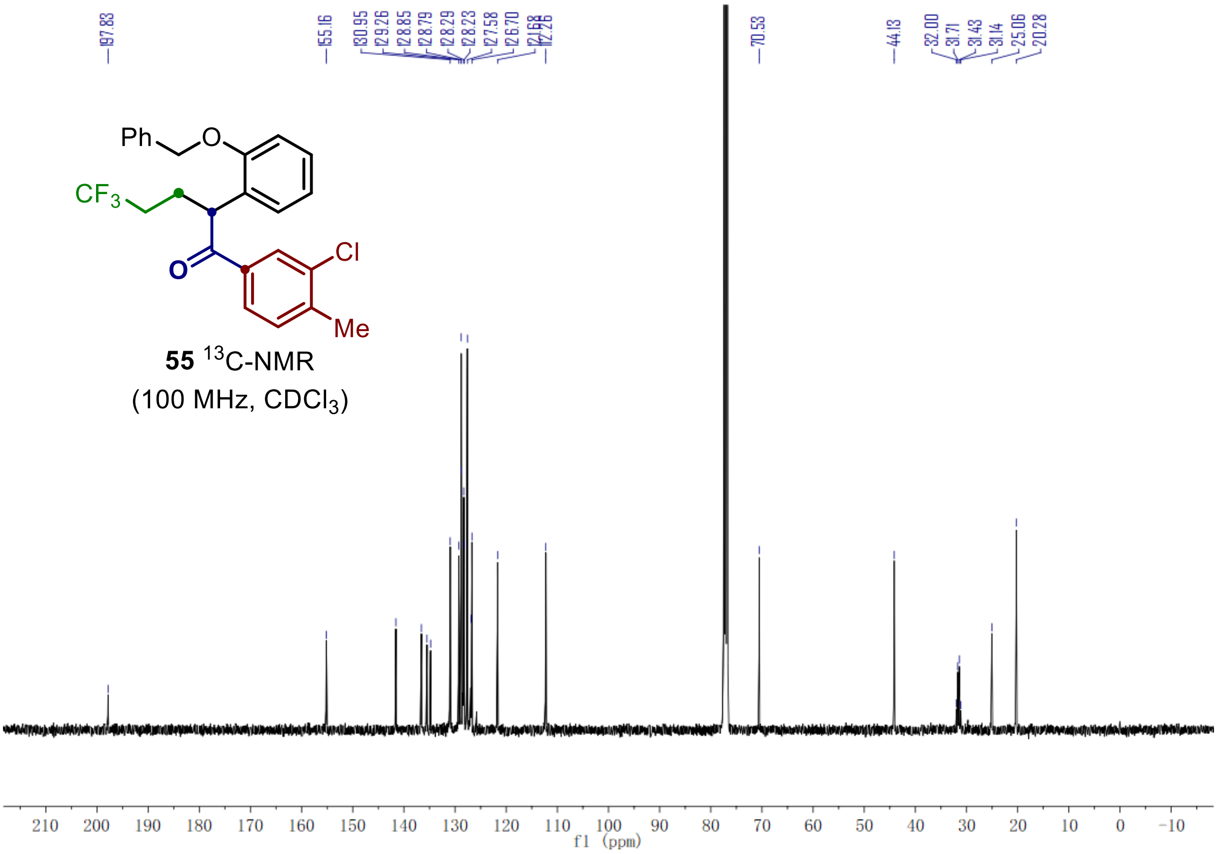
197.83
155.16
130.95
129.26
128.85
128.79
128.29
128.23
127.58
126.70
121.68
112.26
70.53
44.13
32.00
31.71
31.43
31.14
25.06
20.28
Ph
O
CF3
O
Cl
Me
55 13C-NMR
(100 MHz, CDCl3)
210 200 190 180 170 160 150 140 130 120 110 100 90 80 70 60 50 40 30 20 10 0 -10
f1 (ppm)

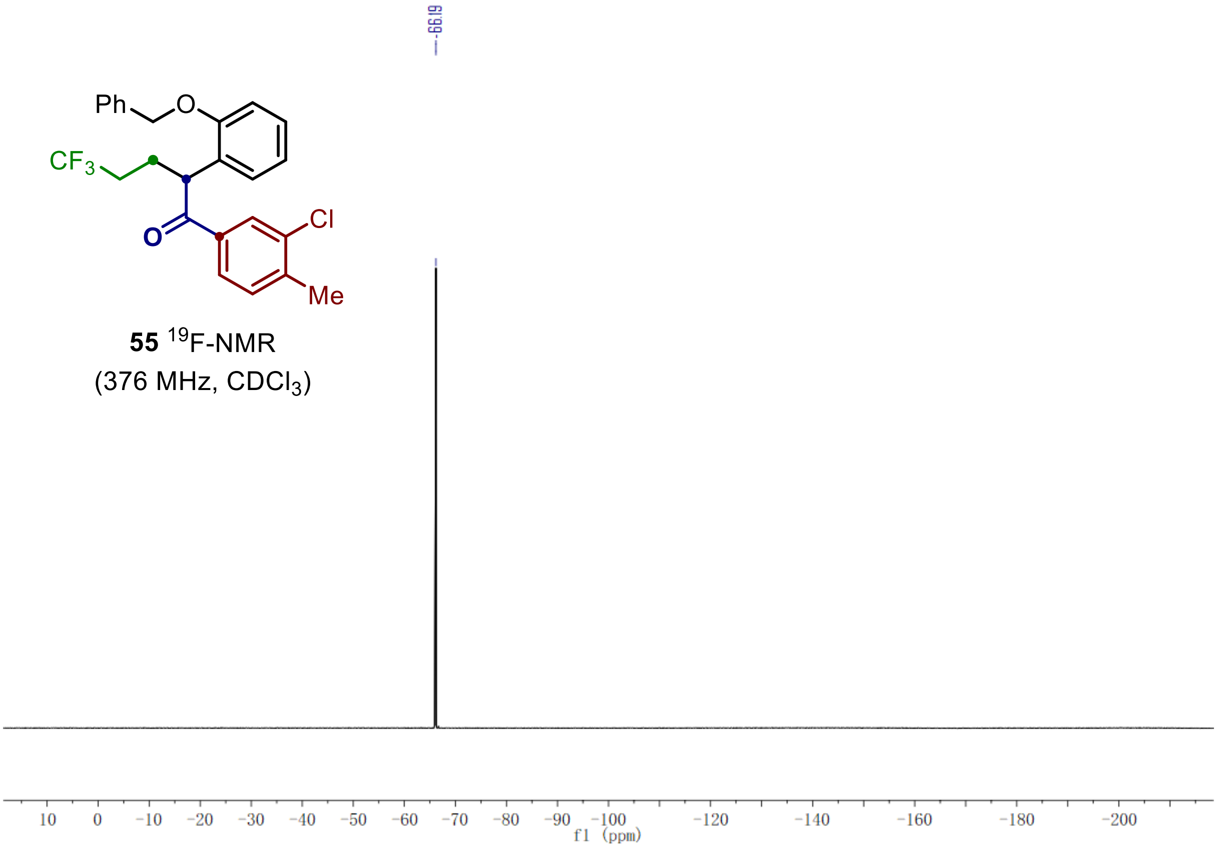
-66.19
Ph
O
CF3
O
Cl
Me
55 19F-NMR
(376 MHz, CDCl3)
10 0 -10 -20 -30 -40 -50 -60 -70 -80 -90 -100 -120 -140 -160 -180 -200
f1 (ppm)

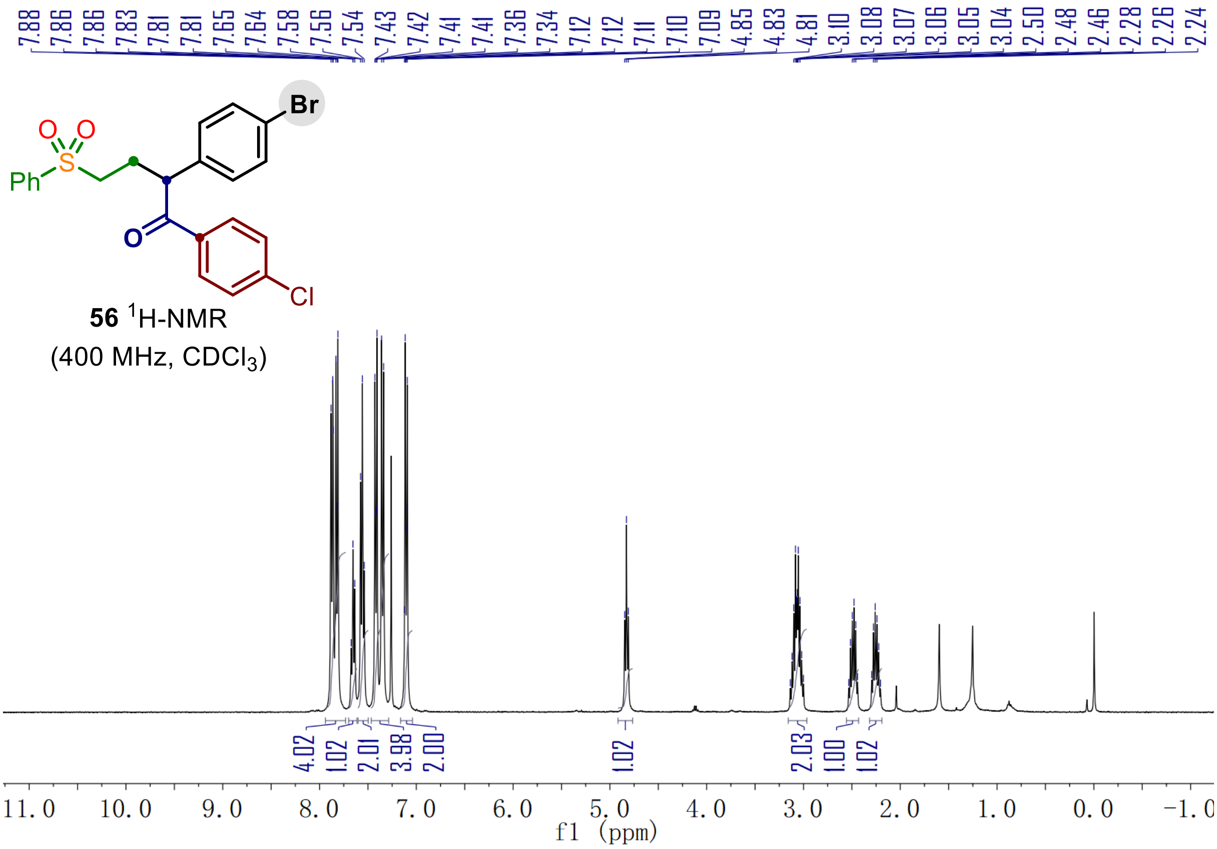
7.88
7.86
7.86
7.83
7.81
7.81
7.65
7.64
7.58
7.56
7.54
7.43
7.42
7.41
7.41
7.36
7.34
7.12
7.12
7.11
7.10
7.09
4.85
4.83
4.81
3.10
3.08
3.07
3.06
3.05
3.04
2.50
2.48
2.46
2.28
2.26
2.24
Br
O
O
Ph
S
O
Cl
56 1H-NMR
(400 MHz, CDCl3)
4.02
1.02
2.01
3.98
2.00
1.02
2.03
1.00
1.02
11.0
10.0
9.0
8.0
7.0
6.0
5.0
4.0
3.0
2.0
1.0
0.0
-1.0
f1 (ppm)

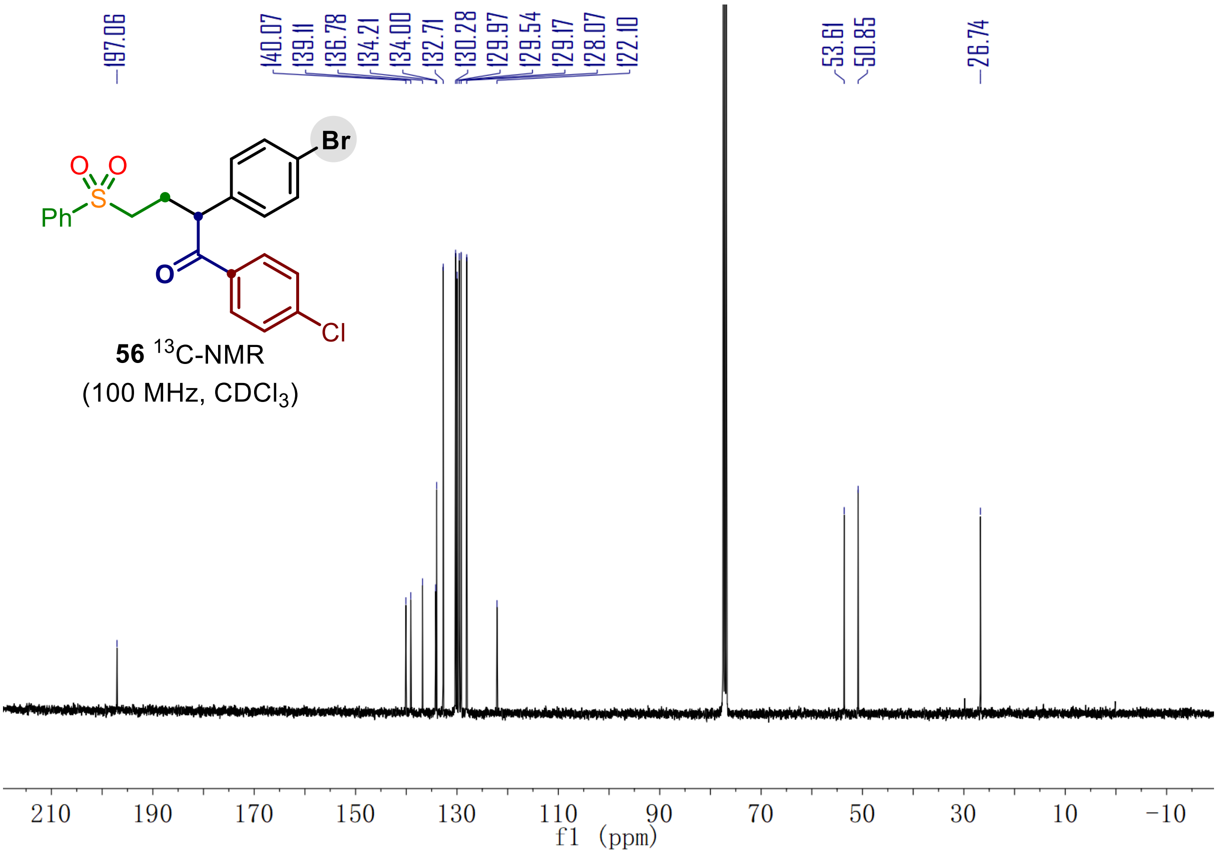
197.06
140.07
139.11
136.78
134.21
134.00
132.71
130.28
129.97
129.54
129.17
128.07
122.10
53.61
50.85
26.74
Br
O
O
Ph
S
O
Cl
56 13C-NMR
(100 MHz, CDCl3)
210
190
170
150
130
110
90
70
50
30
10
-10
f1 (ppm)

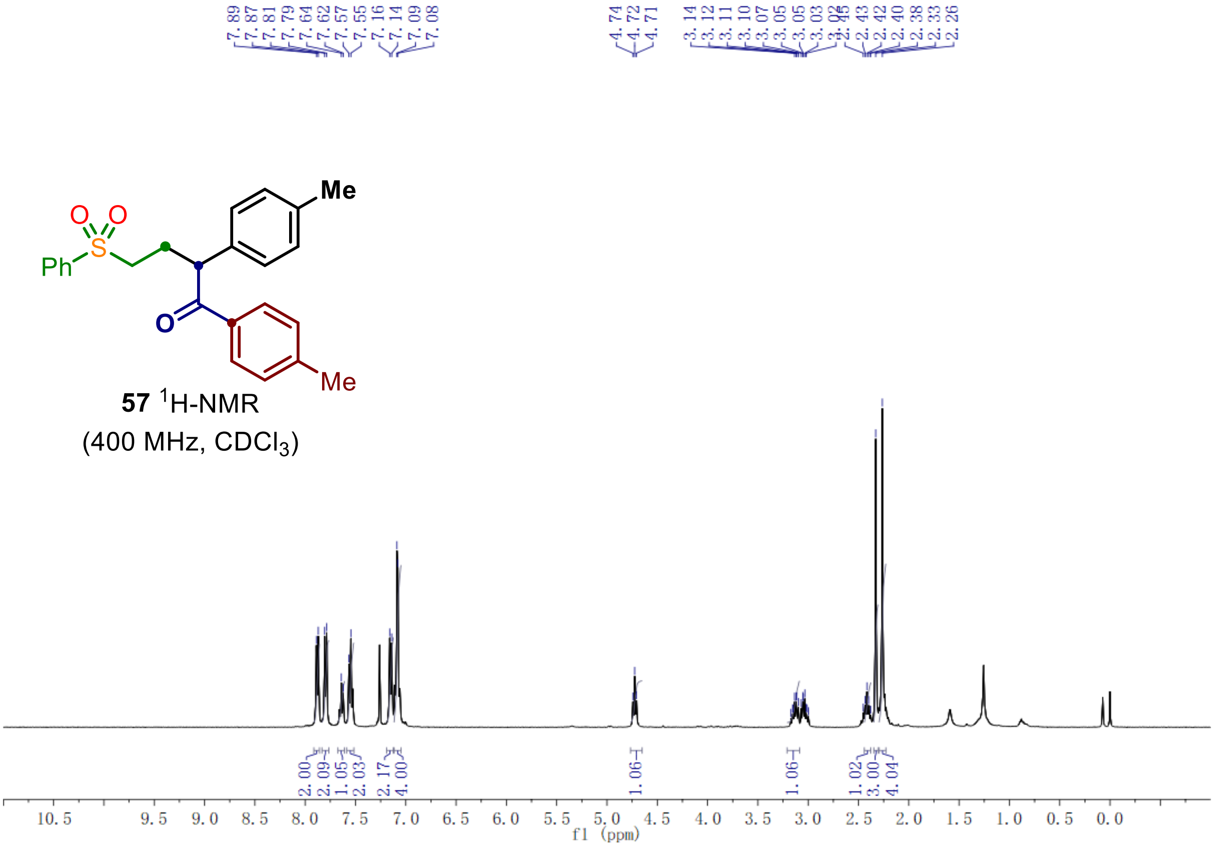
7.89 7.87 7.81 7.79 7.64 7.62 7.57 7.55 7.16 7.14 7.09 7.08
4.74 4.72 4.71
3.14 3.12 3.11 3.10 3.07 3.05 3.05 3.03 [illegible] 2.43 2.42 2.40 2.38 2.33 2.26
Me
O O
Ph S
O
Me
57 1H-NMR
(400 MHz, CDCl3)
2.00 2.09 1.05 2.03 2.17 4.00
1.06
1.06
1.02 3.00 4.04
10.5 9.5 9.0 8.5 8.0 7.5 7.0 6.5 6.0 5.5 5.0 4.5 4.0 3.5 3.0 2.5 2.0 1.5 1.0 0.5 0.0
f1 (ppm)

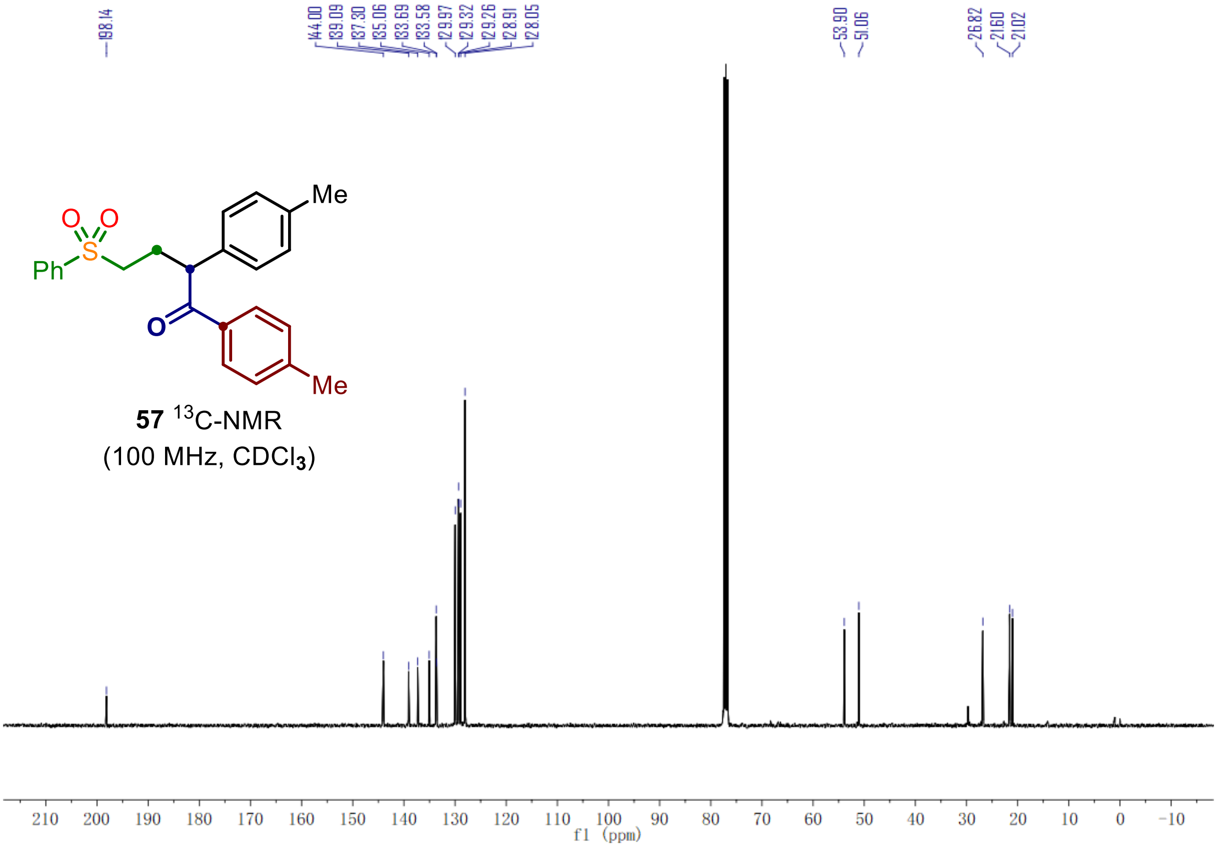
198.14
144.00 139.09 137.30 135.06 133.69 133.58 129.97 129.32 129.26 128.91 128.05
53.90 51.06
26.82 21.60 21.02
Me
O O
Ph S
O
Me
57 13C-NMR
(100 MHz, CDCl3)
210 200 190 180 170 160 150 140 130 120 110 100 90 80 70 60 50 40 30 20 10 0 -10
f1 (ppm)

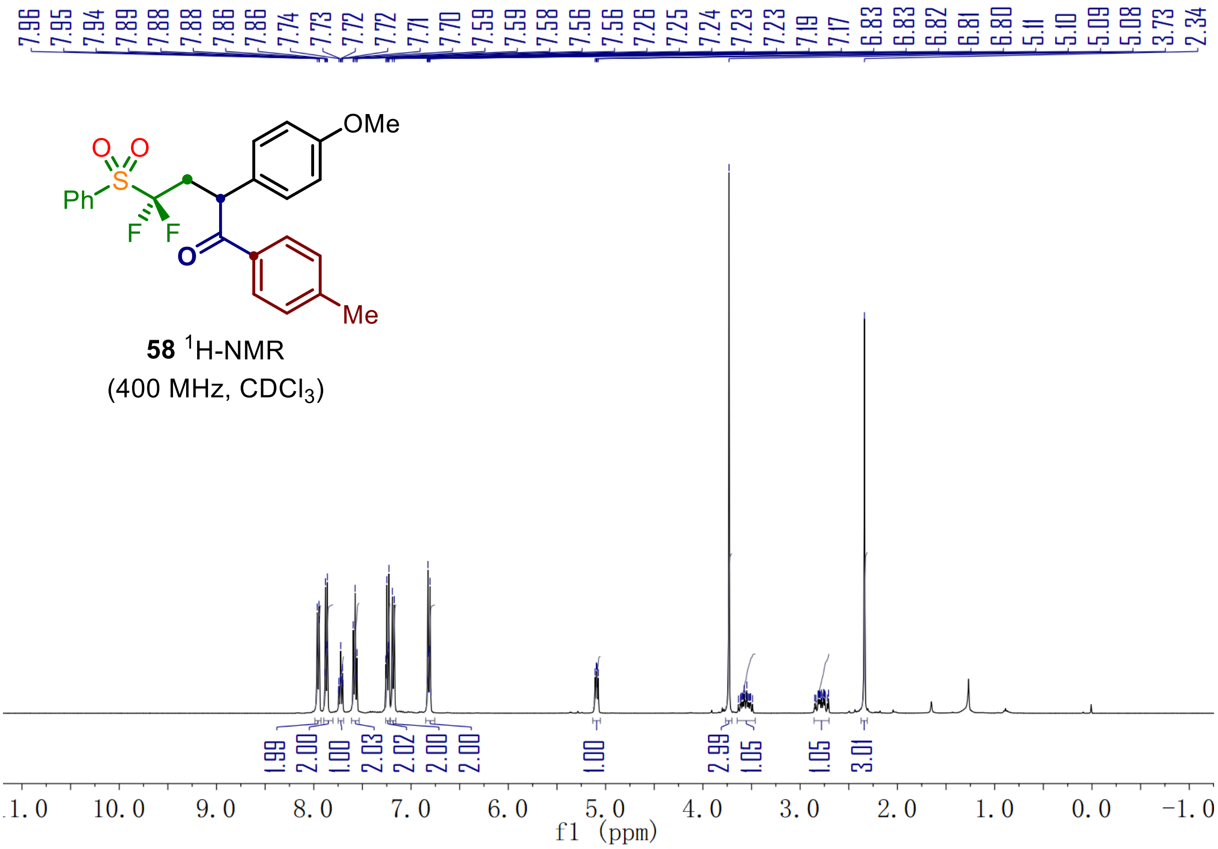

7.96
7.95
7.94
7.89
7.88
7.88
7.86
7.86
7.74
7.73
7.72
7.72
7.71
7.70
7.59
7.59
7.58
7.56
7.56
7.26
7.25
7.24
7.23
7.23
7.19
7.17
6.83
6.83
6.82
6.81
6.80
5.11
5.10
5.09
5.08
3.73
2.34
OMe
O
O
Ph
S
F
F
O
Me
58 1H-NMR
(400 MHz, CDCl3)
1.99
2.00
1.00
2.03
2.02
2.00
2.00
1.00
2.99
1.05
1.05
3.01
f1 (ppm)

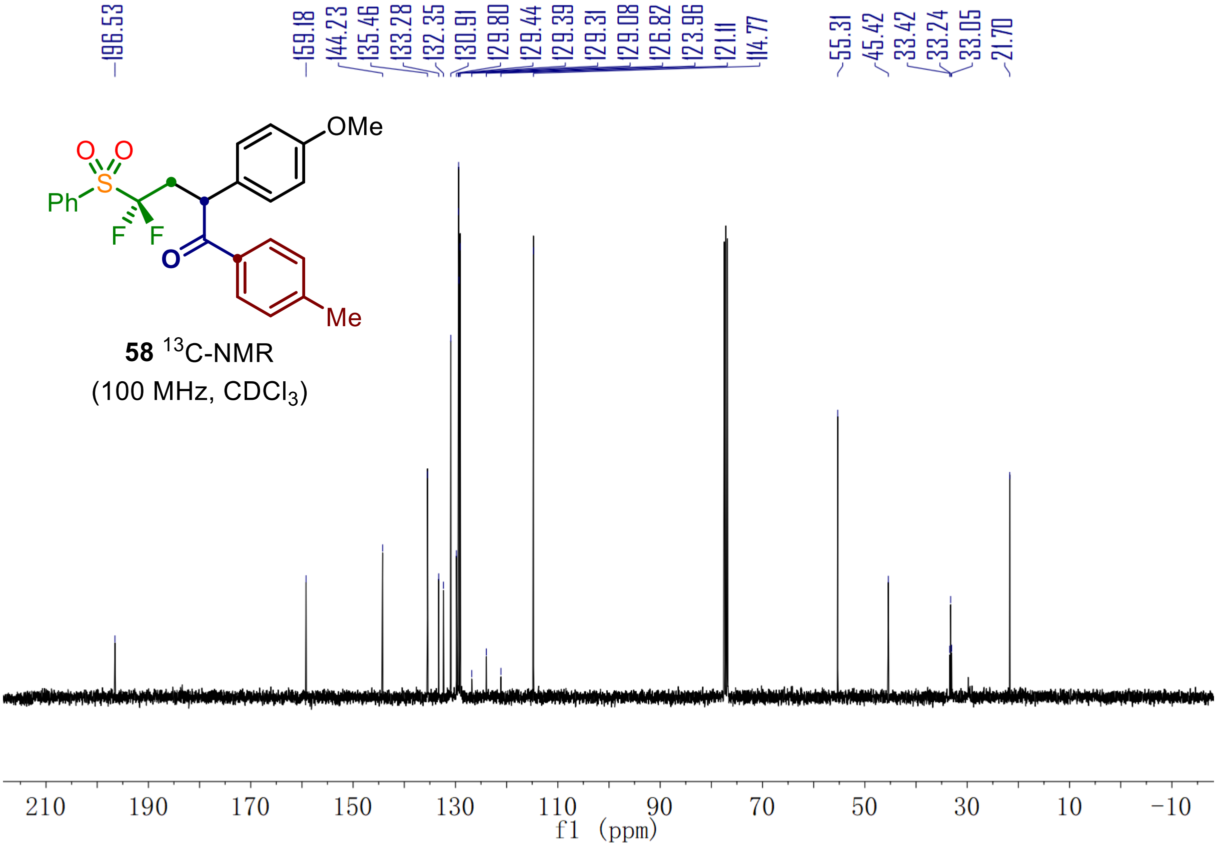

196.53
159.18
144.23
135.46
133.28
132.35
130.91
129.80
129.44
129.39
129.31
129.08
126.82
123.96
121.11
114.77
55.31
45.42
33.42
33.24
33.05
21.70
OMe
O
O
Ph
S
F
F
O
Me
58 13C-NMR
(100 MHz, CDCl3)
f1 (ppm)

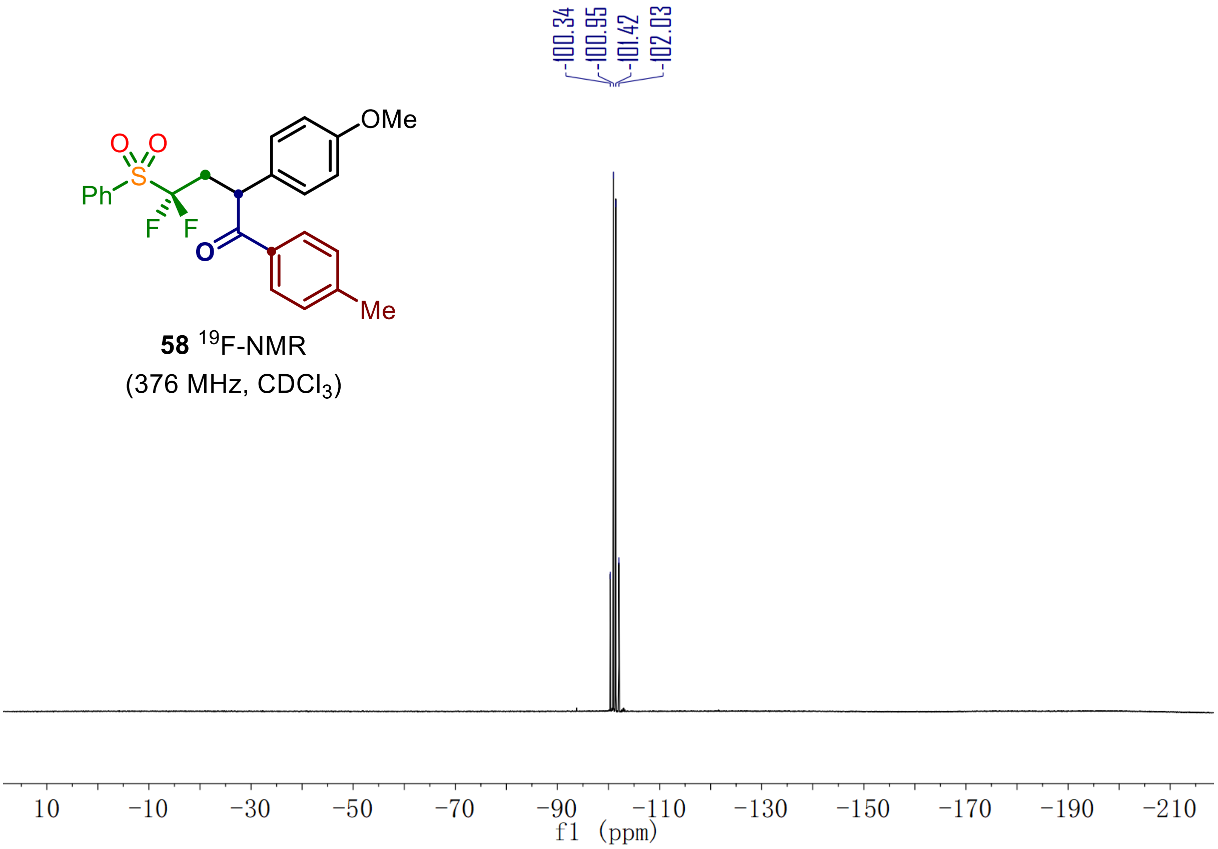

-100.34
-100.95
-101.42
-102.03
OMe
Ph
O
S
O
F
F
O
Me
58 19F-NMR
(376 MHz, CDCl3)
f1 (ppm)

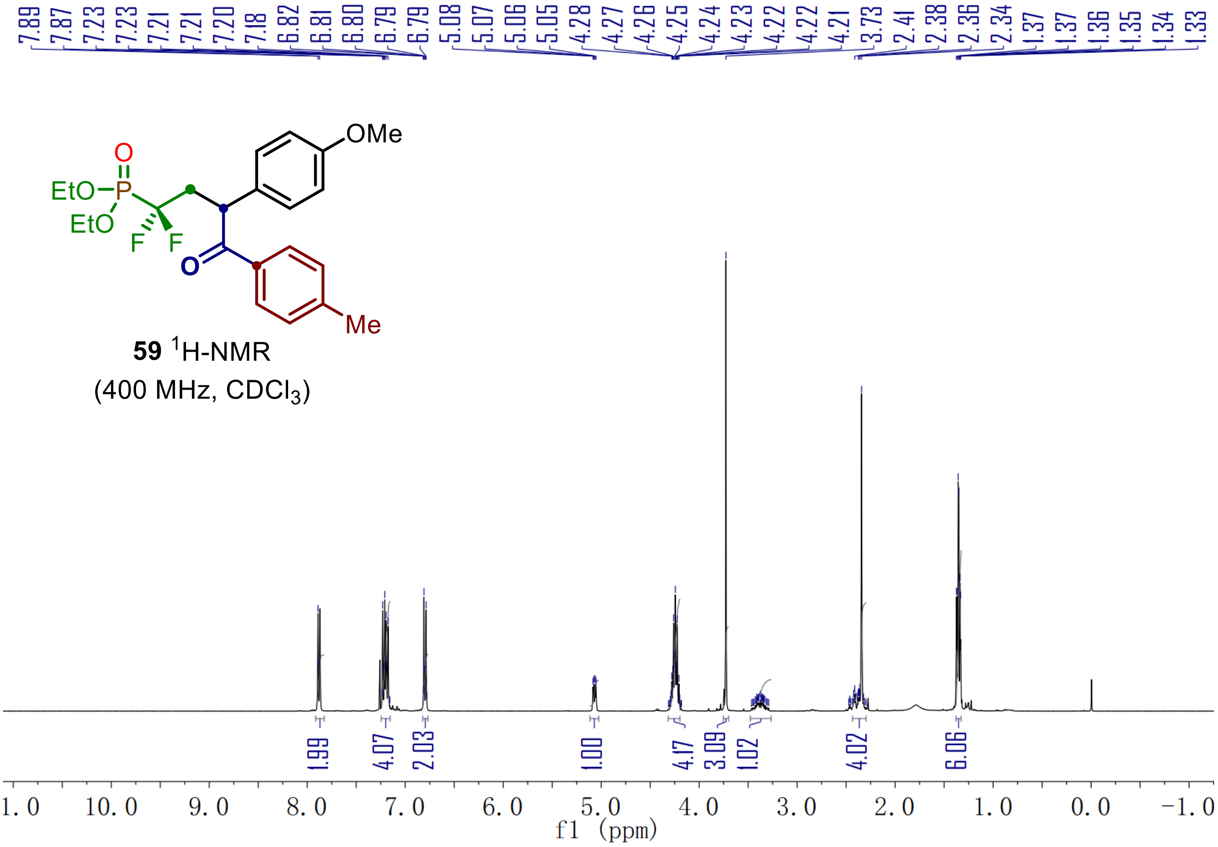

7.89 7.87 7.23 7.23 7.21 7.21 7.20 7.18 6.82 6.81 6.80 6.79 6.79 5.08 5.07 5.06 5.05 4.28 4.27 4.26 4.25 4.24 4.23 4.22 4.22 4.21 3.73 2.41 2.38 2.36 2.34 1.37 1.37 1.36 1.35 1.34 1.33
OMe
O
EtO
P
EtO
F
F
O
Me
59 1H-NMR
(400 MHz, CDCl3)
1.99 4.07 2.03 1.00 4.17 3.09 1.02 4.02 6.06
f1 (ppm)

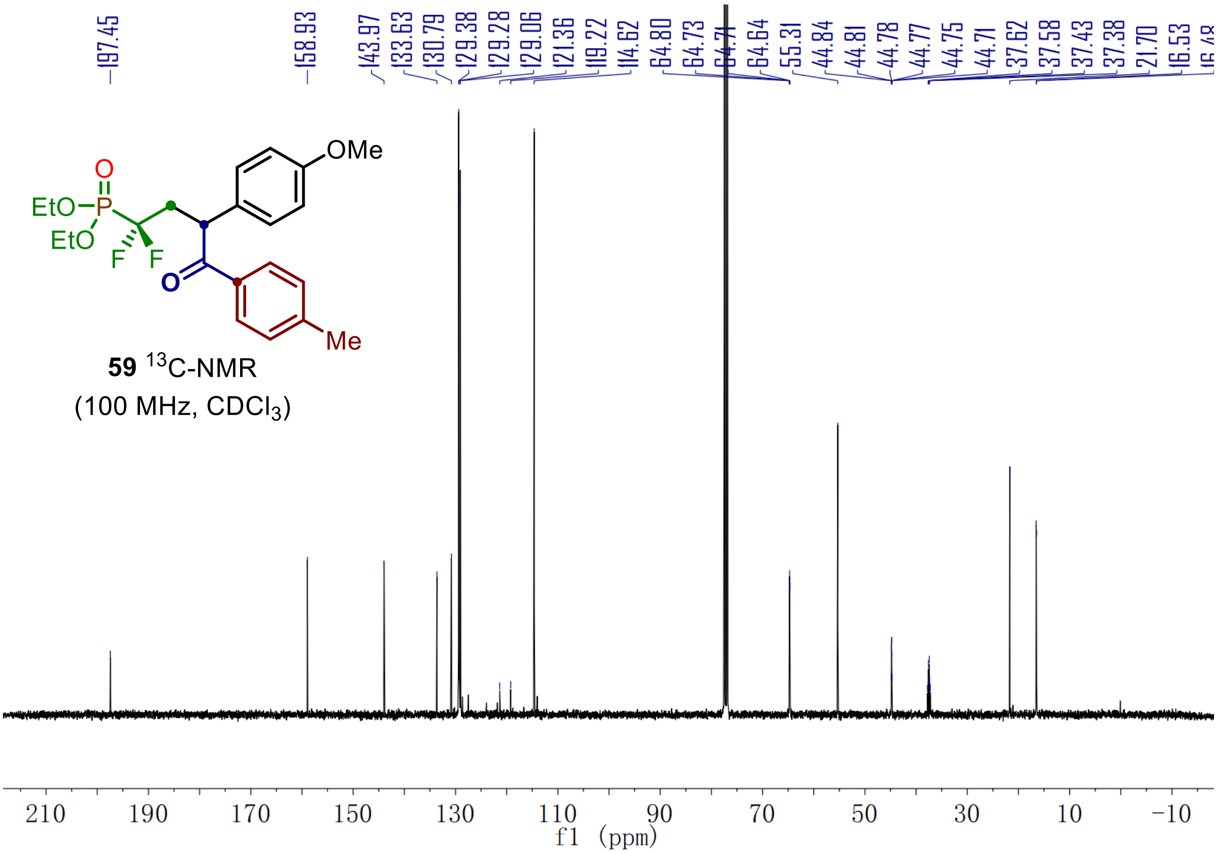

197.45
158.93
143.97
133.63
130.79
129.38
129.28
129.06
121.36
119.22
114.62
64.80
64.73
64.71
64.64
55.31
44.84
44.81
44.78
44.77
44.75
44.71
37.62
37.58
37.43
37.38
21.70
16.53
OMe
O
EtO–P
EtO
F F
O
Me
59 ^{13}C-NMR
(100 MHz, $CDCl_3$)
210 190 170 150 130 110 90 70 50 30 10 −10
f1 (ppm)

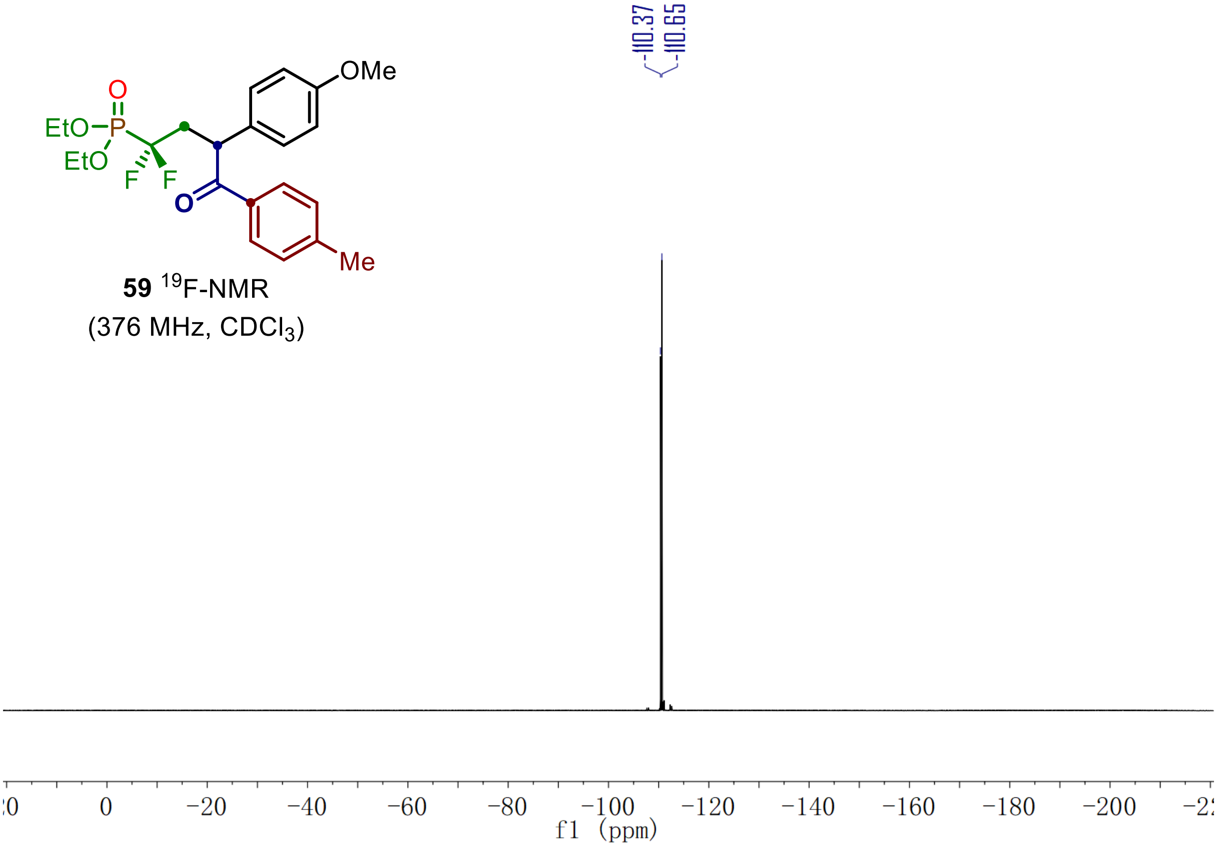

-110.37
-110.65
OMe
O
EtO–P
EtO
F F
O
Me
59 ^{19}F-NMR
(376 MHz, $CDCl_3$)
0 −20 −40 −60 −80 −100 −120 −140 −160 −180 −200
f1 (ppm)

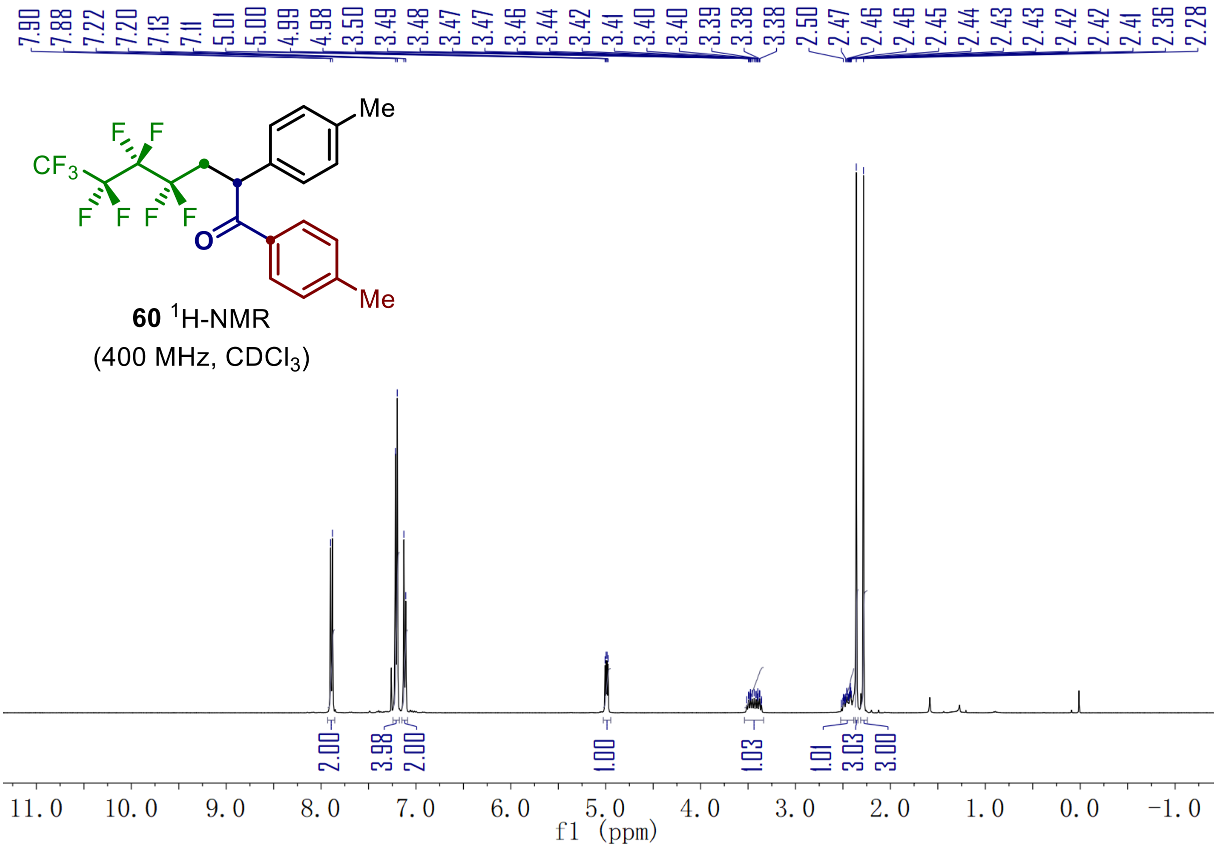

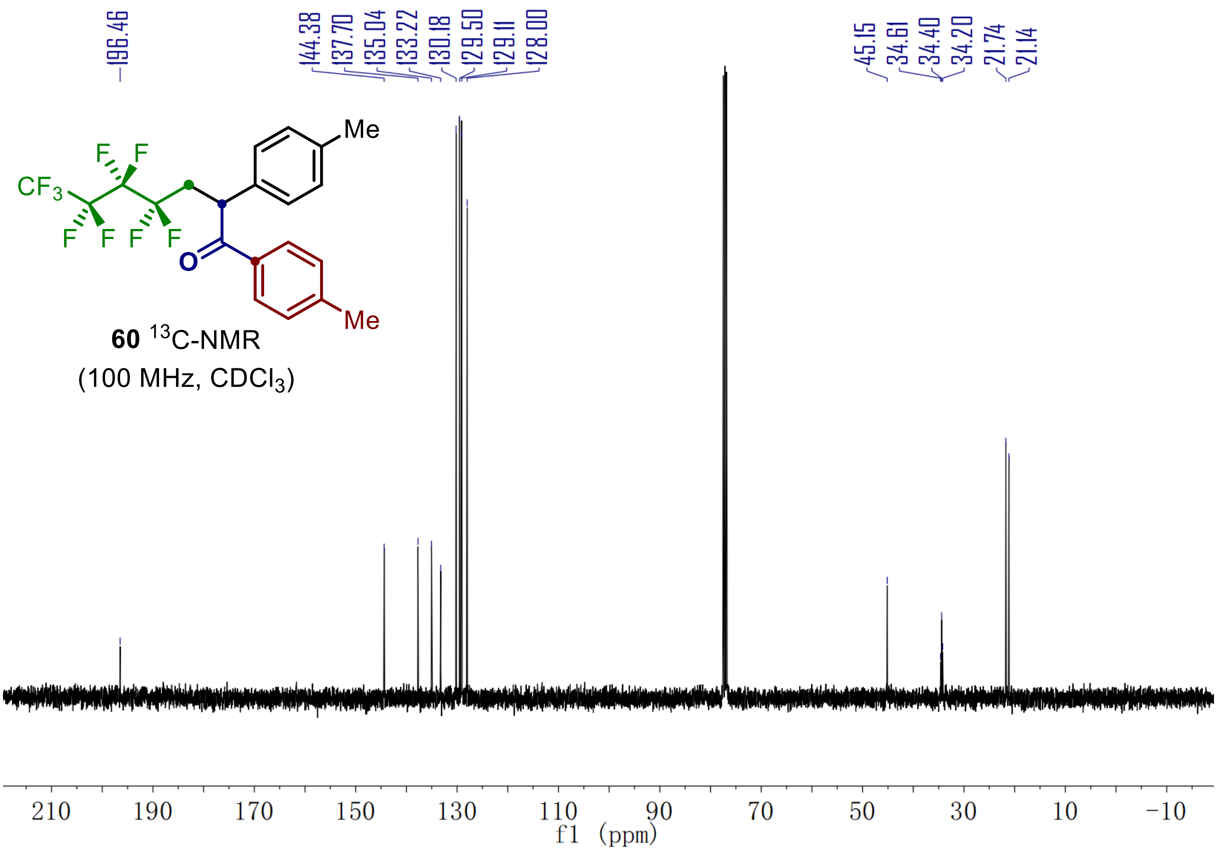

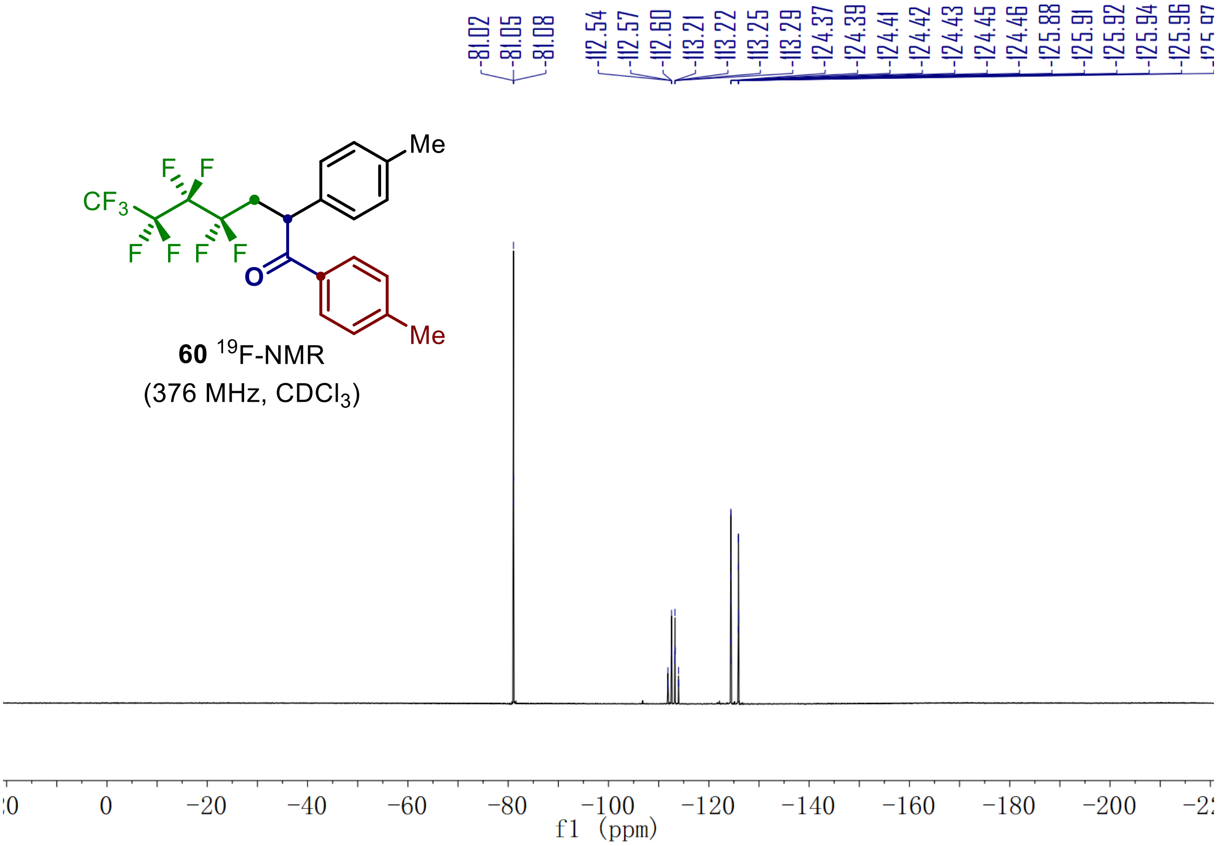

60 ^{19}F-NMR

(376 MHz, $CDCl_3$)

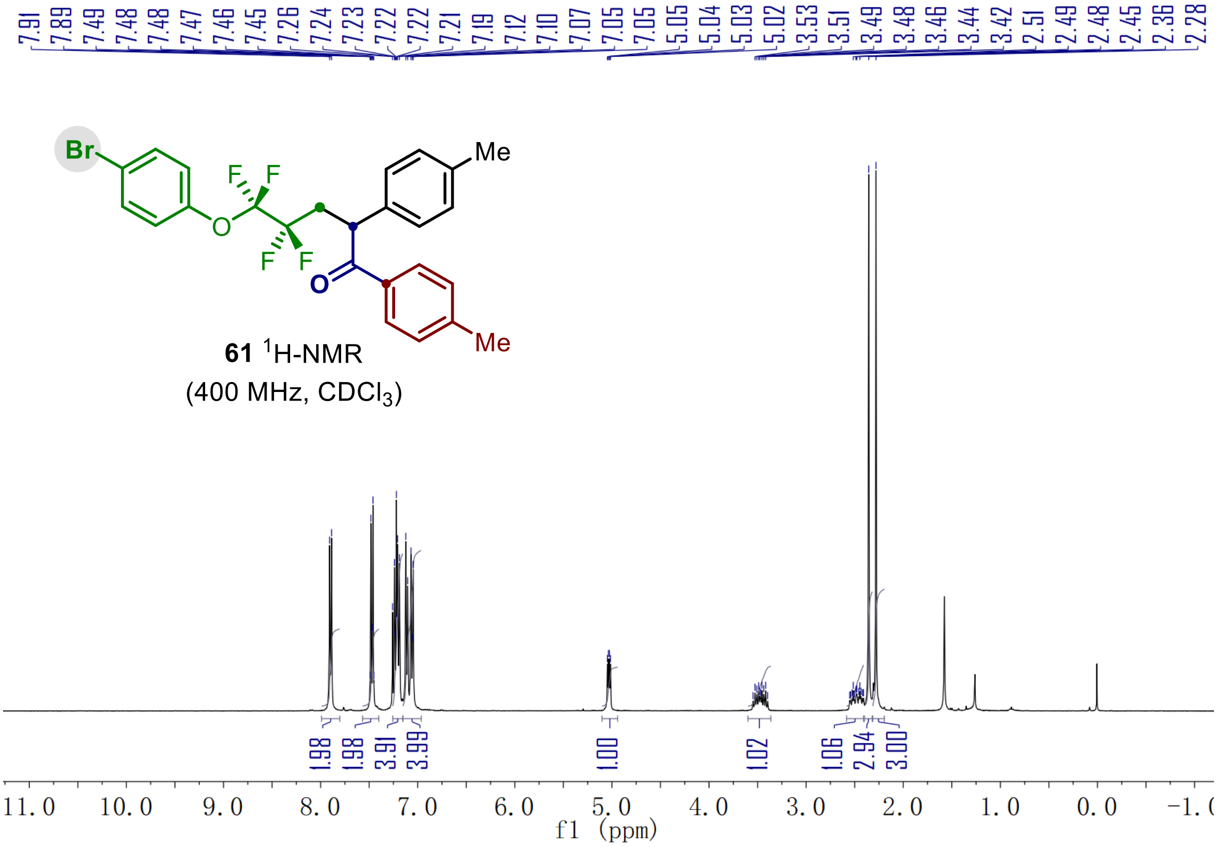

61 ^{1}H-NMR

(400 MHz, $CDCl_3$)

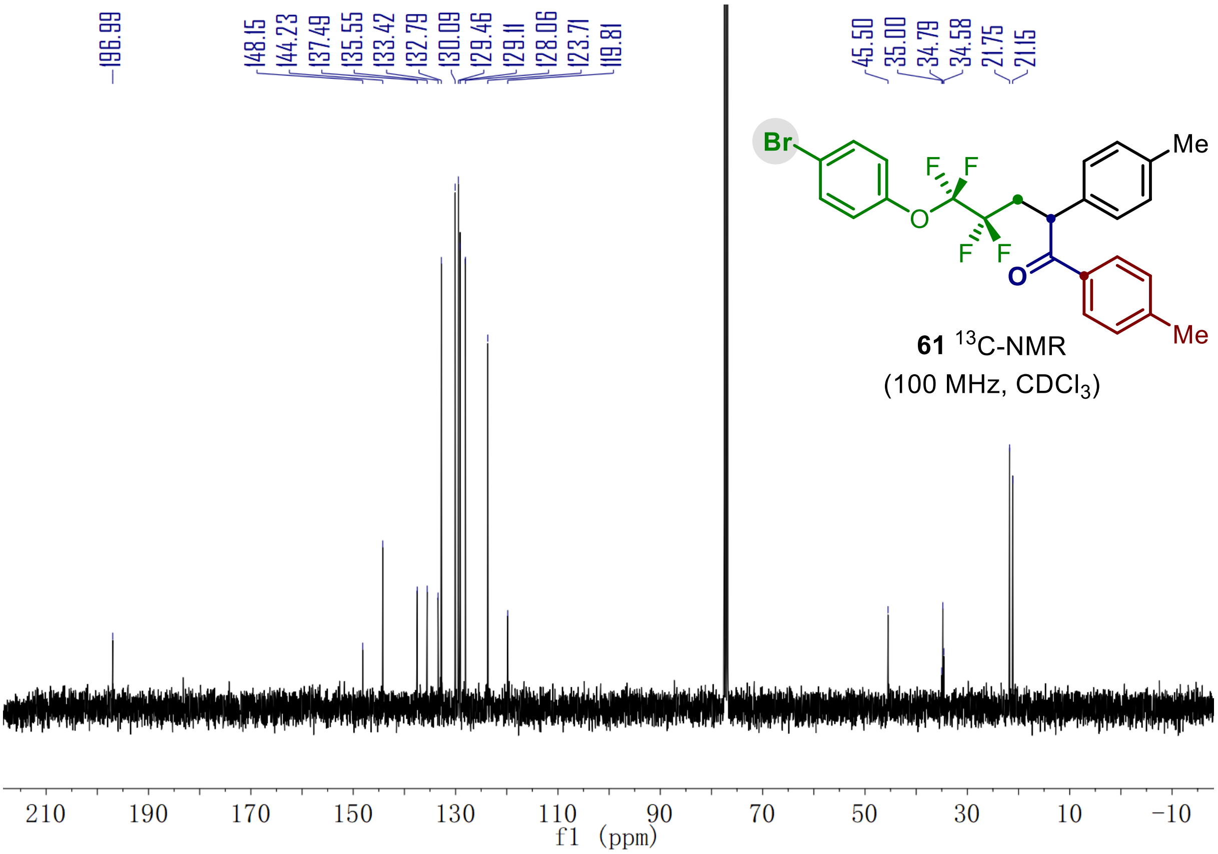

196.99
148.15
144.23
137.49
135.55
133.42
132.79
130.09
129.46
129.11
128.06
123.71
119.81
45.50
35.00
34.79
34.58
21.75
21.15
Br
F
F
F
F
O
O
Me
Me
61 13C-NMR
(100 MHz, CDCl3)
210
190
170
150
130
110
90
70
50
30
10
−10
f1 (ppm)

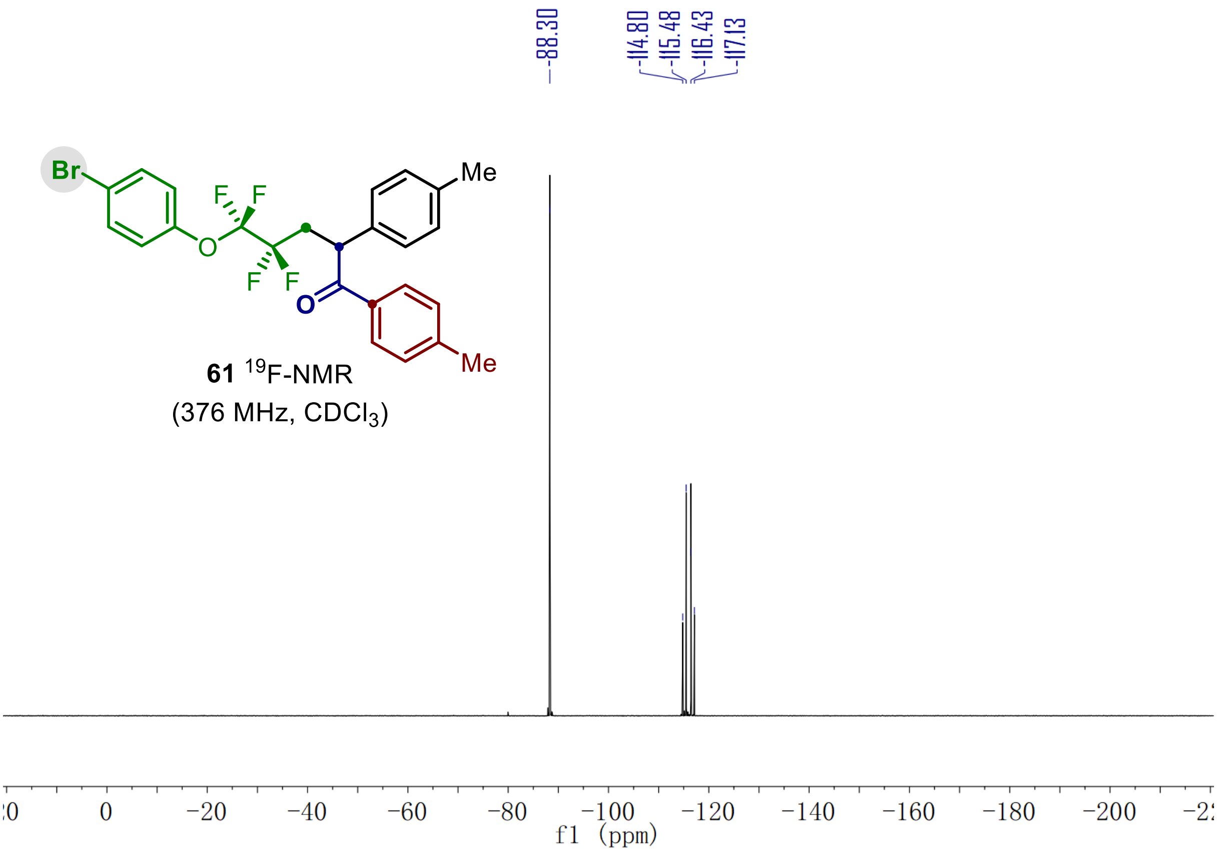

-88.30
-114.80
-115.48
-116.43
-117.13
Br
F
F
F
F
O
O
Me
Me
61 19F-NMR
(376 MHz, CDCl3)
0
−20
−40
−60
−80
−100
−120
−140
−160
−180
−200
f1 (ppm)

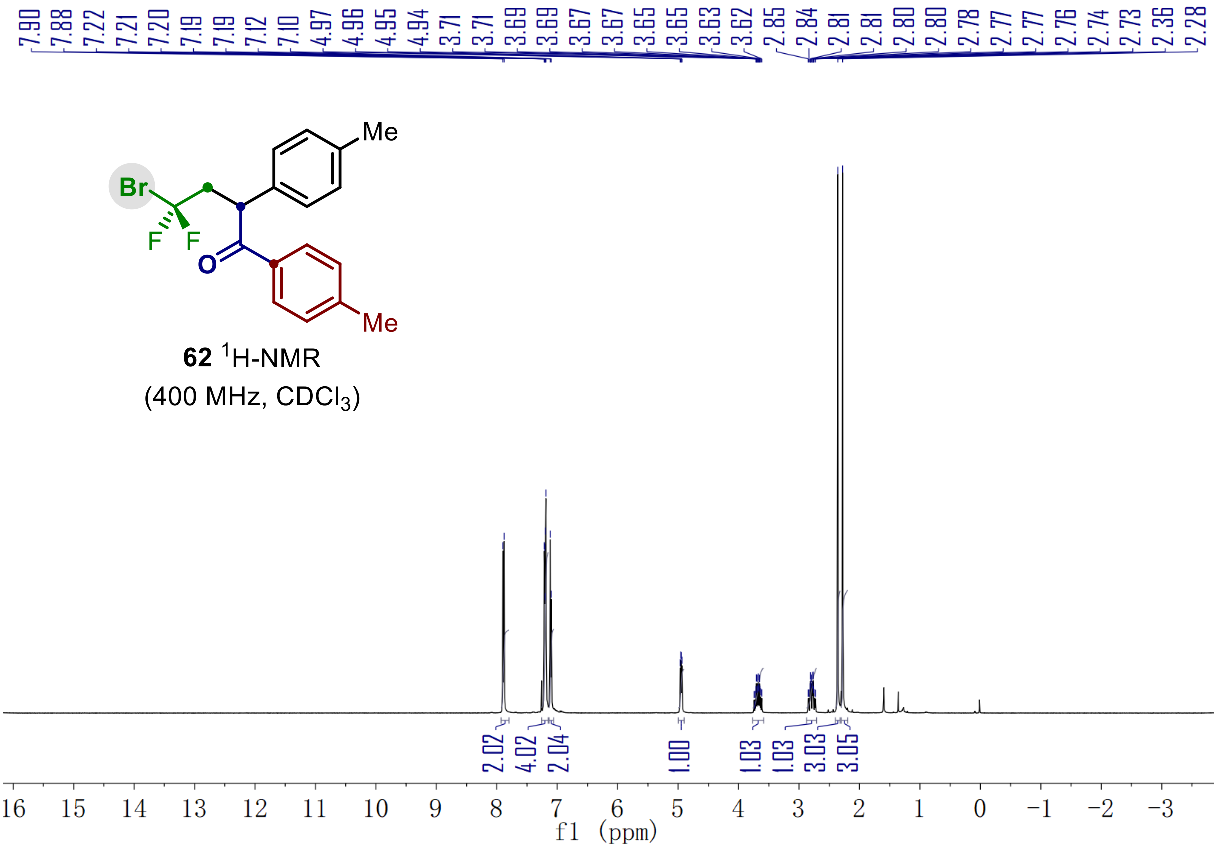
7.90 7.88 7.22 7.21 7.20 7.19 7.19 7.12 7.10 4.97 4.96 4.95 4.94 3.71 3.71 3.69 3.69 3.67 3.67 3.65 3.65 3.63 3.62 2.85 2.84 2.81 2.81 2.80 2.80 2.78 2.77 2.77 2.76 2.74 2.73 2.36 2.28
Me
Br
F
F
O
Me
62 1H-NMR
(400 MHz, CDCl3)
2.02 4.02 2.04 1.00 1.03 1.03 3.03 3.05
16 15 14 13 12 11 10 9 8 7 6 5 4 3 2 1 0 −1 −2 −3
f1 (ppm)

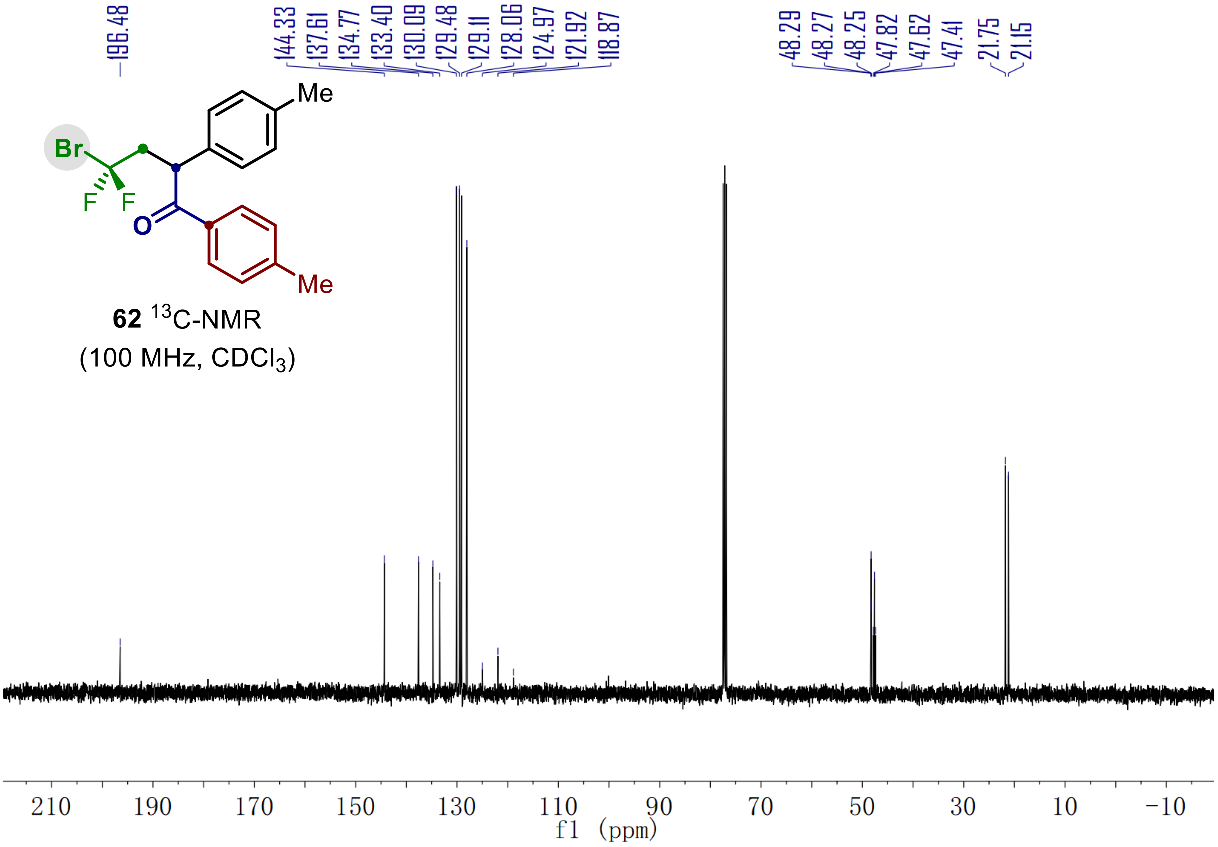
196.48
144.33 137.61 134.77 133.40 130.09 129.48 129.11 128.06 124.97 121.92 118.87
48.29 48.27 48.25 47.82 47.62 47.41 21.75 21.15
Me
Br
F
F
O
Me
62 13C-NMR
(100 MHz, CDCl3)
210 190 170 150 130 110 90 70 50 30 10 −10
f1 (ppm)

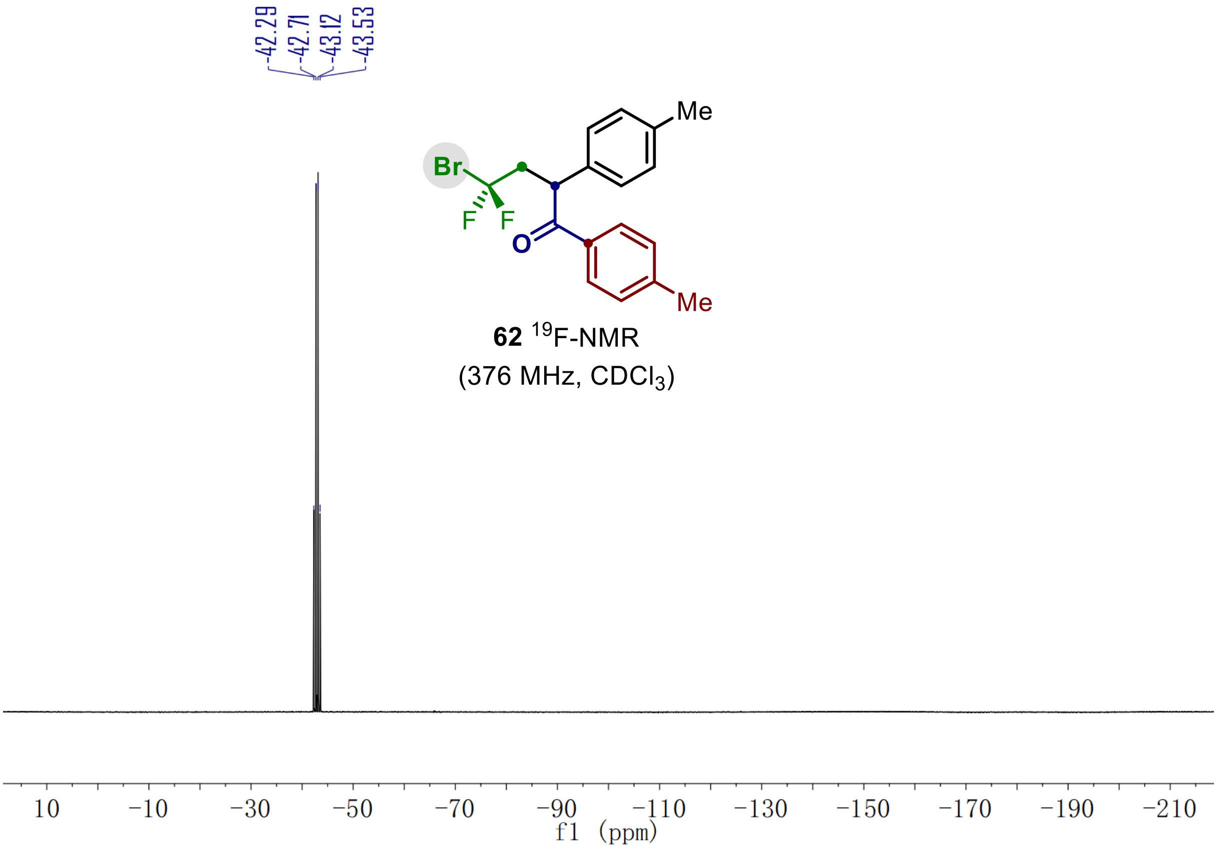
-42.29
-42.71
-43.12
-43.53
Br
F
F
O
Me
Me
62 ¹⁹F-NMR
(376 MHz, CDCl₃)
10 −10 −30 −50 −70 −90 −110 −130 −150 −170 −190 −210
f1 (ppm)

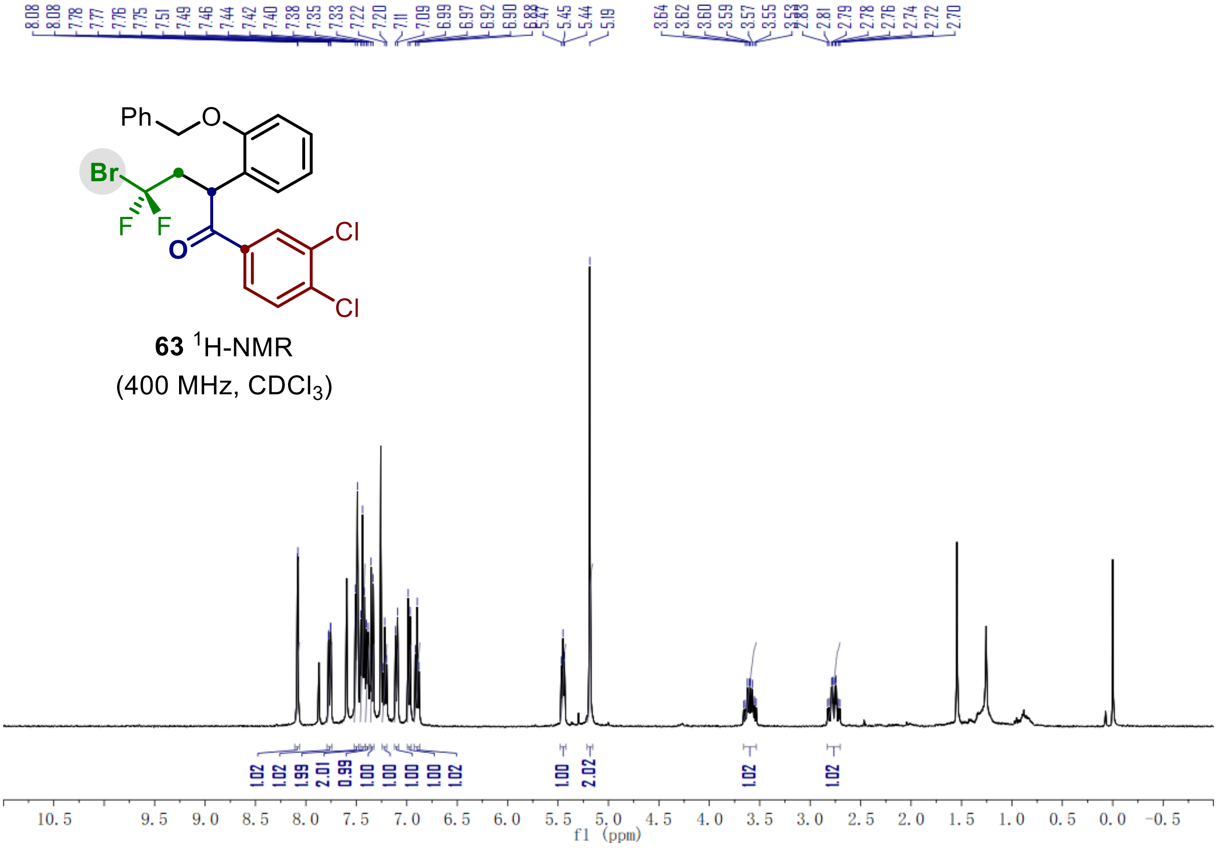
8.08
8.08
7.78
7.77
7.76
7.75
7.51
7.49
7.46
7.44
7.42
7.40
7.38
7.35
7.33
7.22
7.20
7.11
7.09
6.99
6.97
6.92
6.90
5.45
5.44
5.19
3.64
3.62
3.60
3.59
3.57
3.55
2.81
2.79
2.78
2.76
2.74
2.72
2.70
Ph
O
Br
F
F
O
Cl
Cl
63 ¹H-NMR
(400 MHz, CDCl₃)
1.02 1.02 1.99 2.01 0.99 1.00 1.00 1.00 1.02 1.00 2.02 1.02 1.02
10.5 9.5 9.0 8.5 8.0 7.5 7.0 6.5 6.0 5.5 5.0 4.5 4.0 3.5 3.0 2.5 2.0 1.5 1.0 0.5 0.0 −0.5
f1 (ppm)

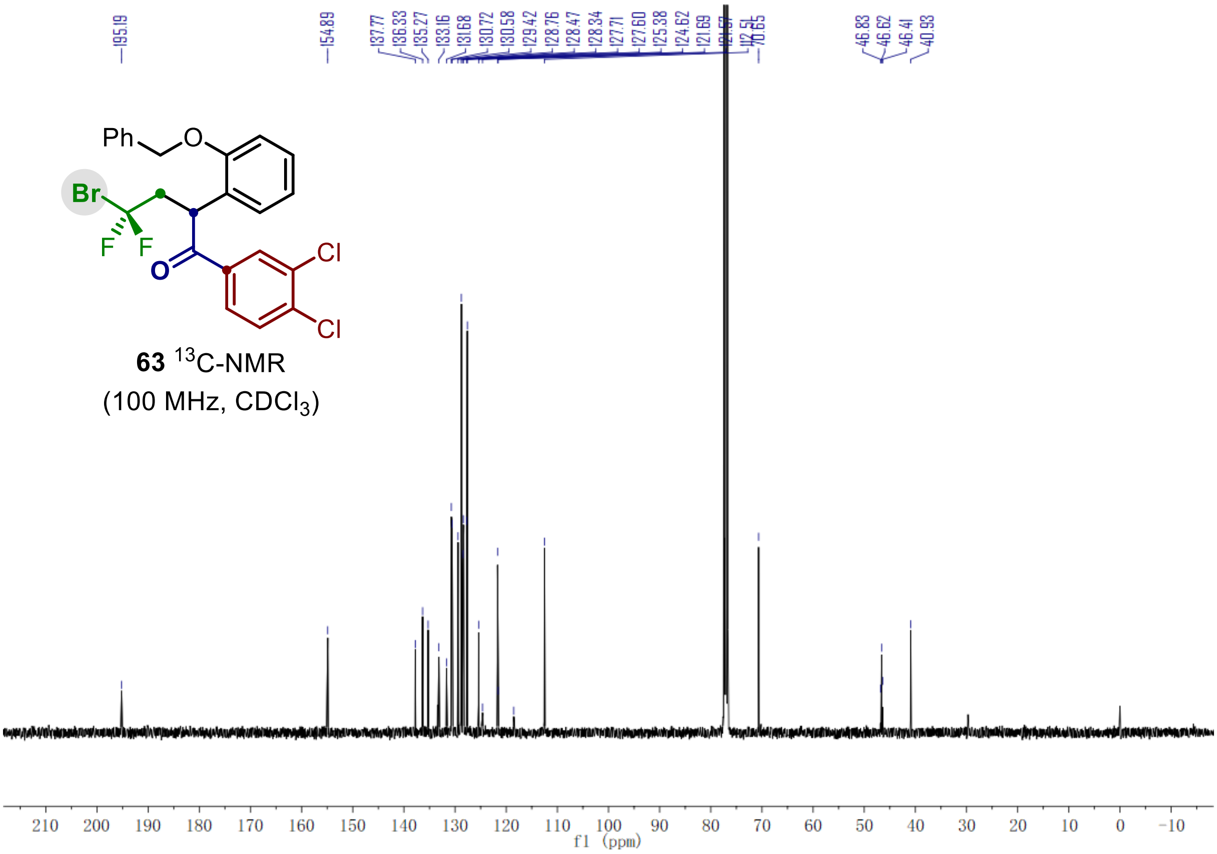

63 ^{13}C-NMR (100 MHz, $CDCl_3$)

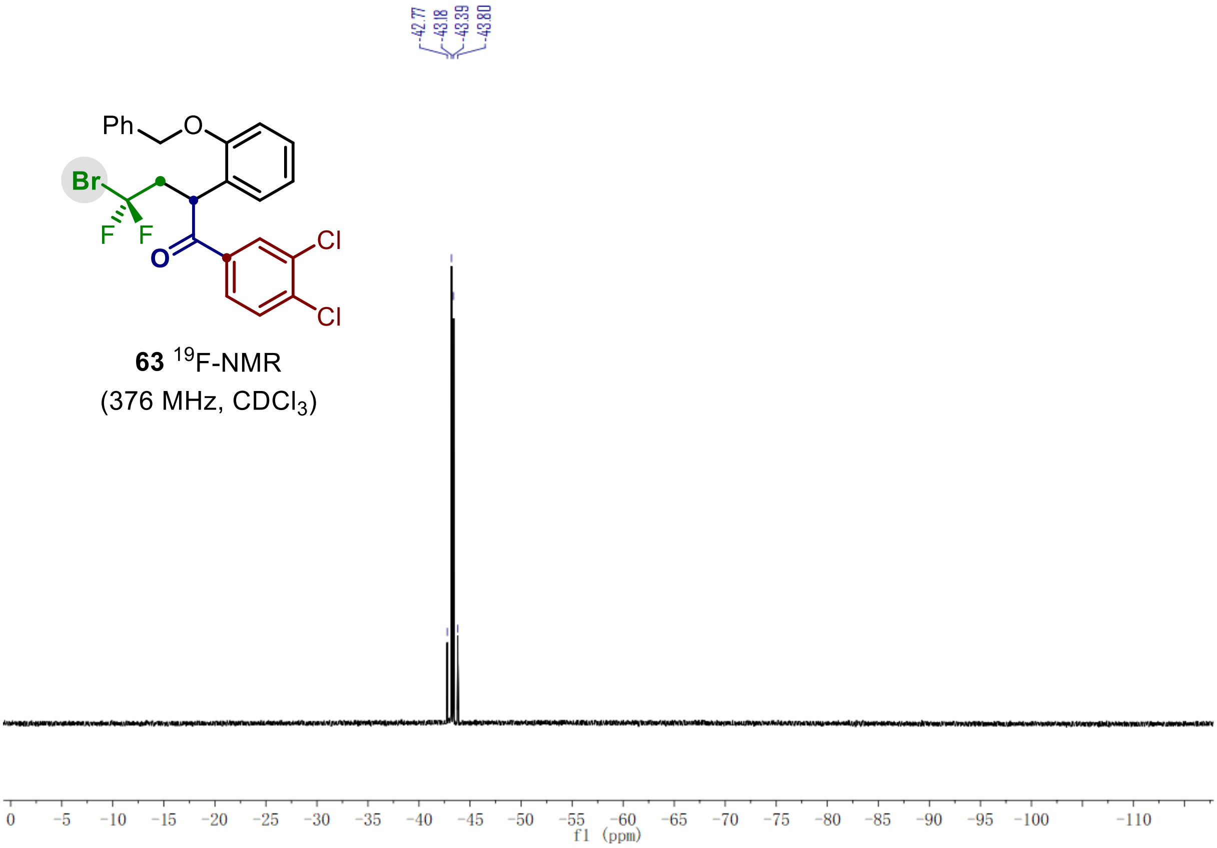

63 ^{19}F-NMR (376 MHz, $CDCl_3$)

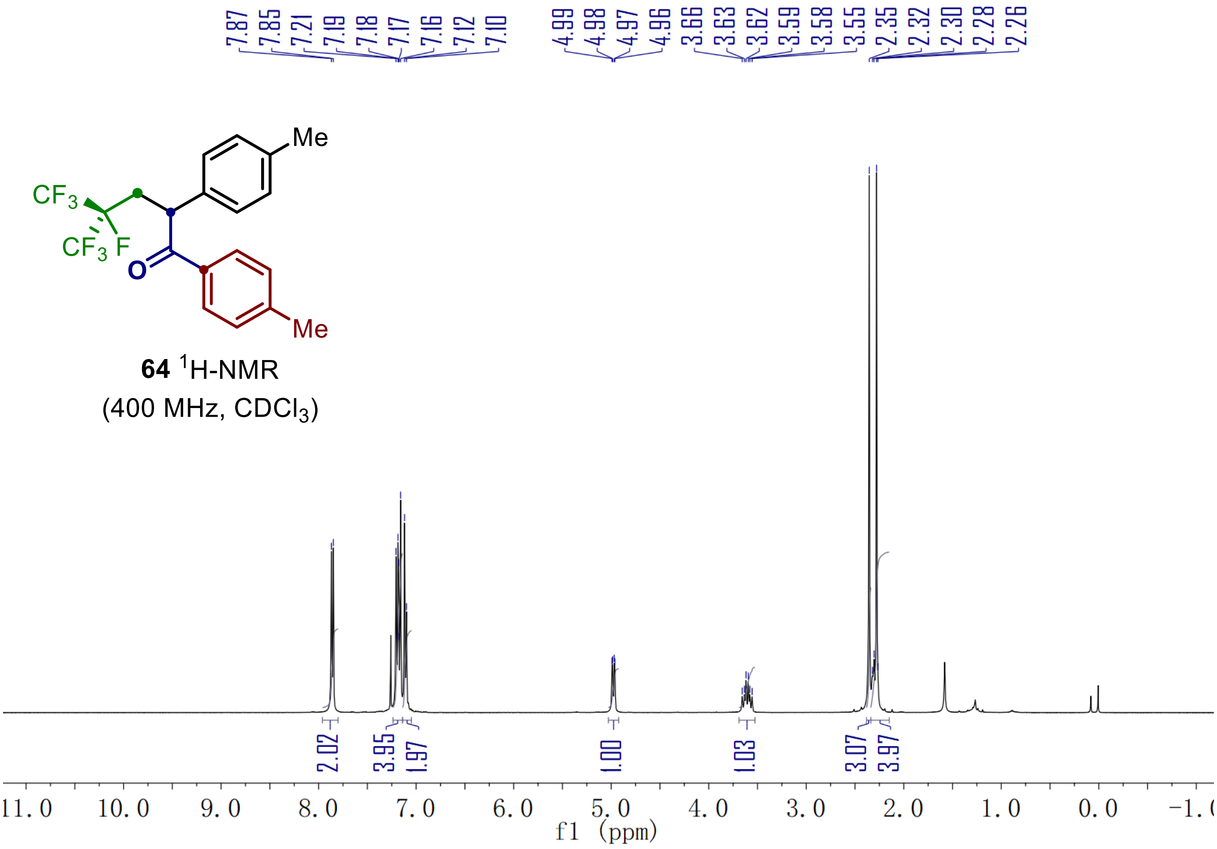
7.87
7.85
7.21
7.19
7.18
7.17
7.16
7.12
7.10
4.99
4.98
4.97
4.96
3.66
3.63
3.62
3.59
3.58
3.55
2.35
2.32
2.30
2.28
2.26
CF3
CF3 F
O
Me
Me
64 1H-NMR
(400 MHz, CDCl3)
2.02
3.95
1.97
1.00
1.03
3.07
3.97
11.0
10.0
9.0
8.0
7.0
6.0
5.0
4.0
3.0
2.0
1.0
0.0
f1 (ppm)

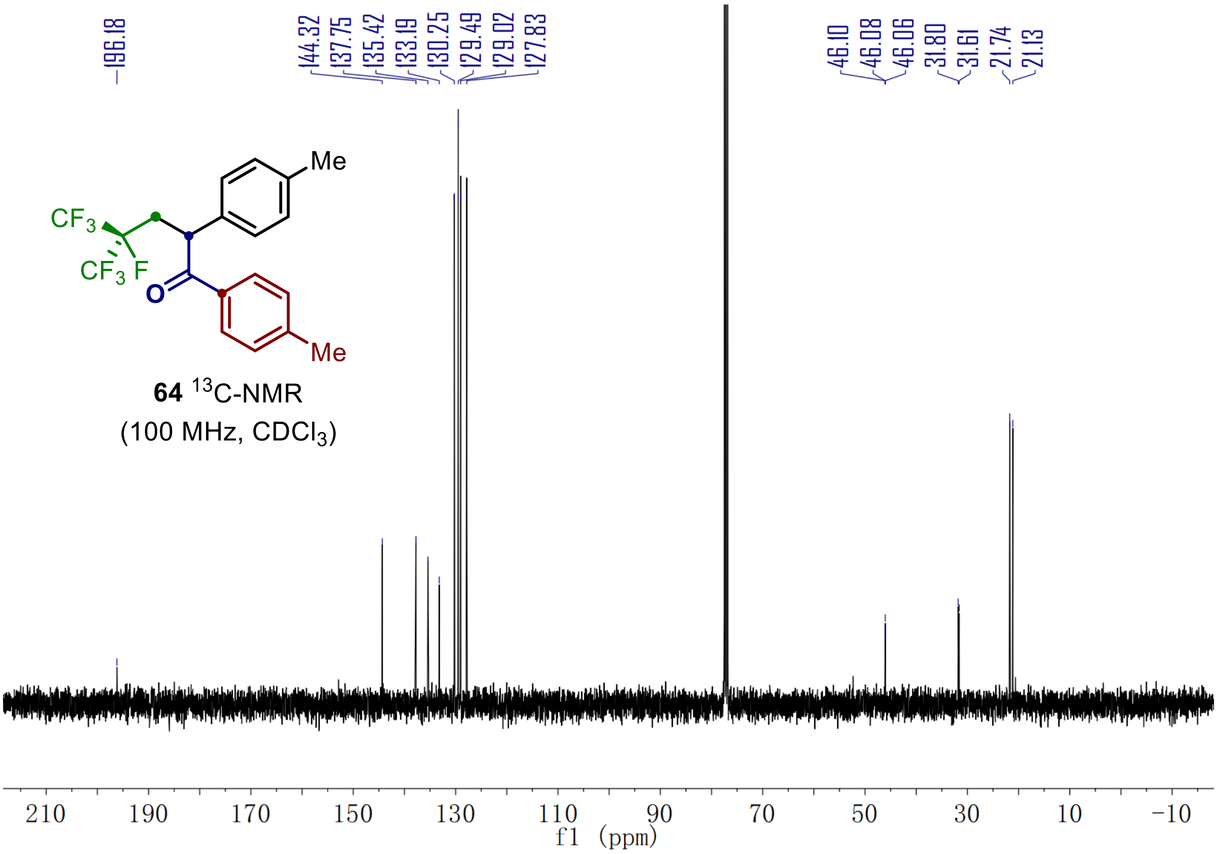
196.18
144.32
137.75
135.42
133.19
130.25
129.49
129.02
127.83
46.10
46.08
46.06
31.80
31.61
21.74
21.13
CF3
CF3 F
O
Me
Me
64 13C-NMR
(100 MHz, CDCl3)
210
190
170
150
130
110
90
70
50
30
10
-10
f1 (ppm)

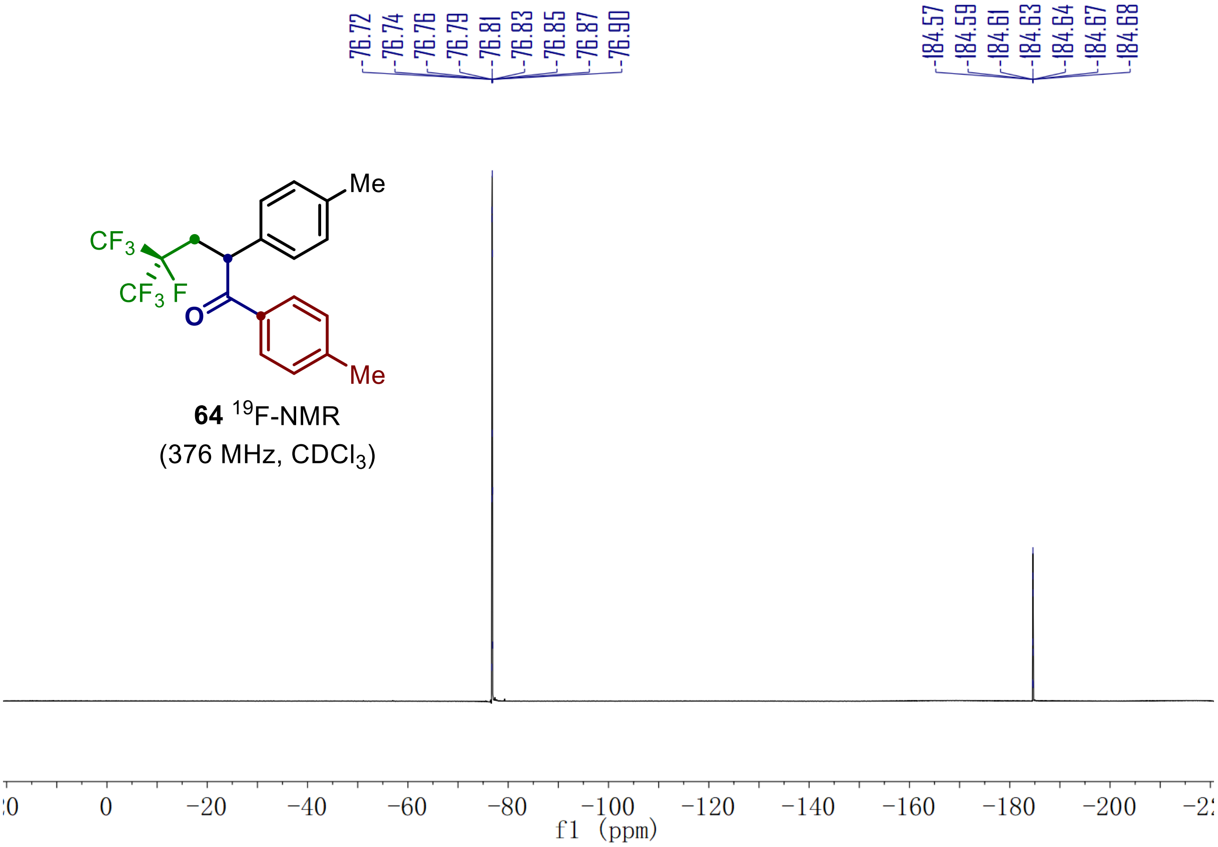

64 ^{19}F-NMR
(376 MHz, $CDCl_3$)

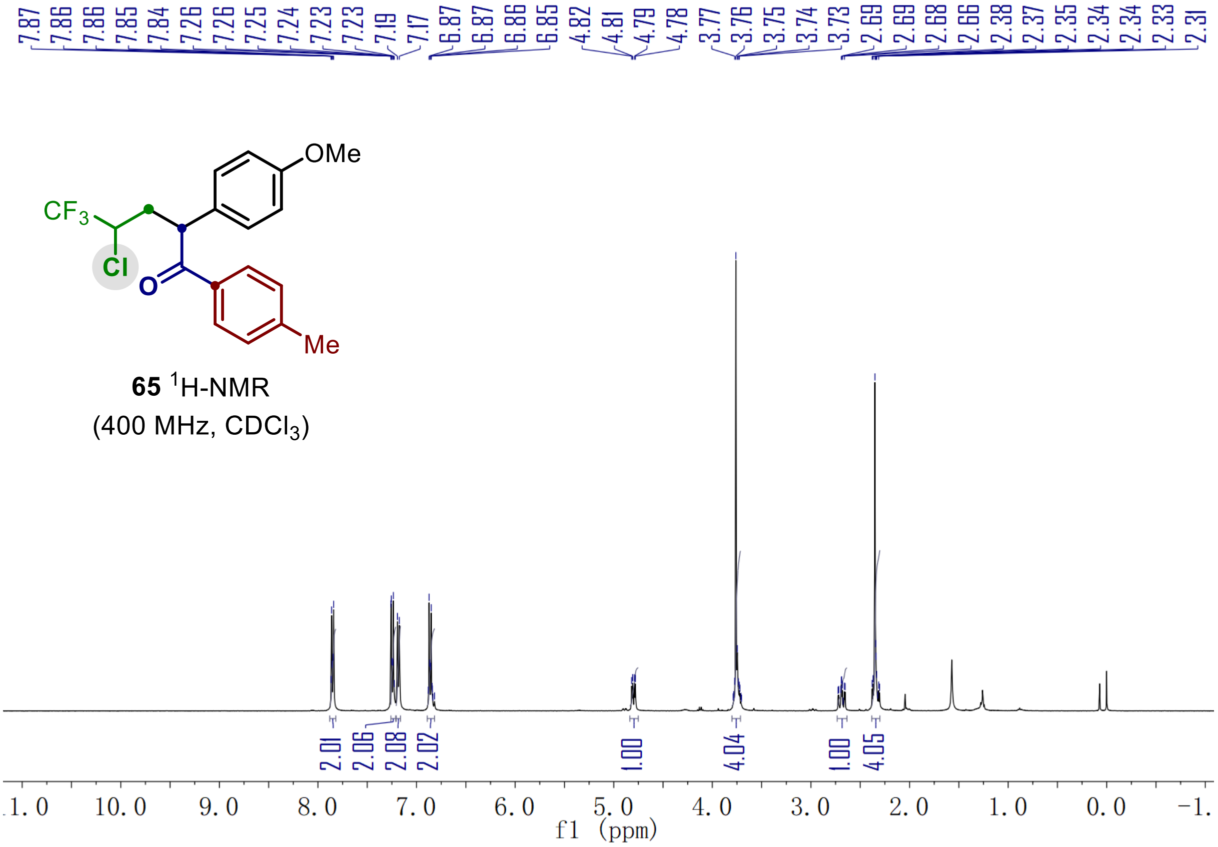

65 ^{1}H-NMR
(400 MHz, $CDCl_3$)

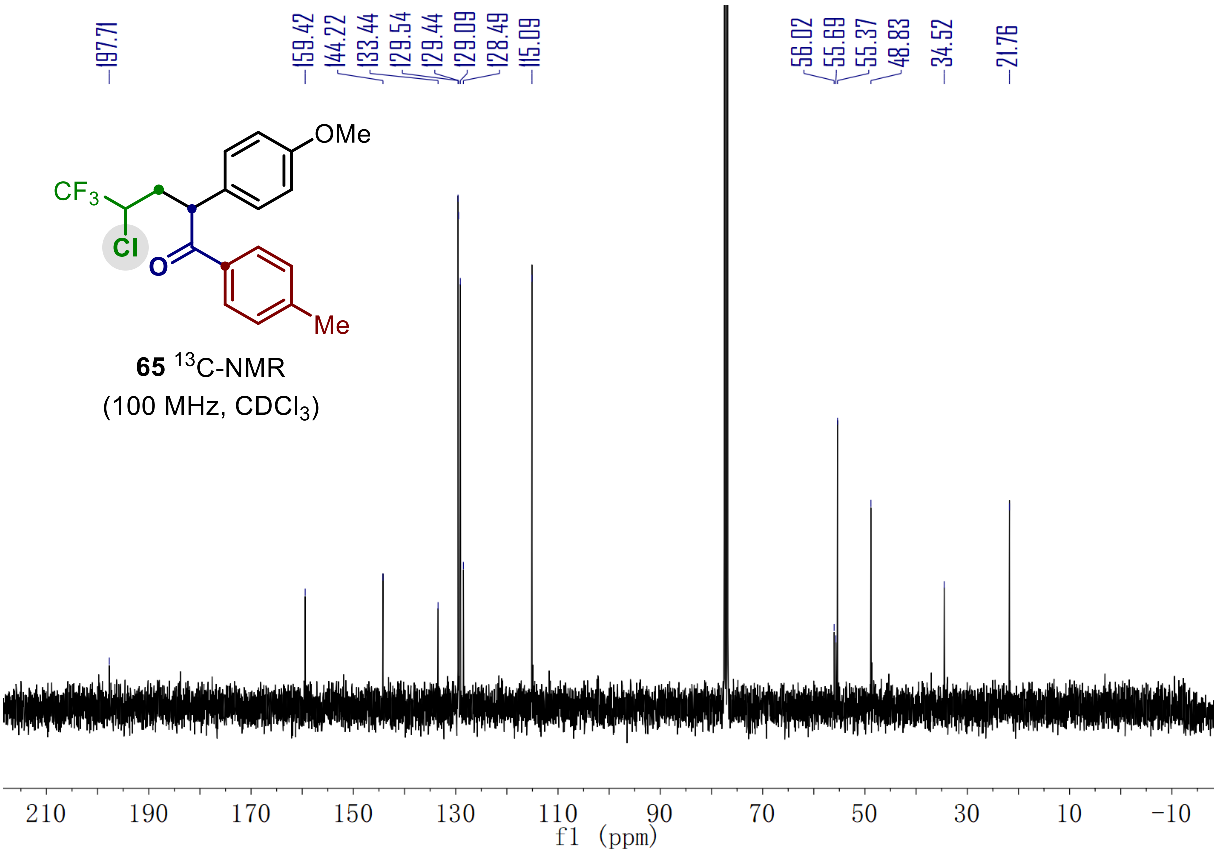
197.71
159.42
144.22
133.44
129.54
129.44
129.09
128.49
115.09
56.02
55.69
55.37
48.83
34.52
21.76
OMe
CF3
Cl
O
Me
65 13C-NMR
(100 MHz, CDCl3)
210 190 170 150 130 110 90 70 50 30 10 −10
f1 (ppm)

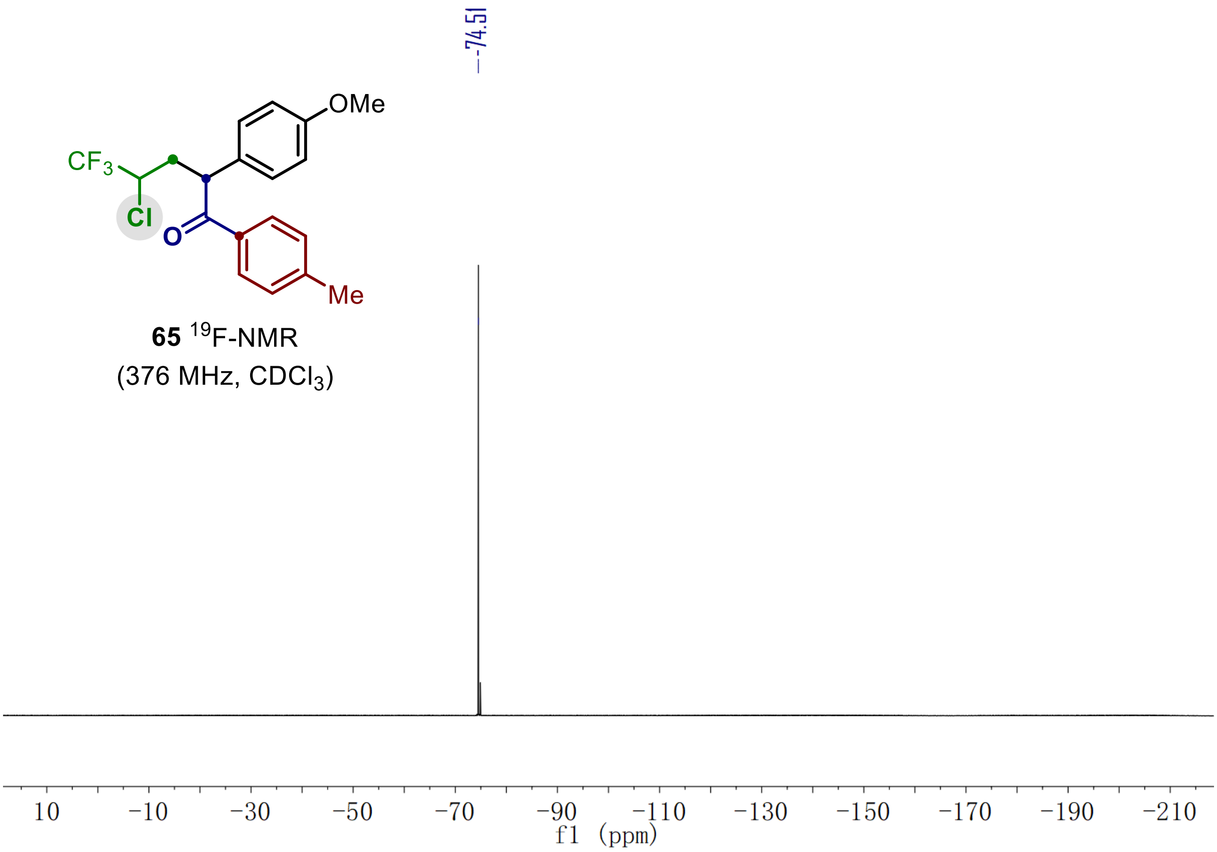
-74.51
OMe
CF3
Cl
O
Me
65 19F-NMR
(376 MHz, CDCl3)
10 −10 −30 −50 −70 −90 −110 −130 −150 −170 −190 −210
f1 (ppm)

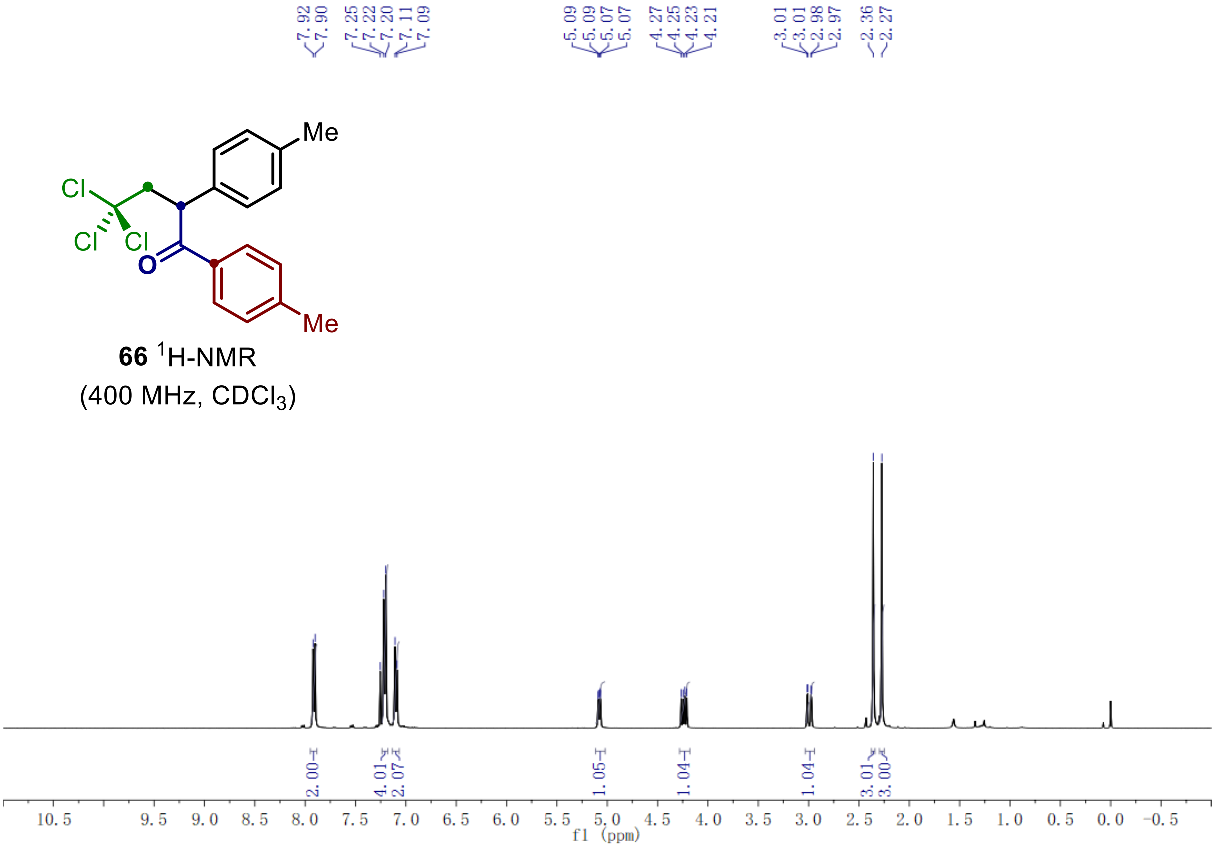

66 ^{1}H-NMR (400 MHz, $CDCl_3$)

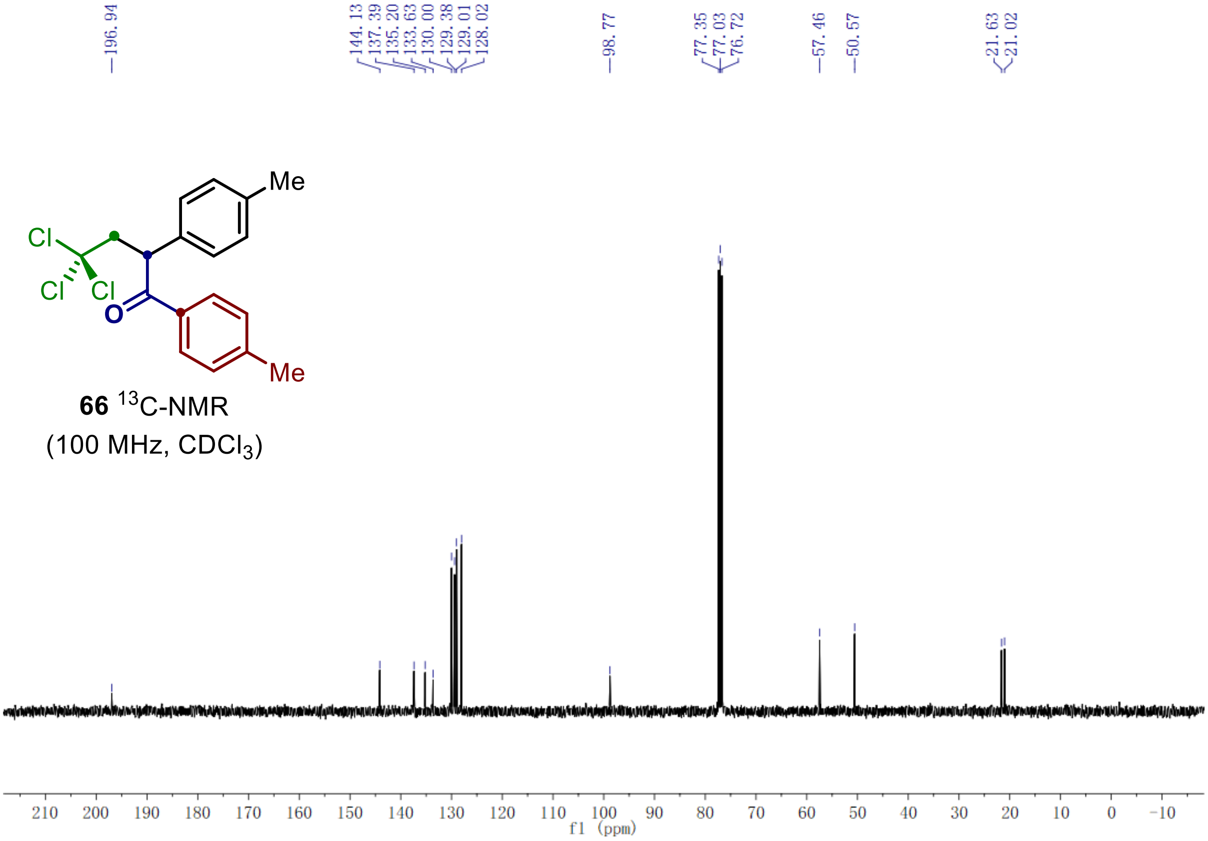

66 ^{13}C-NMR (100 MHz, $CDCl_3$)

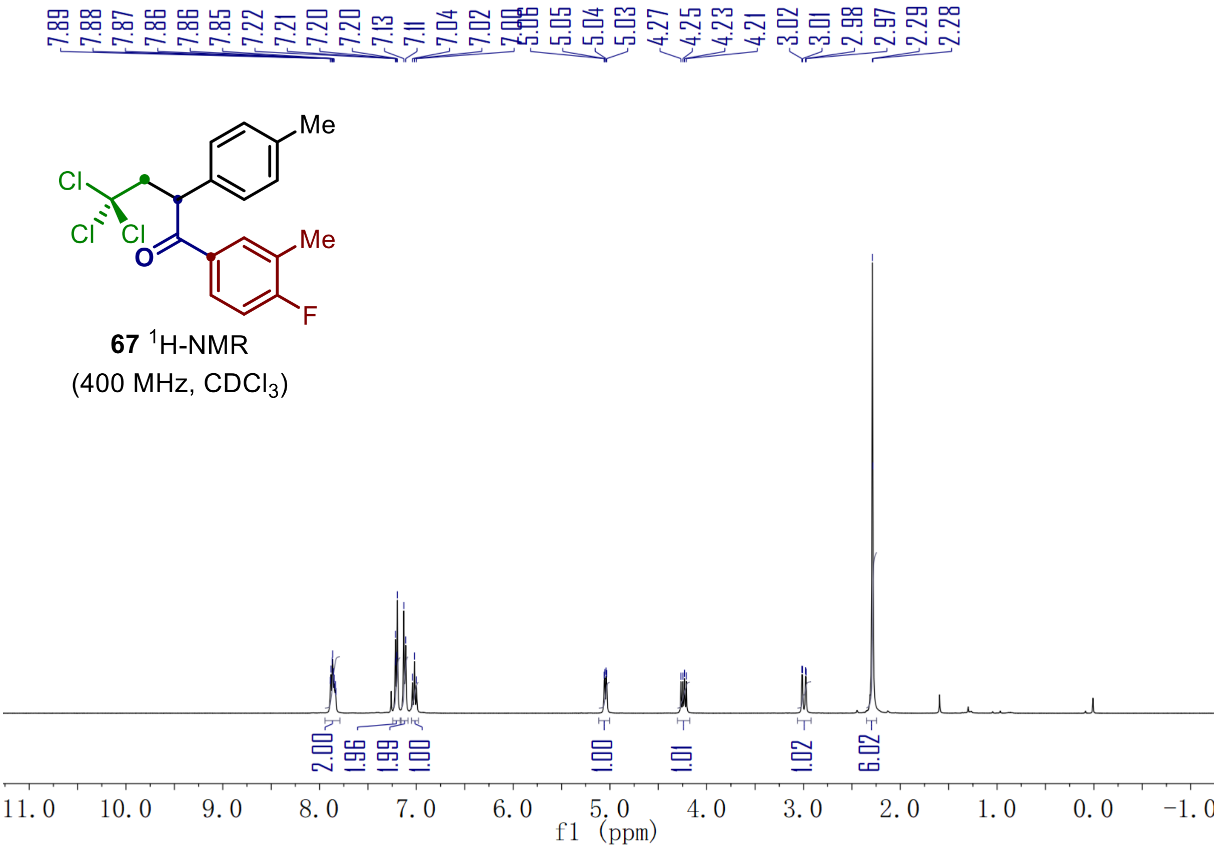
7.89 7.88 7.87 7.86 7.86 7.85 7.22 7.21 7.20 7.20 7.13 7.11 7.04 7.02 7.00 5.06 5.05 5.04 5.03 4.27 4.25 4.23 4.21 3.02 3.01 2.98 2.97 2.29 2.28
Me
Cl
Cl
Cl
O
Me
F
67 ¹H-NMR
(400 MHz, CDCl3)
2.00 1.96 1.99 1.00 1.00 1.01 1.02 6.02
11.0 10.0 9.0 8.0 7.0 6.0 5.0 4.0 3.0 2.0 1.0 0.0 -1.0
f1 (ppm)

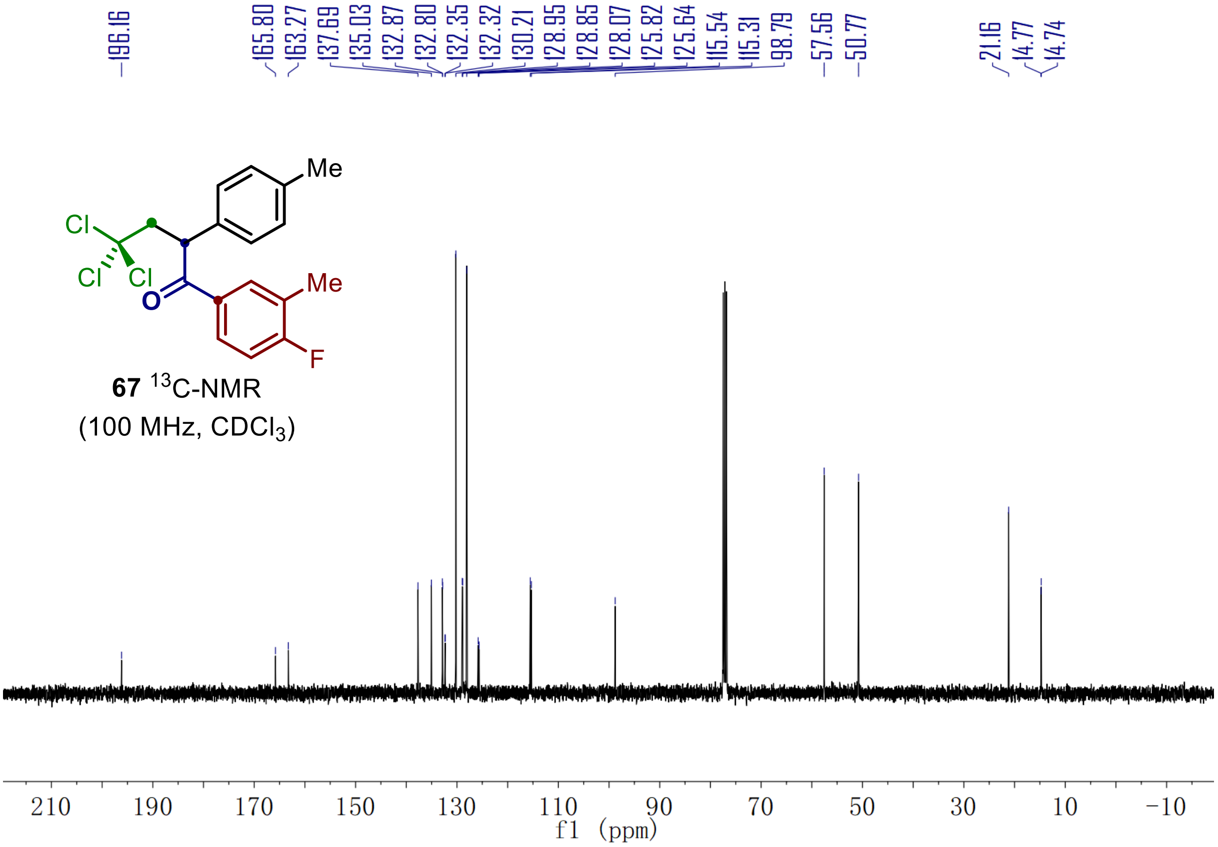
196.16 165.80 163.27 137.69 135.03 132.87 132.80 132.35 132.32 130.21 128.95 128.85 128.07 125.82 125.64 115.54 115.31 98.79 57.56 50.77 21.16 14.77 14.74
Me
Cl
Cl
Cl
O
Me
F
67 ¹³C-NMR
(100 MHz, CDCl3)
210 190 170 150 130 110 90 70 50 30 10 -10
f1 (ppm)

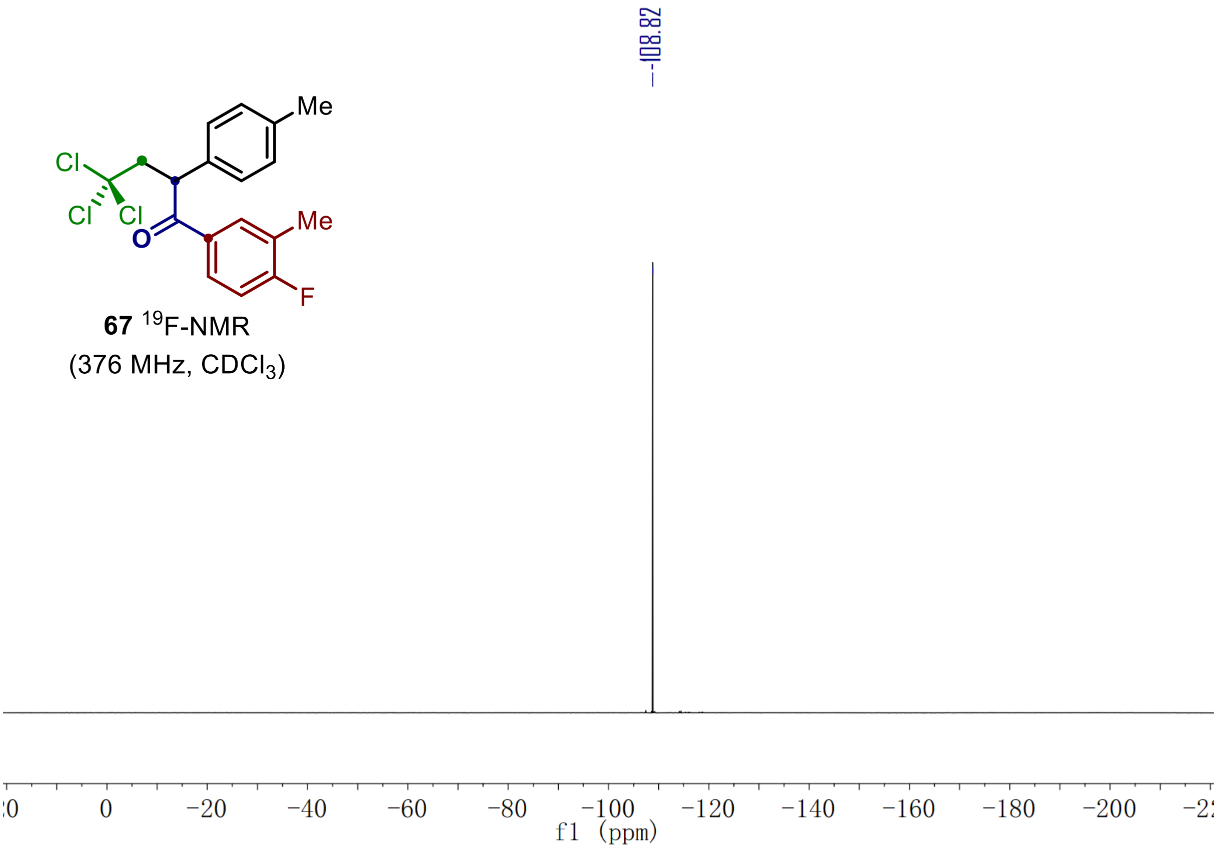
-108.82
Cl
Cl
Cl
O
Me
Me
F
67 ^{19}F-NMR
(376 MHz, $CDCl_3$)
f1 (ppm)

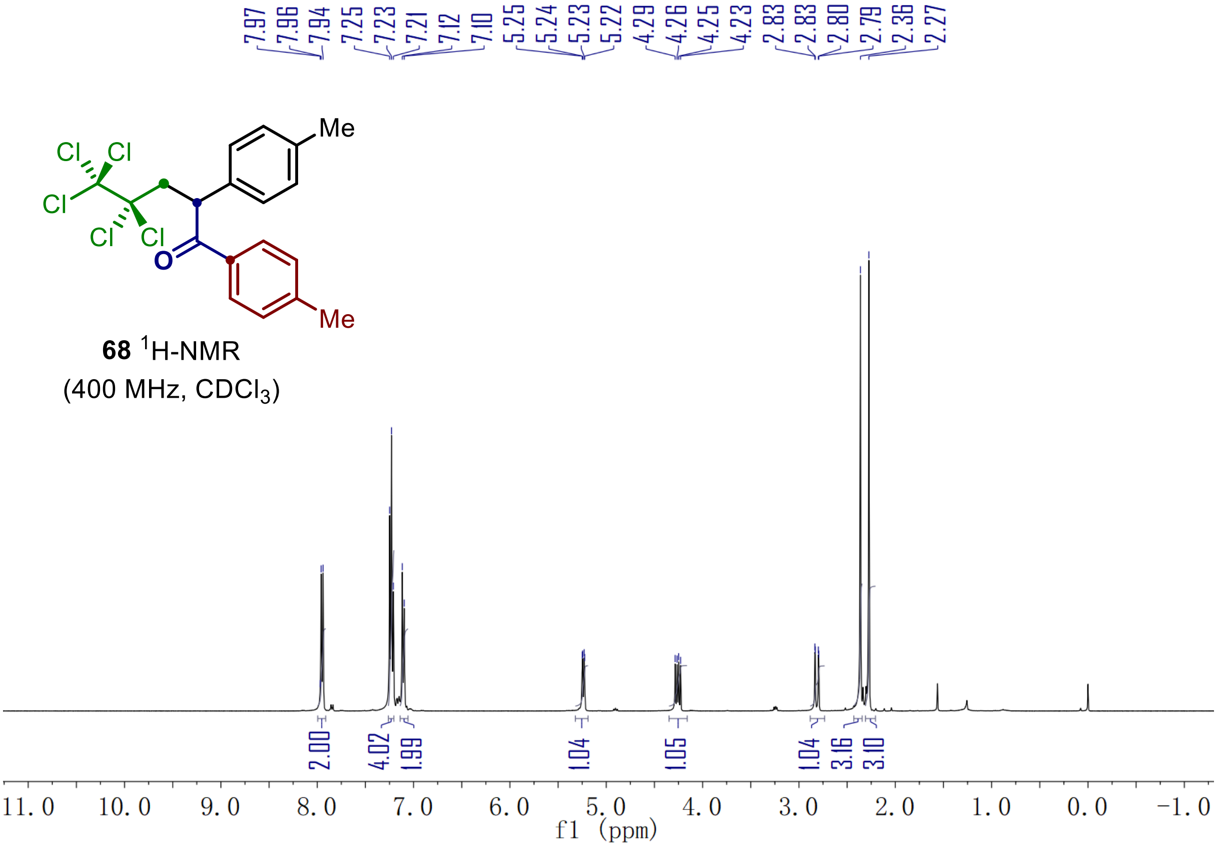
7.97 7.96 7.94 7.25 7.23 7.21 7.12 7.10 5.25 5.24 5.23 5.22 4.29 4.26 4.25 4.23 2.83 2.83 2.80 2.79 2.36 2.27
Cl
Cl
Cl
Cl
Cl
O
Me
Me
68 ^{1}H-NMR
(400 MHz, $CDCl_3$)
2.00 4.02 1.99 1.04 1.05 1.04 3.16 3.10
f1 (ppm)

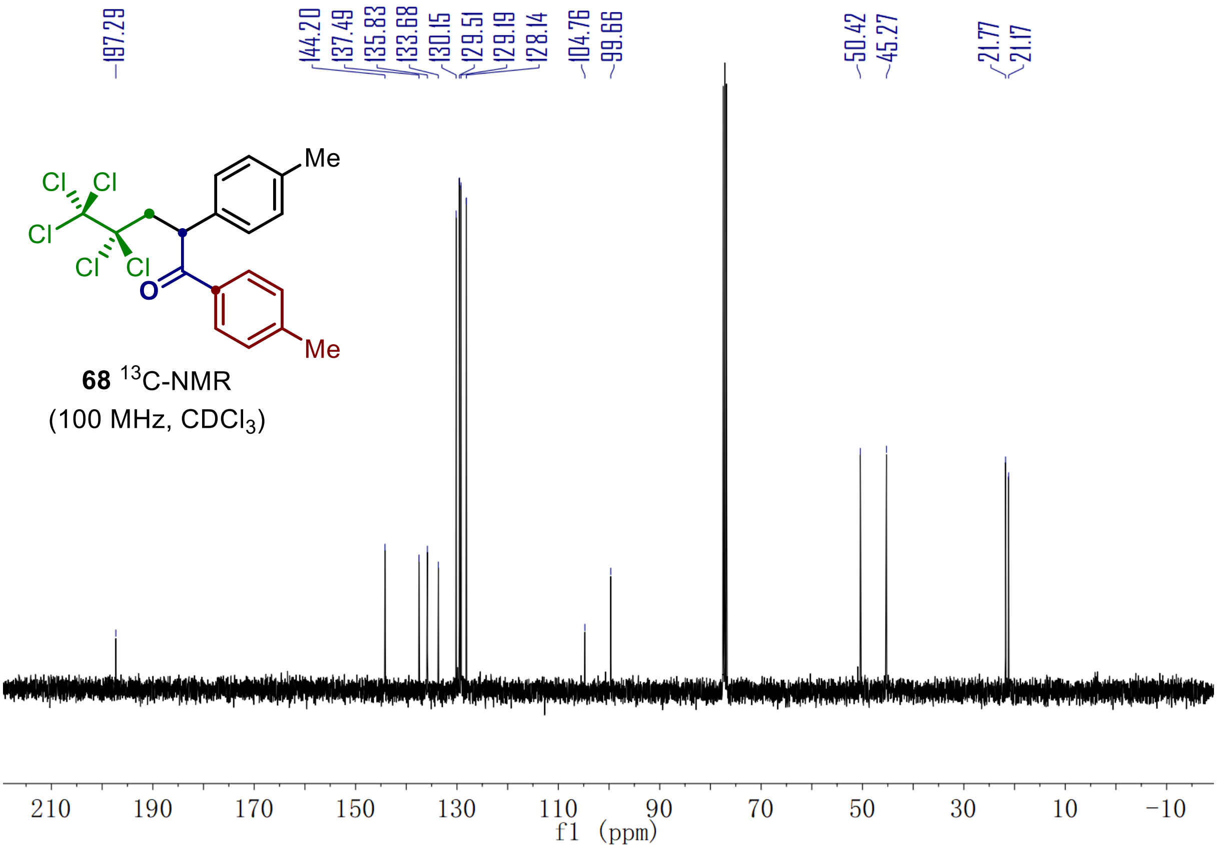

68 ^{13}C-NMR
(100 MHz, $CDCl_3$)

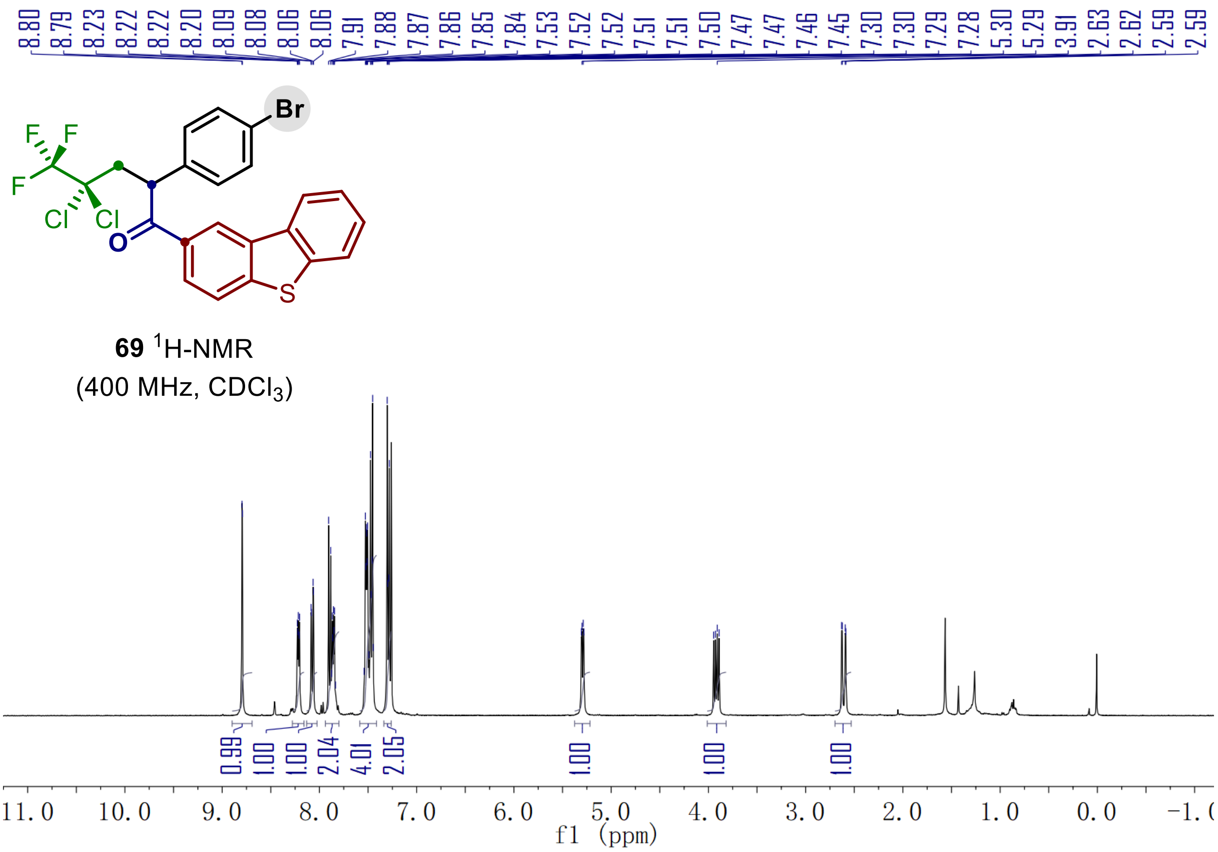

69 ^{1}H-NMR
(400 MHz, $CDCl_3$)

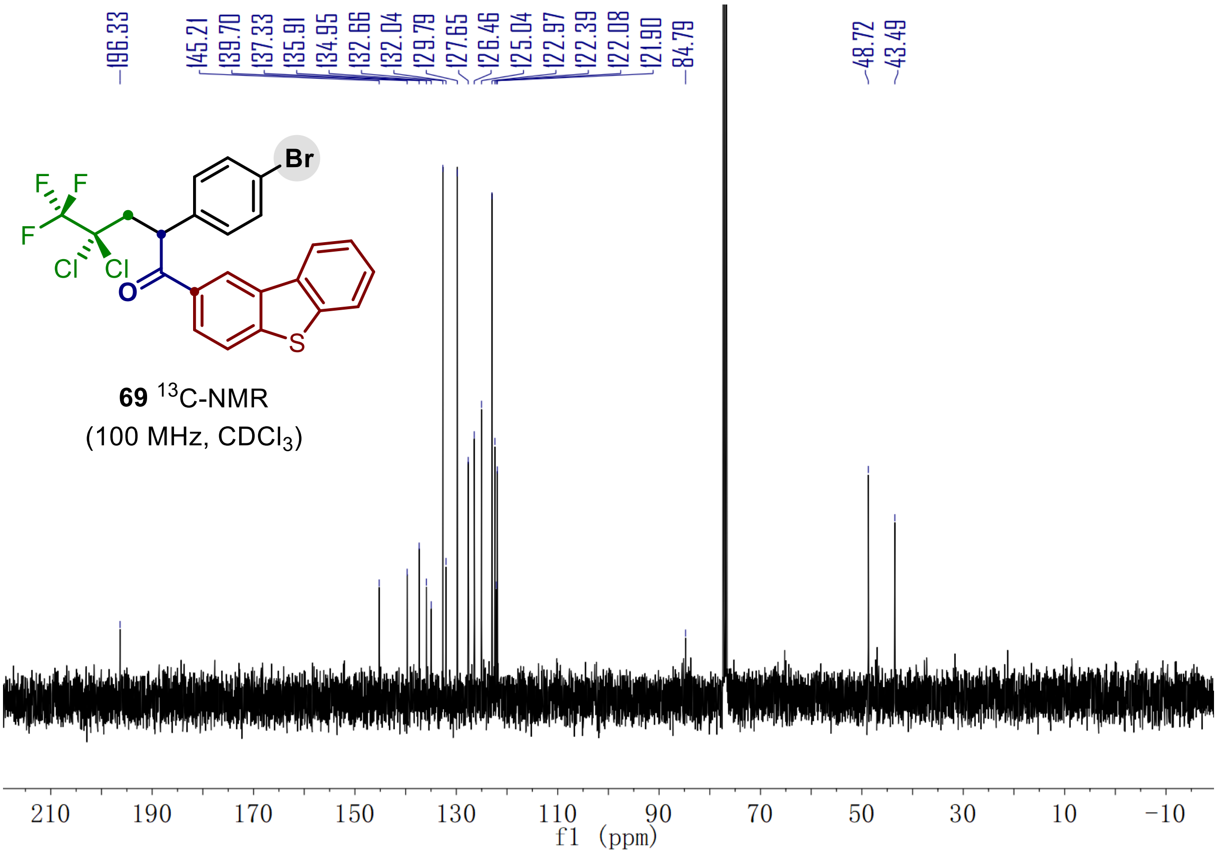

196.33
145.21
139.70
137.33
135.91
134.95
132.66
132.04
129.79
127.65
126.46
125.04
122.97
122.39
122.08
121.90
84.79
48.72
43.49
Br
F
F
F
Cl
Cl
O
S
69 ^{13}C-NMR
(100 MHz, $CDCl_3$)
210
190
170
150
130
110
90
70
50
30
10
−10
f1 (ppm)

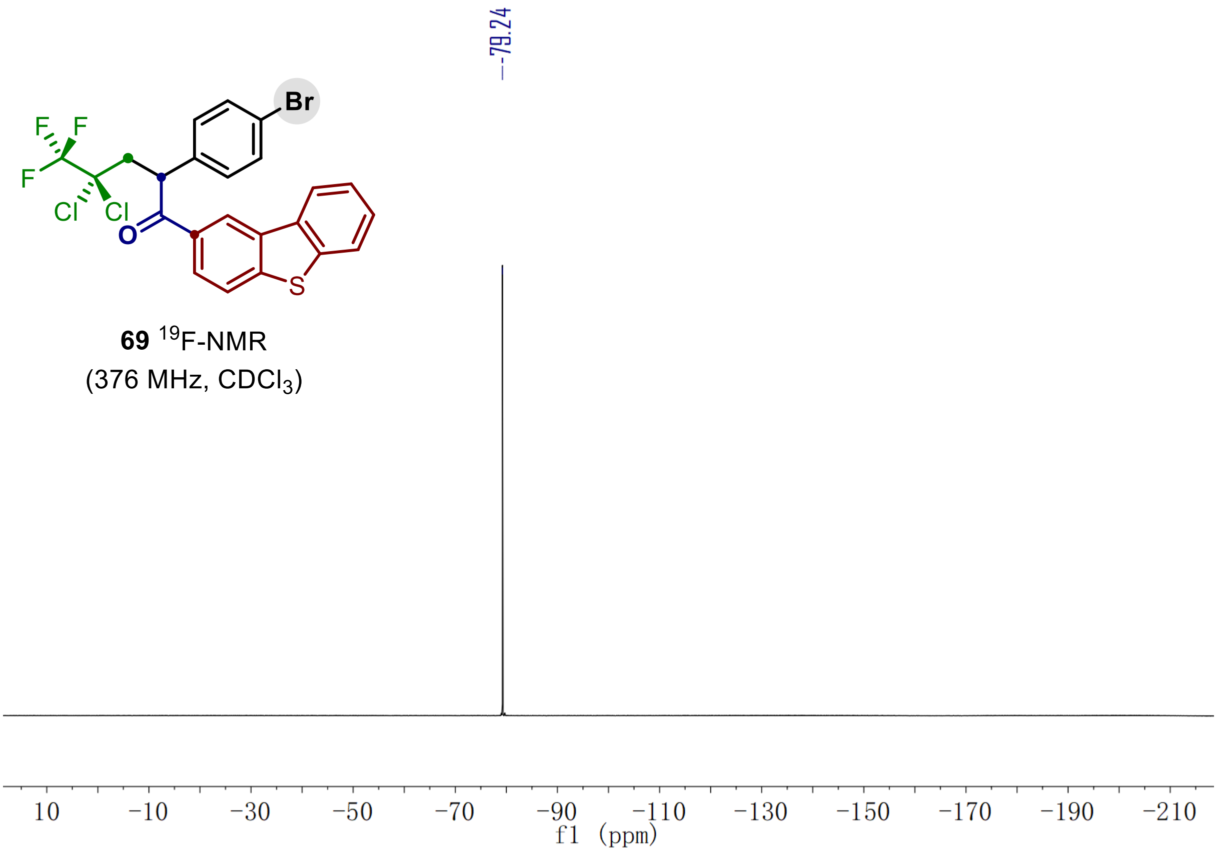

-79.24
Br
F
F
F
Cl
Cl
O
S
69 ^{19}F-NMR
(376 MHz, $CDCl_3$)
10
−10
−30
−50
−70
−90
−110
−130
−150
−170
−190
−210
f1 (ppm)

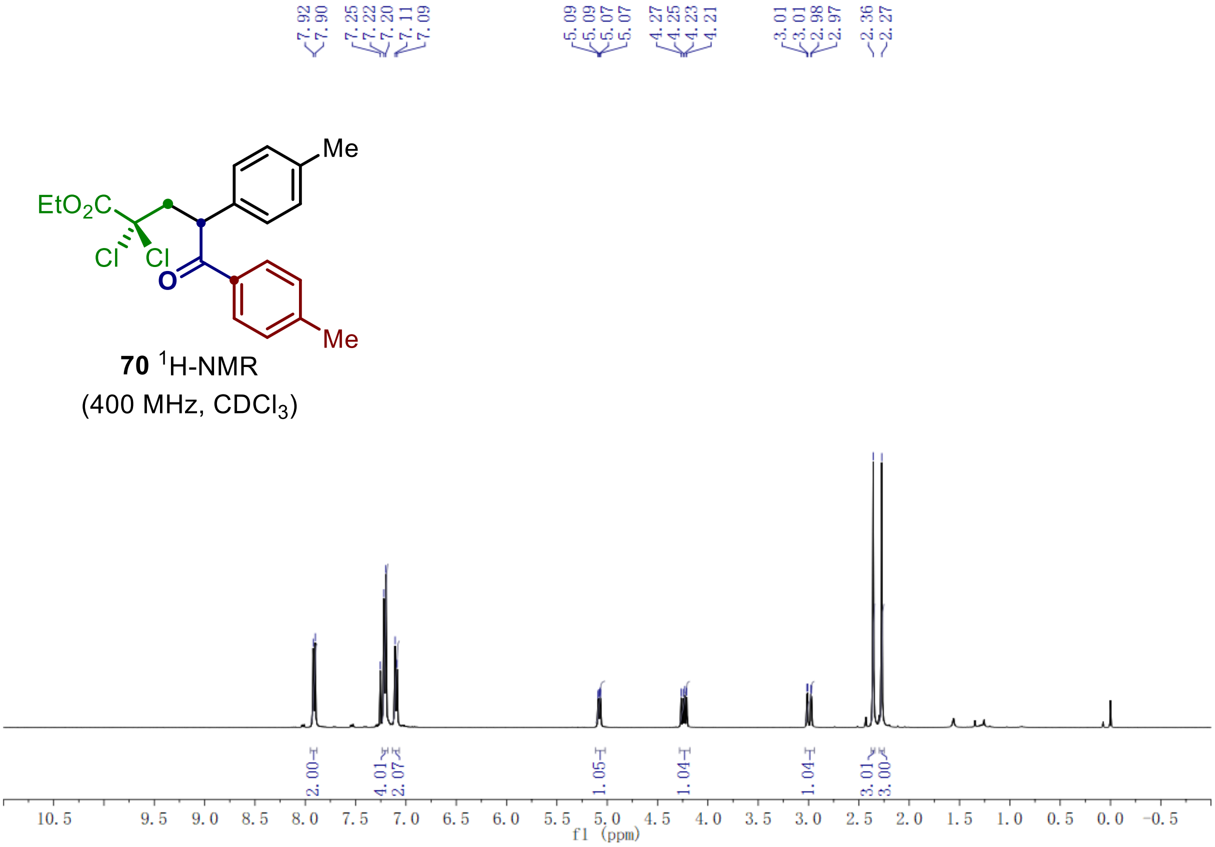
7.92
7.90
7.25
7.22
7.20
7.11
7.09
5.09
5.09
5.07
5.07
4.27
4.25
4.23
4.21
3.01
3.01
2.98
2.97
2.36
2.27
EtO2C
Cl
Cl
O
Me
Me
70 1H-NMR
(400 MHz, CDCl3)
2.00
4.01
2.07
1.05
1.04
1.04
3.01
3.00
f1 (ppm)

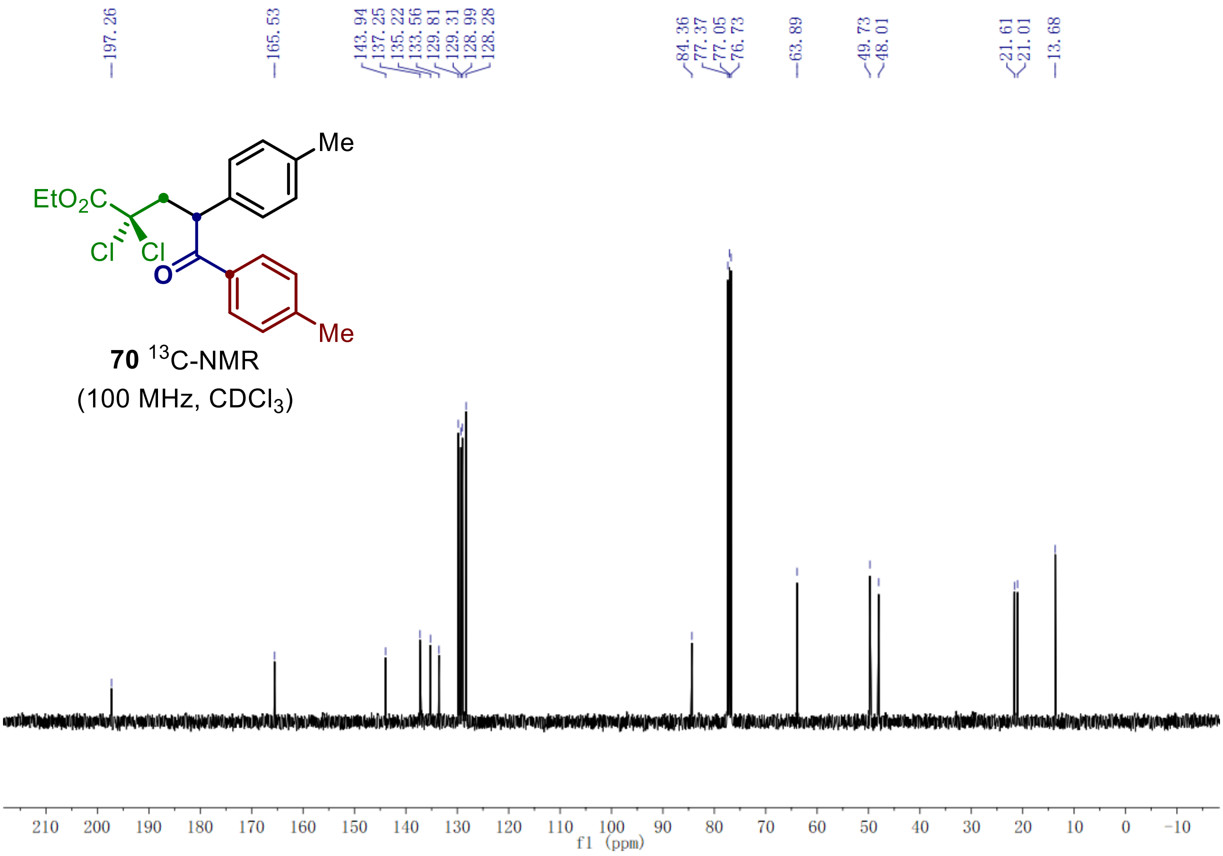
197.26
165.53
143.94
137.25
135.22
133.56
129.81
129.31
128.99
128.28
84.36
77.37
77.05
76.73
63.89
49.73
48.01
21.61
21.01
13.68
EtO2C
Cl
Cl
O
Me
Me
70 13C-NMR
(100 MHz, CDCl3)
f1 (ppm)

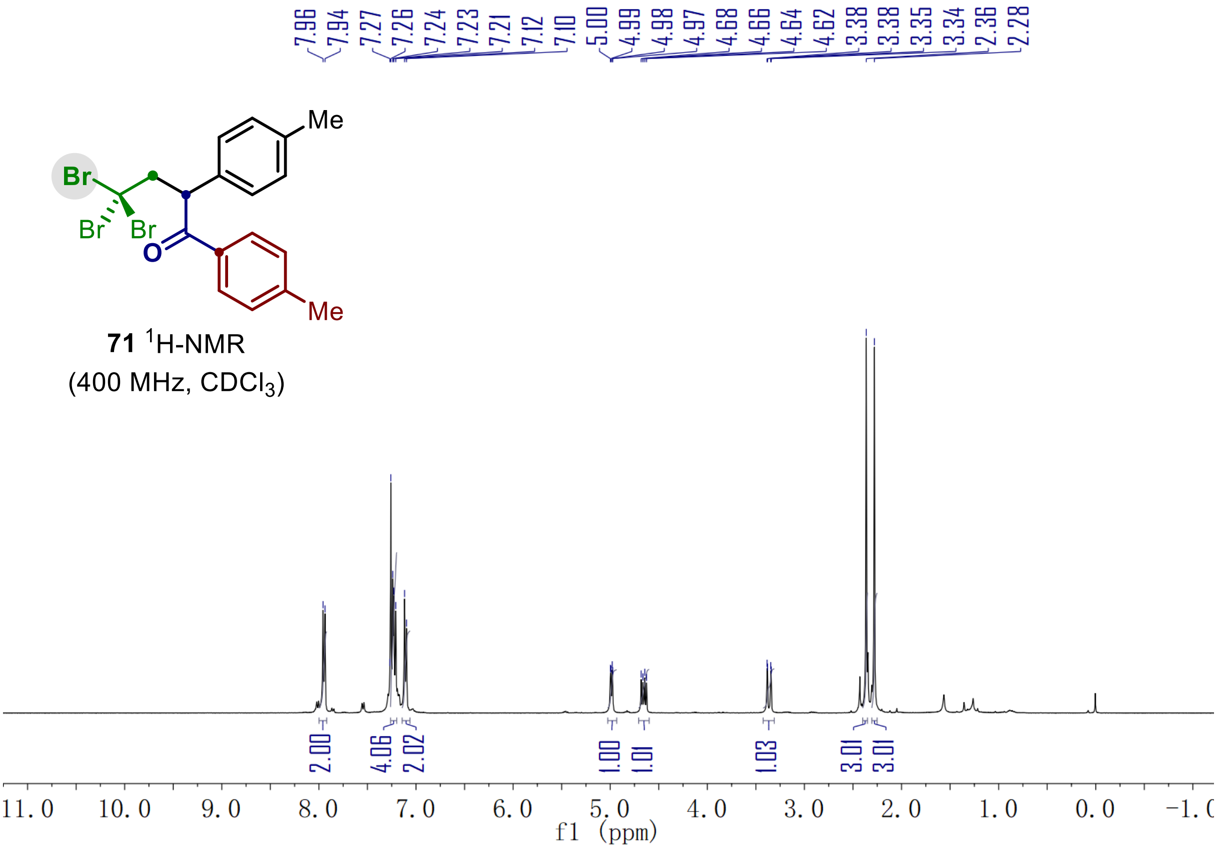
7.96
7.94
7.27
7.26
7.24
7.23
7.21
7.12
7.10
5.00
4.99
4.98
4.97
4.68
4.66
4.64
4.62
3.38
3.38
3.35
3.34
2.36
2.28
Me
Br
Br
Br
O
Me
71 ^{1}H-NMR
(400 MHz, $CDCl_3$)
2.00
4.06
2.02
1.00
1.01
1.03
3.01
3.01
f1 (ppm)

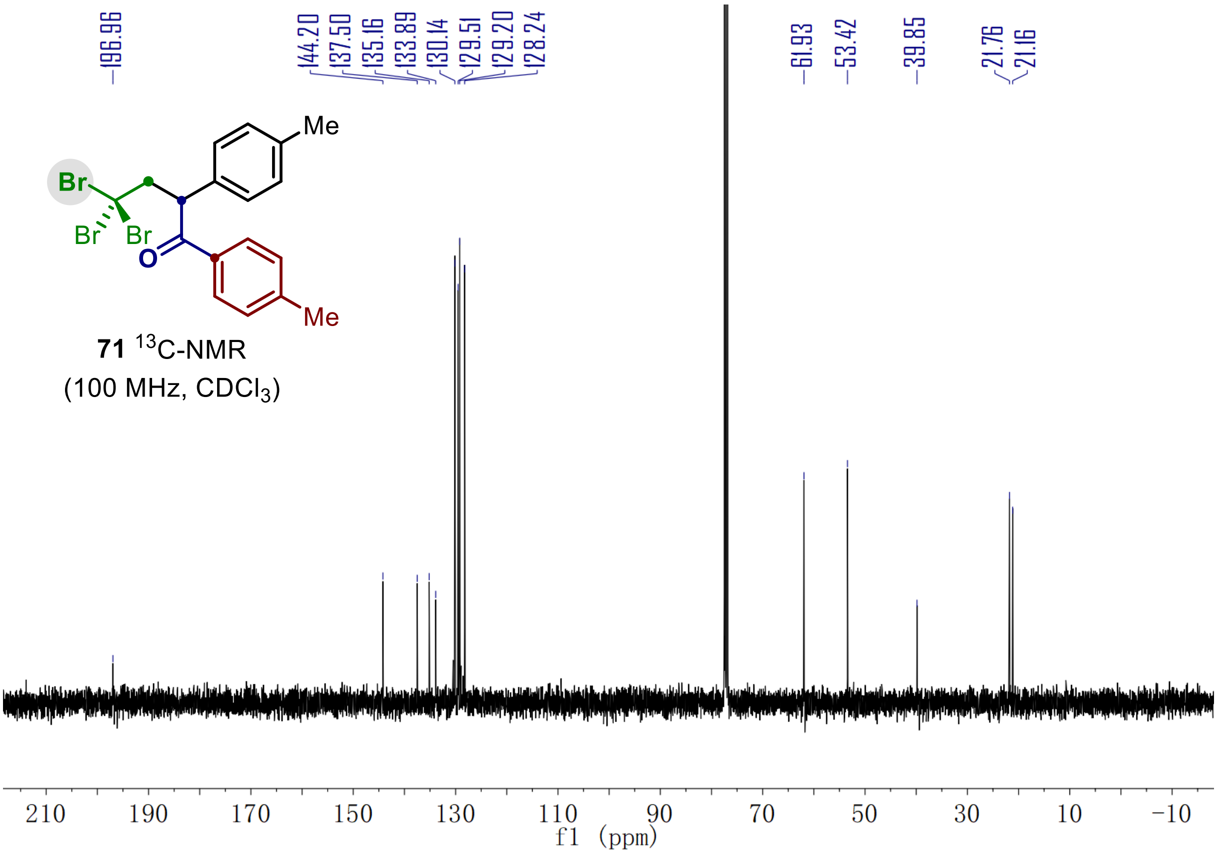
196.96
144.20
137.50
135.16
133.89
130.14
129.51
129.20
128.24
61.93
53.42
39.85
21.76
21.16
Me
Br
Br
Br
O
Me
71 ^{13}C-NMR
(100 MHz, $CDCl_3$)
f1 (ppm)

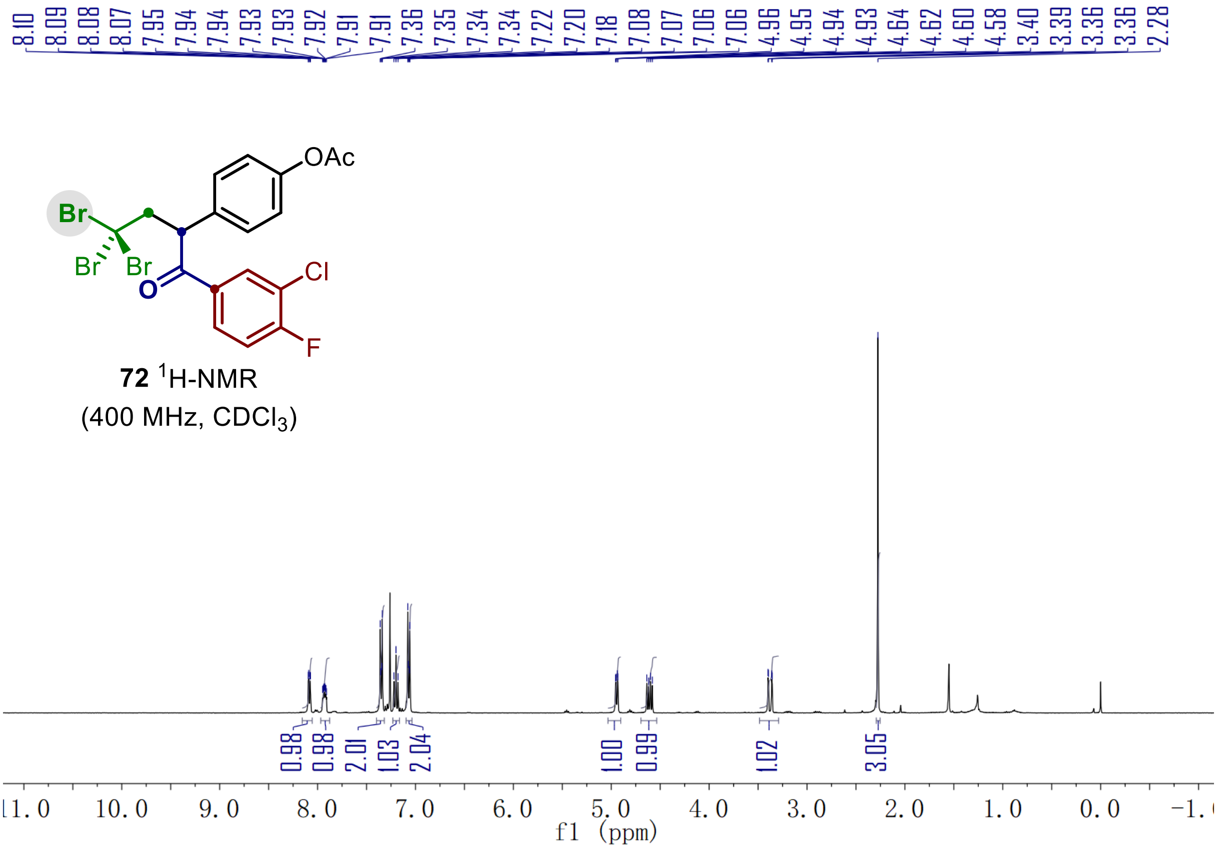
8.10
8.09
8.08
8.07
7.95
7.94
7.94
7.93
7.93
7.92
7.91
7.91
7.36
7.35
7.34
7.34
7.22
7.20
7.18
7.08
7.07
7.06
7.06
4.96
4.95
4.94
4.93
4.64
4.62
4.60
4.58
3.40
3.39
3.36
3.36
2.28
OAc
Br
Br
Br
O
Cl
F
72 1H-NMR
(400 MHz, CDCl3)
0.98
0.98
2.01
1.03
2.04
1.00
0.99
1.02
3.05
11.0
10.0
9.0
8.0
7.0
6.0
5.0
4.0
3.0
2.0
1.0
0.0
f1 (ppm)

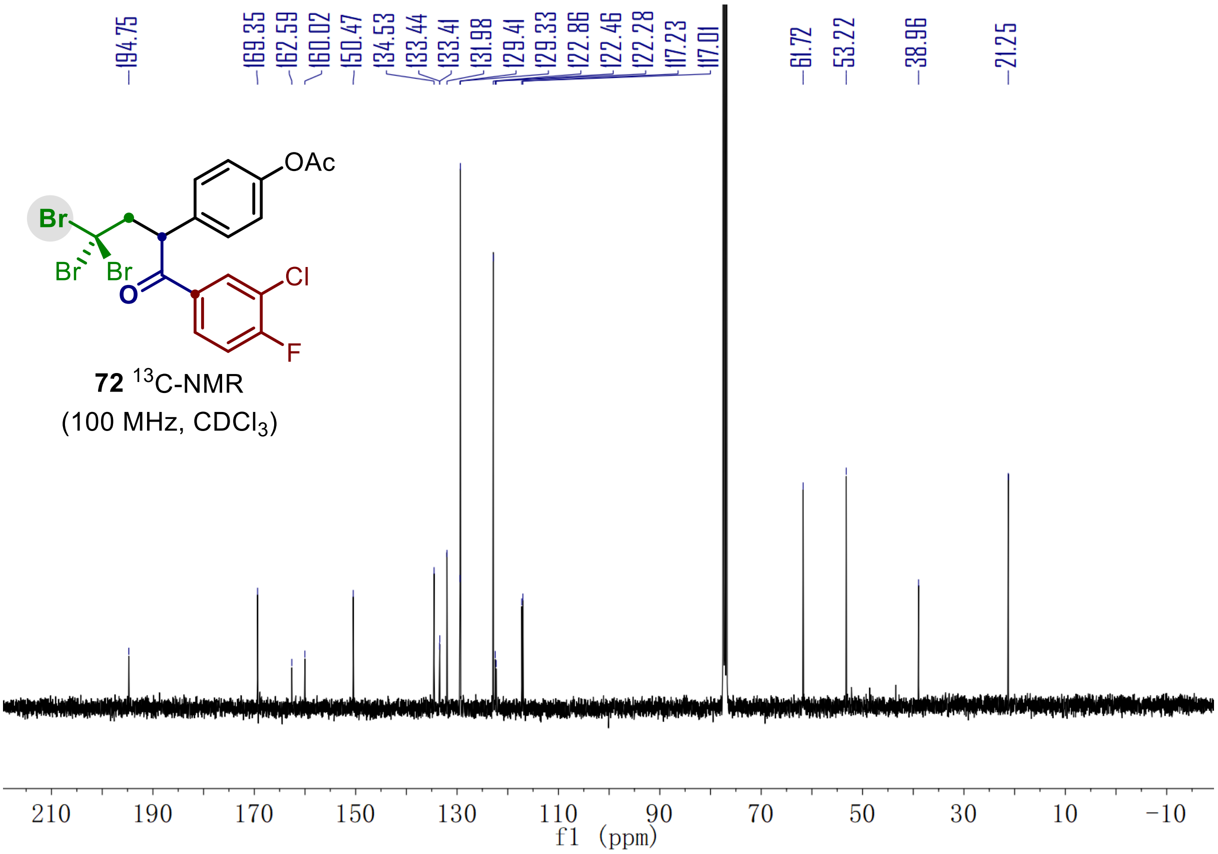
194.75
169.35
162.59
160.02
150.47
134.53
133.44
133.41
131.98
129.41
129.33
122.86
122.46
122.28
117.23
117.01
61.72
53.22
38.96
21.25
OAc
Br
Br
Br
O
Cl
F
72 13C-NMR
(100 MHz, CDCl3)
210
190
170
150
130
110
90
70
50
30
10
-10
f1 (ppm)

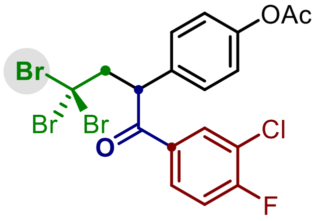

72 ^{19}F-NMR
(376 MHz, $CDCl_3$)

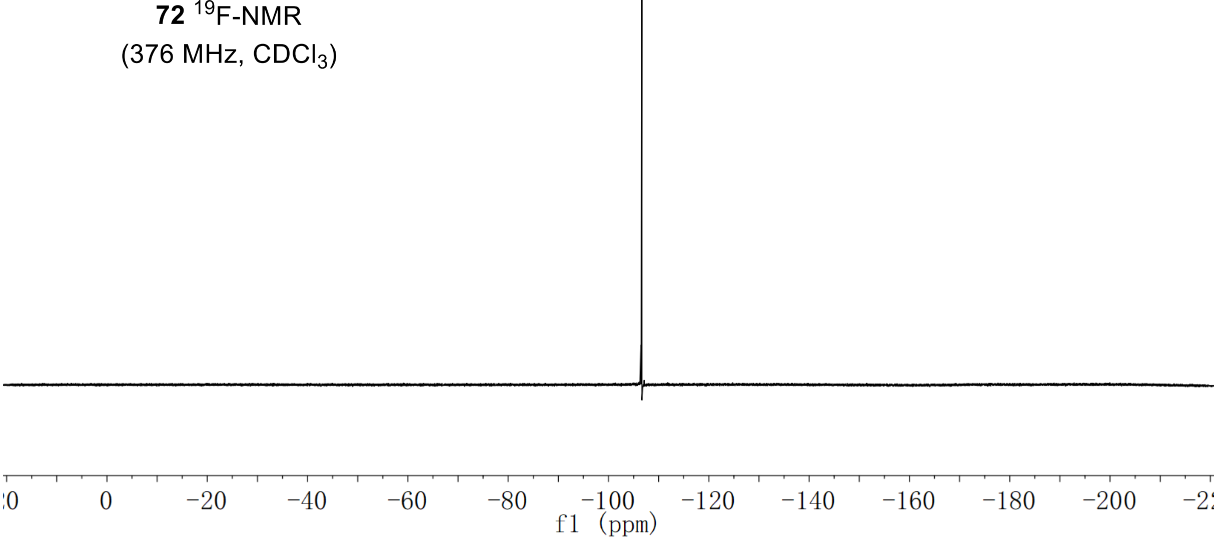

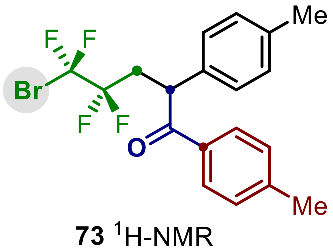

73 ^{1}H-NMR
(400 MHz, $CDCl_3$)

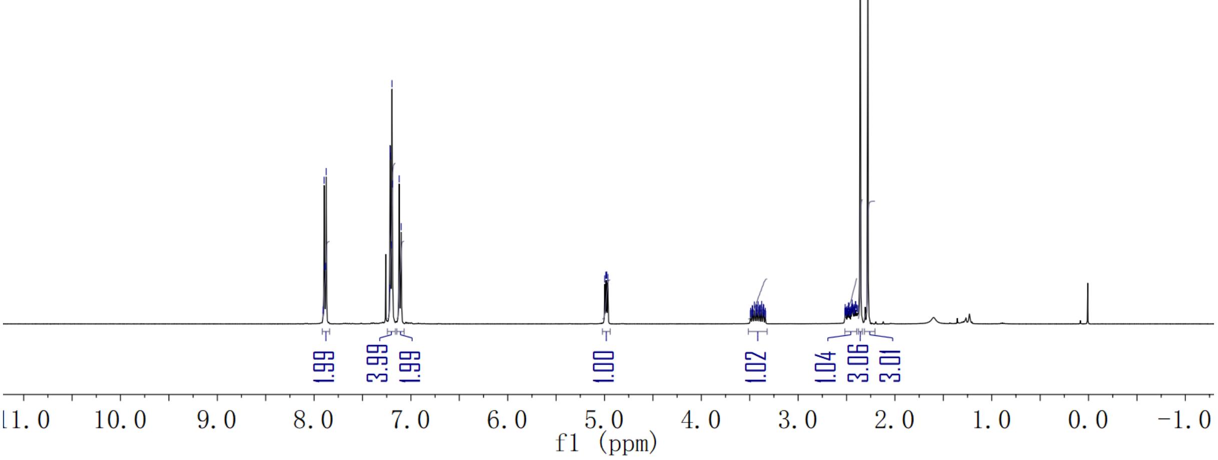

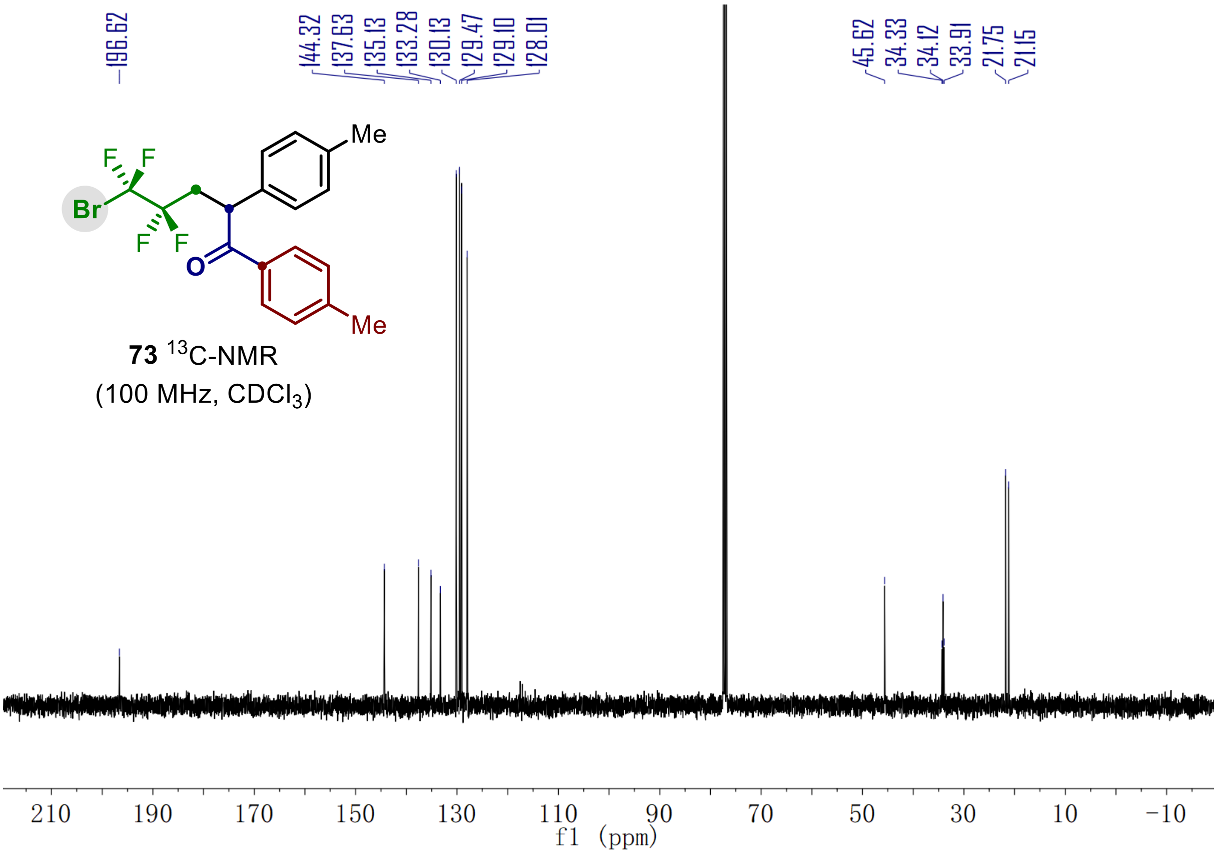

196.62
144.32
137.63
135.13
133.28
130.13
129.47
129.10
128.01
45.62
34.33
34.12
33.91
21.75
21.15
Br
F
F
F
F
O
Me
Me
73 13C-NMR
(100 MHz, CDCl3)
210
190
170
150
130
110
90
70
50
30
10
−10
f1 (ppm)

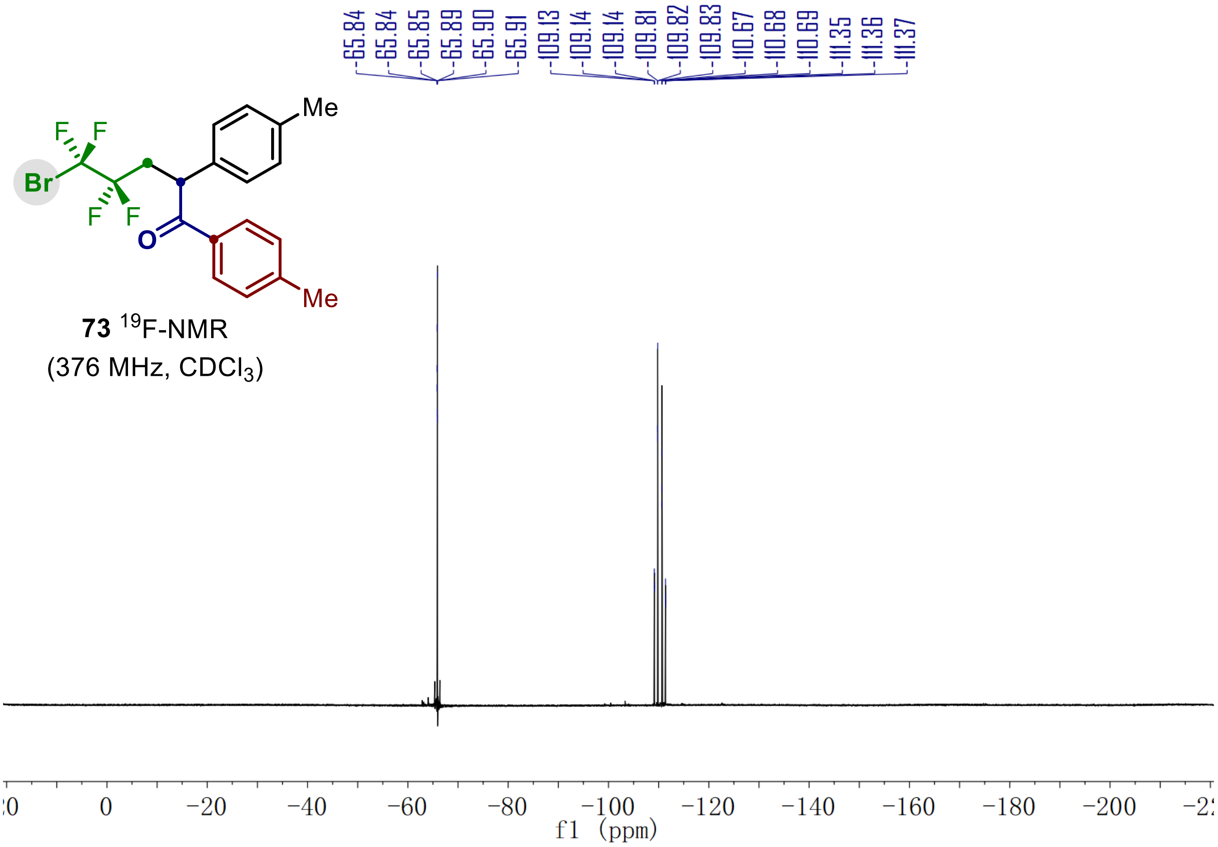

-65.84
-65.84
-65.85
-65.89
-65.90
-65.91
-109.13
-109.14
-109.14
-109.81
-109.82
-109.83
-110.67
-110.68
-110.69
-111.35
-111.36
-111.37
Br
F
F
F
F
O
Me
Me
73 19F-NMR
(376 MHz, CDCl3)
0
−20
−40
−60
−80
−100
−120
−140
−160
−180
−200
f1 (ppm)

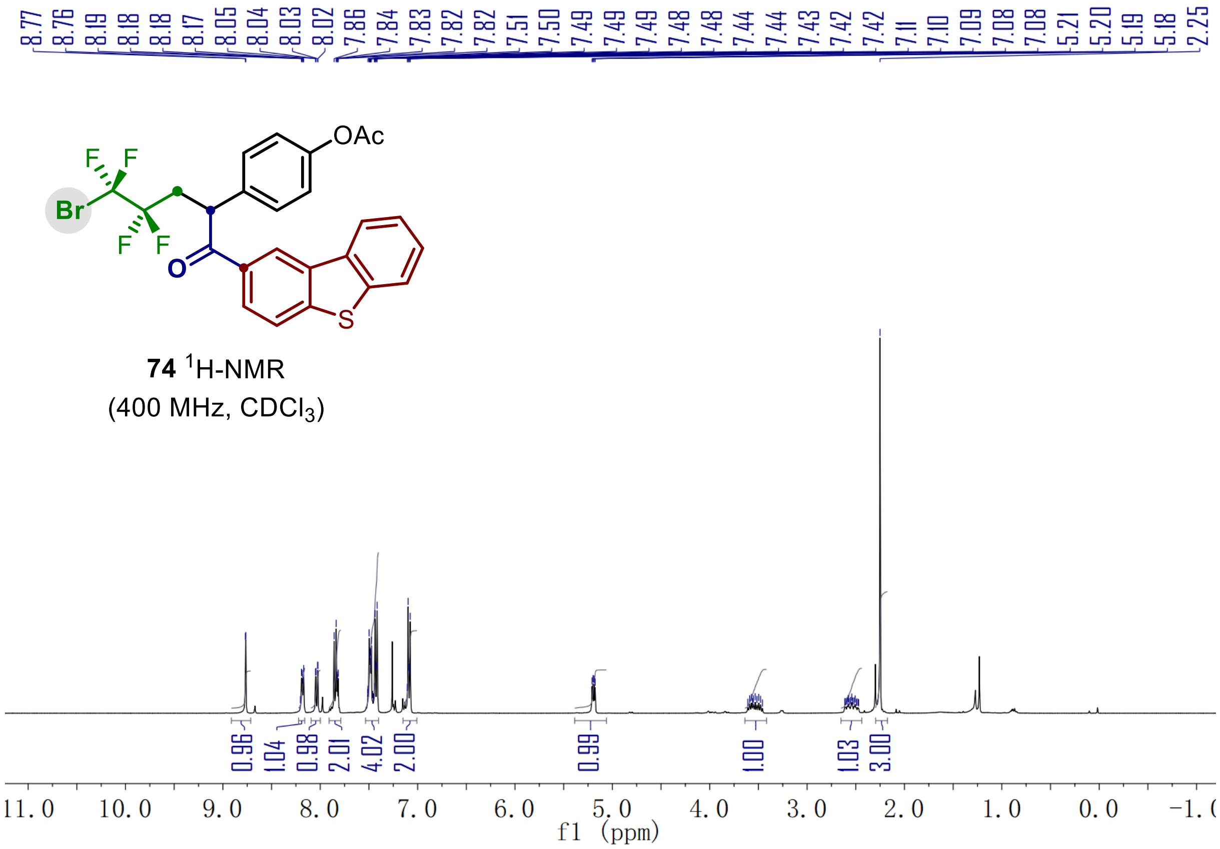

74 ^{1}H-NMR
(400 MHz, $CDCl_3$)

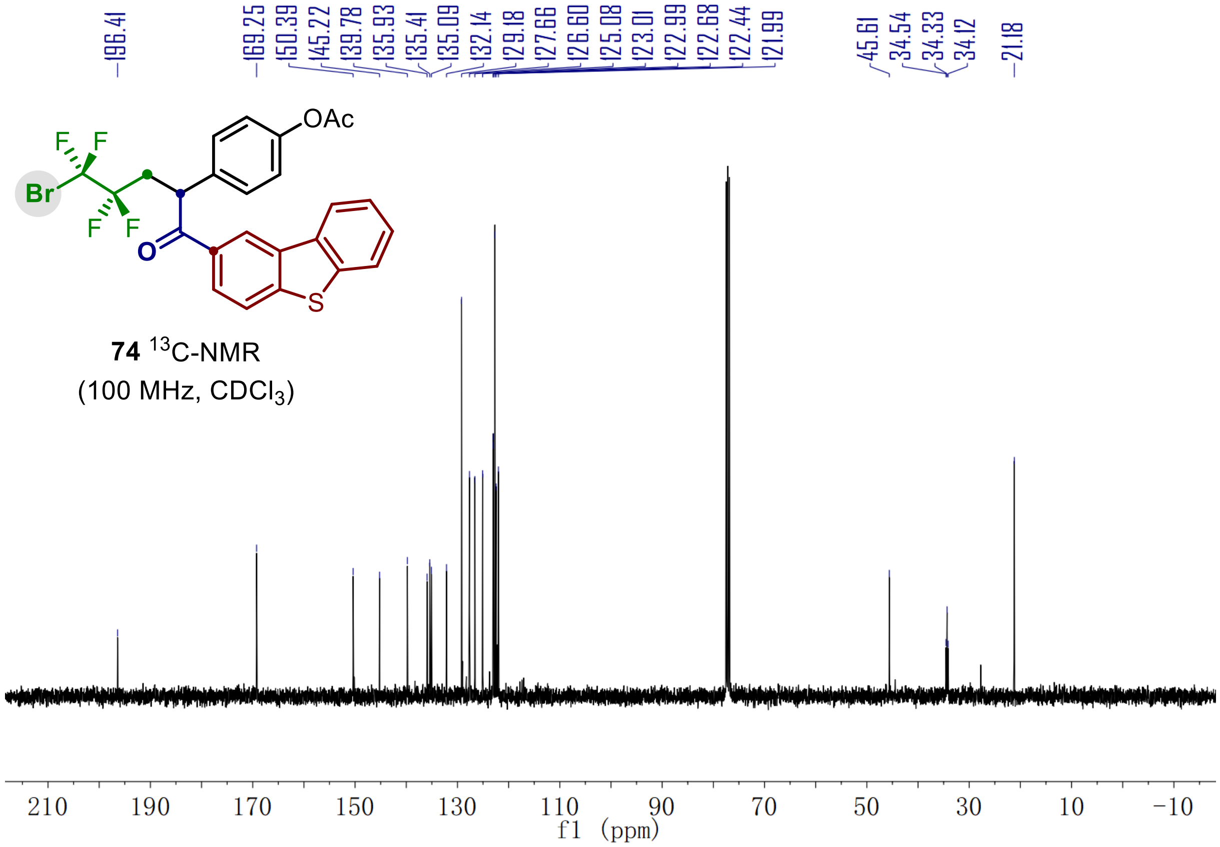

74 ^{13}C-NMR
(100 MHz, $CDCl_3$)

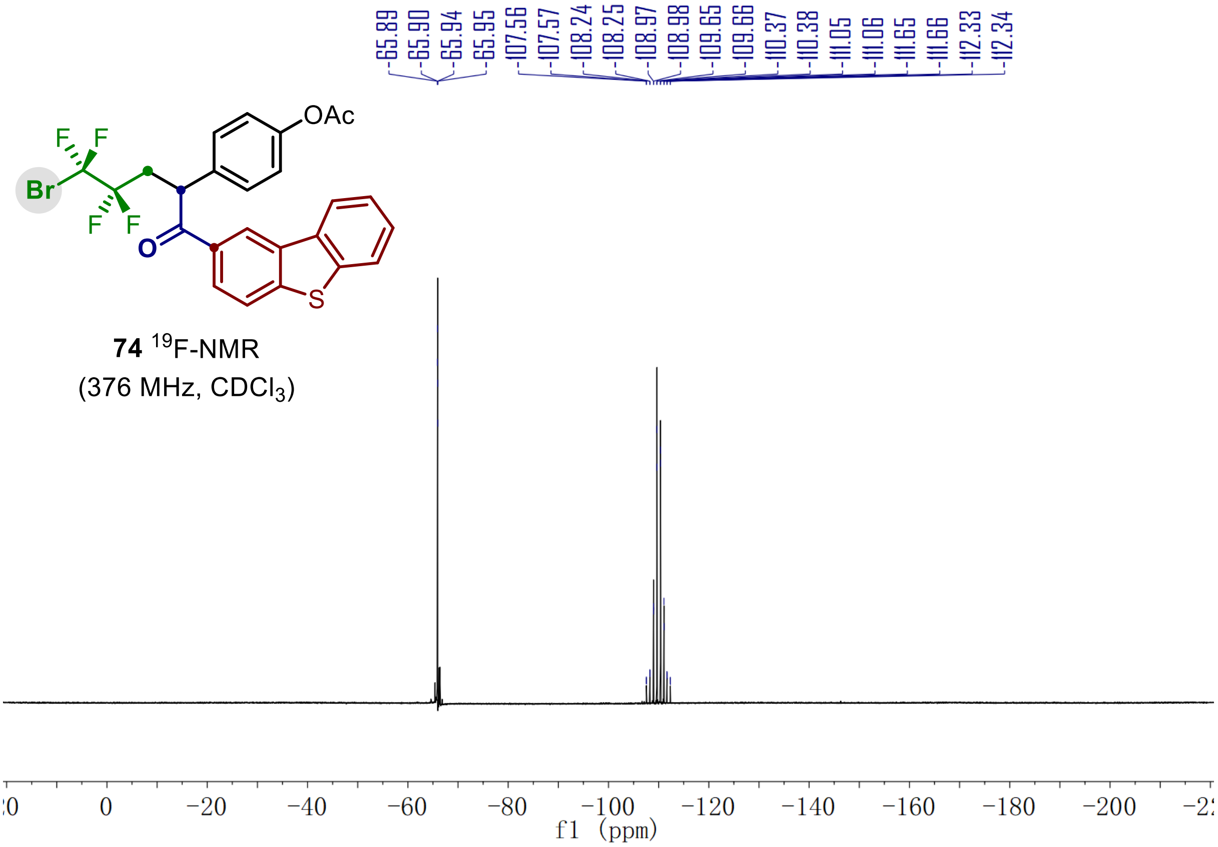
-65.89
-65.90
-65.94
-65.95
-107.56
-107.57
-108.24
-108.25
-108.97
-108.98
-109.65
-109.66
-110.37
-110.38
-111.05
-111.06
-111.65
-111.66
-112.33
-112.34
OAc
F F
Br
F F
O
S
74 ^{19}F-NMR
(376 MHz, $CDCl_3$)
0 −20 −40 −60 −80 −100 −120 −140 −160 −180 −200
f1 (ppm)

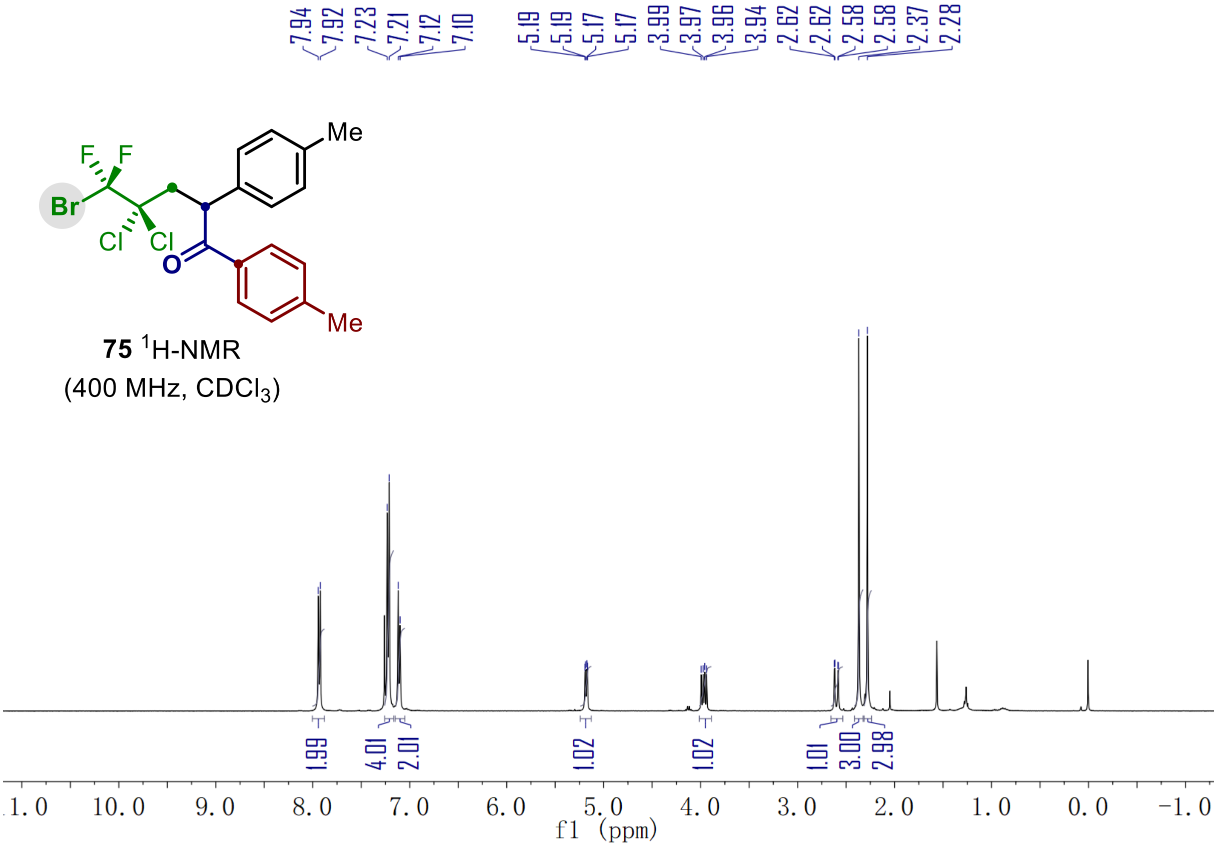
7.94
7.92
7.23
7.21
7.12
7.10
5.19
5.19
5.17
5.17
3.99
3.97
3.96
3.94
2.62
2.62
2.58
2.58
2.37
2.28
Me
F F
Br
Cl Cl
O
Me
75 ^{1}H-NMR
(400 MHz, $CDCl_3$)
1.99
4.01
2.01
1.02
1.02
1.01
3.00
2.98
10.0 9.0 8.0 7.0 6.0 5.0 4.0 3.0 2.0 1.0 0.0 -1.0
f1 (ppm)

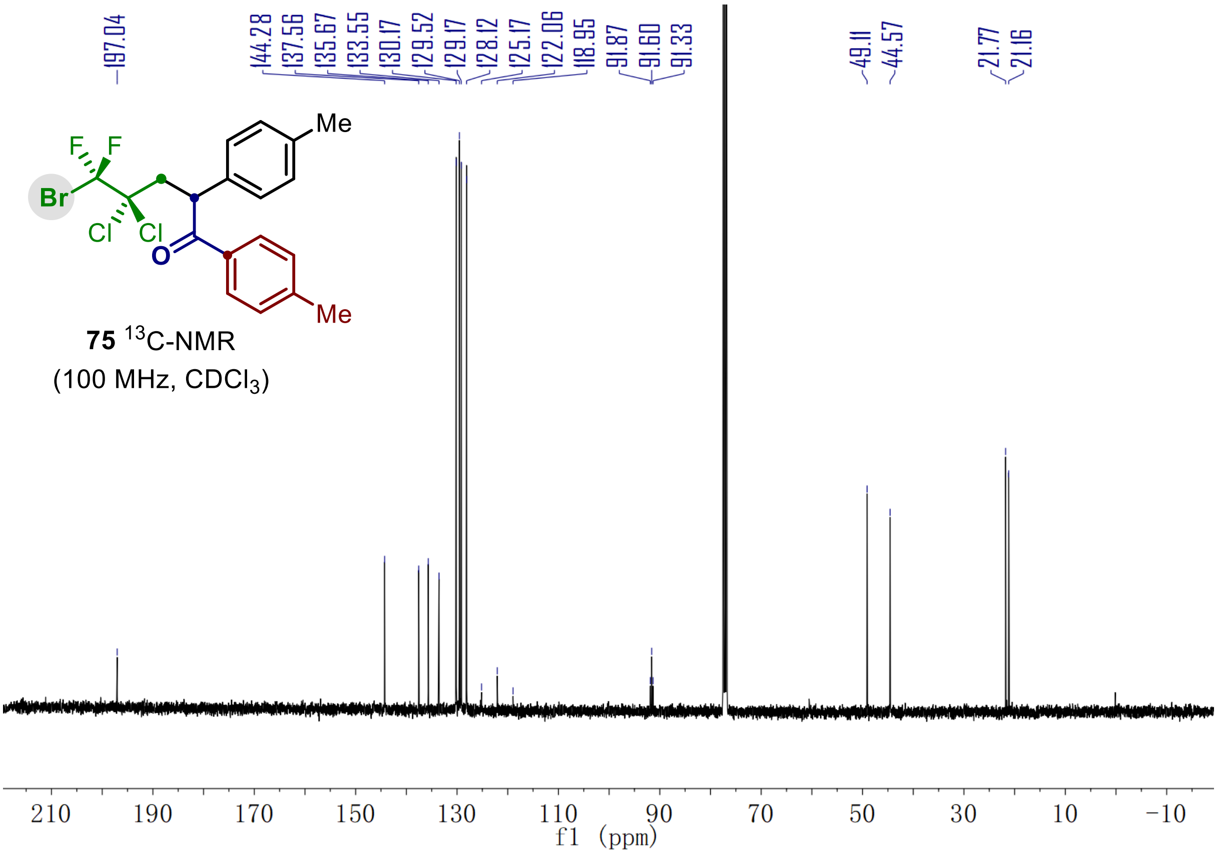

197.04
144.28
137.56
135.67
133.55
130.17
129.52
129.17
128.12
125.17
122.06
118.95
91.87
91.60
91.33
49.11
44.57
21.77
21.16
Br
F
F
Cl
Cl
O
Me
Me
75 13C-NMR
(100 MHz, CDCl3)
210 190 170 150 130 110 90 70 50 30 10 −10
f1 (ppm)

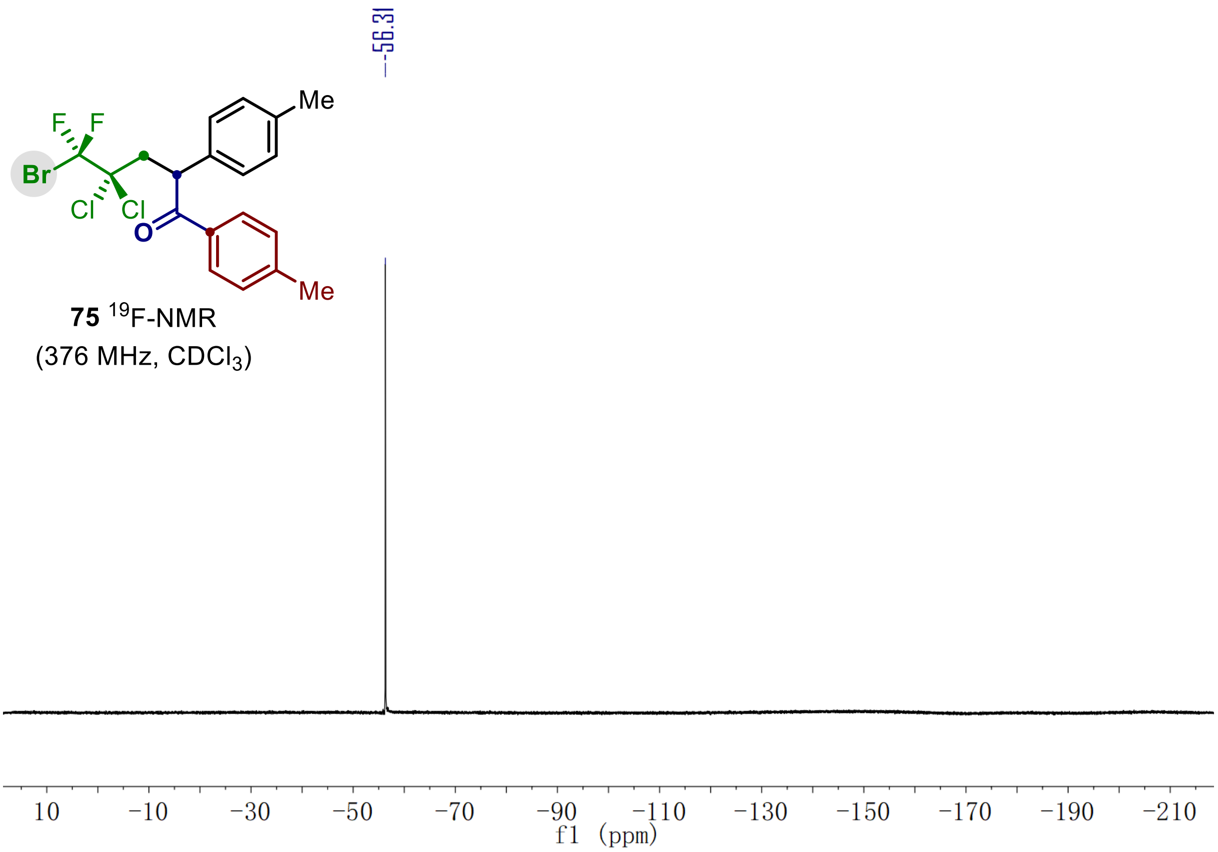

−56.31
Br
F
F
Cl
Cl
O
Me
Me
75 19F-NMR
(376 MHz, CDCl3)
10 −10 −30 −50 −70 −90 −110 −130 −150 −170 −190 −210
f1 (ppm)

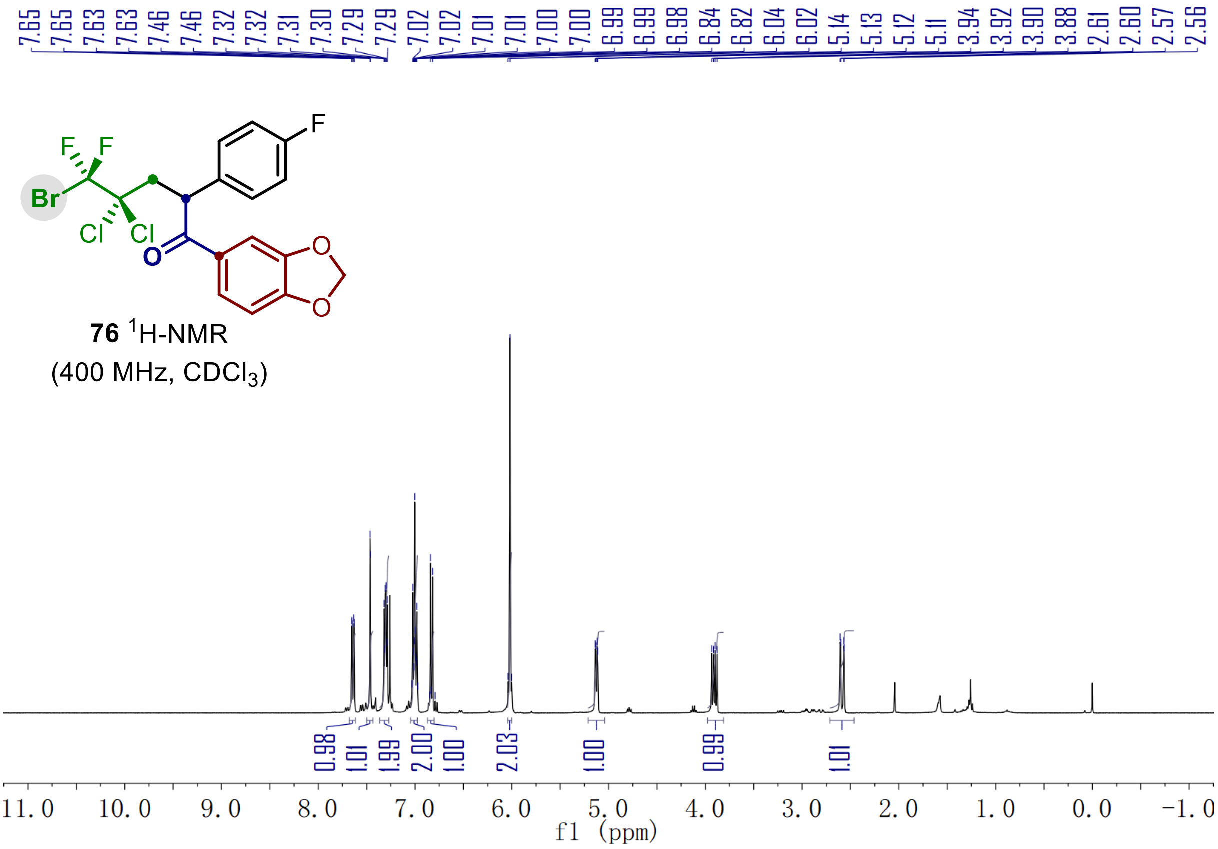
7.65 7.65 7.63 7.63 7.46 7.46 7.32 7.32 7.31 7.30 7.29 7.29 7.02 7.02 7.01 7.01 7.00 7.00 6.99 6.99 6.98 6.84 6.82 6.04 6.02 5.14 5.13 5.12 5.11 3.94 3.92 3.90 3.88 2.61 2.60 2.57 2.56
F
F
F
Br
Cl
Cl
O
O
O
76 1H-NMR
(400 MHz, CDCl3)
0.98 1.01 1.99 2.00 1.00 2.03 1.00 0.99 1.01
11.0 10.0 9.0 8.0 7.0 6.0 5.0 4.0 3.0 2.0 1.0 0.0 -1.0
f1 (ppm)

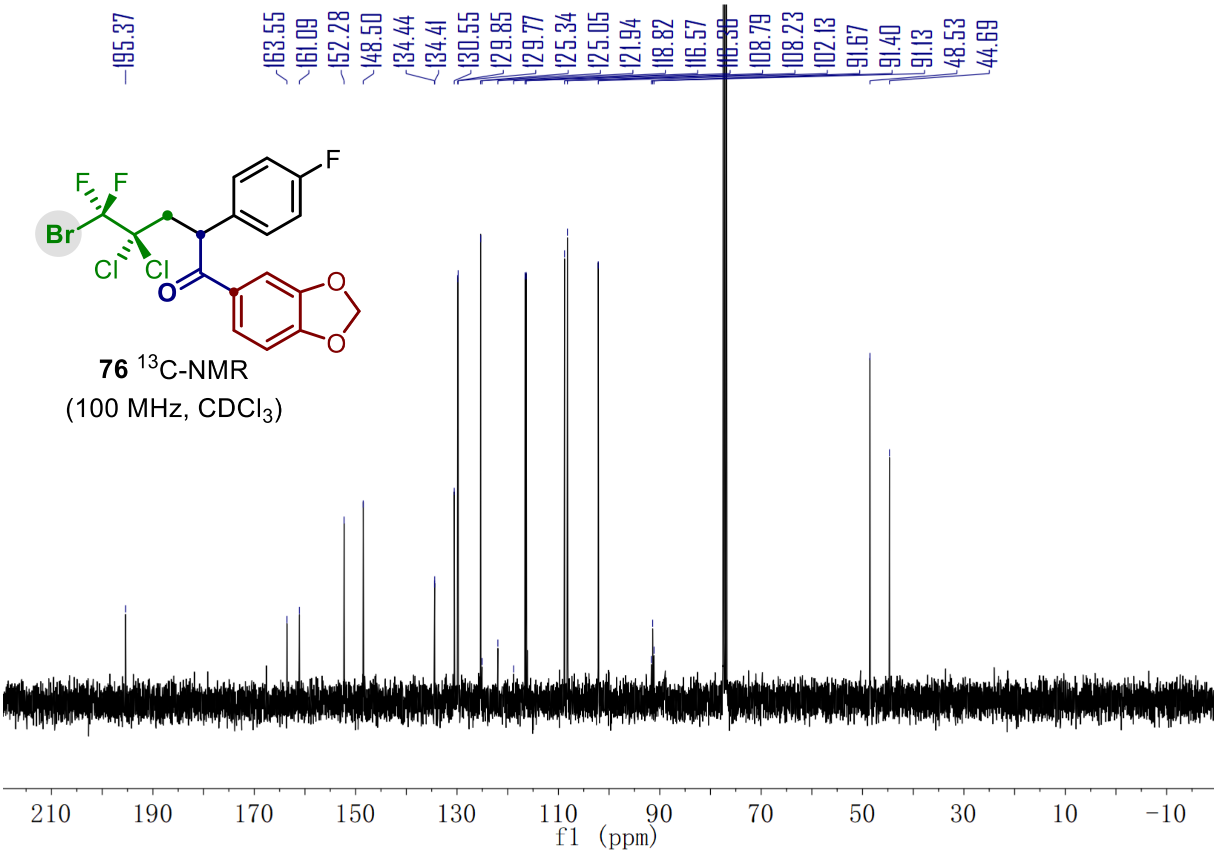
195.37
163.55 161.09 152.28 148.50 134.44 134.41 130.55 129.85 129.77 125.34 125.05 121.94 118.82 116.57 108.79 108.23 102.13 91.67 91.40 91.13 48.53 44.69
F
F
F
Br
Cl
Cl
O
O
O
76 13C-NMR
(100 MHz, CDCl3)
210 190 170 150 130 110 90 70 50 30 10 -10
f1 (ppm)

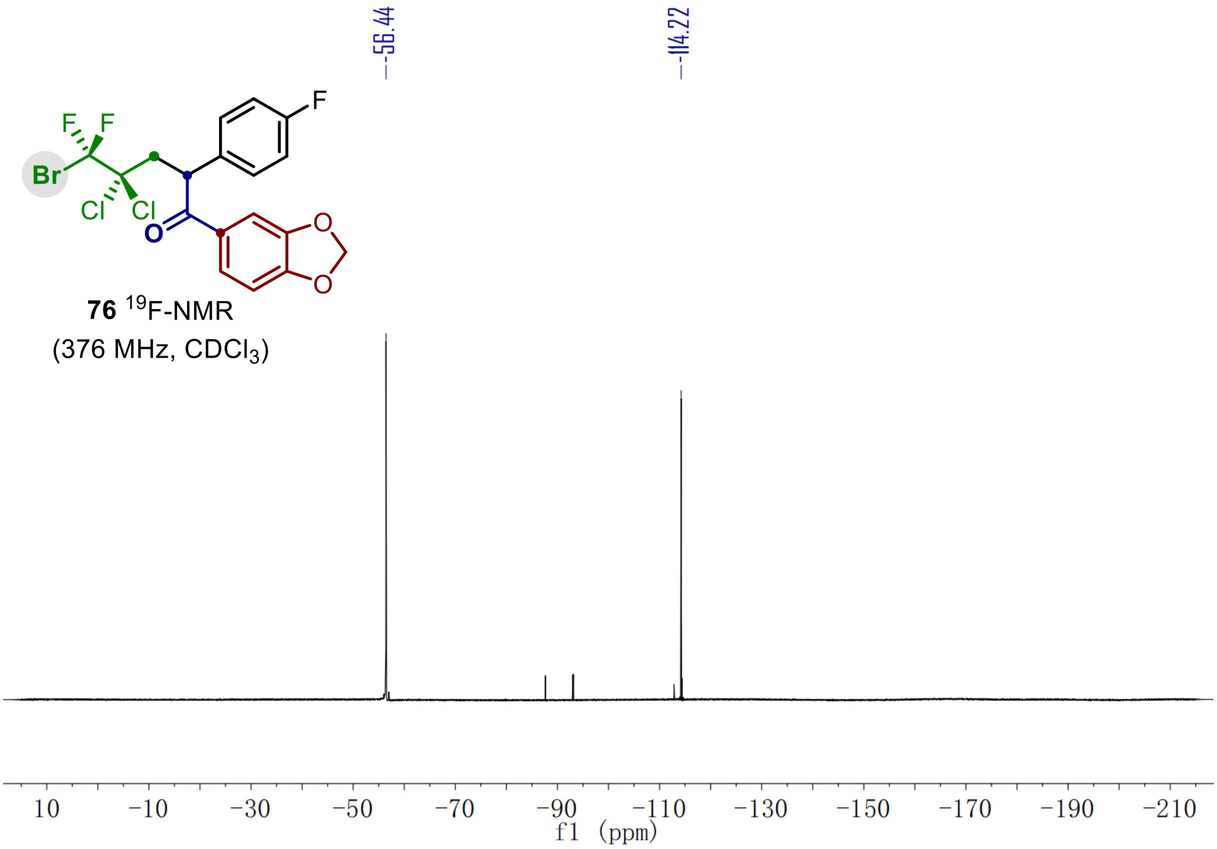

76 ^{19}F-NMR (376 MHz, $CDCl_3$)

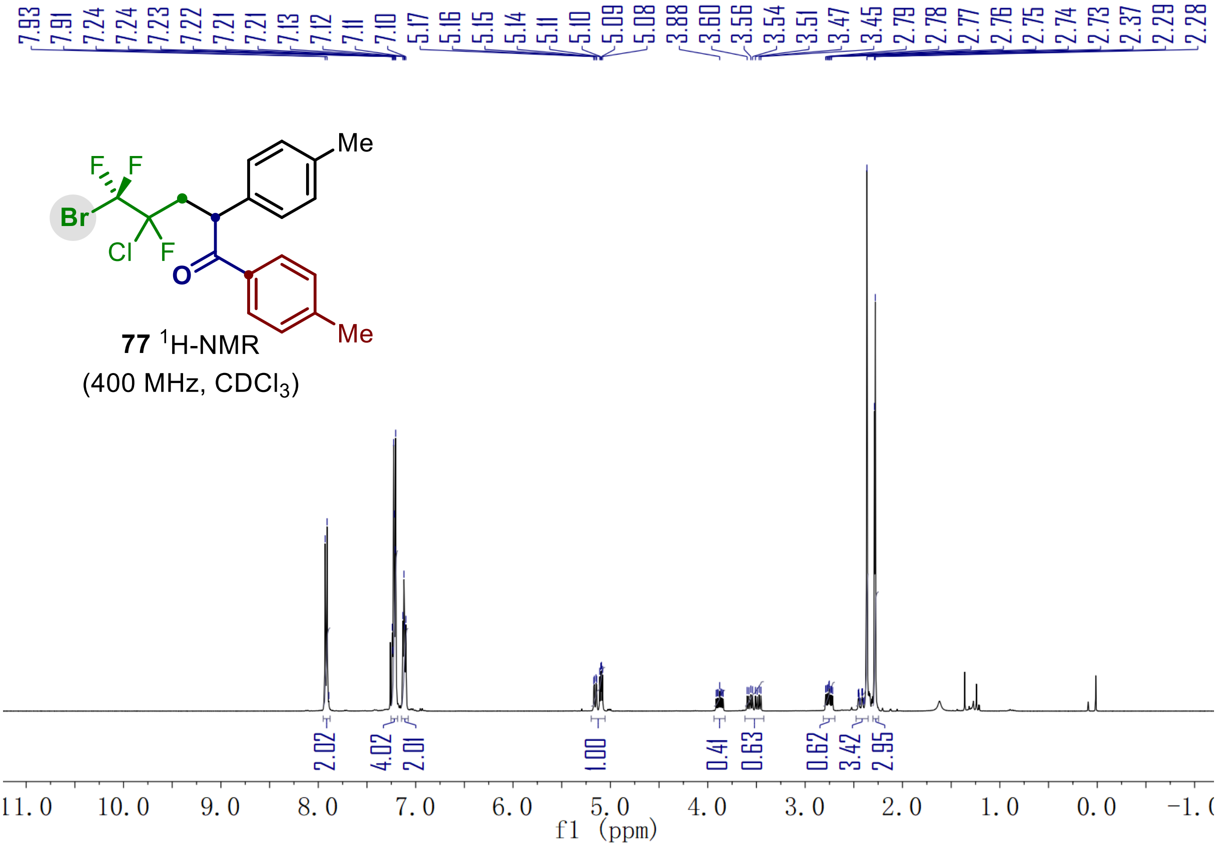

77 ^{1}H-NMR (400 MHz, $CDCl_3$)

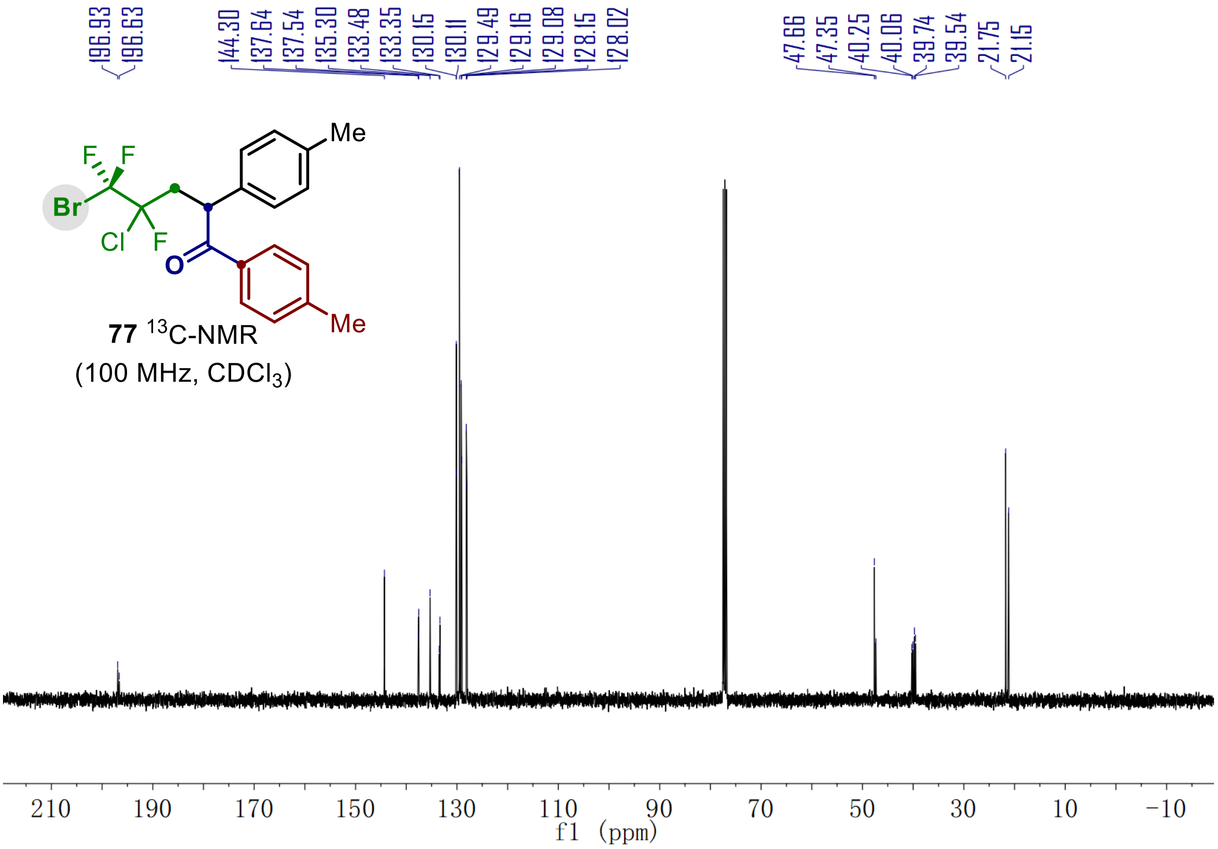
196.93
196.63
144.30
137.64
137.54
135.30
133.48
133.35
130.15
130.11
129.49
129.16
129.08
128.15
128.02
47.66
47.35
40.25
40.06
39.74
39.54
21.75
21.15
Me
F
F
Br
Cl
F
O
Me
77 13C-NMR
(100 MHz, CDCl3)
210
190
170
150
130
110
90
70
50
30
10
−10
f1 (ppm)

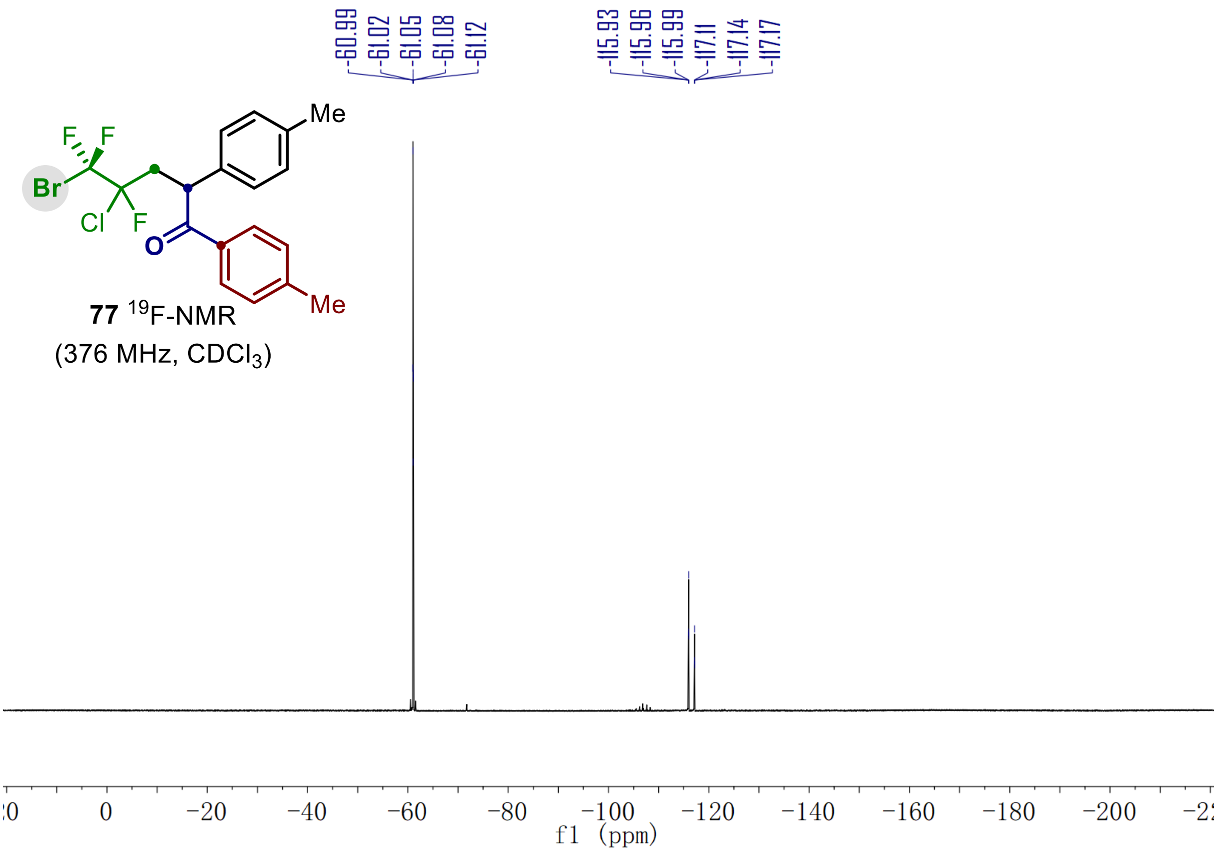
-60.99
-61.02
-61.05
-61.08
-61.12
-115.93
-115.96
-115.99
-117.11
-117.14
-117.17
Me
F
F
Br
Cl
F
O
Me
77 19F-NMR
(376 MHz, CDCl3)
0
−20
−40
−60
−80
−100
−120
−140
−160
−180
−200
f1 (ppm)

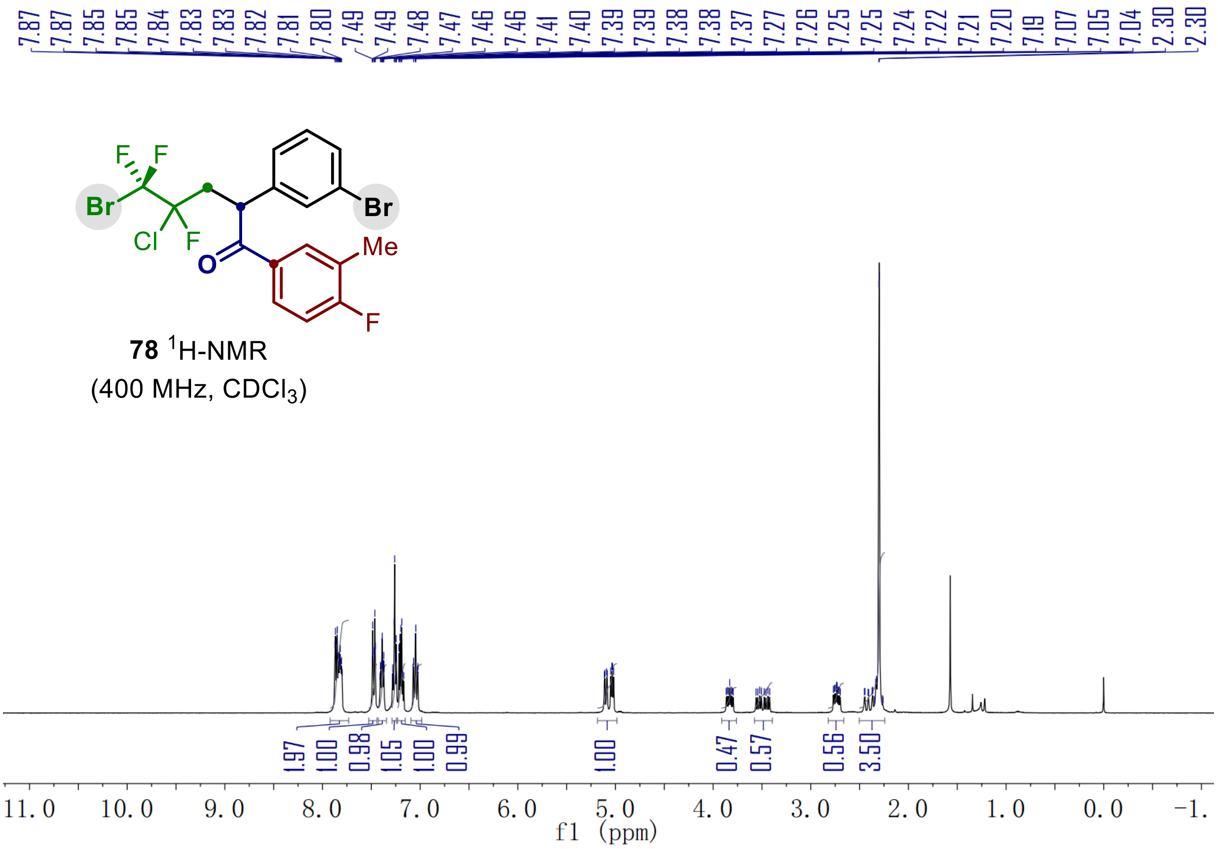
7.87 7.87 7.85 7.85 7.84 7.83 7.83 7.82 7.81 7.80 7.49 7.49 7.48 7.47 7.46 7.46 7.41 7.40 7.39 7.39 7.38 7.38 7.37 7.27 7.26 7.25 7.25 7.24 7.22 7.21 7.20 7.19 7.07 7.05 7.04 2.30 2.30
F F Br Cl F Br O Me F
78 1H-NMR
(400 MHz, CDCl3)
1.97 1.00 0.98 1.05 1.00 0.99 1.00 0.47 0.57 0.56 3.50
11.0 10.0 9.0 8.0 7.0 6.0 5.0 4.0 3.0 2.0 1.0 0.0 -1.
f1 (ppm)

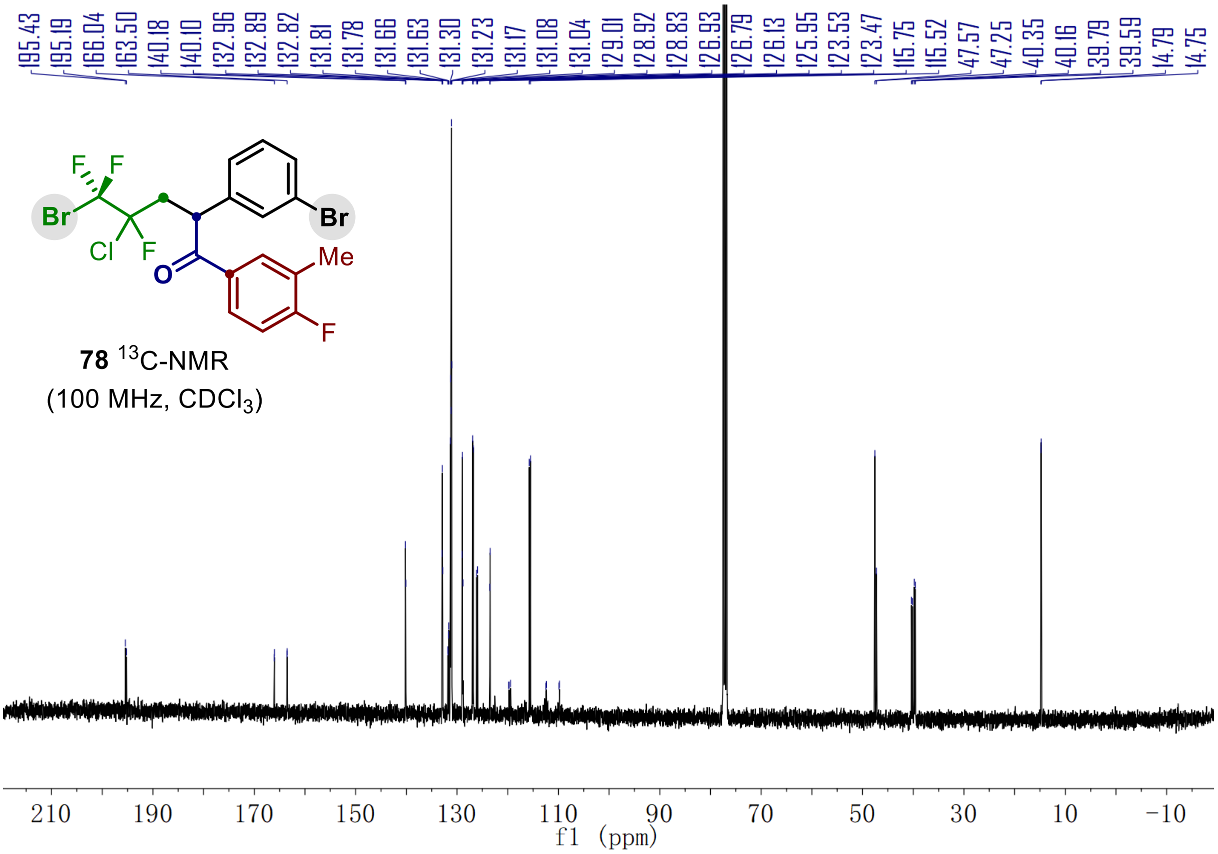
195.43 195.19 166.04 163.50 140.18 140.10 132.96 132.89 132.82 131.81 131.78 131.66 131.63 131.30 131.23 131.17 131.08 131.04 129.01 128.92 128.83 126.93 126.79 126.13 125.95 123.53 123.47 115.75 115.52 47.57 47.25 40.35 40.16 39.79 39.59 14.79 14.75
F F Br Cl F Br O Me F
78 13C-NMR
(100 MHz, CDCl3)
210 190 170 150 130 110 90 70 50 30 10 -10
f1 (ppm)

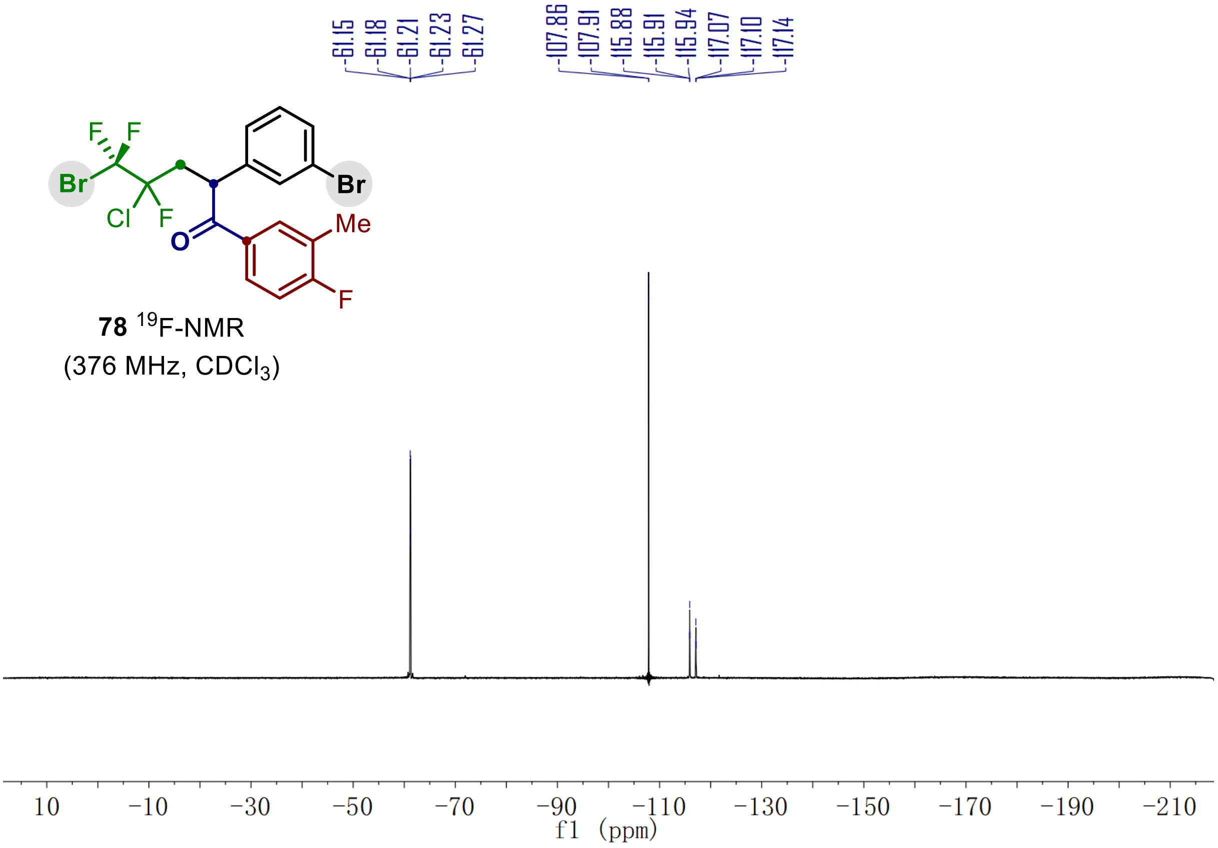

78 ^{19}F-NMR
(376 MHz, $CDCl_3$)

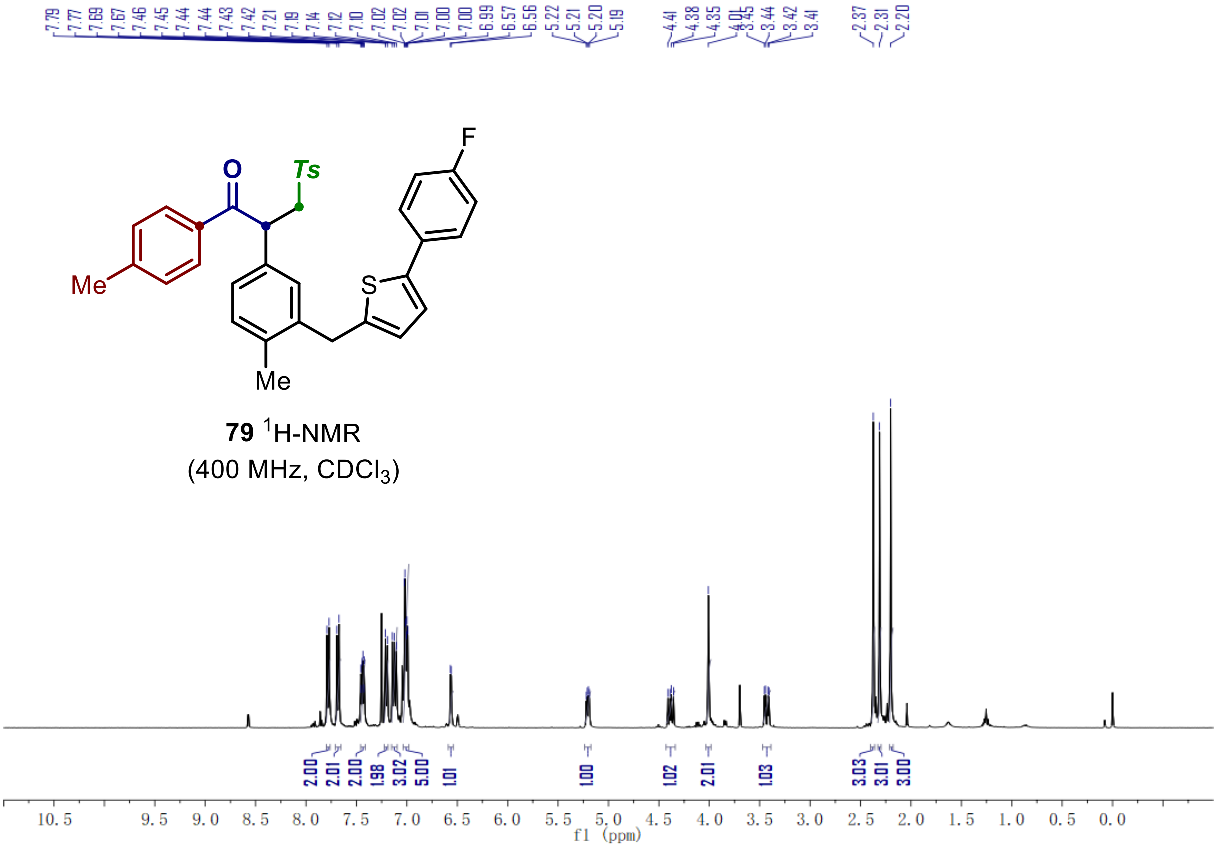

79 ^{1}H-NMR
(400 MHz, $CDCl_3$)

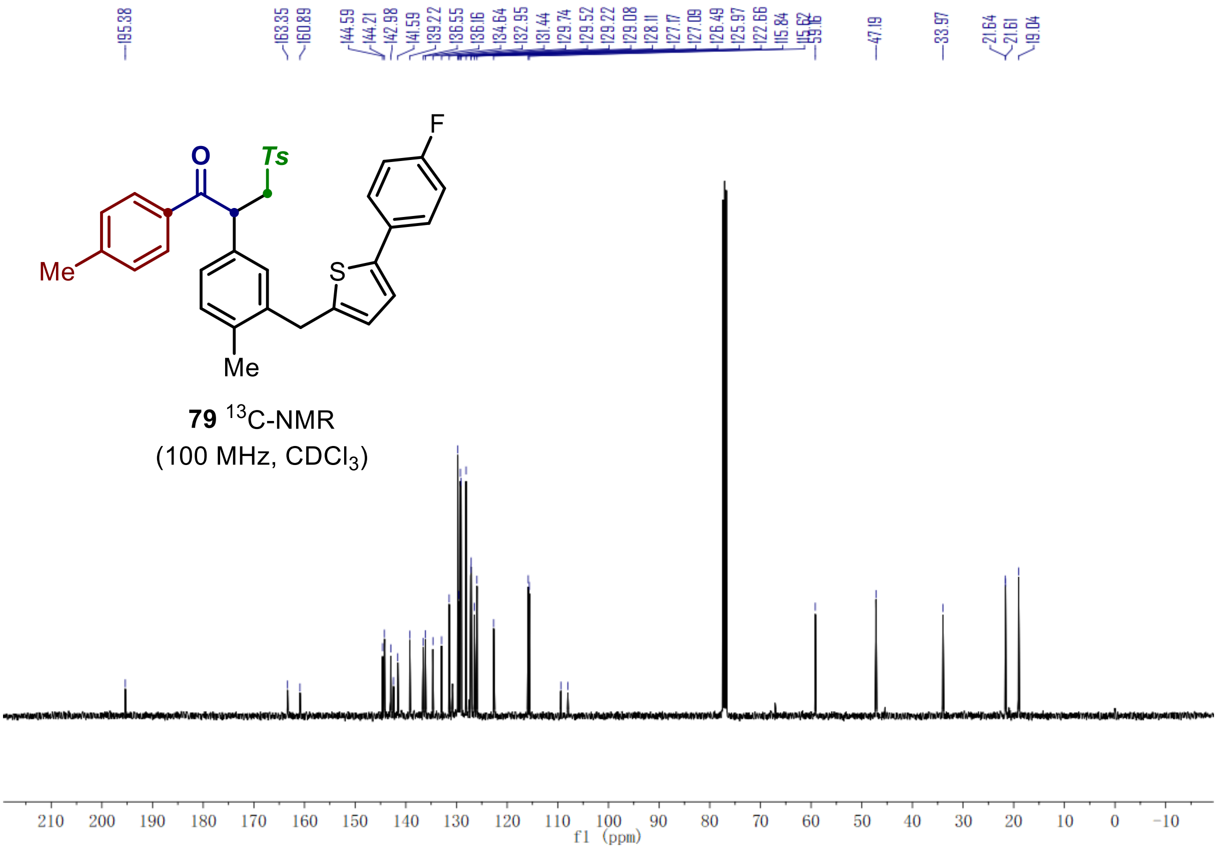

195.38
163.35
160.89
144.59
144.21
142.98
141.59
139.22
136.55
136.16
134.64
132.95
131.44
129.74
129.52
129.22
129.08
128.11
127.17
127.09
126.49
125.97
122.66
115.84
115.62
59.16
47.19
33.97
21.64
21.61
19.04
O
Ts
F
Me
S
Me
79 ^{13}C-NMR
(100 MHz, $CDCl_3$)
f1 (ppm)

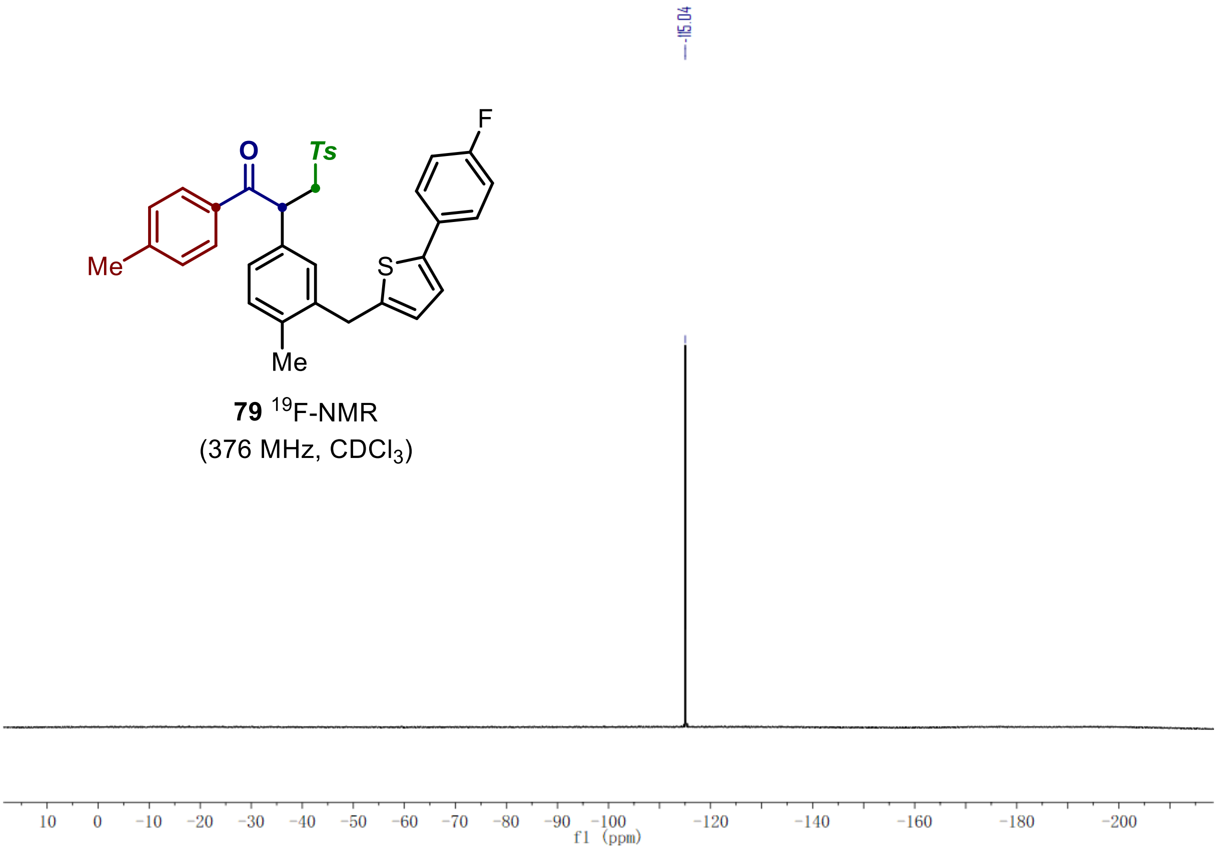

-115.04
O
Ts
F
Me
S
Me
79 ^{19}F-NMR
(376 MHz, $CDCl_3$)
f1 (ppm)

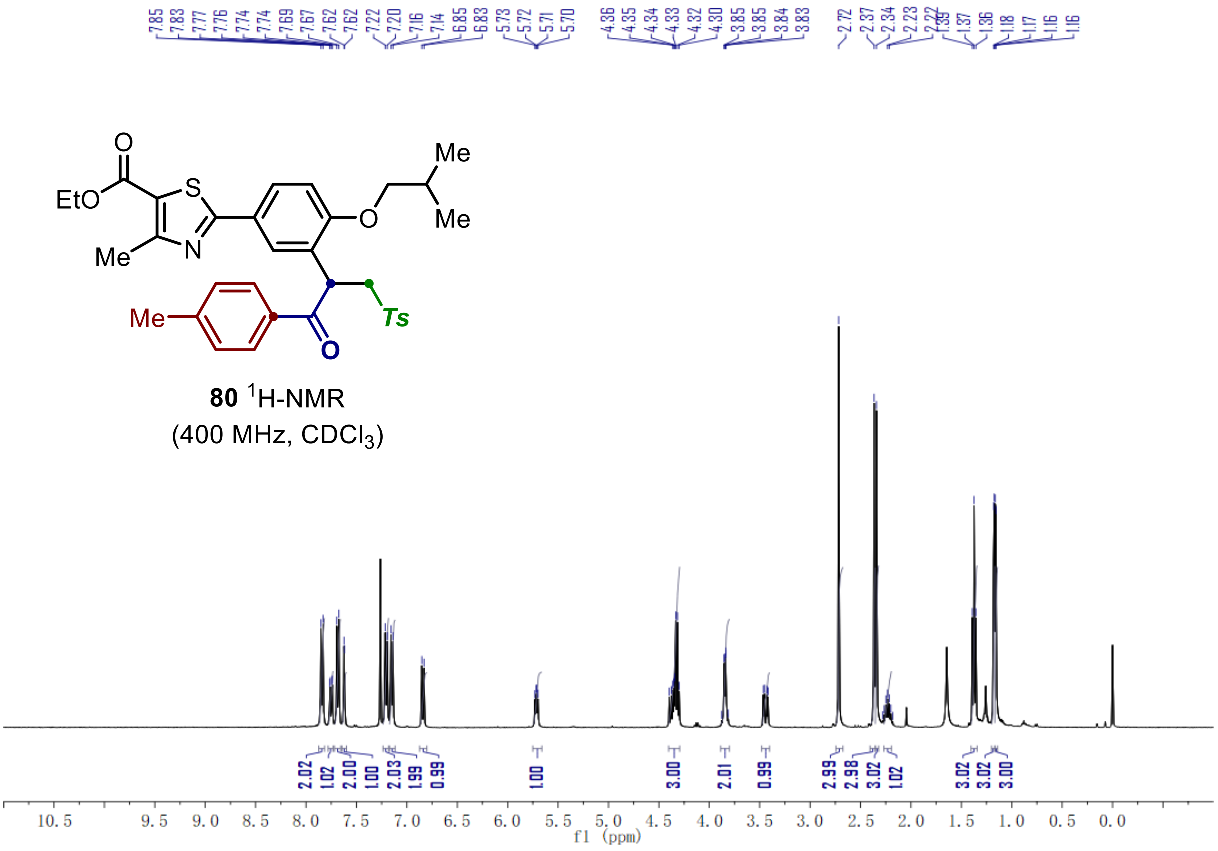

80 ^{1}H-NMR
(400 MHz, $CDCl_3$)

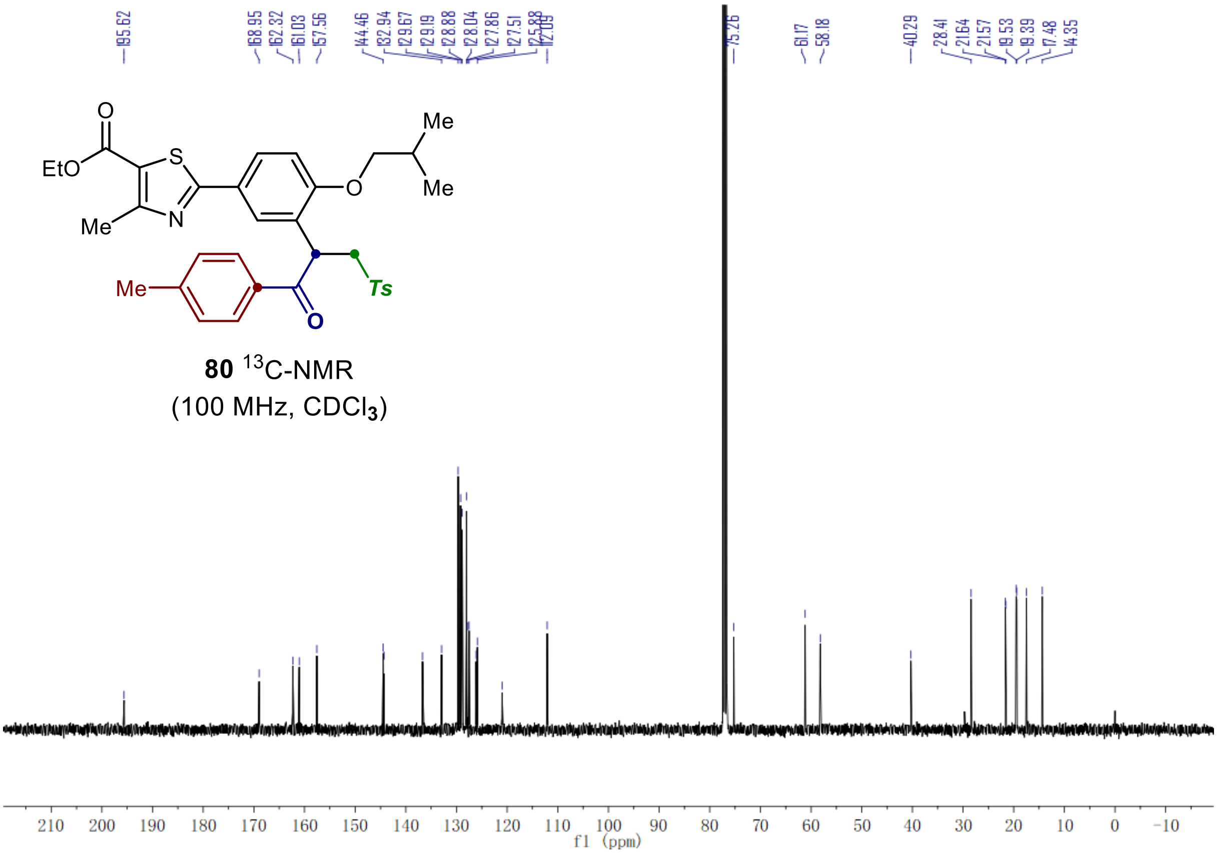

80 ^{13}C-NMR
(100 MHz, $CDCl_3$)

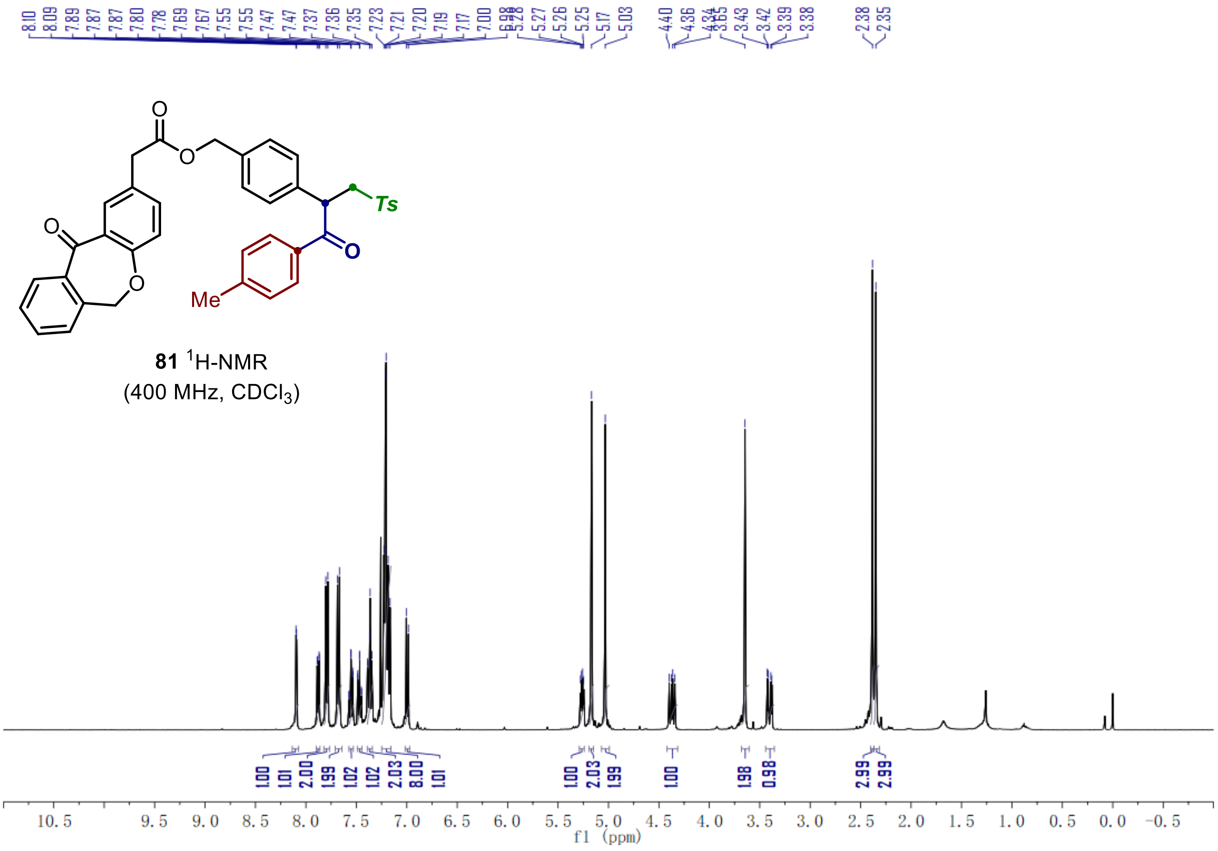
81 1H-NMR
(400 MHz, CDCl3)
f1 (ppm)

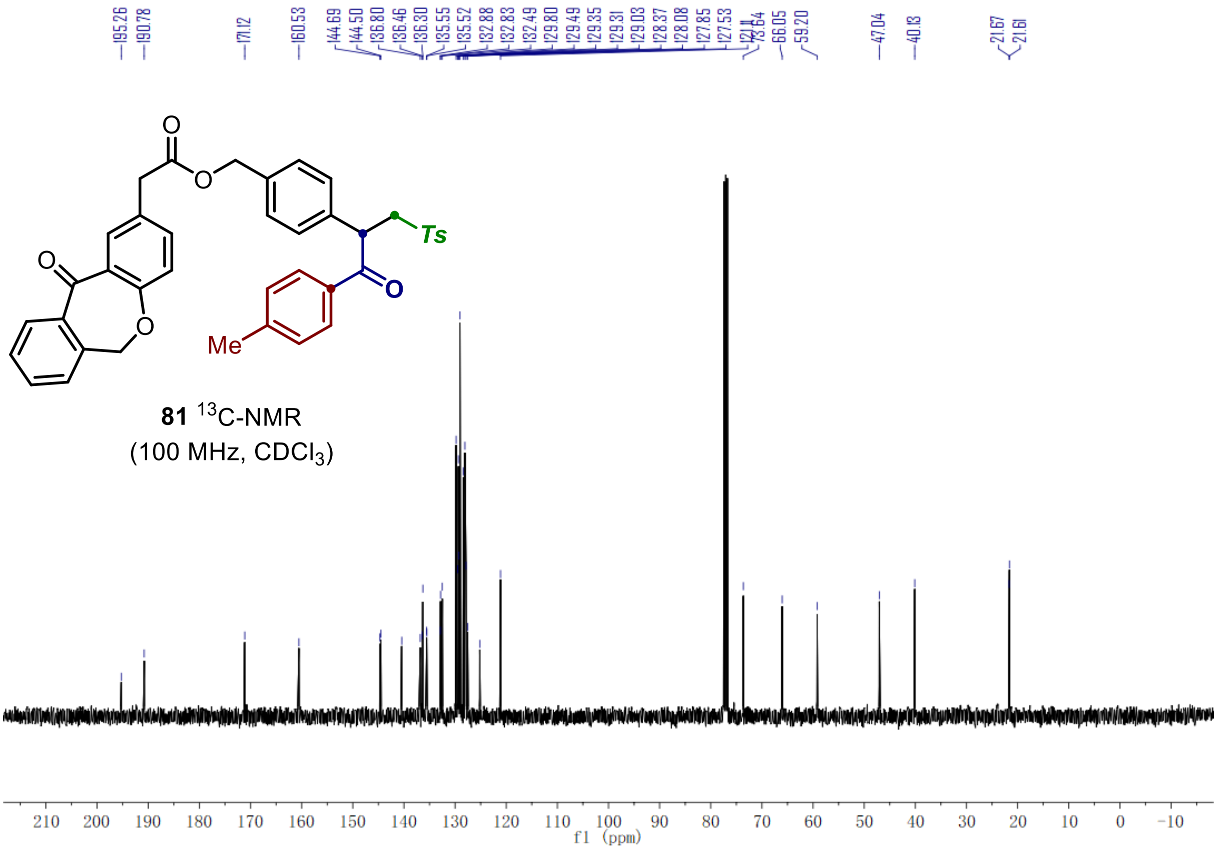
81 13C-NMR
(100 MHz, CDCl3)
f1 (ppm)

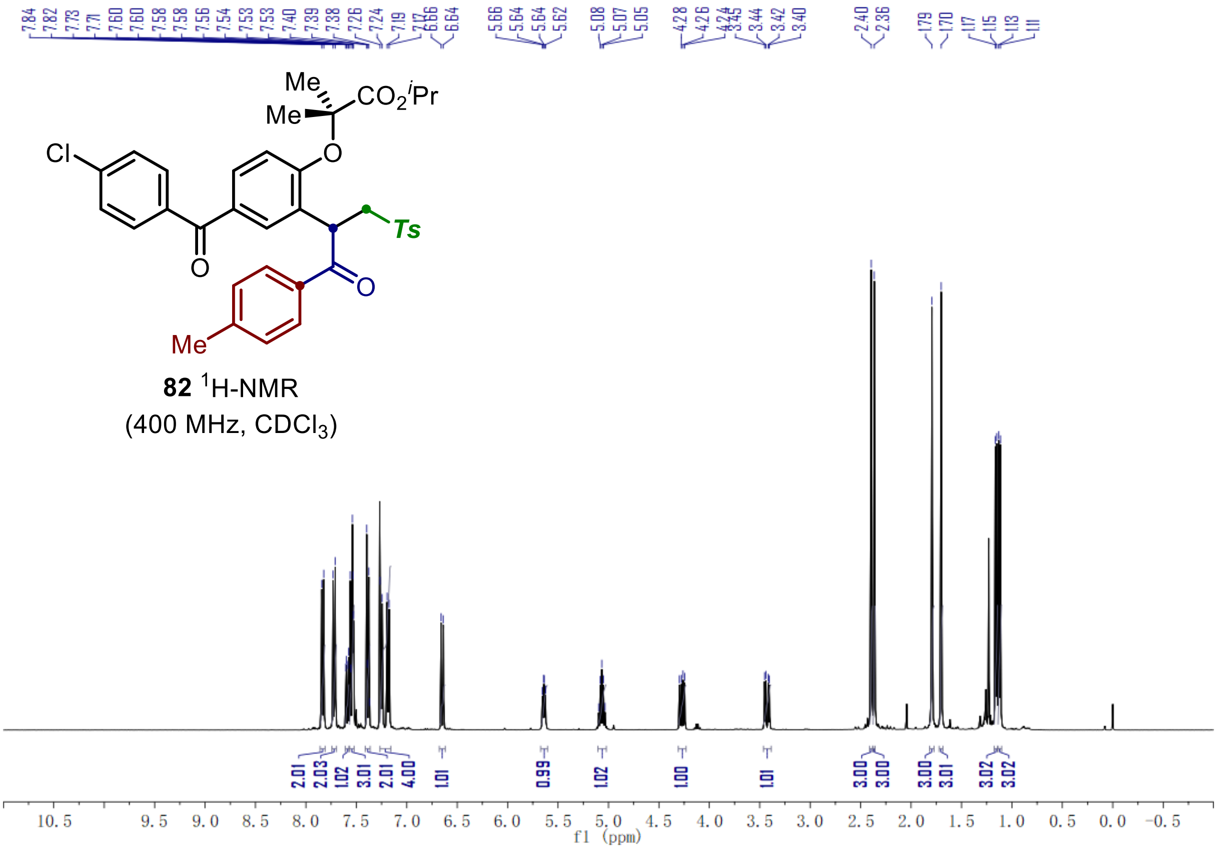

82 ^{1}H-NMR
(400 MHz, $CDCl_3$)

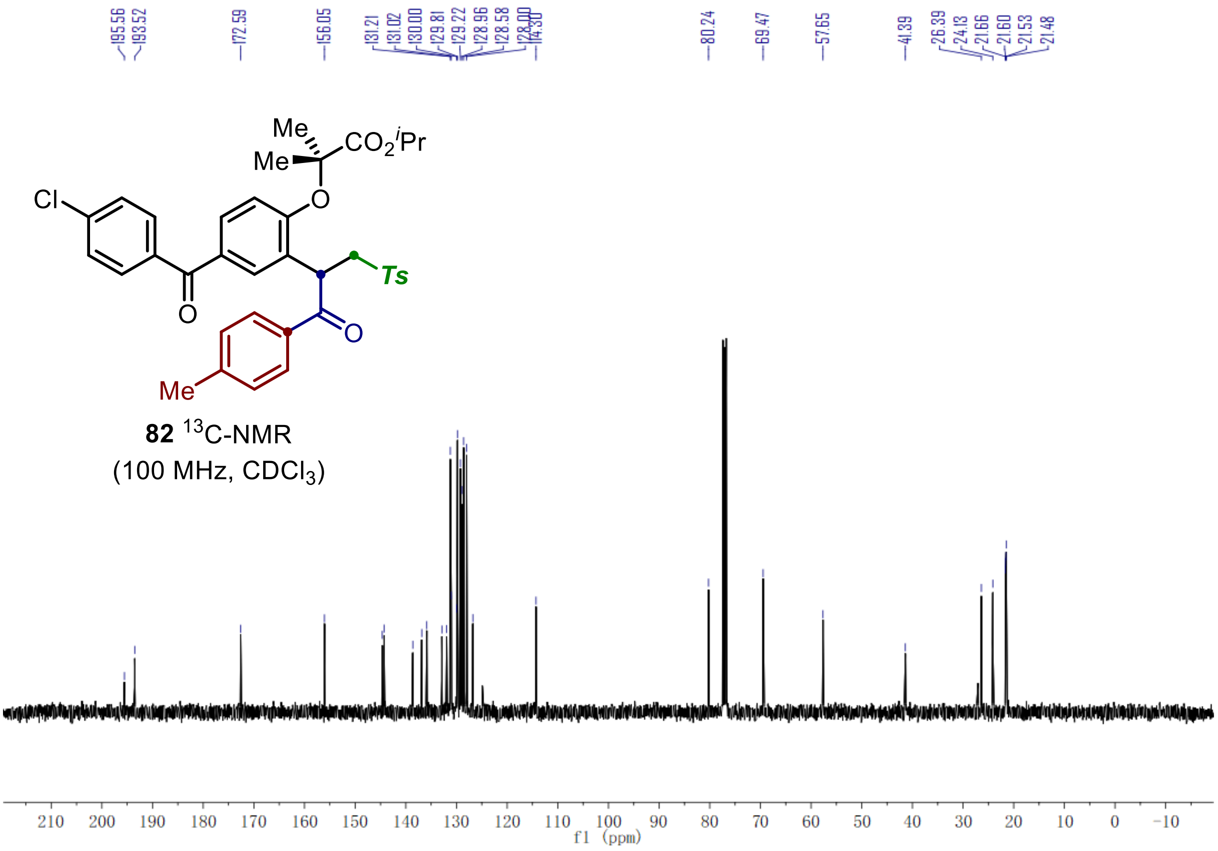

82 ^{13}C-NMR
(100 MHz, $CDCl_3$)

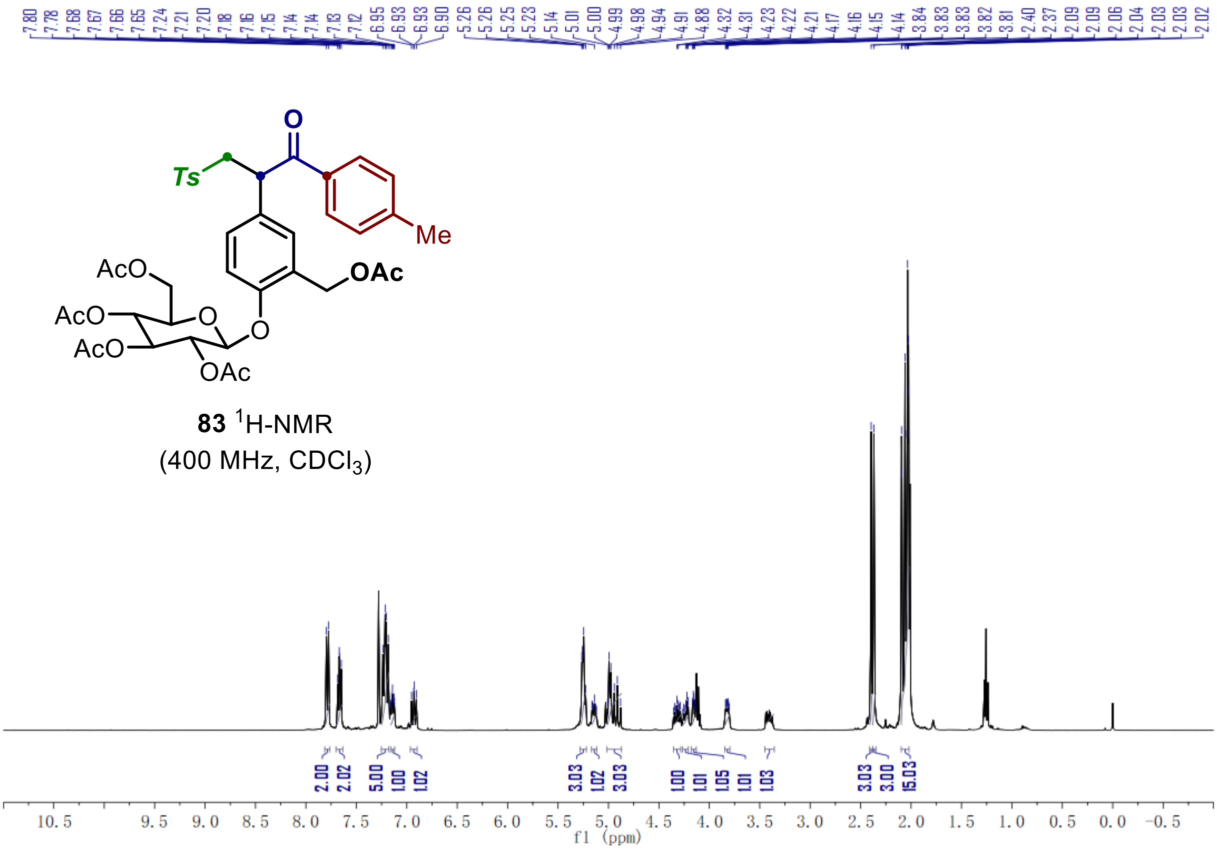

83 ^{1}H-NMR
(400 MHz, $CDCl_3$)

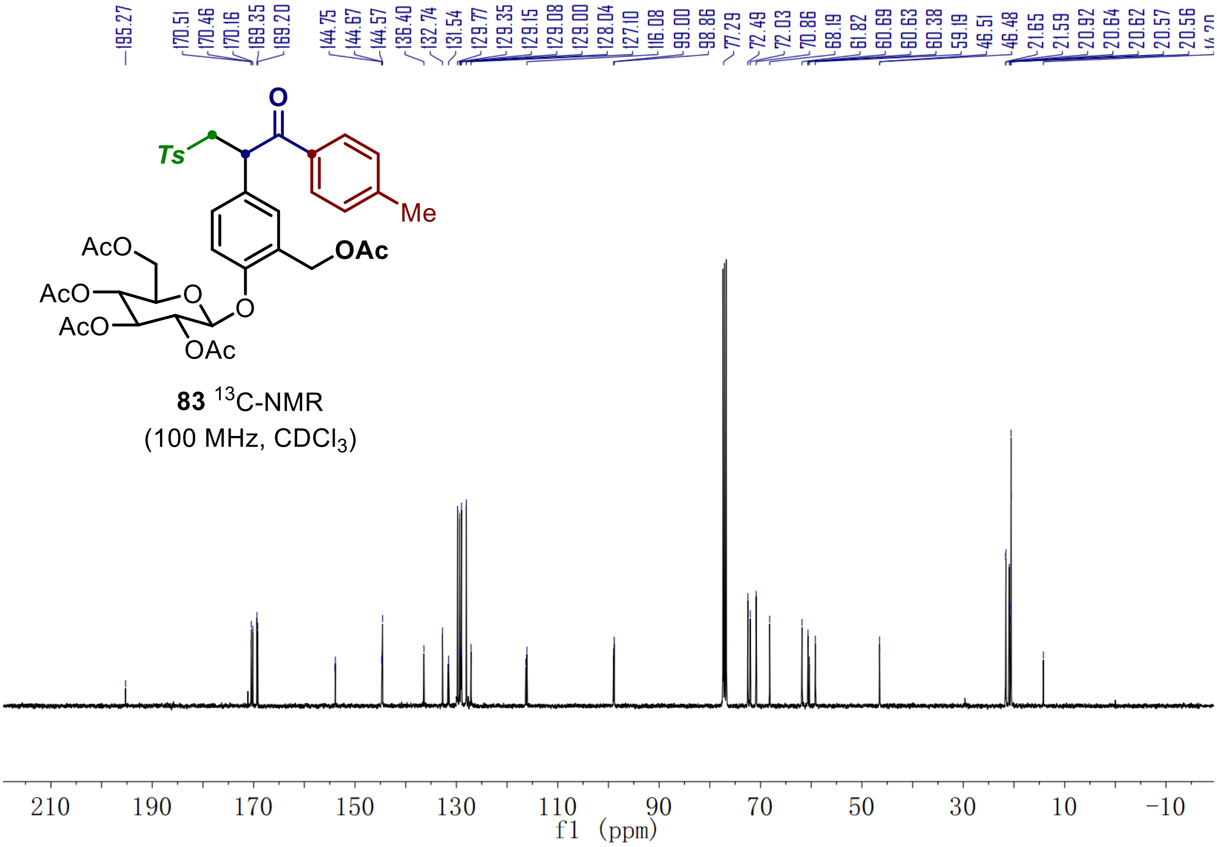

83 ^{13}C-NMR
(100 MHz, $CDCl_3$)

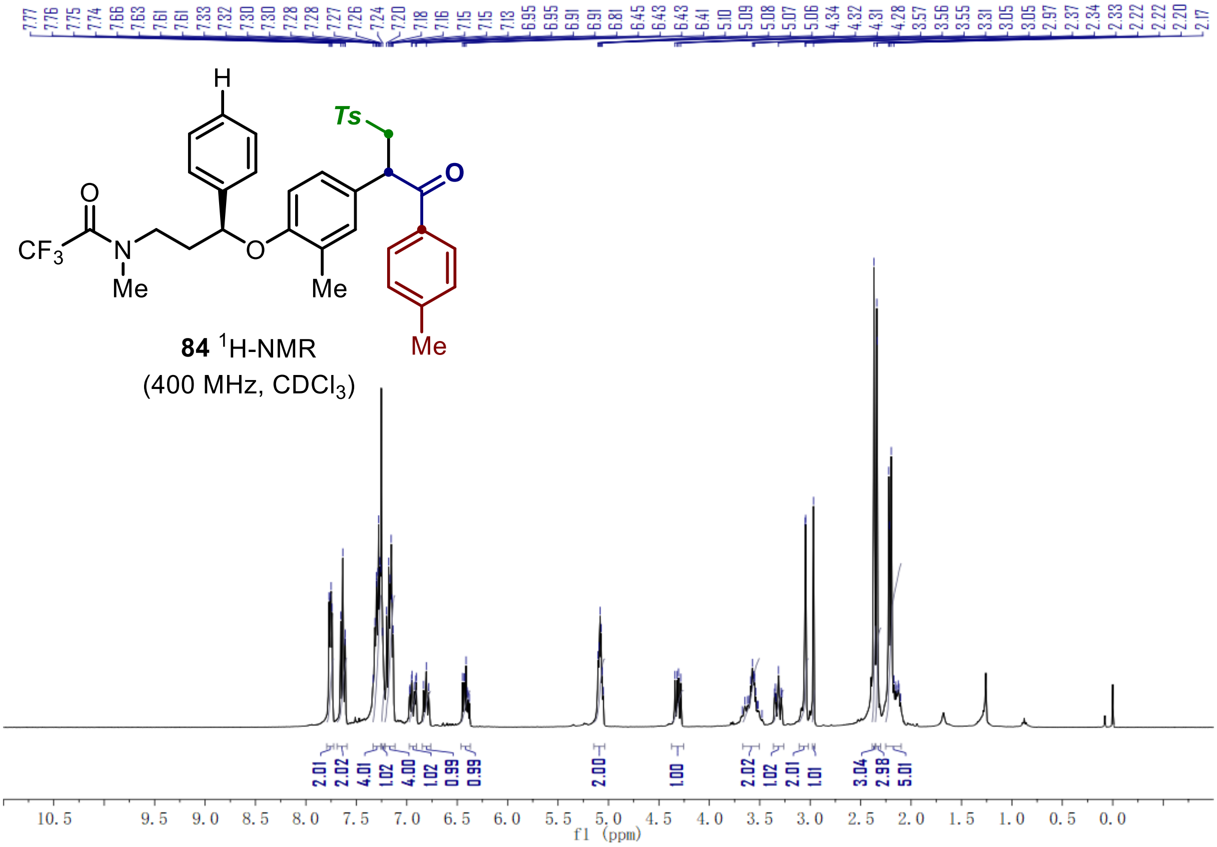
7.77 7.76 7.75 7.74 7.66 7.63 7.61 7.61 7.33 7.32 7.30 7.30 7.28 7.28 7.27 7.26 7.24 7.20 7.18 7.16 7.15 7.15 7.13 6.95 6.95 6.91 6.91 6.81 6.45 6.43 6.43 6.41 5.10 5.09 5.08 5.07 5.06 4.34 4.32 4.31 4.28 3.57 3.56 3.55 3.31 3.05 3.05 2.97 2.37 2.34 2.33 2.22 2.22 2.20 2.17
H
Ts
O
CF3
N
Me
O
Me
Me
84 1H-NMR
(400 MHz, CDCl3)
2.01 2.02 4.01 1.02 4.00 1.02 0.99 0.99 2.00 1.00 2.02 1.02 2.01 1.01 3.04 2.98 5.01
10.5 9.5 9.0 8.5 8.0 7.5 7.0 6.5 6.0 5.5 5.0 4.5 4.0 3.5 3.0 2.5 2.0 1.5 1.0 0.5 0.0
f1 (ppm)

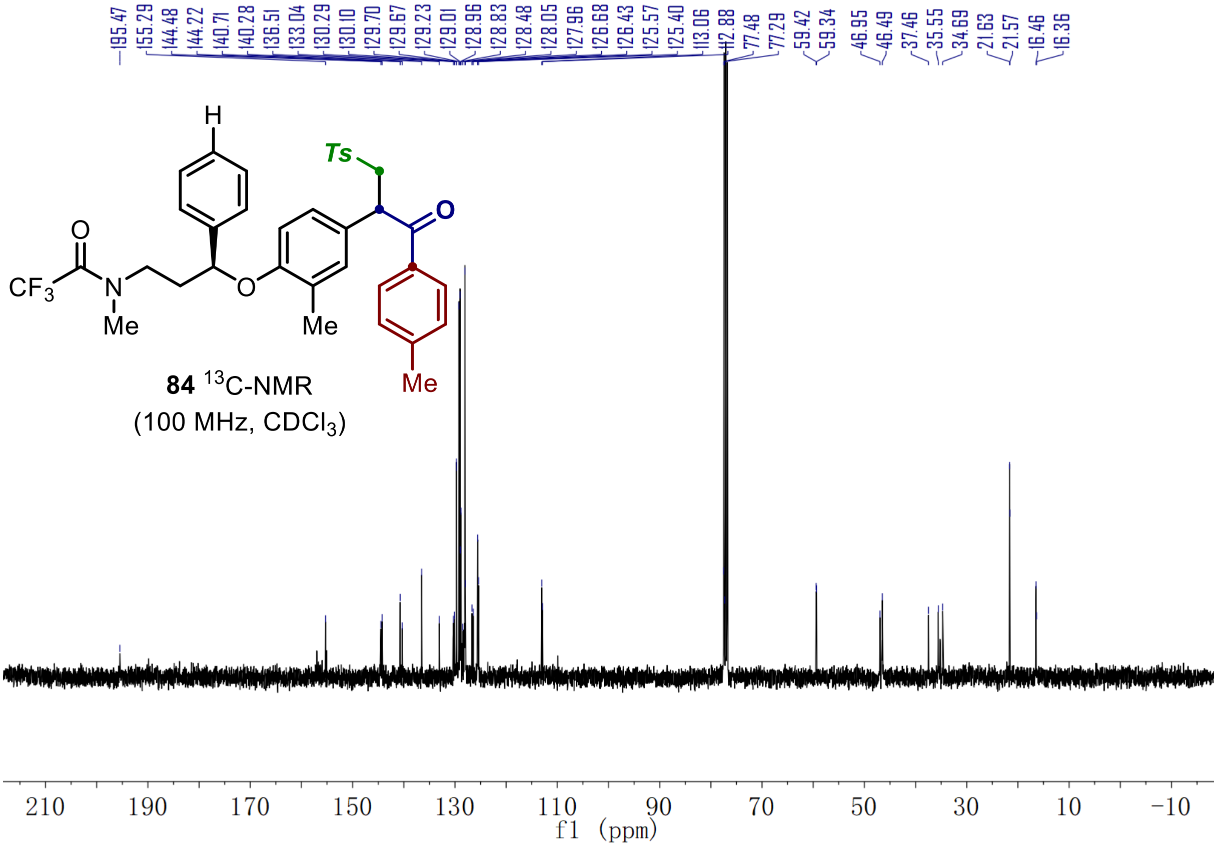
195.47 155.29 144.48 144.22 140.71 140.28 136.51 133.04 130.29 130.10 129.70 129.67 129.23 129.01 128.96 128.83 128.48 128.05 127.96 126.68 126.43 125.57 125.40 113.06 112.88 77.48 77.29 59.42 59.34 46.95 46.49 37.46 35.55 34.69 21.63 21.57 16.46 16.36
H
Ts
O
CF3
N
Me
O
Me
Me
84 13C-NMR
(100 MHz, CDCl3)
210 190 170 150 130 110 90 70 50 30 10 -10
f1 (ppm)

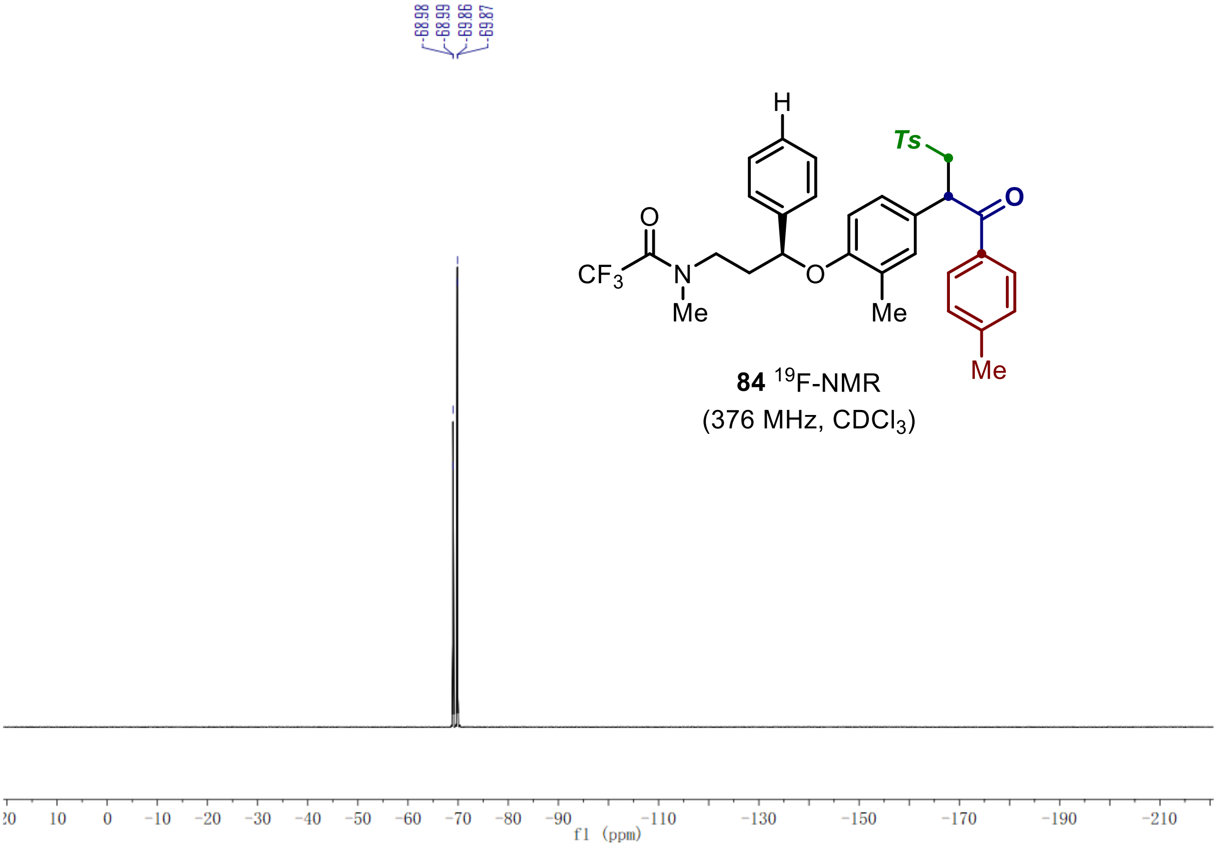
-68.98
-68.99
-69.86
-69.87
H
Ts
O
O
CF3
N
Me
O
Me
Me
84 19F-NMR
(376 MHz, CDCl3)
f1 (ppm)

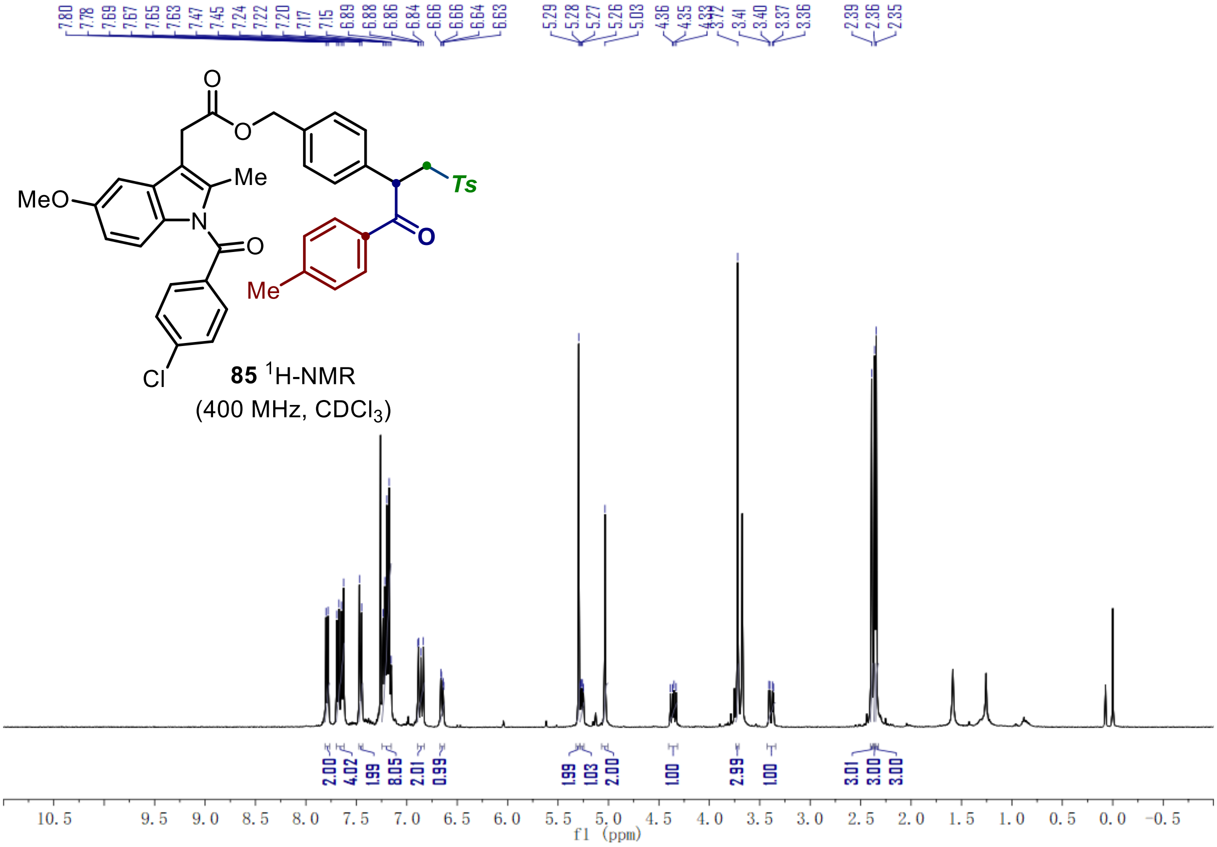
O
O
Me
Ts
MeO
N
O
O
Me
Cl
85 1H-NMR
(400 MHz, CDCl3)
f1 (ppm)

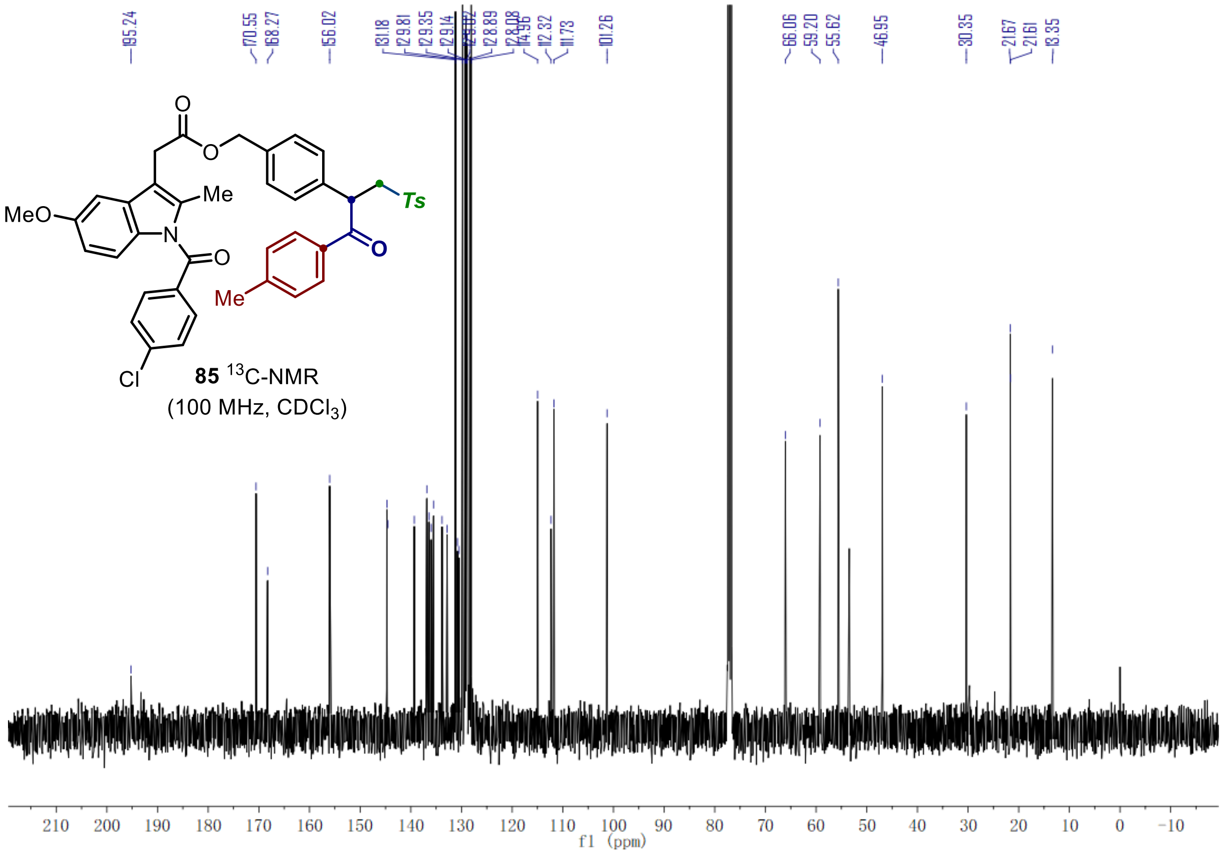

195.24
170.55
168.27
156.02
131.18
129.81
129.35
129.14
128.89
128.08
114.96
112.32
111.73
101.26
66.06
59.20
55.62
46.95
30.35
21.67
21.61
13.35
MeO
Me
N
O
Ts
Me
Cl
85 13C-NMR
(100 MHz, CDCl3)
f1 (ppm)

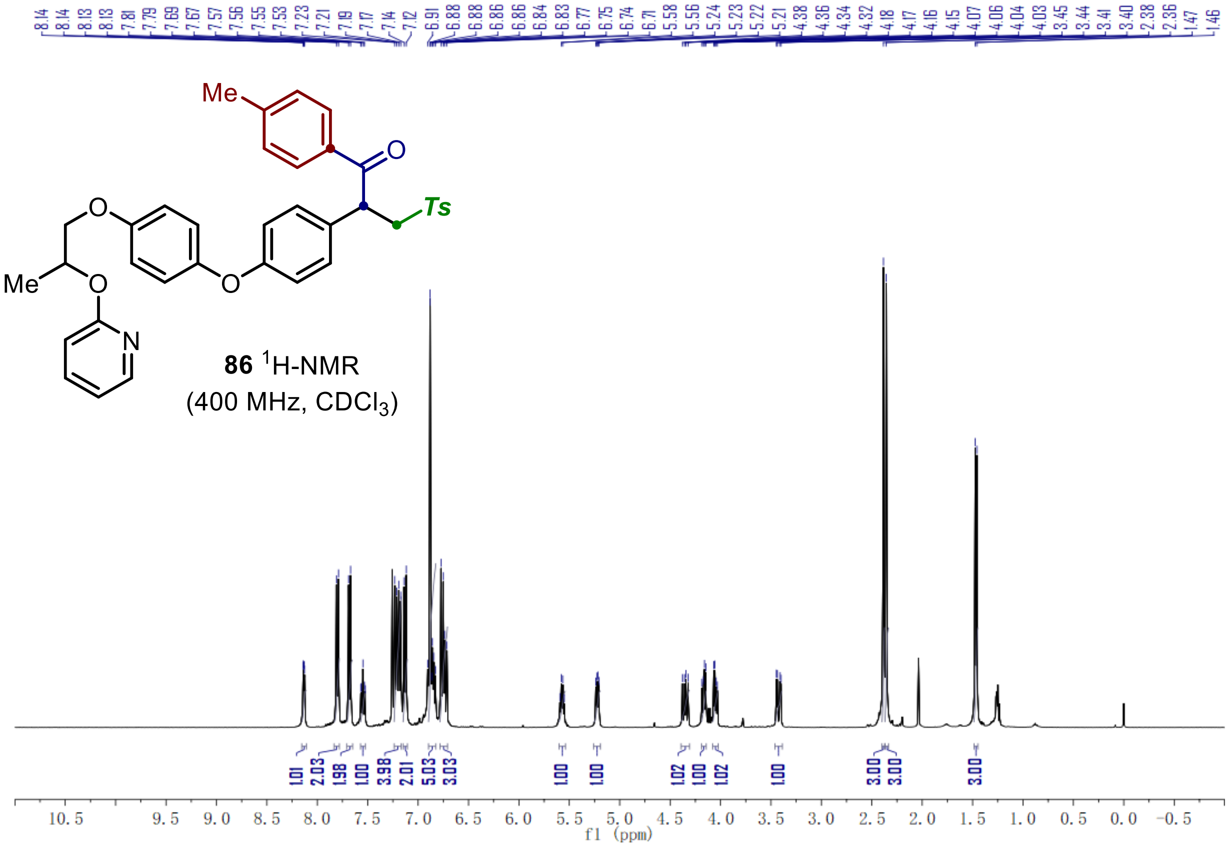

8.14
8.14
8.13
8.13
7.81
7.79
7.69
7.67
7.57
7.56
7.55
7.53
7.23
7.21
7.19
7.17
7.14
7.12
6.91
6.88
6.88
6.86
6.86
6.84
6.83
6.77
6.75
6.74
6.71
5.58
5.56
5.24
5.23
5.22
5.21
4.38
4.36
4.34
4.32
4.18
4.17
4.16
4.15
4.07
4.06
4.04
4.03
3.45
3.44
3.41
3.40
2.38
2.36
1.47
1.46
Me
O
Ts
Me
N
86 1H-NMR
(400 MHz, CDCl3)
1.01
2.03
1.98
1.00
3.98
2.01
5.03
3.03
1.00
1.00
1.02
1.00
1.02
1.00
3.00
3.00
3.00
f1 (ppm)

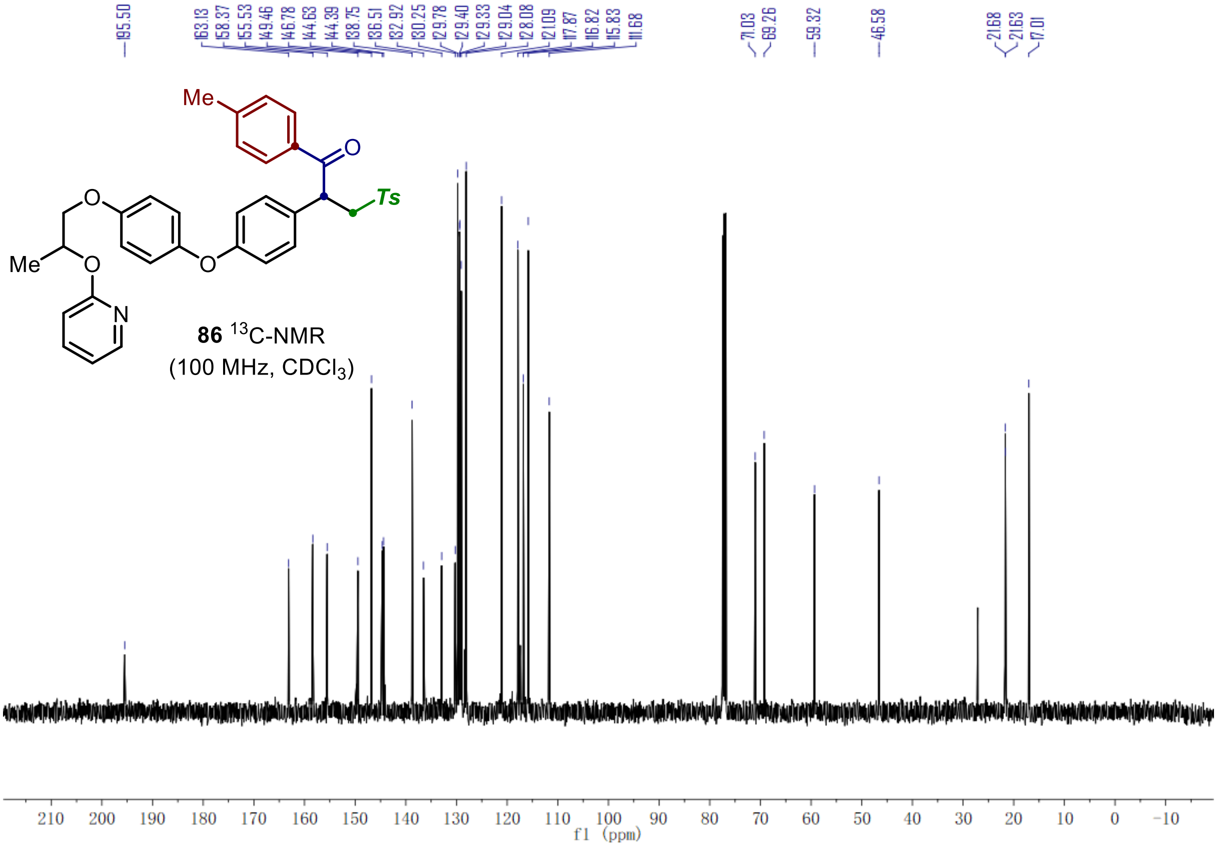

86 ^{13}C-NMR
(100 MHz, $CDCl_3$)

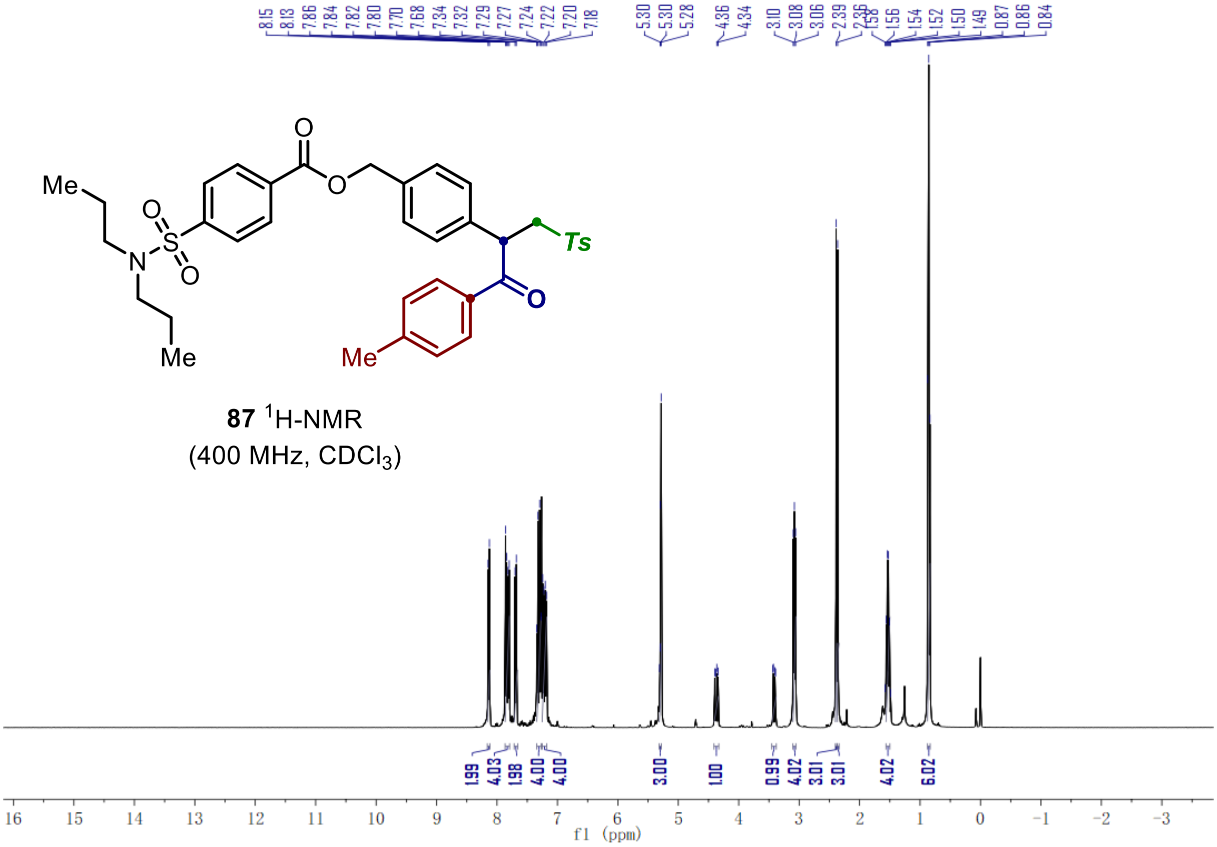

87 ^{1}H-NMR
(400 MHz, $CDCl_3$)

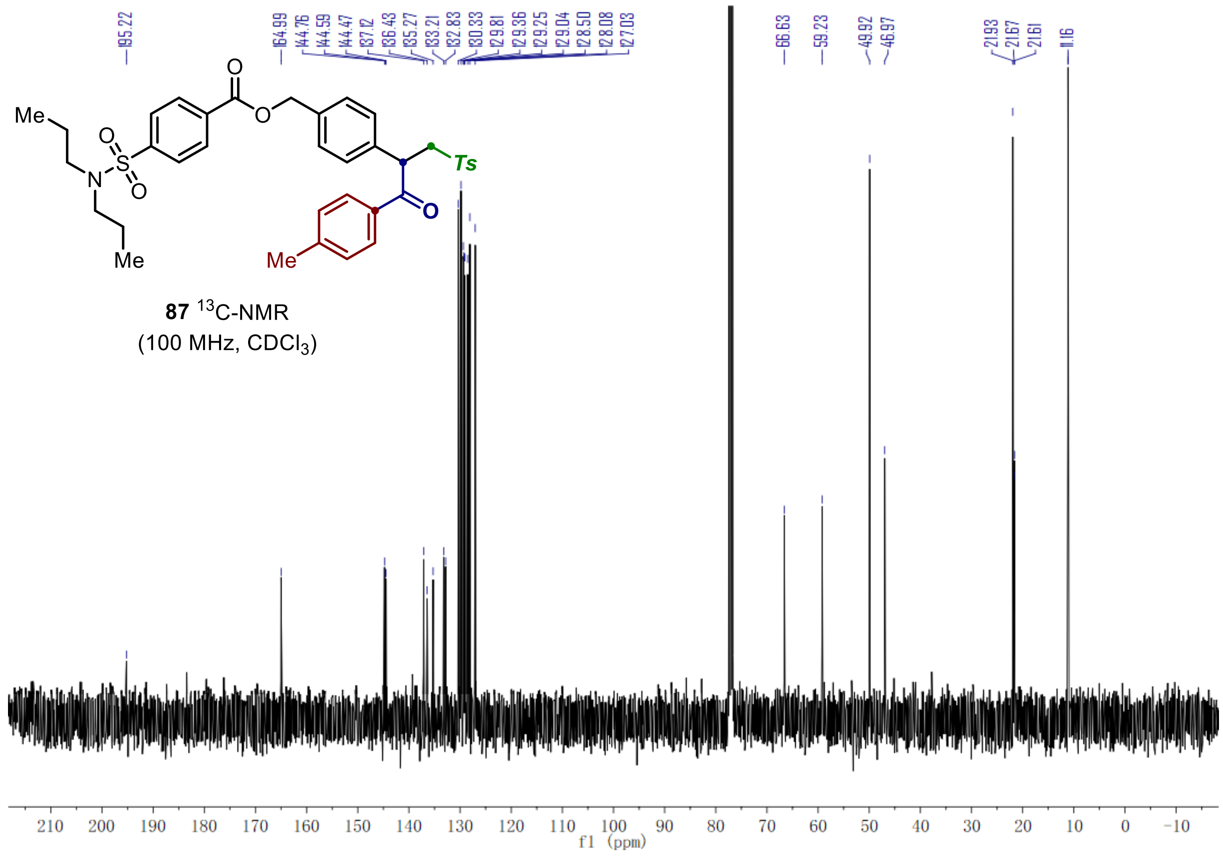

87 ^{13}C-NMR (100 MHz, $CDCl_3$)

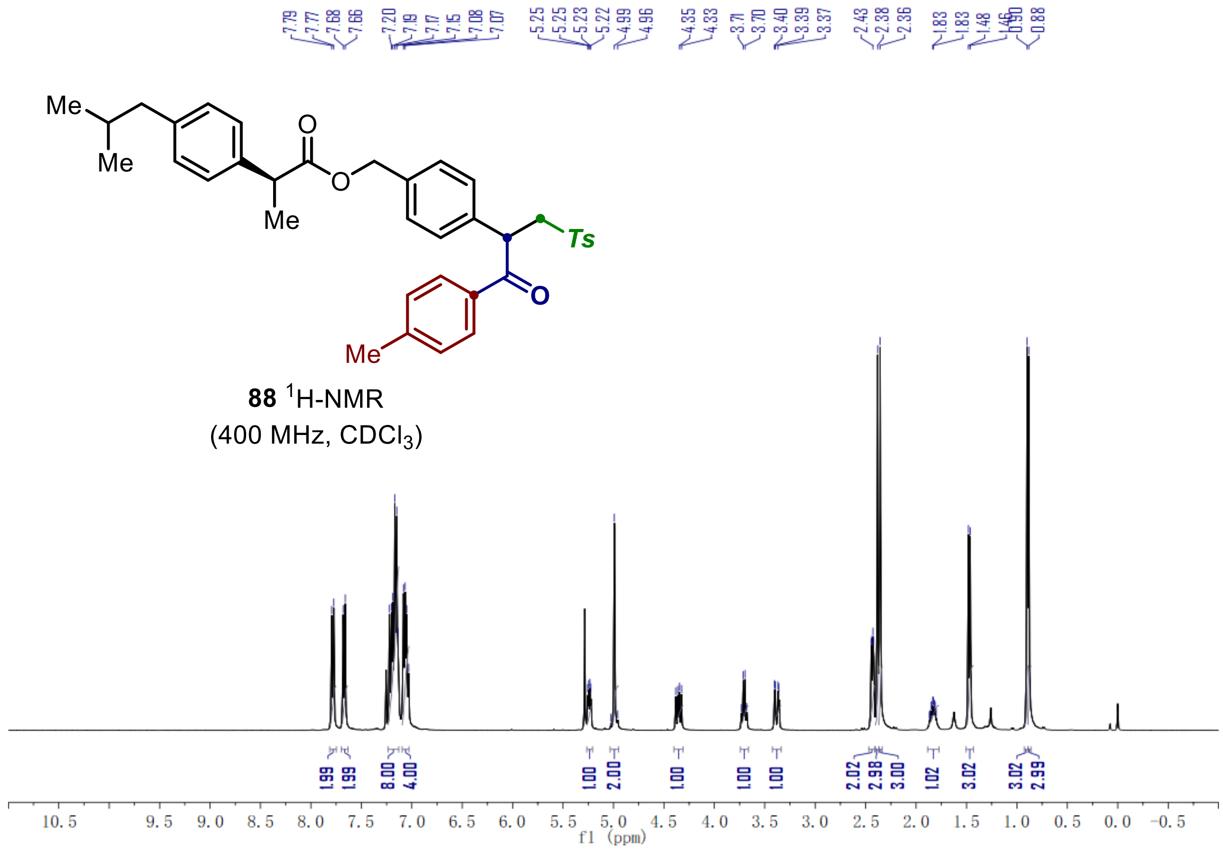

88 ^{1}H-NMR (400 MHz, $CDCl_3$)

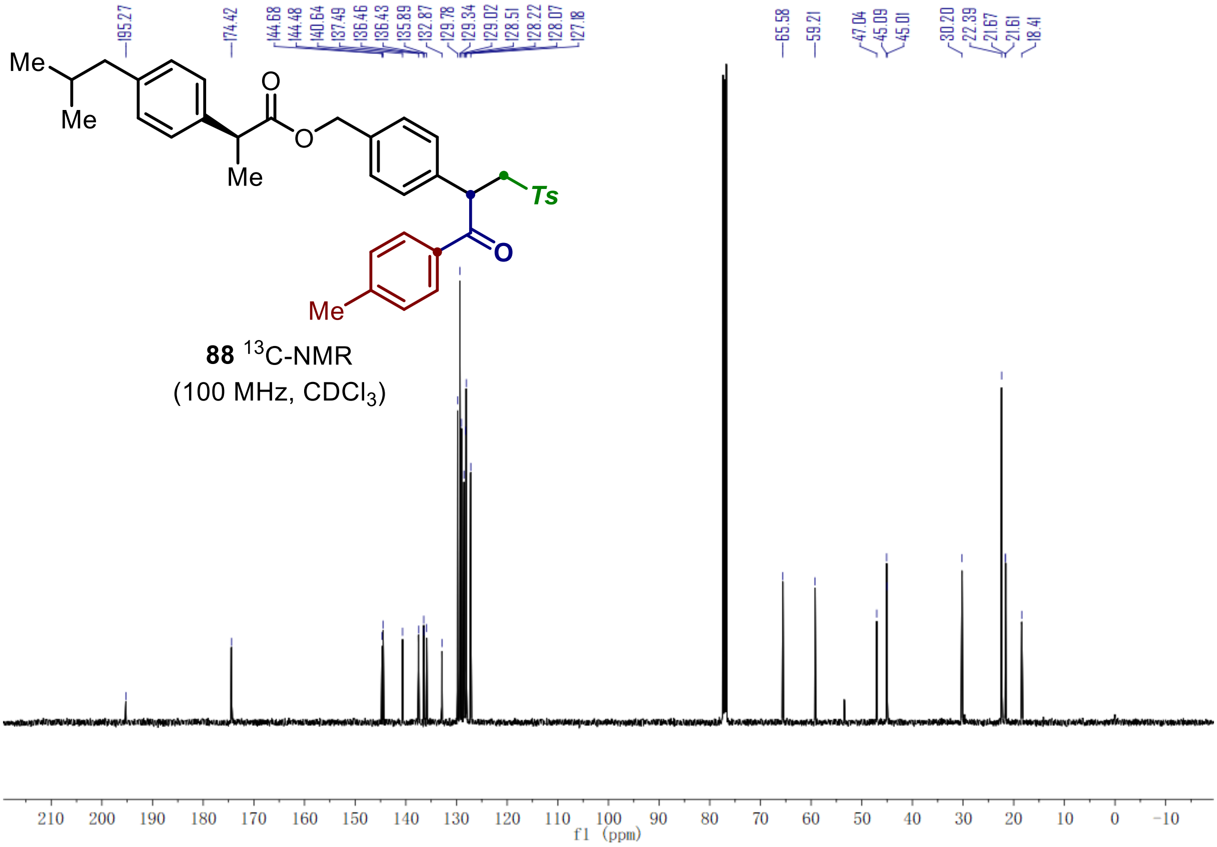

88 ^{13}C-NMR (100 MHz, $CDCl_3$)

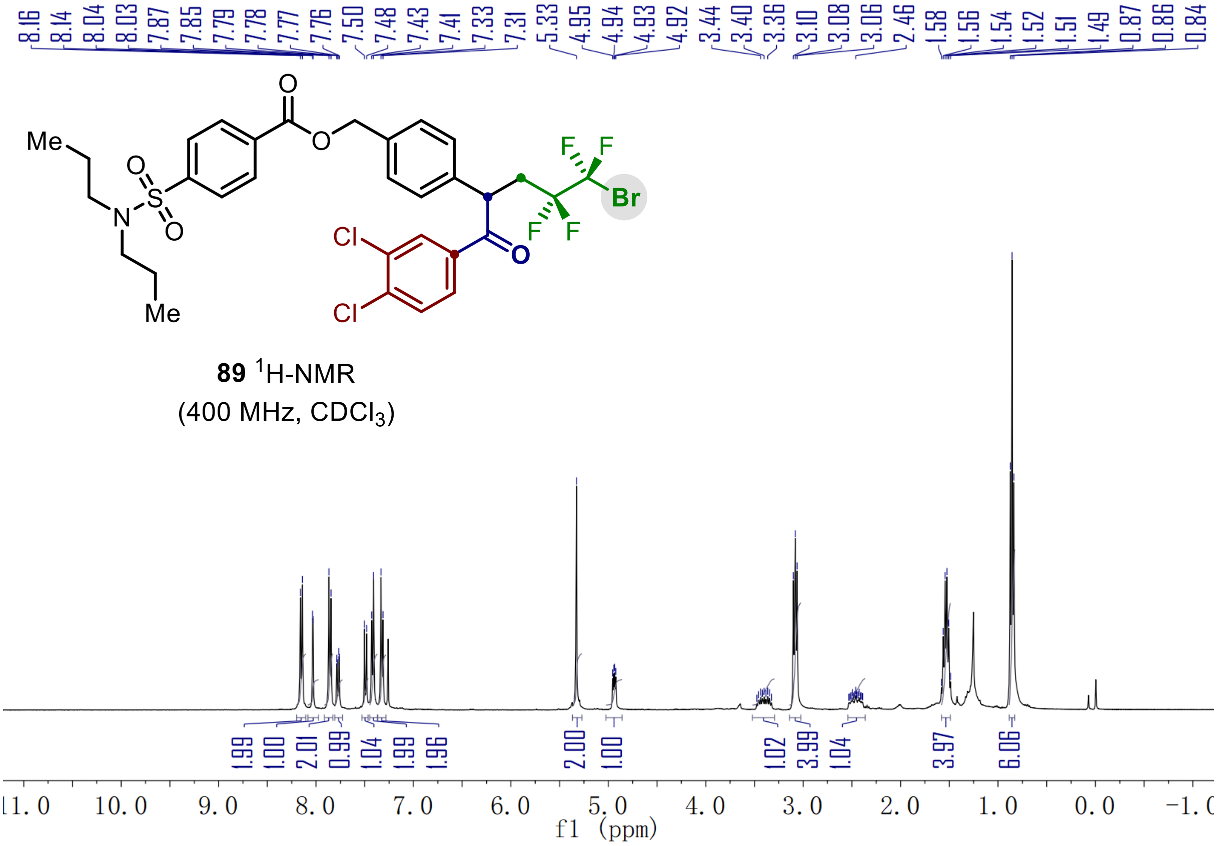

89 ^{1}H-NMR (400 MHz, $CDCl_3$)

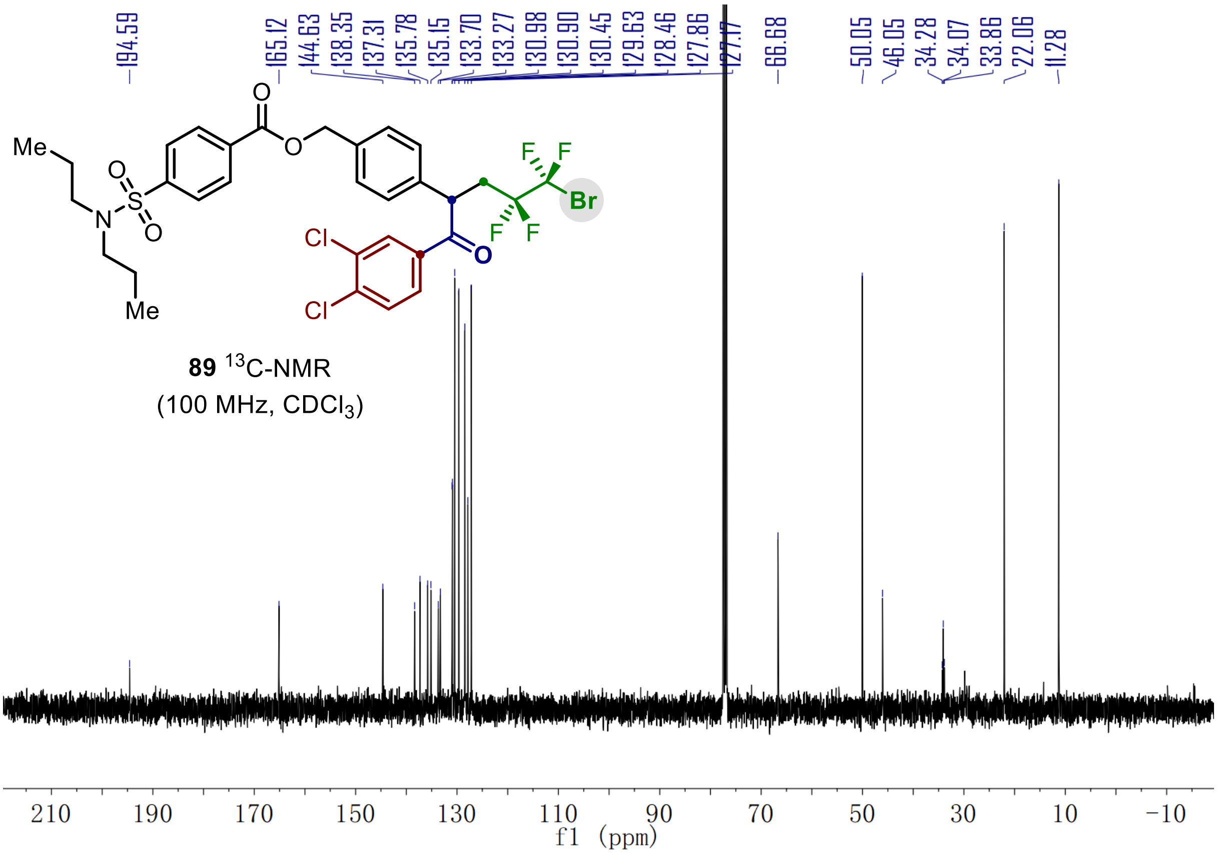

89 ^{13}C-NMR
(100 MHz, $CDCl_3$)

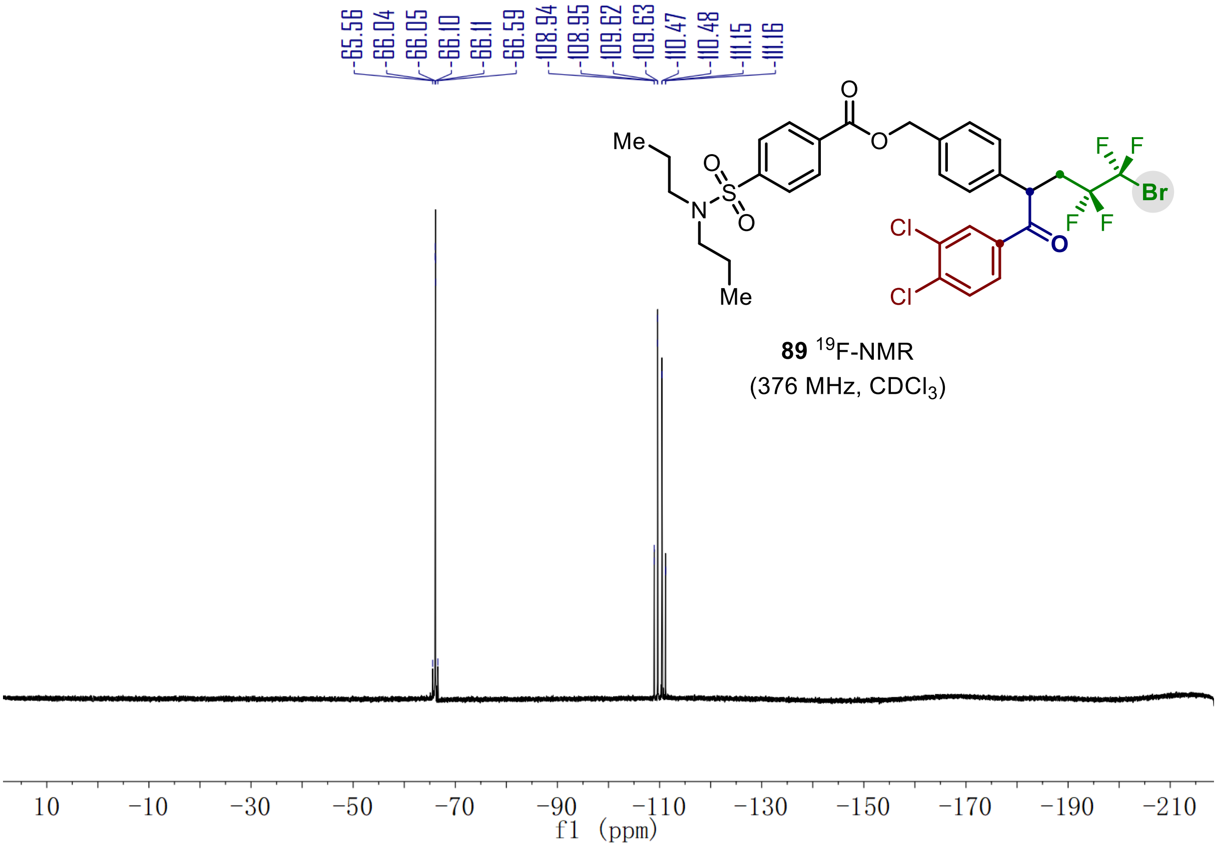

89 ^{19}F-NMR
(376 MHz, $CDCl_3$)

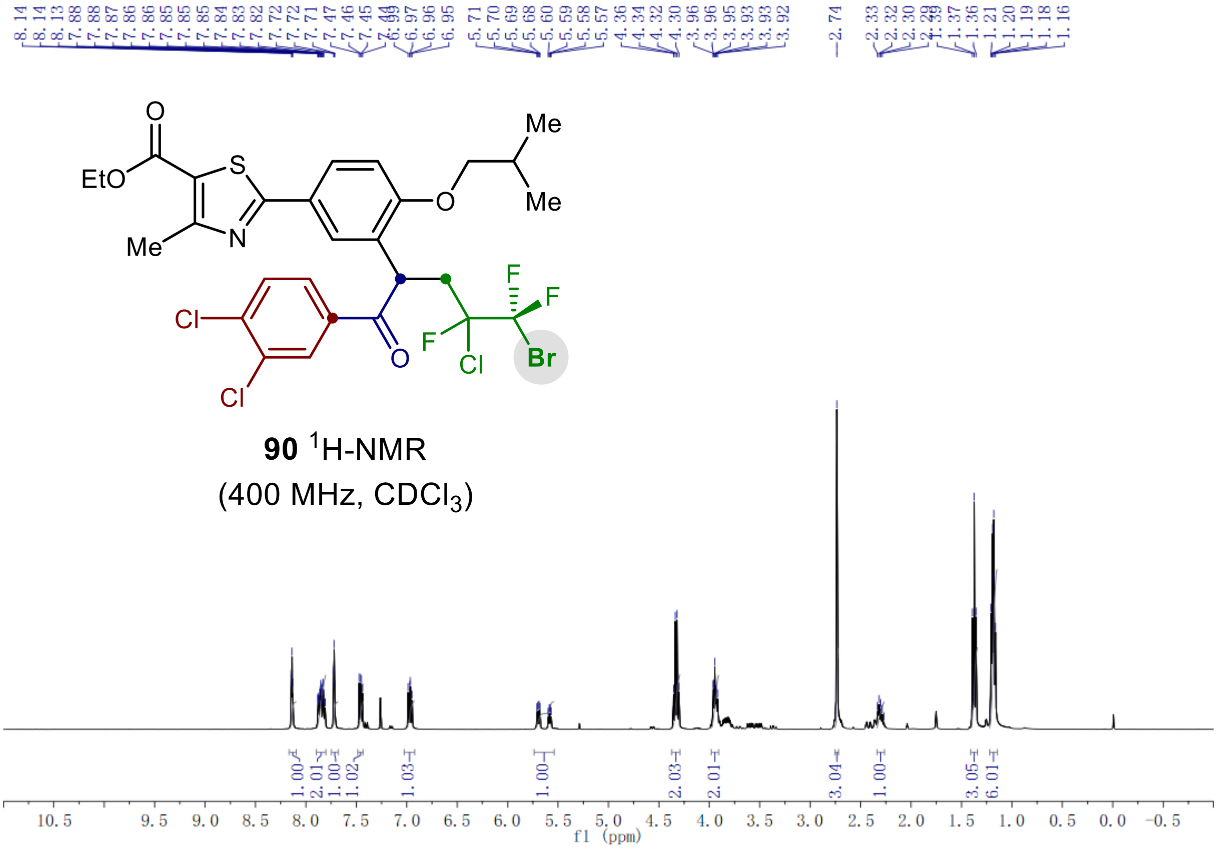

90 ^{1}H-NMR
(400 MHz, $CDCl_3$)

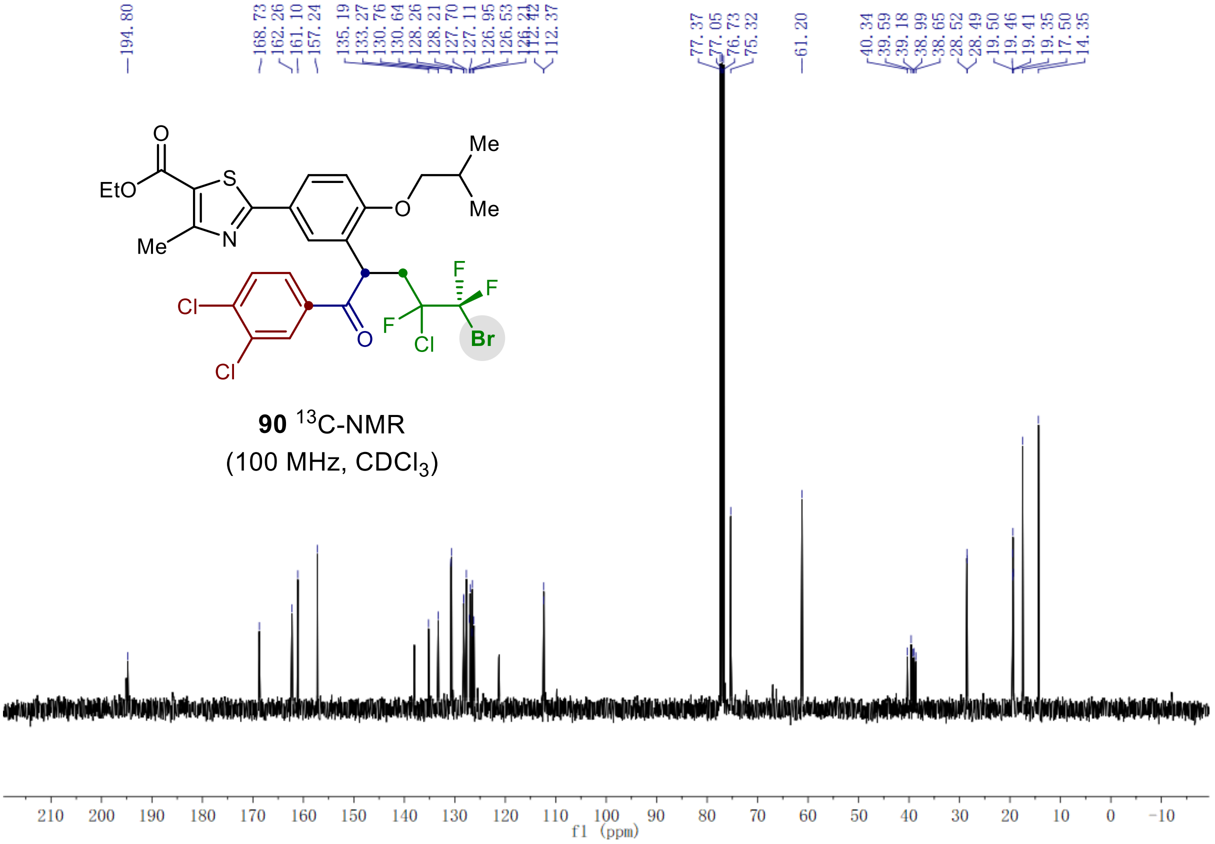

90 ^{13}C-NMR
(100 MHz, $CDCl_3$)

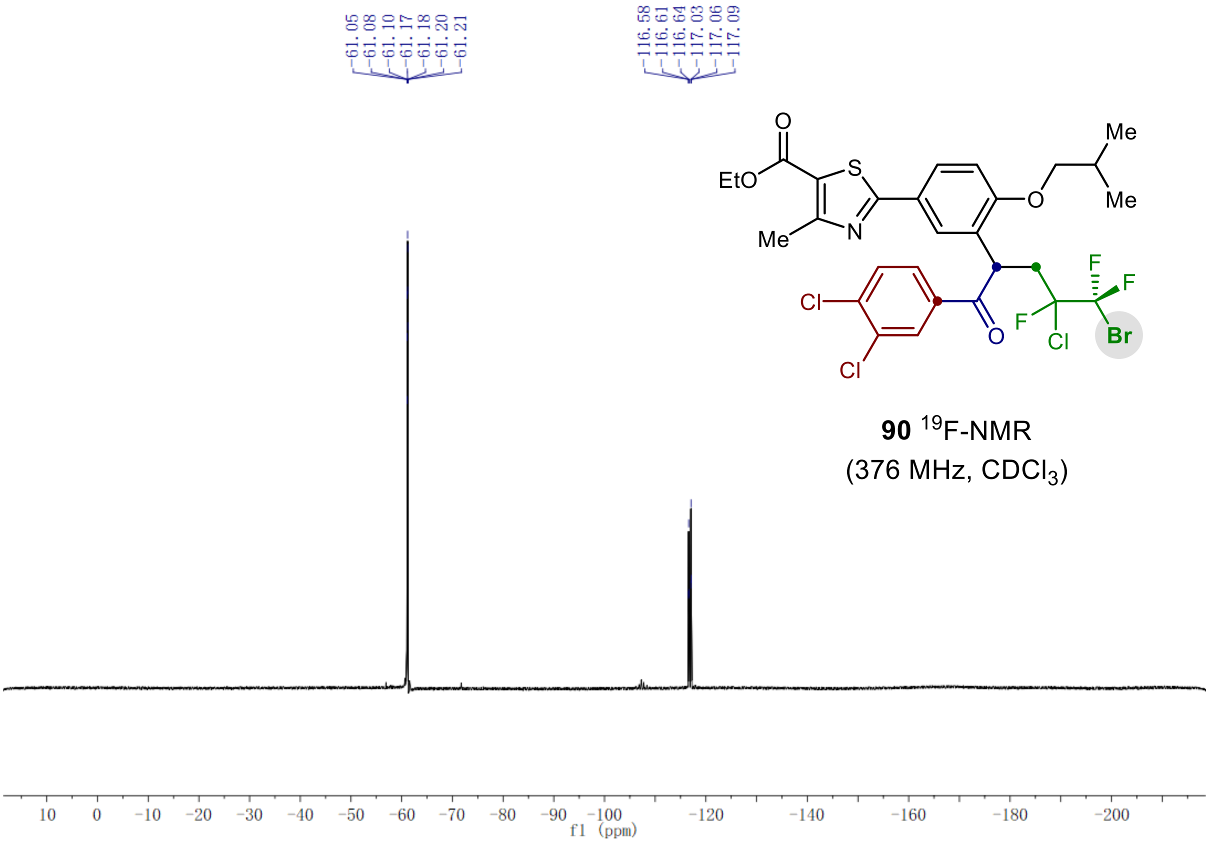

90 ^{19}F-NMR
(376 MHz, $CDCl_3$)

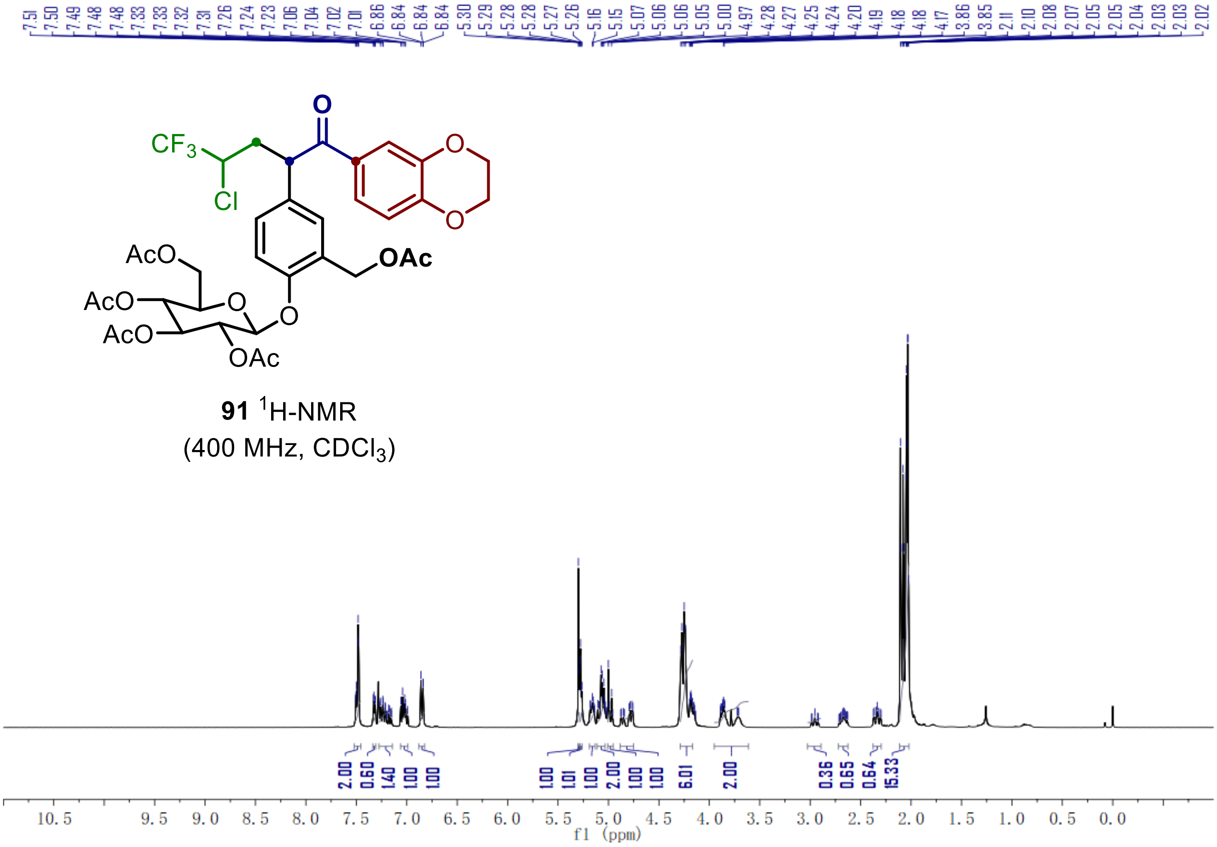

91 ^{1}H-NMR
(400 MHz, $CDCl_3$)

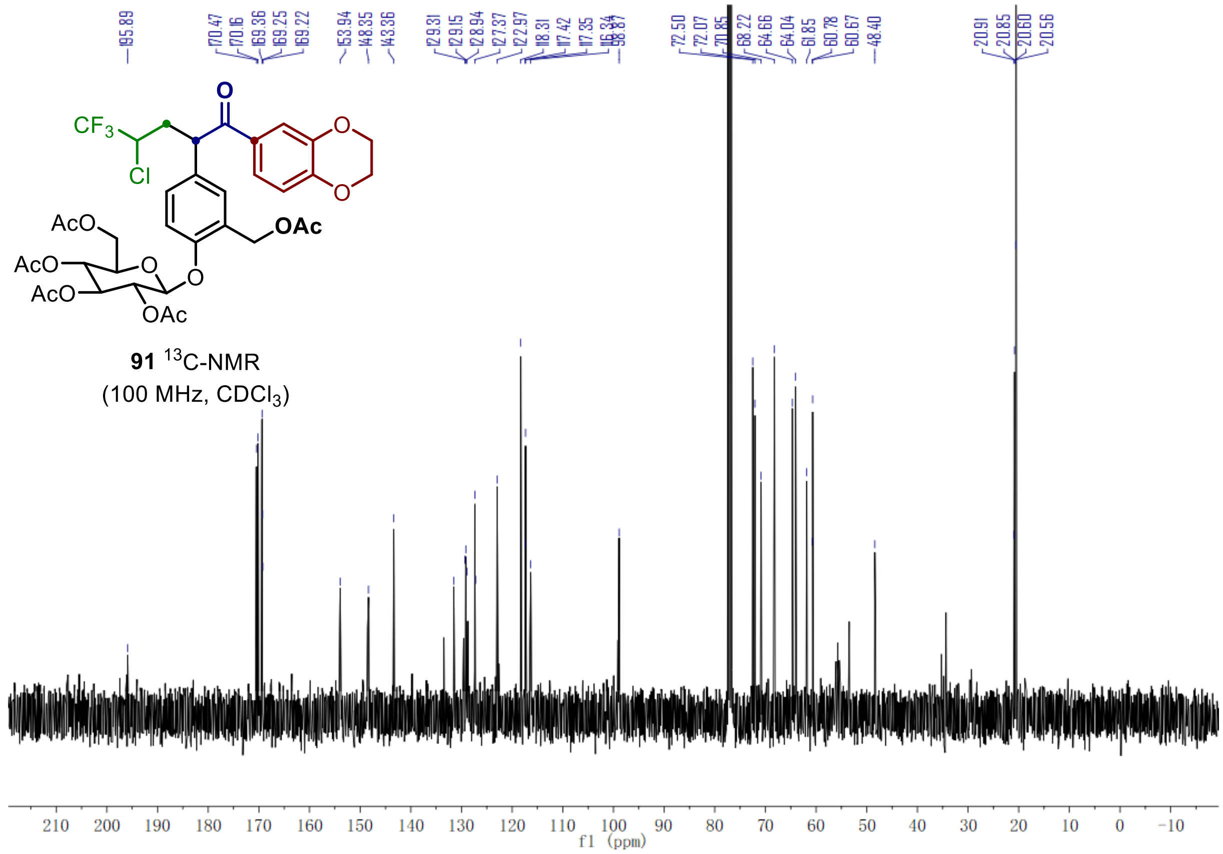

91 ^{13}C-NMR
(100 MHz, $CDCl_3$)

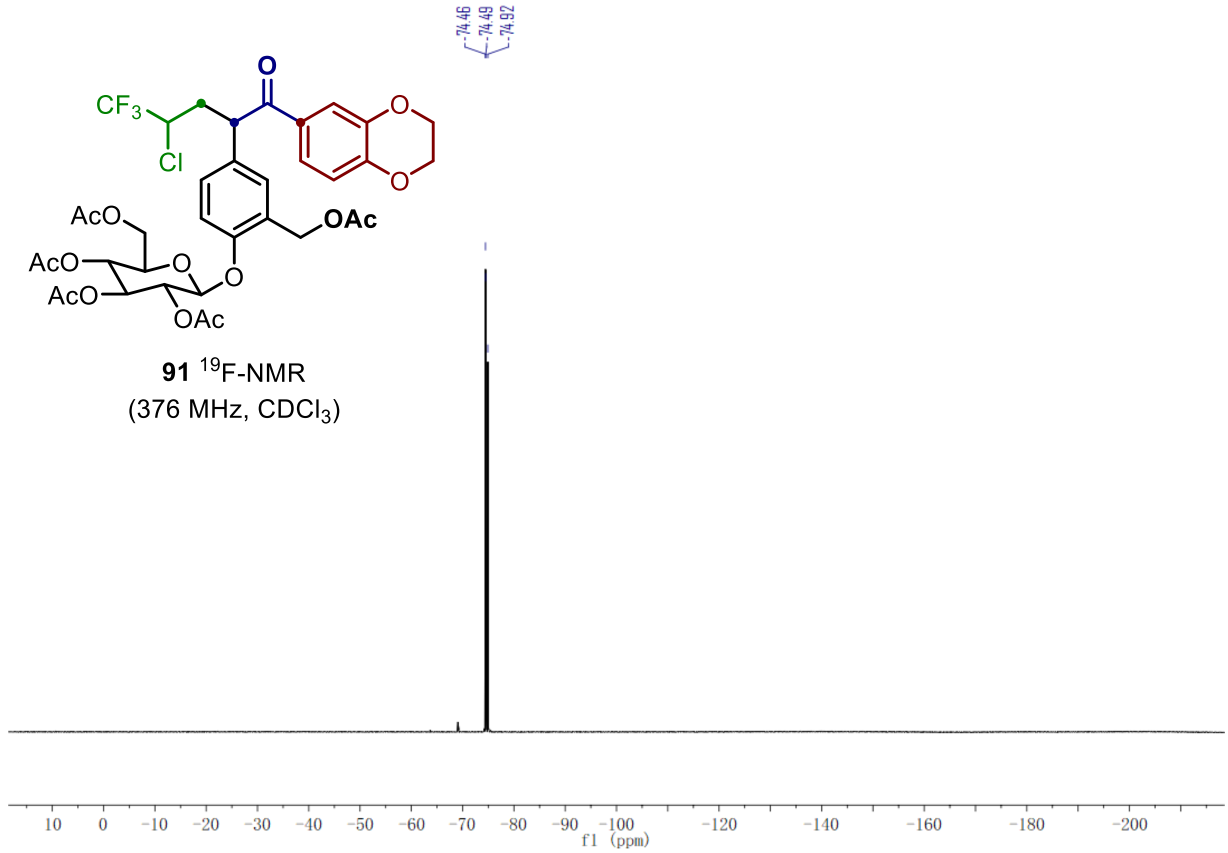

91 ^{19}F-NMR
(376 MHz, $CDCl_3$)

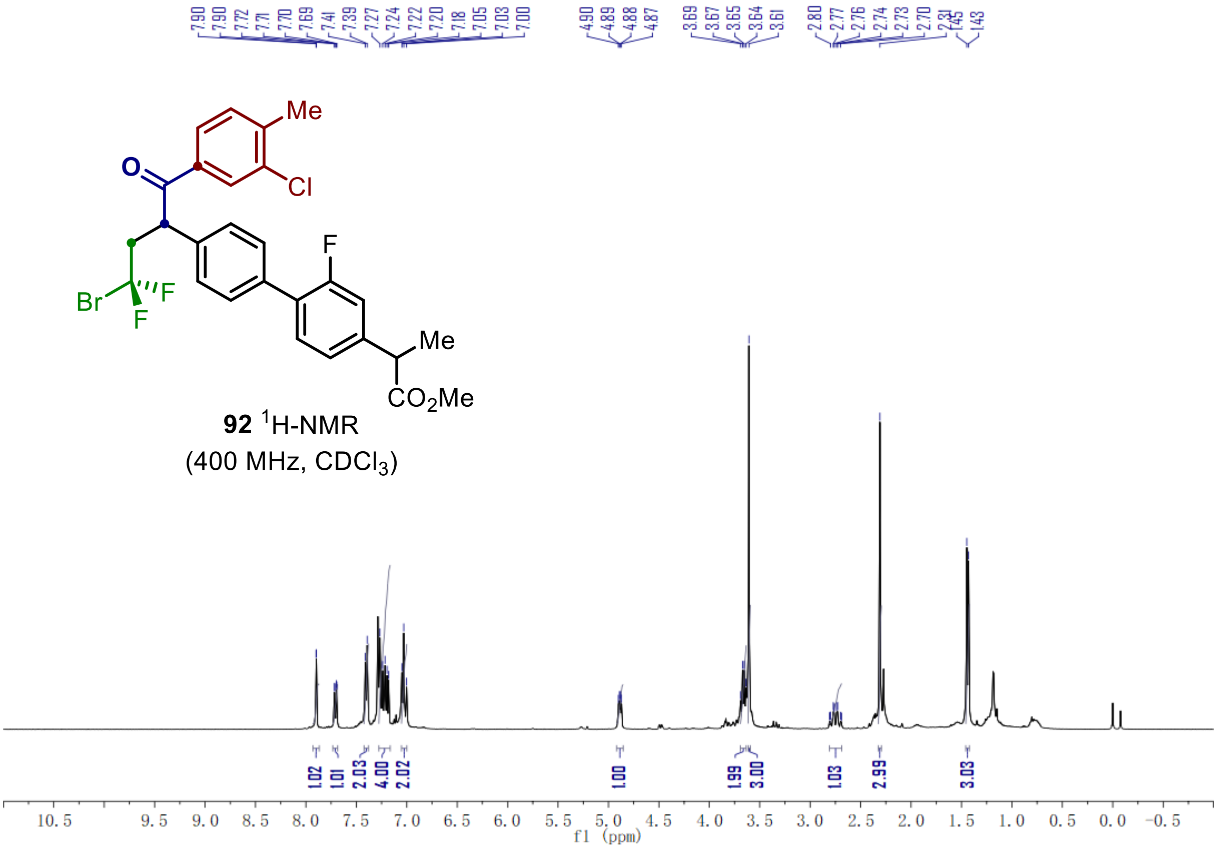
7.90
7.90
7.72
7.71
7.70
7.69
7.41
7.39
7.27
7.24
7.22
7.20
7.18
7.05
7.03
7.00
4.90
4.89
4.88
4.87
3.69
3.67
3.65
3.64
3.61
2.80
2.77
2.76
2.74
2.73
2.70
2.31
1.45
1.43
O
Me
Cl
F
Br
F
F
Me
CO2Me
92 1H-NMR
(400 MHz, CDCl3)
1.02
1.01
2.03
4.00
2.02
1.00
1.99
3.00
1.03
2.99
3.03
10.5 9.5 9.0 8.5 8.0 7.5 7.0 6.5 6.0 5.5 5.0 4.5 4.0 3.5 3.0 2.5 2.0 1.5 1.0 0.5 0.0 -0.5
f1 (ppm)

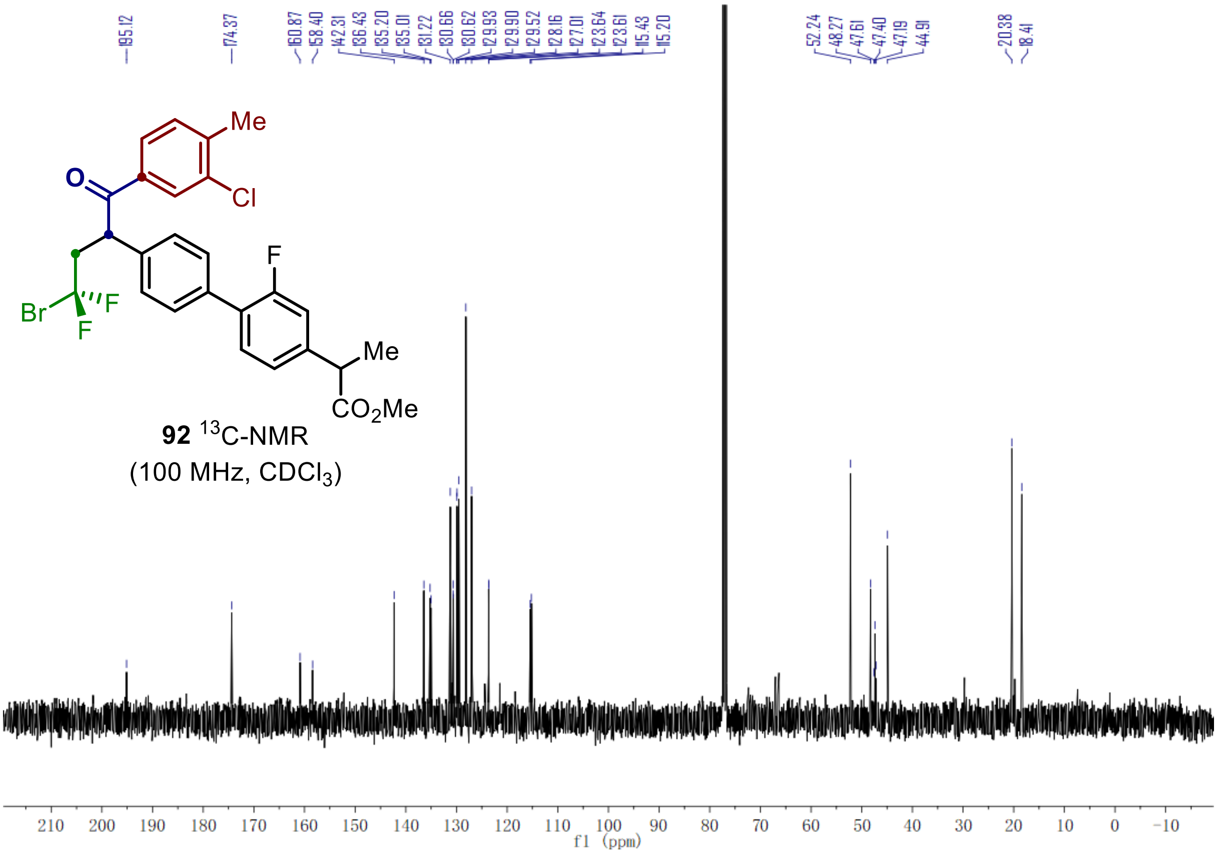
195.12
174.37
160.87
158.40
142.31
136.43
135.20
135.01
131.22
130.66
130.62
129.93
129.90
129.52
128.16
127.01
123.64
123.61
115.43
115.20
52.24
48.27
47.61
47.40
47.19
44.91
20.38
18.41
O
Me
Cl
F
Br
F
F
Me
CO2Me
92 13C-NMR
(100 MHz, CDCl3)
210 200 190 180 170 160 150 140 130 120 110 100 90 80 70 60 50 40 30 20 10 0 -10
f1 (ppm)

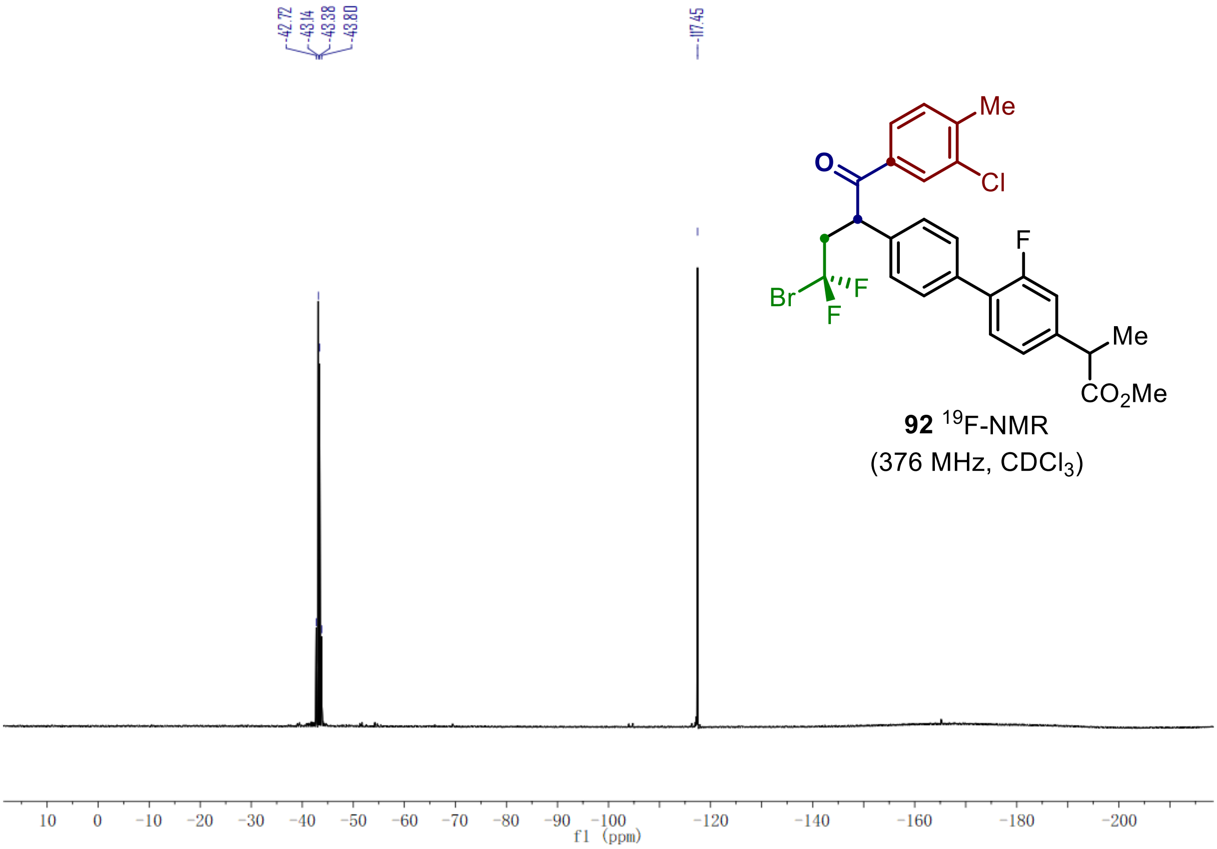
-42.72
-43.14
-43.38
-43.80
-117.45
Me
O
Cl
F
Br
F
F
Me
CO2Me
92 19F-NMR
(376 MHz, CDCl3)
f1 (ppm)

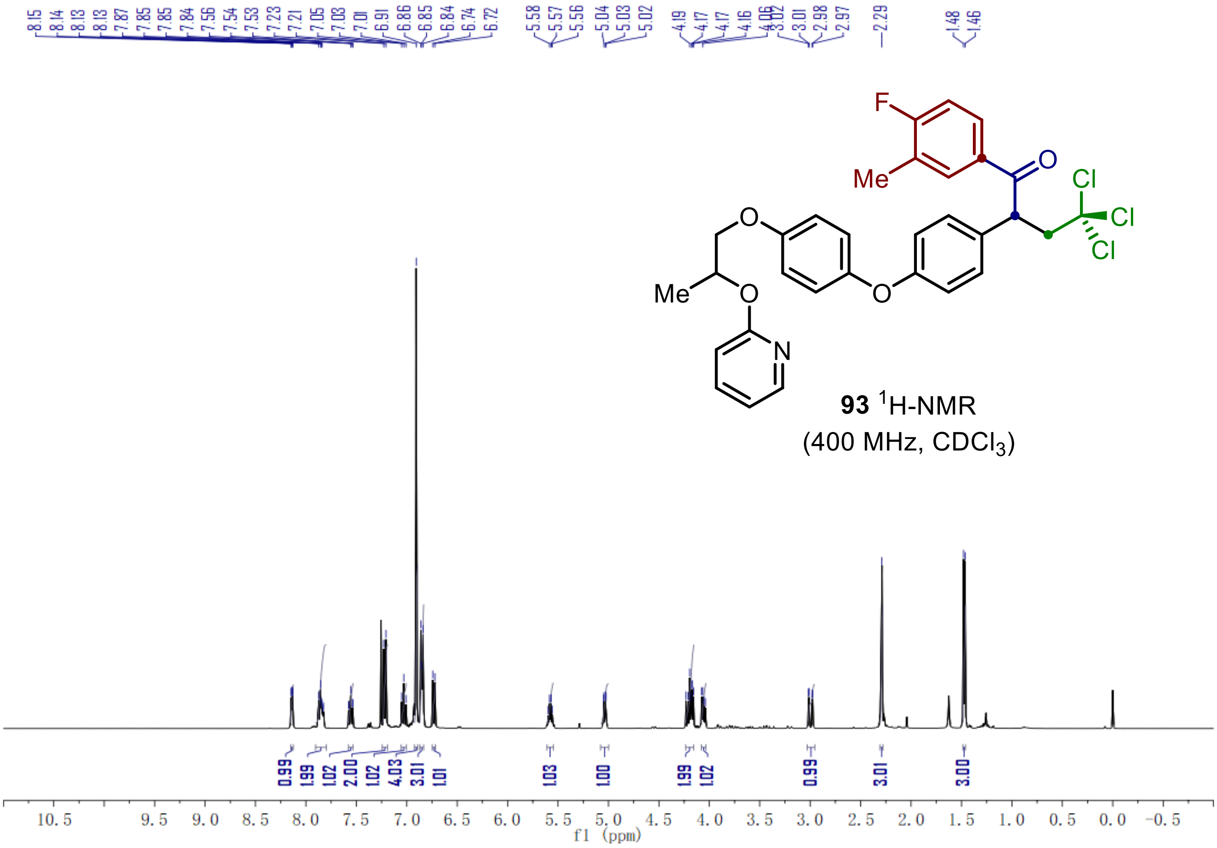
F
Me
O
Cl
Cl
Cl
O
O
Me
O
N
93 1H-NMR
(400 MHz, CDCl3)
0.99
1.99
1.02
2.00
1.02
4.03
3.01
1.01
1.03
1.00
1.99
1.02
0.99
3.01
3.00
f1 (ppm)

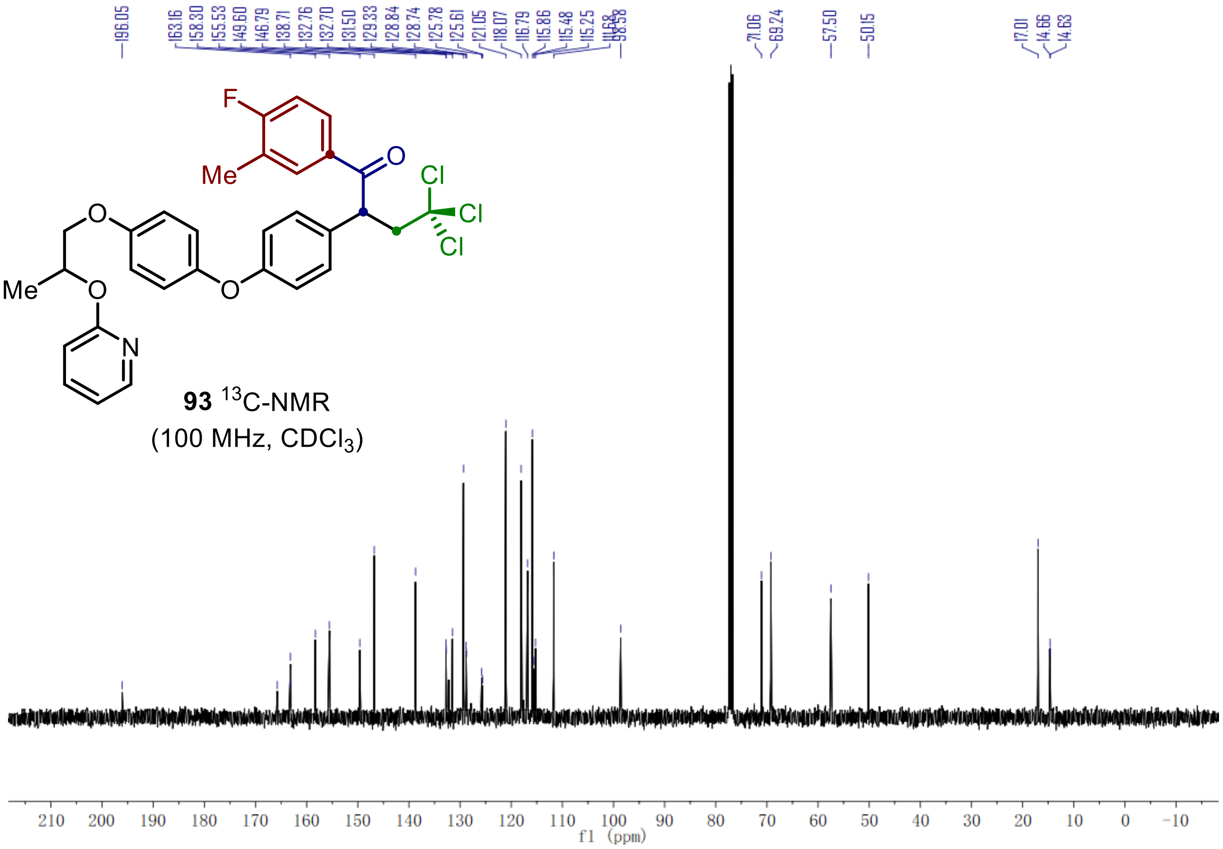

93 ^{13}C-NMR
(100 MHz, $CDCl_3$)

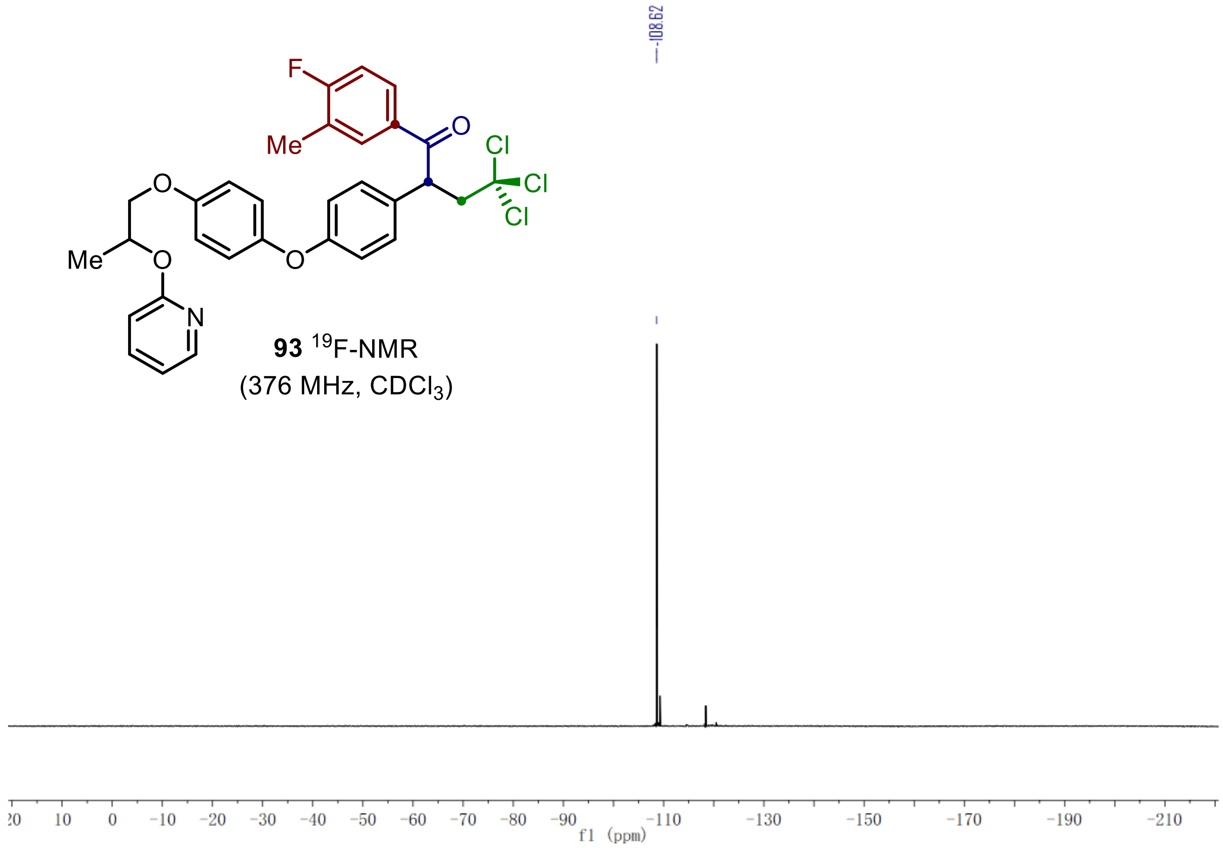

93 ^{19}F-NMR
(376 MHz, $CDCl_3$)

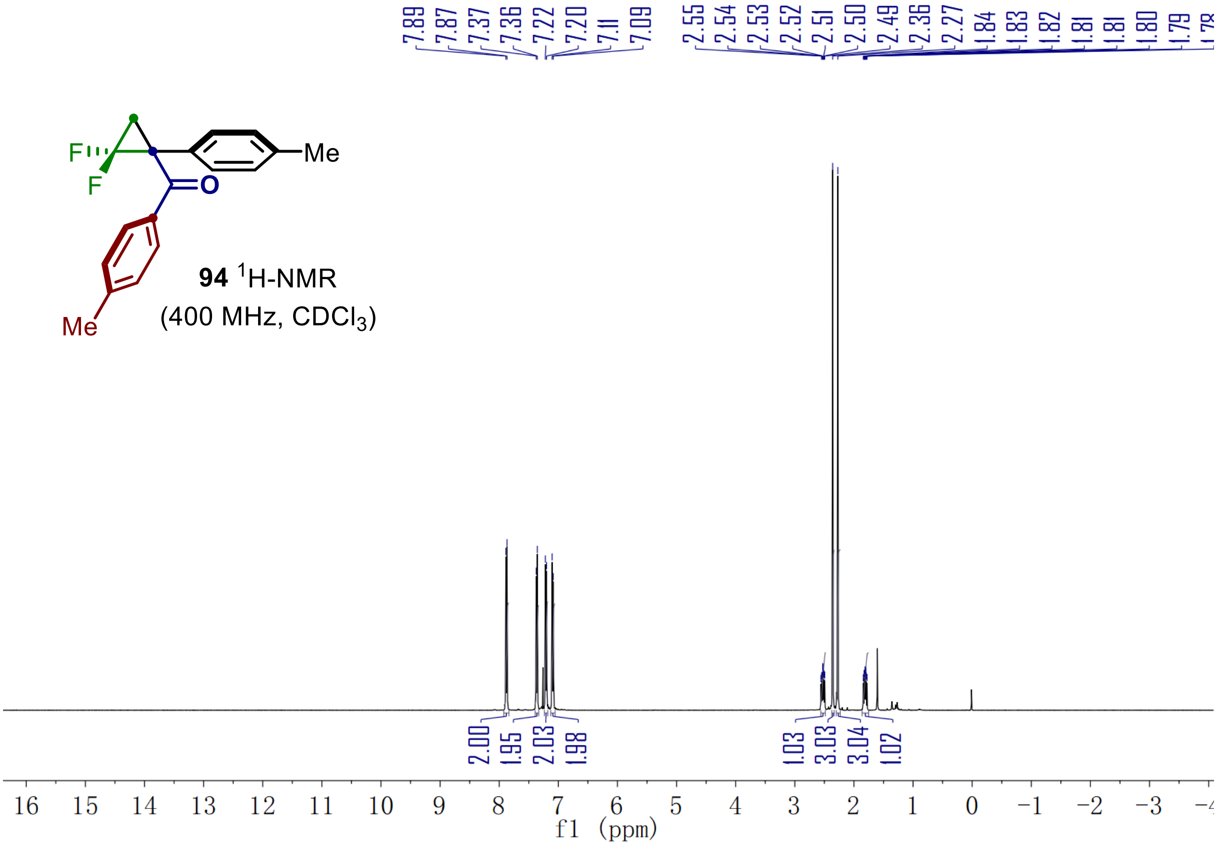
7.89 7.87 7.37 7.36 7.22 7.20 7.11 7.09 2.55 2.54 2.53 2.52 2.51 2.50 2.49 2.36 2.27 1.84 1.83 1.82 1.81 1.81 1.80 1.79
F
F
O
Me
Me
94 1H-NMR
(400 MHz, CDCl3)
2.00 1.95 2.03 1.98 1.03 3.03 3.04 1.02
16 15 14 13 12 11 10 9 8 7 6 5 4 3 2 1 0 −1 −2 −3
f1 (ppm)

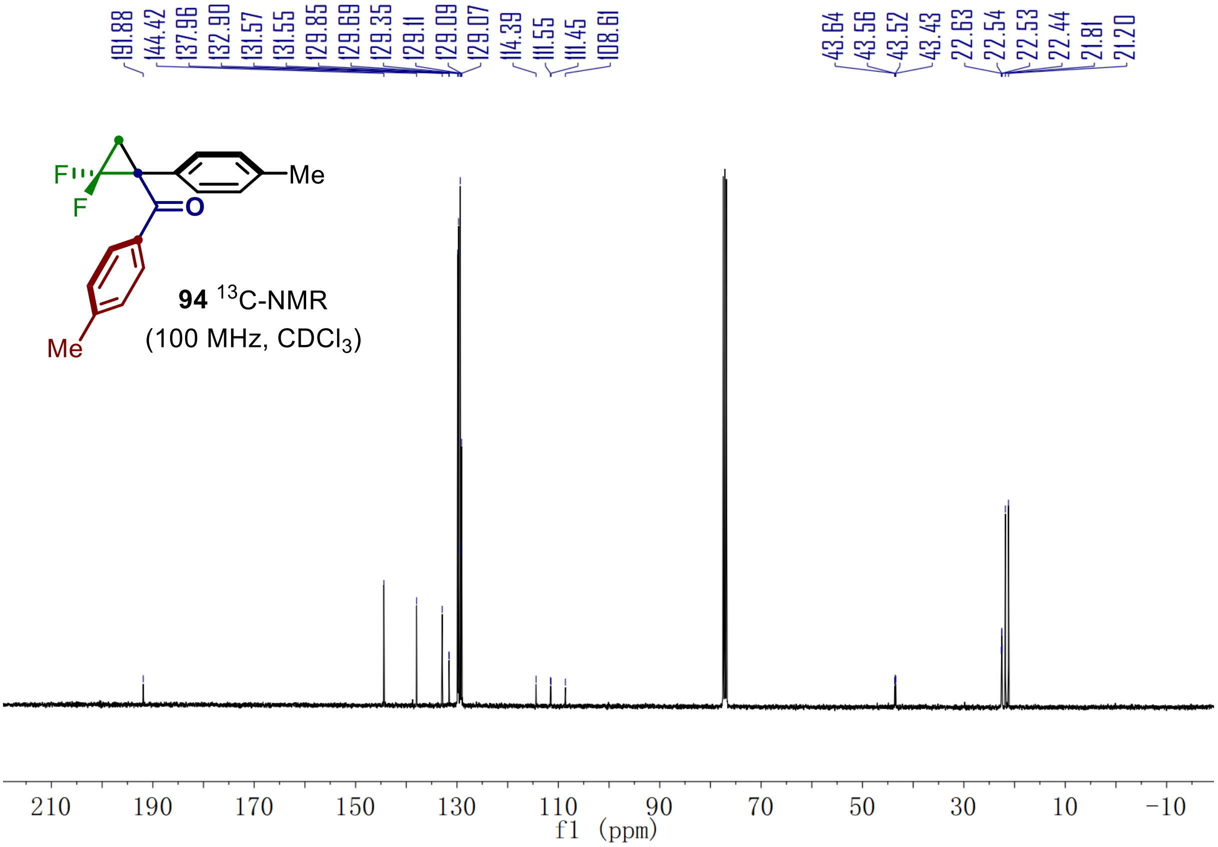
191.88 144.42 137.96 132.90 131.57 131.55 129.85 129.69 129.35 129.11 129.09 129.07 114.39 111.55 111.45 108.61 43.64 43.56 43.52 43.43 22.63 22.54 22.53 22.44 21.81 21.20
F
F
O
Me
Me
94 13C-NMR
(100 MHz, CDCl3)
210 190 170 150 130 110 90 70 50 30 10 −10
f1 (ppm)

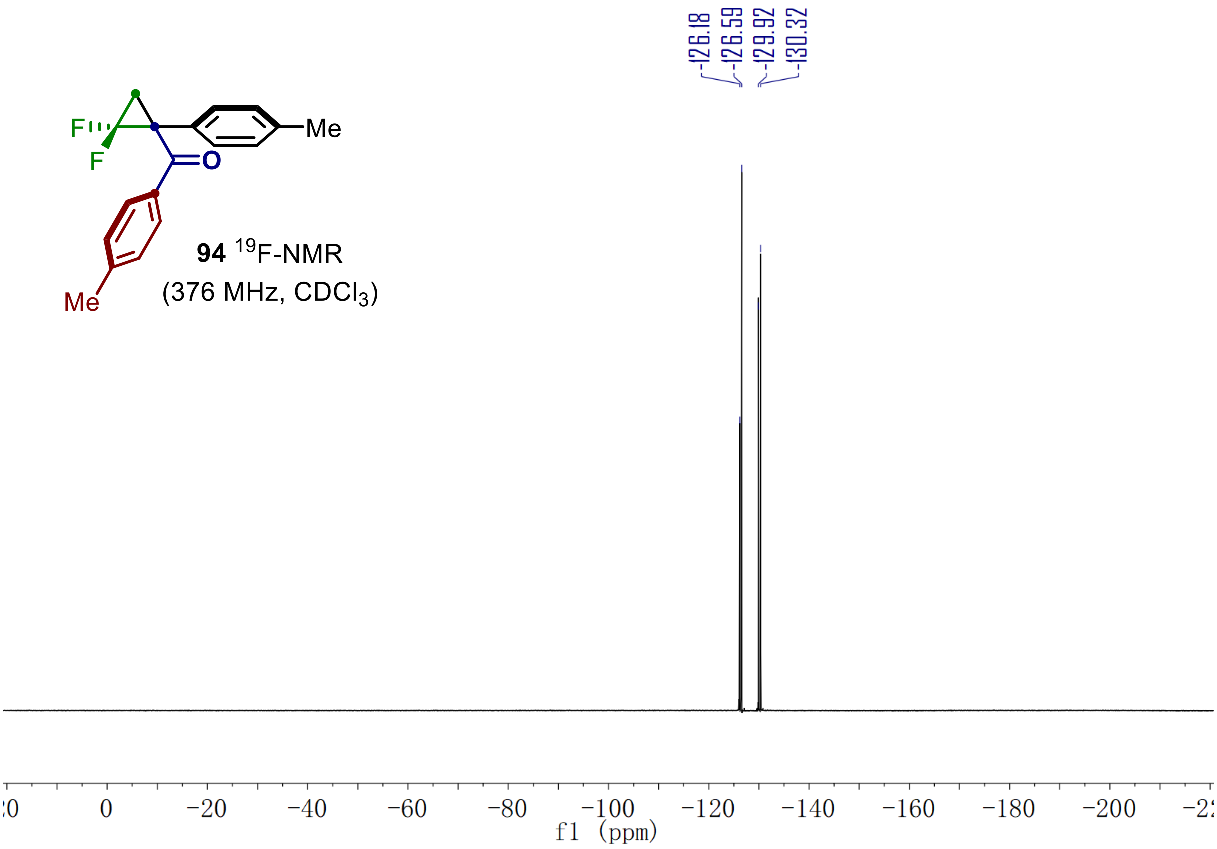
F
F
Me
O
Me
94 19F-NMR
(376 MHz, CDCl3)
-126.18
-126.59
-129.92
-130.32
0
−20
−40
−60
−80
−100
−120
−140
−160
−180
−200
f1 (ppm)

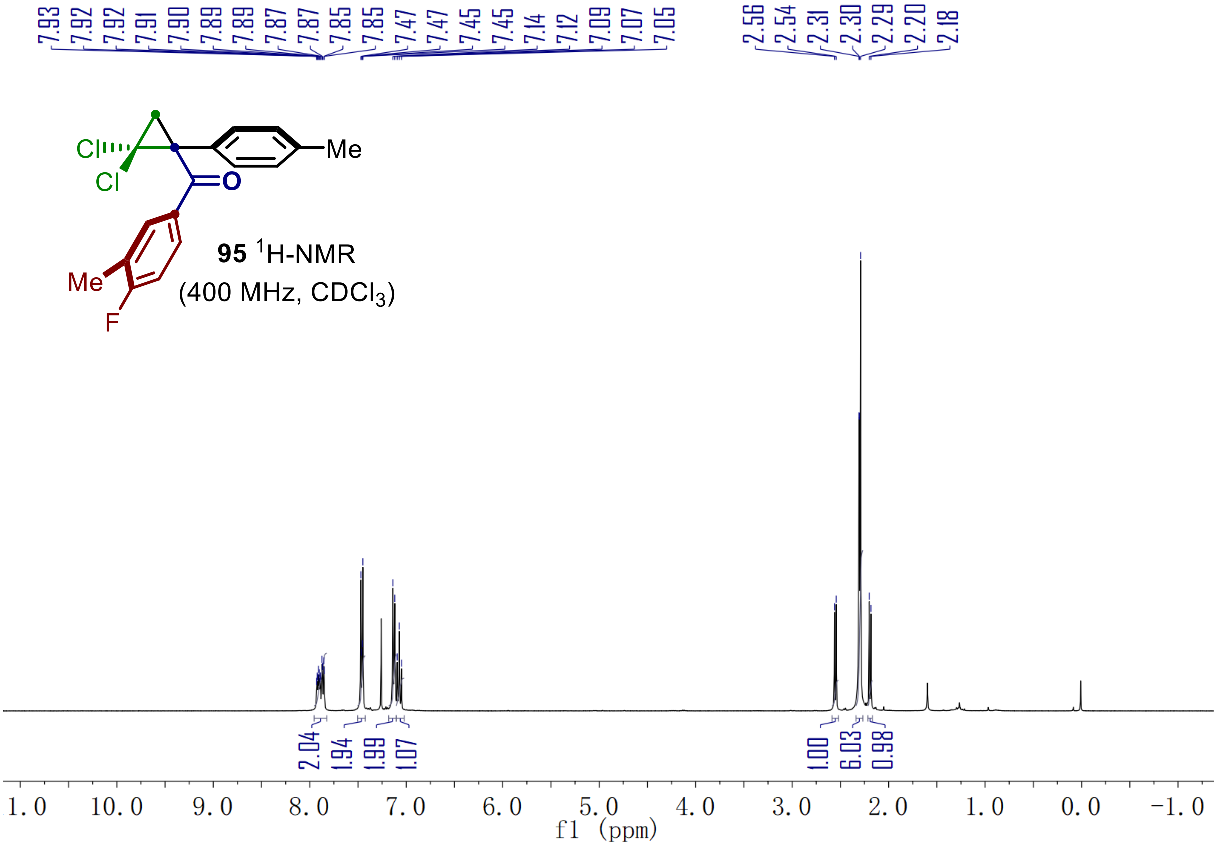
7.93
7.92
7.92
7.91
7.90
7.89
7.89
7.87
7.87
7.85
7.85
7.47
7.47
7.45
7.45
7.14
7.12
7.09
7.07
7.05
2.56
2.54
2.31
2.30
2.29
2.20
2.18
Cl
Cl
Me
O
Me
F
95 1H-NMR
(400 MHz, CDCl3)
2.04
1.94
1.99
1.07
1.00
6.03
0.98
10.0
9.0
8.0
7.0
6.0
5.0
4.0
3.0
2.0
1.0
0.0
−1.0
f1 (ppm)

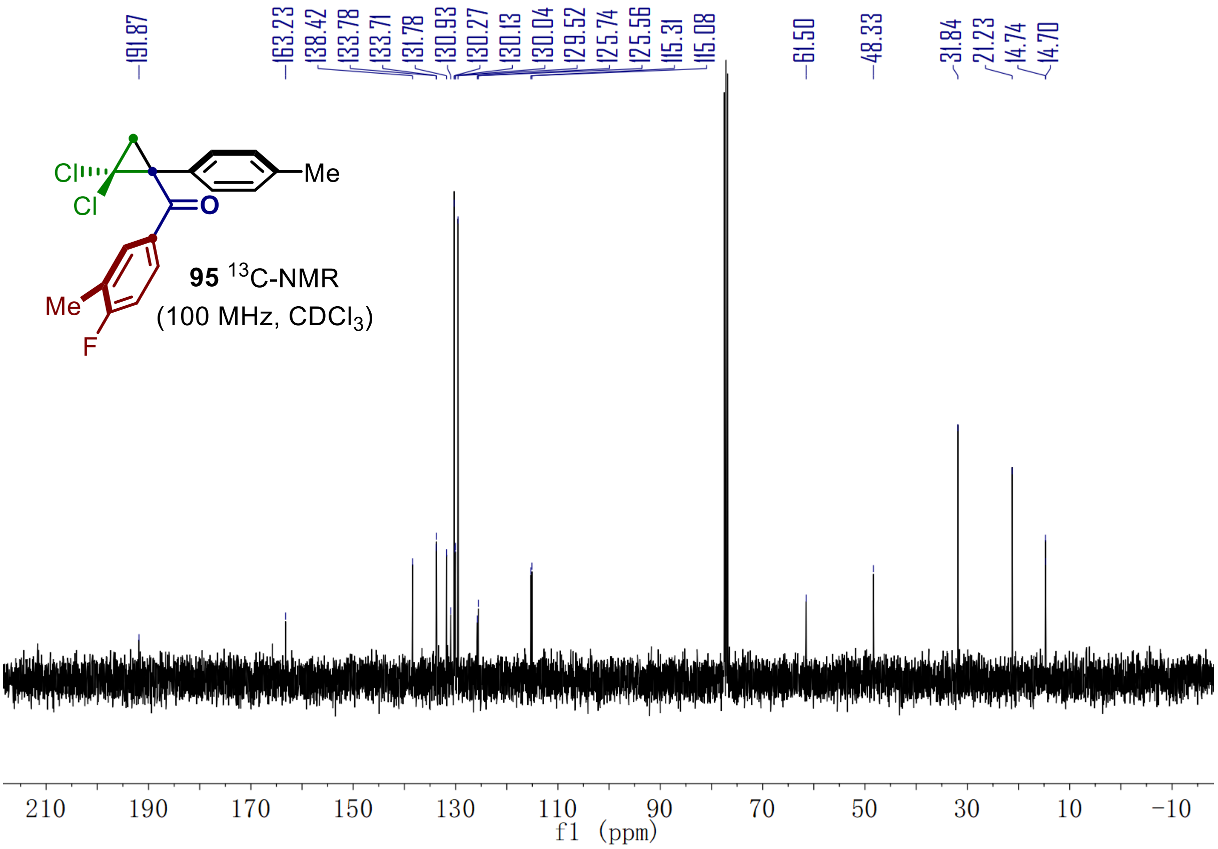
191.87
163.23
138.42
133.78
133.71
131.78
130.93
130.27
130.13
130.04
129.52
125.74
125.56
115.31
115.08
61.50
48.33
31.84
21.23
14.74
14.70
Cl
Cl
O
Me
Me
F
95 13C-NMR
(100 MHz, CDCl3)
210
190
170
150
130
110
90
70
50
30
10
−10
f1 (ppm)

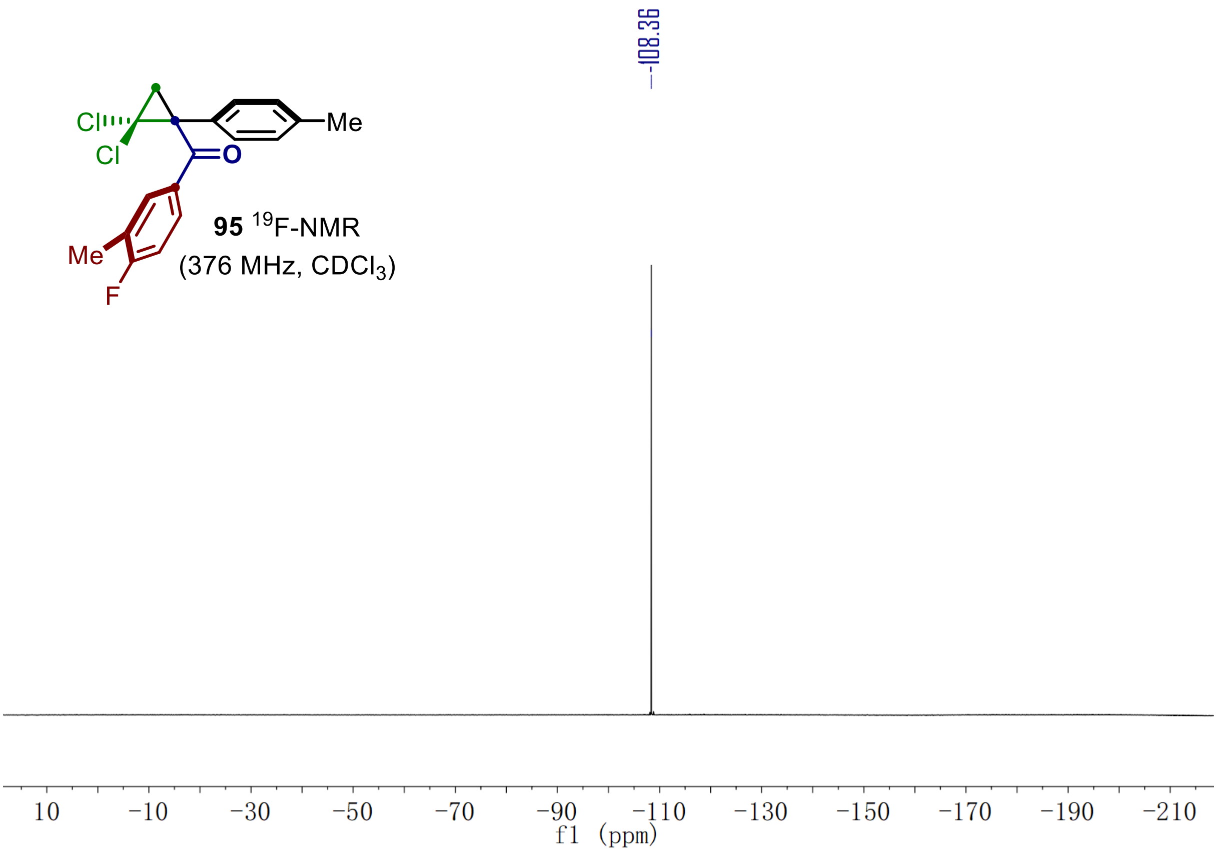
-108.36
Cl
Cl
O
Me
Me
F
95 19F-NMR
(376 MHz, CDCl3)
10
−10
−30
−50
−70
−90
−110
−130
−150
−170
−190
−210
f1 (ppm)

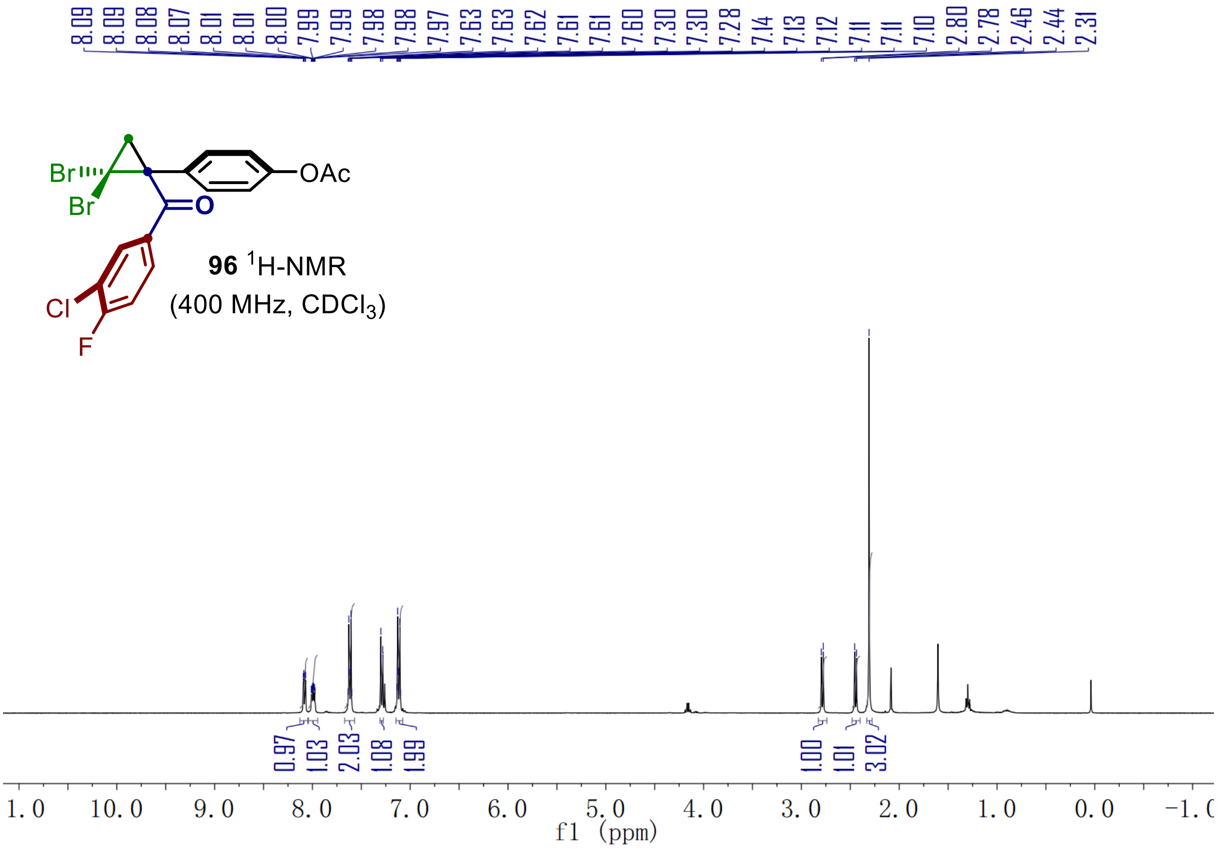
8.09 8.09 8.08 8.07 8.01 8.01 8.00 7.99 7.99 7.98 7.98 7.97 7.63 7.63 7.62 7.61 7.61 7.60 7.30 7.30 7.28 7.14 7.13 7.12 7.11 7.11 7.10 2.80 2.78 2.46 2.44 2.31
Br
Br
OAc
O
Cl
F
96 ^{1}H-NMR
(400 MHz, CDCl$_3$)
0.97 1.03 2.03 1.08 1.99 1.00 1.01 3.02
f1 (ppm)

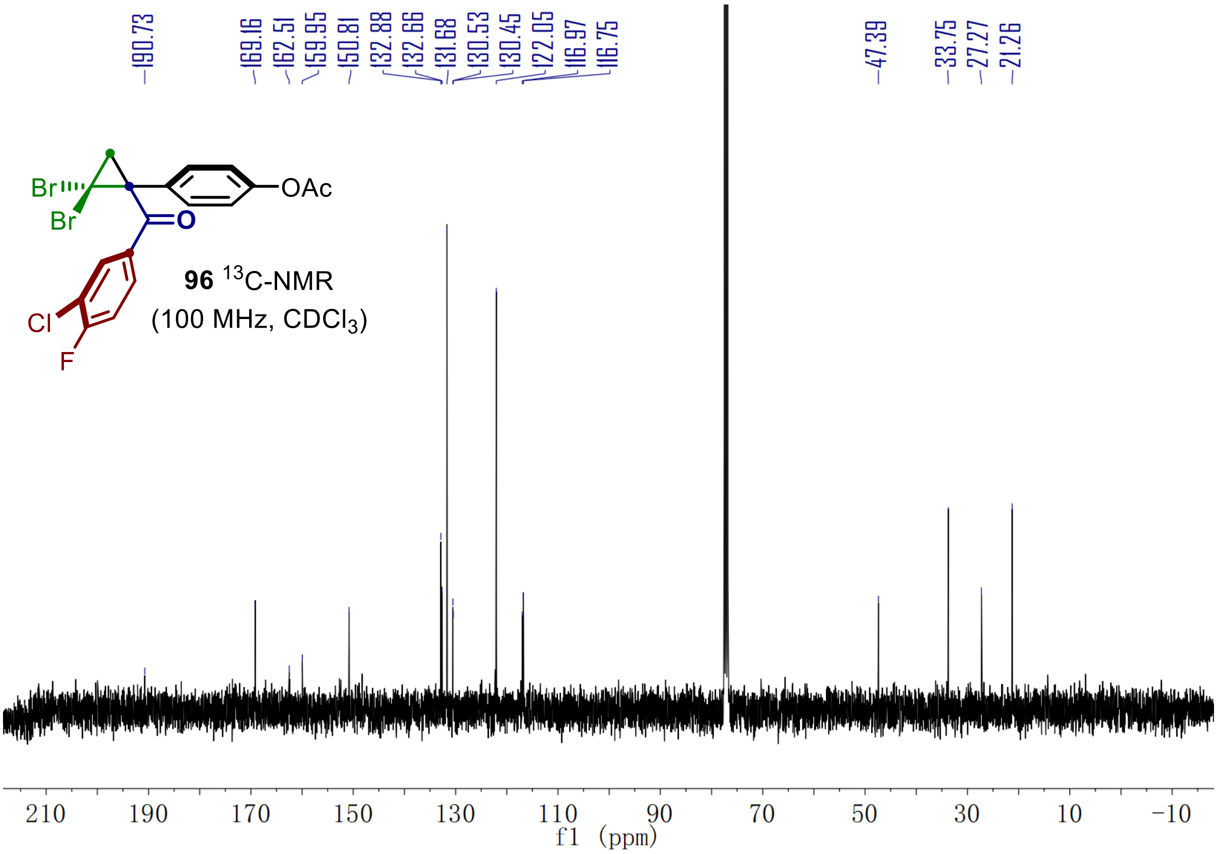
190.73 169.16 162.51 159.95 150.81 132.88 132.66 131.68 130.53 130.45 122.05 116.97 116.75 47.39 33.75 27.27 21.26
Br
Br
OAc
O
Cl
F
96 ^{13}C-NMR
(100 MHz, CDCl$_3$)
f1 (ppm)

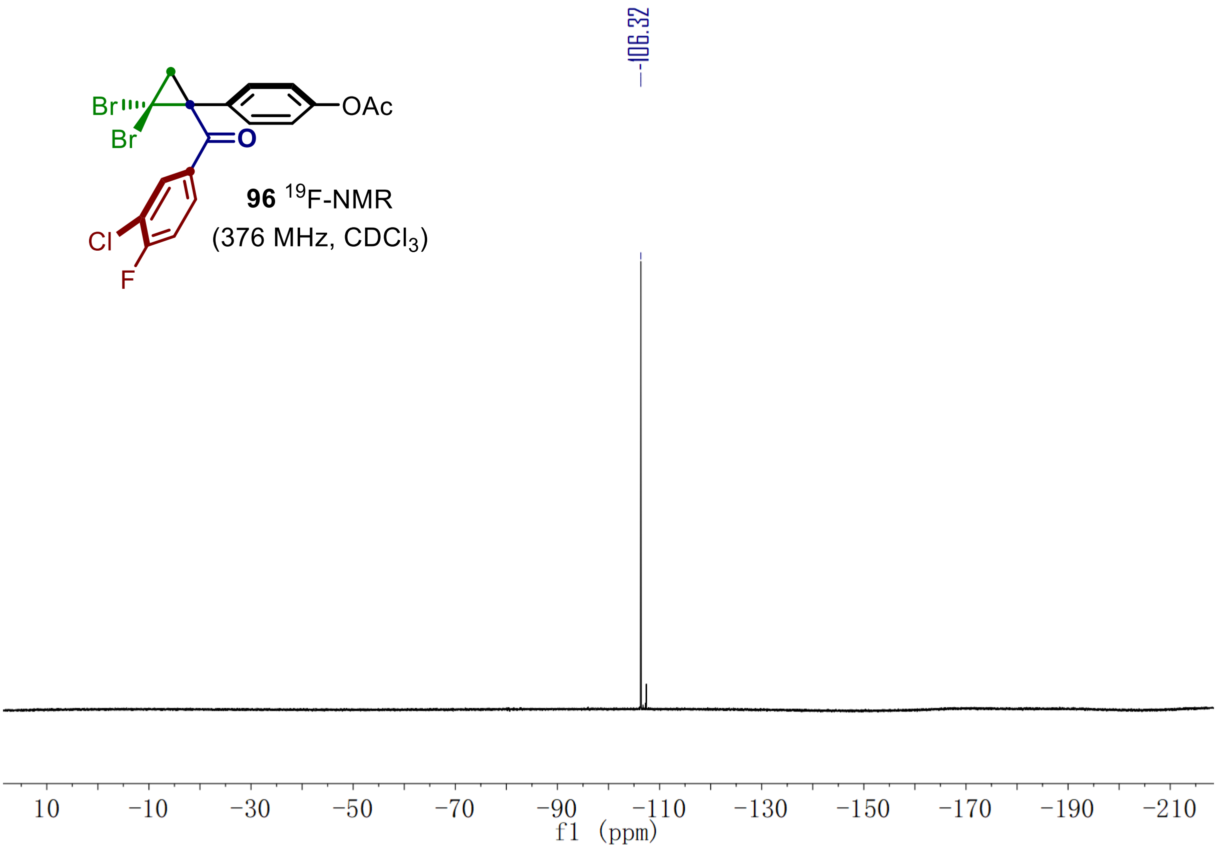
Br
Br
OAc
O
Cl
F
96 19F-NMR
(376 MHz, CDCl3)
-106.32
10
-10
-30
-50
-70
-90
-110
-130
-150
-170
-190
-210
f1 (ppm)

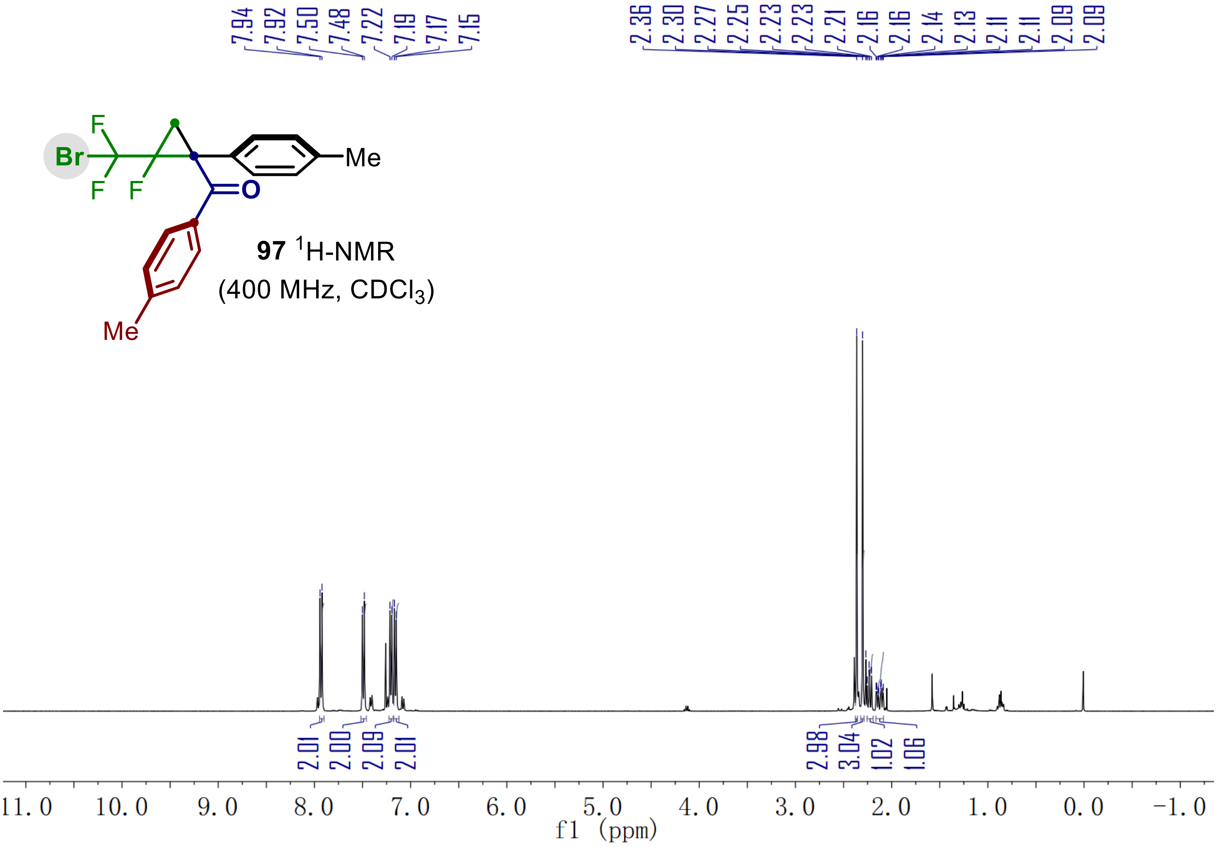
7.94
7.92
7.50
7.48
7.22
7.19
7.17
7.15
2.36
2.30
2.27
2.25
2.23
2.23
2.21
2.16
2.16
2.14
2.13
2.11
2.11
2.09
2.09
F
Br
F
F
Me
O
Me
97 1H-NMR
(400 MHz, CDCl3)
2.01
2.00
2.09
2.01
2.98
3.04
1.02
1.06
11.0
10.0
9.0
8.0
7.0
6.0
5.0
4.0
3.0
2.0
1.0
0.0
-1.0
f1 (ppm)

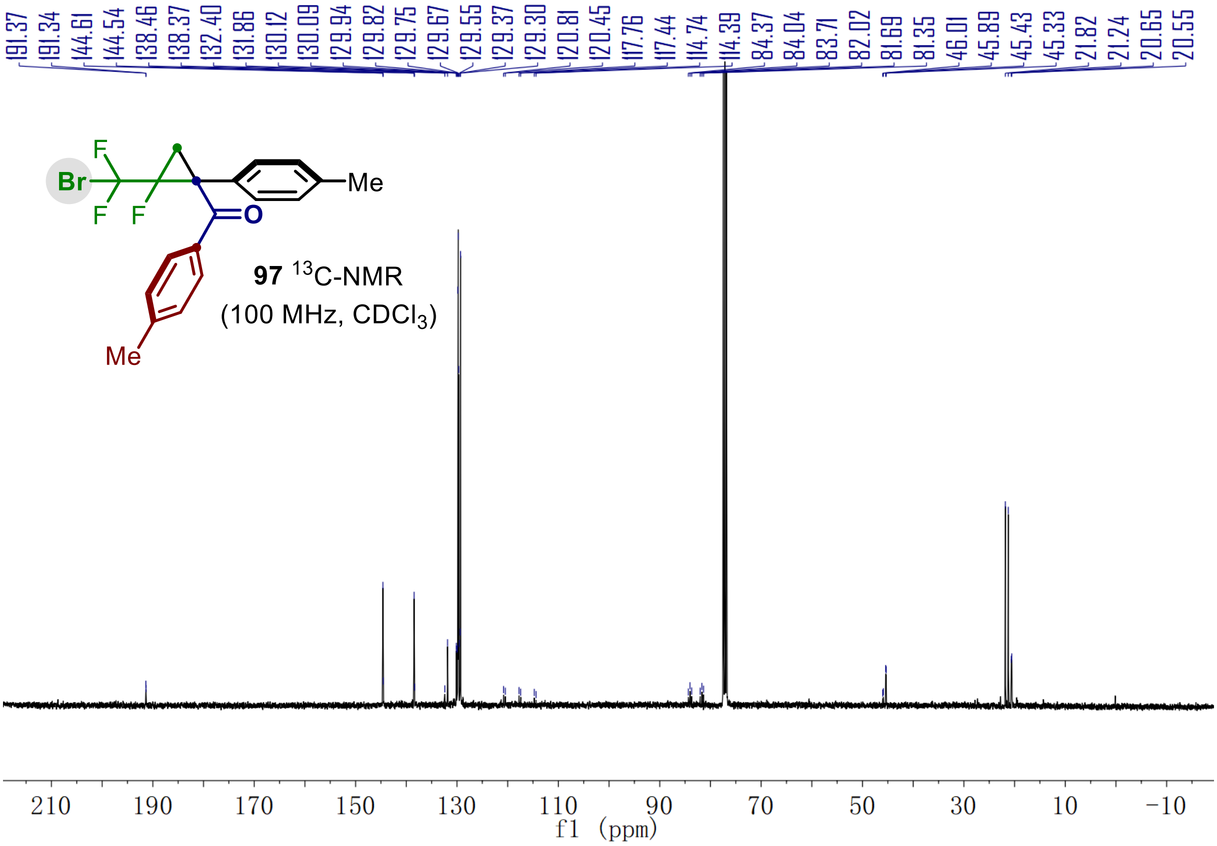
191.37
191.34
144.61
144.54
138.46
138.37
132.40
131.86
130.12
130.09
129.94
129.82
129.75
129.67
129.55
129.37
129.30
120.81
120.45
117.76
117.44
114.74
114.39
84.37
84.04
83.71
82.02
81.69
81.35
46.01
45.89
45.43
45.33
21.82
21.24
20.65
20.55
Br
F
F
F
O
Me
Me
97 13C-NMR
(100 MHz, CDCl3)
f1 (ppm)

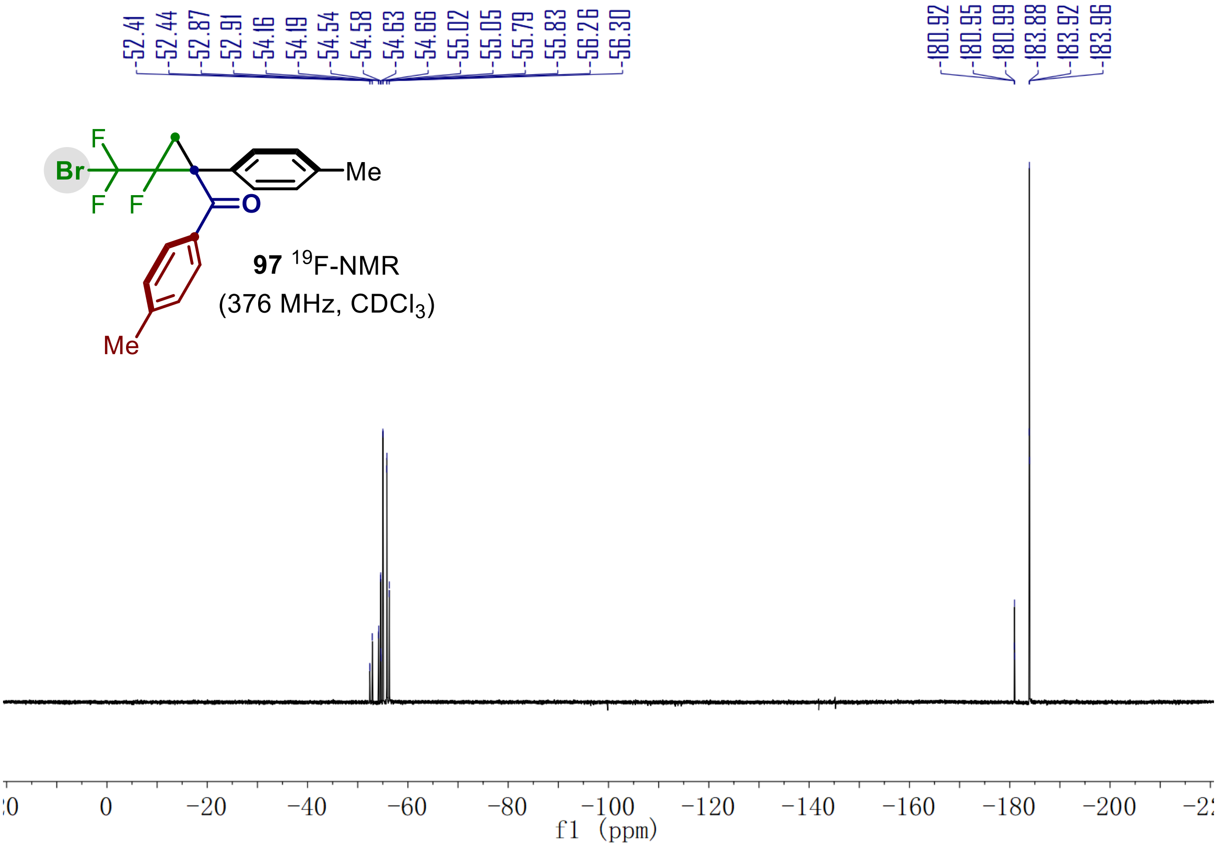
-52.41
-52.44
-52.87
-52.91
-54.16
-54.19
-54.54
-54.58
-54.63
-54.66
-55.02
-55.05
-55.79
-55.83
-56.26
-56.30
-180.92
-180.95
-180.99
-183.88
-183.92
-183.96
Br
F
F
F
O
Me
Me
97 19F-NMR
(376 MHz, CDCl3)
f1 (ppm)

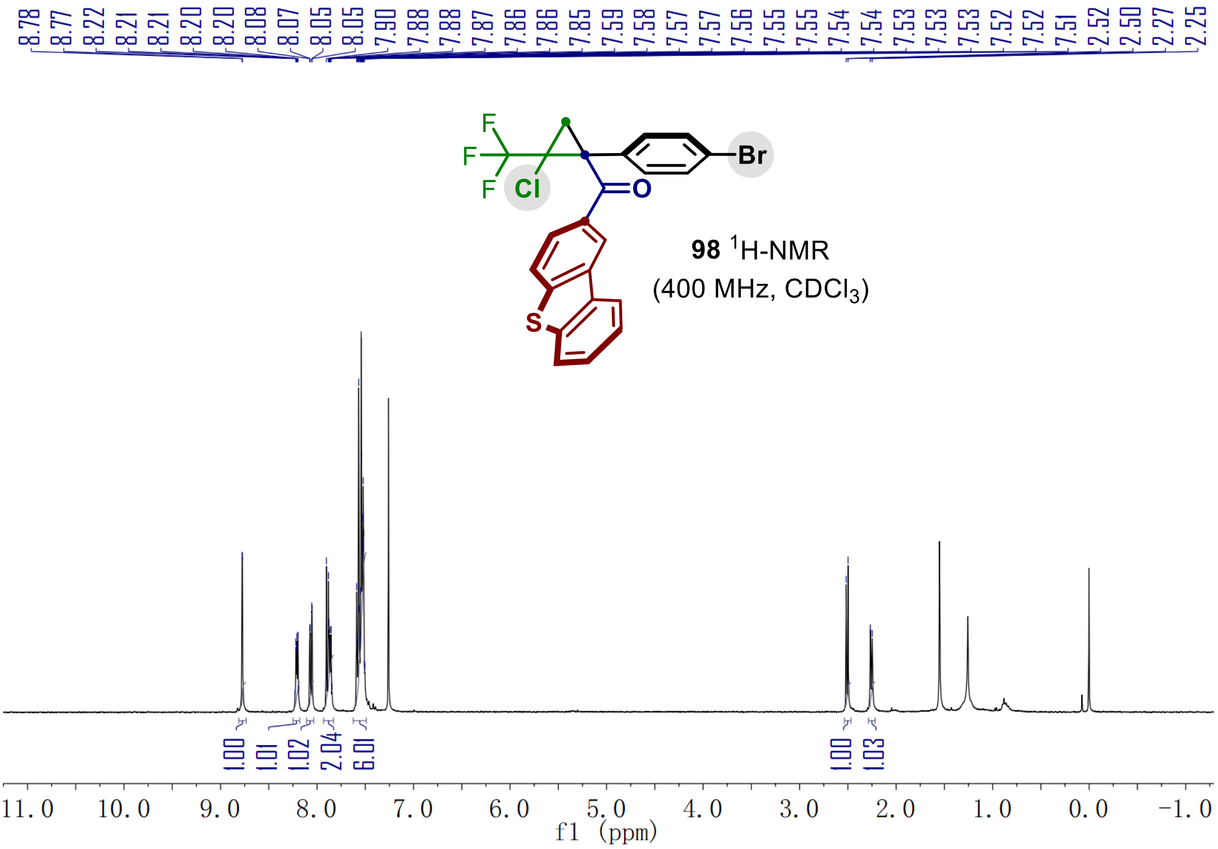

98 ¹H-NMR (400 MHz, $CDCl_3$)

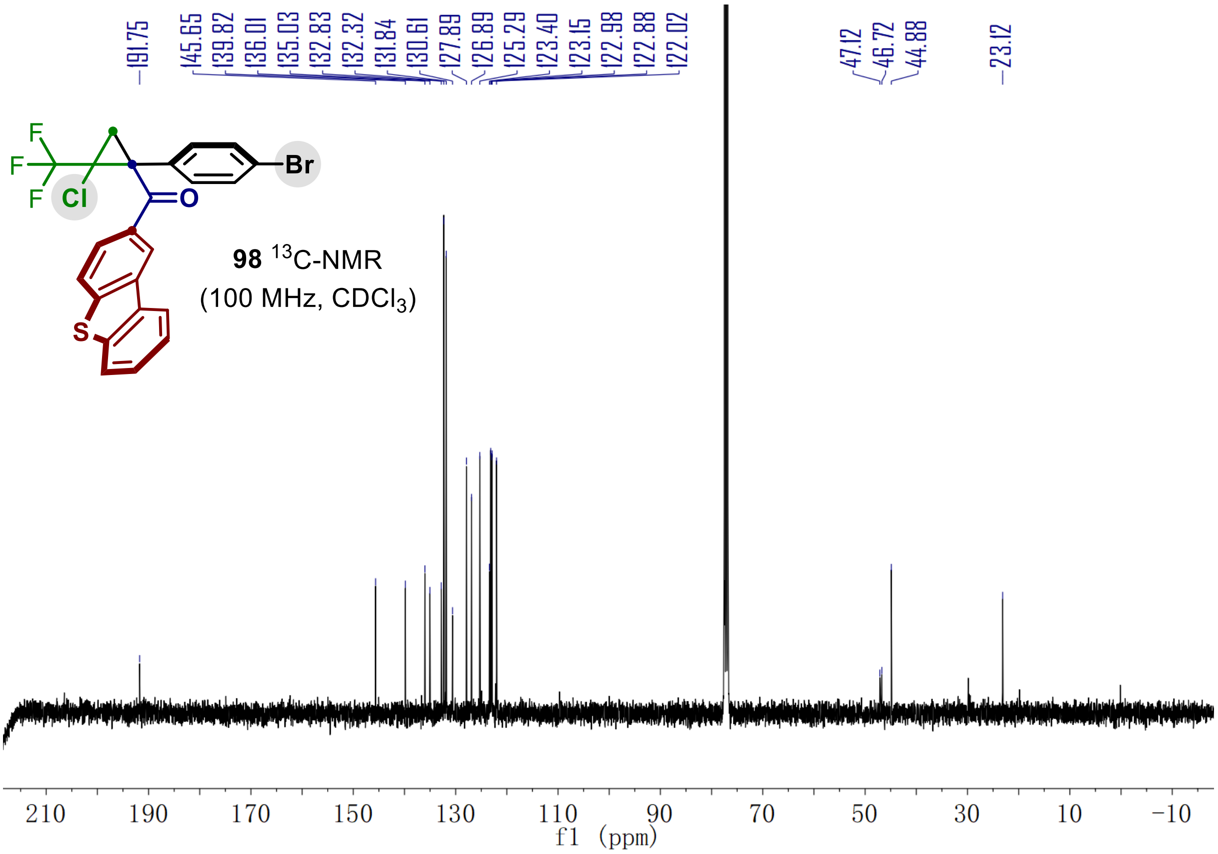

98 ¹³C-NMR (100 MHz, $CDCl_3$)

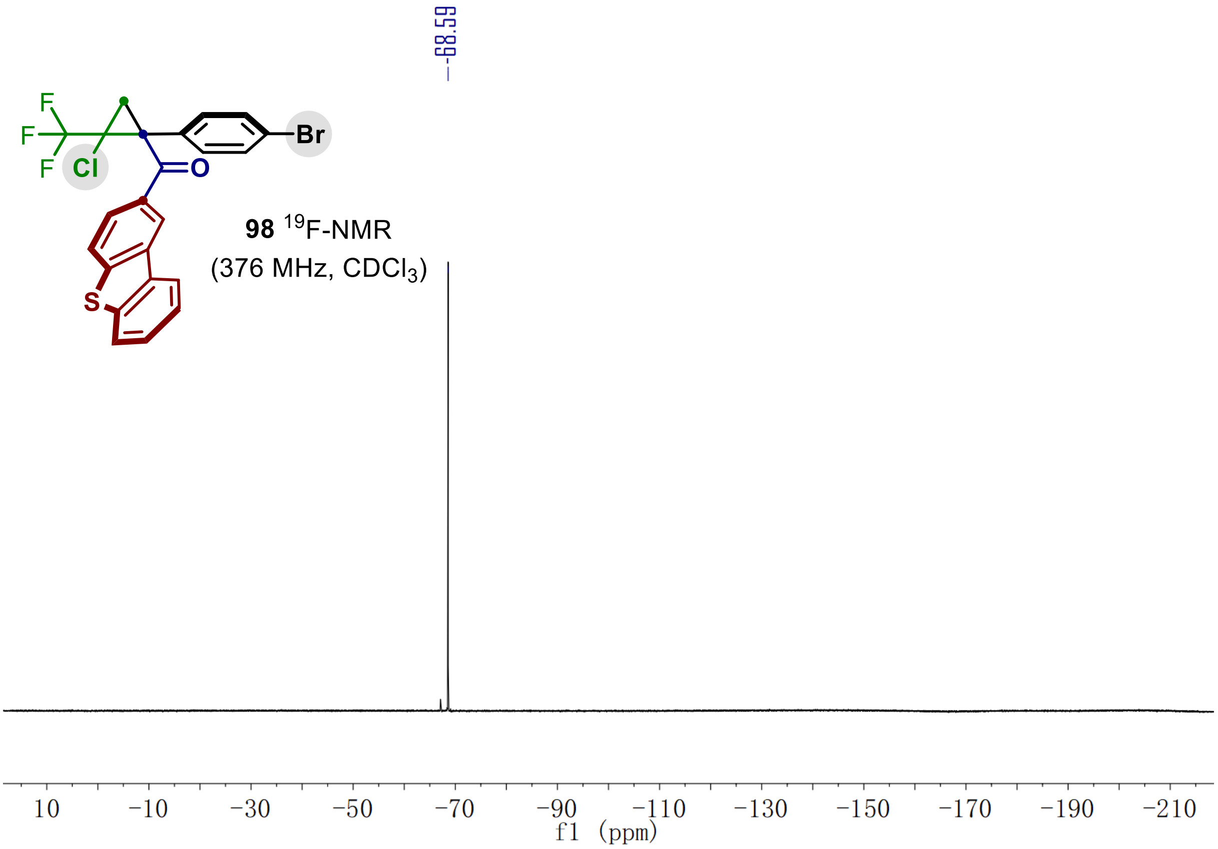
-68.59
F
F
F
Cl
Br
O
S
98 ^{19}F-NMR
(376 MHz, CDCl$_3$)
10 -10 -30 -50 -70 -90 -110 -130 -150 -170 -190 -210
f1 (ppm)

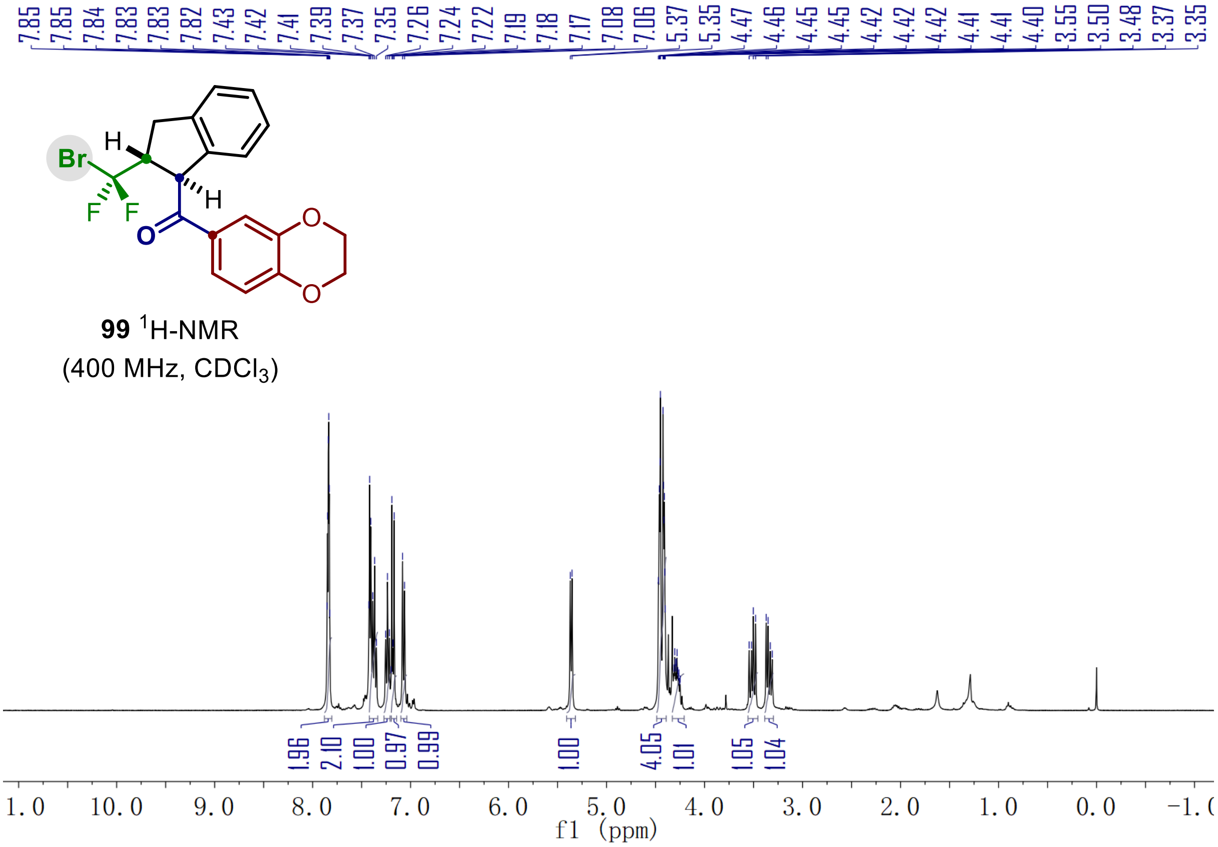
7.85 7.85 7.84 7.83 7.83 7.82 7.43 7.42 7.41 7.39 7.37 7.35 7.26 7.24 7.22 7.19 7.18 7.17 7.08 7.06 5.37 5.35 4.47 4.46 4.45 4.45 4.42 4.42 4.42 4.41 4.41 4.40 3.55 3.50 3.48 3.37 3.35
H
Br
F
F
H
O
O
O
99 ^{1}H-NMR
(400 MHz, CDCl$_3$)
1.96 2.10 1.00 0.97 0.99 1.00 4.05 1.01 1.05 1.04
1.0 10.0 9.0 8.0 7.0 6.0 5.0 4.0 3.0 2.0 1.0 0.0 -1.0
f1 (ppm)

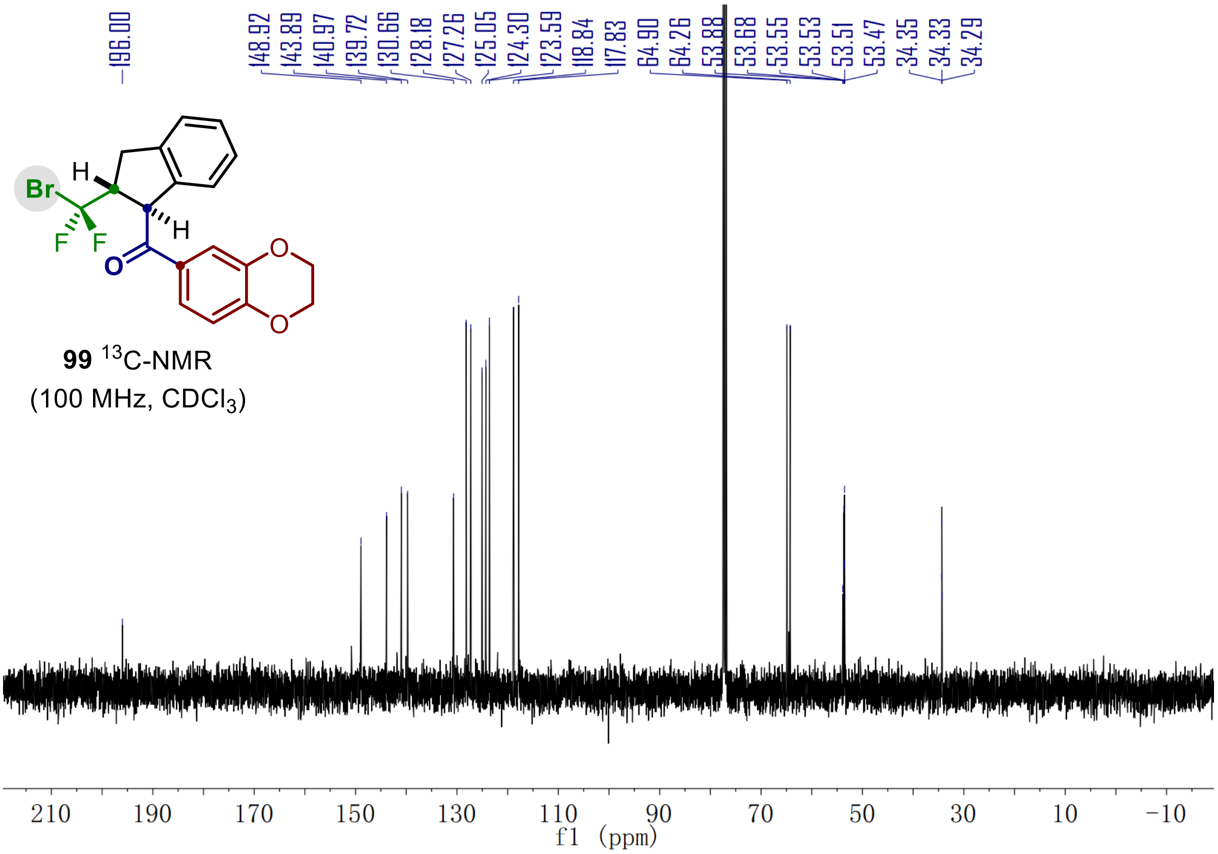
196.00
148.92
143.89
140.97
139.72
130.66
128.18
127.26
125.05
124.30
123.59
118.84
117.83
64.90
64.26
53.88
53.68
53.55
53.53
53.51
53.47
34.35
34.33
34.29
Br
H
F
F
H
O
O
O
99 ^{13}C-NMR
(100 MHz, $CDCl_3$)
210 190 170 150 130 110 90 70 50 30 10 −10
f1 (ppm)

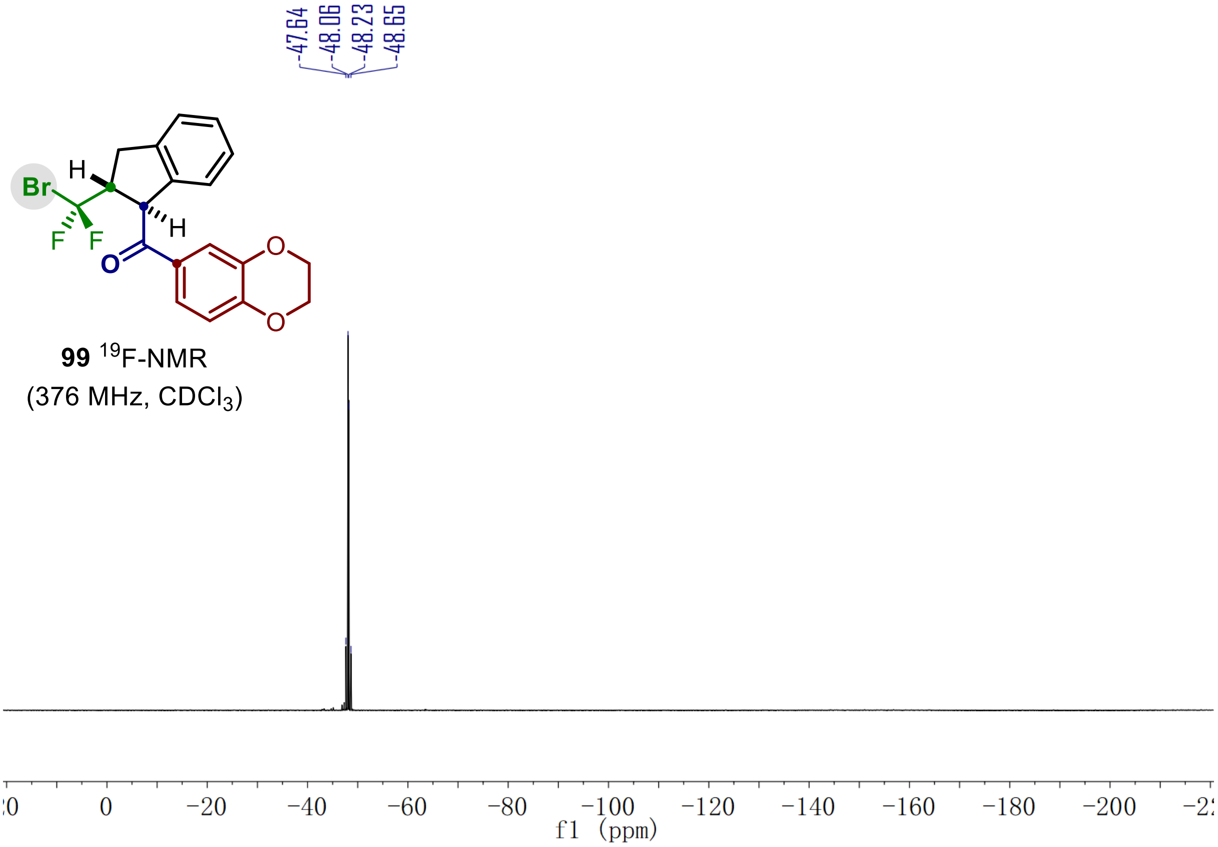
-47.64
-48.06
-48.23
-48.65
Br
H
F
F
H
O
O
O
99 ^{19}F-NMR
(376 MHz, $CDCl_3$)
0 −20 −40 −60 −80 −100 −120 −140 −160 −180 −200
f1 (ppm)

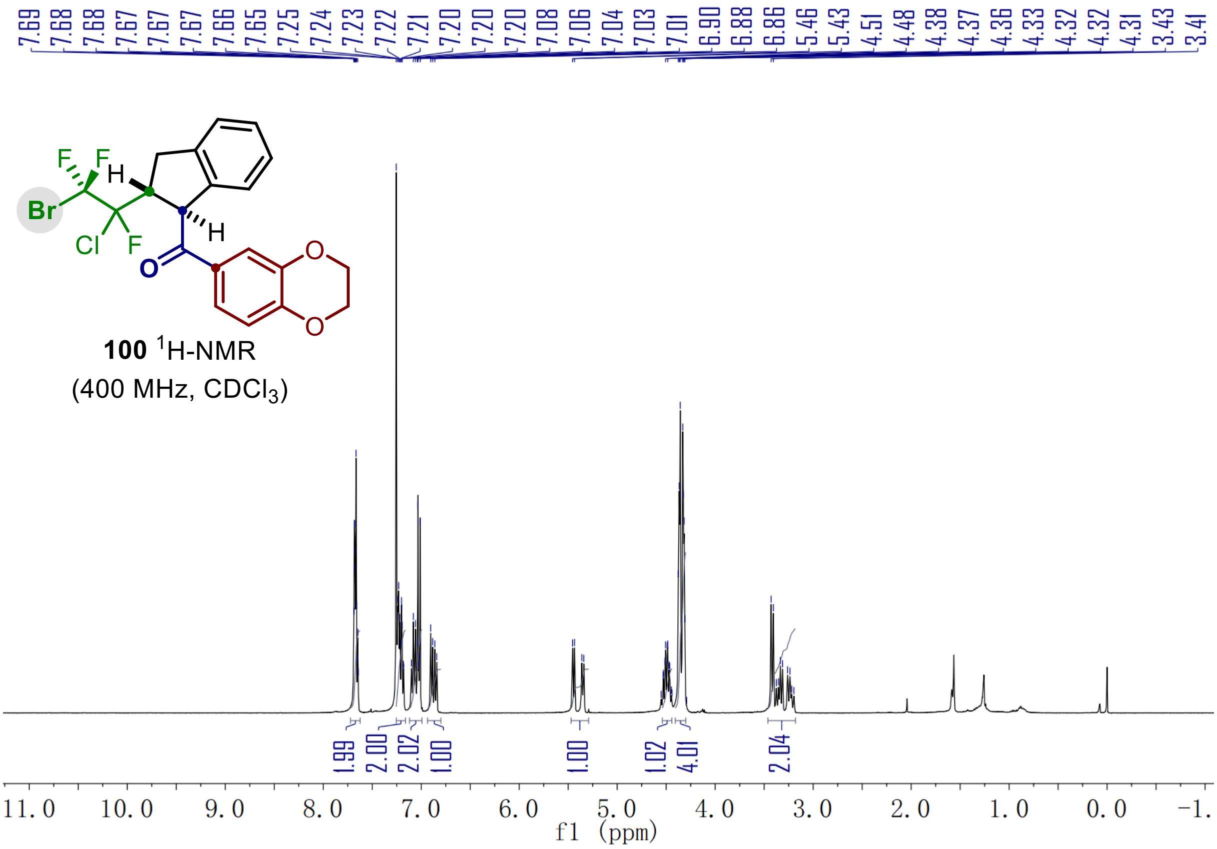
7.69 7.68 7.68 7.67 7.67 7.67 7.66 7.65 7.25 7.24 7.23 7.22 7.21 7.20 7.20 7.20 7.08 7.06 7.04 7.03 7.01 6.90 6.88 6.86 5.46 5.43 4.51 4.48 4.38 4.37 4.36 4.33 4.32 4.32 4.31 3.43 3.41
F F H Br Cl F O H O O
100 1H-NMR
(400 MHz, CDCl3)
1.99 2.00 2.02 1.00 1.00 1.02 4.01 2.04
11.0 10.0 9.0 8.0 7.0 6.0 5.0 4.0 3.0 2.0 1.0 0.0 -1.
f1 (ppm)

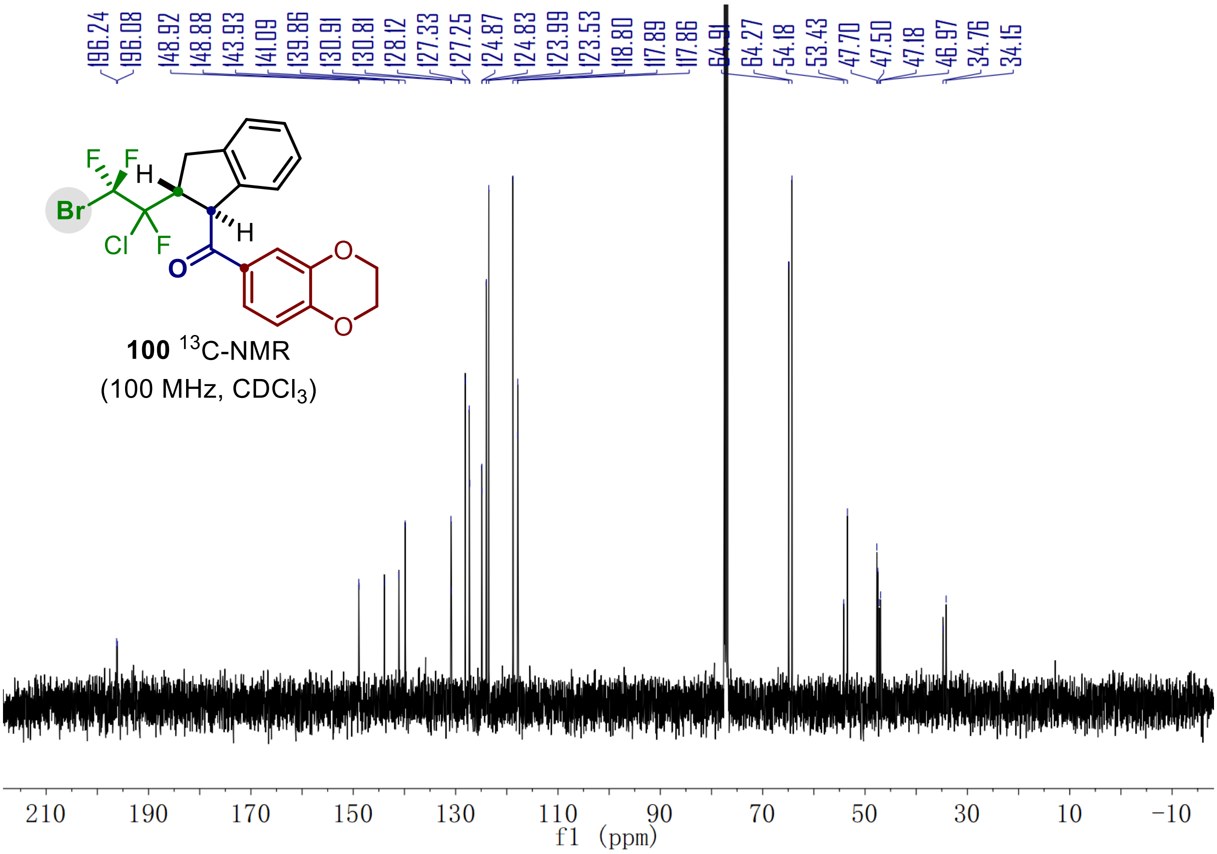
196.24 196.08 148.92 148.88 143.93 141.09 139.86 130.91 130.81 128.12 127.33 127.25 124.87 124.83 123.99 123.53 118.80 117.89 117.86 64.91 64.27 54.18 53.43 47.70 47.50 47.18 46.97 34.76 34.15
F F H Br Cl F O H O O
100 13C-NMR
(100 MHz, CDCl3)
210 190 170 150 130 110 90 70 50 30 10 -10
f1 (ppm)

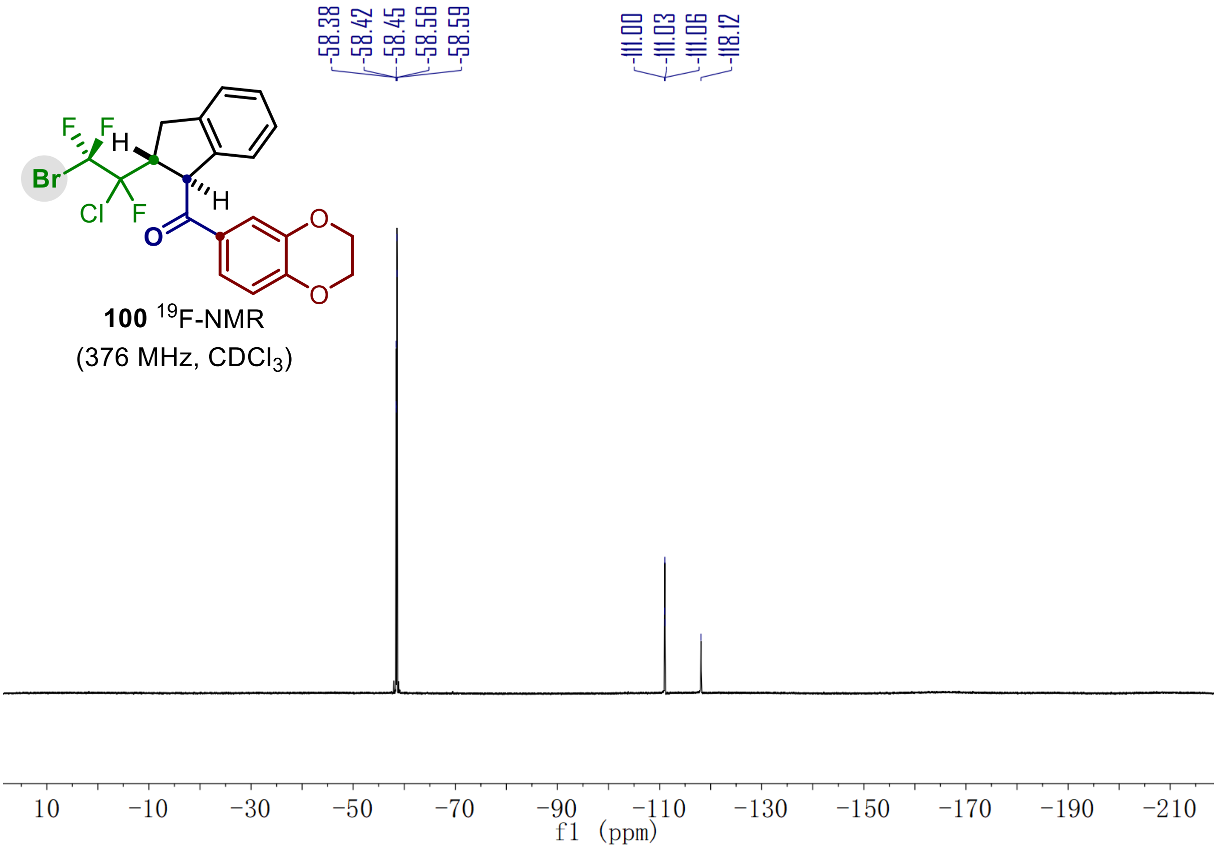
-58.38
-58.42
-58.45
-58.56
-58.59
-111.00
-111.03
-111.06
-118.12
Br
F
F
H
Cl
F
H
O
O
O
100 ^{19}F-NMR
(376 MHz, $CDCl_3$)
f1 (ppm)

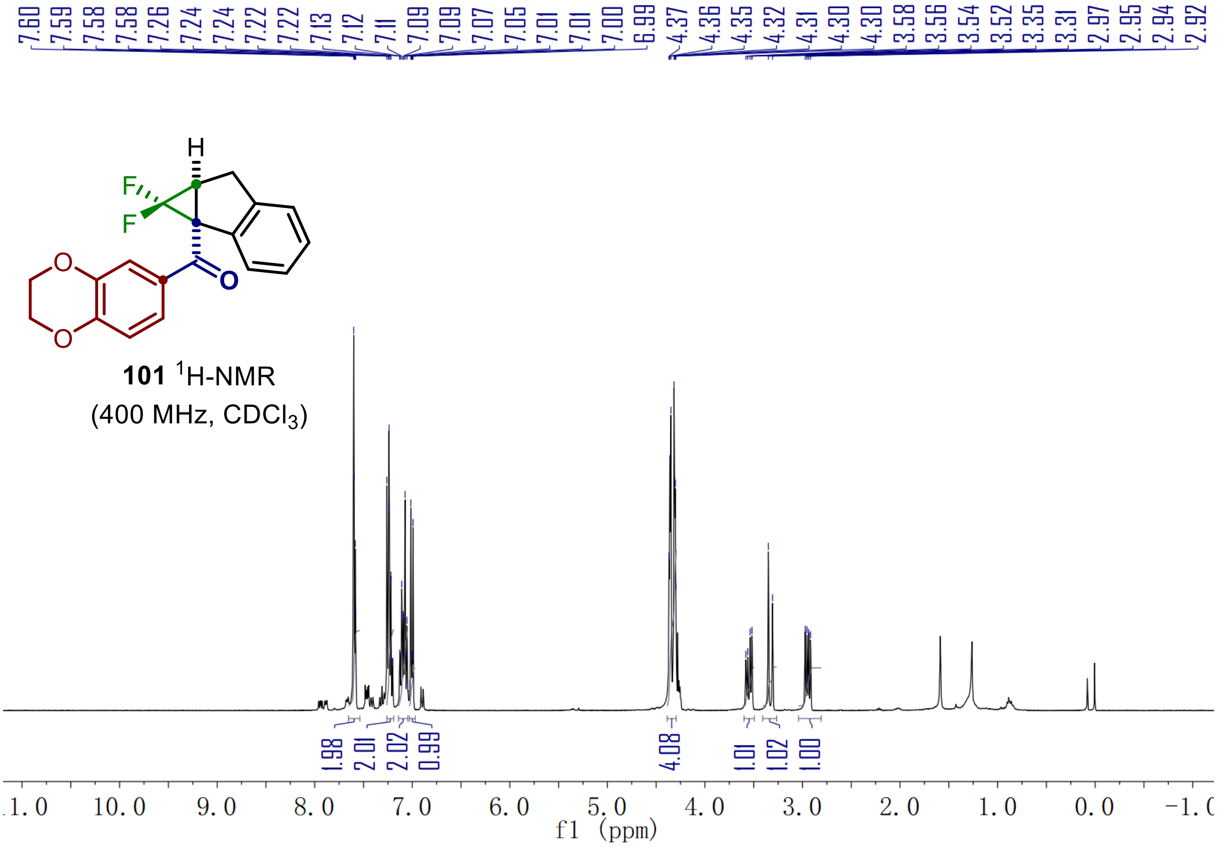
7.60
7.59
7.58
7.58
7.26
7.24
7.24
7.22
7.22
7.13
7.12
7.11
7.09
7.09
7.07
7.05
7.01
7.01
7.00
6.99
4.37
4.36
4.35
4.32
4.31
4.30
4.30
3.58
3.56
3.54
3.52
3.35
3.31
2.97
2.95
2.94
2.92
H
F
F
O
O
O
101 ^{1}H-NMR
(400 MHz, $CDCl_3$)
1.98
2.01
2.02
0.99
4.08
1.01
1.02
1.00
f1 (ppm)

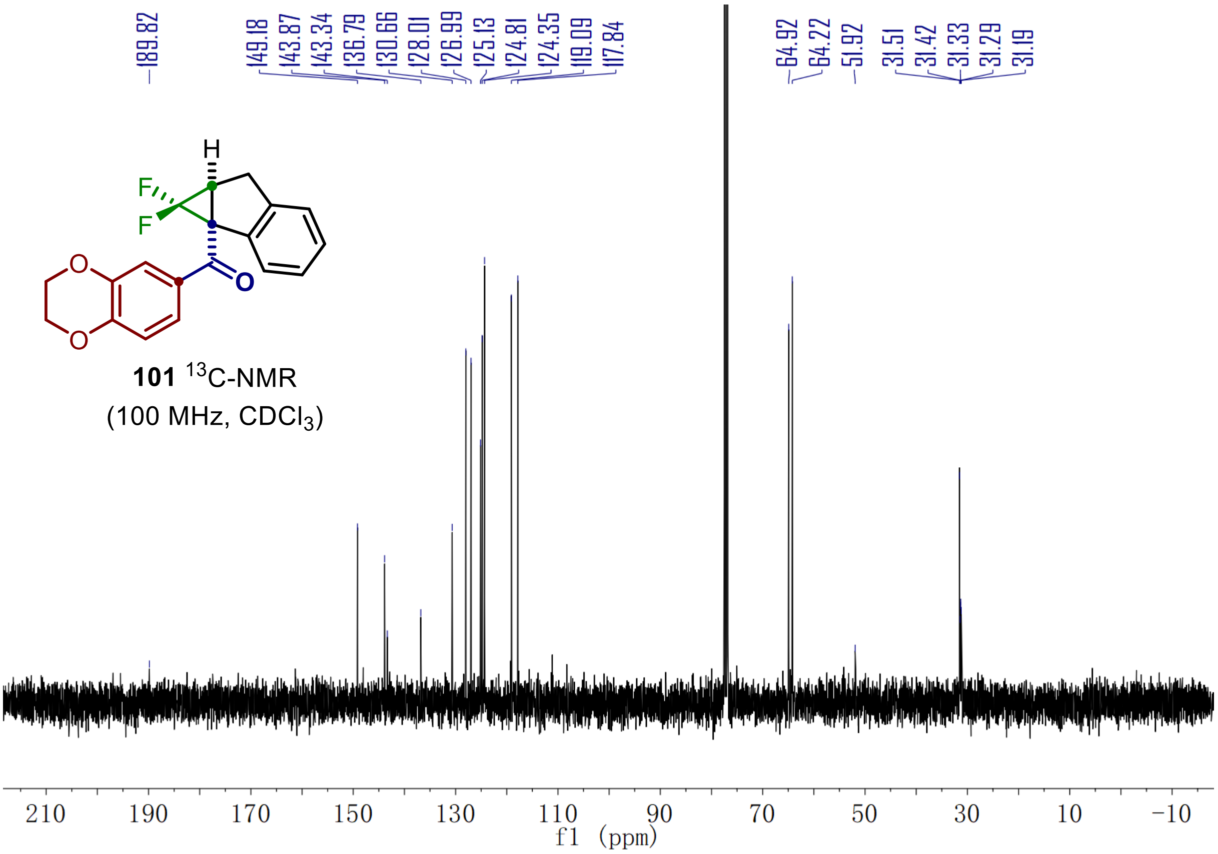
189.82
149.18
143.87
143.34
136.79
130.66
128.01
126.99
125.13
124.81
124.35
119.09
117.84
64.92
64.22
51.92
31.51
31.42
31.33
31.29
31.19
H
F
F
O
O
O
101 ^{13}C-NMR
(100 MHz, CDCl$_3$)
210
190
170
150
130
110
90
70
50
30
10
−10
f1 (ppm)

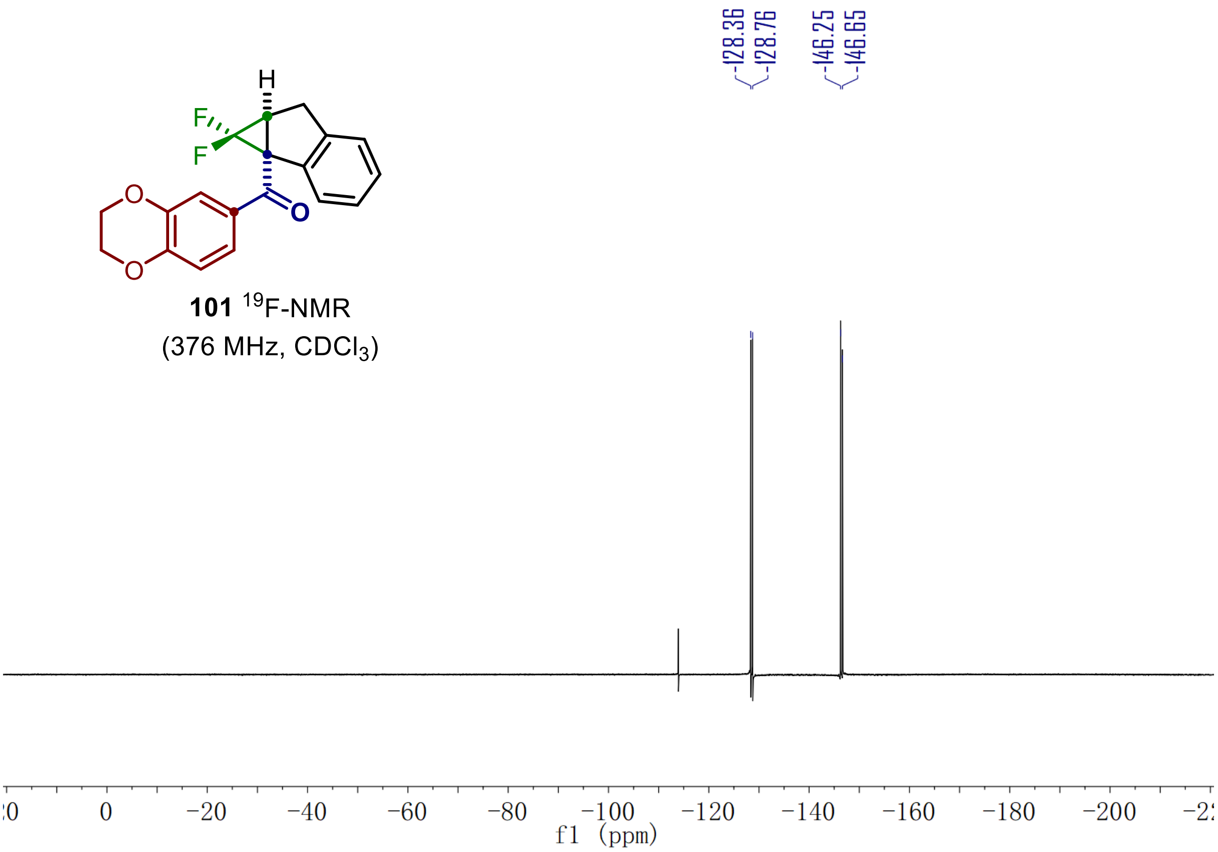
-128.36
-128.76
-146.25
-146.65
H
F
F
O
O
O
101 ^{19}F-NMR
(376 MHz, CDCl$_3$)
0
−20
−40
−60
−80
−100
−120
−140
−160
−180
−200
f1 (ppm)

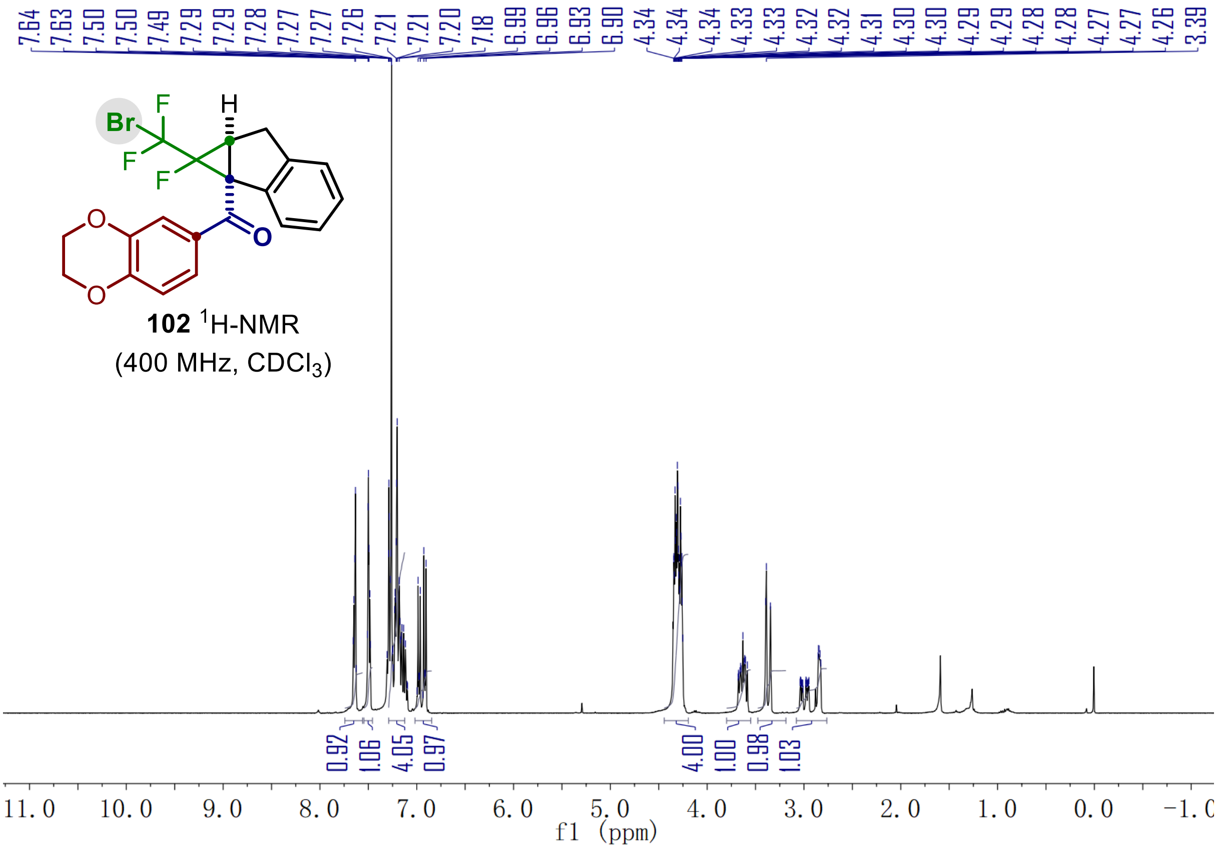
102 ¹H-NMR
(400 MHz, CDCl3)
f1 (ppm)

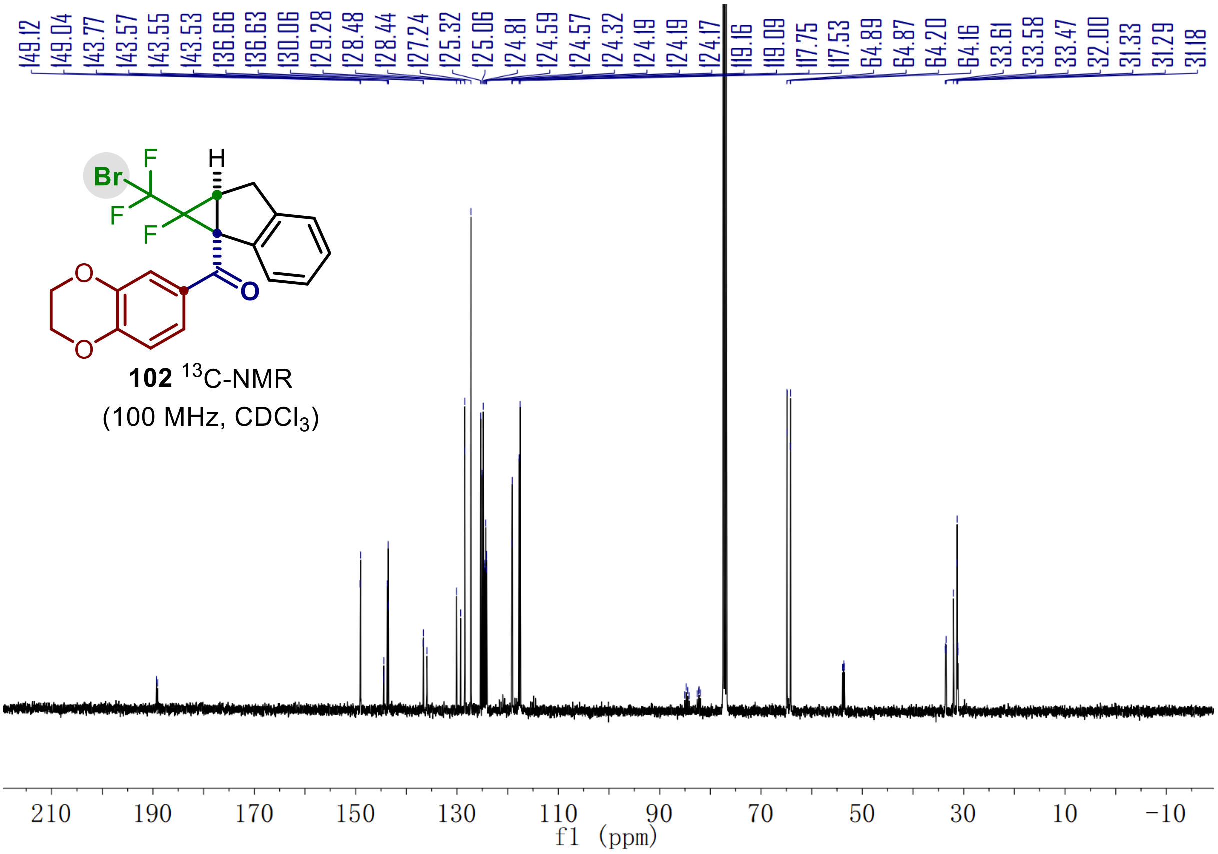
102 ¹³C-NMR
(100 MHz, CDCl3)
f1 (ppm)

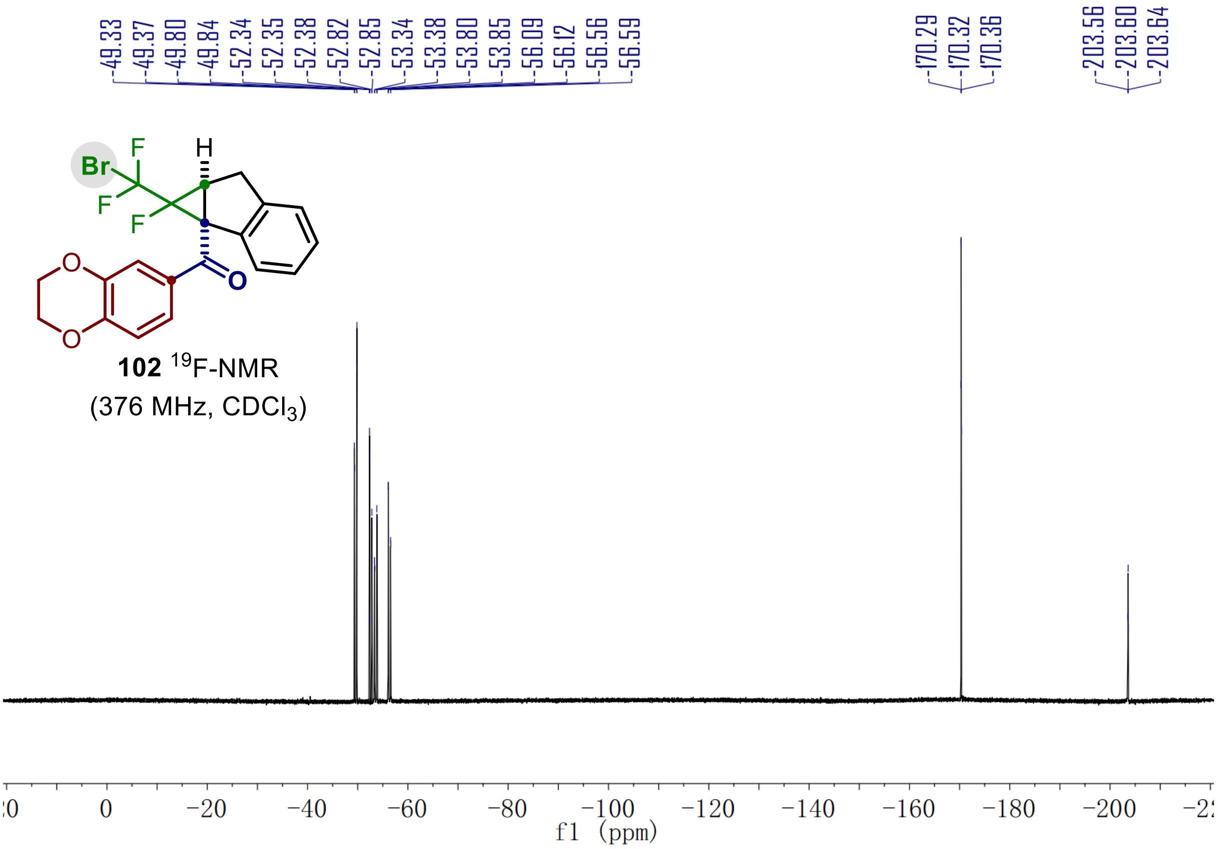
-49.33
-49.37
-49.80
-49.84
-52.34
-52.35
-52.38
-52.82
-52.85
-53.34
-53.38
-53.80
-53.85
-56.09
-56.12
-56.56
-56.59
-170.29
-170.32
-170.36
-203.56
-203.60
-203.64
Br
F
F
F
H
O
O
O
102 19F-NMR
(376 MHz, CDCl3)
0
−20
−40
−60
−80
−100
−120
−140
−160
−180
−200
f1 (ppm)

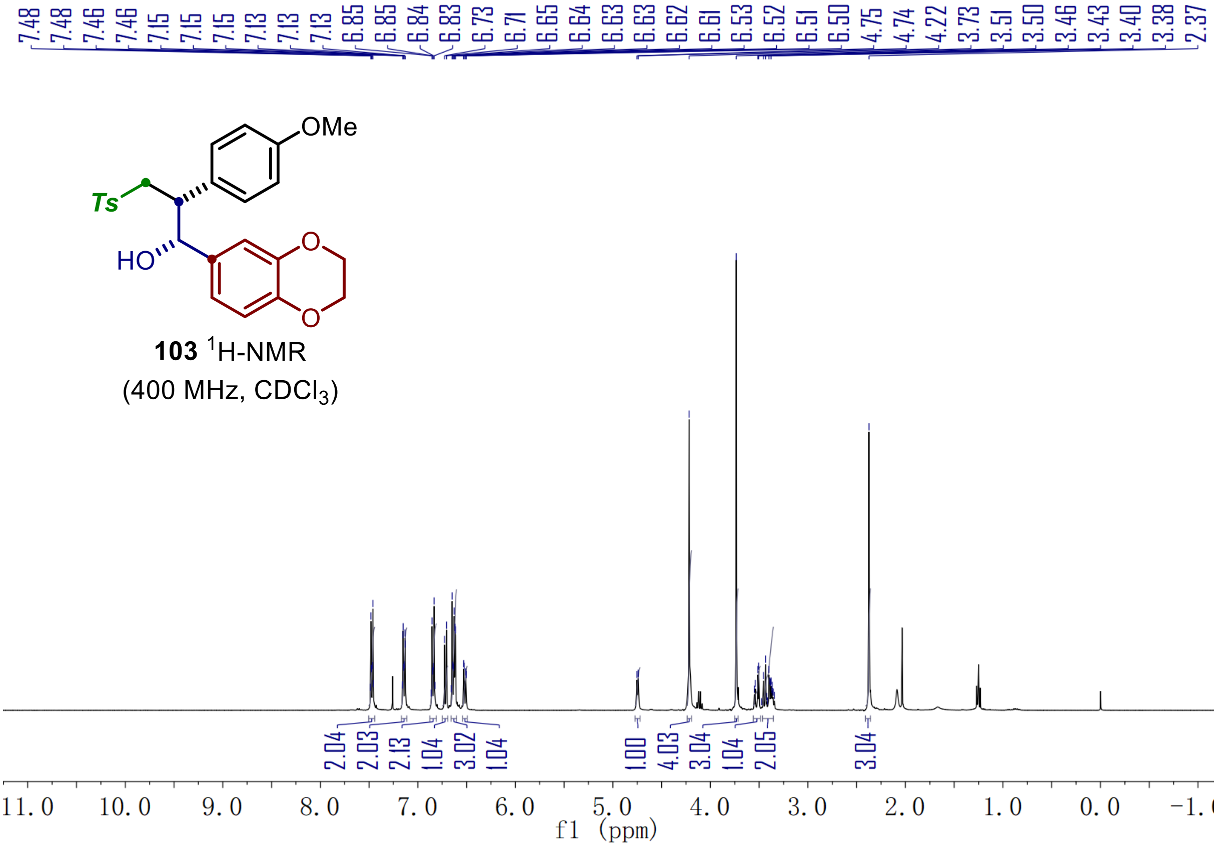
7.48
7.48
7.46
7.46
7.15
7.15
7.15
7.13
7.13
7.13
6.85
6.85
6.84
6.83
6.73
6.71
6.65
6.64
6.63
6.63
6.62
6.61
6.53
6.52
6.51
6.50
4.75
4.74
4.22
3.73
3.51
3.50
3.46
3.43
3.40
3.38
2.37
OMe
Ts
HO
O
O
103 1H-NMR
(400 MHz, CDCl3)
2.04
2.03
2.13
1.04
3.02
1.04
1.00
4.03
3.04
1.04
2.05
3.04
11.0
10.0
9.0
8.0
7.0
6.0
5.0
4.0
3.0
2.0
1.0
0.0
f1 (ppm)

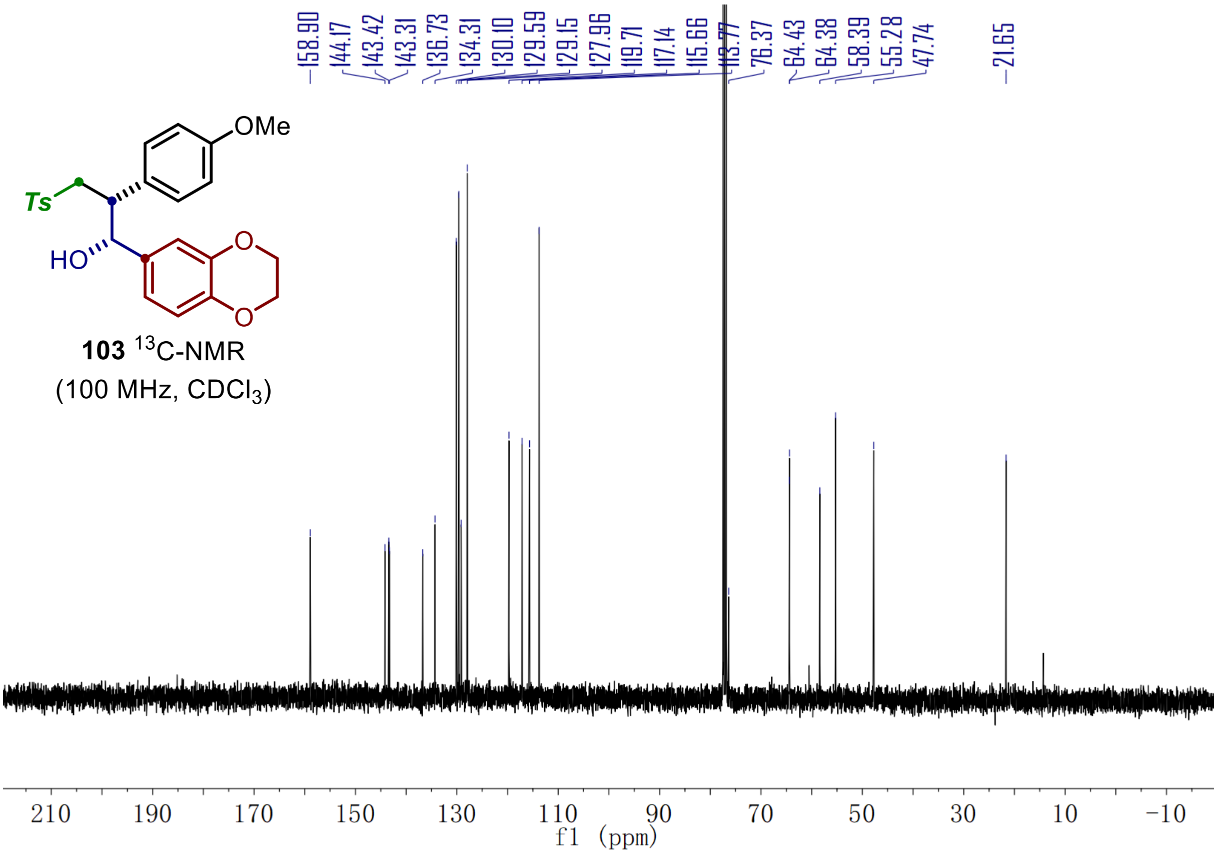

158.90
144.17
143.42
143.31
136.73
134.31
130.10
129.59
129.15
127.96
119.71
117.14
115.66
113.77
76.37
64.43
64.38
58.39
55.28
47.74
21.65
OMe
Ts
HO
O
O
103 ¹³C-NMR
(100 MHz, CDCl3)
210
190
170
150
130
110
90
70
50
30
10
−10
f1 (ppm)

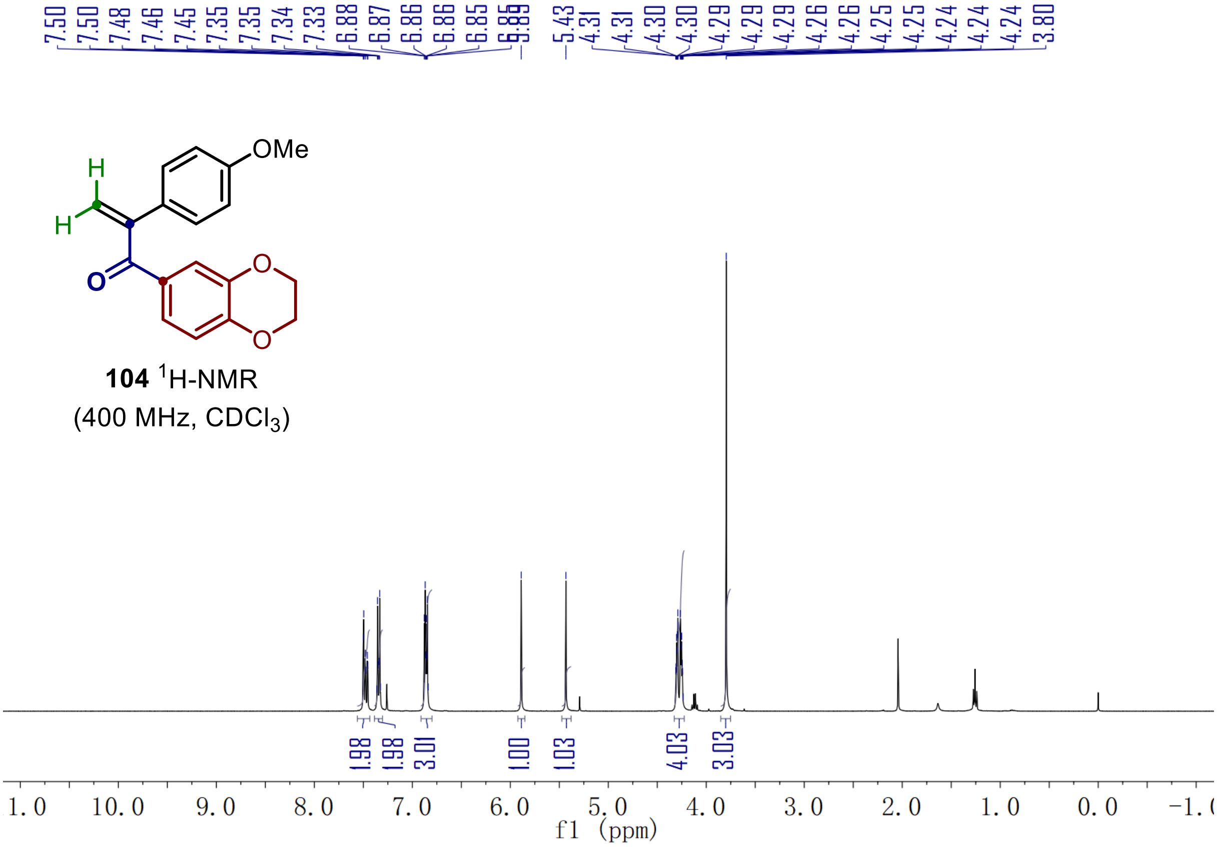

7.50
7.50
7.48
7.46
7.45
7.35
7.35
7.34
7.33
6.88
6.87
6.86
6.86
6.85
5.89
5.43
4.31
4.31
4.30
4.30
4.29
4.29
4.29
4.26
4.26
4.25
4.25
4.24
4.24
4.24
3.80
H
H
OMe
O
O
O
104 ¹H-NMR
(400 MHz, CDCl3)
1.98
1.98
3.01
1.00
1.03
4.03
3.03
10.0
9.0
8.0
7.0
6.0
5.0
4.0
3.0
2.0
1.0
0.0
f1 (ppm)

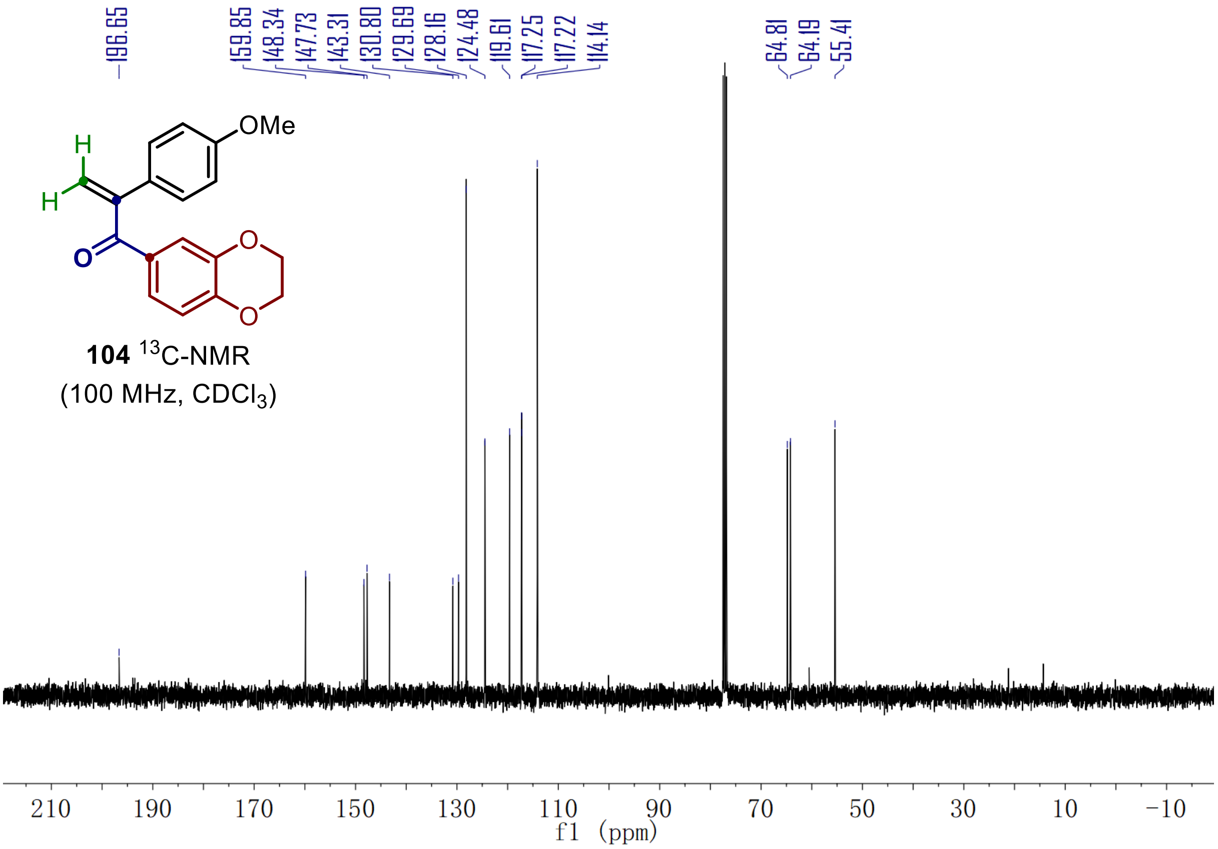

104 ^{13}C-NMR
(100 MHz, $CDCl_3$)

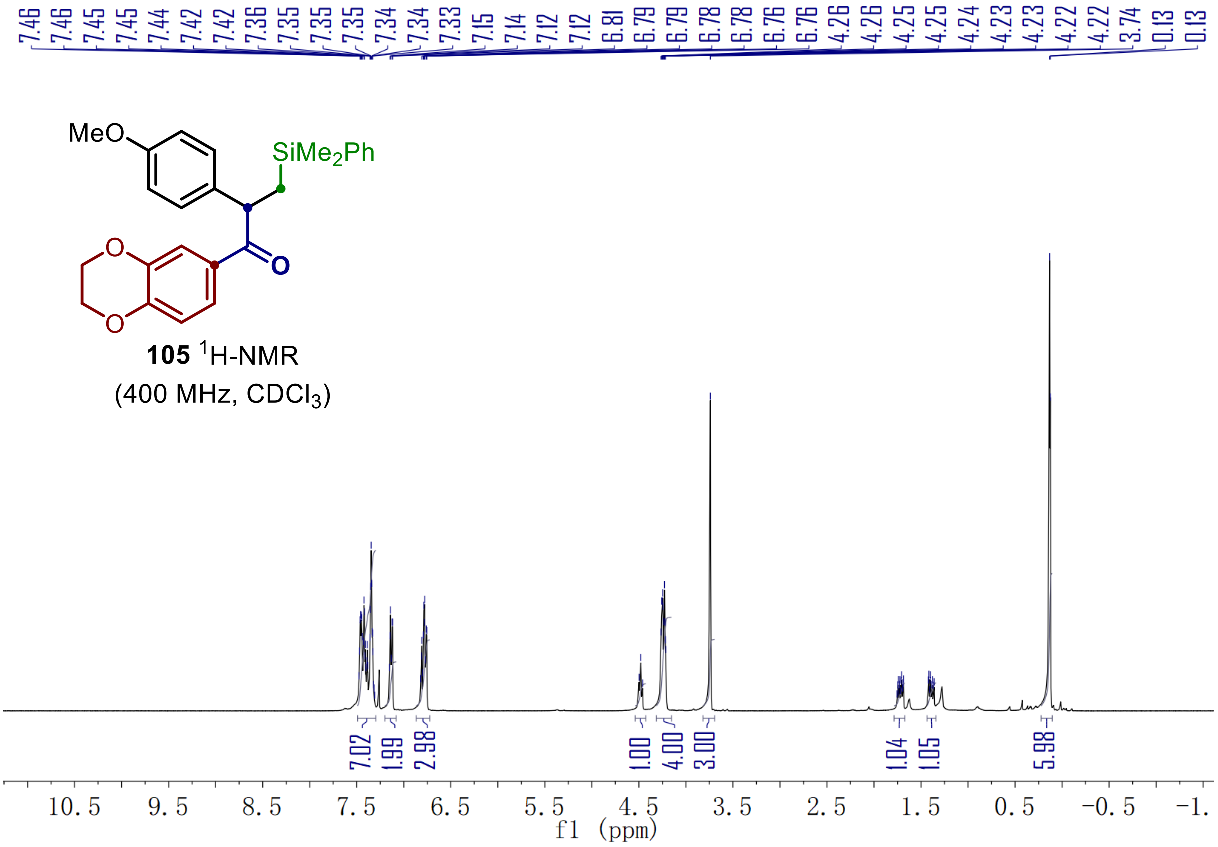

105 ^{1}H-NMR
(400 MHz, $CDCl_3$)

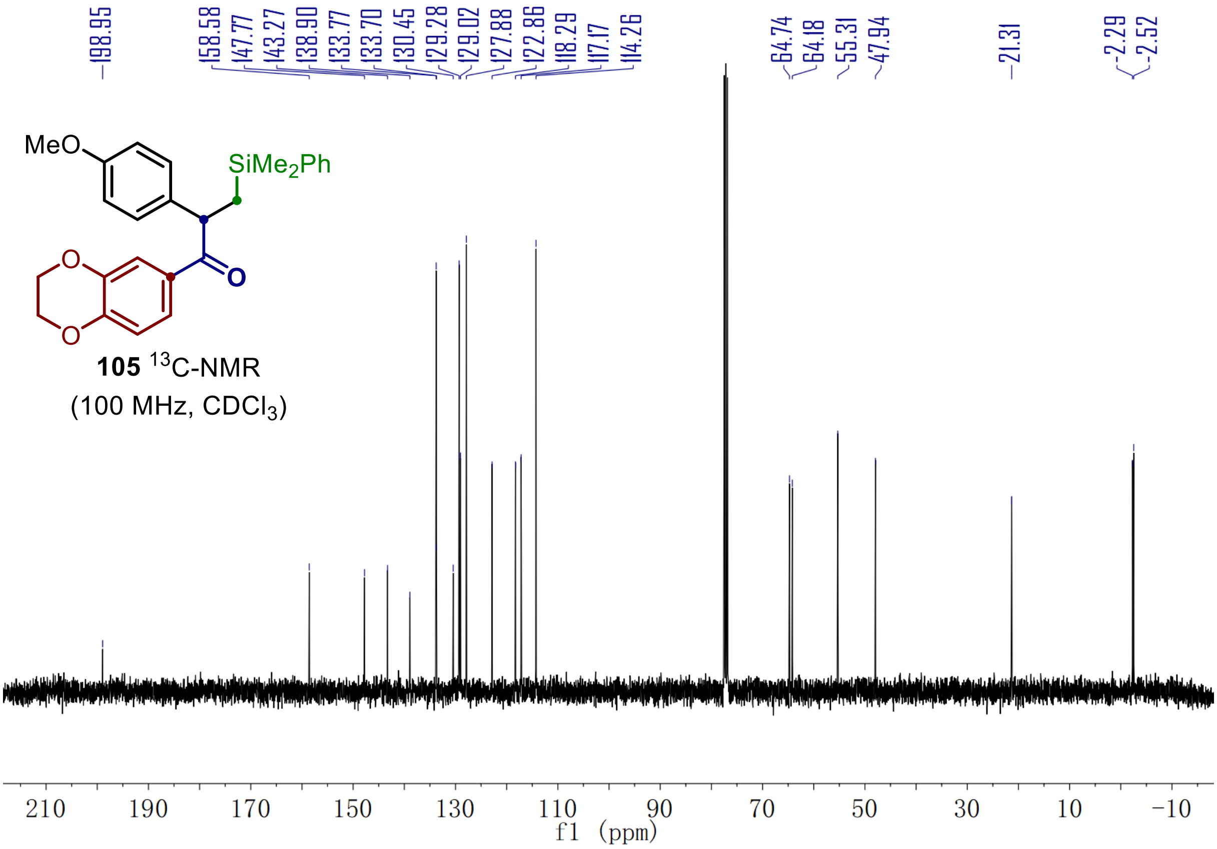
198.95
158.58
147.77
143.27
138.90
133.77
133.70
130.45
129.28
129.02
127.88
122.86
118.29
117.17
114.26
64.74
64.18
55.31
47.94
21.31
-2.29
-2.52
MeO
SiMe2Ph
O
O
O
105 13C-NMR
(100 MHz, CDCl3)
f1 (ppm)

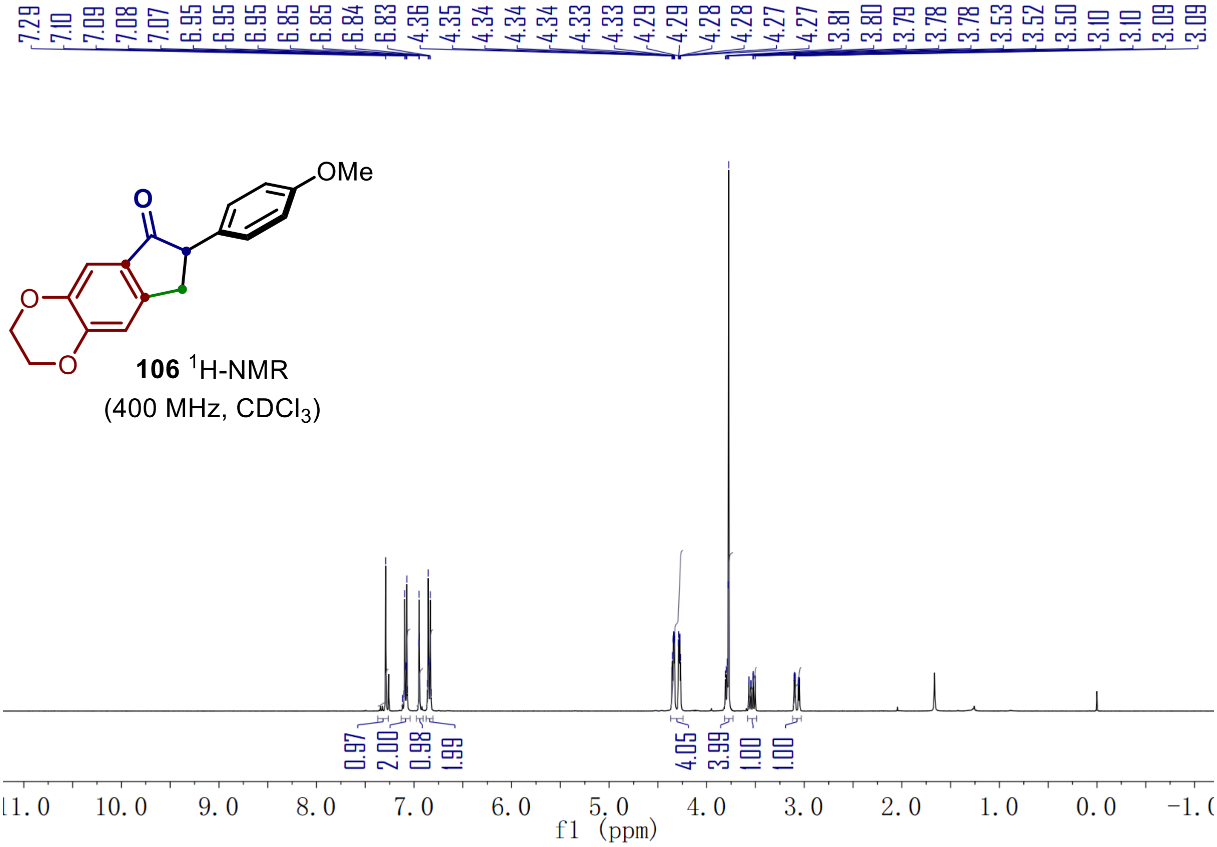
OMe
O
O
O
106 1H-NMR
(400 MHz, CDCl3)
0.97
2.00
0.98
1.99
4.05
3.99
1.00
1.00
f1 (ppm)

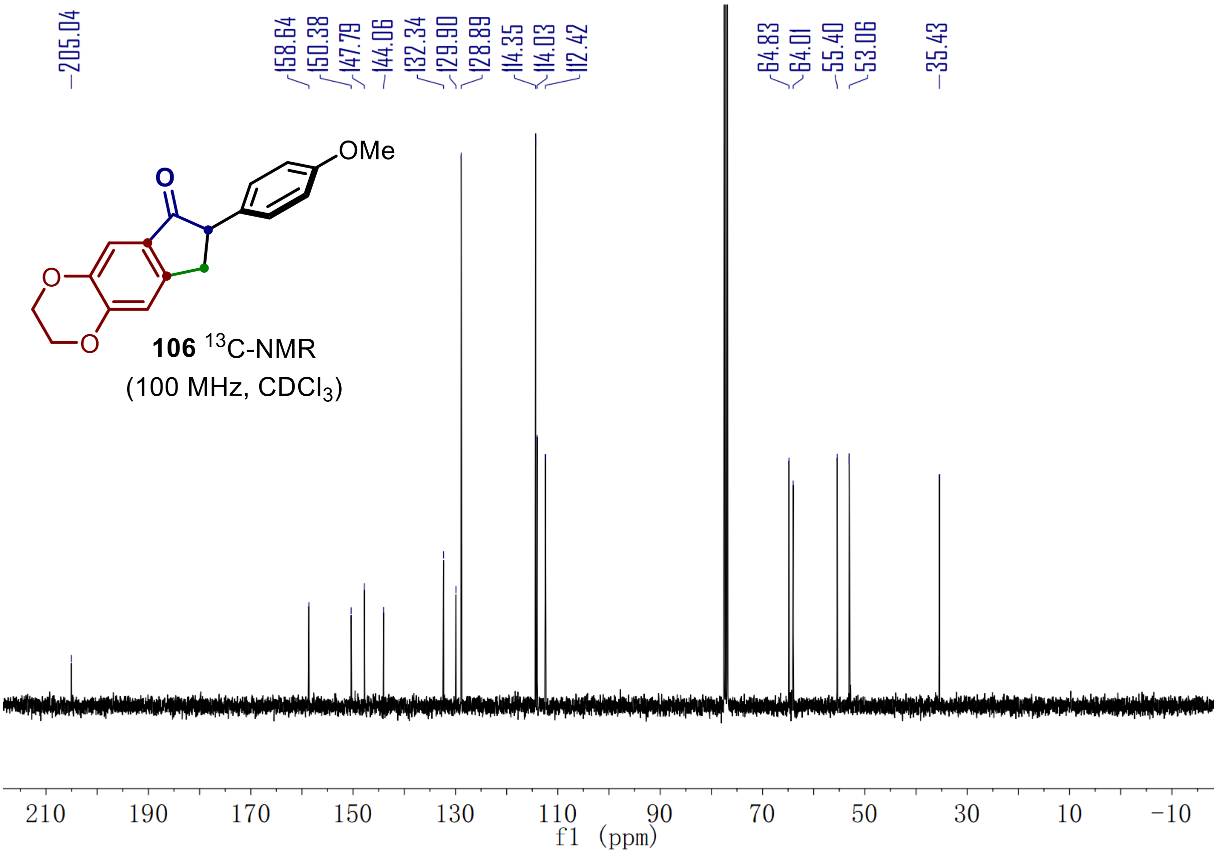

205.04
158.64
150.38
147.79
144.06
132.34
129.90
128.89
114.35
114.03
112.42
64.83
64.01
55.40
53.06
35.43
OMe
O
O
O
106 13C-NMR
(100 MHz, CDCl3)
210
190
170
150
130
110
90
70
50
30
10
−10
f1 (ppm)

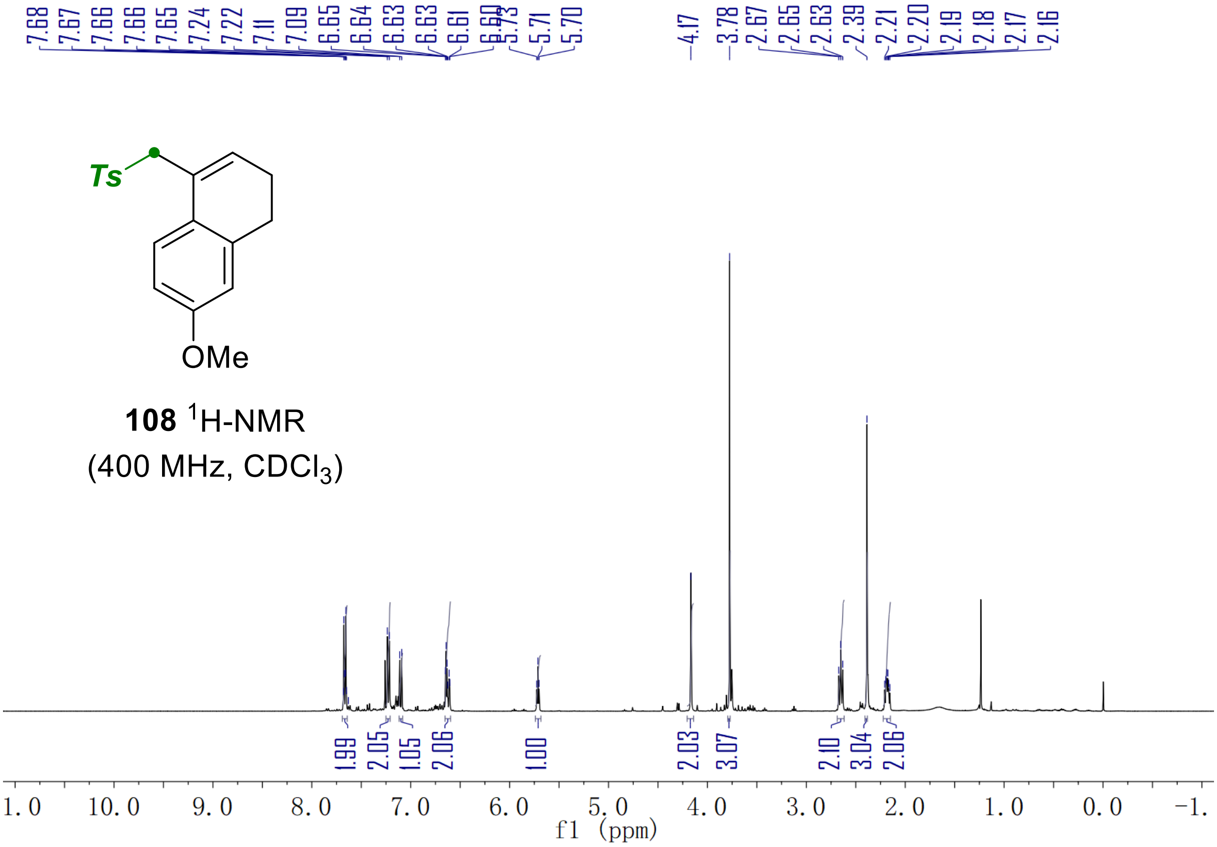

7.68
7.67
7.66
7.66
7.65
7.24
7.22
7.11
7.09
6.65
6.64
6.63
6.63
6.61
5.73
5.71
5.70
4.17
3.78
2.67
2.65
2.63
2.39
2.21
2.20
2.19
2.18
2.17
2.16
Ts
OMe
108 1H-NMR
(400 MHz, CDCl3)
1.99
2.05
1.05
2.06
1.00
2.03
3.07
2.10
3.04
2.06
1.0
10.0
9.0
8.0
7.0
6.0
5.0
4.0
3.0
2.0
1.0
0.0
−1.
f1 (ppm)

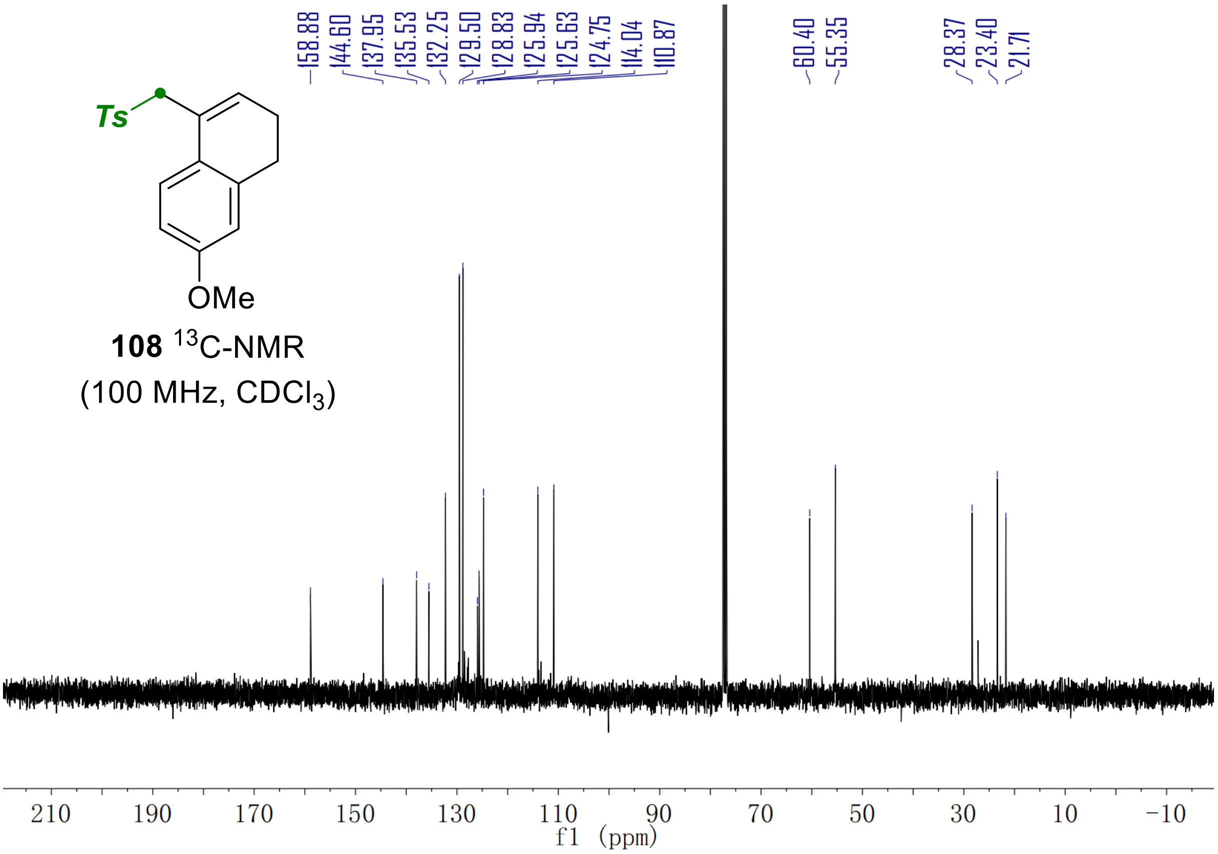
158.88
144.60
137.95
135.53
132.25
129.50
128.83
125.94
125.63
124.75
114.04
110.87
60.40
55.35
28.37
23.40
21.71
Ts
OMe
108 ^{13}C-NMR
(100 MHz, $CDCl_3$)
210 190 170 150 130 110 90 70 50 30 10 −10
f1 (ppm)

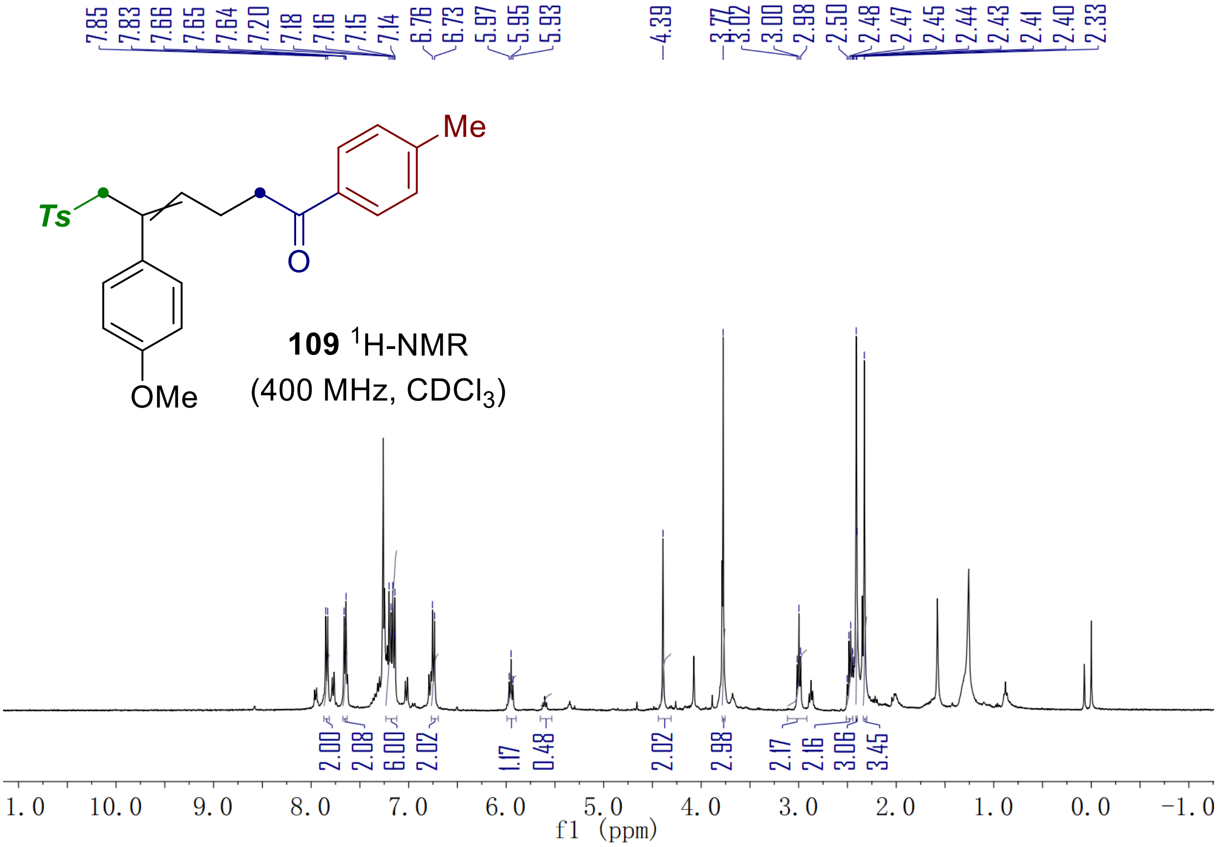
7.85
7.83
7.66
7.65
7.64
7.20
7.18
7.16
7.15
7.14
6.76
6.73
5.97
5.95
5.93
4.39
3.77
3.02
3.00
2.98
2.50
2.48
2.47
2.45
2.44
2.43
2.41
2.40
2.33
Me
Ts
O
OMe
109 ^{1}H-NMR
(400 MHz, $CDCl_3$)
2.00
2.08
6.00
2.02
1.17
0.48
2.02
2.98
2.17
2.16
3.06
3.45
1.0 10.0 9.0 8.0 7.0 6.0 5.0 4.0 3.0 2.0 1.0 0.0 −1.0
f1 (ppm)

13. References

[1] L. Xiao, M. Bhadbhade, A. T. Baker, Reversible Five-coordinate Six-coordinate Transformation in Cobalt(II) Complexes. *J. Mol. Struct*. **2018**, *1157*, 112-118.

[2] H. Motohashi, M. Kato, K. Mikami, Ligand-Less Iron-Catalyzed Aromatic Cross-Coupling Difluoromethylation of Grignard Reagents with Difluoroiodomethane. *J. Org. Chem*. **2019**, *84*, 6483-6490.

[3] F. Berger, M. B. Plutschack, J. Riegger, W. Yu, S. Speicher, M. Ho, N. Frank, T. Ritter, Site-Selective and Versatile Aromatic C−H Functionalization by Thianthrenation. *Nature* **2019**, *567*, 223-228.

[4] J. Wang, S. Wang, Z. Wei, P. Wang, Y. Cao, Y. Huang, L. He, A. Lei, Synchronous Recognition of Amines in Oxidative Carbonylation Toward Unsymmetrical Ureas. *Science* **2024**, *386*,776-782.

[5] a) J. Li, Z. Liang, Y. Ren, J. Gao, D. Du, Facile Access to Gem-difluorocyclopropanes via An N-heterocyclic Carbene-Catalyzed Radical Relay/Cyclization Strategy. *Org. Chem. Front*. **2023**, *10*, 1669-1674. b) M. Kusakabe, K. Nagao, H. Ohmiya, Radical Relay Trichloromethylacylation of Alkenes through N-Heterocyclic Carbene Catalysis. *Org. Lett*. **2021**, *23*, 7242-7247.

[6] M. Liu, L. Min, B. Chen, W. Shu, Dual Catalysis Relay: Coupling of Aldehydes and Alkenes Enabled by Visible-Light and NHC-Catalyzed Cross-Double C−H Functionalizations. *ACS Catal*. **2021**, *11*, 9715-9721.

[7] O. Martin, W. Barbara, Copper-Catalyzed Conjugate Addition of A Bis(triorganosilyl) zinc and A Methyl(triorganosilyl) Magnesium. *Synlett* **2004**, *12*, 2139-2142.

[8] a) Y. Chen, K. Zhu, Q. Huang, Y. Lu, Regiodivergent Sulfonylarylation of 1,3-Enynes via Nickel/Photoredox Dual Catalysis. *Chem. Sci*. **2021**, *12*, 13564-13571. b) B. S. Martins, D. Kaiser, A. Bauer, I. Tiefenbrunner, N. Maulide, Formal Enone α-Arylation via I(III)-Mediated Aryl Migration/Elimination. *Org. Lett*. **2021**, *23*, 2094-2098.

[9] M. J. Frisch, G. W. Trucks, H. B. Schlegel, G. E. Scuseria, M. A. Robb, J. R. Cheeseman, G. Scalmani, V. Barone, B. Mennucci, G. A. Petersson, H. Nakatsuji, M. Caricato, X. Li, H. P. Hratchian, A. F. Izmaylov, J. Bloino, G. Zheng, J. L. Sonnenberg, M. Hada, M. Ehara, K. Toyota, R. Fukuda, J. Hasegawa, M. Ishida, T. Nakajima, Y. Honda, O. Kitao, H. Nakai, T. Vreven, J. A. Jr. Montgomery, J. E. Peralta, F. Ogliaro, M. Bearpark, J. J. Heyd, E. Brothers, K. N. Kudin, V. N. Staroverov, R. Kobayashi, J. Normand, K. Raghavachari, A. Rendell, J. C. Burant, S. S. Iyengar, J. Tomasi, M. Cossi, N. Rega, N. J. Millam, M. Klene, J. E. Knox,

J. B. Cross, V. Bakken, C. Adamo, J. Jaramillo, R. Gomperts, R. E. Stratmann, O. Yazyev, A. J. Austin, R. Cammi, C. Pomelli, J. W. Ochterski, R. L. Martin, K. Morokuma, V. G. Zakrzewski, G. A. Voth, P. Salvador, J. J. Dannenberg, S. Dapprich, A. D. Daniels, Ö. Farkas, J. B. Foresman, J. V. Ortiz, J. Cioslowski, D. J. Fox, Gaussian 16, Revision A.03; Gaussian, Inc., Wallingford, CT, 2016.

[10] A. D. Becke, Density-functional Thermochemistry. III. The Role of Exact Exchange. *J. Chem. Phys.* **1993**, 98, 5648-5652.

[11] M. Dolg, U. Wedig, H. Stoll, H. Preuss, Energy-Adjusted ab initio Pseudopotentials for the First Row Transition Elements. *J. Chem. Phys.* **1987**, *86*, 866-872.

[12] Y. Zhao, D. G. Truhlar, The M06 Suite of Density Functionals for Main Group Thermochemistry, Thermochemical Kinetics, Noncovalent Interactions, Excited States, and Transition Elements: Two New Functionals and Systematic Testing of Four M06-Class Functionals and 12 Other Functionals. *Theor. Chem. Acc.* **2008**, *120*, 215-241.

[13] A. V. Marenich, C. J. Cramer, D. G. Truhlar, Universal Solvation Model Based on Solute Electron Density and on a Continuum Model of the Solvent Defined by the Bulk Dielectric Constant and Atomic Surface Tensions. *J. Phys. Chem. B*. **2009**, *113*, 6378-6396.
